# Supplementary material for: Asymmetric synthesis of P-stereogenic phosphindane oxides via kinetic resolution and their biological activity
Source: Nat Commun. 2024 Mar 21;15:2548. doi: 10.1038/s41467-024-46892-7 (PMC10957969; doi:10.1038/s41467-024-46892-7)
Supplement: Supplementary file 1 — Supplementary Information [file 41467_2024_46892_MOESM1_ESM.pdf]

Supplementary Information

Asymmetric Synthesis of *P*-Stereogenic Phosphindane Oxides via Kinetic Resolution and Their Biological Activity

Long Yin,[1,3] Jiajia Li,[1,3] Changhui Wu,[2,3] Haoran Zhang,[1] Wenchao Zhao,[1] Zhiyuan Fan,[1] Mengxuan Liu,[1] Siqi Zhang,[1] Mengzhe Guo,[1,]* Xiaowei Dou[2,]* & Dong Guo[1,]*

[1]Jiangsu Key Laboratory of New Drug Research and Clinical Pharmacy, Xuzhou Medical University, Xuzhou, 221004, China

[2]Department of Chemistry, School of Science, China Pharmaceutical University, Nanjing, 211198, China

[3]These authors contributed equally: Long Yin, Jiajia Li, Changhui Wu.

E-mail: guo@xzhmu.edu.cn; dxw@cpu.edu.cn; guomengzhe@xzhmu.edu.cn

Table of Contents

1. Supplementary Notes........3

2. Supplementary Methods........4

2.1 Preparation of catalyst $[RhCl(L4)]_2$........4

2.2 General procedure for the synthesis of substrates........7

2.3 Experimental section........9

2.3.1 A general procedure for Table 1........9

2.3.2 A general procedure for Figure 2........9

2.3.3 Synthetic applications........10

2.3.4 Synthesis of *ent*-3az........12

2.3.5 Mechanistic investigations........12

2.4 Product characterization........16

2.5 DFT calculation........48

2.6 Biological activity study........53

2.7 Proteomic analysis........57

2.8 Molecular docking........60

2.9 Single crystal X-ray diffraction........60

3. Supplementary Figures........65

3.1 NMR spectra........65

3.2 HPLC charts........155

4. Supplementary References........216

1. Supplementary Notes

Unless otherwise mentioned, all air-sensitive manipulations were carried out with standard Schlenk techniques under nitrogen or argon. Solvents and reagents were purchased from commercial suppliers and used without further purification. Chemical shifts were reported in δ (ppm) referenced to the residual solvent peak of $CHCl_3$-*d* (δ 7.26) for ^1H NMR and $CHCl_3$-*d* (δ 77.0) for ^{13}C NMR, the residual solvent peak of acetone-d_6 (δ 2.05) for ^1H NMR and acetone-d_6 (δ 29.84, 206.26) for ^{13}C NMR, the residual solvent peak of DMSO-d_6 (δ 2.50) for ^1H NMR and acetone-d_6 (δ 39.52) for ^{13}C NMR. Multiplicity was indicated as follows: s (singlet), d (doublet), t (triplet), q (quartet), dd (doublet of doublets), dt (doublet of triplets), dq (doublet of quartets), td (triplet of doublets), tt (triplet of triplets), ddd (doublet of doublet of doublets), ddt (doublet of doublet of triplets), dtd (doublet of triplet of doublets), m (multiplet), br (broad), etc. Coupling constants were reported in Hertz (Hz). Enantiomeric excesses (*ee*) were determined by HPLC analysis on Shimadzu HPLC system with Daicel chiral columns. Optical rotations were measured on an Anton Paar MCP 100 automatic polarimeter. High resolution mass spectra (HRMS) were performed on Waters XEVO G2-S TOF or Agilent G6550A Q-TOF (ESI). Single crystal X-ray diffraction were obtained on a Bruker D8 Quest diffractometer. For thin layer chromatography (TLC), Yantai pre-coated TLC plates (HSGF 254) were used, and compounds were visualized with a UV light at 254 nm. Further visualization was achieved by staining with $KMnO_4$ followed by heating. Column chromatography separations were performed on silica gel (300–400 mesh).

The solvents (Toluene, 1,4-Dioxane, THF, EtOAc, Hexane, *i*PrOH, EtOH and MeOH, AR grade) used in the kinetic resolution study were purchased from commercial supplier and degassed with N_2 before use. All the organoboronic acids were purchased from commercial suppliers and used as received.

2. Supplementary Methods

The catalyst [RhCl(**L1**)]$_2$ was prepared according to the reported procedures.[1] The catalysts [RhCl(**L2**)]$_2$ and [RhCl(**L5**)]$_2$ were prepared according to the reported procedures.[2] The catalysts [RhCl(**L3**)]$_2$ and [RhCl(**L4**)]$_2$ were prepared according to the reported procedures.[3] The catalyst [RhCl(**L6**)]$_2$ was prepared according to the reported procedures.[4] The catalyst [RhCl(***(R,R)*-Ph-bod**)]$_2$ was prepared according to the reported procedures.[5]

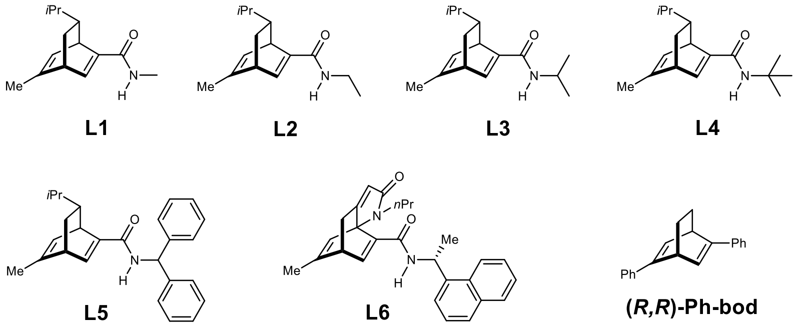

Supplementary Figure 1. Selected chiral diene ligands

2.1 Preparation of catalyst [RhCl(L4)]$_2$

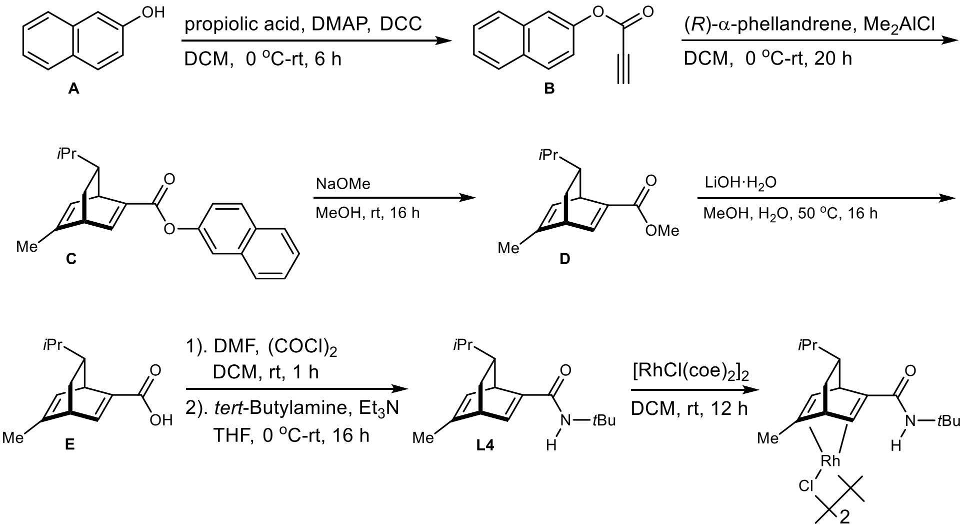

Supplementary Figure 2. Procedure for synthesis of catalyst [RhCl(L4)]$_2$

step 1. To a solution of 2-naphthol (20.0 g, 138.8 mmol, 1.0 equiv) and DMAP (169.8 mg, 1.39 mmol) in dichloromethane (300.0 mL) was added propiolic acid (10.7 g, 152.7 mmol, 1.1 equiv) and subsequently dicyclohexylcarbodiimide (DCC, 31.5 g,

152.7 mmol, 1.1 equiv) at 0 ^{o}C. The mixture was allowed to warm to room temperature and stirred for 6 h. The precipitates were filtered off and the filtrate was concentrated under vacuum. The residue was chromatographed on silica gel (petroleum ether/EtOAc = 20/1) to afford white solid **B** (22.9 g, 116.6 mmol, 84% yield).

step 2. To a solution of (*R*)-α-phellandrene (~85% chemical purity, 9.0 g, 56.1 mmol, 1.1 equiv) and **B** (10.0 g, 51.0 mmol, 1.0 equiv) in CH_2Cl_2 (150.0 mL) was added Me_2AlCl (1.0 M in hexane, 57.0 mL, 56.1 mmol, 1.1 equiv) slowly at -78 ^{o}C. The resulting orange solution was allowed to sit in the cold bath and slowly warm to room temperature. After stirring for 18 h, the solution was carefully poured into a vigorously stirred, ice-cooled aqueous solution of 2N HCl (100.0 mL). The mixture was filtered and washed with 50 mL of dichloromethane. The filtrate was then extracted with CH_2Cl_2 (50.0 mL × 3). The combined organic extracts were washed with brine (180.0 mL), dried over $MgSO_4$, filtered, and concentrated under vacuum. The residue was chromatographed on silica gel (petroleum ether/EtOAc = 25/1) to give 15.9 g of a mixture of **C** and (*E*)-2-naphthyl 3-(5-isopropyl-2-methylenecyclohex-3-enyl)propenoate. The mixture was diluted with 2.5 mL of dichloromethane and 36 mL of hexane. The flask was placed at room temperature overnight. The crystals precipitated were collected by filtration and washed with 5 mL of ice-cooled hexane, and then dried under vacuum to give 7.4 g of **C** (22.3 mmol, >99% ee) as a white needle. The mother liquor was subject to the same procedure using 1.0 mL of dichloromethane and 15 mL of hexane to give **C** (3.1 g, 9.3 mmol, >99% ee).

step 3. A solution of **C** (664.0 mg, 2.0 mmol, 1.0 equiv) and NaOMe (216.0 mg, 4.0 mmol, 2.0 equiv) in MeOH (5.0 mL) was stirred at room temperature for 16 h. The reaction mixture was concentrated under vacuum. The residue was chromatographed on silica gel (petroleum ether/EtOAc = 20/1) to give **D** (436.1 mg, 1.98 mmol, 99% yield) as a colorless oil.

step 4. A solution of methyl ester **D** (220.1 mg, 1.0 mmol, 1.0 equiv) and $LiOH\cdot H_2O$ (167.8 mg, 4.0 mmol, 4.0 equiv) in MeOH (5.0 mL) and H_2O (2.5 mL) was stirred at 50 ^{o}C for 16 h. It was cooled to room temperature and 4N HCl (10.0 mL) was added.

The mixture was extracted with $CHCl_3$ (10.0 mL × 3). The combined organic extracts were dried over $MgSO_4$, filtered, and concentrated under vacuum to give the crude carboxylic acid. The residue was chromatographed on silica gel (CH_2Cl_2/MeOH = 20/1) to give the desired acid **E** (197.9 mg, 0.96 mmol, 96% yield).

step 5. To a solution of the carboxylic acid **E** (206.0 mg, 1.0 mmol, 1.0 equiv) and DMF (15.0 μL) in DCM (5.0 mL) was added oxalyl chloride (0.8 mL, 2.0 mol/L in methylene chloride, 1.6 mmol, 1.6 equiv) dropwise. The resulting solution was stirred at rt for 1 h to generate the acid chloride. This solution was then transferred via syringe to a mixture of *tert*-butylamine (210.0 μL, 2.0 mmol, 2.0 equiv) and Et_3N (418.0 μL, 3.0 mmol, 3.0 equiv) in THF (5.0 mL) at 0 °C. Once the addition was complete, the mixture was stirred at room temperature for 20 h. Upon completion, the reaction mixture was diluted with EtOAc (20.0 mL) and water (10.0 mL). The layers were separated and the aqueous layer was extracted again with EtOAc for two more times (15.0 mL × 2). The combined organic extracts were washed with saturated aqueous NH_4Cl solution, dried over $MgSO_4$, filtered, and concentrated in vacuo. Purification of the residue by silica gel column chromatography (petroleum ether/EtOAc = 5/1) gave the chiral diene **L4** as a white solid (227.0 mg, 87% yield).

step 6. $[RhCl(coe)_2]_2$ (158.0 mg, 0.2 mmol, 1.0 equiv) and **L4** (115.0 mg, 0.44 mmol, 2.2 equiv) were placed in a Schlenk tube under nitrogen. Dichloromethane (4.0 mL) was added and the resulting solution was stirred at room temperature for 16 h. Upon completion, the crude mixture was directly purified by flash silica gel column chromatography using petroleum ether/EtOAc (v/v = 1/1) as eluent to give [RhCl(**L4**)]$_2$ as an orange solid (137.0 mg, 85% yield).

2.2 General procedure for the synthesis of substrates

Procedure A: Preparation of substrate 1a and 1h-1i

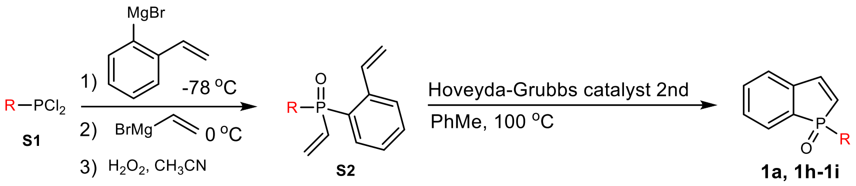

Supplementary Figure 3. Procedure for synthesis of compound 1a and 1h-1i

step 1. To a dry nitrogen-flushed two-neck flask charged with magnesium turnings (0.56 g, 23.0 mmol, 1.0 equiv), a solution of 2-bromostyrene (3.56 mL, 28.0 mmol, 1.2 equiv) in dry THF (30.0 mL) was added dropwise via syringe over 10 min at room temperature. The mixture was heated to 60 °C and stirred for 3 h until the magnesium was consumed. In a dry nitrogen-flushed two-neck flask, a solution of **S1** (23.0 mmol, 1.0 equiv) in dry THF (40.0 mL) was prepared. To this solution the above prepared Grignard solution was added dropwise via syringe over 30 min at -78 °C and a yellow color was noted upon addition. The mixture was allowed to warm to room temperature for 6 h. Then, the solution was cooled in an ice-water bath, and vinylmagnesium bromide solution (28.0 mL, 28.0 mmol, 1 M in THF, 1.2 equiv) was added via a dropping funnel over 30 min. The resulting mixture was allowed to warm to room temperature and stirred overnight. Upon completion, the mixture was concentrated under reduced pressure to a slurry, and it was diluted with DCM (100.0 mL) and water (10.0 mL). The layer was separated and the aqueous layer was extracted again with DCM for two more times (20.0 mL × 2). The combined organic extracts were washed with saturated aqueous NH_4Cl solution, dried over $MgSO_4$, filtered, and concentrated in vacuo. Subsequently, acetonitrile (50.0 mL) was added into the flask and the mixture was cooled in an ice-water bath. To the mixture was added H_2O_2 (4.0 equiv, 30% w/v) dropwise via syringe over 10 min, and the solution was stirred for 1 h. Acetonitrile was removed under reduced pressure, leaving a cloudy aqueous solution. It was extracted with DCM (70.0 mL × 2), and the combined organic layer was dried over $MgSO_4$ and

concentrated under reduced pressure. The residues were purified by column chromatography to afford **S2**.[6]

step 2. To a dry flask charged with nitrogen was added **S2** (10.0 mmol, 1.0 equiv) followed by dry toluene (50.0 mL). The solution was heated to 40 °C, and Hoveyda-Grubbs second generation catalyst (156.0 mg, 2.5 mol %) was added as a solid. The mixture was stirred overnight at 100 °C under nitrogen, which were filtered through a pad of Celite and concentrated. The residue was chromatographed on silica gel to give the compound **1a** and **1h-1i**.

Procedure B: Preparation of substrate 1b-1i and 1l-1m

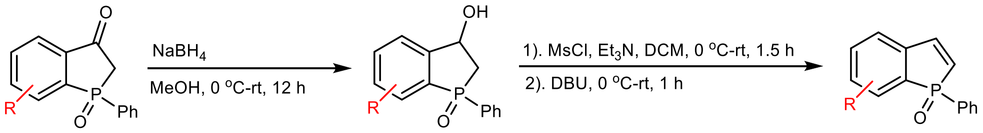

Supplementary Figure 4. Procedure for synthesis of compound 1b-1i and 1l-1m

step 1. To a solution of the 1-phenyl-2-hydrophosphindol-3-one 1-oxide **S3**[7] (1.5 mmol, 1.0 equiv) in methanol (5.0 mL) was added sodium borohydride (113.5 mg, 3.0 mmol, 2.0 equiv) in portions at 0 °C. The mixture was stirred for 30 minutes and warmed to rt. Then the mixture was stirred at the same temperature for 12 hours. After that, the mixture was concentrated in vacuo to give the crude **S4** (>95% yield).

step 2. To a stirred solution of **S4** (1.0 mmol, 1.0 equiv) in dichloromethane (5.0 mL) at 0 °C was added methanesulfonyl chloride (93.0 μL, 1.2 mmol, 1.2 equiv) and triethylamine (348.0 μL, 2.5 mmol, 2.5 equiv). The reaction was warmed up to room temperature over 30 minutes, and stirred for 1 h. Then, DBU (598.0 μL, 4.0 mmol, 4.0 equiv) was added and the mixture was stirred at 25 °C for another 1 h. The reaction was then quenched with 1N HCl (5.0 mL), and the mixture was extracted with CH_2Cl_2 (5.0 mL × 3). The combined organic layers were washed with brine, dried over $MgSO_4$, and concentrated in vacuo. The residue was purified by column chromatography to afford **1b-1i** and **1l-1m**.

2.3 Experimental section

2.3.1 A general procedure for Table 1

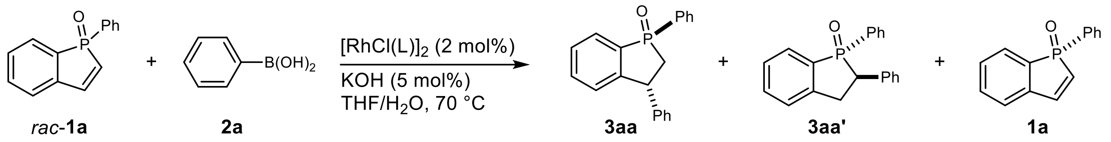

Rhodium catalysts (4.0 μmol, 2 mol%), *rac*-**1a** (45.2 mg, 0.20 mmol, 1.0 equiv) and **2a** (36.6 mg, 0.30 mmol, 1.5 equiv) were placed in an oven-dried Schlenk tube (25 mL) under nitrogen. Solvent (1.0 mL) and aqueous solution of KOH (0.01 mmol in 0.1 mL H_2O) were added and the resulting mixture was stirred at 70 °C for 20 h. Upon completion, the reaction mixture was diluted with EtOAc (6.0 mL) and water (4.0 mL). The layers were separated and the aqueous layer was extracted again with EtOAc for two more times (6.0 mL × 2). The combined organic layers were then concentrated in vacuo, and the residue was purified by silica gel chromatography eluting with petroleum ether/THF/EtOH (v/v/v = 80 : 20 : 1) to give **3aa** and **1a.**

2.3.2 A general procedure for Figure 2

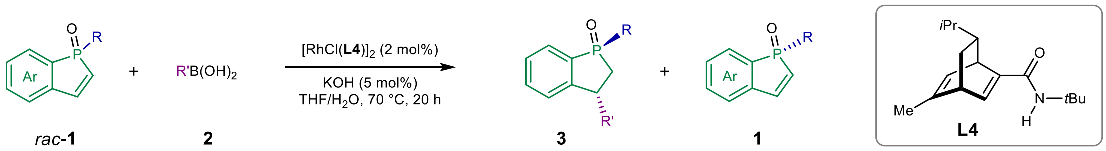

[RhCl(**L4**)]$_2$ (3.2 mg, 4.0 μmol, 2 mol%), *rac*-**1** (0.20 mmol, 1.0 equiv) and **2** (0.30 mmol, 1.5 equiv) were placed in an oven-dried Schlenk tube (25 mL) under nitrogen. THF (1.0 mL) and aqueous KOH (0.1 mL, 0.1 M, 5 mol%) were added and the resulting mixture was stirred at 70 °C for 20 h. Upon completion, the reaction mixture was diluted with EtOAc (6.0 mL) and water (3.0 mL). The layers were separated and the aqueous layer was extracted again with EtOAc for two more times (6.0 mL × 2). The combined organic layers were then concentrated in vacuo, and the residue was purified by silica gel chromatography eluting with petroleum ether/THF/EtOH to give **3** and **1**.

2.3.3 Synthetic applications

a) Scale-up kinetic resolution of *rac*-1a

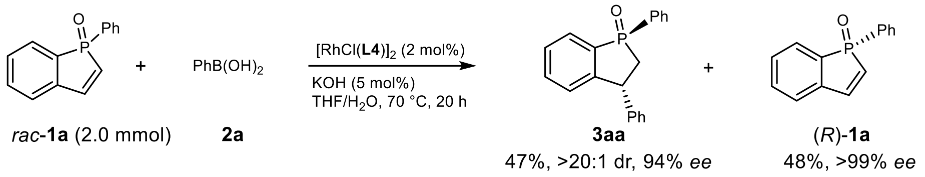

$[RhCl(\mathbf{L4})]_2$ (32.0 mg, 0.04 mmol, 2 mol%), *rac*-**1a** (452.0 mg, 2.0 mmol, 1.0 equiv) and **2a** (366.0 mg, 3.0 mmol, 1.5 equiv) were placed in an oven-dried Schlenk tube (50 mL) under nitrogen. THF (5.0 mL) and aqueous KOH (1.0 mL, 0.1 M, 5 mol%) were added and the resulting mixture was stirred at 70 °C for 20 h. Upon completion, the reaction mixture was diluted with EtOAc (20.0 mL) and water (10.0 mL). The layers were separated and the aqueous layer was extracted again with EtOAc for two more times (20.0 mL × 2). The combined organic layers were then concentrated in vacuo, and the residue was purified by silica gel chromatography eluting with petroleum ether/THF/EtOH (v/v/v = 80 : 20 : 1) to give **3aa** and (*R*)-**1a**.

b) Derivatization of the enantio-enriched products

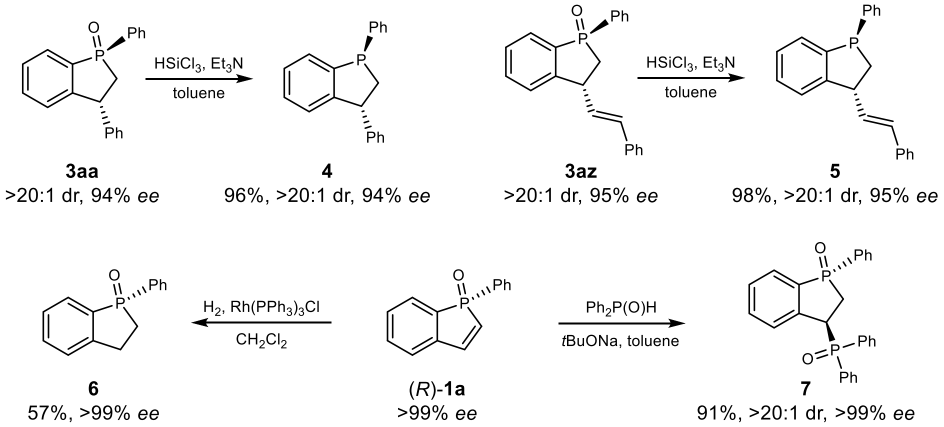

Procedure for synthesis of 4: A solution of **3aa** (30.4 mg, 0.10 mmol, 1.0 equiv) and triethylamine (42.0 μL, 0.30 mmol, 3.0 equiv) in anhydrous toluene (2.0 mL) was degassed by bubbling N_2 through the solution for 10 min. This solution was stirred and $HSiCl_3$ (31.0 μL, 0.30 mmol, 3.0 equiv) was added dropwise at room temperature. The mixture was stirred vigorously for 6 h at 80 °C. The yellow solution was allowed to

cool to room temperature and then was cooled in an ice bath. Water (5.0 mL) was added dropwise while the mixture was stirred. The aqueous layer was extracted with dichloromethane (5.0 mL × 3). The DCM fractions were combined and dried under vacuum. The residue was purified by silica gel chromatography eluting with petroleum ether/ethyl acetate (v/v = 40 : 1) to give **4** (96% yield, >20:1 dr, 94% *ee*).

Procedure for synthesis of 5: A solution of **3az** (33.0 mg, 0.10 mmol, 1.0 equiv) and triethylamine (42.0 μL, 0.30 mmol, 3.0 equiv) in anhydrous toluene (2.0 mL) was degassed by bubbling N_2 through the solution for 10 min. This solution was stirred and $HSiCl_3$ (31.0 μL, 0.30 mmol, 3.0 equiv) was added dropwise at room temperature. The mixture was stirred vigorously for 6 h at 80 ^{o}C. The yellow solution was allowed to cool to room temperature and then was cooled in an ice bath. Water (5.0 mL) was added dropwise while the mixture was stirred. The aqueous layer was extracted with dichloromethane (5.0 mL × 3). The DCM fractions were combined and dried under vacuum. The residue was purified by silica gel chromatography eluting with petroleum ether/ethyl acetate (v/v = 40 : 1) to give **5** (98% yield, >20:1 dr, 95% *ee*).

Procedure for synthesis of 6: A mixture of (*R*)-**1a** (45.2 mg, 0.20 mmol, 1.0 equiv) and $Rh(PPh_3)_3Cl$ (9.3 mg, 1.0 mmol, 5 mol %) in dichloromethane (10.0 mL) was stirred under 1 atm hydrogen at rt for 24 h. The reaction mixture was diluted with EtOAc (10.0 mL) and water (5.0 mL). The layers were separated and the aqueous layer was extracted again with EtOAc for two more times (5.0 mL × 2). The combined organic layers were then concentrated in vacuo, and the residue was purified by silica gel chromatography eluting with petroleum ether/ethyl acetate (v/v = 1 : 1) to give **6** (57% yield, >99% *ee*).

Procedure for synthesis of 7: To a solution of (*R*)-**1a** (22.6 mg, 0.10 mmol) in toluene (1.0 mL) was added a solution of NaO*t*Bu (2.0 M in THF, 10 μL, 0.020 mmol) at room temperature. The mixture was cooled to -20 °C and stirred for 5 min. Then, diphenylphosphine oxide (24.3 mg, 0.12 mmol) was added at -20 °C. After stirring for 5 min, the mixture was allowed to warm to room temperature for 6 h. The reaction was quenched with sat. aq. NH_4Cl, and the product was extracted with EtOAc. The

combined organic layer was dried over $MgSO_4$ and concentrated under reduced pressure. The residue was purified by silica gel column chromatography (CH_2Cl_2/MeOH = 50:1) to afford **7** (91% yield, >20:1 dr, >99% *ee*).

2.3.4 Synthesis of *ent*-3az

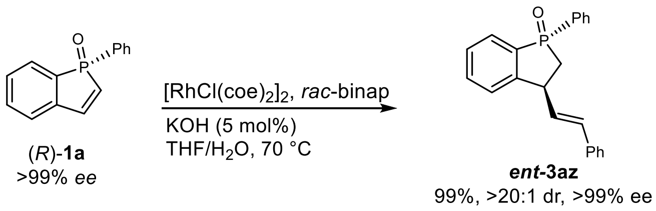

Supplementary Figure 5. Procedure for synthesis of *ent*-3az

$[RhCl(coe)_2]_2$ (1.4 mg, 2.0 μmol, 2.0 mol%) and *rac*-binap (3.2 mg, 0.50 mmol) were placed in a Schlenk tube (25 mL) under nitrogen. THF (0.4 mL) was added and the resulting solution was stirred at 25 °C for 5 min. Then, (*R*)-**1a** (22.6 mg, 0.10 mmol), (*E*)-phenylethenylboronic acid (22.2 mg, 0.15 mmol), aqueous KOH (0.1 mL, 0.1 M, 5 mol%) and THF (0.6 mL) were added. The mixture was stirred overnight at 80 °C. Upon completion, the reaction mixture was diluted with EtOAc (6.0 mL) and water (4.0 mL). The layers were separated and the aqueous layer was extracted again with EtOAc for two more times (6.0 mL × 2). The combined organic layers were then concentrated in vacuo, and the residue was purified by silica gel chromatography eluting with petroleum ether/EtOAc (v/v = 2 : 1) to give ***ent*-3az** (99% yield, >20:1 dr, >99% *ee*).

2.3.5 Mechanistic investigations

a) Discrimination of (*R*)-1a by chiral catalyst

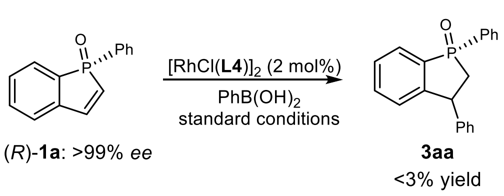

$[RhCl(\mathbf{L4})]_2$ (3.2 mg, 4.0 μmol, 2 mol%), (*R*)-**1a** (0.20 mmol, 1.0 equiv) and phenylboronic acid (0.30 mmol, 1.5 equiv) were placed in an oven-dried Schlenk tube

(25 mL) under nitrogen. THF (1.0 mL) and aqueous KOH (0.1 mL, 0.1 M, 5 mol%) were added and the resulting mixture was stirred at 70 °C for 20 h. The reaction mixture was diluted with EtOAc (6.0 mL) and water (3.0 mL). The layers were separated and the aqueous layer was extracted with EtOAc for two more times (6.0 mL × 2). The combined organic layers were removed on a rotary evaporator, and the crude product was analyzed by ^{1}H NMR with $CHCl_2CHCl_2$ (0.20 mmol, 21 μL) as an internal standard. The conversion of (*R*)-**1a** was calculated by comparing the integral of residual (*R*)-**1a** with the integral of $CHCl_2CHCl_2$ (s, 2H), and the NMR yield of **3aa** was calculated by comparing the integral of **3aa** with the integral of $CHCl_2CHCl_2$ (s, 2H).

b) Deuterium-labelling experiment of the model reaction

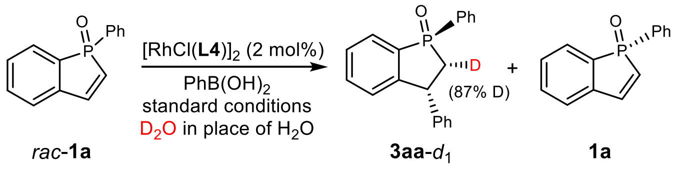

[RhCl(**L4**)]$_2$ (3.2 mg, 4.0 *μ*mol, 2 mol%), *rac*-**1a** (0.20 mmol, 1.0 equiv) and phenylboronic acid (0.30 mmol, 1.5 equiv) were placed in an oven-dried Schlenk tube (25 mL) under nitrogen. THF (1.0 mL) and aqueous solution of KOH (0.01 mmol in 0.1 mL D_2O) were added and the resulting mixture was stirred at 70 °C for 20 h. The reaction mixture was diluted with EtOAc (6.0 mL) and water (3.0 mL). The layers were separated and the aqueous layer was extracted with EtOAc for two more times (6.0 mL × 2). The combined organic layers were then concentrated in vacuo, and the residue was purified by silica gel chromatography eluting with petroleum ether/THF/EtOH to give **3aa-*d*$_1$** (39% yield) and **1a** (47% yield).

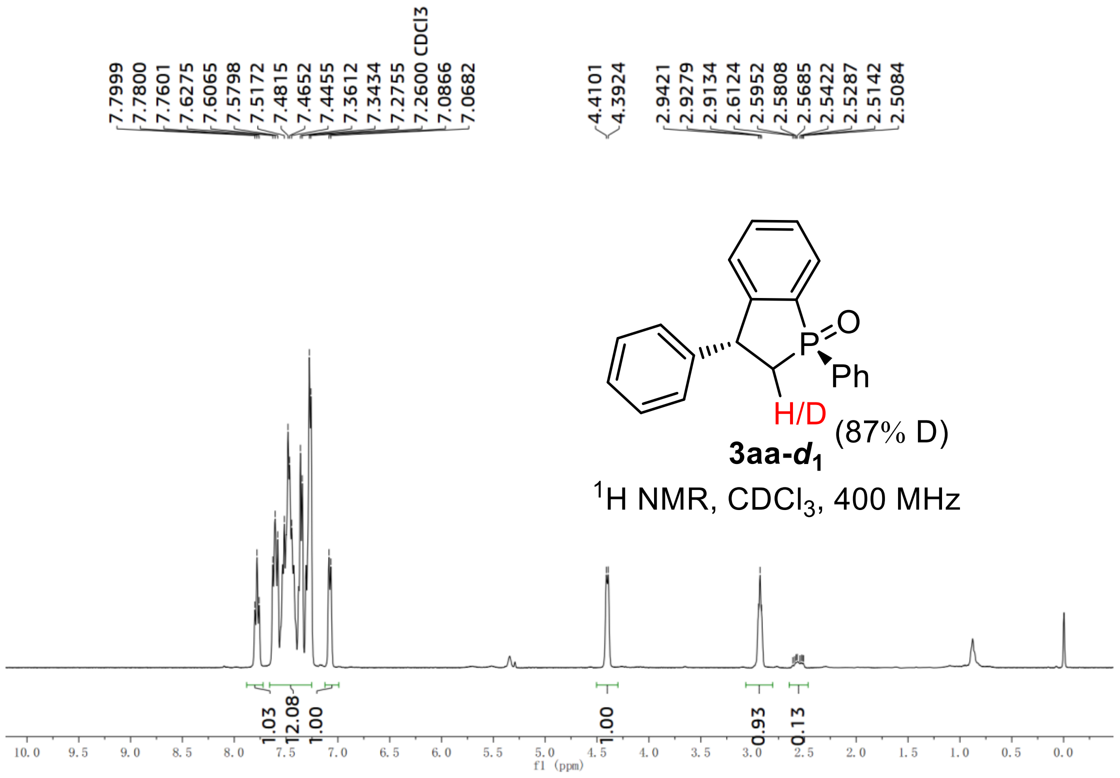

Supplementary Figure 6. ^{1}H NMR of the 3aa-d_1 (400 MHz, CDCl$_3$)

c) Control experiment

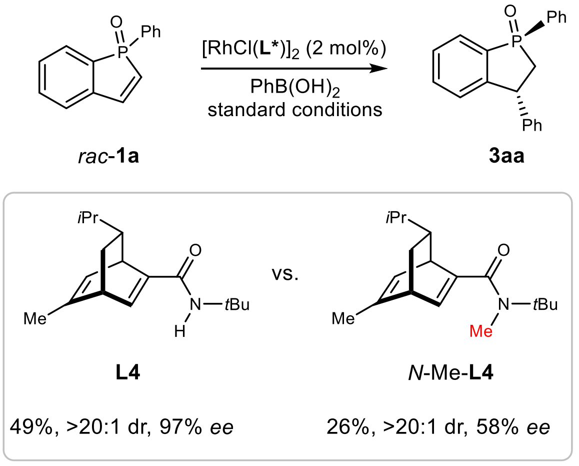

[RhCl(*N*-Me-**L4**)]$_2$ (3.3 mg, 4.0 μmol, 2 mol%), *rac*-**1a** (0.20 mmol, 1.0 equiv) and phenylboronic acid (0.30 mmol, 1.5 equiv) were placed in an oven-dried Schlenk tube (25 mL) under nitrogen. THF (1.0 mL) and aqueous solution of KOH (0.01 mmol in 0.1 mL H_2O) were added and the resulting mixture was stirred at 70 °C for 20 h. The reaction mixture was diluted with EtOAc (6.0 mL) and water (3.0 mL). The layers were separated and the aqueous layer was extracted again with EtOAc for two more times

(6.0 mL × 2). The combined organic layers were then concentrated in vacuo, and the residue was purified by silica gel chromatography eluting with petroleum ether/THF/EtOH to give **3aa** (26% yield, >20:1 dr, 58% *ee*).

2.4 Product characterization

(1*S*,3*R*)-1,3-diphenyl-2,3-dihydrophosphindole 1-oxide (3aa)

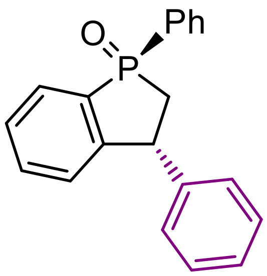

White solid, 29.8 mg at 0.20 mmol scale, 49% yield, >20:1 dr, 97% *ee*. **Optical rotation:** $[\alpha]^{25}_{D}$ -42 (c 0.4, $CHCl_3$). The enantiomeric excess was determined by chiral HPLC using CHIRALPAK IB N-5 column, *n*-Hexane/IPA = 88/12, flow rate 1.0 mL/min, uv-vis detection at λ = 210 nm, t_{major} = 19.06 min, t_{minor} = 11.70 min. **^{1}H NMR (400 MHz, $CDCl_3$)** δ 7.78 (t, J = 8.3 Hz, 1H), 7.64 – 7.57 (m, 2H), 7.56 – 7.40 (m, 5H), 7.38 – 7.34 (m, 2H), 7.31 – 7.27 (m, 3H), 7.10 – 7.05 (m, 1H), 4.41 (td, J = 8.0, 3.2 Hz, 1H), 3.01 – 2.87 (m, 1H), 2.56 (ddd, J = 19.0, 15.4, 8.2 Hz, 1H). **^{13}C NMR (101 MHz, $CDCl_3$)** δ 150.8 (d, J = 27.2 Hz), 143.8 (d, J = 8.8 Hz), 133.7 (d, J = 41.5 Hz), 133.2 (d, J = 1.5 Hz), 132.7 (d, J = 44.6 Hz), 132.1 (d, J = 2.2 Hz), 130.6 (d, J = 10.4 Hz), 129.2, 129.1, 128.9 (d, J = 11.9 Hz), 128.4, 128.3, 127.4, 127.1 (d, J = 12.2 Hz), 46.8 (d, J = 5.2 Hz), 39.0 (d, J = 68.0 Hz). **^{31}P NMR (162 MHz, $CDCl_3$)** δ 49.7. **HRMS-ESI (m/z):** calcd for $C_{20}H_{18}OP^+[M+H]^+$ 305.1090, found 305.1092.

(1*S*,3*R*)-1-phenyl-3-(*p*-tolyl)-2,3-dihydrophosphindole 1-oxide (3ab)

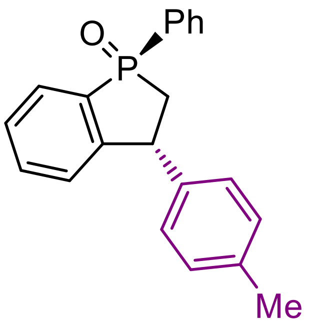

White solid, 30.0 mg at 0.20 mmol scale, 47% yield, >20:1 dr, 97% *ee*. **Optical rotation:** $[\alpha]^{25}_{D}$ -9.1 (c 0.04, $CHCl_3$). The enantiomeric excess was determined by chiral HPLC using CHIRALPAK IB N-5 column, *n*-Hexane/IPA = 88/12, flow rate 1.0 mL/min, uv-vis detection at λ = 210 nm, t_{major} = 17.75 min, t_{minor} = 10.64 min. **^{1}H NMR (400 MHz, $CDCl_3$)** δ 7.79 – 7.73 (m, 1H), 7.62-7.57 (m, 2H), 7.52 – 7.41 (m, 5H), 7.21 – 7.12 (m, 4H), 7.09 – 7.06 (m, 1H), 4.37 (td, J = 8.1, 3.1 Hz, 1H), 2.93 (ddd, J = 15.5, 7.8, 6.3 Hz, 1H), 2.54 (ddd, J = 19.1, 15.4, 8.3 Hz, 1H), 2.35 (s, 3H). **^{13}C NMR (101 MHz, $CDCl_3$)** δ 151.0 (d, J = 27.1 Hz), 140.9 (d, J = 9.1 Hz), 137.1, 133.1, 132.1, 130.6 (d, J = 10.4 Hz), 129.8, 129.0 (d, J = 8.5 Hz), 128.97, 128.85, 128.4, 128.29, 128.26, 127.1 (d, J = 12.2 Hz), 46.4 (d, J = 5.3 Hz), 39.1 (d, J = 68.3 Hz), 21.2. **^{31}P NMR (162**

MHz, $CDCl_3$) δ 49.6. **HRMS-ESI (m/z):** calcd for $C_{21}H_{20}OP^+[M+H]^+$ 319.1246, found 319.1244.

(1*S*,3*R*)-1-phenyl-3-(*m*-tolyl)-2,3-dihydrophosphindole 1-oxide (3ac)

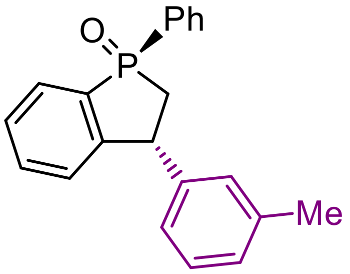

White solid, 31.2 mg at 0.20 mmol scale, 49% yield, >20:1 dr, 97% *ee*. **Optical rotation:** $[\alpha]^{25}_D$ -38.6 (c 0.11, $CHCl_3$). The enantiomeric excess was determined by chiral HPLC using CHIRALPAK IB N-5 column, *n*-Hexane/IPA = 88/12, flow rate 1.0 mL/min, uv-vis detection at λ = 210 nm, t_{major} = 16.04 min, t_{minor} = 10.82 min. **^{1}H NMR (400 MHz, $CDCl_3$)** δ 7.79 – 7.74 (m, 1H), 7.62 – 7.57 (m, 2H), 7.52 – 7.41 (m, 5H), 7.26 – 7.22 (m, 1H), 7.11 – 7.06 (m, 4H), 4.37 (td, *J* = 8.1, 3.1 Hz, 1H), 2.93 (ddd, *J* = 15.5, 7.9, 6.4 Hz, 1H), 2.55 (ddd, *J* = 19.1, 15.4, 8.2 Hz, 1H), 2.53 (s, 3H). **^{13}C NMR (101 MHz, $CDCl_3$)** δ 150.8 (d, *J* = 27.3 Hz), 143.8 (d, *J* = 9.1 Hz), 138.9, 133.7 (d, *J* = 50.8 Hz), 133.1 (d, *J* = 1.5 Hz), 132.7 (d, *J* = 54.7 Hz), 132.1 (d, *J* = 2.2 Hz), 130.6 (d, *J* = 10.5 Hz), 129.1, 129.0, 128.9, 128.8, 128.3 (d, *J* = 10.6 Hz), 128.2, 127.1 (d, *J* = 12.3 Hz), 125.5, 46.7 (d, *J* = 5.3 Hz), 39.0 (d, *J* = 68.3 Hz), 21.5. **^{31}P NMR (162 MHz, $CDCl_3$)** δ 49.7. **HRMS-ESI (m/z):** calcd for $C_{21}H_{20}OP^+[M+H]^+$ 319.1246, found 319.1246.

(1*S*,3*R*)-1-phenyl-3-(4-vinylphenyl)-2,3-dihydrophosphindole 1-oxide (3ad)

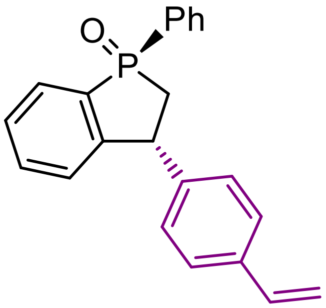

White solid, 26.4 mg at 0.20 mmol scale, 40% yield, >20:1 dr, 90% *ee*. **Optical rotation:** $[\alpha]^{25}_D$ -96.6 (c 0.06, $CHCl_3$). The enantiomeric excess was determined by chiral HPLC using CHIRALPAK IB N-5 column, *n*-Hexane/IPA = 88/12, flow rate 1.0 mL/min, uv-vis detection at λ = 254 nm, t_{major} = 20.91 min, t_{minor} = 11.79 min. **^{1}H NMR (400 MHz, $CDCl_3$)** δ 7.78 (t, *J* = 8.3 Hz, 1H), 7.62 – 7.39 (m, 9H), 7.22 (d, *J* = 8.1 Hz, 2H), 7.08 (d, *J* = 8.7 Hz, 1H), 6.71 (dd, *J* = 17.6, 10.9 Hz, 1H), 5.75 (d, *J* = 17.6 Hz, 1H), 5.25 (d, *J* = 10.9 Hz, 1H), 4.40 (td, *J* = 7.9, 3.0 Hz, 1H), 2.97 – 2.89 (m, 1H), 2.54 (ddd, *J* = 19.0, 15.4, 8.2 Hz, 1H). **^{13}C NMR (101 MHz, $CDCl_3$)** δ 150.6

(d, J = 27.1 Hz), 143.3 (d, J = 9.1 Hz), 136.8, 136.4, 133.3 (d, J = 4.6 Hz), 133.2 (d, J = 98.8 Hz), 132.3, 132.2, 130.6 (d, J = 10.5 Hz), 128.99, 128.97 (d, J = 20.5 Hz), 128.6, 128.4 (d, J = 10.6 Hz), 127.1 (d, J = 12.5 Hz), 127.0, 114.2, 46.4 (d, J = 5.3 Hz), 38.8 (d, J = 68.0 Hz). **^{31}P NMR (162 MHz, CDCl$_3$)** δ 49.2. **HRMS-ESI (m/z):** calcd for $C_{22}H_{20}OP^+[M+H]^+$ 331.1246, found 331.1244.

<u>(1*S*,3*R*)-1-phenyl-3-(3-vinylphenyl)-2,3-dihydrophosphindole 1-oxide (3ae)</u>

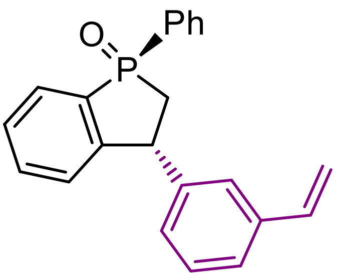

White solid, 31.8 mg at 0.20 mmol scale, 48% yield, >20:1 dr, 95% *ee*. **Optical rotation:** $[\alpha]^{25}_{D}$ -78.8 (c 0.12, CHCl$_3$). The enantiomeric excess was determined by chiral HPLC using CHIRALPAK IB N-5 column, *n*-Hexane/IPA = 95/5, flow rate 1.0 mL/min, uv-vis detection at λ = 254 nm, t_{major} = 42.65 min, t_{minor} = 30.01 min. **^{1}H NMR (400 MHz, CDCl$_3$)** δ 7.83 – 7.74 (m, 1H), 7.64 – 7.42 (m, 7H), 7.32 (dd, J = 10.1, 2.5 Hz, 3H), 7.15 (d, J = 7.0 Hz, 1H), 7.12 – 7.05 (m, 1H), 6.69 (dd, J = 17.6, 10.9 Hz, 1H), 5.74 (d, J = 17.6 Hz, 1H), 5.25 (d, J = 10.9 Hz, 1H), 4.40 (td, J = 7.9, 2.9 Hz, 1H), 3.00 – 2.88 (m, 1H), 2.57 (ddd, J = 19.2, 15.4, 8.3 Hz, 1H). **^{13}C NMR (101 MHz, CDCl$_3$)** δ 150.6 (d, J = 27.4 Hz), 144.0 (d, J = 8.8 Hz), 138.4, 136.6, 133.3, 133.1 (d, J = 98.5 Hz), 132.2, 130.6 (d, J = 10.5 Hz), 129.4, 129.1, 128.9 (d, J = 11.8 Hz), 128.5 (d, J = 10.5 Hz), 127.8, 127.1 (d, J = 12.3 Hz), 126.3, 125.3, 114.6, 46.7 (d, J = 5.2 Hz), 38.9 (d, J = 67.9 Hz). **^{31}P NMR (162 MHz, CDCl$_3$)** δ 49.2. **HRMS-ESI (m/z):** calcd for $C_{22}H_{20}OP^+[M+H]^+$ 331.1246, found 331.1247.

<u>(1*S*,3*R*)-3-(4-fluorophenyl)-1-phenyl-2,3-dihydrophosphindole 1-oxide (3af)</u>

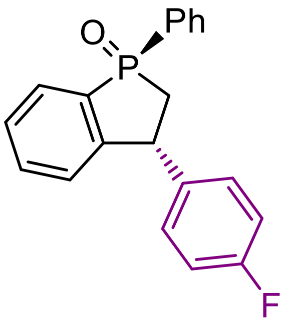

White solid, 31.6 mg at 0.20 mmol scale, 49% yield, >20:1 dr, 97% *ee*. **Optical rotation:** $[\alpha]^{25}_{D}$ -48.8 (c 0.24, CHCl$_3$). The enantiomeric excess was determined by chiral HPLC using CHIRALPAK IB N-5 column, *n*-Hexane/IPA = 95/5, flow rate 1.0 mL/min, uv-vis detection at λ = 210 nm, t_{major} = 55.86 min, t_{minor} = 27.52 min. **^{1}H NMR (400 MHz, CDCl$_3$)** δ 7.77 (t, J = 8.2 Hz, 1H), 7.61 – 7.42 (m, 7H), 7.26 – 7.20 (m, 2H), 7.07 – 7.01 (m,

3H), 4.42 (td, J = 7.7, 3.4 Hz, 1H), 2.93 (ddd, J = 14.7, 7.9, 6.2 Hz, 1H), 2.49 (ddd, J = 18.7, 15.4, 7.8 Hz, 1H). **^{13}C NMR (101 MHz, CDCl$_3$)** δ 162.1 (d, J = 246.0 Hz), 150.6 (d, J = 26.9 Hz), 139.7 (d, J = 10.9 Hz), 133.5 (d, J = 23.1 Hz) d, 133.3, 132.5 (d, J = 29.8 Hz), 132.2, 130.6 (d, J = 10.2 Hz), 129.9 (d, J = 8.0 Hz), 129.1, 128.9 (d, J = 12.1 Hz), 128.5 (d, J = 10.1 Hz), 127.0 (d, J = 11.9 Hz), 116.0 (d, J = 21.4 Hz), 46.1 (d, J = 4.4 Hz), 39.9 (d, J = 67.8 Hz). **^{31}P NMR (162 MHz, CDCl$_3$)** δ 49.6. **HRMS-ESI (m/z):** calcd for $C_{20}H_{17}FOP^+[M+H]^+$ 323.0996, found 323.0982.

<u>(1*S*,3*R*)-3-(4-chlorophenyl)-1-phenyl-2,3-dihydrophosphindole 1-oxide (3ag)</u>

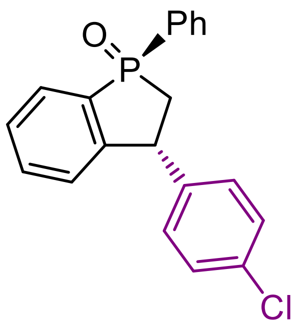

White solid, 30.4 mg at 0.20 mmol scale, 45% yield, >20:1 dr, 97% *ee*. **Optical rotation:** $[\alpha]^{25}_D$ -88.4 (c 0.09, CHCl$_3$). The enantiomeric excess was determined by chiral HPLC using CHIRALPAK IB N-5 column, *n*-Hexane/IPA = 88/12, flow rate 1.0 mL/min, uv-vis detection at λ = 210 nm, t_{major} = 26.90 min, t_{minor} = 12.32 min. **^{1}H NMR (400 MHz, CDCl$_3$)** δ 7.77 (t, J = 8.4 Hz, 1H), 7.59 – 7.56 (m, 2H), 7.53 – 7.51 (m, 2H), 7.48 – 7.44 (m, 3H), 7.36 – 7.31 (m, 2H), 7.22 – 7.20 (m, 2H), 7.07 (d, J = 7.8 Hz, 1H), 4.41 (q, J = 6.9, 6.2 Hz, 1H), 2.98 – 2.87 (m, 1H), 2.54 – 2.43 (m, 1H). **^{13}C NMR (101 MHz, CDCl$_3$)** δ 150.2 (d, J = 27.0 Hz), 142.4 (d, J = 8.8 Hz), 133.6 (d, J = 10.4 Hz), 133.3, 132.6 (d, J = 14.0 Hz), 132.2 (d, J = 1.8 Hz), 130. 6 (d, J = 10.5 Hz), 129.7, 129.3, 129.2, 129.1, 129.0 (d, J = 12.1 Hz), 128.6 (d, J = 10.5 Hz), 127.0 (d, J = 12.3 Hz), 46.2 (d, J = 5.2 Hz), 38.9 (d, J = 68.4 Hz). **^{31}P NMR (162 MHz, CDCl$_3$)** δ 49.6. **HRMS-ESI (m/z):** calcd for $C_{20}H_{17}ClOP^+[M+H]^+$ 339.0700, found 339.0697.

<u>(1*S*,3*R*)-3-(4-bromophenyl)-1-phenyl-2,3-dihydrophosphindole 1-oxide (3ah)</u>

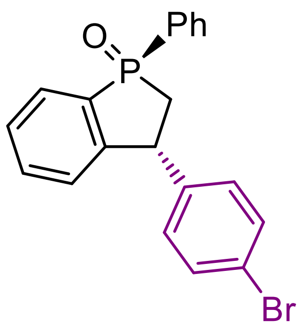

White solid, 35.2 mg at 0.20 mmol scale, 46% yield, >20:1 dr, 98% *ee*. **Optical rotation:** $[\alpha]^{25}_D$ -95.0 (c 0.12, CHCl$_3$). The enantiomeric excess was determined by chiral HPLC using CHIRALPAK IB N-5 column, *n*-Hexane/IPA = 88/12, flow rate 1.0 mL/min, uv-vis detection at λ = 210 nm, t_{major} = 28.87 min, t_{minor} = 12.96 min. **^{1}H NMR (400 MHz, CDCl$_3$)**

δ 7.76 (t, J = 9.0 Hz, 1H), 7.61 – 7.56 (m, 2H), 7.53 – 7.51 (m, 1H), 7.49 – 7.43 (m, 6H), 7.17 – 7.13 (m, 2H), 7.08 – 7.05 (m, 1H), 4.38 (td, J = 7.9, 4.2 Hz, 1H), 2.91 (ddd, J = 15.0, 8.0, 6.5 Hz, 1H), 2.47 (ddd, J = 18.8, 15.4, 7.8 Hz, 1H). **^{13}C NMR (101 MHz, $CDCl_3$)** δ 150.1 (d, J = 27.2 Hz), 143.0 (d, J = 8.6 Hz), 133.6 (d, J = 8.7 Hz), 133.3 (d, J = 1.7 Hz), 132.6 (d, J = 11.5 Hz), 132.3, 132.2 (d, J = 2.4 Hz), 130.6 (d, J = 10.6 Hz), 130.1, 129.1 (d, J = 8.7 Hz), 128.9 (d, J = 12.2 Hz), 128.6 (d, J = 10.5 Hz), 127.0 (d, J = 12.2 Hz), 121.4, 46.3, (d, J = 5.4 Hz), 38.9 (d, J = 68.4 Hz). **^{31}P NMR (162 MHz, $CDCl_3$)** δ 49.6. **HRMS-ESI (m/z):** calcd for $C_{20}H_{17}{}^{79}BrOP^+[M+H]^+$ 383.0195, found 383.0188; calcd for $C_{20}H_{17}{}^{81}BrOP^+[M+H]^+$ 385.0174, found 385.0168.

<u>(1*S*,3*R*)-3-(3-chlorophenyl)-1-phenyl-2,3-dihydrophosphindole 1-oxide (3ai)</u>

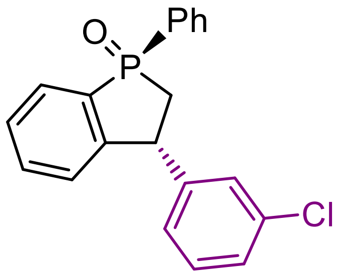

White solid, 33.8 mg at 0.20 mmol scale, 50% yield, >20:1 dr, 98% *ee*. **Optical rotation:** $[\alpha]^{25}_{D}$ -105.2 (c 0.33, $CHCl_3$). The enantiomeric excess was determined by chiral HPLC using CHIRALPAK IB N-5 column, *n*-Hexane/IPA = 88/12, flow rate 1.0 mL/min, uv-vis detection at λ = 210 nm, t_{major} = 20.88 min, t_{minor} = 12.37 min. **^{1}H NMR (400 MHz, $CDCl_3$)** δ 7.76 (t, J = 8.0 Hz, 1H), 7.60 – 7.55 (m, 2H), 7.53 – 7.48 (m, 2H), 7.46 – 7.40 (m, 3H), 7.29 – 7.23 (m, 3H), 7.14 – 7.06 (m, 2H), 4.40 – 4.36 (m, 1H), 2.92 (dt, J = 14.3, 6.6 Hz, 1H), 2.57 – 2.42 (m, 1H). **^{13}C NMR (101 MHz, $CDCl_3$)** δ 149.9 (d, J = 27.0 Hz), 145.8 (d, J = 8.8 Hz), 134.9, 133.5 (d, J = 6.6 Hz), 133.3 (d, J = 2.0 Hz), 132.5 (d, J = 9.5 Hz), 132.2 (d, J = 2.5 Hz), 130.6, 130.5, 129.1 (d, J = 8.7 Hz), 128.9 (d, J = 12.1 Hz), 128.7, 128.6, 127.6, 127.0 (d, J = 12.1 Hz), 126.5, 46.4 (d, J = 5.5 Hz), 38.7 (d, J = 68.0 Hz). **^{31}P NMR (162 MHz, $CDCl_3$)** δ 49.7. **HRMS-ESI (m/z):** calcd for $C_{20}H_{17}ClOP^+[M+H]^+$ 339.0700, found 339.0697.

(1*S*,3*R*)-1-phenyl-3-(4-(trifluoromethyl)phenyl)-2,3-dihydrophosphindole 1-oxide (3aj)

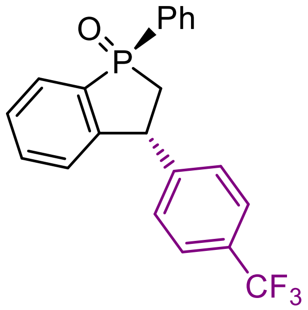

White solid, 35.8 mg at 0.20 mmol scale, 48% yield, >20:1 dr, 97% *ee*. **Optical rotation:** $[\alpha]^{25}_{D}$ -89.7 (c 0.12, $CHCl_3$). The enantiomeric excess was determined by chiral HPLC using CHIRALPAK IB N-5 column, *n*-Hexane/IPA = 88/12, flow rate 1.0 mL/min, uv-vis detection at λ = 210 nm, t_{major} = 24.74 min, t_{minor} = 10.29 min. **^{1}H NMR (400 MHz, $CDCl_3$)** δ 7.78 (t, *J* = 8.2 Hz, 1H), 7.62 – 7.57 (m, 4H), 7.54 – 7.44 (m, 5H), 7.40 (d, *J* = 8.0 Hz, 2H), 7.06 (d, *J* = 8.0 Hz, 1H), 4.51 (td, *J* = 7.7, 3.9 Hz, 1H), 3.00 – 2.88 (m, 1H), 2.51 (ddd, *J* = 18.4, 15.4, 7.6 Hz, 1H). **^{13}C NMR (101 MHz, $CDCl_3$)** δ 149.8 (d, *J* = 27.1 Hz), 147.9 (d, *J* = 7.8 Hz), 133.7, 133.4, 132.6, 132.3, 130.7 (d, *J* = 10.2 Hz), 129.8 (d, *J* = 32.7 Hz), 129.2 (d, *J* = 8.6 Hz), 129.0 (d, *J* = 3.9 Hz), 128.8, 127.0 (d, *J* = 11.8 Hz), 126.1 (q, *J* = 3.6 Hz), 124.2 (q, *J* = 272.6 Hz), 46.5 (d, *J* = 4.2 Hz), 38.7 (d, *J* = 68.3 Hz). **^{31}P NMR (162 MHz, $CDCl_3$)** δ 49.8. **HRMS-ESI (m/z):** calcd for $C_{21}H_{17}F_3OP^+[M+H]^+$ 373.0964, found 373.0962.

1-(4-((1*S*,3*R*)-1-oxido-1-phenyl-2,3-dihydrophosphindol-3-yl)phenyl)ethan-1-one (3ak)

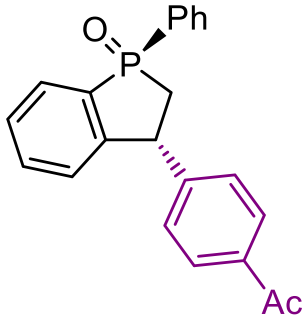

White solid, 32.6 mg at 0.20 mmol scale, 47% yield, >20:1 dr, 98% *ee*. **Optical rotation:** $[\alpha]^{25}_{D}$ +54.5 (c 0.02, $CHCl_3$). The enantiomeric excess was determined by chiral HPLC using CHIRALPAK IB N-5 column, *n*-Hexane/IPA = 80/20, flow rate 1.0 mL/min, uv-vis detection at λ = 210 nm, t_{major} = 30.61 min, t_{minor} = 17.25 min. **^{1}H NMR (400 MHz, $CDCl_3$)** δ 7.95 (d, *J* = 8.4 Hz, 2H), 7.78 (t, *J* = 8.2 Hz, 1H), 7.62 – 7.57 (m, 2H), 7.53 – 7.44 (m, 5H), 7.38 (d, *J* = 8.4 Hz, 2H), 7.06 – 7.04 (m, 1H), 4.50 (td, *J* = 7.8, 4.3 Hz, 1H), 2.95 (ddd, *J* = 15.0, 8.1, 6.5 Hz, 1H), 2.59 (m, 4H). **^{13}C NMR (101 MHz, $CDCl_3$)** δ 197.7, 149.8 (d, *J* = 27.3 Hz), 149.2 (d, *J* = 8.4 Hz), 136.4, 133.5 (d, *J* = 3.8 Hz), 133.4 (d, *J* = 1.6 Hz), 132.5, 132.3 (d, *J* = 1.9 Hz), 130.7 (d, *J* = 10.5 Hz), 129.3, 129.2, 129.0 (d, *J* = 3.8 Hz), 128.8, 128.7, 127.0 (d, *J* = 12.1 Hz), 46.7 (d, *J* = 5.2 Hz), 38.5 (d, *J* = 68.4 Hz), 26.8. **^{31}P NMR**

(162 MHz, CDCl3) δ 50.1. **HRMS-ESI (m/z):** calcd for $C_{22}H_{20}O_2P^+[M+H]^+$ 347.1195, found 347.1197.

1-(3-((1*S*,3*R*)-1-oxido-1-phenyl-2,3-dihydrophosphindol-3-yl)phenyl)ethan-1-one (3al)

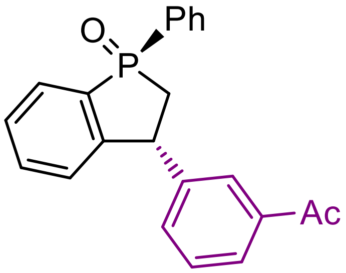

White solid, 32.1 mg at 0.20 mmol scale, 46% yield, >20:1 dr, 99% *ee*. **Optical rotation:** $[\alpha]^{25}_{D}$ -96.0 (c 0.43, CHCl3). The enantiomeric excess was determined by chiral HPLC using CHIRALPAK IB N-5 column, *n*-Hexane/IPA = 80/20, flow rate 1.0 mL/min, uv-vis detection at λ = 210 nm, t_{major} = 17.63 min, t_{minor} = 12.27 min. **^{1}H NMR (400 MHz, CDCl3)** δ 7.91 – 7.85 (m, 2H), 7.78 (t, *J* = 7.7 Hz, 1H), 7.62 – 7.58 (m, 2H), 7.53 – 7.43 (m, 7H), 7.06 (d, *J* = 7.7 Hz, 1H), 4.51 (t, *J* = 7.6 Hz, 1H), 2.97 – 2.92 (m, 1H), 2.61 – 2.48 (m, 4H). **^{13}C NMR (101 MHz, CDCl3)** δ 198.1, 150.1 (d, *J* = 25.0 Hz), 144.4 (d, *J* = 7.0 Hz), 137.8, 133.7, 133.4, 132.9, 132.7, 132.2, 130.7 (d, *J* = 9.0 Hz), 129.6, 129.2, (d, *J* = 7.3 Hz), 129.0 (d, *J* = 11.1 Hz), 128.7 (d, *J* = 9.1 Hz), 128.2, 127.6, 127.0 (d, *J* = 10.9 Hz), 46.7, 39.0 (d, *J* = 68.4 Hz), 26.9. **^{31}P NMR (162 MHz, CDCl3)** δ 49.7. **HRMS-ESI (m/z):** calcd for $C_{22}H_{20}O_2P^+[M+H]^+$ 347.1195, found 347.1203.

(1*S*,3*R*)-1-phenyl-3-(*o*-tolyl)-2,3-dihydrophosphindole 1-oxide (3am)

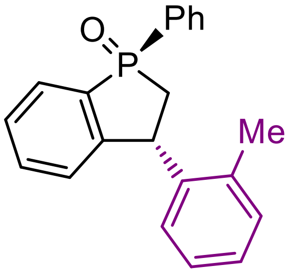

White solid, 30.6 mg at 0.20 mmol scale, 48% yield, >20:1 dr, 90% *ee*. **Optical rotation:** $[\alpha]^{25}_{D}$ -35.3 (c 0.33, CHCl3). The enantiomeric excess was determined by chiral HPLC using CHIRALPAK IB N-5 column, *n*-Hexane/IPA = 88/12, flow rate 1.0 mL/min, uv-vis detection at λ = 210 nm, t_{major} = 16.50 min, t_{minor} = 11.46 min. **^{1}H NMR (400 MHz, DMSO-d_6)** δ 7.68 – 7.61 (m, 3H), 7.56 (dd, *J* = 14.3, 7.3 Hz, 4H), 7.44 (td, *J* = 7.2, 2.9 Hz, 1H), 7.25 – 7.22 (m, 1H), 7.19 – 7.13 (m, 2H), 7.05 (d, *J* = 7.6 Hz, 1H), 6.99 (s, 1H), 4.88 (q, *J* = 6.9 Hz, 1H), 3.14 (ddd, *J* = 15.3, 8.4, 6.8 Hz, 1H), 2.37 (s, 3H), 2.17 (td, *J* = 17.4, 6.7 Hz, 1H). **^{13}C NMR (101 MHz, CDCl3)** δ 150.7 (d, *J* = 27.4 Hz), 142.2, 135.7, 134.0, 133.5, 133.2 (d, *J* = 1.7 Hz), 133.0, 132.6, 132.1 (d, *J* = 2.4 Hz), 130.5 (d, *J* = 10.5 Hz), 129.1, 129.0 (d, *J*

= 12.2 Hz), 128.3 (d, J = 10.5 Hz), 127.2, 127.1, 126.9, 42.0, 37.6 (d, J = 61.2 Hz), 19.9. **^{31}P NMR (162 MHz, CDCl$_3$)** δ 49.9. **HRMS-ESI (m/z):** calcd for $C_{21}H_{20}OP^+[M+H]^+$ 319.1246, found 319.1248.

(1*S*,3*R*)-3-([1,1'-biphenyl]-2-yl)-1-phenyl-2,3-dihdrophosphindole 1-oxide (3an)

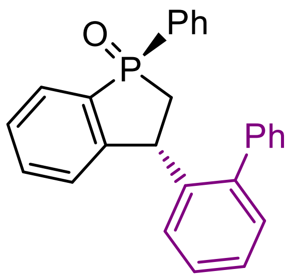

White solid, 38.0 mg at 0.20 mmol scale, 50% yield, >20:1 dr, 96% *ee*. **Optical rotation:** $[\alpha]^{25}_{D}$ -120.0 (c 0.44, CHCl$_3$). The enantiomeric excess was determined by chiral HPLC using CHIRALPAK IB N-5 column, *n*-Hexane/IPA = 88/12, flow rate 1.0 mL/min, uv-vis detection at λ = 210 nm, t_{major} = 13.39 min, t_{minor} = 10.98 min. **^{1}H NMR (400 MHz, CDCl$_3$)** δ 7.72 (t, J = 8.1 Hz, 1H), 7.43 – 7.33 (m, 4H), 7.43 – 7.33 (m, 9H), 7.31 – 7.30 (m, 2H), 7.22 (d, J = 7.0 Hz, 1H), 7.03 (d, J = 7.8 Hz, 1H), 4.66 (dt, J = 8.0, 4.7 Hz, 1H), 2.83 (dt, J = 14.5, 7.3 Hz, 1H), 2.55 (ddd, J = 18.8, 15.4, 8.0 Hz, 1H). **^{13}C NMR (101 MHz, CDCl$_3$)** δ 151.2 (d, J = 27.2 Hz), 142.3, 141.8 (d, J = 8.2 Hz), 141.2, 134.0, 133.1, 133.0, 132.0, 130.3 (d, J = 9.9 Hz), 129.8, 129.3, 128.8, 128.71, 128.65, 128.5, 128.1 (d, J = 9.9 Hz), 127.4, 127.1, 127.0, 126.9, 42.5 (d, J = 2.7 Hz), 39.4 (d, J = 67.9 Hz). **^{31}P NMR (162 MHz, CDCl$_3$)** δ 49.5. **HRMS-ESI (m/z):** calcd for $C_{26}H_{22}OP^+[M+H]^+$ 381.1403, found 381.1403.

(1*S*,3*R*)-3-(benzo[d][1,3]dioxol-5-yl)-1-phenyl-2,3-dihydrophosphindole 1-oxide (3ao)

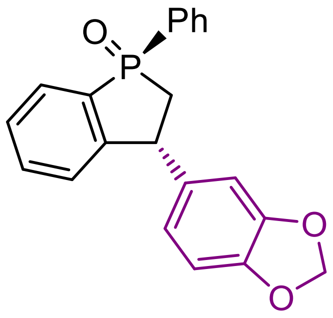

White solid, 30.0 mg at 0.20 mmol scale, 43% yield, >20:1 dr, 95% *ee*. **Optical rotation:** $[\alpha]^{25}_{D}$ -101.1 (c 0.09, CHCl$_3$). The enantiomeric excess was determined by chiral HPLC using CHIRALPAK IB N-5 column, *n*-Hexane/IPA = 88/12, flow rate 1.0 mL/min, uv-vis detection at λ = 210 nm, t_{major} = 32.07 min, t_{minor} = 19.00 min. **^{1}H NMR (400 MHz, CDCl$_3$)** δ 7.76 (t, J = 8.2 Hz, 1H), 7.60 – 7.55 (m, 2H), 7.52 – 7.49 (m, 2H), 7.46 – 7.40 (m, 3H), 7.12 (d, J = 10.1 Hz, 1H), 6.79 – 6.72 (m, 2H), 6.70 (d, J = 1.7 Hz, 1H), 5.95 (s, 2H), 4.33 (td, J = 7.7, 2.8 Hz, 1H), 2.91 (ddd, J = 15.4,

7.9, 6.3 Hz, 1H), 2.49 (ddd, *J* = 19.1, 15.4, 8.2 Hz, 1H). **^{13}C NMR (101 MHz, $CDCl_3$)** *δ* 150.7 (d, *J* = 26.8 Hz), 148.4, 146.9, 137.7 (d, *J* = 9.1 Hz), 133.4, 133.2, 132.8, 132.3, 132.1, 130.6 (d, *J* = 10.4 Hz), 129.0 (d, *J* = 8.2 Hz), 128.9 (d, *J* = 12.0 Hz), 128.4 (d, *J* = 10.4 Hz), 127.1 (d, *J* = 12.2 Hz), 121.7, 108.5 (d, *J* = 5.3 Hz), 101.3, 46.5 (d, *J* = 5.3 Hz), 39.1 (d, *J* = 67.5 Hz). **^{31}P NMR (162 MHz, $CDCl_3$)** *δ* 49.4. **HRMS-ESI (m/z):** calcd for $C_{21}H_{18}O_3P^+$[M+H]$^+$ 349.0988, found 349.0971.

(1*S*,3*R*)-3-(3,5-dichlorophenyl)-1-phenyl-2,3-dihydrophosphindole 1-oxide (3ap)

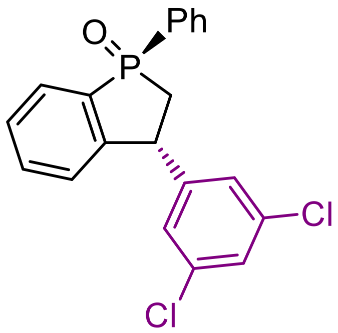

White solid, 35.8 mg at 0.20 mmol scale, 48% yield, >20:1 dr, 97% *ee*. **Optical rotation:** $[\alpha]^{25}_D$ -131.3 (c 0.31, $CHCl_3$). The enantiomeric excess was determined by chiral HPLC using CHIRALPAK IB N-5 column, *n*-Hexane/IPA = 88/12, flow rate 1.0 mL/min, uv-vis detection at λ = 210 nm, t_{major} = 18.00 min, t_{minor} = 10.39 min. **^{1}H NMR (400 MHz, $CDCl_3$)** *δ* 7.76 (t, *J* = 8.2 Hz, 1H), 7.57 – 7.49 (m, 4H), 7.46 – 7.42 (m, 3H), 7.27 (t, *J* = 1.8 Hz, 1H), 7.15 (d, *J* = 1.9 Hz, 2H), 7.08 (d, *J* = 7.7 Hz, 1H), 4.37 (td, *J* = 7.8, 3.6 Hz, 1H), 2.96 – 2.84 (m, 1H), 2.45 (ddd, *J* = 18.6, 15.5, 7.7 Hz, 1H). **^{13}C NMR (101 MHz, $CDCl_3$)** *δ* 135.7, 133.5, 132.7, 132.3, 130.60 (d, *J* = 10.3 Hz), 130.58, 129.3 (d, *J* = 8.1 Hz), 129.0, 128.96, 128.92, 128.87, 127.8, 127.0, 126.94, 126.89, 46.2 (d, *J* = 4.1 Hz), 38.6 (d, *J* = 67.3 Hz). **^{31}P NMR (162 MHz, $CDCl_3$)** *δ* 49.7. **HRMS-ESI (m/z):** calcd for $C_{20}H_{16}Cl_2OP^+$[M+H]$^+$ 373.0310, found 373.0311.

(1*S*,3*R*)-3-([1,1'-biphenyl]-4-yl)-1-phenyl-2,3-dihydrophosphindole 1-oxide (3aq)

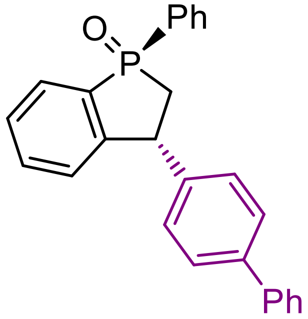

White solid, 37.2 mg at 0.20 mmol scale, 49% yield, >20:1 dr, 95% *ee*. **Optical rotation:** $[\alpha]^{25}_D$ -113.6 (c 0.27, $CHCl_3$). The enantiomeric excess was determined by chiral HPLC using CHIRALPAK IB N-5 column, *n*-Hexane/IPA = 88/12, flow rate 1.0 mL/min, uv-vis detection at λ = 210 nm, t_{major} = 43.09 min, t_{minor} = 18.95 min. **^{1}H NMR (400 MHz, $CDCl_3$)** *δ* 7.78 (t, *J* = 8.2 Hz, 1H), 7.65 – 7.58 (m, 6H), 7.54 – 7.42 (m, 7H), 7.36 – 7.32

(m, 3H), 7.14 (d, *J* = 7.7 Hz, 1H), 4.46 (td, *J* = 8.0, 3.1 Hz, 1H), 2.96 (ddd, *J* = 14.6, 7.8, 6.2 Hz, 1H), 2.58 (ddd, *J* = 18.9, 15.4, 8.1 Hz, 1H). **^{13}C NMR (101 MHz, CDCl$_3$)** *δ* 150.7 (d, *J* = 27.2 Hz), 142.9 (d, *J* = 8.7 Hz), 140.5 (d, *J* = 32.8 Hz), 133.7 (d, *J* = 35.3 Hz), 133.2, 132.7 (d, *J* = 40.3 Hz), 132.1, 130.6 (d, *J* = 10.3 Hz), 129.1, 128.98, 128.92, 128.86, 128.81, 128.4 (d, *J* = 10.2 Hz), 127.8, 127.5, 127.2, 127.1, 46.4 (d, *J* = 4.5 Hz), 39.0 (d, *J* = 67.7 Hz). **^{31}P NMR (162 MHz, CDCl$_3$)** *δ* 49.7. **HRMS-ESI (m/z):** calcd for $C_{26}H_{22}OP^+$[M+H]$^+$ 381.1403, found 381.1405.

(1*S*,3*R*)-3-(naphthalen-2-yl)-1-phenyl-2,3-dihydrophosphindole 1-oxide (3ar)

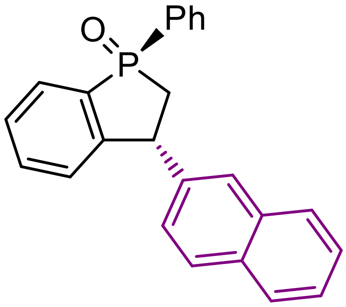

White solid, 34.0 mg at 0.20 mmol scale, 48% yield, >20:1 dr, 97% *ee*. **Optical rotation:** $[\alpha]^{25}_D$ -129.2 (c 0.24, CHCl$_3$). The enantiomeric excess was determined by chiral HPLC using CHIRALPAK IB N-5 column, *n*-Hexane/IPA = 88/12, flow rate 1.0 mL/min, uv-vis detection at λ = 210 nm, t_{major} = 29.81 min, t_{minor} = 21.28 min. **^{1}H NMR (400 MHz, CDCl$_3$)** *δ* 7.84 – 7.76 (m, 5H), 7.63 – 7.60 (d, *J* = 12.2 Hz, 2H), 7.55 – 7.40 (m, 7H), 7.33 (dd, *J* = 8.5, 1.8 Hz, 1H), 7.08 (d, *J* = 6.8 Hz, 1H), 4.58 (td, *J* = 8.1, 2.9 Hz, 1H), 3.00 (ddd, *J* = 15.5, 7.9, 6.3 Hz, 1H), 2.65 (ddd, *J* = 19.1, 15.4, 8.2 Hz, 1H). **^{13}C NMR (101 MHz, CDCl$_3$)** *δ* 150.6 (d, *J* = 27.4 Hz), 141.0 (d, *J* = 8.9 Hz), 133.7 (d, *J* = 35.2 Hz), 133.6, 133.2 (d, *J* = 1.1 Hz), 132.78 (d, *J* = 38.2 Hz), 132.76, 132.1 (d, *J* = 1.9 Hz), 130.6 (d, *J* = 10.5 Hz), 129.3, 129.1, 129.0, 128.9, 128.5 (d, *J* = 10.5 Hz), 127.9 (d, *J* = 2.8 Hz), 127.4, 127.3, 127.2, 126.6, 126.1 (d, *J* = 20.0 Hz), 47.0 (d, *J* = 5.2 Hz), 38.9 (d, *J* = 68.2 Hz). **^{31}P NMR (162 MHz, CDCl$_3$)** *δ* 49.8. **HRMS-ESI (m/z):** calcd for $C_{24}H_{20}OP^+$[M+H]$^+$ 355.1246, found 355.1245.

(1*S*,3*R*)-3-(1-methyl-1*H*-indol-5-yl)-1-phenyl-2,3-dihydrophosphindole 1-oxide (3as)

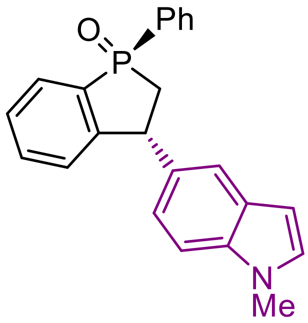

White solid, 34.4 mg at 0.20 mmol scale, 48% yield, >20:1 dr, 92% *ee*. **Optical rotation:** $[\alpha]^{25}_{D}$ -100.4 (c 0.25, $CHCl_3$). The enantiomeric excess was determined by chiral HPLC using CHIRALPAK IB N-5 column, *n*-Hexane/IPA = 88/12, flow rate 1.0 mL/min, uv-vis detection at λ = 210 nm, t_{major} = 39.82 min, t_{minor} = 32.41 min. **^{1}H NMR (400 MHz, $CDCl_3$)** δ 7.81 – 7.69 (m, 2H), 7.65 – 7.60 (m, 2H), 7.46 – 7.30 (m, 7H), 7.10 – 7.06 (m, 2H), 6.45 (d, *J* = 3.1 Hz, 1H), 4.50 (t, *J* = 7.3 Hz, 1H), 3.79 (s, 3H), 3.05 – 2.89 (m, 1H), 2.64 (ddd, *J* = 19.2, 15.4, 8.4 Hz, 1H). **^{13}C NMR (101 MHz, $CDCl_3$)** δ 151.4 (d, *J* = 27.1 Hz), 145.0 (d, *J* = 13.1 Hz), 141.7 (d, *J* = 31.3 Hz), 135.6, 134.3 (d, *J* = 9.4 Hz), 132.6 (d, *J* = 11.5 Hz), 131.8 (d, *J* = 42.9 Hz), 130.5 (d, *J* = 10.9 Hz), 130.1 (d, *J* = 10.4 Hz), 129.2, 128.4 (d, *J* = 11.9 Hz), 127.6 (d, *J* = 10.4 Hz), 126.8 (d, *J* = 12.3 Hz), 124.5 (d, *J* = 10.1 Hz), 121.4, 120.3, 109.6, 100.5, 46.5 (d, *J* = 5.3 Hz), 39.1 (d, *J* = 67.4 Hz), 32.6. **^{31}P NMR (162 MHz, $CDCl_3$)** δ 50.2. **HRMS-ESI (m/z):** calcd for $C_{23}H_{21}NOP^+$$[M+H]^+$ 358.1355, found 358.1355.

(1*S*,3*S*)-3-(1-methyl-1*H*-indol-2-yl)-1-phenyl-2,3-dihydrophosphindole 1-oxide (3at)

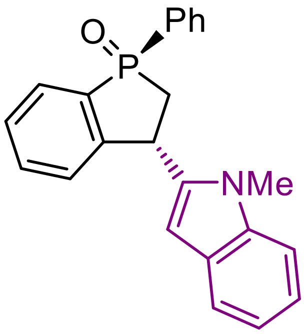

White solid, 26.4 mg at 0.20 mmol scale, 37% yield, >20:1 dr, 97% *ee*. **Optical rotation:** $[\alpha]^{25}_{D}$ -0.59 (c 0.34, $CHCl_3$). The enantiomeric excess was determined by chiral HPLC using CHIRALPAK IB N-5 column, *n*-Hexane/IPA = 80/20, flow rate 1.0 mL/min, uv-vis detection at λ = 210 nm, t_{major} = 22.42 min, t_{minor} = 20.17 min. **^{1}H NMR (400 MHz, $CDCl_3$)** δ 7.83 (t, *J* = 8.3 Hz, 1H), 7.65 – 7.45 (m, 8H), 7.36 – 7.19 (m, 3H), 7.12 (t, *J* = 7.0 Hz, 1H), 6.36 (s, 1H), 4.83 – 4.72 (m, 1H), 3.59 (s, 3H), 2.99 (ddd, *J* = 15.1, 8.2, 6.7 Hz, 1H), 2.67 (ddd, *J* = 19.0, 15.5, 8.3 Hz, 1H). **^{13}C NMR (101 MHz, $CDCl_3$)** δ 147.4 (d, *J* = 26.2 Hz), 139.6, 137.1, 132.7, 131.8 (d, *J* = 118.2 Hz), 129.5 (d, *J* = 10.4 Hz), 128.4

(d, J = 8.3 Hz), 128.0 (d, J = 12.2 Hz), 128.0, 126.5, 125.7 (d, J = 12.1 Hz), 120.8, 119.6, 118.9, 108.2, 100.7, 38.2 (d, J = 7.7 Hz), 35.6 (d, J = 67.6 Hz), 29.7. **^{31}P NMR** (162 MHz, $CDCl_3$) δ 48.0. **HRMS-ESI (m/z):** calcd for $C_{23}H_{21}NOP^+[M+H]^+$ 358.1355, found 358.1356.

(1*S*,3*R*)-1-phenyl-3-(quinolin-7-yl)-2,3-dihydrophosphindole 1-oxide (3au)

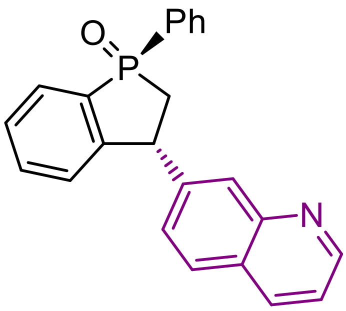

White solid, 29.2 mg at 0.20 mmol scale, 41% yield, >20:1 dr, 98% *ee*. **Optical rotation:** $[\alpha]^{25}_D$ -58.3 (c 0.02, $CHCl_3$). The enantiomeric excess was determined by chiral HPLC using CHIRALPAK IB N-5 column, *n*-Hexane/IPA = 80/20, flow rate 1.0 mL/min, uv-vis detection at λ = 210 nm, t_{major} = 35.23 min, t_{minor} = 17.93 min. **^{1}H NMR (400 MHz, $CDCl_3$)** δ 8.92 (d, J = 2.6 Hz, 1H), 8.15 (d, J = 8.2 Hz, 1H), 8.06 (s, 1H), 7.82 – 7.77 (m, 2H), 7.65 – 7.57 (m, 2H), 7.56 – 7.35 (m, 7H), 7.09 – 7.07 (m, 1H), 4.68 (td, J = 7.9, 3.9 Hz, 1H), 3.02 (ddd, J = 15.0, 8.1, 6.4 Hz, 1H), 2.63 (ddd, J = 18.9, 15.5, 7.8 Hz, 1H). **^{13}C NMR (101 MHz, $CDCl_3$)** δ 150.9, 150.0 (d, J = 27.4 Hz), 148.2, 145.1 (d, J = 8.6 Hz), 136.2, 133.6, 133.4 (d, J = 2.0 Hz), 132.5 (d, J = 5.4 Hz), 132.2, 130.6 (d, J = 10.6 Hz), 129.3, 129.1 (d, J = 8.8 Hz), 129.0 (d, J = 12.2 Hz), 128.7 (d, J = 6.1 Hz), 128.6, 127.5, 127.2 (d, J = 12.1 Hz), 126.5, 121.4, 46.9 (d, J = 5.3 Hz), 38.4 (d, J = 68.1 Hz). **^{31}P NMR (162 MHz, $CDCl_3$)** δ 49.9. **HRMS-ESI (m/z):** calcd for $C_{23}H_{19}NOP^+[M+H]^+$ 356.1199, found 356.1200.

(1*S*,3*R*)-3-(benzofuran-5-yl)-1-phenyl-2,3-dihydrophosphindole 1-oxide (3av)

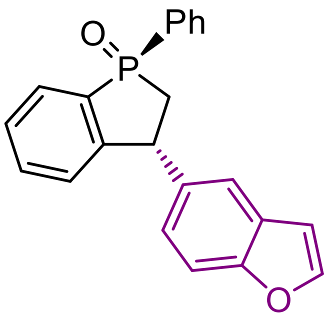

White solid, 30.4 mg at 0.20 mmol scale, 44% yield, >20:1 dr, 94% *ee*. **Optical rotation:** $[\alpha]^{25}_D$ -133.3 (c 0.09, $CHCl_3$). The enantiomeric excess was determined by chiral HPLC using CHIRALPAK IB N-5 column, *n*-Hexane/IPA = 88/12, flow rate 1.0 mL/min, uv-vis detection at λ = 210 nm, t_{major} = 26.44 min, t_{minor} = 16.78 min. **^{1}H NMR (400 MHz, $CDCl_3$)** δ 7.79 (t, J = 7.7 Hz, 1H), 7.66 – 7.59 (m, 3H), 7.55 – 7.40 (m, 7H), 7.21 – 7.05 (m, 2H), 6.75 – 6.71 (m, 1H), 4.52 (td, J = 7.5, 3.1 Hz,

1H), 2.99 (dp, J = 13.9, 6.8 Hz, 1H), 2.61 (ddd, J = 26.8, 14.9, 7.4 Hz, 1H). **^{13}C NMR (101 MHz, CDCl$_3$)** δ 154.3, 151.2 (d, J = 27.3 Hz), 145.8, 138.6 (d, J = 8.8 Hz), 133.6 (d, J = 47.5 Hz), 133.2, 132.6 (d, J = 50.9 Hz), 132.1, 130.6 (d, J = 10.2 Hz), 129.0 (d, J = 12.4 Hz), 128.9, 128.4 (d, J = 10.0 Hz), 128.1, 127.2 (d, J = 11.7 Hz), 124.7, 120.9, 112.0, 106.7, 46.8 (d, J = 2.0 Hz), 39.4 (d, J = 67.8 Hz). **^{31}P NMR (162 MHz, CDCl$_3$)** δ 49.6. **HRMS-ESI (m/z):** calcd for $C_{22}H_{18}O_2P^+$[M+H]$^+$ 345.1039, found 345.1031.

(1*S*,3*R*)-3-(dibenzo[b,d]furan-2-yl)-1-phenyl-2,3-dihydrophosphindole 1-oxide (3aw)

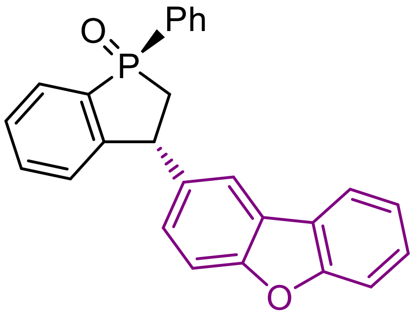

White solid, 33.2 mg at 0.20 mmol scale, 42% yield, >20:1 dr, 95% *ee*. **Optical rotation:** $[\alpha]^{25}_{D}$ –344.2 (c 0.05, CHCl$_3$). The enantiomeric excess was determined by chiral HPLC using CHIRALPAK IB N-5 column, *n*-Hexane/IPA = 88/12, flow rate 1.0 mL/min, uv-vis detection at λ = 210 nm, t_{major} = 29.71 min, t_{minor} = 19.65 min. **^{1}H NMR (400 MHz, CDCl$_3$)** δ 7.91 – 7.79 (m, 3H), 7.66 – 7.61 (m, 2H), 7.57 – 7.43 (m, 8H), 7.36 – 7.30 (m, 2H), 7.10 (d, J = 7.9 Hz, 1H), 4.59 (t, J = 8.1 Hz, 1H), 3.08 – 3.0 (dt, J = 16.4, 8.5 Hz, 1H), 2.66 (td, J = 17.3, 16.2, 9.4 Hz, 1H). **^{13}C NMR (101 MHz, CDCl$_3$)** δ 156.7, 155.5, 151.1 (d, J = 27.0 Hz), 138.6 (d, J = 9.1 Hz), 133.7, 133.3, 132.7, 132.3, 132.2 (d, J = 2.2 Hz), 130.6 (d, J = 10.5 Hz), 129.1, 129.0 (d, J = 12.0 Hz), 128.5 (d, J = 10.5 Hz), 127.5 (d, J = 2.9 Hz), 127.2 (d, J = 12.3 Hz), 125.0, 124.0, 123.0, 120.9, 120.4, 112.0 (d, J = 30.5 Hz), 108.0, 46.8 (d, J = 5.4 Hz), 39.5 (d, J = 67.7 Hz). **^{31}P NMR (162 MHz, CDCl$_3$)** δ 49.6. **HRMS-ESI (m/z):** calcd for $C_{26}H_{20}O_2P^+$[M+H]$^+$ 395.1195, found 395.1188.

(1*S*,3*S*)-3-(benzo[b]thiophen-3-yl)-1-phenyl-2,3-dihydrophosphindole 1-oxide (3ax)

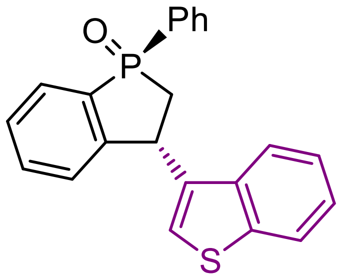

White solid, 31.0 mg at 0.20 mmol scale, 43% yield, >20:1 dr, >99% *ee*. **Optical rotation:** $[\alpha]^{25}_{D}$ -54.0 (c 0.25, $CHCl_3$). The enantiomeric excess was determined by chiral HPLC using CHIRALPAK IB N-5 column, *n*-Hexane/IPA = 88/12, flow rate 1.0 mL/min, uv-vis detection at λ = 210 nm, t_{major} = 26.58 min, t_{minor} = 20.68 min. **^{1}H NMR (400 MHz, $CDCl_3$)** δ 7.89 – 7.81 (m, 2H), 7.65 – 7.60 (m, 2H), 7.57 – 7.45 (m, 6H), 7.38 – 7.27 (m, 3H), 7.23 – 7.18 (m, 1H), 4.90 (td, *J* = 8.0, 3.9 Hz, 1H), 2.97 (ddd, *J* = 15.0, 8.1, 6.6 Hz, 1H), 2.76 (ddd, *J* = 18.7, 15.5, 7.9 Hz, 1H). **^{13}C NMR (101 MHz, $CDCl_3$)** δ 149.3 (d, *J* = 26.9 Hz), 141.2, 137.5 (d, *J* = 8.5 Hz), 137.2, 133.4 (d, *J* = 49.9 Hz), 133.4 (d, *J* = 2.0 Hz), 132.4 (d, *J* = 52.9 Hz), 132.2 (d, *J* = 2.6 Hz), 130.6 (d, *J* = 10.5 Hz), 129.4 (d, *J* = 8.7 Hz), 129.0 (d, *J* = 12.1 Hz), 128.7 (d, *J* = 10.5 Hz), 126.8, (d, *J* = 12.0 Hz), 124.7, 124.4, 124.4, 123.4, 122.2, 40. (d, *J* = 5.3 Hz), 36.6 (d, *J* = 68.0 Hz). **^{31}P NMR (162 MHz, $CDCl_3$)** δ 49.2. **HRMS-ESI (m/z):** calcd for $C_{22}H_{18}OPS^+[M+H]^+$ 361.0810, found 361.0812.

(1*S*,3*R*)-3-(dibenzo[b,d]thiophen-2-yl)-1-phenyl-2,3-dihydrophosphindole 1-oxide (3ay)

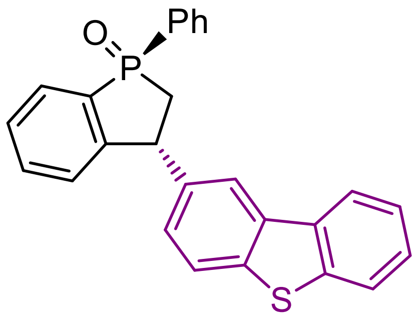

White solid, 28.8 mg at 0.20 mmol scale, 35% yield, >20:1 dr, 97% *ee*. **Optical rotation:** $[\alpha]^{25}_{D}$ -129.6 (c 0.15, $CHCl_3$). The enantiomeric excess was determined by chiral HPLC using CHIRALPAK IB N-5 column, *n*-Hexane/IPA = 88/12, flow rate 1.0 mL/min, uv-vis detection at λ = 210 nm, t_{major} = 35.16 min, t_{minor} = 27.36 min. **^{1}H NMR (400 MHz, $CDCl_3$)** δ 8.11 – 8.08 (m, 2H), 7.84 – 7.82 (m, 3H), 7.67 – 7.62 (m, 2H), 7.49 – 7.44 (m, 7H), 7.36 (d, *J* = 8.3 Hz, 1H), 7.12 – 6.97 (m, 1H), 4.60 (t, *J* = 8.5 Hz, 1H), 3.05 (dt, *J* = 16.0, 7.8 Hz, 1H), 2.67 (td, *J* = 18.5, 17.9, 7.8 Hz, 1H). **^{13}C NMR (101 MHz, $CDCl_3$)** δ 150.8 (d, *J* = 27.0 Hz), 140.3 (d, *J* = 9.1 Hz), 140.0, 138.5, 136.3, 135.2, 133.5 (d, *J* = 28.8 Hz), 133.3 (d, *J* = 1.9 Hz), 132.5 (d, *J* =

32.4 Hz), 132.2 (d, J = 2.4 Hz), 130.6 (d, J = 10.5 Hz), 129.1, 129.0 (d, J = 12.0 Hz), 128.5 (d, J = 10.5 Hz), 127.2, 127.1 (d, J = 2.3 Hz), 125.6, 124.6, 123.5, 123.0, 121.8, 121.4, 46.8 (d, J = 5.4 Hz), 39.4 (d, J = 67.8 Hz). **^{31}P NMR (162 MHz, CDCl$_3$)** δ 49.9. **HRMS-ESI (m/z):** calcd for $C_{26}H_{20}OPS^+[M+H]^+$ 411.0967, found 411.0961.

(1*S*,3*R*)-1-phenyl-3-((*E*)-styryl)-2,3-dihydrophosphindole 1-oxide (3az)

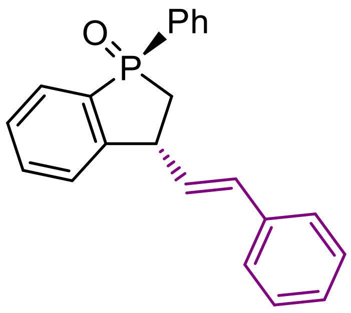

White solid, 27.8 mg at 0.20 mmol scale, 42% yield, >20:1 dr, 95% *ee*. **Optical rotation:** $[\alpha]^{25}_{D}$ -26.0 (c 0.10, CHCl$_3$). The enantiomeric excess was determined by chiral HPLC using CHIRALPAK IB N-5 column, *n*-Hexane/IPA = 88/12, flow rate 1.0 mL/min, uv-vis detection at λ = 210 nm, t_{major} = 29.35 min, t_{minor} = 15.75 min. **^{1}H NMR (400 MHz, CDCl$_3$)** δ 8.03 (t, J = 8.7 Hz, 1H), 7.88 – 7.82 (m, 3H), 7.80 – 7.78 (m, 1H), 7.75 – 7.68 (m, 5H), 7.67 – 7.66 (m, 1H), 7.63 – 7.59 (m, 2H), 7.56 – 7.52 (m, 1H), 6.92 (d, J = 15.6 Hz, 1H), 6.54 (dd, J = 15.6, 9.1 Hz, 1H), 4.32 (qd, J = 8.1, 3.3 Hz, 1H), 3.06 (ddd, J = 15.3, 7.7, 6.1 Hz, 1H), 2.68 (ddd, J = 19.1, 15.3, 7.9 Hz, 1H). **^{13}C NMR (101 MHz, CDCl$_3$)** δ 149.3 (d, J = 27.6 Hz), 136.5, 135.9, 133.3 (d, J = 98.4 Hz), 133.1 (d, J = 1.6 Hz), 132.4, 132.1 (d, J = 1.7 Hz), 131.2 (d, J = 9.5 Hz), 130.5 (d, J = 10.5 Hz), 129.3 (d, J = 8.7 Hz), 128.9, 128.8, 128.6 (d, J = 10.5 Hz), 128.0, 126.5, 126.1 (d, J = 101.7 Hz), 44.5 (d, J = 5.4 Hz), 36.3 (d, J = 68.3 Hz). **^{31}P NMR (162 MHz, CDCl$_3$)** δ 49.9. **HRMS-ESI (m/z):** calcd for $C_{22}H_{20}OP^+[M+H]^+$ 331.1246, found 331.1240.

(1*S*,3*R*)-3-((*E*)-4-methoxystyryl)-1-phenyl-2,3-dihydrophosphindole 1-oxide (3az-2)

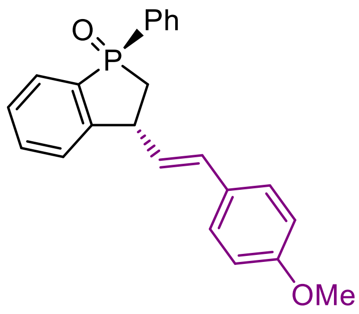

White solid, 28.2 mg at 0.20 mmol scale, 39% yield, >20:1 dr, 97% *ee*. **Optical rotation:** $[\alpha]^{25}_{D}$ -91.1 (c 0.11, CHCl$_3$). The enantiomeric excess was determined by chiral HPLC using CHIRALPAK IB N-5 column, n-Hexane/IPA = 85/15, flow rate 1.0 mL/min, uv-vis detection at λ = 210 nm, t_{major} = 27.69 min, t_{minor}

= 21.12 min. **^{1}H NMR (400 MHz, CDCl$_3$)** δ 7.74 (t, J = 8.3 Hz, 1H), 7.60 – 7.55 (m, 3H), 7.54 – 7.42 (m, 5H), 7.35 (t, J = 7.3 Hz, 2H), 6.68 (d, J = 15.6 Hz, 2H), 6.31 (dd, J = 15.6, 9.1 Hz, 1H), 4.07 (td, J = 8.1, 3.3 Hz, 1H), 3.80 (s, 3H), 2.87 – 2.72 (m, 1H), 2.43 (ddd, J = 19.0, 15.4, 7.8 Hz, 1H). **^{13}C NMR (101 MHz, CDCl$_3$)** δ 159.5, 149.60 (d, J = 27.5 Hz), 133.1 (d, J = 1.8 Hz), 132.09, 132.07, 131.9, 130.5 (d, J = 10.5 Hz), 129.3, 129.1 (d, J = 9.8 Hz), 128.99, 128.95, 128.8, 128.5 (d, J = 10.5 Hz), 127.7, 126.6 (d, J = 12.1 Hz), 114.2, 55.5, 44.6 (d, J = 5.5 Hz), 36.4 (d, J = 68.4 Hz). **^{31}P NMR (162 MHz, CDCl$_3$)** δ 49.3. **HRMS-ESI (m/z):** calcd for $C_{23}H_{22}O_2P^+[M+H]^+$ 361.1352, found 361.1346.

(1*S*,3*R*)-3-((*E*)-3-fluorostyryl)-1-phenyl-2,3-dihydrophosphindole 1-oxide (3az-3)

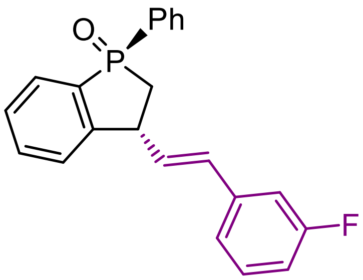

White solid, 32.0 mg at 0.20 mmol scale, 46% yield, >20:1 dr, 92% *ee*. **Optical rotation:** $[\alpha]^{25}_D$ -40.5 (c 0.07, CHCl$_3$). The enantiomeric excess was determined by chiral HPLC using CHIRALPAK IB N-5 column, n-Hexane/IPA = 85/15, flow rate 1.0 mL/min, uv-vis detection at λ = 210 nm, t_{major} = 23.45 min, t_{minor} = 12.57 min. **^{1}H NMR (400 MHz, CDCl$_3$)** δ 7.75 (t, J = 8.3 Hz, 1H), 7.60 – 7.52 (m, 4H), 7.47 – 7.43 (m, 4H), 7.31 – 7.26 (m, 1H), 7.17 (d, J = 7.8 Hz, 1H), 7.10 (d, J = 10.0 Hz, 1H), 6.95 (td, J = 8.4, 2.5 Hz, 1H), 6.60 (d, J = 15.6 Hz, 1H), 6.27 (dd, J = 15.6, 9.0 Hz, 1H), 4.06 – 4.03 (m, 1H), 2.79 – 2.73 (m, 1H), 2.40 (ddd, J = 18.9, 15.4, 7.7 Hz, 1H). **^{13}C NMR (101 MHz, CDCl$_3$)** δ 163.2 (d, J = 245.6 Hz), 149.0 (d, J = 27.5 Hz), 138.8 (d, J = 7.7 Hz), 133.6, 133.2 (d, J = 1.9 Hz), 133.1, 132.6 (d, J = 9.0 Hz), 132.2 (d, J = 2.5 Hz), 131.3, 130.6 (d, J = 10.5 Hz), 130.3 (d, J = 8.4 Hz), 129.4 (d, J = 8.7 Hz), 128.9 (d, J = 12.1 Hz), 128.7 (d, J = 10.5 Hz), 126.5 (d, J = 12.0 Hz), 122.3 (d, J = 2.5 Hz), 114.8 (d, J = 21.3 Hz), 113.1 (d, J = 21.8 Hz), 44.4 (d, J = 5.6 Hz), 36.1 (d, J = 68.6 Hz). **^{31}P NMR (162 MHz, CDCl$_3$)** δ 49.1. **HRMS-ESI (m/z):** calcd for $C_{22}H_{19}FOP^+[M+H]^+$ 349.1152, found 349.1149.

(1*S*,3*R*)-1-phenyl-3-((*E*)-4-(trifluoromethyl)styryl)-2,3-dihydrophosphindole 1-oxide (3az-4)

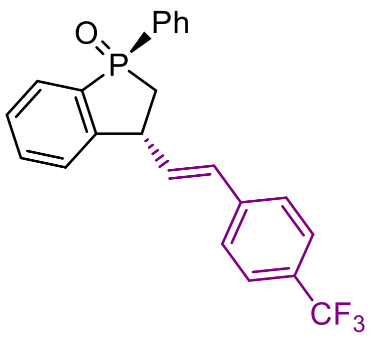

White solid, 33.4 mg at 0.20 mmol scale, 42% yield, >20:1 dr, 91% *ee.* **Optical rotation:** $[\alpha]^{25}_{D}$ -90.8 (c 0.19, $CHCl_3$). The enantiomeric excess was determined by chiral HPLC using CHIRALPAK IB N-5 column, *n*-Hexane/IPA = 88/12, flow rate 1.0 mL/min, uv-vis detection at λ = 210 nm, t_{major} = 24.14 min, t_{minor} = 14.50 min. **^{1}H NMR (400 MHz, $CDCl_3$)** δ 7.75 (t, J = 8.6 Hz, 1H), 7.60 – 7.56 (m, 5H), 7.53 – 7.41 (m, 7H), 6.67 (d, J = 15.7 Hz, 1H), 6.38 (dd, J = 15.8, 9.0 Hz, 1H), 4.10 – 4.06 (m, 1H), 2.77 (dt, J = 13.9, 6.8 Hz, 1H), 2.49 – 2.34 (m, 1H). **^{13}C NMR (101 MHz, $CDCl_3$)** δ 148.8 (d, J = 27.6 Hz), 140.0, 133.9 (d, J = 8.8 Hz), 133.6, 133.3 (d, J =1.8 Hz), 132.6, 132.2 (d, J = 2.0 Hz), 131.0, 130.6 (d, J = 10.5 Hz), 129.7 (d, J =32.5 Hz), 129.4 (d, J = 8.9 Hz), 128.9 (d, J = 12.2 Hz), 128.7, 127.0 (q, J = 273.2 Hz), 126.8, 126.5 (d, J = 12.1 Hz), 125.8 (q, J = 3.8 Hz), 44.5 (d, J = 5.3 Hz), 36.0 (d, J = 68.7 Hz). **^{31}P NMR (162 MHz, $CDCl_3$)** δ 50.0. **HRMS-ESI (m/z):** calcd for $C_{23}H_{19}F_3OP^+$[M+H]$^+$ 399.1120, found 399.1113.

(1*S*,3*R*)-3-((*E*)-2-([1,1'-biphenyl]-4-yl)vinyl)-1-phenyl-2,3-dihydrophosphindole 1-oxide (3az-5)

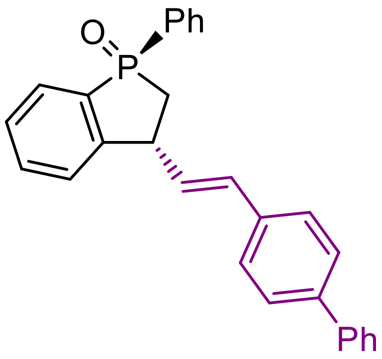

White solid, 32.4 mg at 0.20 mmol scale, 40% yield, >20:1 dr, 96% *ee.* **Optical rotation:** $[\alpha]^{25}_{D}$ -158.5 (c 0.08, $CHCl_3$). The enantiomeric excess was determined by chiral HPLC using CHIRALPAK IB N-5 column, n-Hexane/IPA = 88/12, flow rate 1.0 mL/min, uv-vis detection at λ = 210 nm, t_{major} = 19.52 min, t_{minor} = 12.88 min. **^{1}H NMR (400 MHz, $CDCl_3$)** δ 7.77 (t, J = 8.3 Hz, 1H), 7.63 – 7.56 (m, 7H), 7.54 – 7.40 (m, 9H), 7.35 (t, J = 7.3 Hz, 1H), 6.68 (d, J = 15.6 Hz, 1H), 6.31 (dd, J = 15.6, 9.1 Hz, 1H), 4.07 (td, J = 8.1, 3.3 Hz, 1H), 2.85 – 2.72 (m, 1H), 2.43 (ddd, J = 19.0, 15.4, 7.8 Hz, 1H). **^{13}C NMR (101 MHz, $CDCl_3$)** δ 149.3 (d, J = 27.6 Hz), 140.7 (d, J = 8.6 Hz), 135.5, 133.7, 133.18, 133.16,

133.1, 132.8, 132.13, 132.10, 132.0, 131.3 (d, *J* = 9.4 Hz), 130.6 (d, *J* = 10.5 Hz), 129.3 (d, *J* = 8.7 Hz), 128.9, 128.8, 128.6 (d, *J* = 10.4 Hz), 127.51, 127.47, 127.0 (d, *J* = 6.5 Hz), 126.6 (d, *J* = 12.1 Hz), 44.6 (d, *J* = 5.6 Hz), 36.2 (d, *J* = 68.4 Hz). **^{31}P NMR (162 MHz, CDCl$_3$)** δ 49.2. **HRMS-ESI (m/z):** calcd for $C_{28}H_{24}OP^+[M+H]^+$ 407.1559, found 407.1555.

(1*S*,3*R*)-3-(cyclopent-1-en-1-yl)-1-phenyl-2,3-dihydrophosphindole 1-oxide (3az-6)

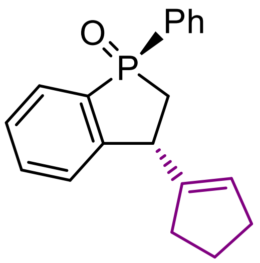

White solid, 24.8 mg at 0.20 mmol scale, 42% yield, >20:1 dr, 92% *ee*. **Optical rotation:** $[\alpha]^{25}_D$ -34.1 (c 0.18, CHCl$_3$). The enantiomeric excess was determined by chiral HPLC using CHIRALPAK IB N-5 column, n-Hexane/IPA = 88/12, flow rate 1.0 mL/min, uv-vis detection at λ = 210 nm, t_{major} = 12.41 min, t_{minor} = 8.55 min. **^{1}H NMR (400 MHz, acetone-*d*$_6$)** δ 7.63 – 7.37 (m, 9H), 5.74 (s, 1H), 4.37 (q, *J* = 7.1 Hz, 1H), 2.72 – 2.65 (m, 1H), 2.40 – 2.33 (m, 3H), 2.29 – 2.08 (m, 2H), 1.99 – 1.84 (m, 2H). **^{13}C NMR (101 MHz, acetone-*d*$_6$)** δ 149.9 (d, *J* = 28.1 Hz), 145.8 (d, *J* = 7.5 Hz), 135.7 (d, *J* = 51.0 Hz), 134.7 (d, *J* = 56.1 Hz), 133.4, 132.4, 131.2 (d, *J* = 10.2 Hz), 129.4 (d, *J* = 11.8 Hz), 129.3 (d, *J* = 9.1 Hz), 128.8 (d, *J* = 10.3 Hz), 128.3, 127.1 (d, *J* = 11.9 Hz), 43.2 (d, *J* = 4.5 Hz), 34.2 (d, *J* = 69.0 Hz), 32.8, 31.4, 24.1. **^{31}P NMR (162 MHz, acetone-*d*$_6$)** δ 47.0. **HRMS-ESI (m/z):** calcd for $C_{19}H_{20}OP^+[M+H]^+$ 295.1246, found 295.1250.

(1*S*,3*R*)-5-methyl-1,3-diphenyl-2,3-dihydrophosphindole 1-oxide (3ba)

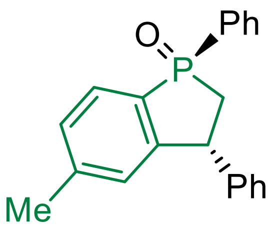

White solid, 30.6 mg at 0.20 mmol scale, 48% yield, >20:1 dr, 98% *ee*. **Optical rotation:** $[\alpha]^{25}_D$ -67.7 (c 0.44, CHCl$_3$). The enantiomeric excess was determined by chiral HPLC using CHIRALPAK IB N-5 column, *n*-Hexane/IPA = 88/12, flow rate 1.0 mL/min, uv-vis detection at λ = 210 nm, t_{major} = 17.20 min, t_{minor} = 10.63 min. **^{1}H NMR (400 MHz, CDCl$_3$)** δ 7.67 – 7.43 (m, 6H), 7.38 – 7.35 (m, 2H), 7.31 – 7.22 (m, 4H), 6.86 (s, 1H), 4.36 (td, *J* = 7.7, 2.9 Hz, 1H), 2.95 – 2.87 (m, 1H), 2.59 – 2.48 (m, 1H), 2.33 (s, 3H). **^{13}C NMR (101 MHz, CDCl$_3$)** δ 151.2 (d, *J* = 27.6 Hz), 144.0, 143.9, 133.5 (d, *J* = 98.6 Hz), 132.0 (d, *J* = 2.4 Hz), 130.6 (d, *J* =

10.5 Hz), 130.2, 129.6 (d, J = 10.9 Hz), 129.1, 128.9 (d, J = 12.0 Hz), 128.8 (d, J = 8.9 Hz), 128.4, 127.5 (d, J = 12.7 Hz), 127.4, 46.6 (d, J = 5.3 Hz), 39.1 (d, J = 68.2 Hz), 21.9. **^{31}P NMR (162 MHz, CDCl$_3$)** δ 49.7. **HRMS-ESI (m/z):** calcd for $C_{21}H_{20}OP^+[M+H]^+$ 319.1246, found 319.1246.

(1*S*,3*R*)-5-methoxy-1,3-diphenyl-2,3-dihydrophosphindole 1-oxide (3ca)

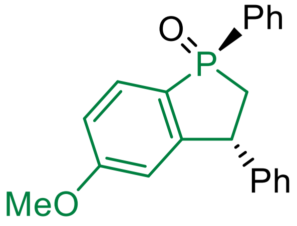

White solid, 31.4 mg at 0.20 mmol scale, 47% yield, >20:1 dr, 95% *ee*. **Optical rotation:** $[\alpha]^{25}_D$ -75.3 (c 0.45, CHCl$_3$). The enantiomeric excess was determined by chiral HPLC using CHIRALPAK IB N-5 column, *n*-Hexane/IPA = 85/15, flow rate 1.0 mL/min, uv-vis detection at λ = 210 nm, t_{major} = 16.23 min, t_{minor} = 11.18 min. **^{1}H NMR (400 MHz, CDCl$_3$)** δ 7.67 (t, J = 8.5 Hz, 1H), 7.62 – 7.56 (m, 2H), 7.54 – 7.43 (m, 3H), 7.38 – 7.32 (m, 2H), 7.30 – 7.28 (m, 3H), 6.96 (d, J = 8.4 Hz, 1H), 6.52 (s, 1H), 4.35 (td, J = 7.8, 3.1 Hz, 1H), 3.74 (s, 3H), 2.94 – 2.87 (m, 1H), 2.54 (ddd, J = 18.8, 15.5, 8.1 Hz, 1H). **^{13}C NMR (101 MHz, CDCl$_3$)** δ 163.9, 153.5 (d, J = 29.0 Hz), 143.7 (d, J = 8.5 Hz), 132.0, 130.7, 130.6, 130.5, 130.4, 129.2, 128.9 (d, J = 12.0 Hz), 128.4, 127.4, 115.5 (d, J = 11.3 Hz), 111.5 (d, J = 13.1 Hz), 55.6, 46.7 (d, J = 4.5 Hz), 39.4 (d, J = 67.6 Hz). **^{31}P NMR (162 MHz, CDCl$_3$)** δ 48.7. **HRMS-ESI (m/z):** calcd for $C_{21}H_{20}O_2P^+[M+H]^+$ 335.1195, found 335.1193.

(1*S*,3*R*)-5-chloro-1,3-diphenyl-2,3-dihydrophosphindole 1-oxide (3da)

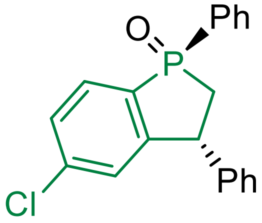

White solid, 31.2 mg at 0.20 mmol scale, 46% yield, >20:1 dr, 97% *ee*. **Optical rotation:** $[\alpha]^{25}_D$ -88.5 (c 0.44, CHCl$_3$). The enantiomeric excess was determined by chiral HPLC using CHIRALPAK IB N-5 column, *n*-Hexane/IPA = 88/12, flow rate 1.0 mL/min, uv-vis detection at λ = 210 nm, t_{major} = 19.47 min, t_{minor} = 12.07 min. **^{1}H NMR (400 MHz, CDCl$_3$)** δ 7.69 (t, J = 8.3 Hz, 1H), 7.62 – 7.53 (m, 3H), 7.50 – 7.46 (m, 2H), 7.41 – 7.36 (m, 3H), 7.33 – 7.30 (m, 1H), 7.27 – 7.25 (m, 2H), 7.05 (s, 1H), 4.37 (td, J = 7.7, 3.9 Hz, 1H), 3.0 – 2.92 (m, 1H), 2.59 (ddd, J = 19.0, 15.6, 8.2 Hz, 1H). **^{13}C NMR (101 MHz, CDCl$_3$)** δ 152.8 (d, J = 28.4 Hz), 142.9 (d, J = 8.8 Hz), 139.9 (d, J = 1.9 Hz), 132.7 (d, J = 131.7 Hz), 132.4 (d, J = 1.0

Hz), 131.0, 130.5 (d, J = 10.4 Hz), 130.2 (d, J = 9.4 Hz), 129.4, 129.07 (d, J = 10.7 Hz), 129.04 (d, J = 12.2 Hz),128.3, 127.7, 127.3 (d, J = 12.7 Hz), 46.6 (d, J = 4.1 Hz), 39.1 (d, J = 67.8 Hz). **^{31}P NMR (162 MHz, CDCl$_3$)** δ 48.5. **HRMS-ESI (m/z):** calcd for $C_{20}H_{17}ClOP^+$[M+H]$^+$ 339.0700, found 339.0703.

(1*S*,3*R*)-4-fluoro-1,3-diphenyl-2,3-dihydrophosphindole 1-oxide (3ea)

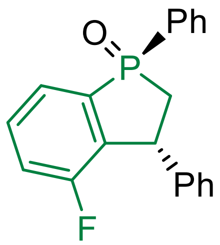

White solid, 27.0 mg at 0.20 mmol scale, 42% yield, >20:1 dr, 98% *ee*. **Optical rotation:** $[\alpha]^{25}_D$ -75.4 (c 0.27, CHCl$_3$). The enantiomeric excess was determined by chiral HPLC using CHIRALPAK IB N-5 column, *n*-Hexane/IPA = 85/15, flow rate 1.0 mL/min, uv-vis detection at λ = 210 nm, t_{major} = 14.19 min, t_{minor} = 10.30 min. **^{1}H NMR (400 MHz, CDCl$_3$)** δ 7.65 – 7.41 (m, 7H), 7.37 – 7.31 (m, 4H), 7.28 – 7.22 (m, 1H), 7.21 – 7.15 (m, 1H), 4.81 – 4.71 (m, 1H), 2.98 – 2.88 (m, 1H), 2.51 (td, J = 16.5, 4.6 Hz, 1H). **^{13}C NMR (101 MHz, CDCl$_3$)** δ 159.9 (dd, J = 254.7, 16.9 Hz), 143.5 (d, J = 4.0 Hz), 134.1, 132.4 (d, J = 1.9 Hz), 131.3 (d, J = 6.6 Hz), 131.2 (d, J = 6.9 Hz), 130.9 (d, J = 10.4 Hz), 129.0, 128.9, 127.6, 127.3, 124.9 (d, J = 4.2 Hz), 124.8 (d, J = 4.0 Hz), 120.1 (d, J = 20.7 Hz), 44.0, 37.9 (d, J = 67.8 Hz). **^{31}P NMR (162 MHz, CDCl$_3$)** δ 48.5. **HRMS-ESI (m/z):** calcd for $C_{20}H_{17}FOP^+$[M+H]$^+$ 323.0996, found 323.0993.

(1*S*,3*R*)-6-chloro-1,3-diphenyl-2,3-dihydrophosphindole 1-oxide (3fa)

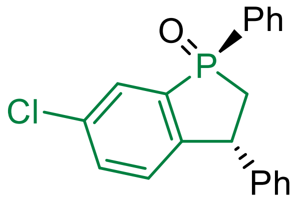

White solid, 29.8 mg at 0.20 mmol scale, 44% yield, >20:1 dr, 93% *ee*. **Optical rotation:** $[\alpha]^{25}_D$ -44.2 (c 0.46, CHCl$_3$). The enantiomeric excess was determined by chiral HPLC using CHIRALPAK IB N-5 column, *n*-Hexane/IPA = 85/15, flow rate 1.0 mL/min, uv-vis detection at λ = 210 nm, t_{major} = 16.49 min, t_{minor} = 9.04 min. **^{1}H NMR (400 MHz, CDCl$_3$)** δ 7.71 (dd, J = 9.3, 2.0 Hz, 1H), 7.64 – 7.54 (m, 3H), 7.51 – 7.43 (m, 3H), 7.38 – 7.24 (m, 5H), 7.01 (dd, J = 8.3, 2.3 Hz, 1H), 4.39 (td, J = 7.9, 3.5 Hz, 1H), 3.02 – 2.94 (m, 1H), 2.58 (ddd, J = 19.2, 15.6, 8.1 Hz, 1H). **^{13}C NMR (101 MHz, CDCl$_3$)** δ 149.0 (d, J = 26.9 Hz), 143.3 (d, J = 8.9 Hz), 135.3 (d, J = 99.9 Hz), 134.7 (d, J = 13.5 Hz), 133.5 (d, J = 1.2 Hz), 132.6 (d, J = 99.2 Hz), 132.4 (d, J =

2.2 Hz), 130.6 (d, J = 10.5 Hz), 129.3, 129.1 (d, J = 12.1 Hz), 128.7 (d, J = 4.6 Hz), 128.6 (d, J = 8.2 Hz), 128.3, 127.7, 46.4, 39.2 (d, J = 68.2 Hz). **^{31}P NMR (162 MHz, CDCl$_3$)** δ 48.6. **HRMS-ESI (m/z):** calcd for $C_{20}H_{17}ClOP^+[M+H]^+$ 339.0700, found 339.0702.

(1*S*,3*R*)-7-methyl-1,3-diphenyl-2,3-dihydrophosphindole 1-oxide (3ga)

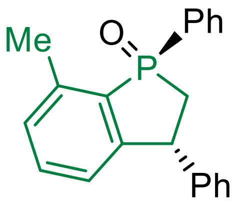

White solid, 30.6 mg at 0.20 mmol scale, 35% yield, >20:1 dr, 85% *ee*. **Optical rotation:** $[\alpha]^{25}_D$ -123.5 (c 0.34, CHCl$_3$). The enantiomeric excess was determined by chiral HPLC using CHIRALPAK IB N-5 column, *n*-Hexane/IPA = 92/8, flow rate 1.0 mL/min, uv-vis detection at λ = 210 nm, t_{major} = 16.98 min, t_{minor} = 11.64 min. **^{1}H NMR (400 MHz, CDCl$_3$)** δ 7.60 (dd, J = 12.1, 7.1 Hz, 2H), 7.54 – 7.43 (m, 3H), 7.38 – 7.32 (m, 3H), 7.28 – 7.24 (m, 3H), 7.13 (dd, J = 7.2, 3.8 Hz, 1H), 6.86 (d, J = 7.8 Hz, 1H), 4.36 (td, J = 7.9, 3.0 Hz, 1H), 2.92 – 2.85 (m, 1H), 2.53 (ddd, J = 19.0, 15.6, 8.0 Hz, 1H), 2.42 (s, 3H). **^{13}C NMR (101 MHz, CDCl$_3$)** δ 151.2 (d, J = 27.8 Hz), 144.2 (d, J = 8.5 Hz), 141.3 (d, J = 8.0 Hz), 133.4, 132.04 (d, J = 1.8 Hz), 130.5 (d, J = 10.5 Hz), 129.4 (d, J = 9.1 Hz), 129.1, 129.0, 128.9, 128.4, 127.3, 124.5 (d, J = 12.3 Hz), 46.5 (d, J = 5.0 Hz), 39.1 (d, J = 68.4 Hz), 19.6 (d, J = 3.8 Hz). **^{31}P NMR (162 MHz, CDCl$_3$)** δ 50.8. **HRMS-ESI (m/z):** calcd for $C_{21}H_{20}OP^+[M+H]^+$ 319.1246, found 319.1242.

(1*S*,3*R*)-1,3-diphenyl-5-(trifluoromethyl)-2,3-dihydrophosphindole 1-oxide (3ha)

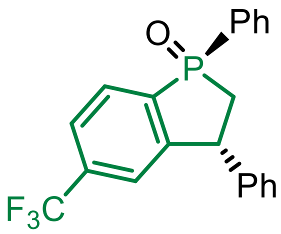

White solid, 31.7 mg at 0.20 mmol scale, 43% yield, >20:1 dr, 95% *ee*. **Optical rotation:** $[\alpha]^{25}_D$ -111.4 (c 0.11, CHCl$_3$). The enantiomeric excess was determined by chiral HPLC using CHIRALPAK IB N-5 column, *n*-Hexane/IPA = 88/12, flow rate 1.0 mL/min, uv-vis detection at λ = 210 nm, t_{major} = 20.05 min, t_{minor} = 9.24 min. **^{1}H NMR (400 MHz, CDCl$_3$)** δ 8.03 (d, J = 8.8 Hz, 1H), 7.72 (d, J = 8.2 Hz, 1H), 7.65 – 7.48 (m, 5H), 7.40 – 7.30 (m, 3H), 7.27 (d, J = 1.3 Hz, 2H), 7.21 (d, J = 8.2 Hz, 1H), 4.48 (t, J = 6.5 Hz, 1H), 3.03 (dt, J = 14.5, 7.6 Hz, 1H), 2.63 (ddd, J = 19.1, 15.7, 8.1 Hz, 1H). **^{13}C NMR (101 MHz, CDCl$_3$)** δ 150.8 (d, J = 27.2 Hz), 143.8 (d, J = 8.8 Hz), 133.7 (d, J = 41.5 Hz), 133.2 (d, J = 1.5 Hz), 132.7 (d, J =

44.6 Hz), 132.1 (d, J = 2.2 Hz), 130.6 (d, J = 10.4 Hz), 129.2, 129.1, 128.9 (d, J = 11.9 Hz), 128.4, 128.3, 127.4, 127.1 (d, J = 12.2 Hz), 46.8 (d, J = 5.2 Hz), 39.0 (d, J = 68.0 Hz). **^{31}P NMR (162 MHz, CDCl$_3$)** δ 47.2. **HRMS-ESI (m/z):** calcd for $C_{21}H_{17}F_3OP^+$[M+H]$^+$ 373.0964, found 373.0941.

(1*S*,3*R*)-6-nitro-1,3-diphenyl-2,3-dihydrophosphindole 1-oxide (3ia)

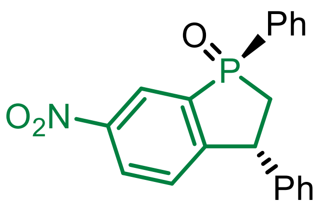

White solid, 28.6 mg at 0.20 mmol scale, 41% yield, >20:1 dr, 92% *ee*. **Optical rotation:** $[\alpha]^{25}_D$ -46.5 (c 0.12, CHCl$_3$). The enantiomeric excess was determined by chiral HPLC using CHIRALPAK IB N-5 column, *n*-Hexane/IPA = 88/12, flow rate 1.0 mL/min, uv-vis detection at λ = 210 nm, t_{major} = 40.80 min, t_{minor} = 35.81 min. **^{1}H NMR (400 MHz, CDCl$_3$)** δ 8.24 (d, J = 8.1 Hz, 1H), 7.94 (t, J = 8.2 Hz, 1H), 7.89 (s, 1H), 7.60 (dt, J = 15.5, 7.7 Hz, 3H), 7.50 (t, J = 7.0 Hz, 2H), 7.42 – 7.32 (m, 3H), 7.27 (d, J = 7.3 Hz, 2H), 4.50 (q, J = 5.1 Hz, 1H), 3.09 (dt, J = 14.5, 7.1 Hz, 1H), 2.70 (ddd, J = 19.1, 15.9, 8.1 Hz, 1H). **^{13}C NMR (101 MHz, CDCl$_3$)** δ 152.5 (d, J = 28.6 Hz), 151.49, 142.3 (d, J = 8.2 Hz), 140.1 (d, J = 96.9 Hz), 132.8, 130.5, 130.4, 130.3, 129.6, 129.3 (d, J = 12.1 Hz), 128.3, 128.2, 123.5 (d, J = 10.4 Hz), 122.2 (d, J = 12.2 Hz), 46.8 (d, J = 2.1 Hz), 39.05 (d, J = 67.7 Hz). **^{31}P NMR (162 MHz, CDCl$_3$)** δ 46.3. **HRMS-ESI (m/z):** calcd for $C_{20}H_{17}NO_3P^+$[M+H]$^+$ 350.0941, found 350.0911.

(1*S*,3*R*)-1-cyclohexyl-3-(*p*-tolyl)-2,3-dihydrophosphindole 1-oxide (3jb)

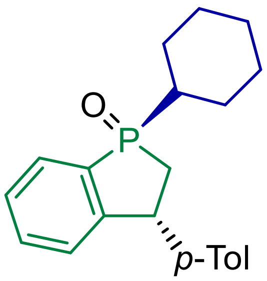

White solid, 23.2 mg at 0.20 mmol scale, 48% yield, >20:1 dr, 91% *ee*. **Optical rotation:** $[\alpha]^{25}_D$ -46.2 (c 0.05, CHCl$_3$). The enantiomeric excess was determined by chiral HPLC using CHIRALPAK IB N-5 column, *n*-Hexane/IPA = 88/12, flow rate 1.0 mL/min, uv-vis detection at λ = 210 nm, t_{major} = 9.75 min, t_{minor} = 8.73 min. **^{1}H NMR (400 MHz, CDCl$_3$)** δ 7.78 (t, J = 7.5 Hz, 1H), 7.46 – 7.34 (m, 2H), 7.15 (d, J = 8.0 Hz, 2H), 7.09 (d, J = 8.1 Hz, 2H), 6.95 (d, J = 7.3 Hz, 1H), 4.19 (t, J = 8.4 Hz, 1H), 2.87 (ddd, J = 15.4, 7.8, 4.6 Hz, 1H), 2.34 (s, 3H), 2.22 (ddd, J = 19.3, 14.8, 8.2 Hz, 1H), 2.05 – 1.98 (m, 2H), 1.94 – 1.82 (m, 2H), 1.53 – 1.45 (m, 1H), 1.41 – 1.14 (m, 6H). **^{13}C NMR (101 MHz, CDCl$_3$)** δ

149.3 (d, J = 24.5 Hz), 140.1 (d, J = 8.9 Hz), 136.0, 131. 7, 128.7, 128.2, 128.1, 127.3, 126.7 (d, J = 9.4 Hz), 126.1 (d, J = 11.3 Hz), 52.5, 45.5 (d, J = 4.2 Hz), 25.3 (d, J = 13.4 Hz), 24.9, 24.5, 24.1, 20.1. **^{31}P NMR (162 MHz, CDCl$_3$)** δ 61.3. **HRMS-ESI (m/z):** calcd for $C_{21}H_{26}OP^+[M+H]^+$ 325.1716, found 325.1710.

(1*S*,3*R*)-1-(*tert*-butyl)-3-(*p*-tolyl)-2,3-dihydrophosphindole 1-oxide (3kb)

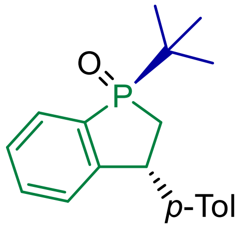

White solid, 18.0 mg at 0.20 mmol scale, 40% yield, >20:1 dr, 90% *ee*. **Optical rotation:** $[\alpha]^{25}_D$ -10.5 (c 0.26, CHCl$_3$). The enantiomeric excess was determined by chiral HPLC using CHIRALPAK IA column, *n*-Hexane/IPA = 85/15, flow rate 1.0 mL/min, uv-vis detection at λ = 210 nm, t_{major} = 12.82 min, t_{minor} = 9.30 min. **^{1}H NMR (400 MHz, CDCl$_3$)** δ 7.82 – 7.72 (m, 1H), 7.45 – 7.34 (m, 2H), 7.14 (d, J = 8.0 Hz, 2H), 7.08 (d, J = 8.1 Hz, 2H), 6.95 (d, J = 7.9 Hz, 1H), 4.20 (t, J = 8.4 Hz, 1H), 2.91 (ddd, J = 15.7, 8.1, 4.0 Hz, 1H), 2.33 (s, 3H), 2.20 (ddd, J = 19.4, 15.8, 8.7 Hz, 1H), 1.23 (d, J = 15.0 Hz, 9H). **^{13}C NMR (101 MHz, CDCl$_3$)** δ 150.7 (d, J = 24.4 Hz), 141.2 (d, J = 8.3 Hz), 136.6, 132.4, 130.7 (d, J = 91.4 Hz), 129.4, 129.1 (d, J = 7.8 Hz), 128.0, 127.3 (d, J = 9.5 Hz), 126.8 (d, J = 11.2 Hz), 47.0 (d, J = 4.1 Hz), 33.6 (d, J = 59.3 Hz), 33.5 (d, J = 68.8 Hz), 23.6, 20.8. **^{31}P NMR (162 MHz, CDCl$_3$)** δ 69.0. **HRMS-ESI (m/z):** calcd for $C_{19}H_{24}OP^+[M+H]^+$ 299.1559, found 299.1555.

(1*R*,3*R*)-1-methyl-3-(*p*-tolyl)-2,3-dihydrophosphindole 1-oxide (lb)

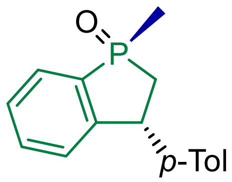

White solid, 20.5 mg at 0.20 mmol scale, 40% yield, >20:1 dr, 88% *ee*. **Optical rotation:** $[\alpha]^{25}_D$ -59.1 (c 0.07, CHCl$_3$). The enantiomeric excess was determined by chiral HPLC using CHIRALPAK IB N-5 column, *n*-Hexane/IPA = 85/15, flow rate 1.0 mL/min, uv-vis detection at λ = 210 nm, t_{major} = 13.65 min, t_{minor} = 10.31 min. **^{1}H NMR (400 MHz, CDCl$_3$)** δ 7.83 (t, J = 7.7 Hz, 1H), 7.45 – 7.38 (m, 2H), 7.16 (d, J = 7.9 Hz, 2H), 7.11 (d, J = 8.1 Hz, 2H), 6.97 (d, J = 7.0 Hz, 1H), 4.24 (t, J = 8.0 Hz, 1H), 2.86 – 2.75 (m, 1H), 2.41 – 2.31 (m, 4H), 1.78 (d, J = 12.8 Hz, 3H). **^{13}C NMR (101 MHz, CDCl$_3$)** δ 149.1 (d, J = 26.6 Hz), 140.5 (d, J = 9.2 Hz), 137.1, 133.9 (d, J = 97.4 Hz), 132.8, 129.7, 128.2, 128.0 (d, J = 15.9 Hz), 127.9, 127.0 (d, J = 11.7 Hz), 45.9 (d, J =

4.8 Hz), 38.4 (d, J = 66.1 Hz), 21.1, 16.8 (d, J = 67.4 Hz). **^{31}P NMR (162 MHz, $CDCl_3$)** δ 53. **HRMS-ESI (m/z):** calcd for $C_{16}H_{18}OP^{+}[M+H]^{+}$ 257.1090, found 257.1081.

(1*R*,3*R*)-1-ethyl-3-phenyl-2,3-dihydrophosphindole 1-oxide (ma)

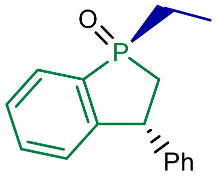

White solid, 20.6 mg at 0.20 mmol scale, 40% yield, >20:1 dr, 87% *ee*. **Optical rotation:** $[\alpha]^{25}_{D}$ -44.3 (c 0.15, $CHCl_3$). The enantiomeric excess was determined by chiral HPLC using CHIRALPAK IB N-5 column, *n*-Hexane/IPA = 85/15, flow rate 1.0 mL/min, uv-vis detection at λ = 210 nm, t_{major} = 11.76 min, t_{minor} = 9.83 min. **^{1}H NMR (400 MHz, DMSO-d_6)** δ 7.74 (t, J = 7.8 Hz, 1H), 7.54 – 7.33 (m, 4H), 7.27 (d, J = 7.1 Hz, 3H), 6.93 (d, J = 7.5 Hz, 1H), 4.47 (q, J = 7.5 Hz, 1H), 2.92 – 2.84 (m, 1H), 1.99 (ddd, J = 19.9, 13.7, 7.6 Hz, 3H), 1.09 (dt, J = 17.5, 7.6 Hz, 3H). **^{13}C NMR (101 MHz, $CDCl_3$)** δ 150.0 (d, J = 25.8 Hz), 143.9 (d, J = 9.0 Hz), 132.81 (d, J = 1.7 Hz), 132.76 (d, J = 98.0 Hz), 129.1, 128.6 (d, J = 8.3 Hz), 128.4, 127.9 (d, J = 10.0 Hz), 127.4, 127.1 (d, J = 11.7 Hz), 46.6 (d, J = 5.1 Hz), 36.4 (d, J = 64.0 Hz), 23.7 (d, J = 68.1 Hz), 6.3 (d, J = 4.6 Hz). **^{31}P NMR (162 MHz, $CDCl_3$)** δ 58.8. **HRMS-ESI (m/z):** calcd for $C_{16}H_{18}OP^{+}[M+H]^{+}$ 257.1090, found 257.1069.

(*R*)-1-phenylphosphindole 1-oxide (1a)

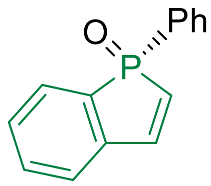

White solid, 22.3 mg at 0.20 mmol scale, 47% yield, >99% *ee*. **Optical rotation:** $[\alpha]^{25}_{D}$ -44.0 (c 0.05, $CHCl_3$). The enantiomeric excess was determined by chiral HPLC using CHIRALPAK IB N-5 column, *n*-Hexane/IPA = 88/12, flow rate 1.0 mL/min, uv-vis detection at λ = 210 nm, t_{major} = 16.94 min, t_{minor} = 18.04 min. **^{1}H NMR (400 MHz, $CDCl_3$)** δ 7.74 – 7.68 (m, 2H), 7.63 – 7.59 (m, 1H), 7.54 – 7.34 (m, 7H), 6.45 (dd, J = 25.6, 8.5 Hz, 1H). **^{13}C NMR (101 MHz, $CDCl_3$)** δ 145.4 (d, J = 12.9 Hz), 142.1 (d, J = 31.1 Hz), 133.1 (d, J = 1.4 Hz), 132.4 (d, J = 2.7 Hz), 132.4 (d, J = 107.8 Hz), 131.0 (d, J = 10.9 Hz), 129.8 (d, J = 10.3 Hz), 129.1 (d, J = 10.5 Hz), 129.0 (d, J = 12.4 Hz), 127.3, 126.3, 124.9 (d, J = 10.0 Hz). **^{31}P NMR (162 MHz, $CDCl_3$)** δ 41.7. **HRMS-ESI (m/z):** calcd for $C_{14}H_{12}OP^{+}[M+H]^{+}$ 227.0620, found 227.0617.

(*R*)-5-methyl-1-phenylphosphindole 1-oxide (1b)

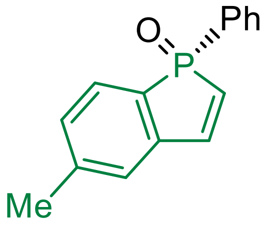

White solid, 22.6 mg at 0.20 mmol scale, 47% yield, 94% *ee*. **Optical rotation:** $[\alpha]^{25}_{D}$ -1.3 (c 1.2, $CHCl_3$). The enantiomeric excess was determined by chiral HPLC using CHIRALPAK IB N-5 column, *n*-Hexane/IPA = 88/12, flow rate 1.0 mL/min, uv-vis detection at λ = 210 nm, t_{major} = 15.32 min, t_{minor} = 16.90 min. **^{1}H NMR (400 MHz, $CDCl_3$)** δ 7.69 – 7.64 (m, 2H), 7.48 – 7.43 (m, 2H), 7.39 – 7.27 (m, 3H), 7.12 (t, *J* = 4.3 Hz, 2H), 6.38 (dd, *J* = 25.6, 8.4 Hz, 1H), 2.35 (s, 3H). **^{13}C NMR (101 MHz, $CDCl_3$)** δ 145.3 (d, *J* = 13.1 Hz), 143.7 (d, *J* = 1.8 Hz), 142.4 (d, *J* = 31.6 Hz), 132.2 (d, *J* = 2.7 Hz), 130.8 (d, *J* = 10.9 Hz), 130.1 (d, *J* = 10.8 Hz), 129.8 (d, *J* = 53.2 Hz), 128.9 (d, *J* = 10.8 Hz), 128.76 (d, *J* = 12.5 Hz), 128.70 (d, *J* = 62.2 Hz),126.9 (d, *J* = 96.3 Hz), 125.8 (d, *J* = 10.4 Hz), 21.72. **^{31}P NMR (162 MHz, $CDCl_3$)** δ 41.4. **HRMS-ESI (m/z):** calcd for $C_{15}H_{14}OP^+[M+H]^+$ 241.0777, found 241.0780.

(*R*)-5-methoxy-1-phenylphosphindole 1-oxide (1c)

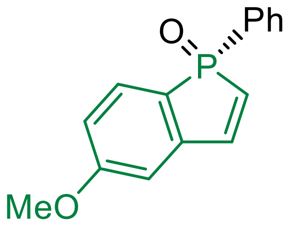

White solid, 24.6 mg at 0.20 mmol scale, 48% yield, >99% *ee*. **Optical rotation:** $[\alpha]^{25}_{D}$ -282.8 (c 0.18, $CHCl_3$). The enantiomeric excess was determined by chiral HPLC using CHIRALPAK IJ column, *n*-Hexane/IPA = 80/20, flow rate 1.0 mL/min, uv-vis detection at λ = 210 nm, t_{major} = 9.95 min, t_{minor} = 13.61 min. **^{1}H NMR (400 MHz, $CDCl_3$)** δ 7.71 – 7.65 (m, 2H), 7.53 – 7.47 (m, 2H), 7.42 – 7.26 (m, 3H), 6.88 – 6.80 (m, 2H), 6.49 – 6.40 (m, 1H), 3.83 (s, 3H). **^{13}C NMR (101 MHz, $CDCl_3$)** δ 163.9 (d, *J* = 1.8 Hz), 144.64 (d, *J* = 12.7 Hz), 144.57 (d, *J* = 32.9 Hz), 132.3 (d, *J* = 2.7 Hz), 131.0 (d, *J* = 10.9 Hz), 130.6 (d, *J* = 12.0 Hz), 129.8 (d, *J* = 102.4 Hz), 128.8 (d, *J* = 12.3 Hz), 127.8, 123.0 (d, *J* = 114.3 Hz), 113.7 (d, *J* = 11.6 Hz), 112.0 (d, *J* = 11.0 Hz), 55.7. **^{31}P NMR (162 MHz, $CDCl_3$)** δ 40.5. **HRMS-ESI (m/z):** calcd for $C_{15}H_{14}O_2P^+[M+H]^+$ 257.0726, found 257.0723.

(*R*)-5-chloro-1-phenylphosphindole 1-oxide (1d)

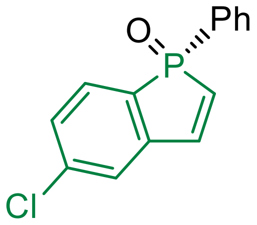

White solid, 22.9 mg at 0.20 mmol scale, 44% yield, 97% *ee*. **Optical rotation:** $[\alpha]^{25}_{D}$ -3.4 (c 0.74, $CHCl_3$). The enantiomeric excess was determined by chiral HPLC using CHIRALPAK IB N-5 column, *n*-Hexane/IPA = 88/12, flow rate 1.0 mL/min, uv-vis detection at λ = 210 nm, t_{major} = 15.32 min, t_{minor} = 17.37 min. **^{1}H NMR (400 MHz, $CDCl_3$)** δ 7.68 (dd, J = 12.7, 7.5 Hz, 2H), 7.54 – 7.31 (m, 7H), 6.56 – 6.47 (m, 1H). **^{13}C NMR (101 MHz, $CDCl_3$)** δ 144.2 (d, J = 11.9 Hz), 143.7, 139.5, 132.7, 131.2, 130.9 (d, J = 10.8 Hz), 130.1 (d, J = 11.5 Hz), 129.6 (d, J = 10.8 Hz), 129.1 (d, J = 12.5 Hz), 128.2, 128.1, 125.3 (d, J = 10.4 Hz). **^{31}P NMR (162 MHz, $CDCl_3$)** δ 40.5. **HRMS-ESI (m/z):** calcd for $C_{14}H_{11}ClOP^+[M+H]^+$ 261.0231, found 261.0235.

(*R*)-4-fluoro-1-phenylphosphindole 1-oxide (1e)

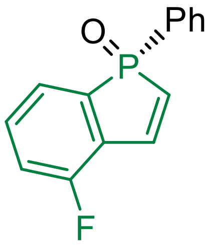

White solid, 23.5 mg at 0.20 mmol scale, 48% yield, >99% *ee*. **Optical rotation:** $[\alpha]^{25}_{D}$ -175.0 (c 0.09, $CHCl_3$). The enantiomeric excess was determined by chiral HPLC using CHIRALPAK IJ column, *n*-Hexane/IPA = 85/15, flow rate 1.0 mL/min, uv-vis detection at λ = 210 nm, t_{major} = 16.86 min, t_{minor} = 13.14 min. **^{1}H NMR (400 MHz, $CDCl_3$)** δ 7.74 – 7.65 (m, 3H), 7.58 – 7.52 (m, 1H), 7.45 – 7.36 (m, 4H), 7.21 – 7.17 (m, 1H), 6.48 (dd, J = 26.3, 8.6 Hz, 1H). **^{13}C NMR (101 MHz, $CDCl_3$)** δ 157.4 (dd, J = 256.1, 15.1 Hz), 138.9 (d, J = 12.0 Hz), 135.0 (d, J = 105.4 Hz), 132.7 (d, J = 2.7 Hz), 132.1 (dd, J = 12.5, 6.5 Hz), 130.9 (d, J = 10.9 Hz), 129.1 (d, J = 12.6 Hz), 128.6 (dd, J = 32.6, 15.2 Hz), 128.1, 127.0 (d, J = 96.7 Hz), 125.0 (dd, J = 10.0, 3.3 Hz), 120.6 (d, J = 20.6 Hz). **^{31}P NMR (162 MHz, $CDCl_3$)** δ 41.0. **HRMS-ESI (m/z):** calcd for $C_{14}H_{11}FOP^+[M+H]^+$ 245.0526, found 245.0522.

(*R*)-6-chloro-1-phenylphosphindole 1-oxide (1f)

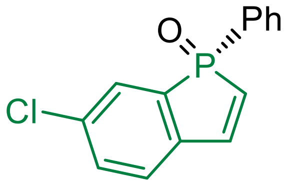

White solid, 24.4 mg at 0.20 mmol scale, 47% yield, >99% *ee*. **Optical rotation:** $[\alpha]^{25}_{D}$ -173.3 (c 0.06, $CHCl_3$). The enantiomeric excess was determined by chiral HPLC using CHIRALPAK IJ column, *n*-Hexane/IPA = 90/10, flow rate 1.0 mL/min, uv-vis detection at λ = 210 nm, t_{major} = 10.83 min, t_{minor} = 13.15 min. **^{1}H NMR (400 MHz, $CDCl_3$)** δ 7.74 – 7.69 (m, 2H), 7.57 – 7.28 (m, 7H), 6.52 – 6.43 (m, 1H). **^{13}C NMR (101 MHz, $CDCl_3$)** δ 144.7 (d, J = 11.9 Hz), 140.2 (d, J = 30.7 Hz), 136.1 (d, J = 13.7 Hz), 134.7 (d, J = 105.4 Hz), 133.0 (d, J = 0.9 Hz), 132.8 (d, J = 2.5 Hz), 130.9 (d, J = 11.0 Hz), 129.4 (d, J = 11.4 Hz), 129.1 (d, J = 12.6 Hz), 128.4 (d, J = 102.4 Hz), 127.0 (d, J = 96.5 Hz), 125.9 (d, J = 10.9 Hz). **^{31}P NMR (162 MHz, $CDCl_3$)** δ 40.3. **HRMS-ESI (m/z):** calcd for $C_{14}H_{11}ClOP^+[M+H]^+$ 261.0231, found 261.0237.

(*R*)-7-methyl-1-phenylphosphindole 1-oxide (1g)

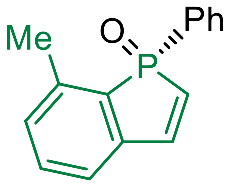

White solid, 22.5 mg at 0.20 mmol scale, 47% yield, >99% *ee*. **Optical rotation:** $[\alpha]^{25}_{D}$ +31.0 (c 0.36, $CHCl_3$). The enantiomeric excess was determined by chiral HPLC using CHIRALPAK IB N-5 column, *n*-Hexane/IPA = 88/12, flow rate 1.0 mL/min, uv-vis detection at λ = 210 nm, t_{major} = 10.93 min, t_{minor} = 12.65 min. **^{1}H NMR (400 MHz, $CDCl_3$)** δ 7.75 – 7.70 (m, 2H), 7.52 (td, J = 7.3, 1.4 Hz, 1H), 7.44 – 7.32 (m, 4H), 7.12 (ddd, J = 22.9, 7.5, 3.9 Hz, 2H), 6.38 (dd, J = 25.8, 8.5 Hz, 1H), 2.33 (s, 3H). **^{13}C NMR (101 MHz, $CDCl_3$)** δ 145.4 (d, J = 13.3 Hz), 142.3 (d, J = 31.6 Hz), 141.3 (d, J = 9.8 Hz), 133.4 (d, J = 1.3 Hz), 132.3 (d, J = 2.7 Hz), 131.4 (d, J = 9.1 Hz), 131.0 (d, J = 10.9 Hz), 129.6 (d, J = 53.0 Hz), 128.9 (d, J = 12.3 Hz), 128.4, 126.7 (d, J = 96.4 Hz), 122.5 (d, J = 10.0 Hz), 19.4. **^{31}P NMR (162 MHz, $CDCl_3$)** δ 42.1. **HRMS-ESI (m/z):** calcd for $C_{15}H_{14}OP^+[M+H]^+$ 241.0777, found 241.0776.

(*R*)-1-phenyl-5-(trifluoromethyl)phosphindole 1-oxide (1h)

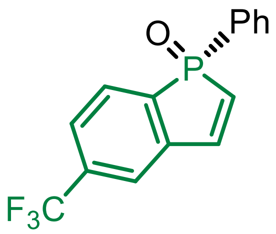

White solid, 26.3 mg at 0.20 mmol scale, 44% yield, >99% *ee*. **Optical rotation:** $[\alpha]^{25}_{D}$ -221.1 (c 0.09, $CHCl_3$). The enantiomeric excess was determined by chiral HPLC using CHIRALPAK IB N-5 column, *n*-Hexane/IPA = 88/12, flow rate 1.0 mL/min, uv-vis detection at λ = 210 nm, t_{major} = 11.36 min, t_{minor} = 13.89 min. **^{1}H NMR (400 MHz, $CDCl_3$)** δ 7.83 (d, *J* = 9.3 Hz, 1H), 7.76 – 7.68 (m, 3H), 7.58 – 7.42 (m, 5H), 6.61 (dd, *J* = 25.7, 8.4 Hz, 1H). **^{13}C NMR (101 MHz, $CDCl_3$)** δ 145.2 (d, *J* = 30.8 Hz), 144.3 (d, *J* = 11.8 Hz), 133.8 (d, *J* = 106.8 Hz), 133.0 (d, *J* = 2.7 Hz), 131.8 (dd, *J* = 33.1, 10.8 Hz), 130.9 (d, *J* = 11.0 Hz), 130.3 (d, *J* = 40.5 Hz), 129.2 (d, *J* = 12.7 Hz), 129.1, 127.8 (d, *J* = 102.8 Hz), 125.9 (dq, *J* = 11.3, 3.6 Hz), 125.1 (d, *J* = 9.9 Hz). **^{31}P NMR (162 MHz, $CDCl_3$)** δ 39.4. **HRMS-ESI (m/z):** calcd for $C_{15}H_{11}F_3OP^+[M+H]^+$ 295.0494, found 295.0485.

(*R*)-6-nitro-1-phenylphosphindole 1-oxide (1i)

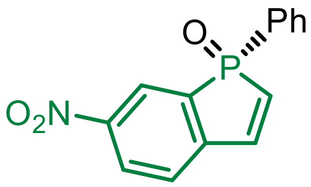

White solid, 24.7 mg at 0.20 mmol scale, 44% yield, 98% *ee*. **Optical rotation:** $[\alpha]^{25}_{D}$ -112.0 (c 0.05, $CHCl_3$). The enantiomeric excess was determined by chiral HPLC using CHIRALPAK IB N-5 column, *n*-Hexane/IPA = 88/12, flow rate 1.0 mL/min, uv-vis detection at λ = 210 nm, t_{major} = 43.26 min, t_{minor} = 50.61 min. **^{1}H NMR (400 MHz, $CDCl_3$)** δ 8.25 – 8.21 (m, 1H), 8.18 (s, 1H), 7.77 (t, *J* = 8.2 Hz, 1H), 7.71 (dd, *J* = 13.1, 7.7 Hz, 2H), 7.60 – 7.43 (m, 4H), 6.68 (dd, *J* = 25.5, 8.5 Hz, 1H). **^{13}C NMR (101 MHz, $CDCl_3$)** δ 151.4, 143.9 (d, *J* = 11.0 Hz), 143.6 (d, *J* = 32.8 Hz), 139.5 (d, *J* = 103.1 Hz), 133.2 (d, *J* = 2.7 Hz), 130.9 (d, *J* = 11.0 Hz), 129.8 (d, *J* = 11.5 Hz), 129.5 (d, *J* = 95.5 Hz), 129.3 (d, *J* = 12.7 Hz), 127.2 (d, *J* = 103.6 Hz), 124.9 (d, *J* = 10.7 Hz), 119.4 (d, *J* = 10.2 Hz). **^{31}P NMR (162 MHz, $CDCl_3$)** δ 38.6. **HRMS-ESI (m/z):** calcd for $C_{14}H_{11}NO_3P^+[M+H]^+$ 272.0471, found 272.0464.

(*S*)-1-cyclohexylphosphindole 1-oxide (1j)

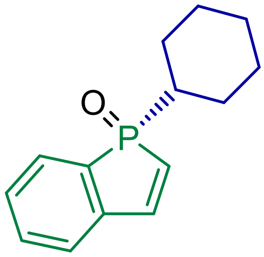

White solid, 23.0 mg at 0.20 mmol scale, 50% yield, 94% *ee*. **Optical rotation:** $[\alpha]^{25}_{D}$ -47.5 (c 0.31, $CHCl_3$). The enantiomeric excess was determined by chiral HPLC using CHIRALPAK IB N-5 column, *n*-Hexane/IPA = 88/12, flow rate 1.0 mL/min, uv-vis detection at λ = 210 nm, t_{major} = 12.64 min, t_{minor} = 12.10 min. **^{1}H NMR (400 MHz, $CDCl_3$)** δ 7.66 (t, J = 7.6 Hz, 1H), 7.46 – 7.42 (m, 1H), 7.38 – 7.24 (m, 3H), 6.33 (dd, J = 25.3, 8.6 Hz, 1H), 2.10 – 2.04 (m, 1H), 1.98 – 1.87 (m, 1H), 1.85 – 1.63 (m, 4H), 1.44 – 1.32 (m, 1H), 1.30 – 1.12 (m, 4H). **^{13}C NMR (101 MHz, $CDCl_3$)** δ 145.4 (d, J = 11.8 Hz), 142.1 (d, J = 28.6 Hz), 132.8, 130.3 (d, J = 99.1 Hz), 129.16 (d, J = 1.3 Hz), 129.15 (d, J = 17.6 Hz), 124.8 (d, J = 9.2 Hz), 124.3 (d, J = 89.0 Hz), 26.3 (d, J = 13.9 Hz), 25.85 (d, J = 1.4 Hz), 25.84 (d, J = 6.9 Hz). **^{31}P NMR (162 MHz, $CDCl_3$)** δ 56.2. **HRMS-ESI (m/z):** calcd for $C_{14}H_{18}OP^+[M+H]^+$ 233.1090, found 233.1083.

(*R*)-1-(*tert*-butyl)phosphindole 1-oxide (1k)

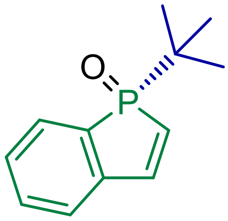

White solid, 17.9 mg at 0.20 mmol scale, 43% yield, >99% *ee*. **Optical rotation:** $[\alpha]^{25}_{D}$ -85.4 (c 0.24, $CHCl_3$). The enantiomeric excess was determined by chiral HPLC using CHIRALPAK IF column, *n*-Hexane/IPA = 85/15, flow rate 1.0 mL/min, uv-vis detection at λ = 210 nm, t_{major} = 21.63 min, t_{minor} = 24.34 min. **^{1}H NMR (400 MHz, $CDCl_3$)** δ 7.67 (t, J = 7.4 Hz, 1H), 7.48 – 7.22 (m, 4H), 6.32 (dd, J = 25.0, 8.6 Hz, 1H), 1.20 (d, J = 15.5 Hz, 9H). **^{13}C NMR (101 MHz, $CDCl_3$)** δ 145.9 (d, J = 11.3 Hz), 142.5 (d, J = 27.5 Hz), 132.7, 130.2, 129.1, 129.1 (d, J = 19.3 Hz), 124.8 (d, J = 8.9 Hz), 123.4 (d, J = 87.0 Hz), 31.9 (d, J = 69.8 Hz), 24.4. **^{31}P NMR (162 MHz, $CDCl_3$)** δ 63.4. **HRMS-ESI (m/z):** calcd for $C_{12}H_{16}OP^+[M+H]^+$ 207.0933, found 207.0929.

(*S*)-1-methylphosphindole 1-oxide (1l)

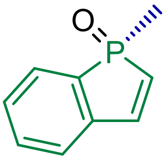

White solid, 10.4 mg at 0.20 mmol scale, 31% yield, >99% *ee*. **Optical rotation:** $[\alpha]^{25}_{D}$ +8.7 (c 0.09, $CHCl_3$). The enantiomeric excess was determined by chiral HPLC using CHIRALPAK IB N-5 column, *n*-

Hexane/IPA = 85/15, flow rate 1.0 mL/min, uv-vis detection at λ = 210 nm, t_{major} = 13.58 min, t_{minor} = 15.11 min. **^{1}H NMR (400 MHz, $CDCl_3$)** δ 7.74 (t, J = 7.9 Hz, 1H), 7.50 – 7.39 (m, 2H), 7.35 – 7.23 (m, 2H), 6.43 (dd, J = 26.3, 8.6 Hz, 1H), 1.77 (d, J = 13.3 Hz, 3H). **^{13}C NMR (101 MHz, $CDCl_3$)** δ 144.1 (d, J = 13.1 Hz), 141.0 (d, J = 31.1 Hz), 132.9 (d, J = 1.5 Hz), 132.2 (d, J = 105.3 Hz), 129.5 (d, J = 10.2 Hz), 128.1 (d, J = 10.6 Hz), 126.4 (d, J = 94.0 Hz), 124.9 (d, J = 9.7 Hz), 15.0 (d, J = 69.7 Hz). **^{31}P NMR (162 MHz, $CDCl_3$)** δ 53.6. **HRMS-ESI (m/z):** calcd for $C_9H_{10}OP^+[M+H]^+$ 165.0464, found 165.0464.

<u>(*S*)-1-ethylphosphindole 1-oxide (1m)</u>

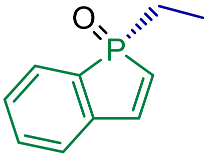

White solid, 14.7 mg at 0.20 mmol scale, 41% yield, >99% *ee*. **Optical rotation:** $[\alpha]^{25}_D$ -12.7 (c 0.07, $CHCl_3$). The enantiomeric excess was determined by chiral HPLC using CHIRALPAK IB N-5 column, *n*-Hexane/IPA = 85/15, flow rate 1.0 mL/min, uv-vis detection at λ = 210 nm, t_{major} = 13.09 min, t_{minor} = 12.11 min. **^{1}H NMR (400 MHz, $CDCl_3$)** δ 7.71 (t, J = 7.7 Hz, 1H), 7.47 (t, J = 7.5 Hz, 1H), 7.42 – 7.28 (m, 3H), 6.37 (dd, J = 25.9, 8.6 Hz, 1H), 2.08 – 1.99 (m, 2H), 1.12 (dt, J = 18.7, 7.7 Hz, 3H). **^{13}C NMR (101 MHz, $CDCl_3$)** δ 145.0 (d, J = 12.1 Hz), 141.7 (d, J = 29.6 Hz), 132.9, 131.0 (d, J = 100.4 Hz), 129.4 (d, J = 9.7 Hz), 128.6 (d, J = 10.3 Hz), 124.9 (d, J = 90.7 Hz), 124.9 (d, J = 9.5 Hz), 22.0 (d, J = 69.1 Hz), 6.6 (d, J = 4.1 Hz). **^{31}P NMR (162 MHz, $CDCl_3$)** δ 46.5. **HRMS-ESI (m/z):** calcd for $C_{10}H_{12}OP^+[M+H]^+$ 179.0620, found 179.0619.

<u>(1*R*,3*R*)-1,3-diphenyl-2,3-dihydro-1*H*-phosphindole (4)</u>

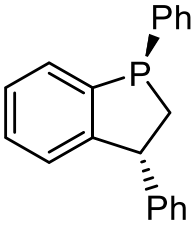

White solid, 27.7 mg at 0.10 mmol scale, 96% yield, >20:1 dr, 94% *ee*. **Optical rotation:** $[\alpha]^{25}_D$ +115.2 (c 0.76, $CHCl_3$). The enantiomeric excess was determined by chiral HPLC using CHIRALPAK IB N-5 column, *n*-Hexane/IPA = 99/1, flow rate 1.0 mL/min, uv-vis detection at λ = 254 nm, t_{major} = 5.73 min, t_{minor} = 6.17 min. **^{1}H NMR (400 MHz, $CDCl_3$)** δ 7.74 (t, J = 5.6 Hz, 1H), 7.33 – 7.13 (m, 12H), 6.91 (d, J = 6.4 Hz, 1H), 4.49 – 4.41 (m, 1H), 2.52 (dd, J = 14.3, 6.8 Hz, 1H), 2.38 (ddd, J = 16.6, 13.1, 10.4 Hz, 1H). **^{13}C NMR (101 MHz, $CDCl_3$)** δ 152.8, 144.8 (d, J = 2.7

Hz), 140.3 (d, J = 7.4 Hz), 139.1 (d, J = 23.0 Hz), 131.7 (d, J = 25.3 Hz), 131.2 (d, J = 16.4 Hz), 129.6, 128.8, 128.6, 128.5 (d, J = 5.2 Hz), 128.2, 127.2 (d, J = 7.8 Hz), 126.9, 126.3, 53.3 (d, J = 6.4 Hz), 38.4 (d, J = 9.6 Hz). **^{31}P NMR (162 MHz, CDCl$_3$)** δ 10.5. **HRMS-ESI (m/z):** calcd for $C_{20}H_{18}P^+[M+H]^+$ 289.1141, found 289.1143.

(1*R*,3*R*)-1-phenyl-3-((*E*)-styryl)-2,3-dihydro-1*H*-phosphindole (5)

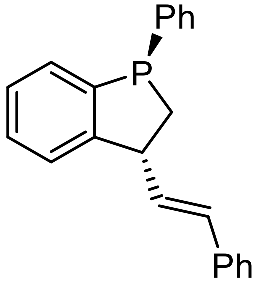

White solid, 30.7 mg at 0.10 mmol scale, 98% yield, >20:1 dr, 95% *ee*. **Optical rotation:** $[\alpha]^{25}_D$ +4.4 (c 0.68, CHCl$_3$). The enantiomeric excess was determined by chiral HPLC using CHIRALPAK IB N-5 column, *n*-Hexane/IPA = 99/1, flow rate 1.0 mL/min, uv-vis detection at λ = 254 nm, t_{major} = 6.62 min, t_{minor} = 7.50 min. **^{1}H NMR (400 MHz, CDCl$_3$)** δ 7.73 (t, J = 6.9 Hz, 1H), 7.40 – 7.15 (m, 13H), 6.51 (d, J = 15.7 Hz, 1H), 6.20 (dd, J = 15.7, 9.0 Hz, 1H), 4.06 (q, J = 9.4 Hz, 1H), 2.37 (dd, J = 14.2, 6.6 Hz, 1H), 2.21 (ddd, J = 16.8, 14.3, 10.0 Hz, 1H). **^{13}C NMR (101 MHz, CDCl$_3$)** δ 151.4, 140.1 (d, J = 8.3 Hz), 139.1 (d, J = 22.6 Hz), 137.3, 132.3 (d, J = 4.3 Hz), 132.0 (d, J = 25.5 Hz), 132.0, 131.2 (d, J = 16.4 Hz), 129.6, 128.7, 128.4 (d, J = 5.6 Hz), 128.2, 127.6, 127.4 (d, J = 8.1 Hz), 126.4, 125.7, 50.7 (d, J = 6.5 Hz), 35.5 (d, J = 9.5 Hz). **^{31}P NMR (162 MHz, CDCl$_3$)** δ 11.2. **HRMS-ESI (m/z):** calcd for $C_{22}H_{20}P^+[M+H]^+$ 315.1297, found 315.1300.

(*R*)-1-phenyl-2,3-dihydrophosphindole 1-oxide (6)

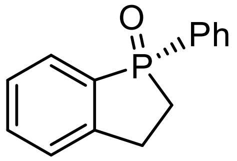

White solid, 26.1 mg at 0.20 mmol scale, 57% yield, >99% *ee*. **Optical rotation:** $[\alpha]^{25}_D$ +28.6 (c 0.52, CHCl$_3$). The enantiomeric excess was determined by chiral HPLC using CHIRALPAK IB N-5 column, *n*-Hexane/IPA = 88/12, flow rate 1.0 mL/min, uv-vis detection at λ = 210 nm, t_{major} = 13.21 min, t_{minor} = 14.57 min. **^{1}H NMR (400 MHz, CDCl$_3$)** δ 7.69 – 7.34 (m, 9H), 3.50 – 3.36 (m, 1H), 3.24 – 3.12 (m, 1H), 2.54 – 2.35 (m, 2H). **^{13}C NMR (101 MHz, CDCl$_3$)** δ 146.7 (d, J = 30.6 Hz), 132.6 (d, J = 50.3 Hz), 131.4, 131.9, 130.9 (d, J = 2.4 Hz), 129.6 (d, J = 10.4 Hz), 128.2 (d, J = 9.4 Hz), 127.7 (d, J = 12.1 Hz), 127.0 (d, J = 10.3 Hz), 125.5 (d, J = 11.2 Hz), 27.4 (d, J = 15.6 Hz), 27.1 (d, J = 59.4 Hz). **^{31}P NMR (162 MHz, CDCl$_3$)** δ 54.0. **HRMS-**

ESI (m/z): calcd for $C_{14}H_{14}OP^+$[M+H]$^+$ 229.0777, found 229.0779.

(1*R*,3*S*)-3-(diphenylphosphoryl)-1-phenyl-2,3-dihydrophosphindole 1-oxide (7)

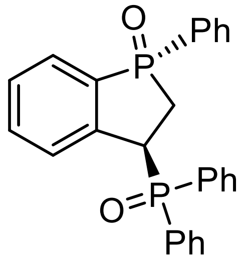

White solid, 38.9 mg at 0.10 mmol scale, 91% yield, >20:1 dr, >99% *ee*. **Optical rotation:** $[\alpha]^{25}_{D}$ +64.5 (c 0.33, $CHCl_3$). The enantiomeric excess was determined by chiral HPLC using CHIRALPAK IB N-5 column, *n*-Hexane/IPA = 80/20, flow rate 1.0 mL/min, uv-vis detection at λ = 210 nm, t_{major} = 14.88 min, t_{minor} = 11.28 min. **^{1}H NMR (400 MHz, $CDCl_3$)** δ 7.92 – 7.87 (m, 2H), 7.82 – 7.76 (m, 2H), 7.68 – 7.61 (m, 3H), 7.58 – 7.33 (m, 11H), 6.98 (s, 1H), 4.84 (q, *J* = 8.1 Hz, 1H), 2.72 – 2.43 (m, 2H). **^{13}C NMR (101 MHz, $CDCl_3$)** δ 143.4 (dd, *J* = 26.8, 4.7 Hz), 136.1 (d, *J* = 5.1 Hz), 135.0 (d, *J* = 4.9 Hz), 133.1, 132.7 (d, *J* = 2.4 Hz), 132.4, 132.3 (d, *J* = 4.2 Hz), 131.9 (d, *J* = 11.0 Hz), 131.7 (d, *J* = 9.0 Hz), 131.3 (d, *J* = 8.9 Hz), 131.0, 129.7 (d, *J* = 9.5 Hz), 129.3, 129.2, 129.1, 128.9, 128.6 (d, *J* = 12.6 Hz), 126.8 (dd, *J* = 11.4, 3.1 Hz), 40.9 (dd, *J* = 69.8, 2.6 Hz), 21.9 (d, *J* = 174.3 Hz). **^{31}P NMR (162 MHz, $CDCl_3$)** δ 50.1, 33.0. **HRMS-ESI (m/z):** calcd for $C_{26}H_{23}O_2P_2^+$[M+H]$^+$ 429.1168, found 429.1171.

2.5 DFT calculation

Geometries of transition states and intermediates were optimized by the DFT method with the PBE0 functional in combination with the RI approximation, the def2-TZVP basis set for rhodium atom and the def2-SVP basis set for other atoms, and the implicit solvent model C-PCM using THF as the solvent, applying the Grimme's D4 empirical correction as implemented in the ORCA 5.0.4 program package.[8-23] Frequency analyses were performed at the same level of theory as that used for geometry optimizations to characterize the stationary points as either minima (no imaginary frequencies) or saddle points (one imaginary frequency) on the potential energy surface. Intrinsic reaction coordinate (IRC) calculations were preformed to confirm that the transition state connects with its respective reactant and product. All geometrically optimized structures have been checked for the wavefunction stability. Grimme's quasi-harmonic approximation implemented in the ORCA 5.0.4 program package was applied to the vibrational entropies to obtain thermal corrections for Gibbs free energies.[24] The ZPE scale factor 0.98008286 was derived from linear regression to the experimental values available in the ZPVE15/10 Database.[25] Single point energies were calculated with the PBE0 functional in combination with the RI approximation and the def2-TZVPP basis set for all atoms, applying the Grimme's D4 empirical correction as implemented in the ORCA 5.0.4 program package.[15-23] The solvation energies were evaluated with the SMD(THF)/M06-2X/Lanl2TZ(Rh)/6-31G*(other atoms).[15-23] Free energies calculated using the gas phase standard state concentration (1 atm) were converted to reproduce the standard state concentration in solution (1 M) at 70 °C by adding 2.276098233 kcal/mol. The non-covalent interaction (NCI) analysis within the sign(λ_2)ρ colored reduced density gradient (RDG) plot were performed with Multiwfn 3.8(dev).[26] Molecular structures were visualized by using CYLView20.[27] The visualizations of IRC path was performed by Python package Matplotlib 3.5.2.[28]

Different transition states of olefin insertion with ligand were examined. The relative Gibbs energy of transition states (TS) of olefin insertion, enone binding (EB) intermediates, carborhodation (CR) intermediates are listed in **Supplementary Table**

1. Representative structures are shown in **Supplementary Figure 7**. The illustration diagram of IRC was shown in **Supplementary Figure 8**.

Supplementary Table 1. Relative Gibbs energy of TS, EB and CR (unit: kcal/mol)

#	Name	Abbr.	$\Delta G_{\mathrm{EB,gas}}$	ΔG_{EB}	$\Delta G_{\mathrm{TS,gas}}$	ΔG_{TS}	$\Delta G_{\mathrm{CR,gas}}$	ΔG_{CR}
1	LigDisf-SubDisf	TS_{SS}	10.39899220	5.607739008	20.30451652	16.83817836	-4.228746356	-8.492870810
2	LigDisf-SubFav	TS_{RS}	9.812829234	1.663479402	22.50506738	17.46298961	-2.061777579	-7.176225889
3	LigFav-SubDisf	TS_{RR}	5.412356138	2.605532240	16.54573311	14.37746271	-10.14577626	-12.62061948
4	LigFav-SubFav	TS_{SR}	0.000000000	0.000000000	12.47037038	11.98206841	-11.93527307	-12.66273943

Supplementary Figure 7. Representative structures of transition states

TS1

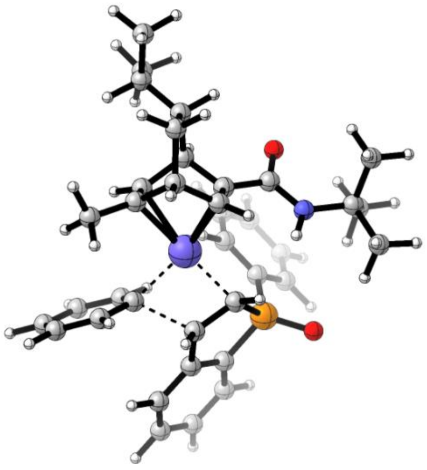

TS2

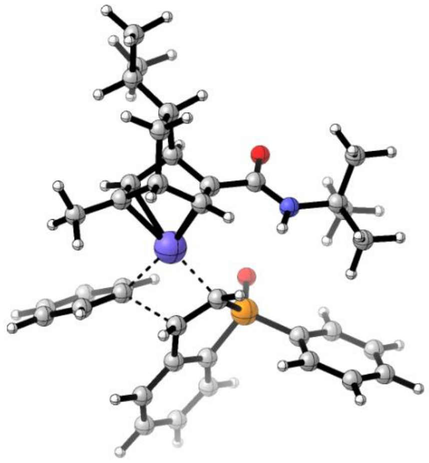

TS3

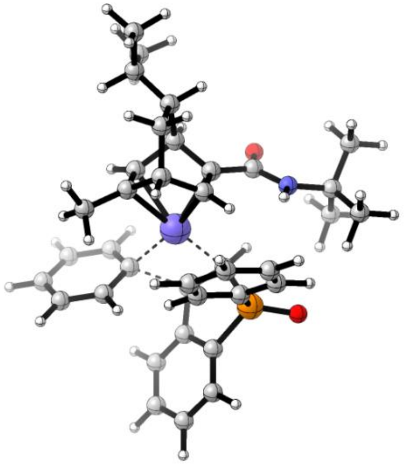

TS4

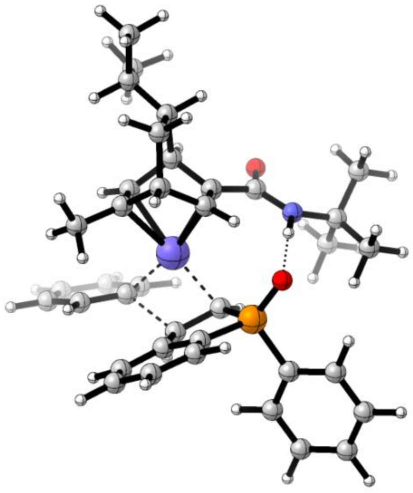

Supplementary Figure 8. The illustration diagram of IRC (for TS4)

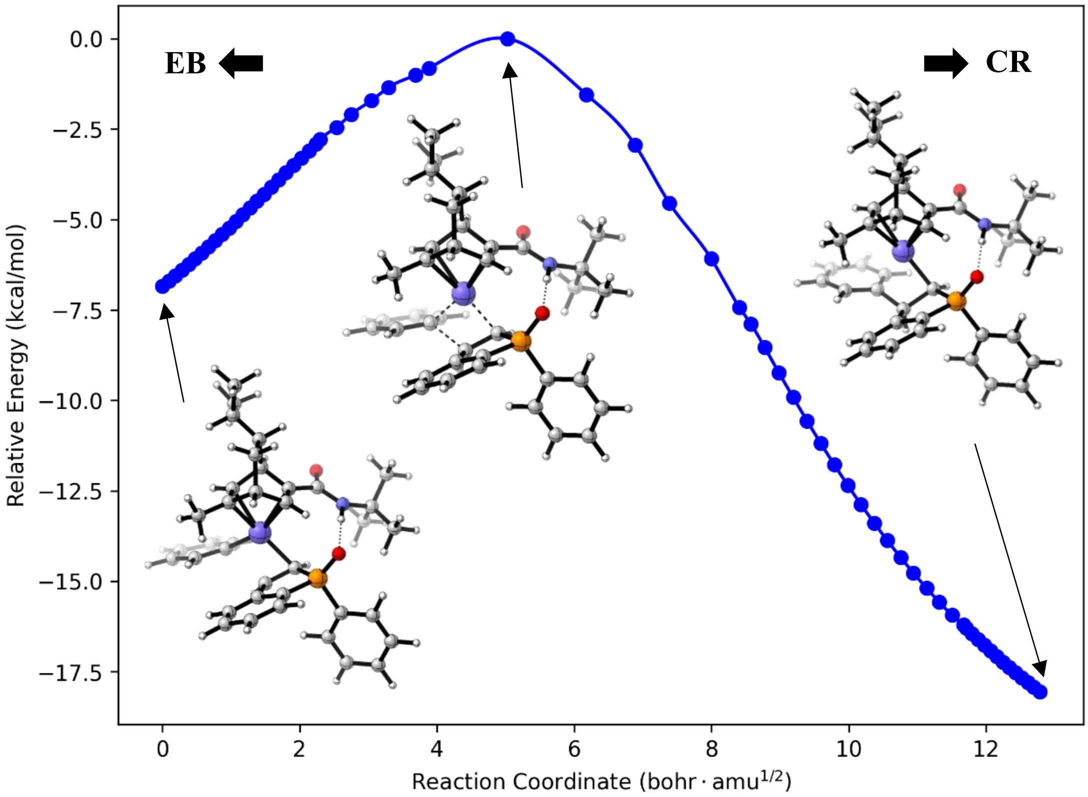

The absolute configuration of the product follows the Curtin–Hammett principle and the EB intermediates are in equilibrium. The relative energy difference between favored transition state and disfavored transition state determines the enantiomeric excess (ee) and diastereomeric ratio (dr), viz. $\mathrm{ee_{calc}} = \frac{\exp(-\Delta\Delta G_{TS}/RT)-1}{\exp(-\Delta\Delta G_{TS}/RT)+1}$, $\mathrm{dr_{calc}} = \exp(-\Delta\Delta G_{TS}/RT)$.

Supplementary Table 2. The calculated and experimental er and dr values (unit: kcal/mol)

Name	$\Delta\Delta G_{TS}$	Calculated	Experimental
ee (TS4 *vs*. TS2)	5.480921203	99.98%	>99%
dr (TS4 *vs*. TS3)	2.395394301	>98:2	>20:1
dr (TS4 *vs*. TS1)	4.856109955	>99:1	>20:1

The RDG plots of TS4 are given in **Supplementary Figure 9**, visualizing the non-covalent interactions in the transition states.[29] Only non-covalent interactions between ligand-Rh-Ph catalyst and olefin substrate are displayed (isovalue = 0.5). The hydrogen bonding interactions between the N-H of the ligand and the P=O of the substrate may

be important for the favorable $\Delta\Delta G_{\mathrm{TS}}$. The transition state without hydrogen bond was also identified (TS4'), whose relative energy ΔG_{TS} was 3.56 kcal/mol higher than that of TS4.

Supplementary Figure 9. The RDG plot of TS4 (left) and the structure of TS4' (right)

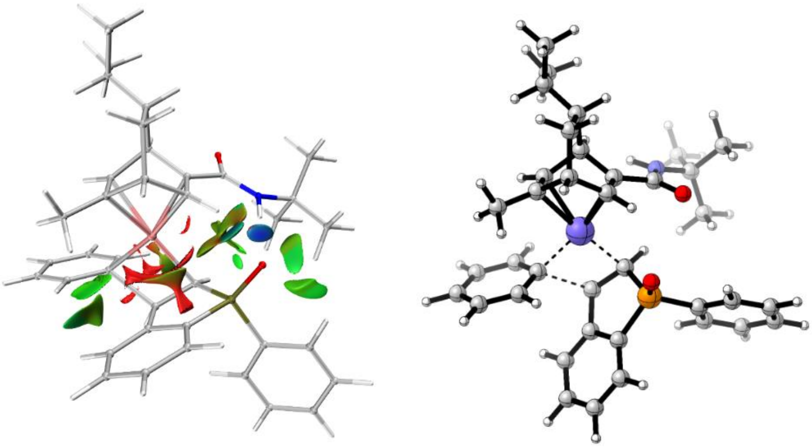

2.6 Biological activity study

Animals. The design, analysis and reporting of animal research were performed according to the COPE guidelines and ARRIVE guidelines. Animal experiments were approved by the Laboratory Animal Ethics Committee of Xuzhou Medical University (Xuzhou, China) (202309T012). Animals received humane care and were housed in a room maintained on a 12-hour light/dark cycle under constant temperature (22-25 °C).

Madin-Darby canine kidney (MDCK) cell colony formation assay. We applied a MDCK cell colony formation assay to screen their potential biological activities. In this model, MDCK cells were planted on 6-well plates and incubated with 10 μM **3aa**, **3ay**, **3az** or **1a** for 8 days, and the effects of these compounds on cell proliferation were evaluated by colony size and number. Encouragingly, the selected compounds **3aa, 3ay**, **3az** and **1a** inhibited cell colony formation to varying degrees, and **3az** displayed the better inhibitory efficacy than others (**Figure 5a**).

Pharmacological characterization of compounds 3az and *ent*-3az using an *in vitro* MDCK cyst model.[30] Next, a MDCK cyst model was used for fast evaluating the pharmacological effect of the synthesized **3az** and ***ent*-3az**. In this model, MDCK cells were cultured in three-dimensional collagen gel and treated with 10 μM forskolin. Fluid-filled cysts formed and progressively expanded. By exposing established cysts (> 50 μm diameter on Day 4) to the compounds **3az** and ***ent*-3az** at 10 μM for six consecutive days, compound **3az** showed a significantly higher inhibitory effect on cystogenesis compared to compound ***ent*-3az** (**Supplementary Figure 10**). It follows from **Supplementary Figure 10** that compound **3az** significantly retarded cyst growth from Day 6 and onwards at 10 μM. Such effect was concentration-dependent with 1 μM compound **3az** showing less inhibitory effect.

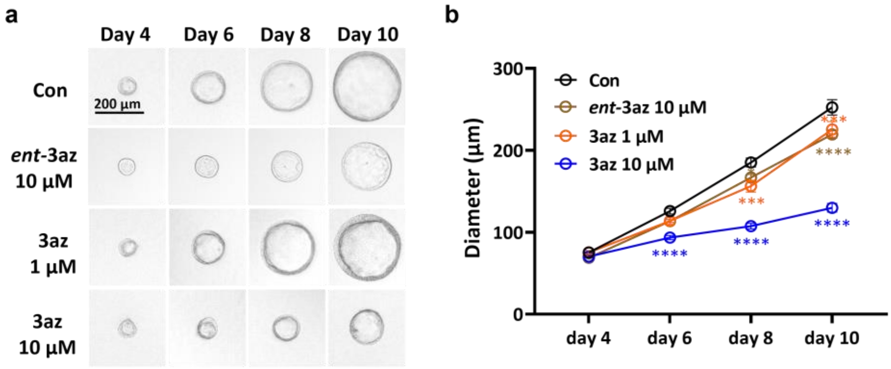

Supplementary Figure 10. **Pharmacological characterization of compounds *ent*-3az and 3az using an *in vitro* MDCK cyst model.** a) Representative images of MDCK cysts from day 4 to day 10 without (Con) or with exposure to compound *ent*-3az at 10 μM or compound 3az at 1 and 10 μM. b) Cyst diameters of Con, *ent*-3az 10 μM, 3az 1 μM and 3az 10 μM from day 4 to day 10. $n = 3$. Day 6, $p < 0.0001$, Con vs 3az 10 μM. Day 8, $p = 0.0407$, Con vs *ent*-3az 10 μM; $p = 0.0002$, Con vs 3az 1 μM; $p < 0.0001$, Con vs 3az 10 μM. Day 10, $p < 0.0001$, Con vs *ent*-3az 10 μM; $p = 0.0008$, Con vs 3az 1 μM; $p < 0.0001$, Con vs 3az 10 μM. Data are presented as mean ± S.E.M., $*p < 0.05$, $***p < 0.001$, $****p < 0.0001$, two-way ANOVA followed by Dunnett's post-test. Source data are provided as a Source Data file.

Pharmacological characterization of compounds 3az and *ent*-3az using an *ex vivo* embryonic kidney cyst model.[31] Subsequently, the inhibitory effect of compounds **3az** and ***ent*-3az** on renal cyst development was further explored using an *ex vivo* embryonic kidney cyst model. In this model, embryonic kidneys obtained from wild-type C57BL/6 mice on embryonic day 13.5 were cultured in transwell filters. Induced with 100 μM 8-bromoadenosine 3′,5′-cyclic monophosphate (8-Br-cAMP), renal cysts developed and swiftly expanded throughout the entire kidneys. It follows from **Supplementary Figure 11** that compound **3az** was significantly higher inhibitory effect in suppressing renal cyst development in comparison with the ***ent*-3az**-treated group.

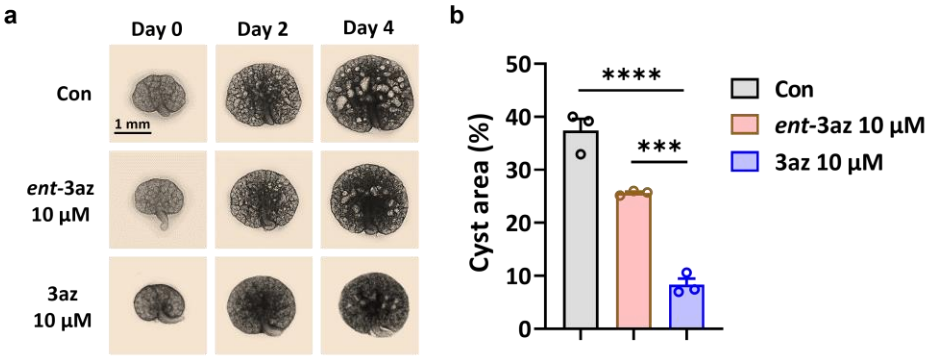

Supplementary Figure 11. Pharmacological characterization of compounds *ent*-3az and 3az using an *ex vivo* embryonic kidney cyst model. a) Representative light micrographs of embryonic kidneys treated without (Con) or with 10 μM compound *ent*-3az or 10 μM compound 3az from day 0 to day 4. b) Fractional cyst area of kidneys treated without (Con) or with 10 μM compound *ent*-3az and compound 3az on day 4. $n = 3$. $p < 0.0001$, Con vs 3az 10 μM; $p = 0.0004$, *ent*-3az 10 μM vs 3az 10 μM. Data are presented as mean ± S.E.M., ***$p < 0.001$, ****$p < 0.0001$, one-way ANOVA followed by Dunnett's post-test. Source data are provided as a Source Data file.

Pharmacological characterization of compounds 3az and *ent*-3az using a kidney-specific *Pkd1* knockout mouse model.[30,31] The above results demonstrated that compounds **3az** and ***ent*-3az** can inhibit renal cysts growth. Compounds **3az** and ***ent*-3az** were progressed for *in vivo* evaluation using kidney-specific *Pkd1* knockout ADPKD mouse model ($Pkd1^{flox/flox}$;*Ksp-Cre* mice). *Pkd1* was specifically knocked out in the kidney to induce the rapid development of renal cysts in the neonatal period and lead to rapidly progressive kidney failure and eventually death by approximately postnatal day 20. Compared with vehicle-treated and ***ent*-3az**-treated ADPKD mice, compound **3az** led to a significant reduction in relative kidney size of the ADPKD mice. Further quantification of the KW/BW ratio (total kidney weight to body weight) of each group revealed that compound **3az** significantly reduced this ratio to 15.05 ± 0.56 % in ADPKD mice compared to 20.80 ± 0.53% in the vehicle-treated ADPKD mice. Hematoxylin-eosin staining of the renal tissues demonstrated that compound **3az** treatment significantly reduced the fractional cyst area in ADPKD kidneys (**Figure 5d**). These data demonstrated that compound **3az** delayed renal cyst growth *in vivo*.

On the contrast, ***ent*-3az** can effectively decrease the KW/BW ratio to 17.18 ± 0.33% in ADPKD mice compared to 21.02 ± 0.66% in the vehicle-treated ADPKD mice, while the inhibitory effect of renal cysts formation was not detected obviously in

hematoxylin-eosin staining (**Supplementary Figure 12**).

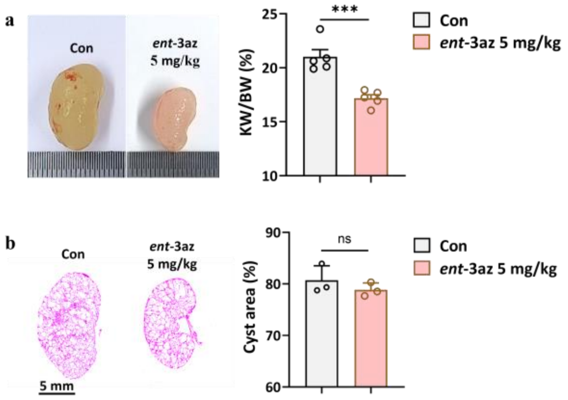

Supplementary Figure 12. **Pharmacological characterization of compounds *ent*-3az using a kidney-specific *Pkd1* knockout mouse model.** a) Representative images of kidneys and KW/BW value (total kidney weight/body weight) from ADPKD mice treated without (Con) or with 5 mg/kg compound *ent*-3az for 7 days, $n = 5$. b) Hematoxylin and eosin staining of kidney sections and fractional cyst area of kidneys from the indicated groups, $n = 3$. For KW/BW, $p = 0.0008$, Con vs *ent*-3az. For cyst area, $p = 0.3636$, Con vs *ent*-3az. Data are presented as mean ± S.E.M., ***$p < 0.001$, ns (no significance), two-sided student's t test. Source data are provided as a Source Data file.

2.7 Proteomic analysis

Protein extraction and tryptic digestion. Tissues were lysed in lysis buffer (RPIA) supplemented with protease inhibitors and phosphatase inhibitors for 30 min. Then they were sonicated by grinding instrument at 4 ^{o}C and centrifuged at 13800 g for 15 min to remove the tissue debris. The protein in supernatants were collected and measured by using BCA protein assay. Next, 20 mM Tris(2-chloroethyl) phosphate (TCEP) was added to the protein solution to react at 60 ^{o}C for 1 h. After that, 200 mM Chloroacetamide (CAA) was added to the mixture and react at room temperature for 30 min in darkness. After the reaction, ice-cold acetone was used to precipitate the protein at -20 ^{o}C for 4 h. The pellet was air-dried and then resuspended in 150 μl 0.1M ABB. Protein samples underwent trypsin digestion (enzyme-to-substrate ratio of 1:25 at 37 ^{o}C for 16 hours) followed by desalting through sola HRP cartridges and vacuum-dried by Speed Vac.

Nano-LC-MS/MS analysis. Peptide samples were analyzed by an EASY-nLC 1200 LC system coupled with Orbitrap mass spectrometry (Thermo Fisher). The column was C18 nano-capillary analytical column (25 cm×75 mm, 1.9 μm). Peptides were re-dissolved in mobile phase A (Water/CAN/formic acid, 98/2/0.1, *v/v*). The gradient was 0-5 min, 5-10% B; 5-55 min, 10-28% B; 55-58 min, 28-40% B，58-63 min, 40-95% B; 63-75 min; 95% B with flow rate of 350 nL/min. Mass spectrometry was operated under a data independent acquisition mode (DIA). The *m/z* range was 350 to 1500 Da in MS1 with the resolution of 60,000. The automatic gain control (ACG) was set as 3e6. The maximal ion injection time was 50 ms. MS2 acquisition was higher energy collision dissociation (HCD) with the collision energy of 30 eV. The resolution was 30,000, with loop count set to: 25, MSX count set to: 1, isolation window set to: 8.3m/z, fixed maximum mass set to: 200m/z, respectively.

MS database search. DIA-NN was used for the MS database search by predict mouse source spectrum library for comparison. Other parameters are set as follows: the number of missed cleveges was 3, the Peptide length range was 5-100, and the Precursor charge range was 1-7, the Precursor m/z range was 250-2000, and the

Fragment ion m/z range was 300-2000.

Bioinformatics analysis. The differentially expressed proteins were annotated from the functional information of molecular functions (MF), biological process BP (BP) and cellular components (CC). The differentially expressed proteins were then compared with the KEGG database to classify the selected differentially expressed genes and obtain the pathway information of protein sequence participation.

Proteomic analysis of downstream targets regulated by Compound 3az. we performed a proteomic analysis of kidneys samples from mice with ADPKD and **3az**-treated mice with ADPKD (**Supplementary Figure 13a**) to explore the mechanisms of **3az** treatment in ADPKD. Over 5000 proteins were obtained in kidney tissues from ADPKD group and drug administration group. Among them, 112 and 135 proteins were found up- and down-regulation between two groups, respectively (**Supplementary Figure 13b** and **13c**). Gene Ontology (GO) analysis of these dysregulated proteins preliminarily revealed that the proteins regulated by **3az** were mainly enriched in many biological processes. In particular, microtubule-based processes, microtubule cytoskeleton organization and mitotic cell cycle changed most significantly and were enriched in a great many of dysregulated proteins following **3az** treatment in ADPKD mice (**Supplementary Figure 13d**). GSEA enrichment analysis demonstrated that compound **3az** can down-regulate the proliferation ability of kidney in ADPKD mice (**Supplementary Figure 13e**). Microtubule-based processes and microtubule cytoskeleton formation have been reported to be associated with retard of cell proliferation and renal cyst expansion.[32]

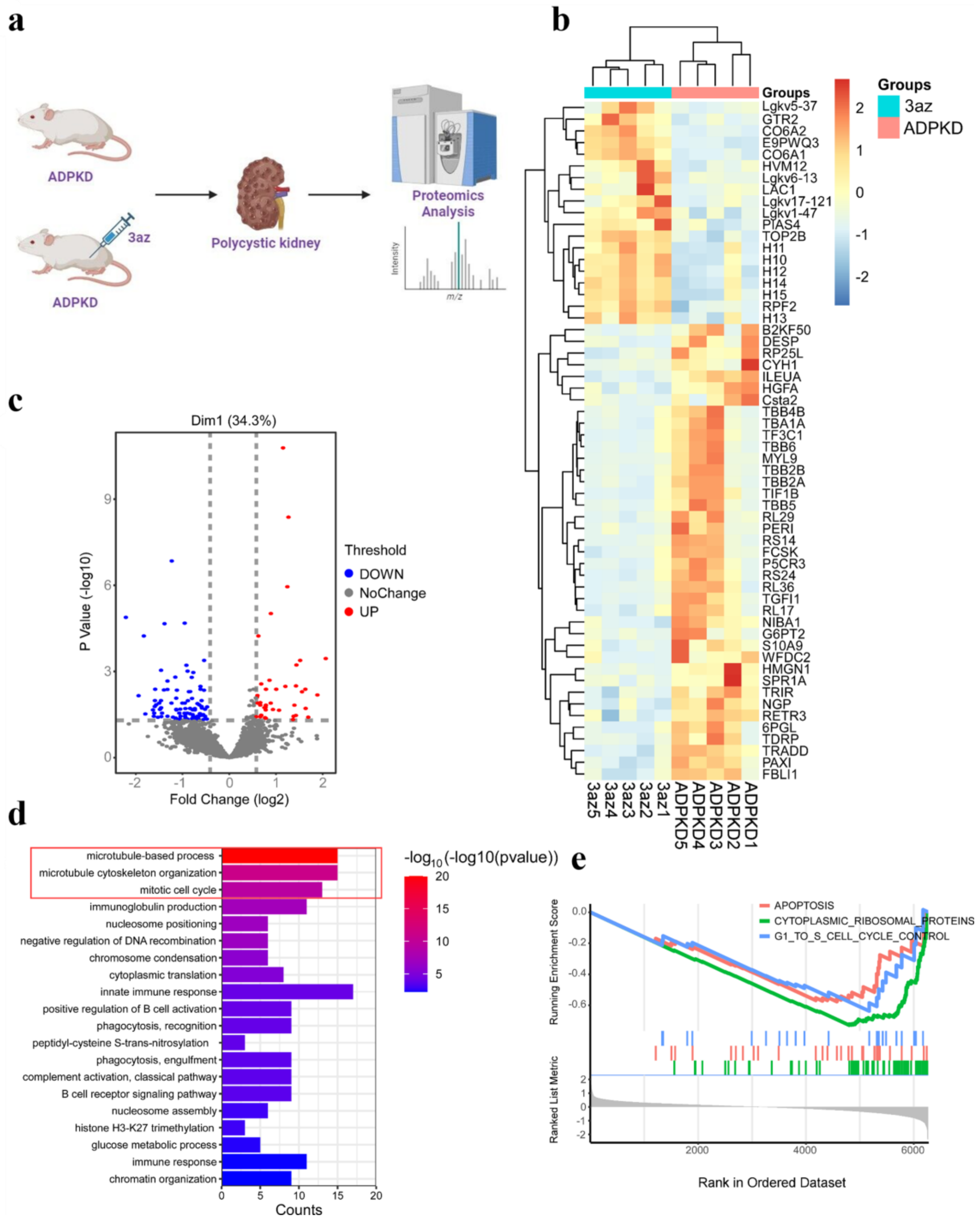

Supplementary Figure 13. Proteomic analysis. **a** The proteomic analysis of kidney tissues in ADPKD mice (Image created with BioRender.com. agreement number: LU26HODQYI). **b** The volcano plot. **c** Heatmap. **d** GO pathway analysis. **e** GSEA analysis of proteins between ADPKD group and drug administration group.

2.8 Molecular docking

Compound **3az** was constructed by Sybyle 6.9 and prepared by Autodock Tools 1.5.6.[33] Then, the optimized **3az** was docked into tubulin (PDB ID: 6EW0) by Autodock (version 4.2.6) software. The location of the paclitaxel was defined as the ligand binding site. The Lamarkian genetic algorithm was used to search for conformation. During the docking phase, ligands were thought to be flexible and the protein was considered to be rigid.

The docking result showed that the binding of **3az** is more stable than that of 1-indanone reported by Li *et al*.[32] 1-Indanone only forms hydrogen bonds with R359 of tubulin, while **3az** also forms π-π stacking interactions with tubulin in addition to this hydrogen bond (**Supplementary Figure 14**).

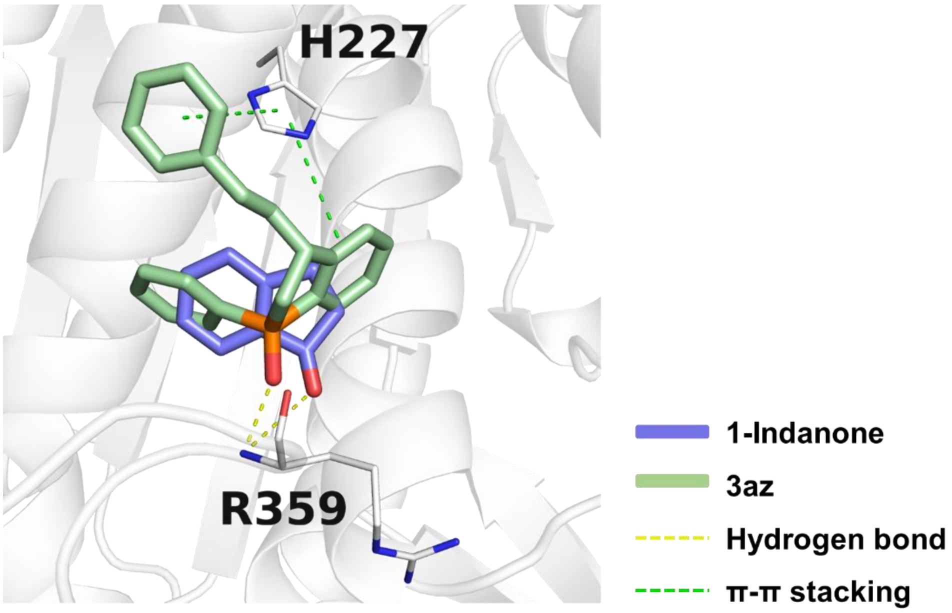

Supplementary Figure 14. Molecular docking. Virtual docking results showing **3az** formed a hydrogen bond with R359 and π-π stacking interactions with H227 in the paclitaxel binding site of tubulin (PDB ID: 6EW0)

2.9 Single crystal X-ray diffraction

Single crystal of **3aa** for X-ray diffraction was grown by liquid-liquid diffusion in a n-hexane/DCM binary solvent system in a 10 mL screw cap vial at room temperature. The X-ray data of **3aa** is deposited in the Cambridge Crystallographic Data Centre with a number of CCDC 2246799.

Supplementary Figure 15. ORTEP representation of the X-ray structure of enantiopure 3aa

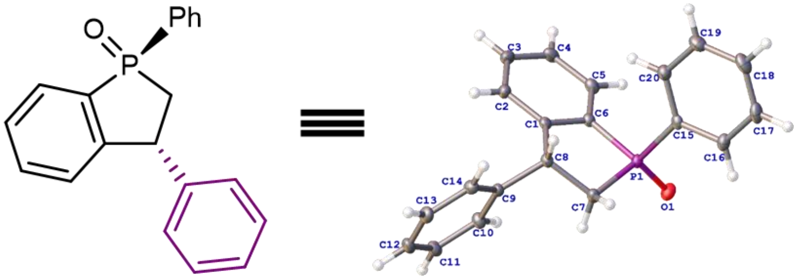

(thermal ellipsoids are drawn at 50% probability)

Supplementary Table 3. Crystal data and structure refinement for 3aa.

Identification code	02-1
Empirical formula	$C_{20}H_{17}OP$
Formula weight	304.30
Temperature/K	169.99(10)
Crystal system	orthorhombic
Space group	$P2_12_12_1$
a/Å	8.21190(10)
b/Å	11.54650(10)
c/Å	16.6999(2)
α/°	90
β/°	90
γ/°	90
Volume/$Å^3$	1583.46(3)
Z	4
$\rho_{calc}g/cm^3$	1.276
μ/mm^{-1}	1.514
F(000)	640.0
Crystal size/mm^3	0.15 × 0.13 × 0.08
Radiation	Cu Kα (λ = 1.54184)
2Θ range for data collection/°	9.312 to 147.474
Index ranges	$-9 \leq h \leq 10$, $-14 \leq k \leq 14$, $-20 \leq l \leq 20$
Reflections collected	17482
Independent reflections	3185 [R_{int} = 0.0423, R_{sigma} = 0.0214]
Data/restraints/parameters	3185/0/200
Goodness-of-fit on F^2	1.046
Final R indexes [I>=2σ (I)]	R_1 = 0.0254, wR_2 = 0.0686
Final R indexes [all data]	R_1 = 0.0257, wR_2 = 0.0690
Largest diff. peak/hole / e $Å^{-3}$	0.20/-0.18

Flack/Hooft parameter	0.024(8)/0.015(6)

Single crystal of **(*R*)-1a** for X-ray diffraction was grown by liquid-liquid diffusion in a n-hexane/DCM binary solvent system in a 10 mL screw cap vial at room temperature. The X-ray data of **(*R*)-1a** is deposited in the Cambridge Crystallographic Data Centre with a number of CCDC 2246797.

Supplementary Figure 16. ORTEP representation of the X-ray structure of enantiopure (*R*)-1a

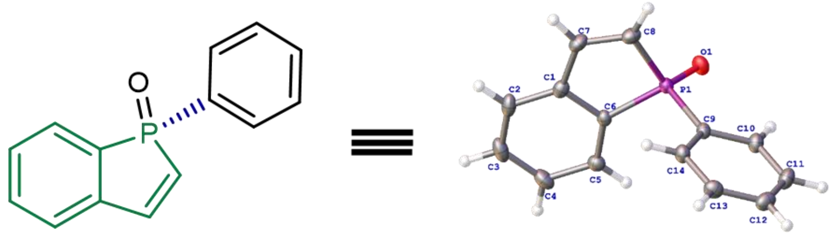

(thermal ellipsoids are drawn at 50% probability)

Supplementary Table 4. Crystal data and structure refinement for (*R*)-1a.

Identification code	01-0302
Empirical formula	$C_{14}H_{11}OP$
Formula weight	226.20
Temperature/K	169.99(10)
Crystal system	hexagonal
Space group	$P6_5$
a/Å	9.5910(2)
b/Å	9.5910(2)
c/Å	21.7683(5)
α/°	90
β/°	90
γ/°	120
Volume/$Å^3$	1734.13(8)
Z	6
$\rho_{calc}g/cm^3$	1.300
μ/mm^{-1}	1.886
F(000)	708.0
Crystal size/mm^3	0.15 × 0.13 × 0.08
Radiation	Cu Kα (λ = 1.54184)
2Θ range for data collection/°	10.652 to 146.856
Index ranges	$-11 \le h \le 11$, $-11 \le k \le 10$, $-23 \le l \le 26$
Reflections collected	6354
Independent reflections	2069 [R_{int} = 0.0315, R_{sigma} = 0.0324]

Data/restraints/parameters	2069/1/146
Goodness-of-fit on F^2	1.040
Final R indexes [I>=2σ (I)]	R_1 = 0.0284, wR_2 = 0.0712
Final R indexes [all data]	R_1 = 0.0301, wR_2 = 0.0728
Largest diff. peak/hole / e $Å^{-3}$	0.15/-0.16
Flack/Hooft parameter	0.015(14)/0.022(14)

Single crystal of **3ak** for X-ray diffraction was grown by liquid -liquid diffusion in a n-hexane/DCM binary solvent system in a 10 mL screw cap vial at room temperature. The X-ray data of **3ak** is deposited in the Cambridge Crystallographic Data Centre with a number of CCDC 2246811.

Supplementary Figure 17. ORTEP representation of the X-ray structure of enantiopure 3ak

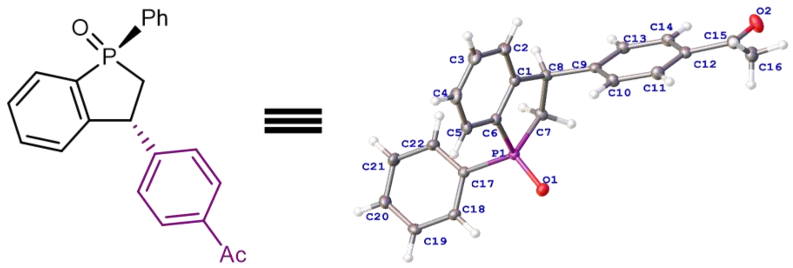

Supplementary Table 5. Crystal data and structure refinement for 3ak.

Identification code	03_2
Empirical formula	$C_{22}H_{19}O_2P$
Formula weight	346.34
Temperature/K	169.99(10)
Crystal system	monoclinic
Space group	$P2_1$
a/Å	7.74140(10)
b/Å	5.83010(10)
c/Å	19.1986(3)
α/°	90
β/°	96.6650(10)
γ/°	90
Volume/$Å^3$	860.64(2)
Z	2
$\rho_{calc}g/cm^3$	1.336
μ/mm^{-1}	1.505
F(000)	364.0
Crystal size/mm^3	0.15 × 0.13 × 0.08

Radiation	Cu Kα (λ = 1.54184)
2Θ range for data collection/°	4.634 to 147.774
Index ranges	$-9 \leq h \leq 9$, $-6 \leq k \leq 6$, $-22 \leq l \leq 23$
Reflections collected	6727
Independent reflections	3221 [R_{int} = 0.0247, R_{sigma} = 0.0284]
Data/restraints/parameters	3221/1/228
Goodness-of-fit on F^2	1.047
Final R indexes [I>=2σ (I)]	R_1 = 0.0267, wR_2 = 0.0722
Final R indexes [all data]	R_1 = 0.0268, wR_2 = 0.0723
Largest diff. peak/hole / e $Å^{-3}$	0.24/-0.16
Flack/Hooft parameter	0.008(10)/0.009(8)

3. Supplementary Figures

3.1 NMR spectra

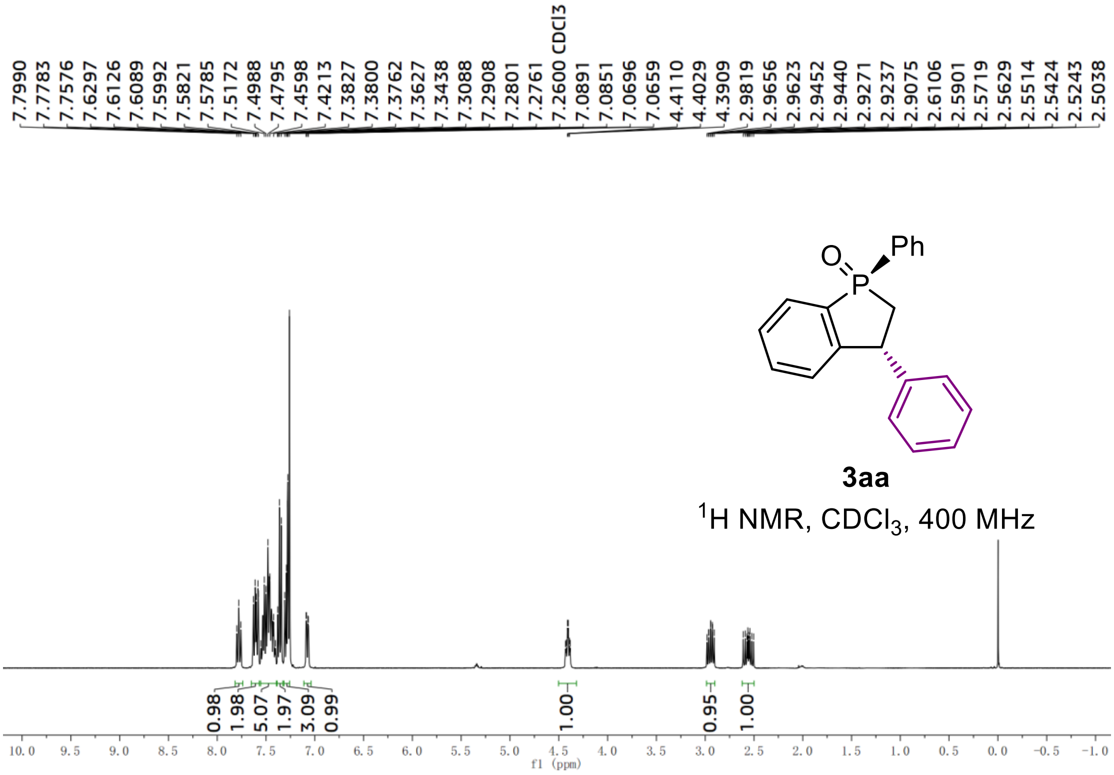

Supplementary Figure 18. ^{1}H NMR of the 3aa (400 MHz, $CDCl_3$)

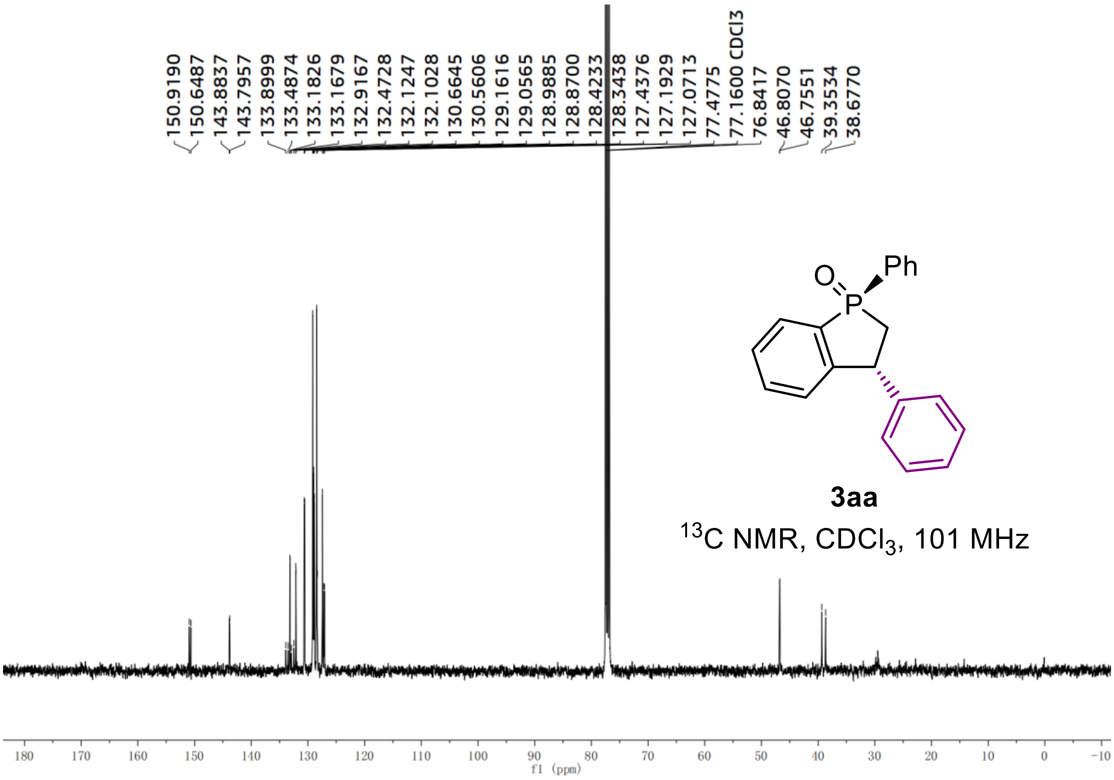

Supplementary Figure 19. ^{13}C NMR of the 3aa (101 MHz, $CDCl_3$)

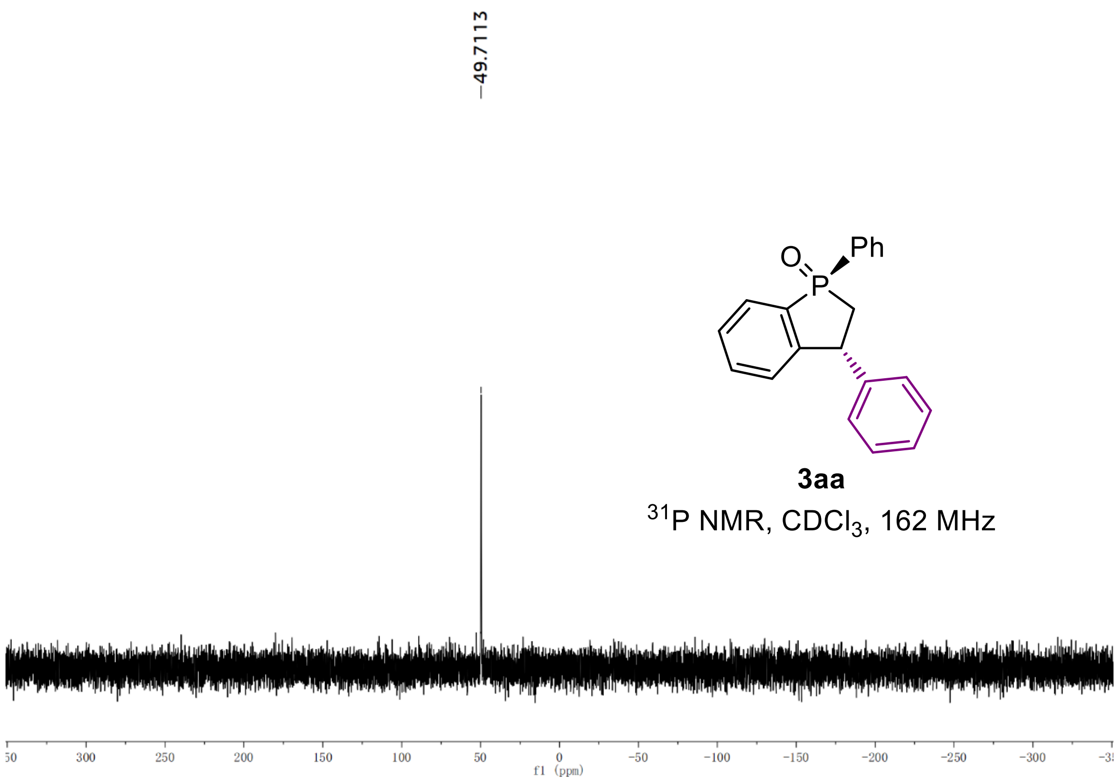

Supplementary Figure 20. ^{31}P NMR of the 3aa (162 MHz, $CDCl_3$)

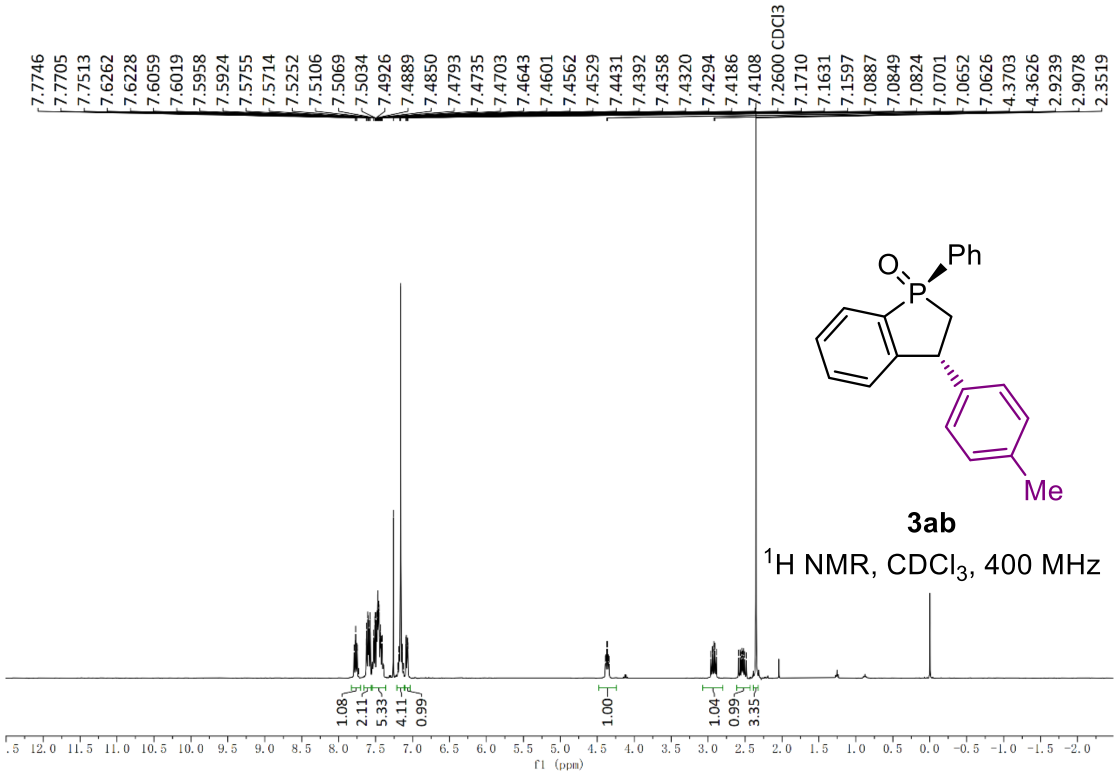

Supplementary Figure 21. ^{1}H NMR of the 3ab (400 MHz, $CDCl_3$)

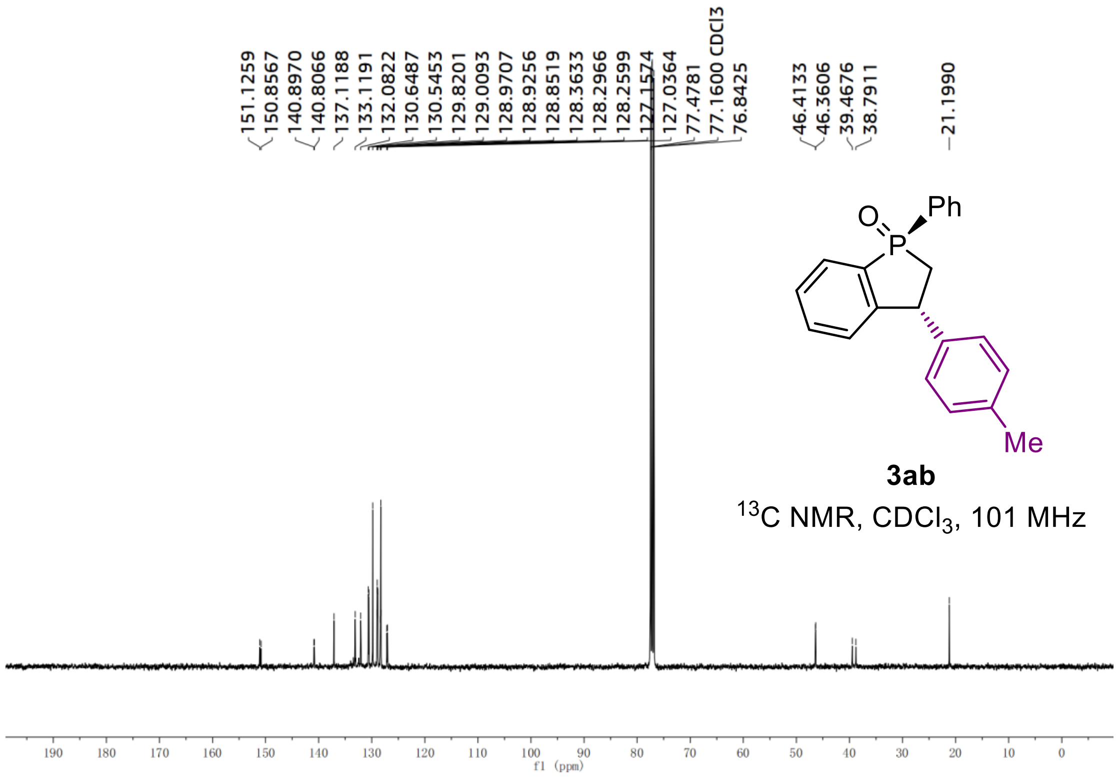

Supplementary Figure 22. ^{13}C NMR of the 3ab (101 MHz, $CDCl_3$)

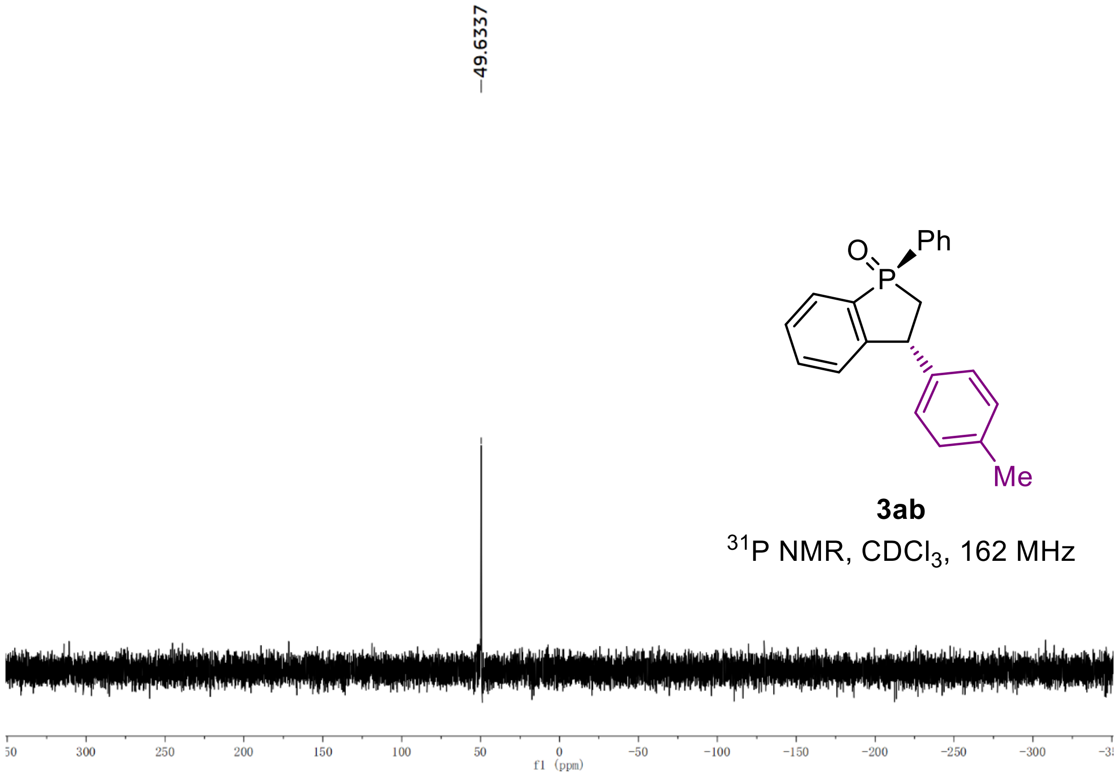

Supplementary Figure 23. ^{31}P NMR of the 3ab (162 MHz, $CDCl_3$)

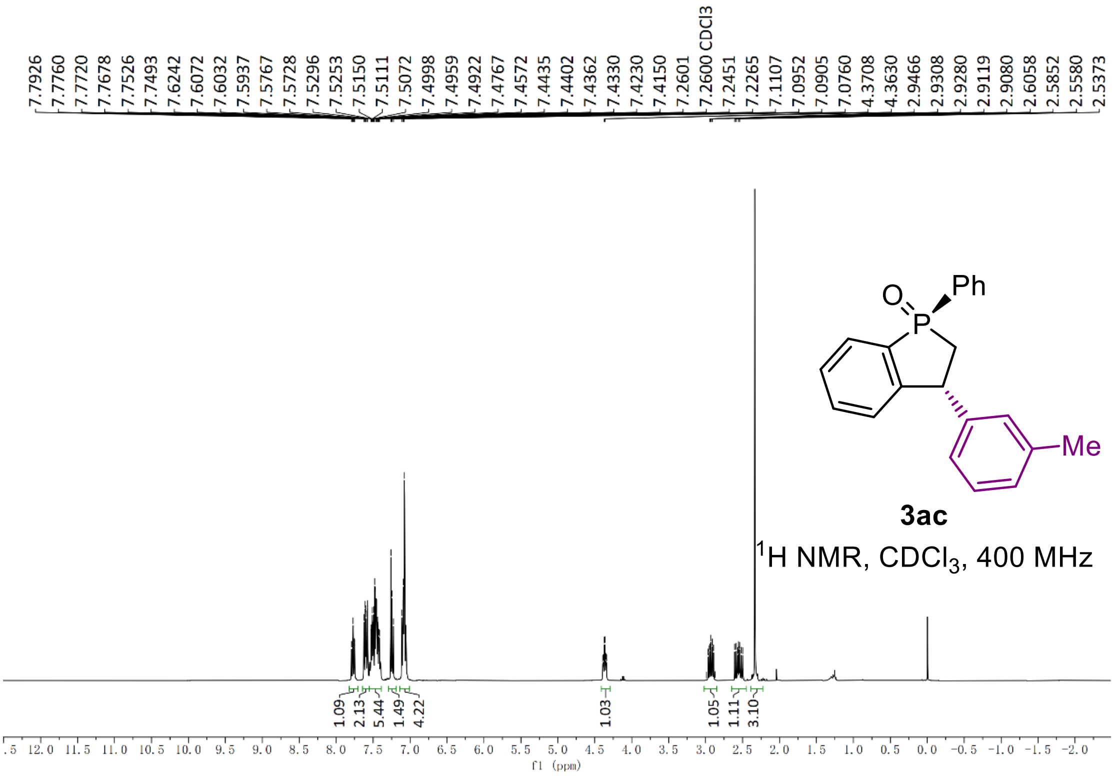

Supplementary Figure 24. ^{1}H NMR of the 3ac (400 MHz, $CDCl_3$)

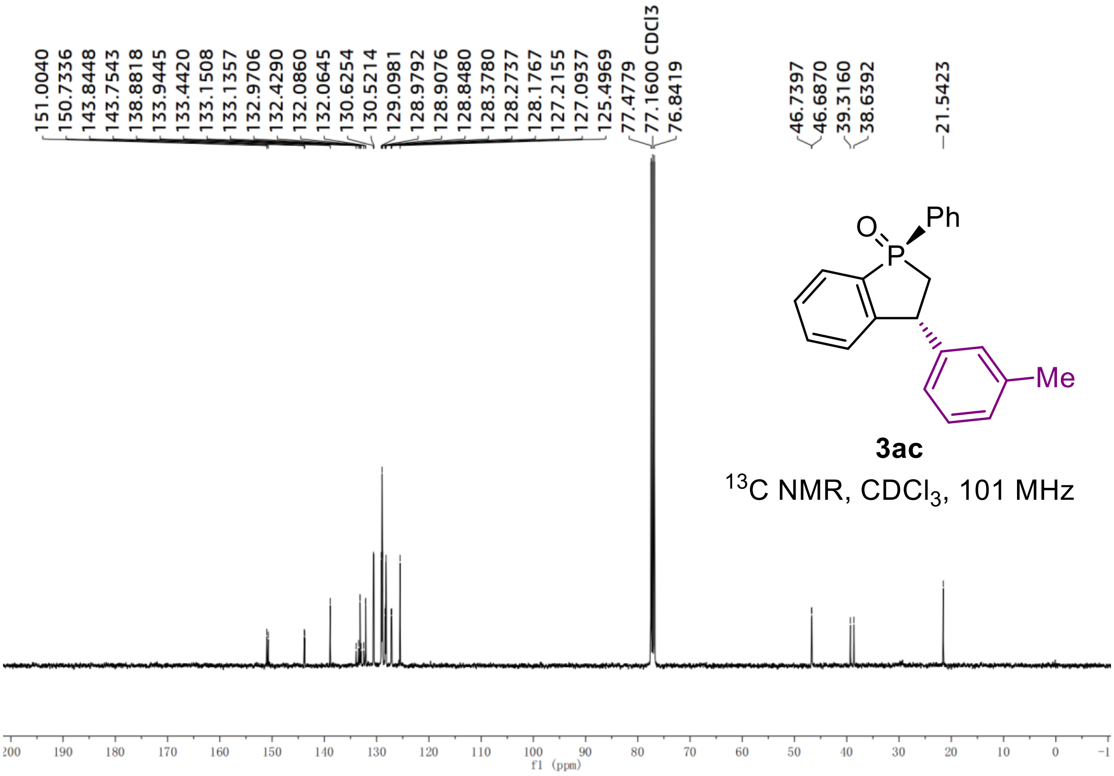

Supplementary Figure 25. ^{13}C NMR of the 3ac (101 MHz, $CDCl_3$)

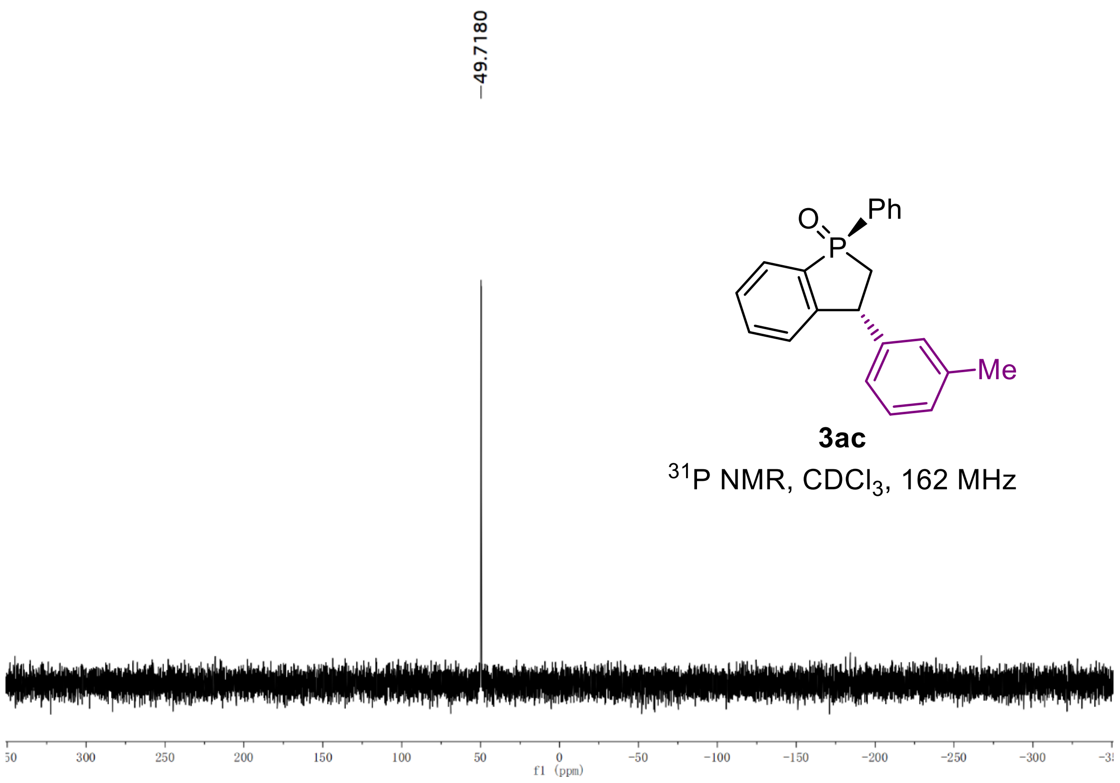

Supplementary Figure 26. ^{31}P NMR of the 3ac (162 MHz, CDCl$_3$)

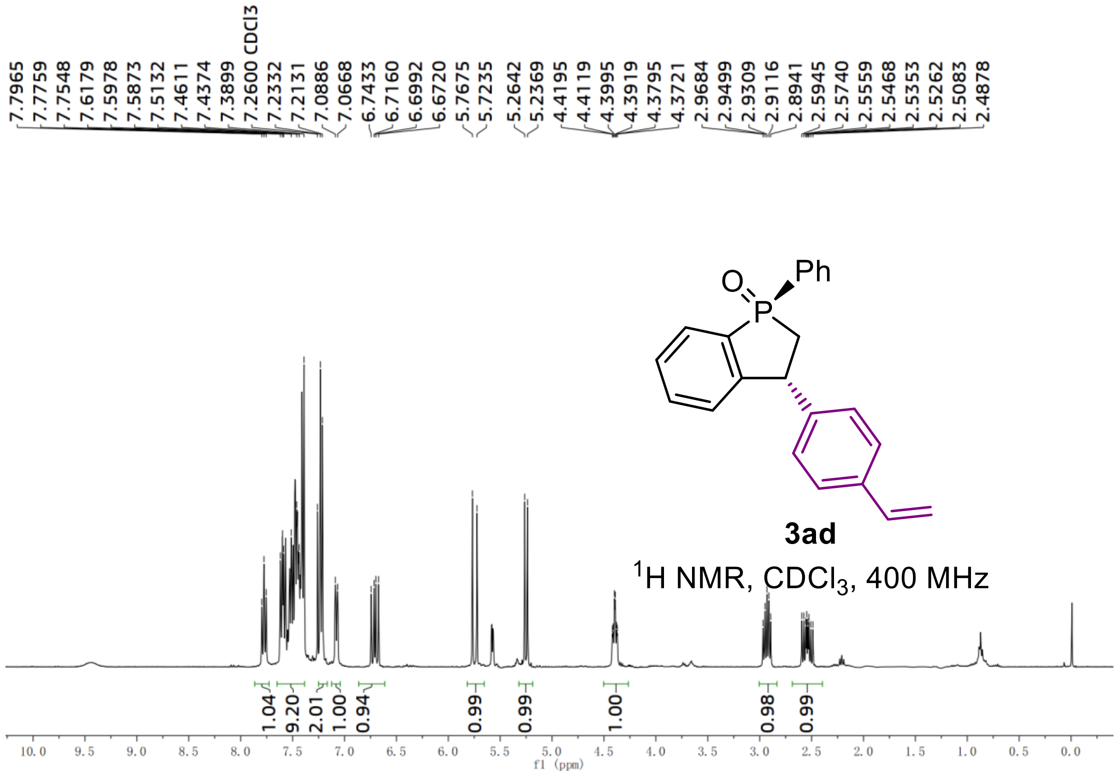

Supplementary Figure 27. ^{1}H NMR of the 3ad (400 MHz, CDCl$_3$)

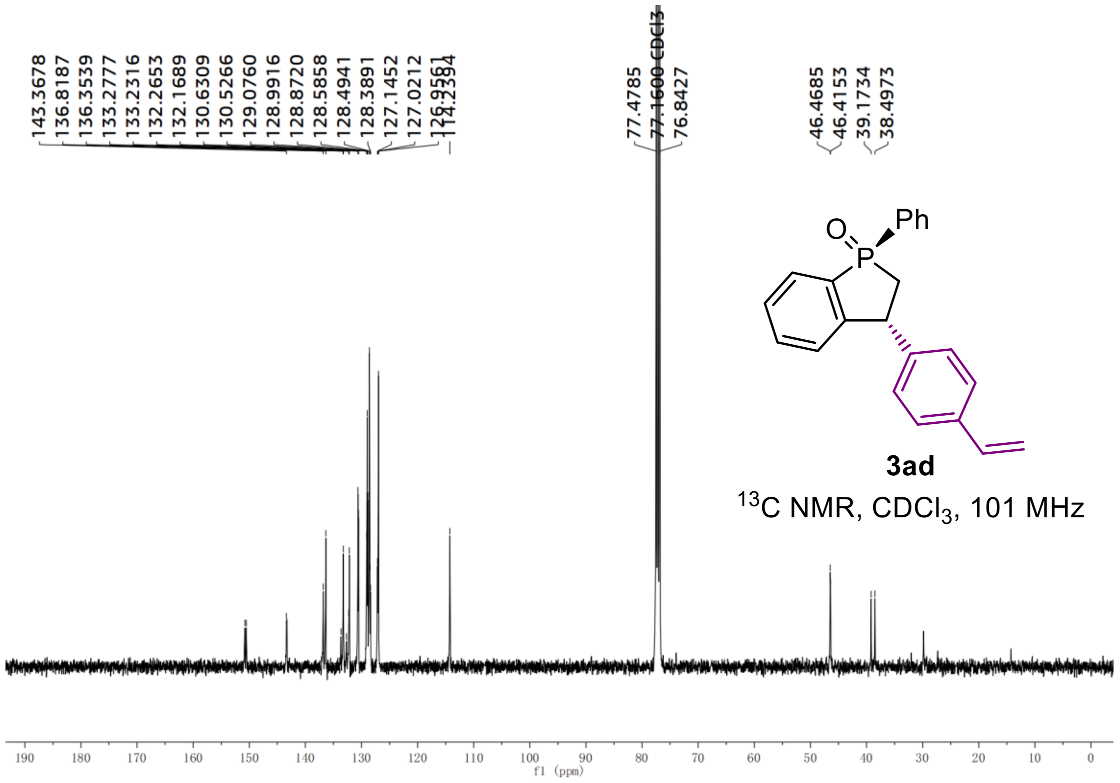

Supplementary Figure 28. ^{13}**C NMR of the 3ad (101 MHz, CDCl$_3$)**

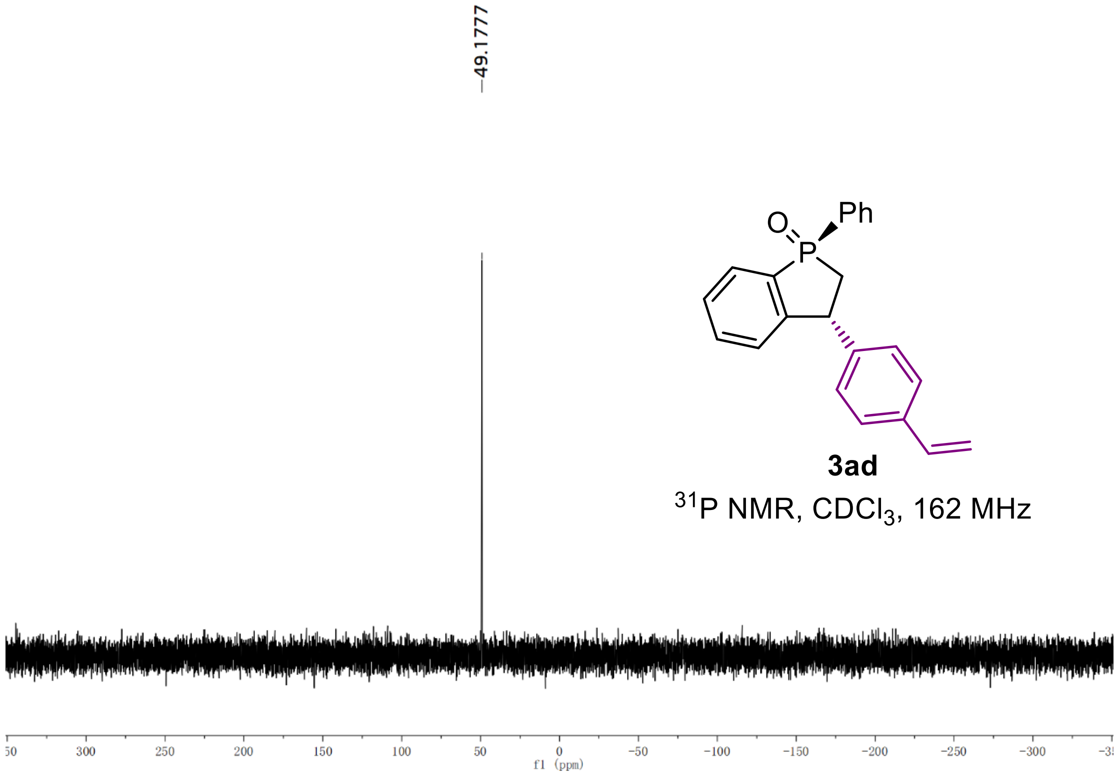

Supplementary Figure 29. ^{31}**P NMR of the 3ad (162 MHz, CDCl$_3$)**

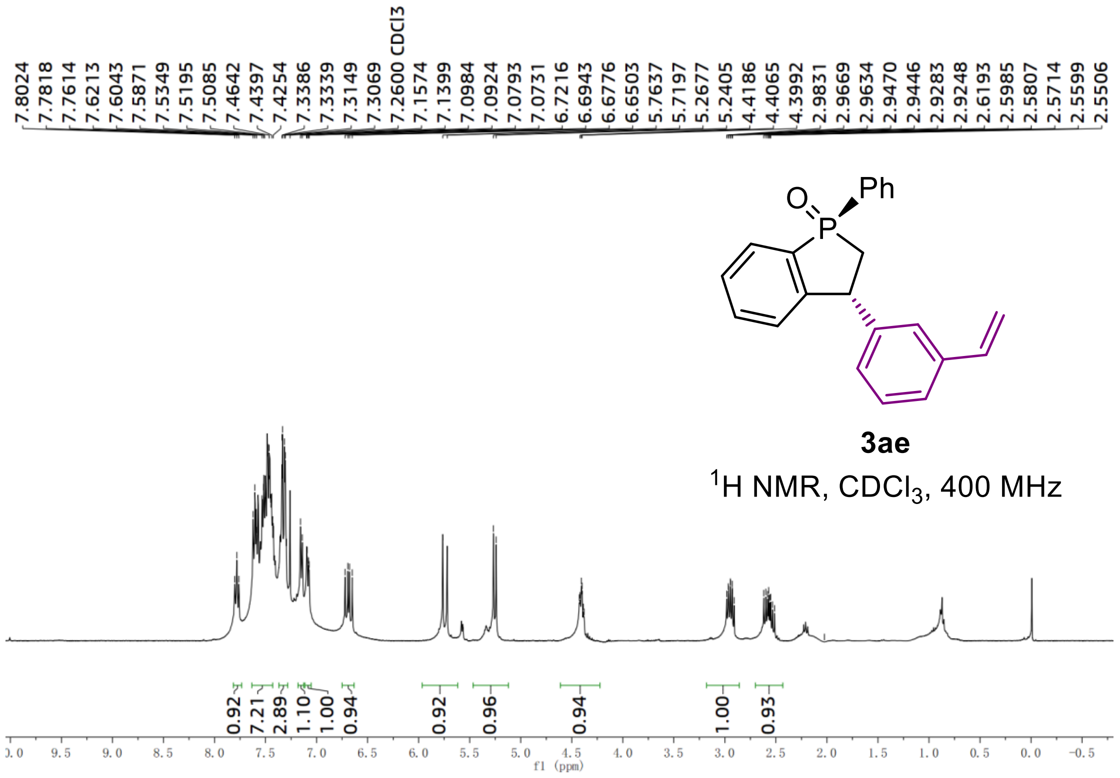

Supplementary Figure 30. ^{1}H NMR of the 3ae (400 MHz, $CDCl_3$)

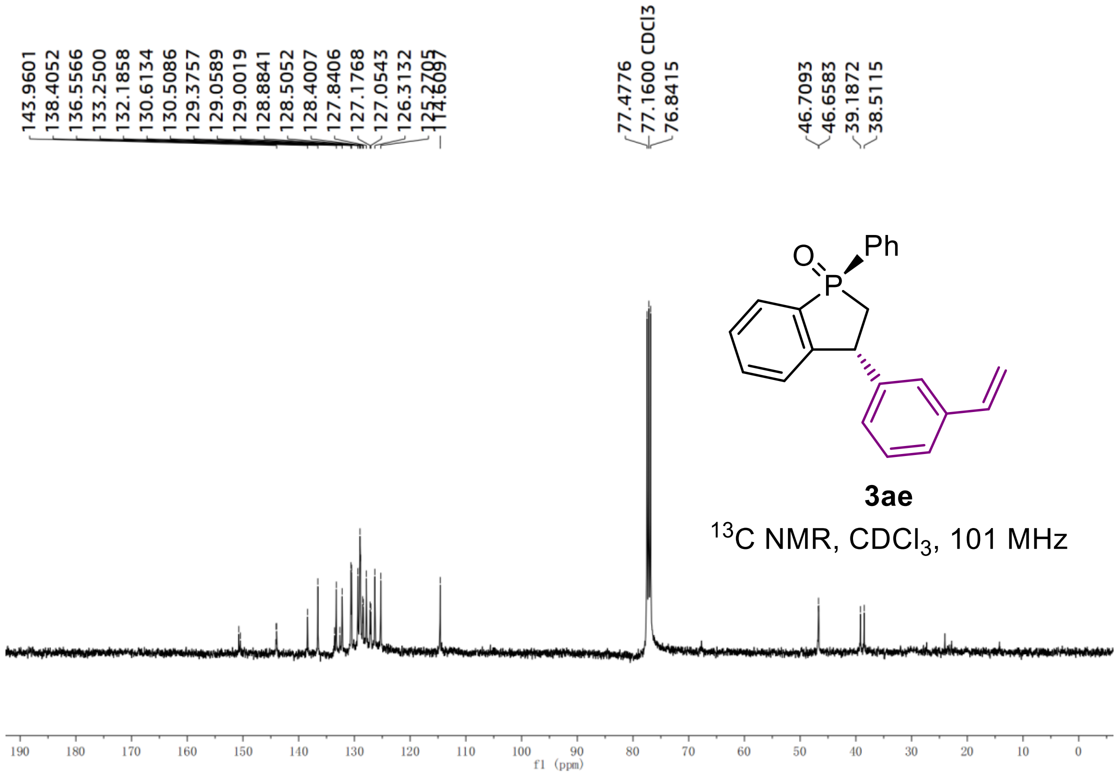

Supplementary Figure 31. ^{13}C NMR of the 3ae (101 MHz, $CDCl_3$)

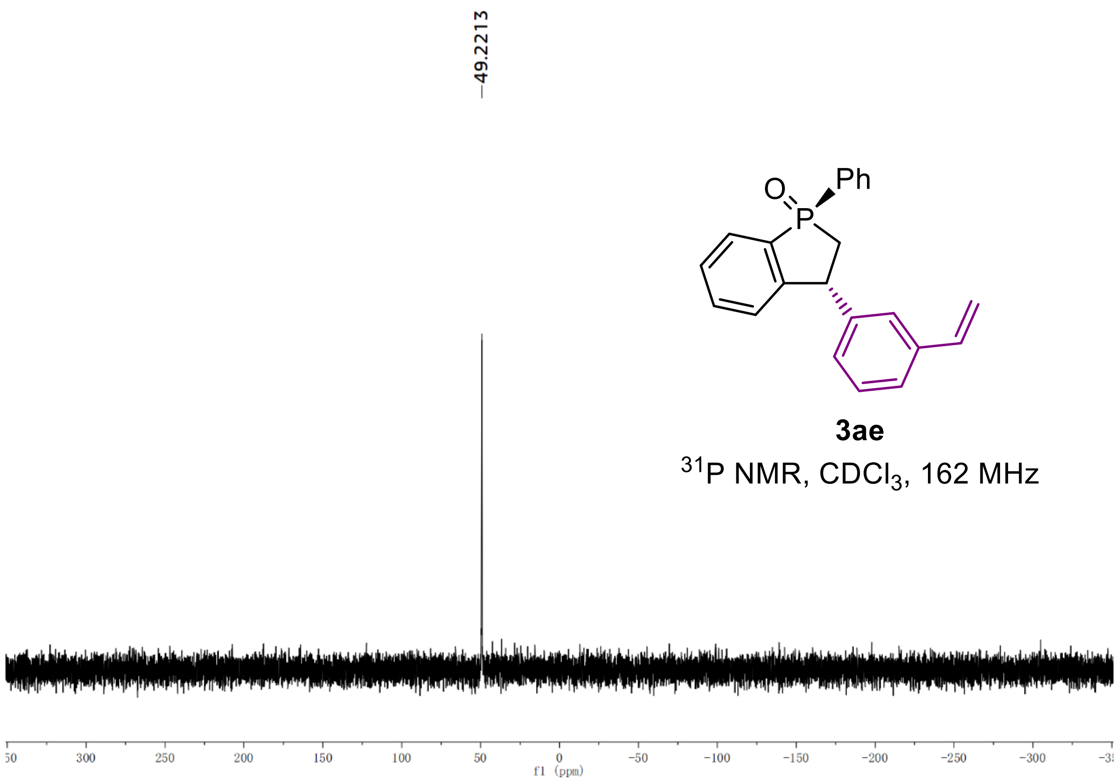

Supplementary Figure 32. ^{31}P NMR of the 3ae (162 MHz, CDCl$_3$)

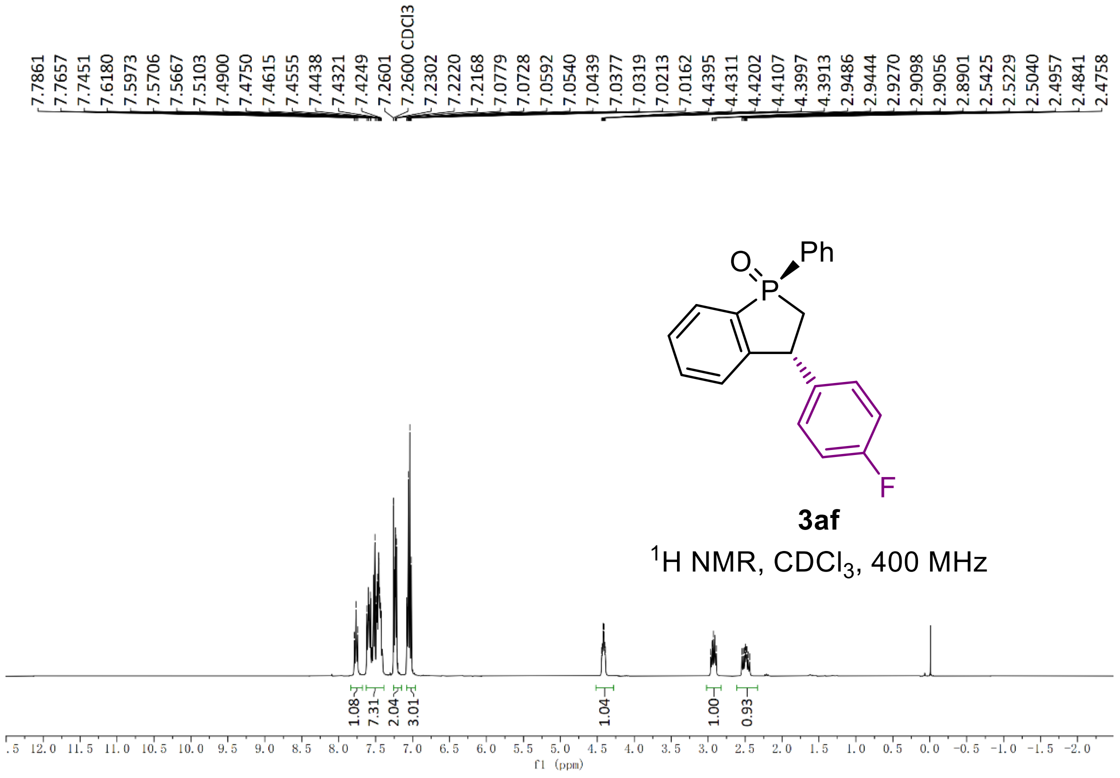

Supplementary Figure 33. ^{1}H NMR of the 3af (400 MHz, CDCl$_3$)

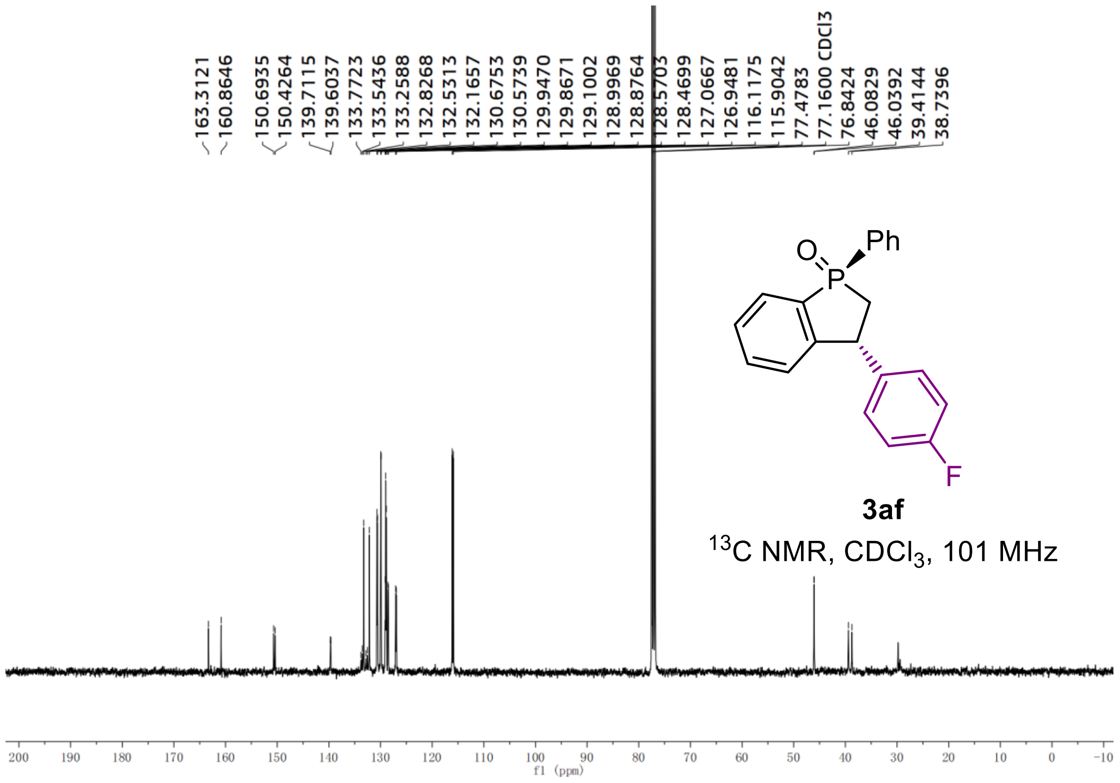

Supplementary Figure 34. ^{13}C NMR of the 3af (101 MHz, $CDCl_3$)

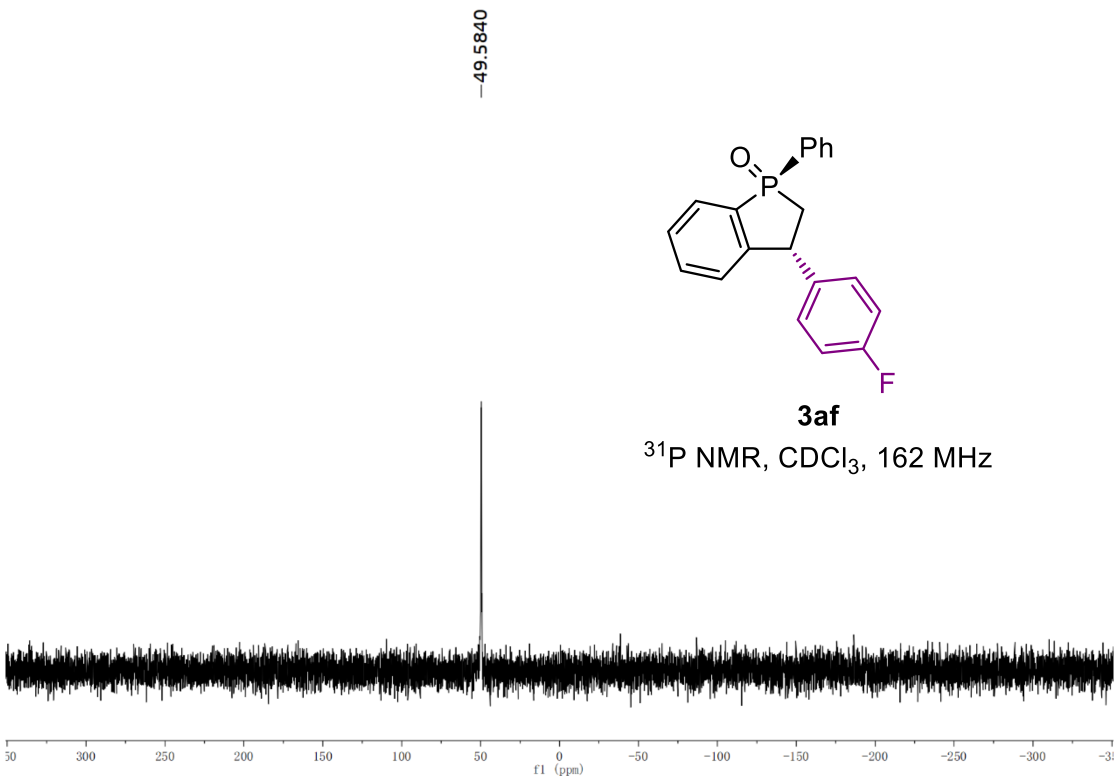

Supplementary Figure 35. ^{31}P NMR of the 3af (162 MHz, $CDCl_3$)

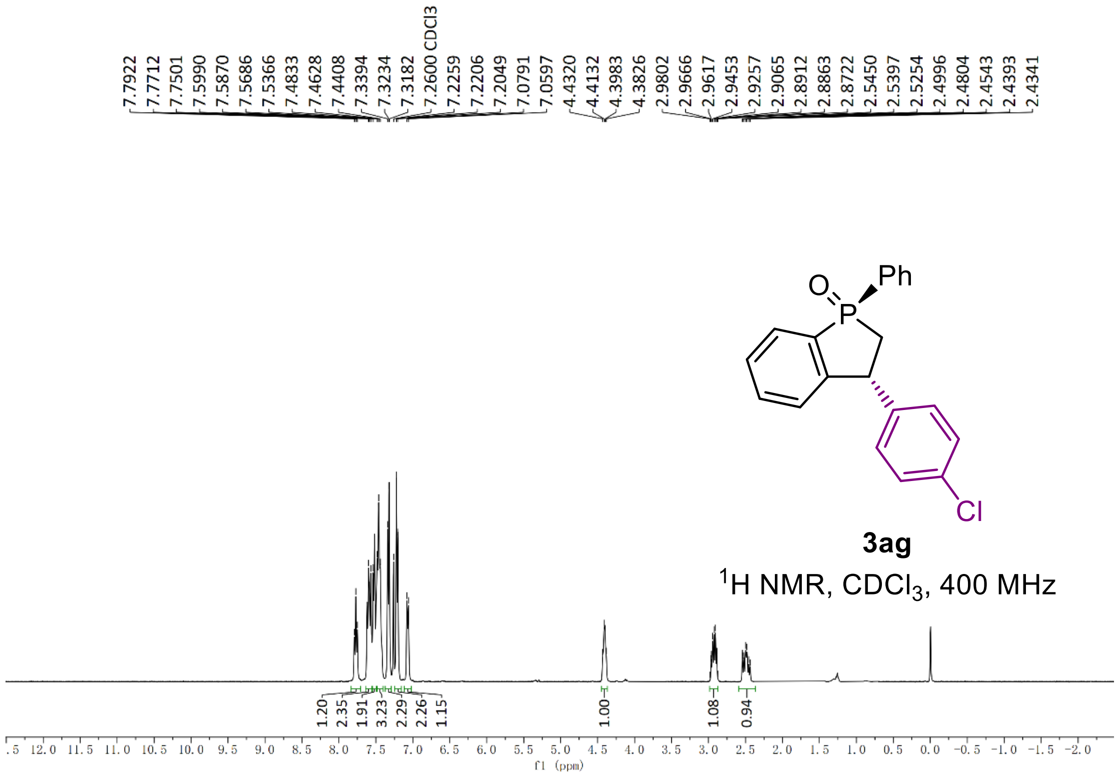

Supplementary Figure 36. ^{1}H NMR of the 3ag (400 MHz, $CDCl_3$)

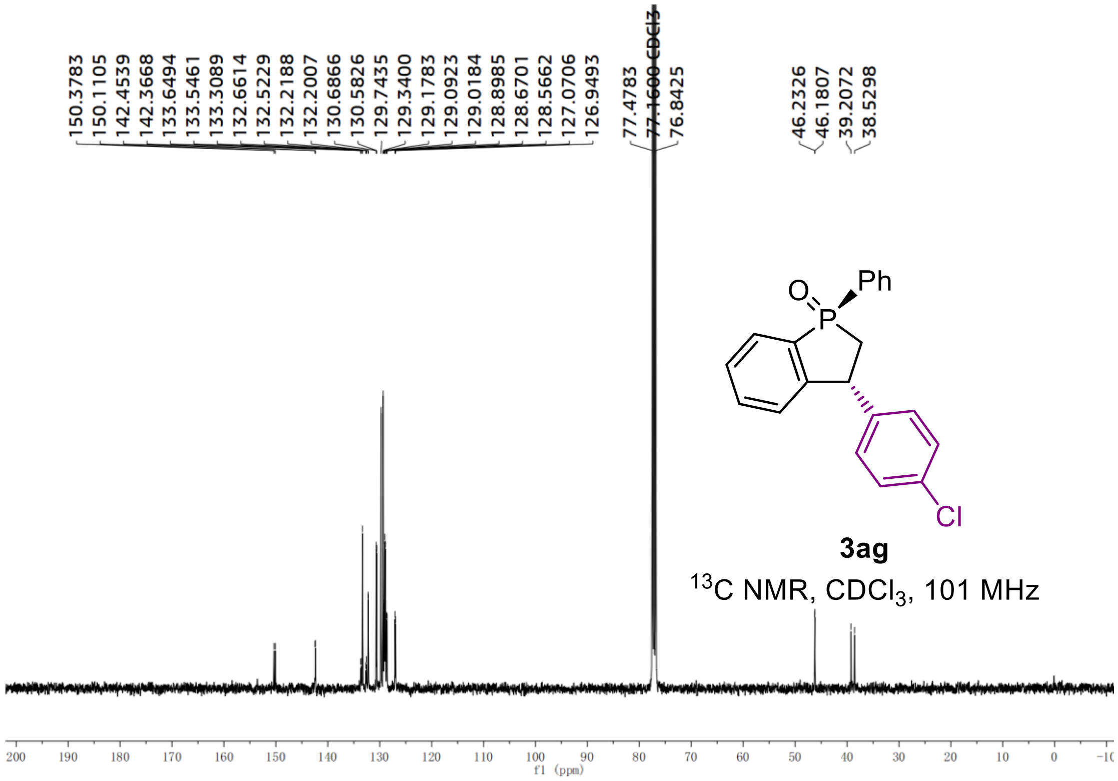

Supplementary Figure 37. ^{13}C NMR of the 3ag (101 MHz, $CDCl_3$)

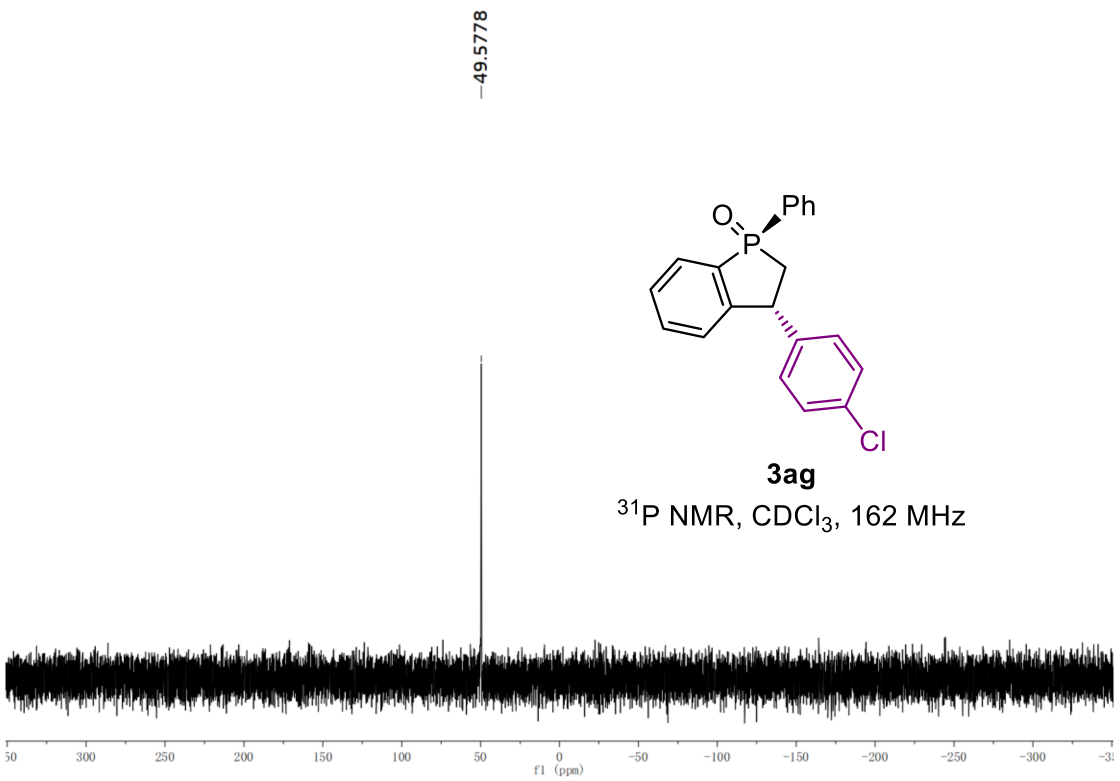

Supplementary Figure 38. ^{31}P NMR of the 3ag (162 MHz, CDCl$_3$)

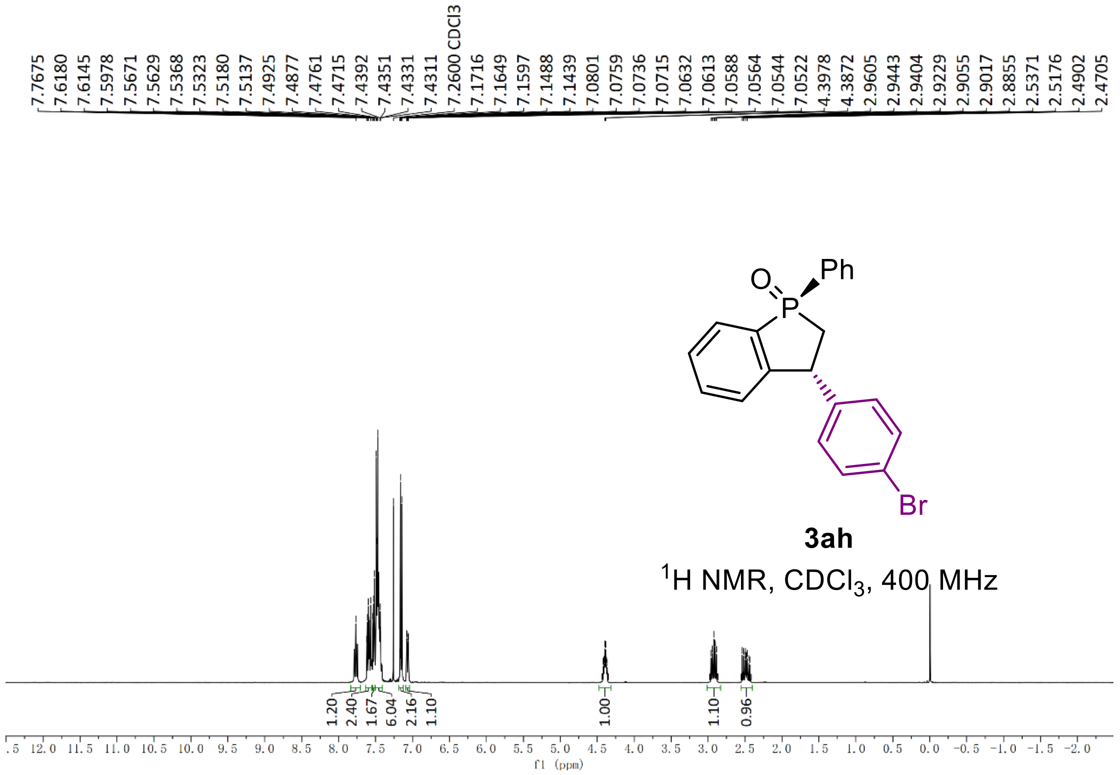

Supplementary Figure 39. ^{1}H NMR of the 3ah (400 MHz, CDCl$_3$)

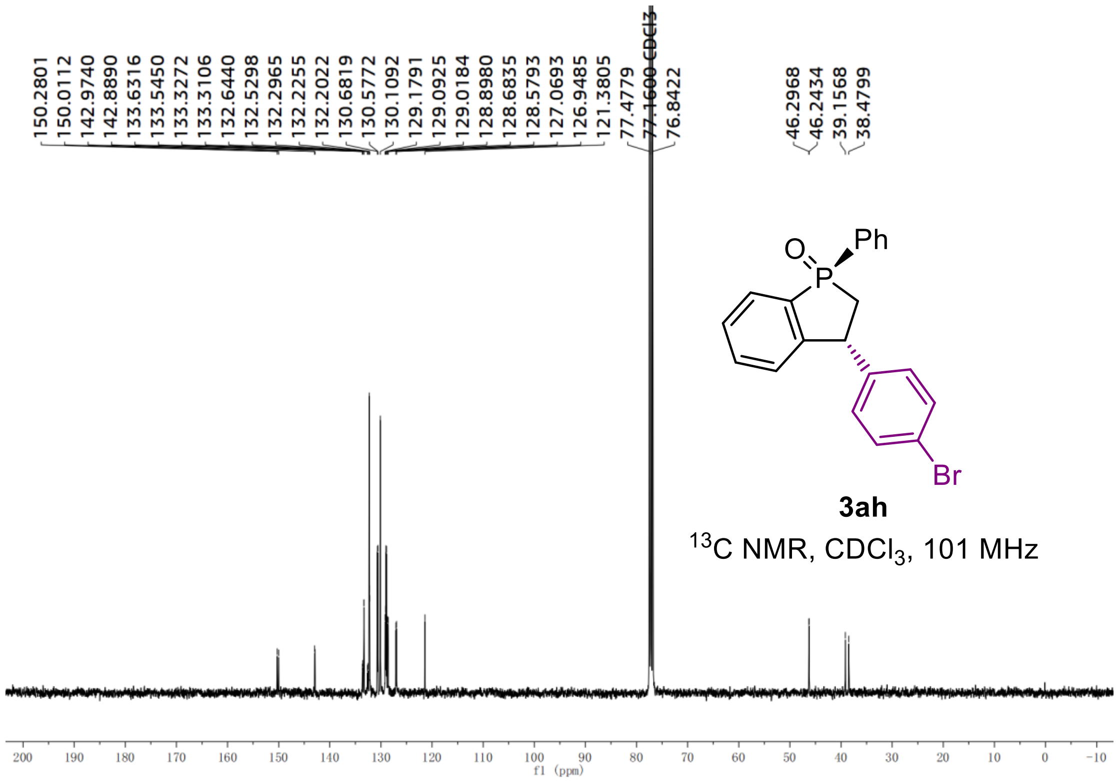

Supplementary Figure 40. ^{13}**C NMR of the 3ah (101 MHz, CDCl$_3$)**

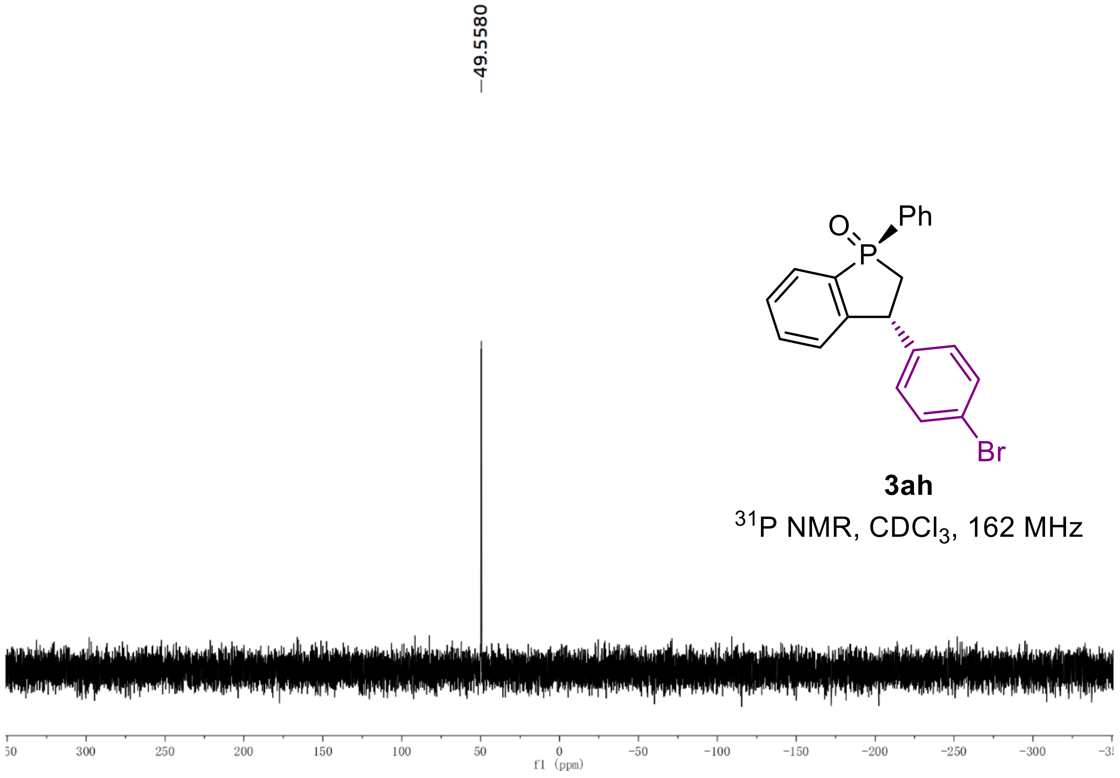

Supplementary Figure 41. ^{31}**P NMR of the 3ah (162 MHz, CDCl$_3$)**

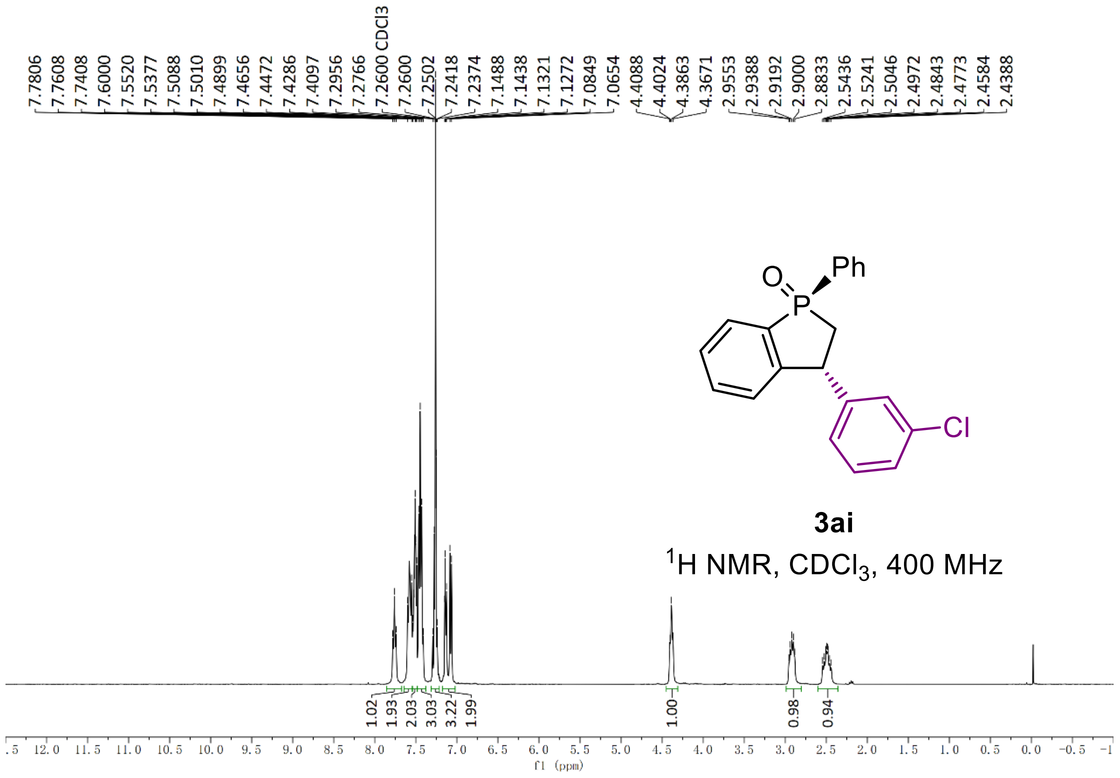

Supplementary Figure 42. ^{1}H NMR of the 3ai (400 MHz, $CDCl_3$)

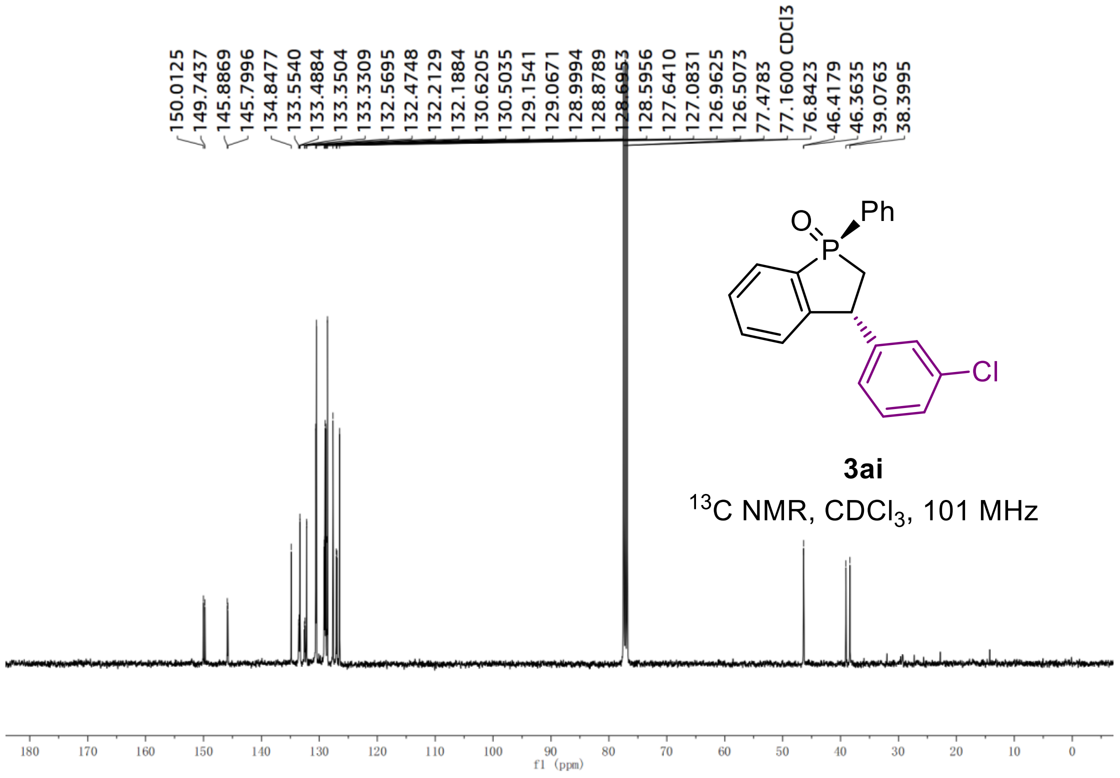

Supplementary Figure 43. ^{13}C NMR of the 3ai (101 MHz, $CDCl_3$)

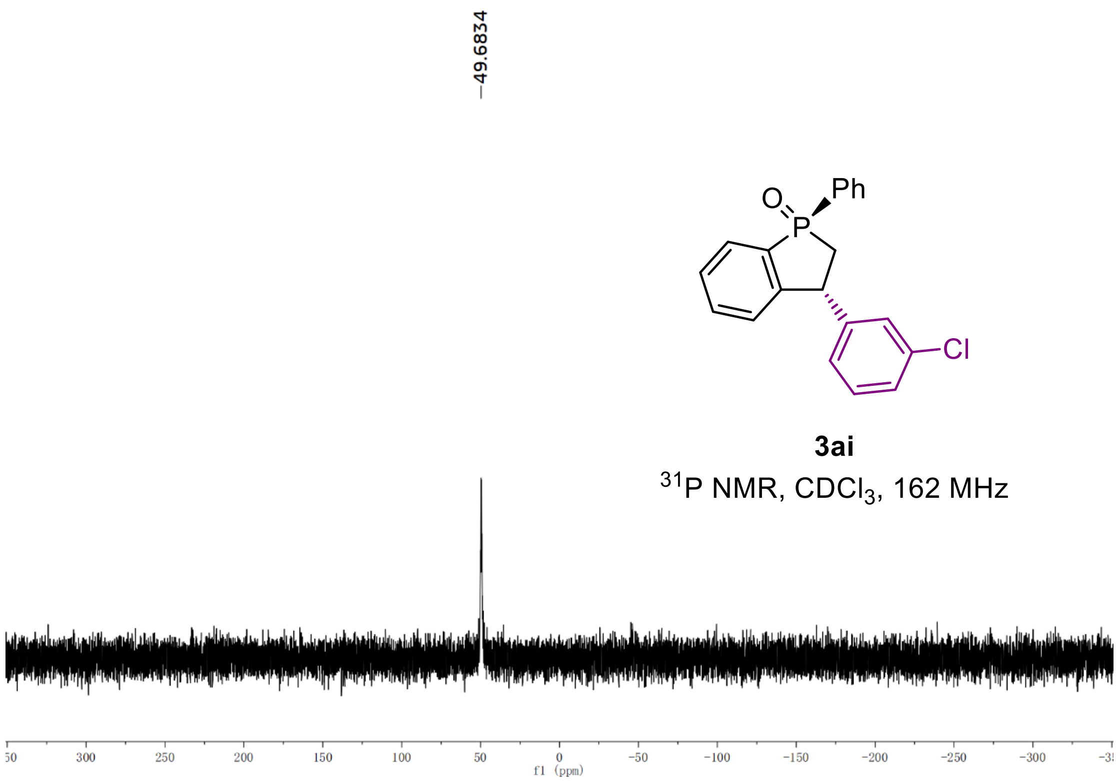

Supplementary Figure 44. ^{31}P NMR of the 3ai (162 MHz, $CDCl_3$)

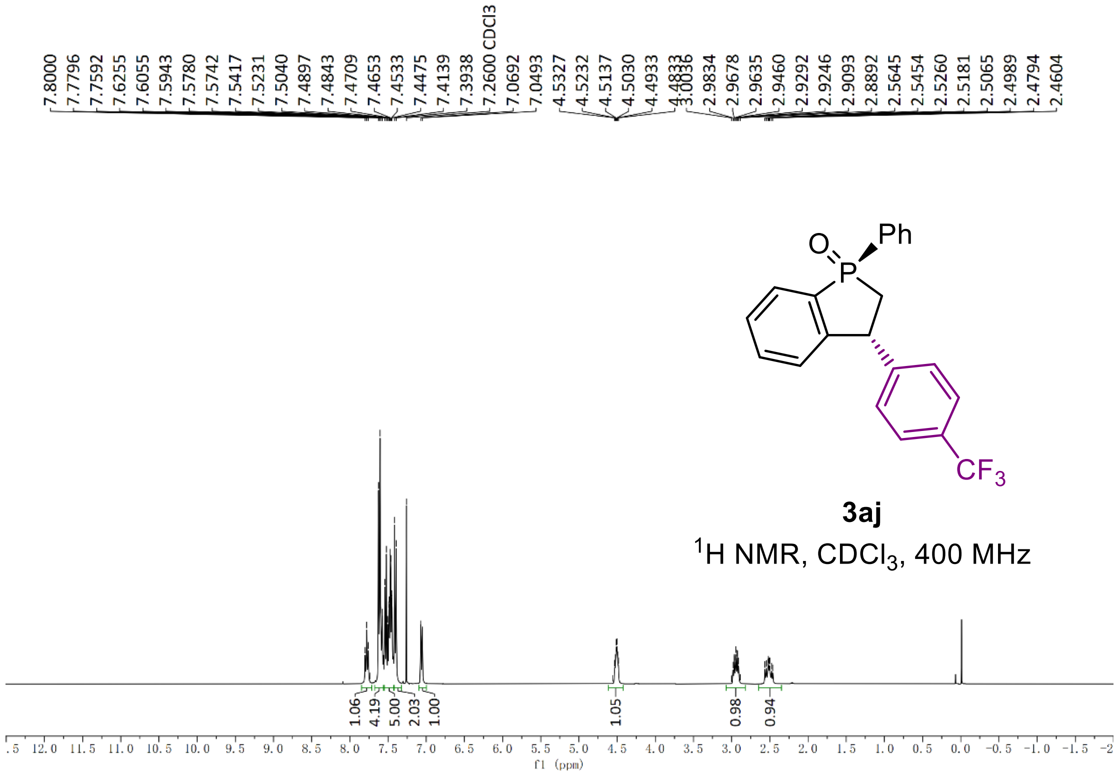

Supplementary Figure 45. ^{1}H NMR of the 3aj (400 MHz, $CDCl_3$)

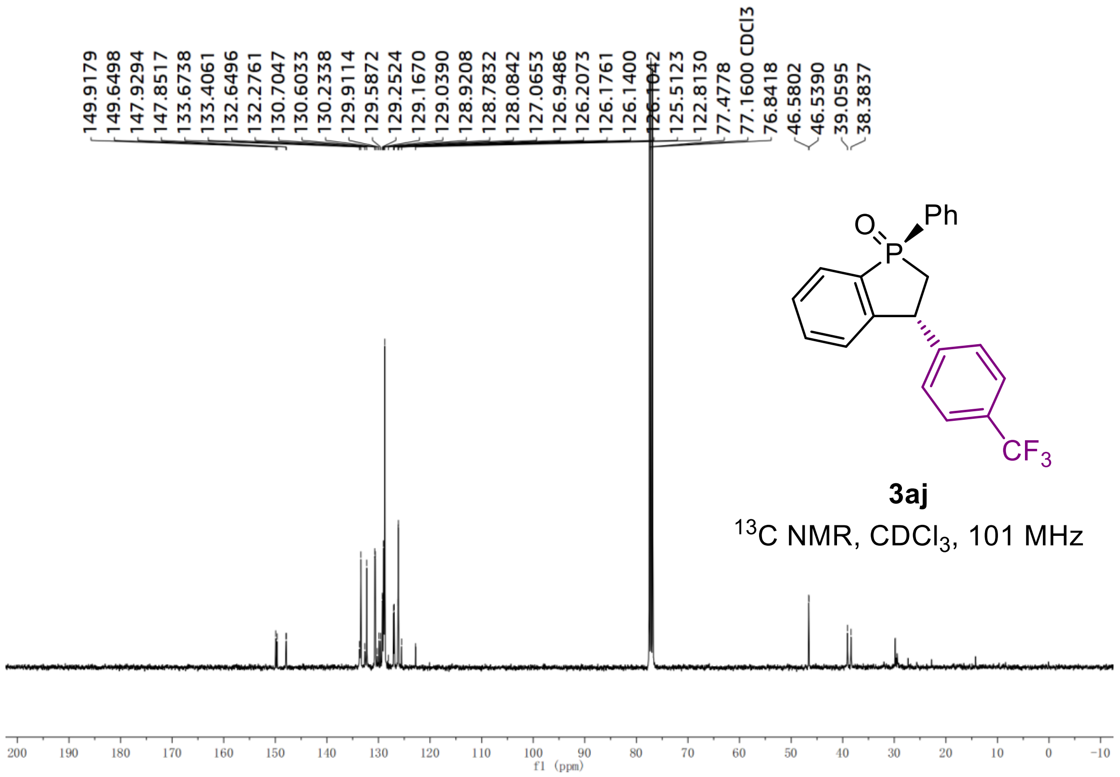

Supplementary Figure 46. ^{13}C NMR of the 3aj (101 MHz, $CDCl_3$)

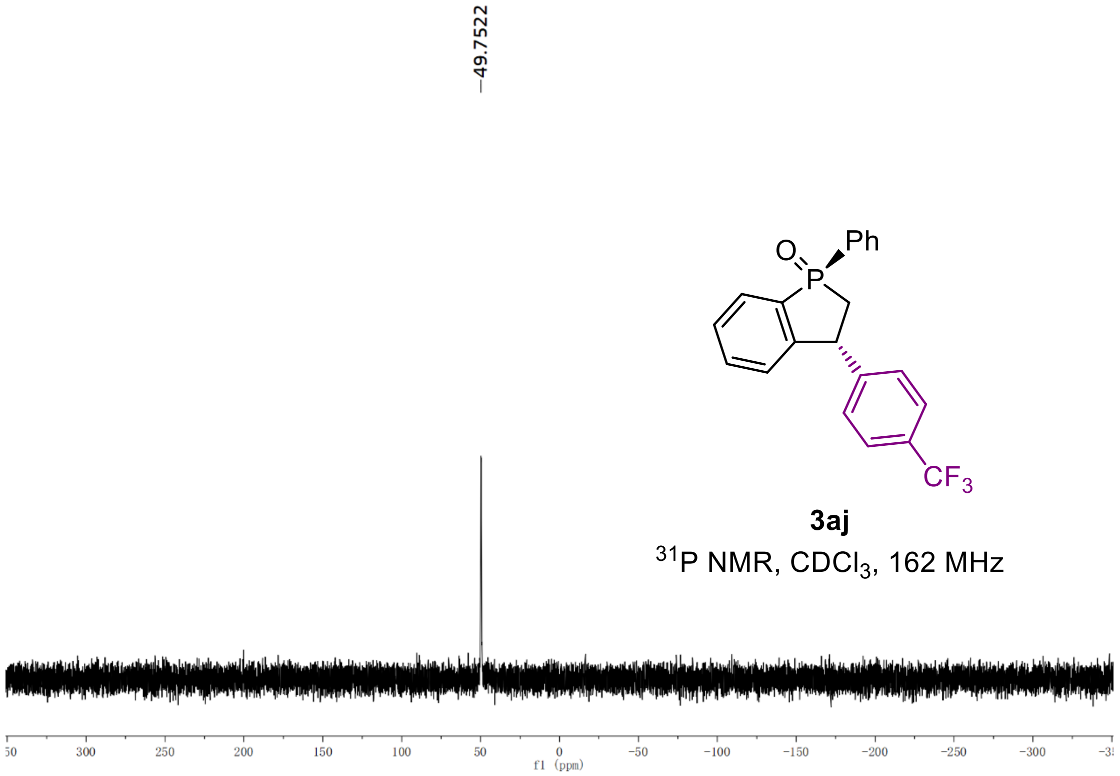

Supplementary Figure 47. ^{13}P NMR of the 3aj (162 MHz, $CDCl_3$)

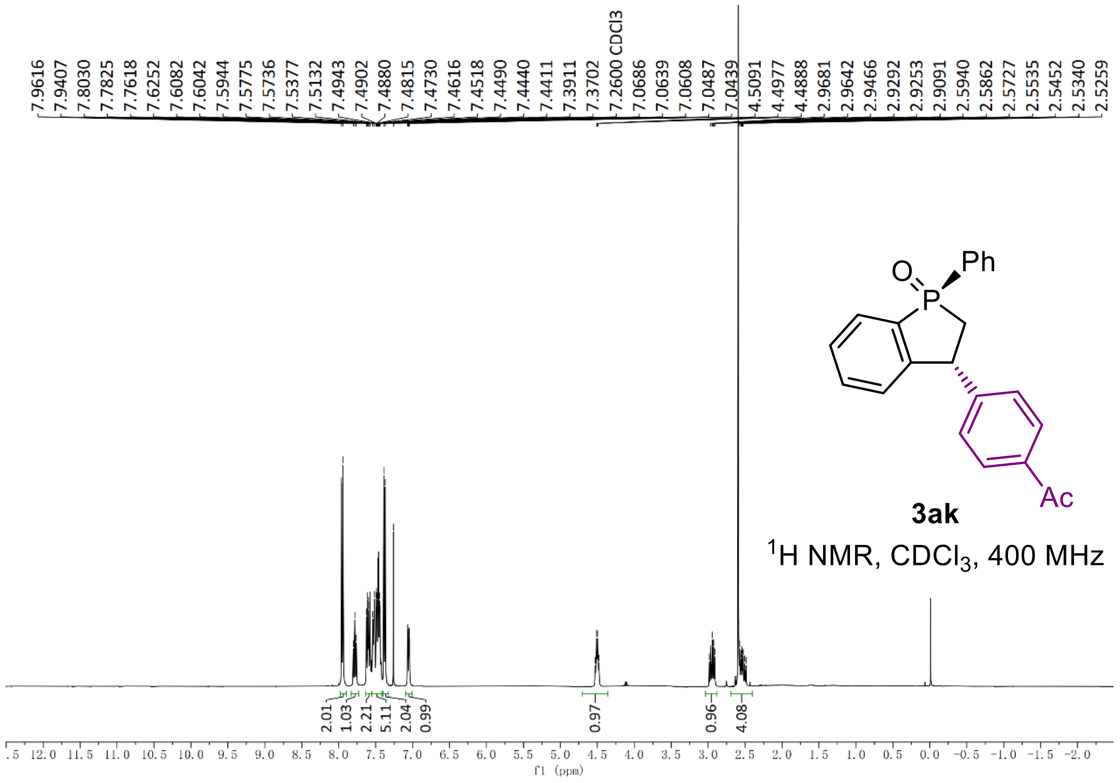

Supplementary Figure 48. ^{1}H NMR of the 3ak (400 MHz, $CDCl_3$)

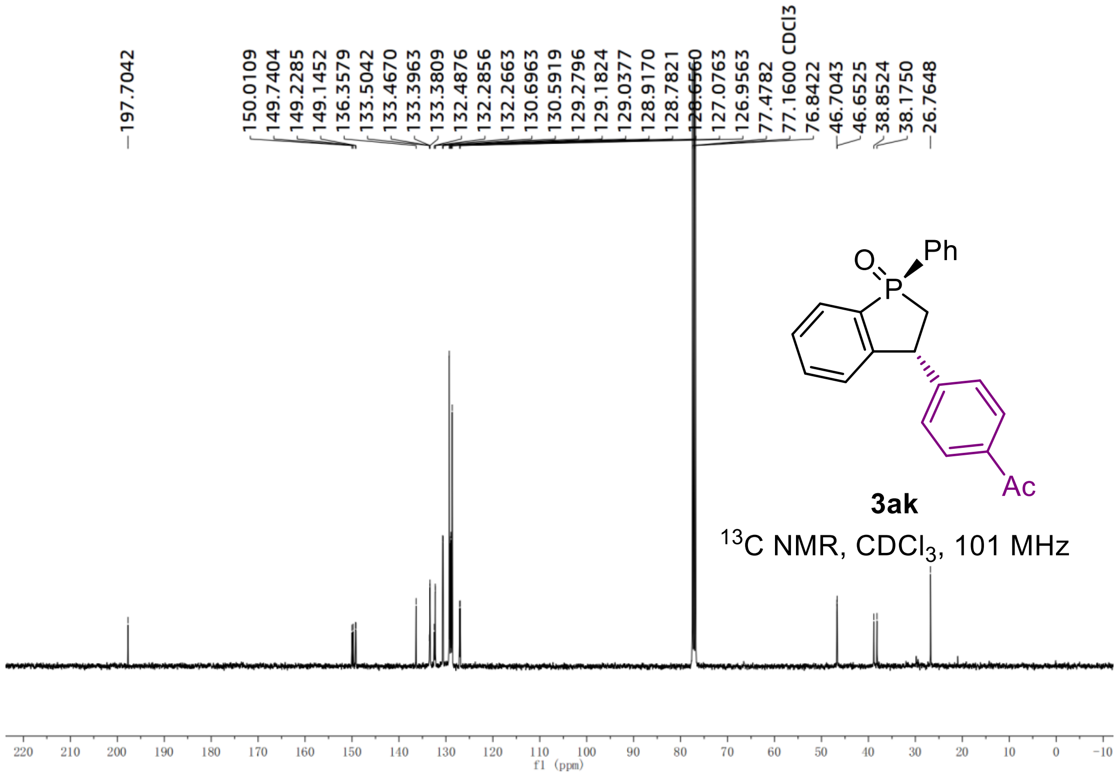

Supplementary Figure 49. ^{13}C NMR of the 3ak (101 MHz, $CDCl_3$)

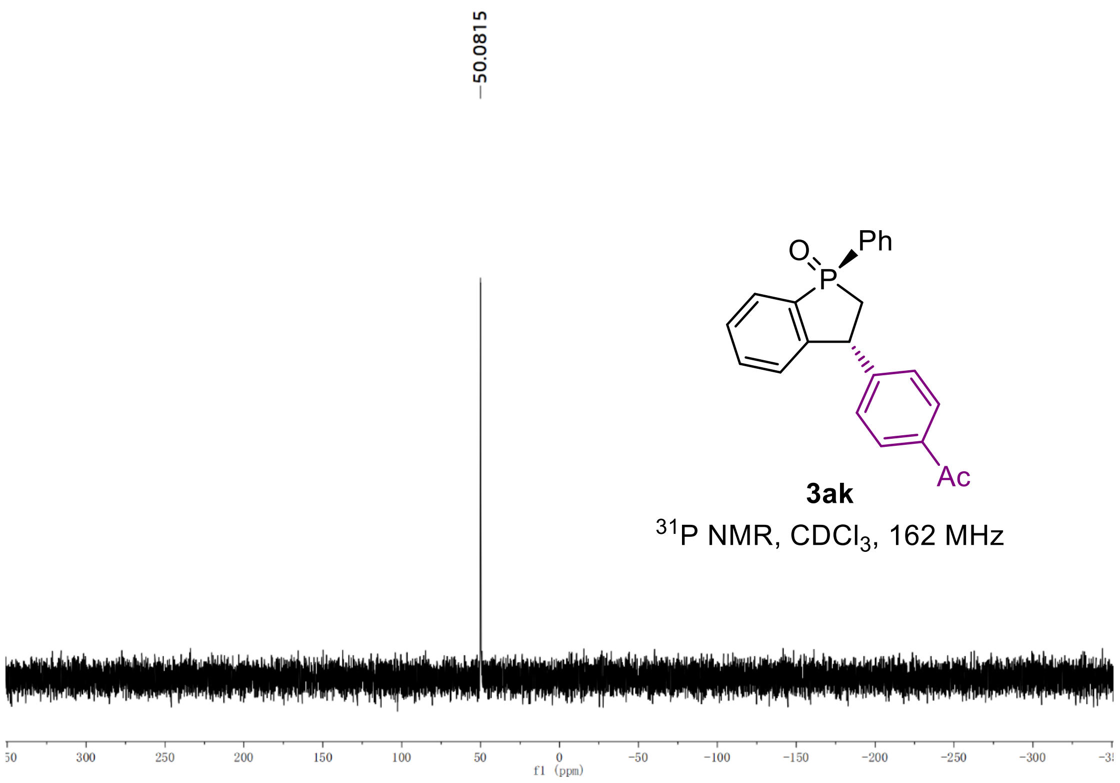

Supplementary Figure 50. ^{31}P NMR of the 3ak (162 MHz, $CDCl_3$)

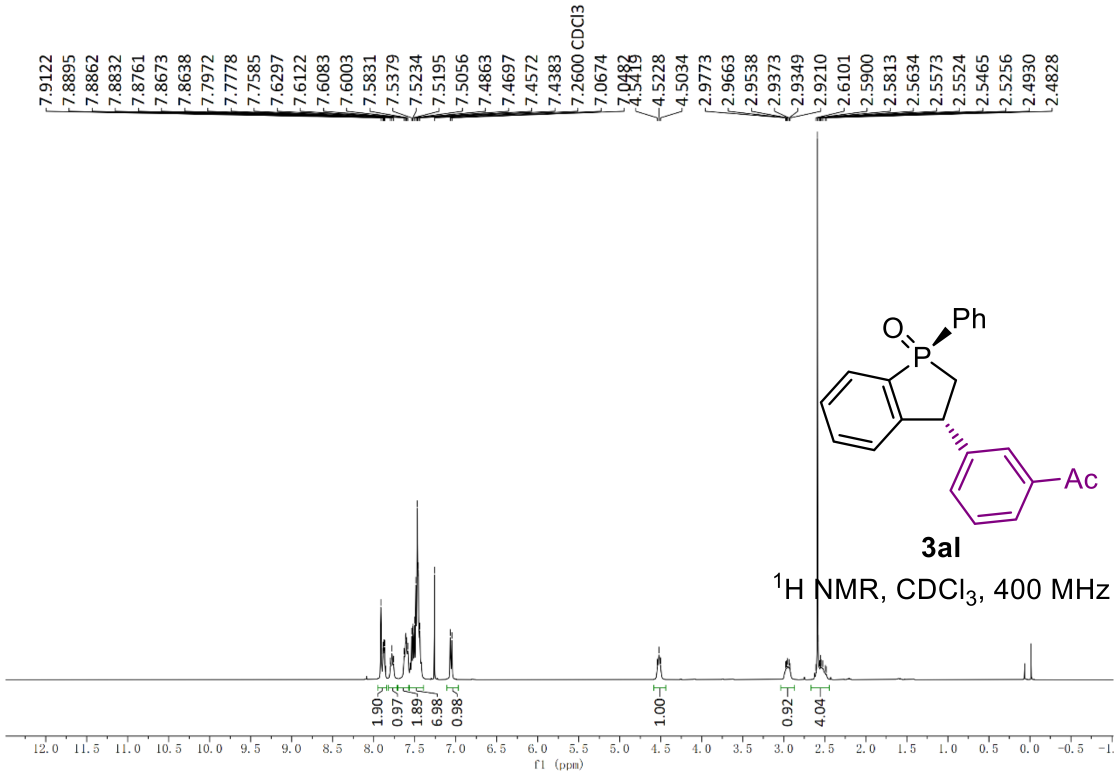

Supplementary Figure 51. ^{1}H NMR of the 3al (400 MHz, $CDCl_3$)

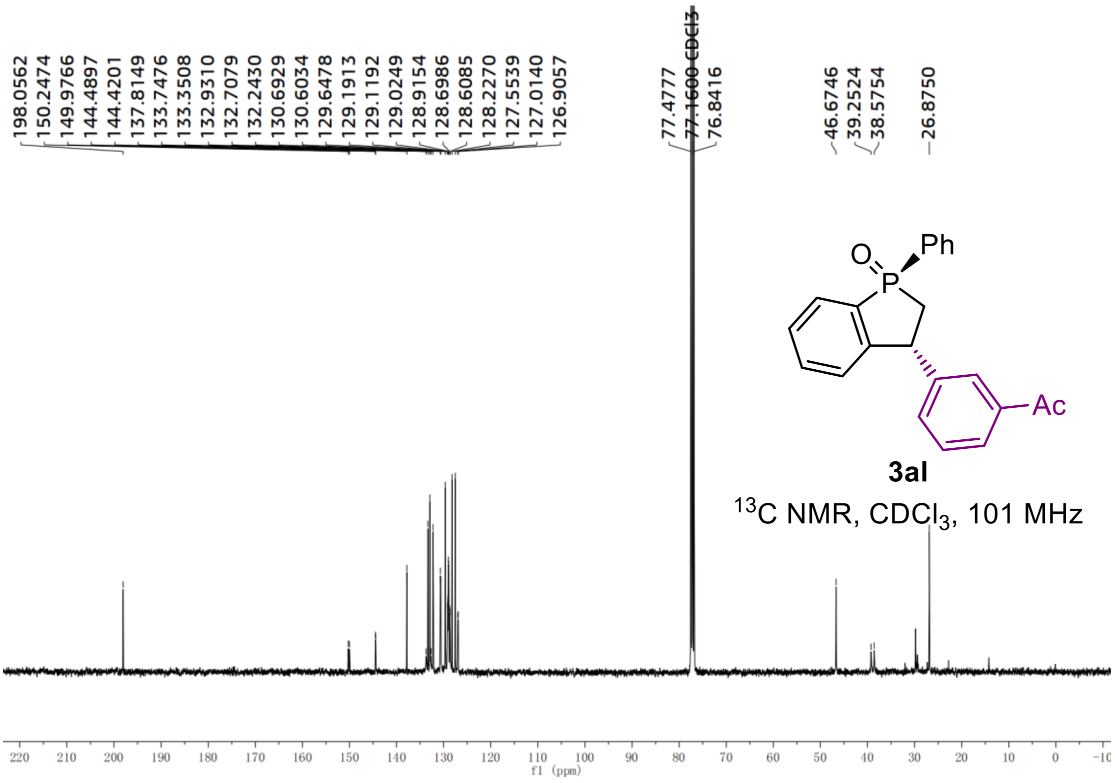

Supplementary Figure 52. ^{13}C NMR of the 3al (101 MHz, $CDCl_3$)

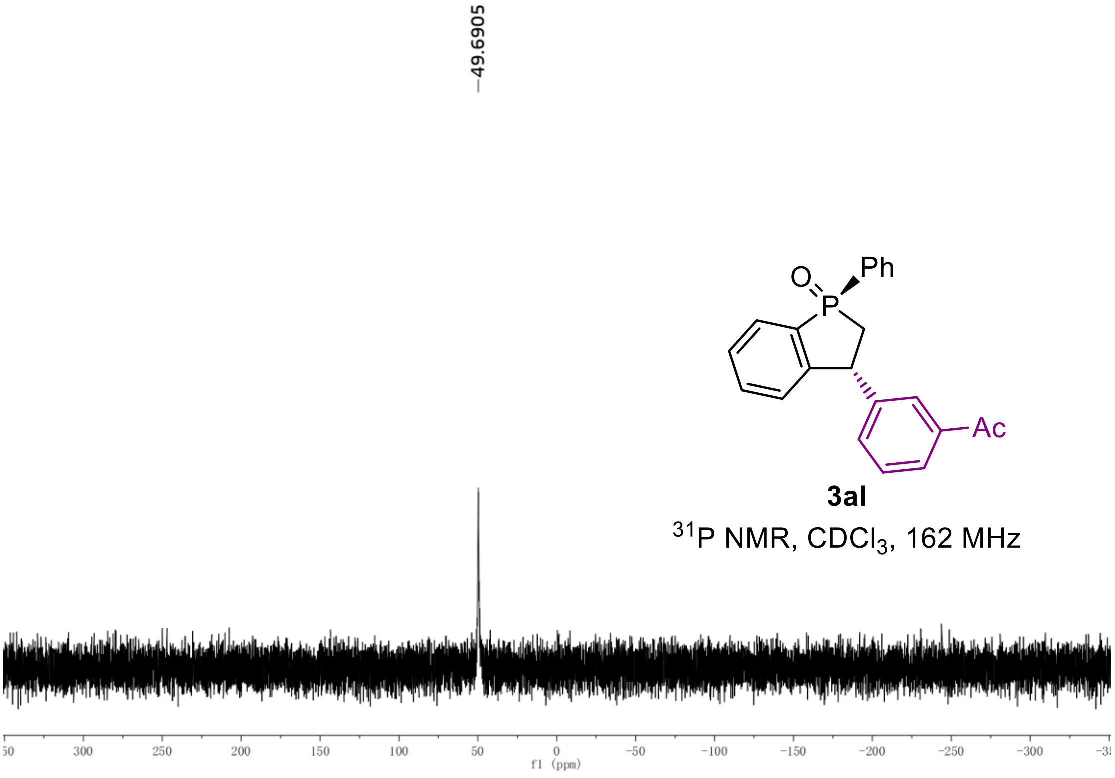

Supplementary Figure 53. ^{31}P NMR of the 3al (162 MHz, $CDCl_3$)

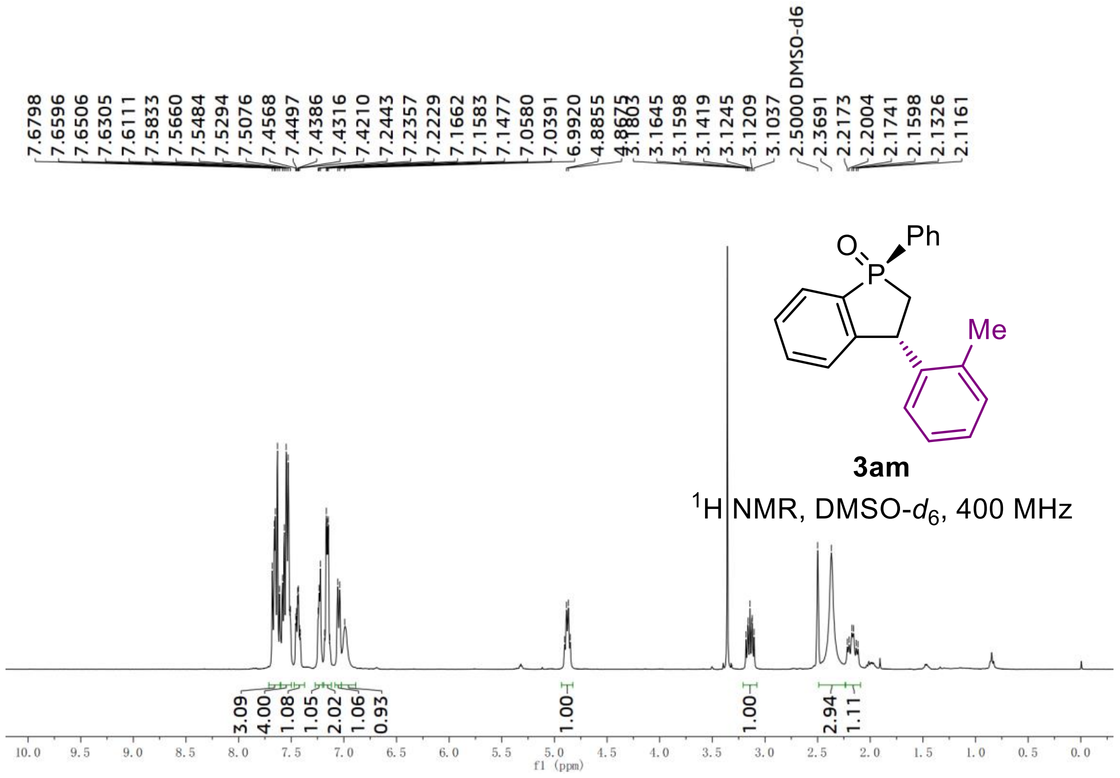

Supplementary Figure 54. ^{1}H NMR of the 3am (400 MHz, DMSO-d_6)

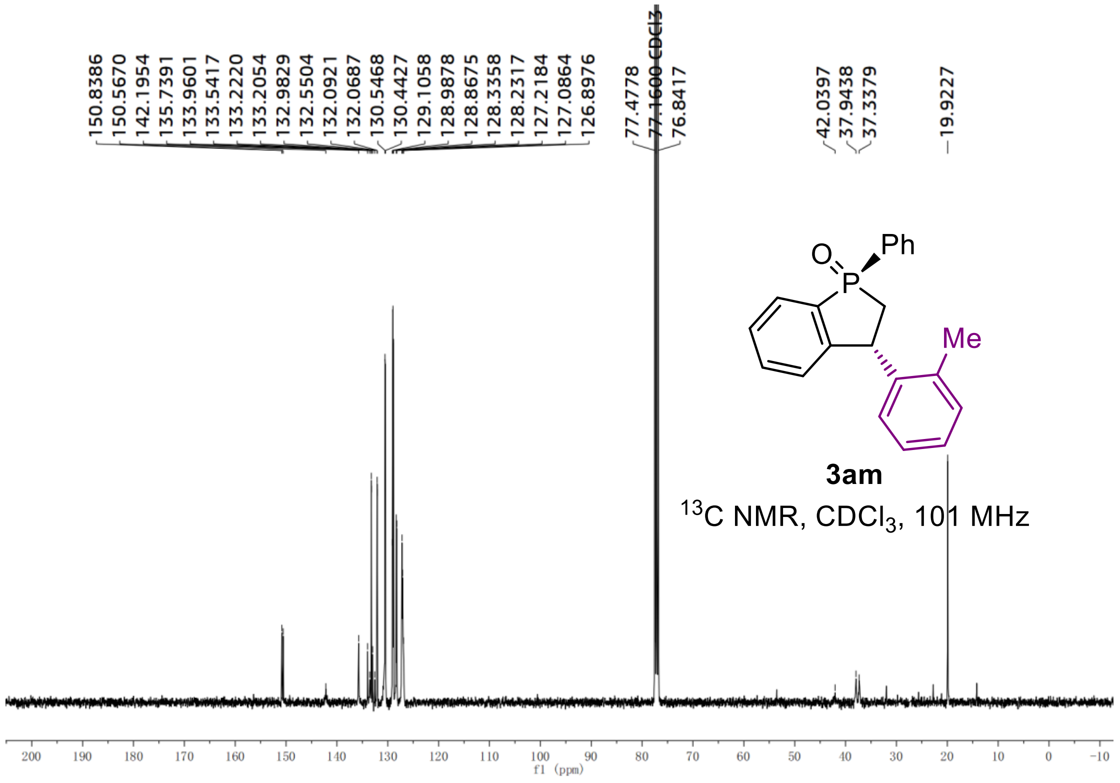

Supplementary Figure 55. ^{13}C NMR of the 3am (101 MHz, $CDCl_3$)

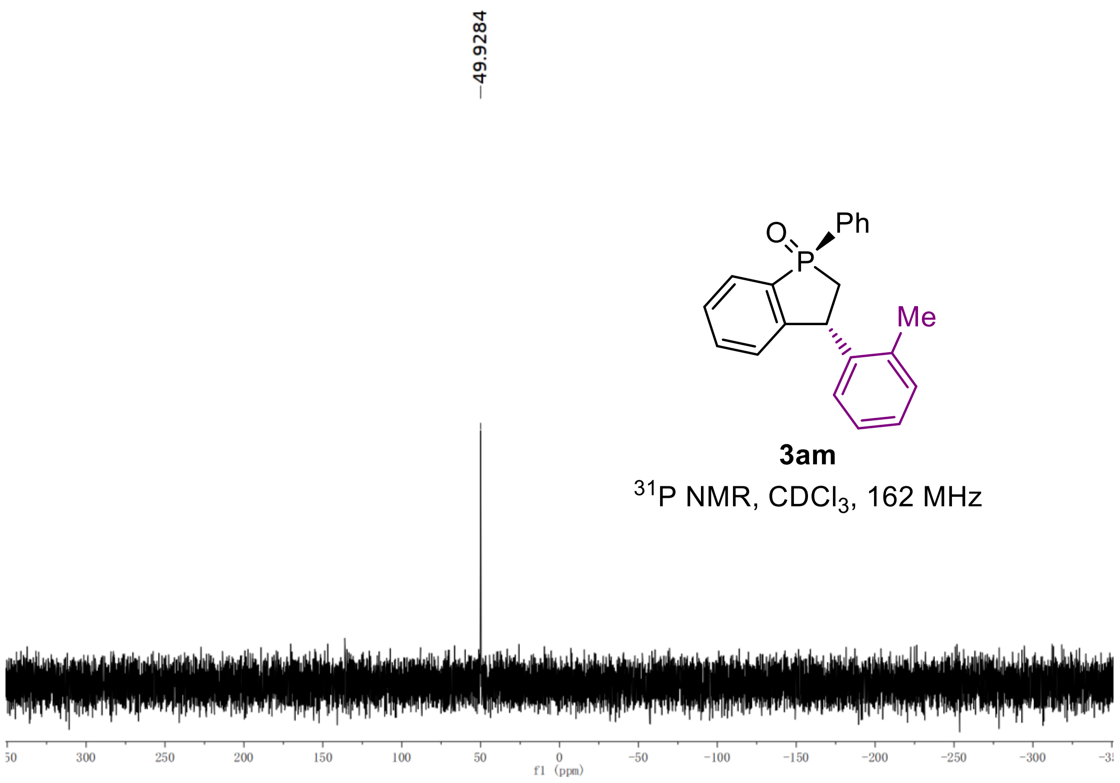

Supplementary Figure 56. ^{31}P NMR of the 3am (162 MHz, CDCl$_3$)

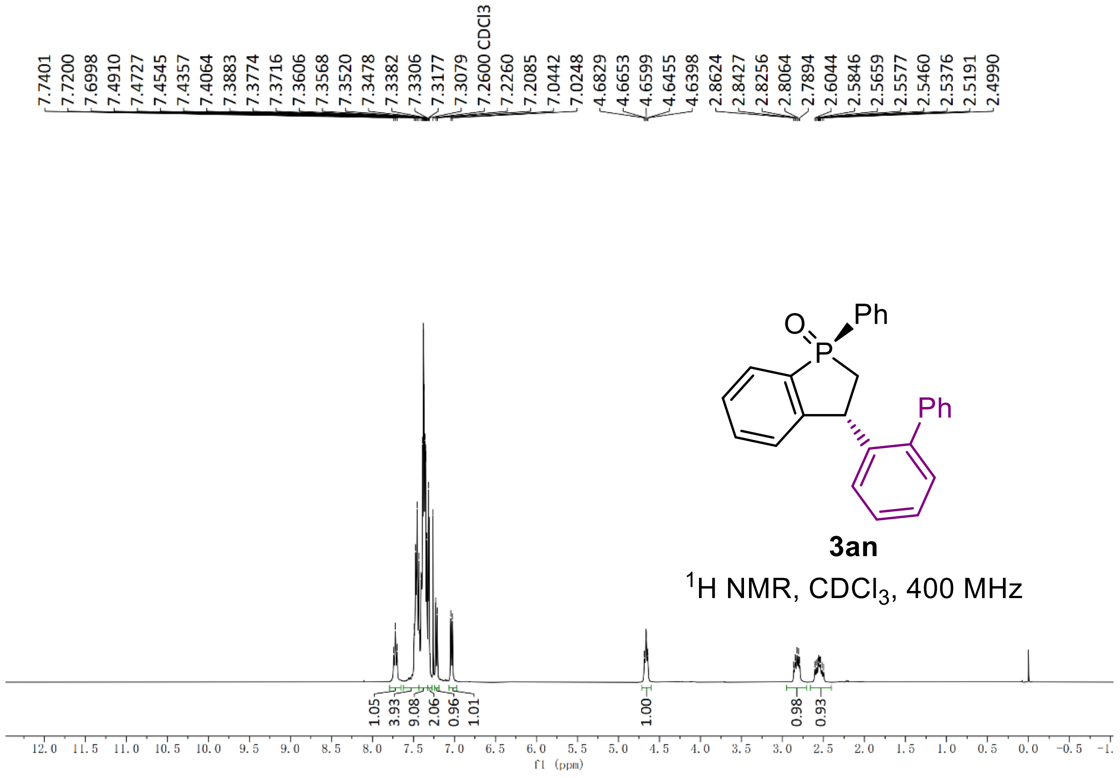

Supplementary Figure 57. ^{1}H NMR of the 3an (400 MHz, CDCl$_3$)

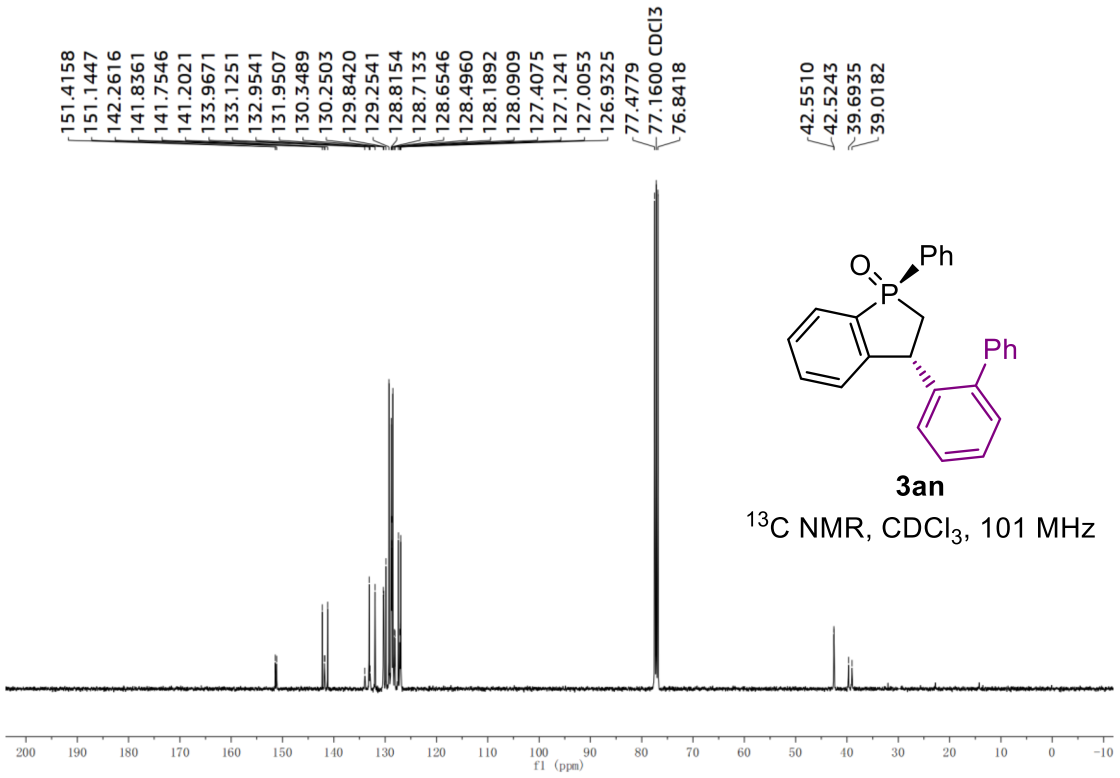

Supplementary Figure 58. ^{13}C NMR of the 3an (101 MHz, $CDCl_3$)

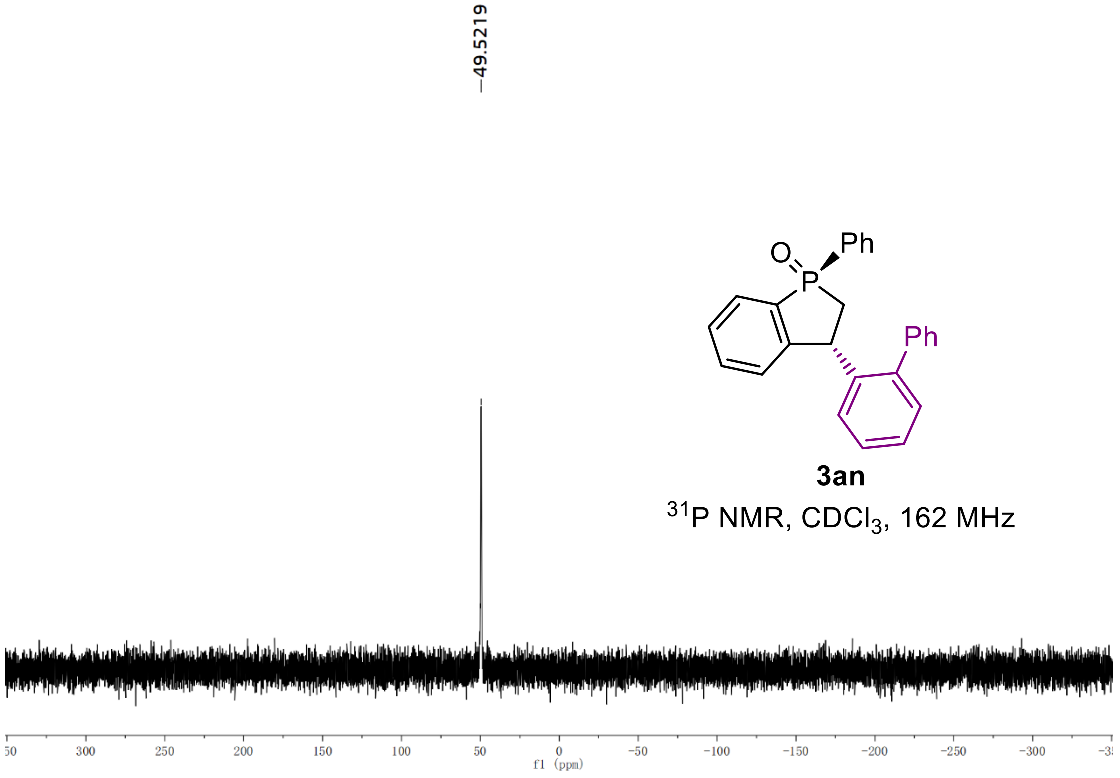

Supplementary Figure 59. ^{31}P NMR of the 3an (162 MHz, $CDCl_3$)

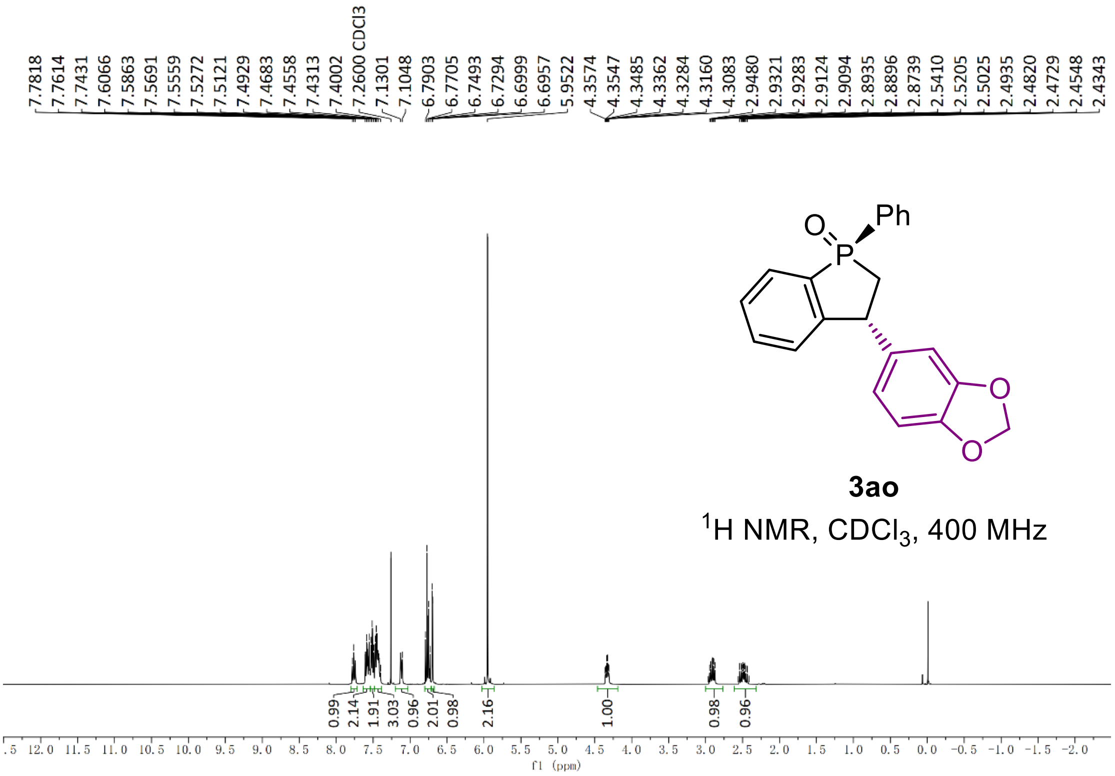

Supplementary Figure 60. ^{1}H NMR of the 3ao (400 MHz, $CDCl_3$)

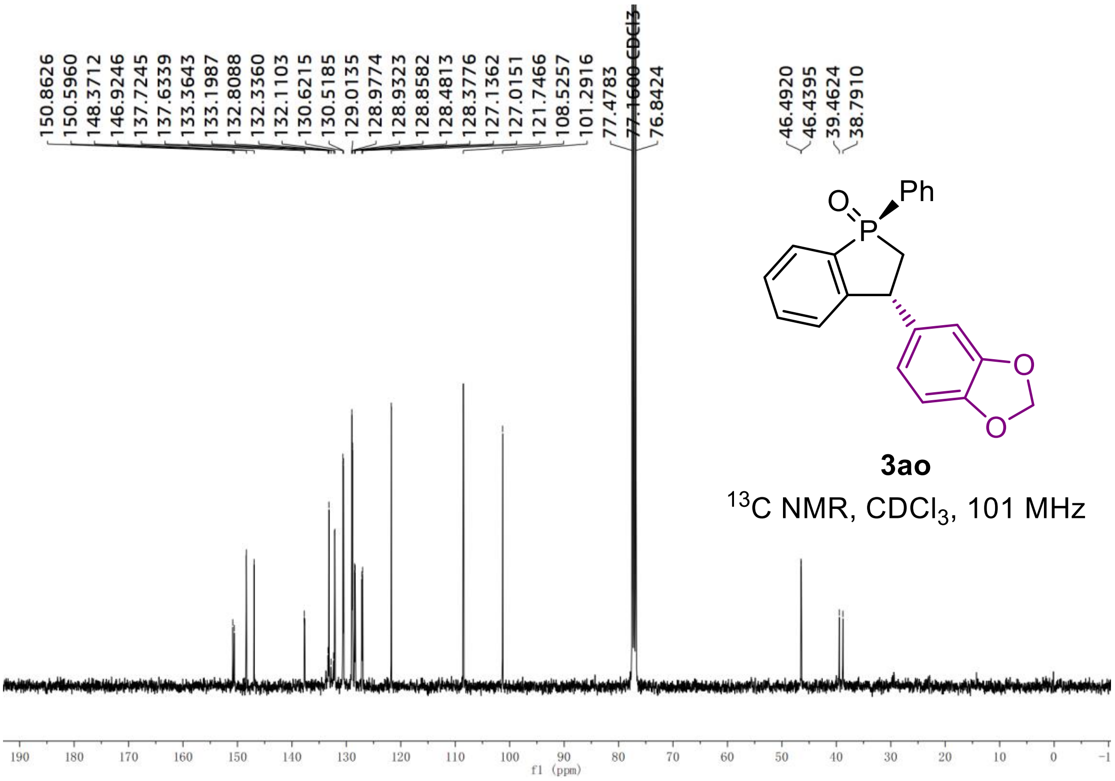

Supplementary Figure 61. ^{13}C NMR of the 3ao (101 MHz, $CDCl_3$)

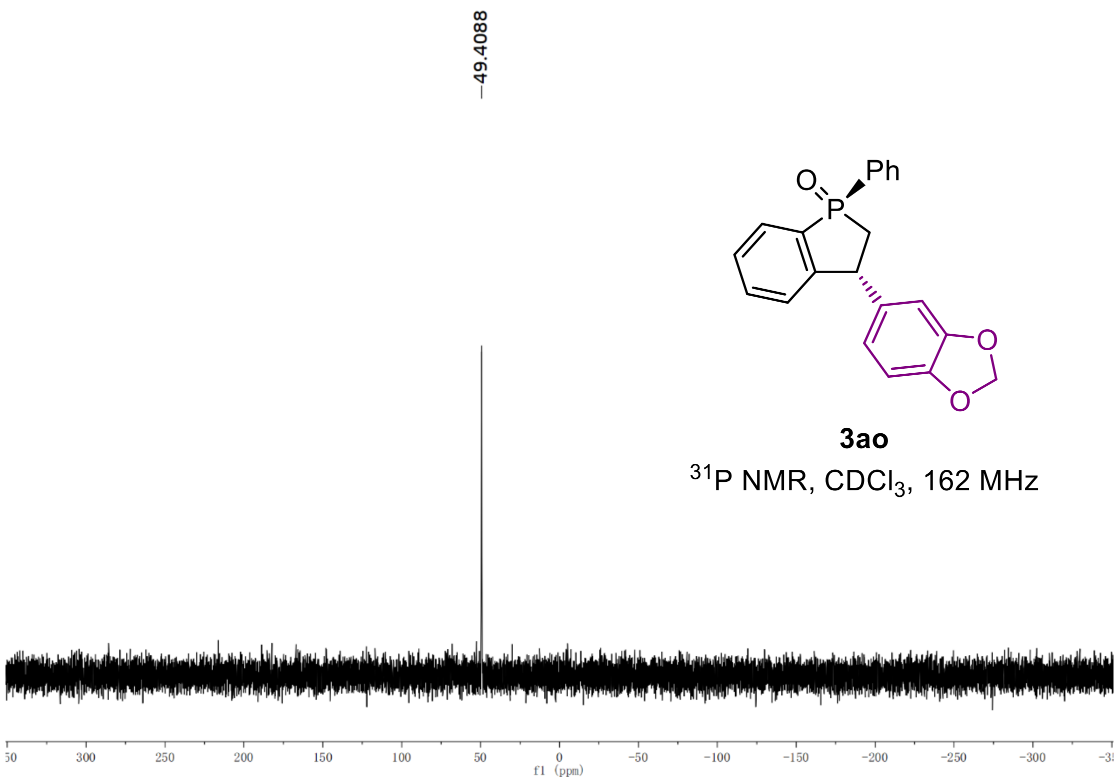

Supplementary Figure 62. ^{31}P NMR of the 3ao (162 MHz, $CDCl_3$)

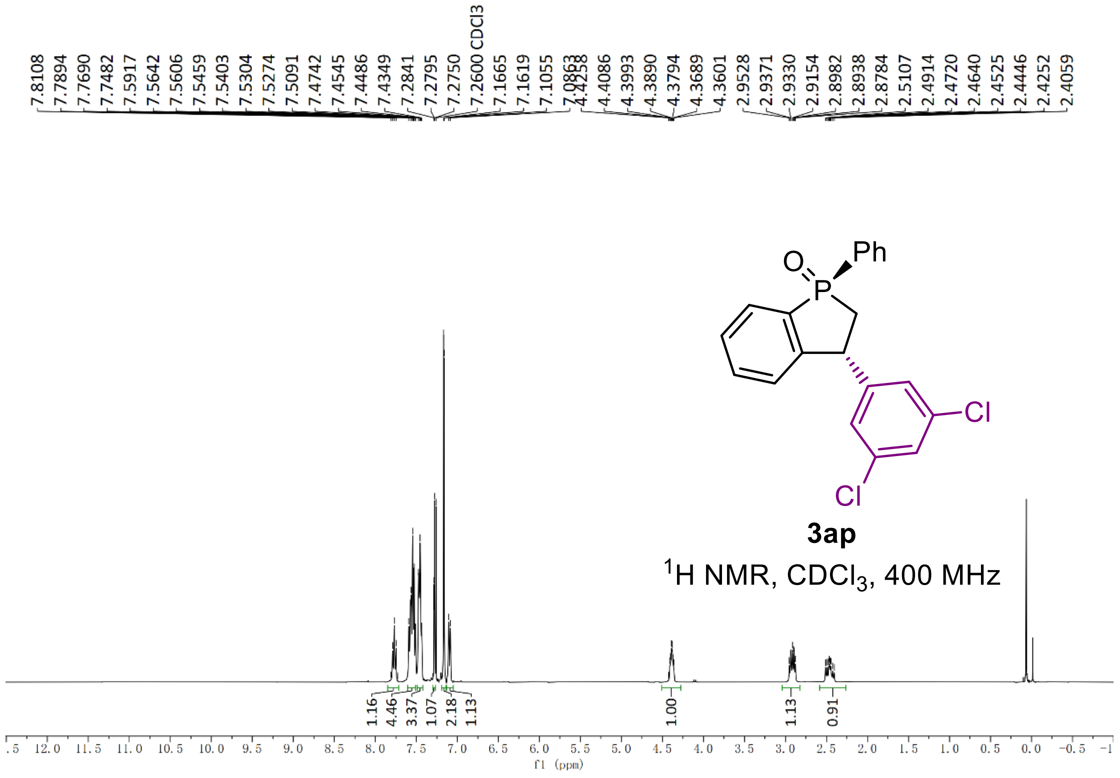

Supplementary Figure 63. ^{1}H NMR of the 3ap (400 MHz, $CDCl_3$)

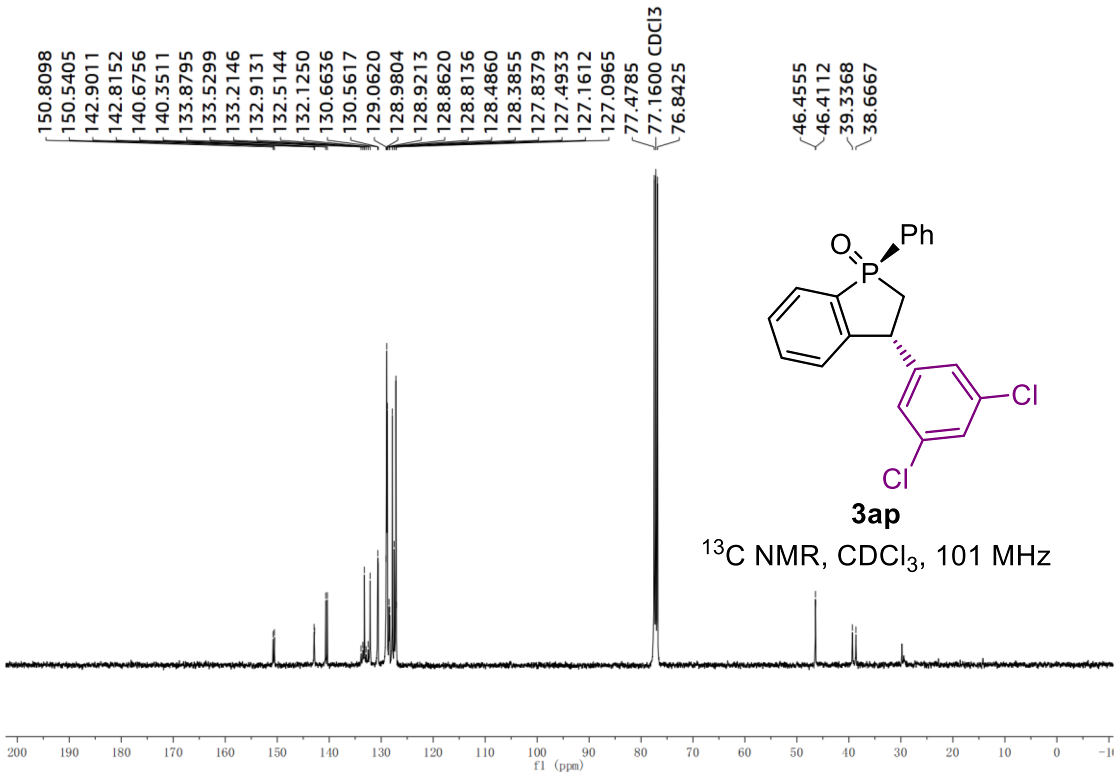

Supplementary Figure 64. ^{13}**C NMR of the 3ap (101 MHz, CDCl$_3$)**

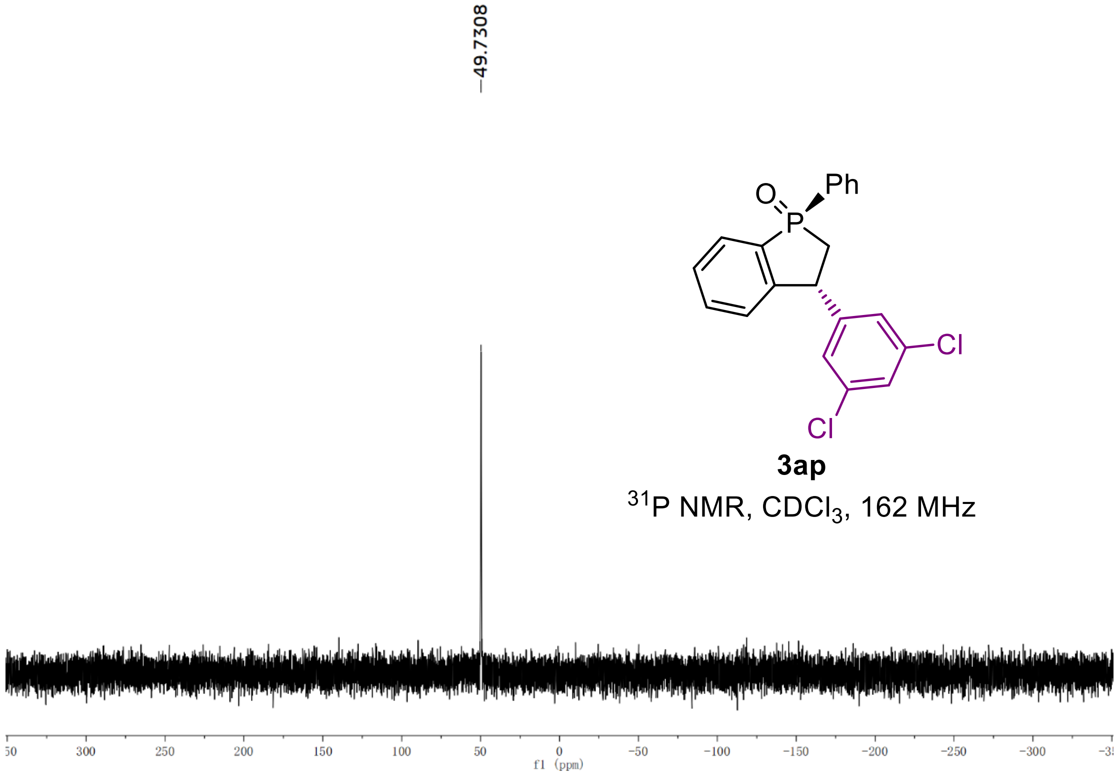

Supplementary Figure 65. ^{31}**P NMR of the 3ap (162 MHz, CDCl$_3$)**

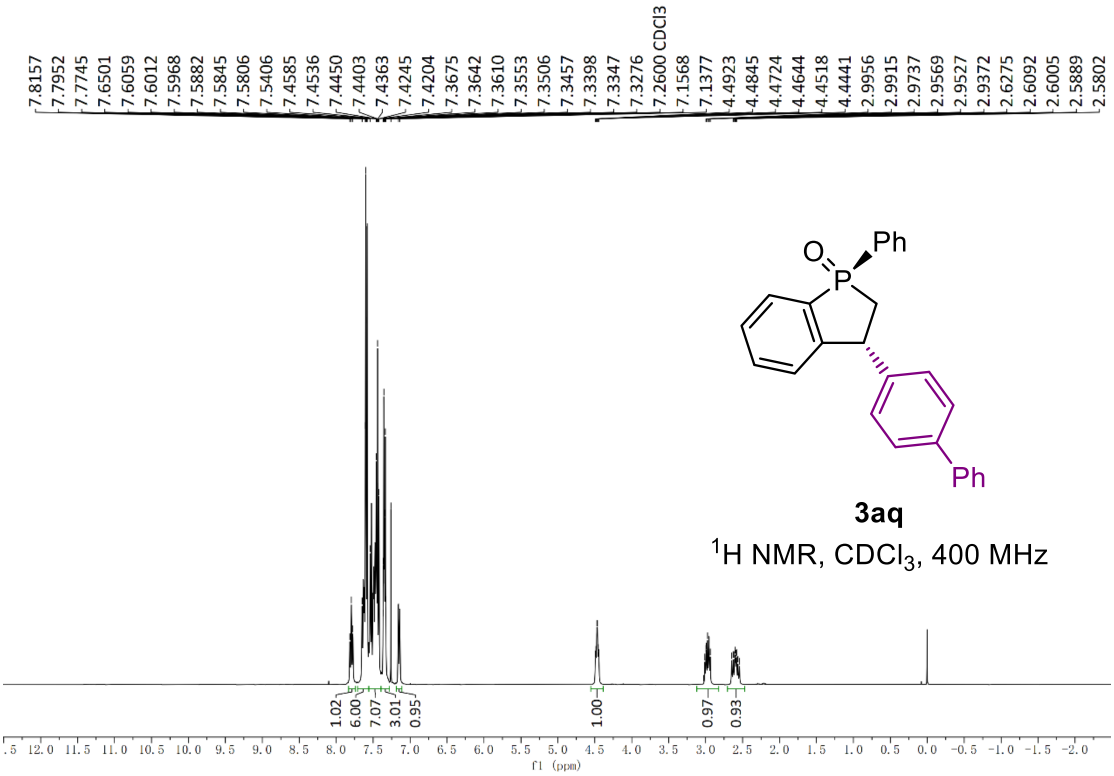

Supplementary Figure 66. ^{1}H NMR of the 3aq (400 MHz, $CDCl_3$)

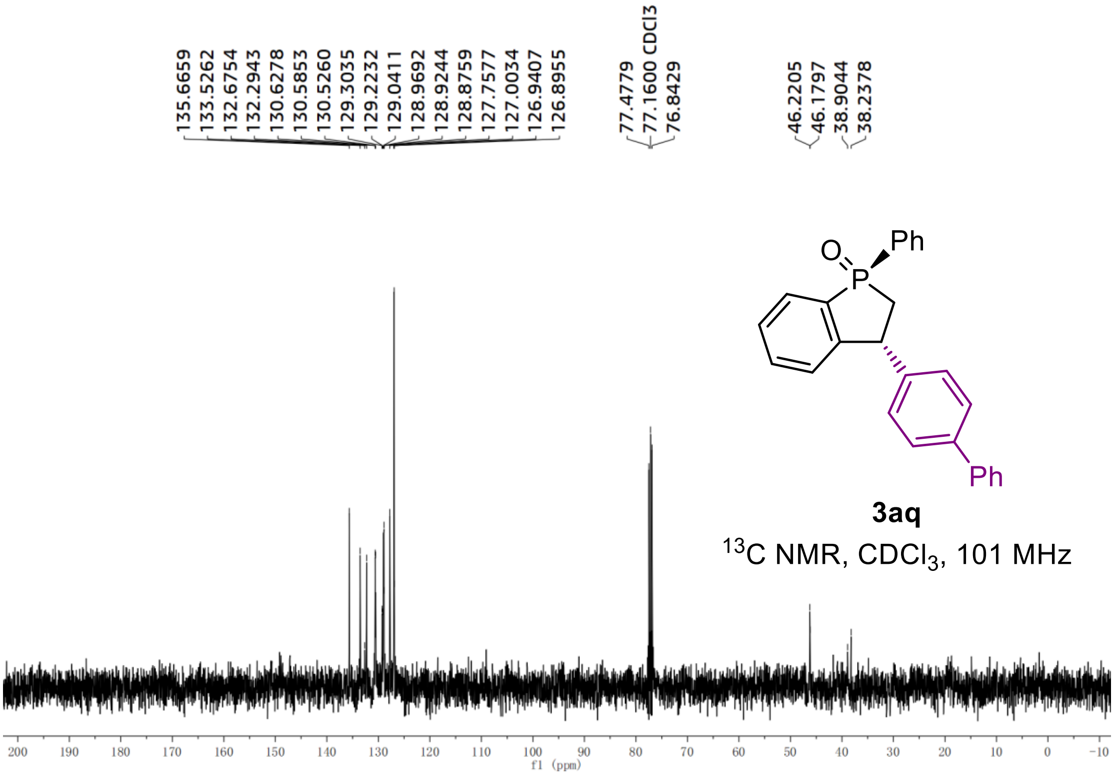

Supplementary Figure 67. ^{13}C NMR of the 3aq (101 MHz, $CDCl_3$)

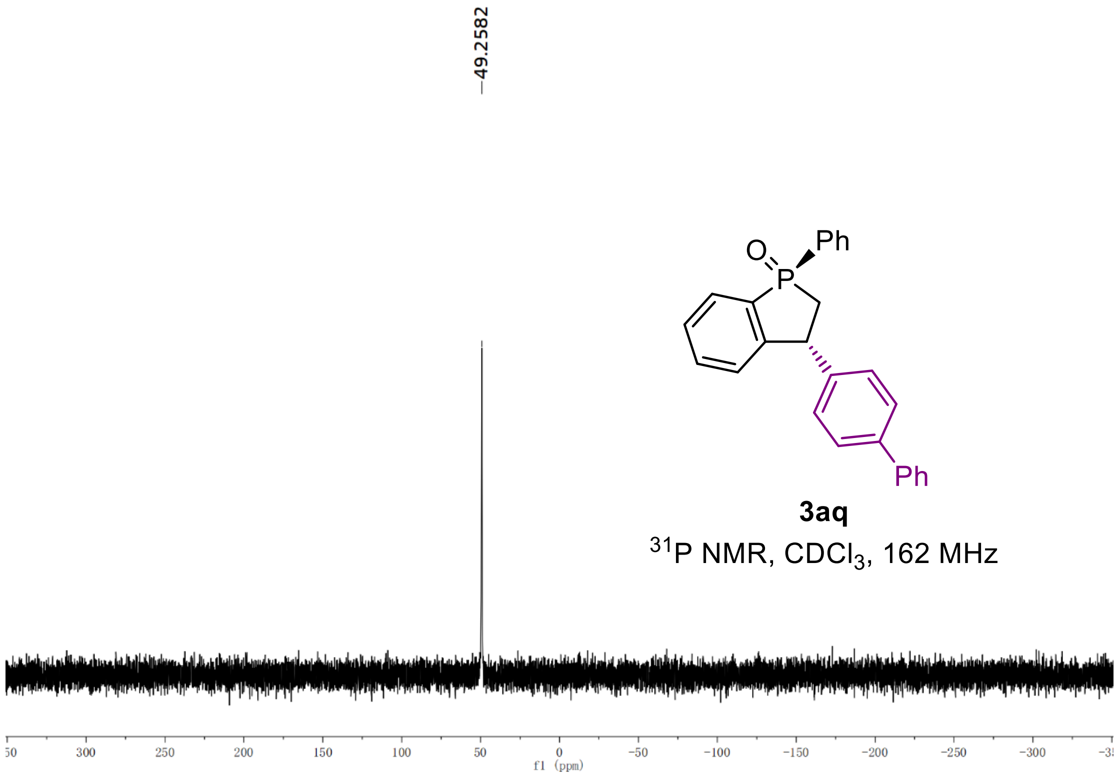

Supplementary Figure 68. ^{31}P NMR of the 3aq (162 MHz, $CDCl_3$)

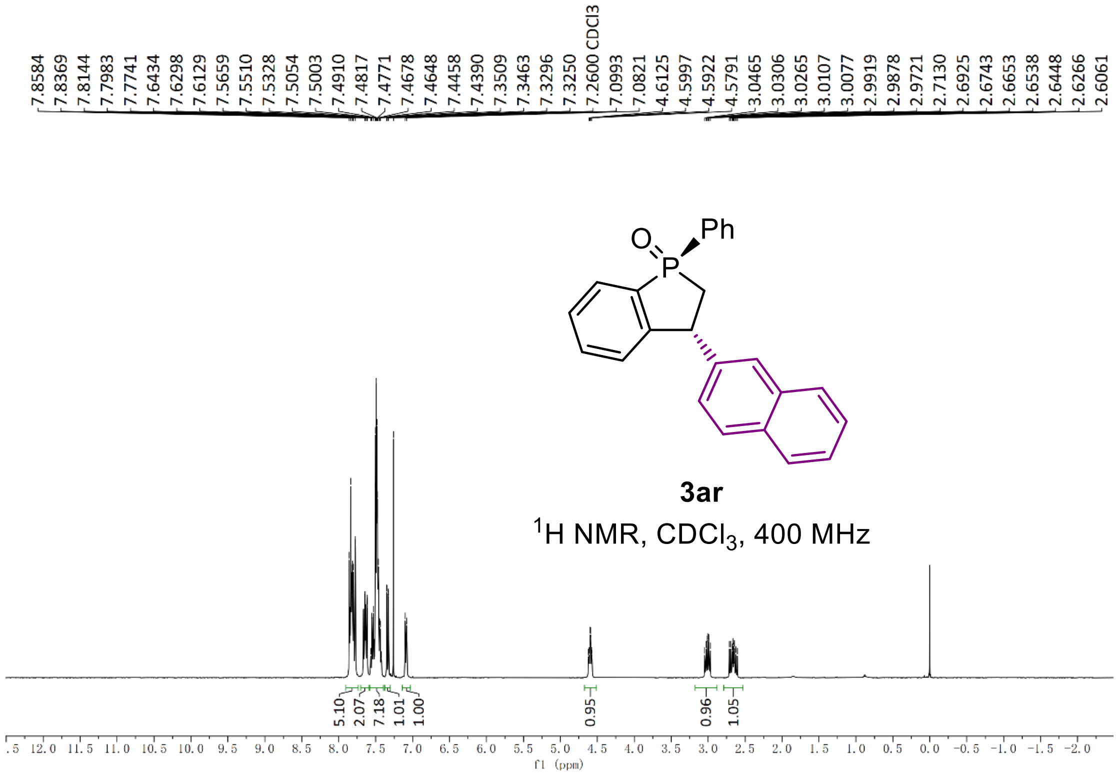

Supplementary Figure 69. ^{1}H NMR of the 3ar (400 MHz, $CDCl_3$)

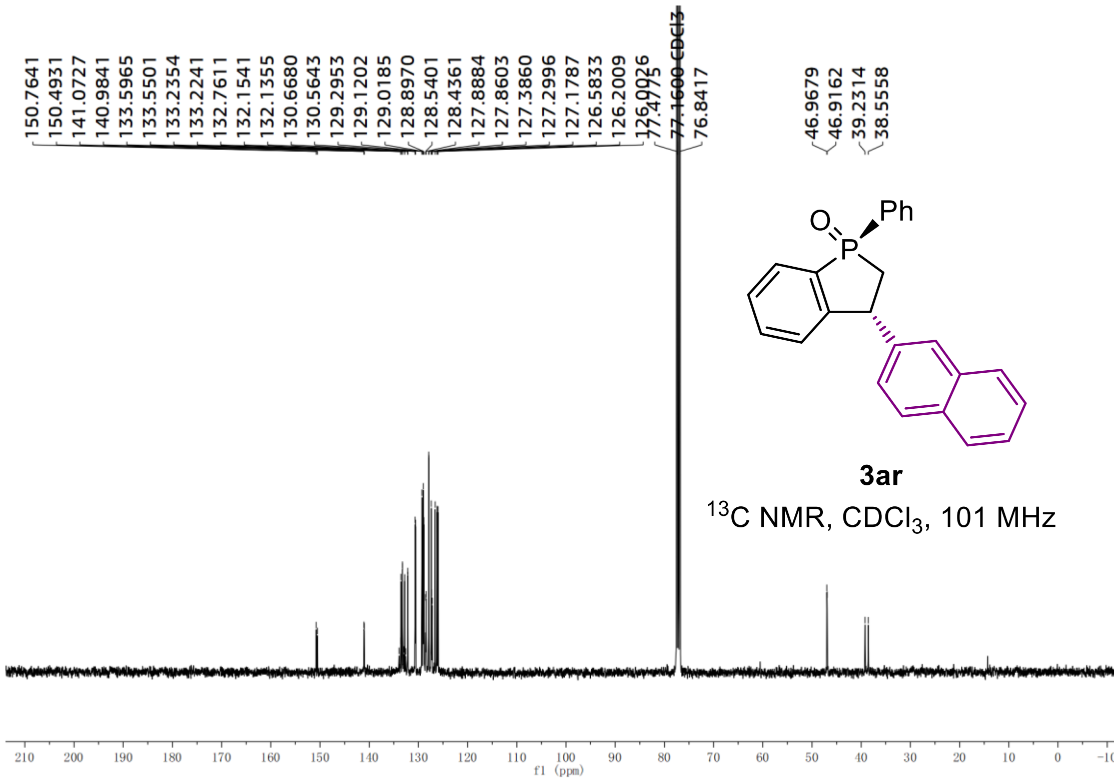

Supplementary Figure 70. ^{13}C NMR of the 3ar (101 MHz, CDCl$_3$)

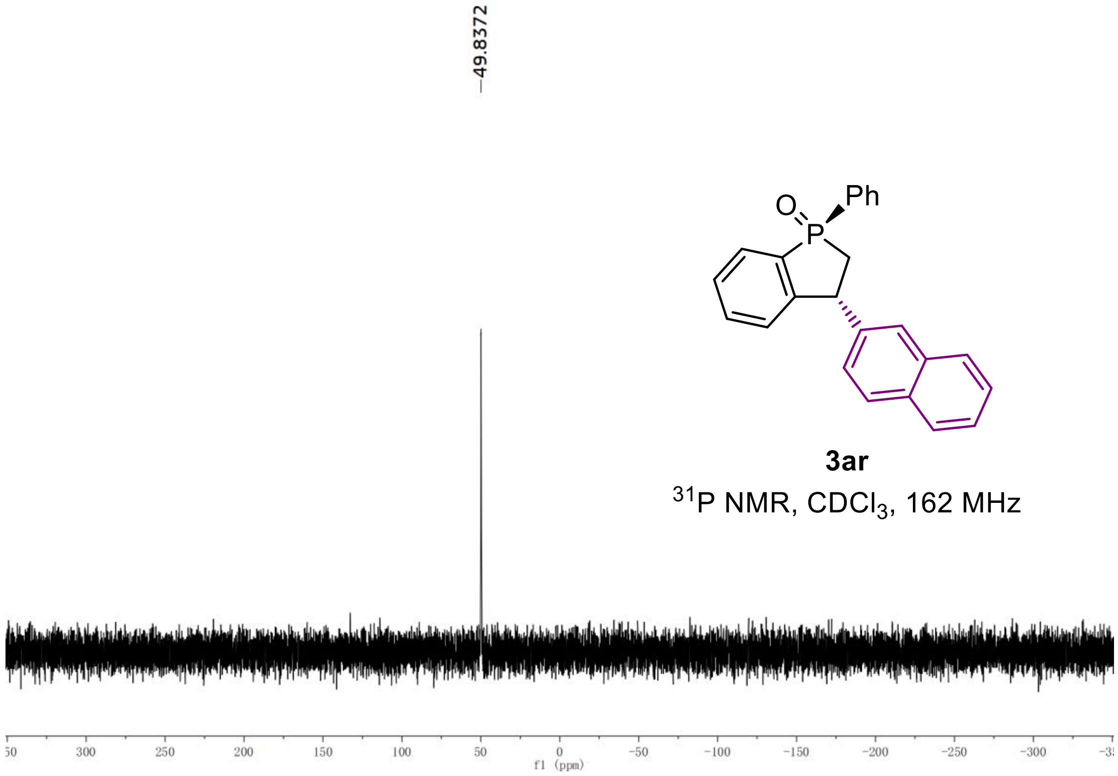

Supplementary Figure 71. ^{31}P NMR of the 3ar (162 MHz, CDCl$_3$)

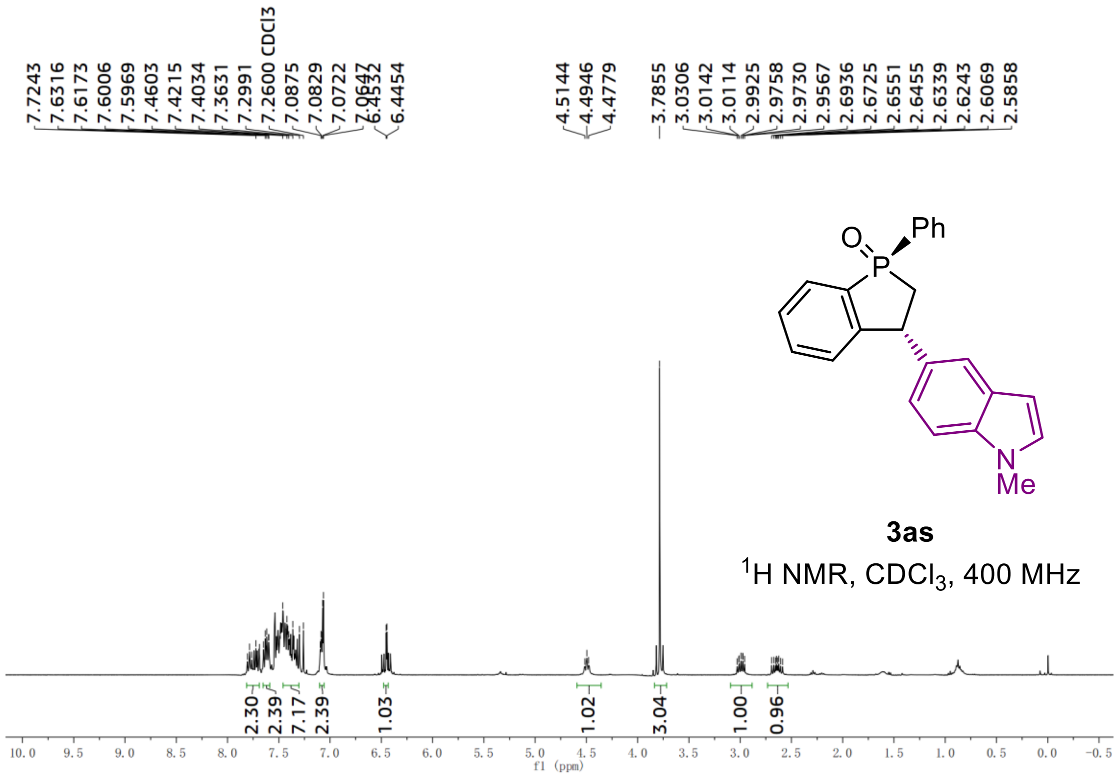

Supplementary Figure 72. ^{1}**H NMR of the 3as (400 MHz, $CDCl_3$)**

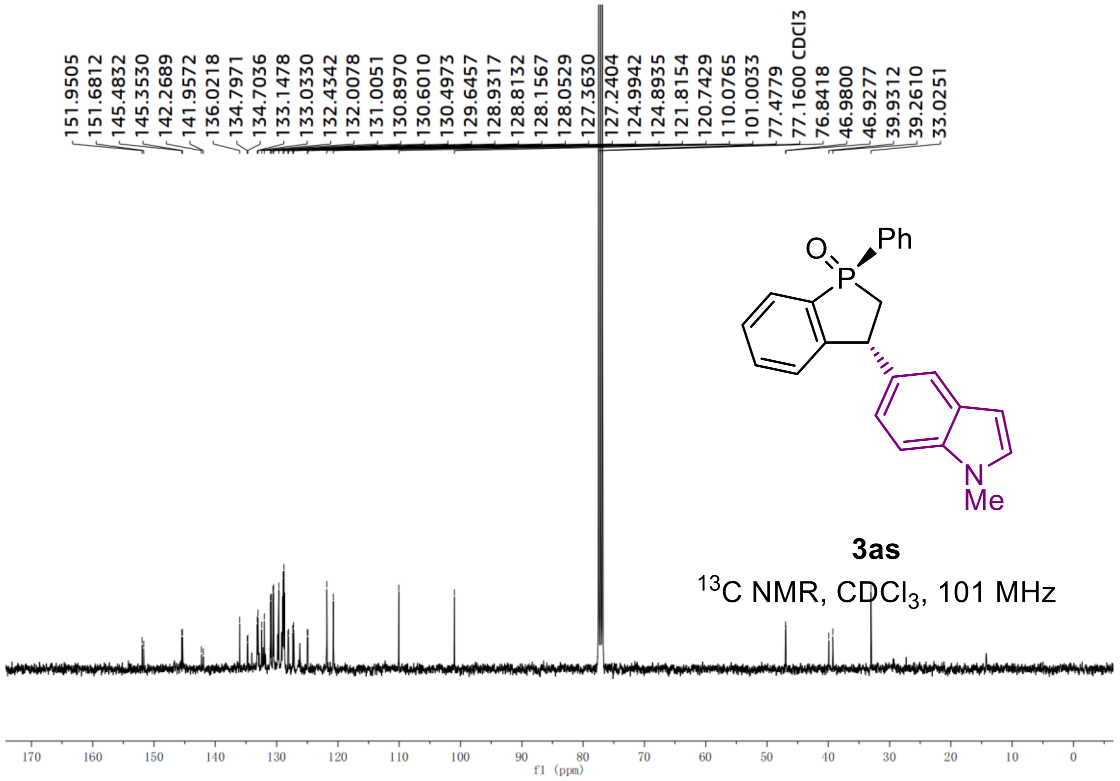

Supplementary Figure 73. ^{13}**C NMR of the 3as (101 MHz, $CDCl_3$)**

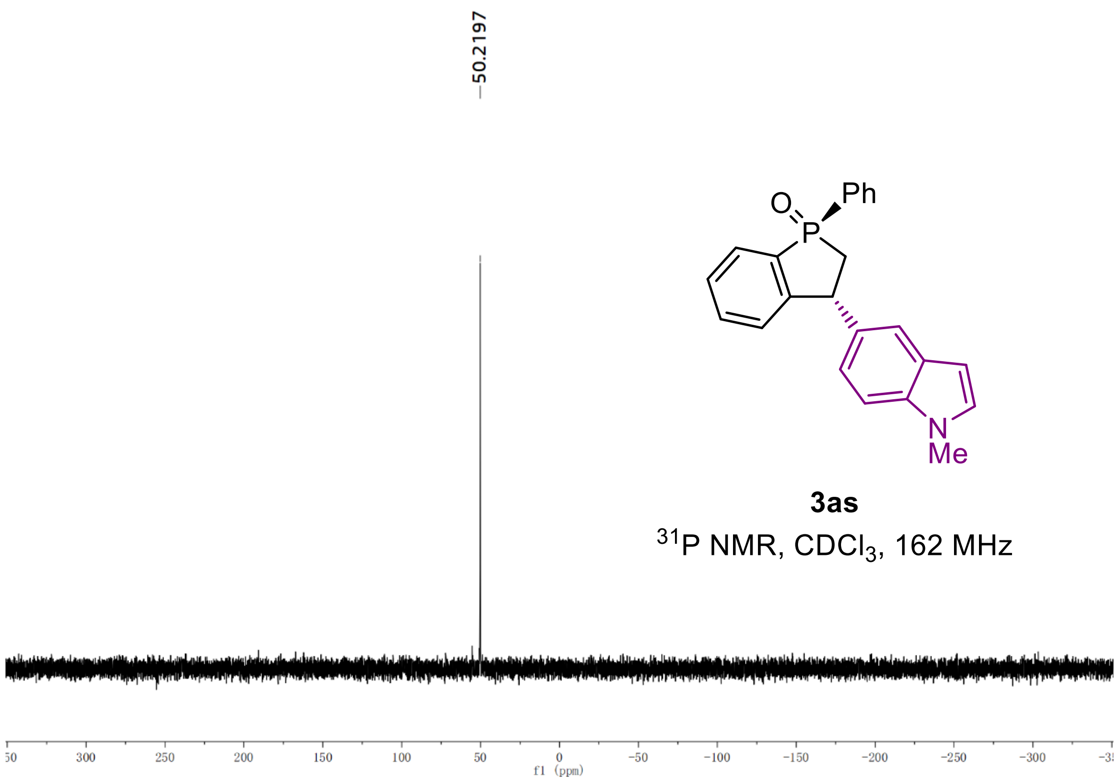

Supplementary Figure 74. ^{31}P NMR of the 3as (162 MHz, CDCl$_3$)

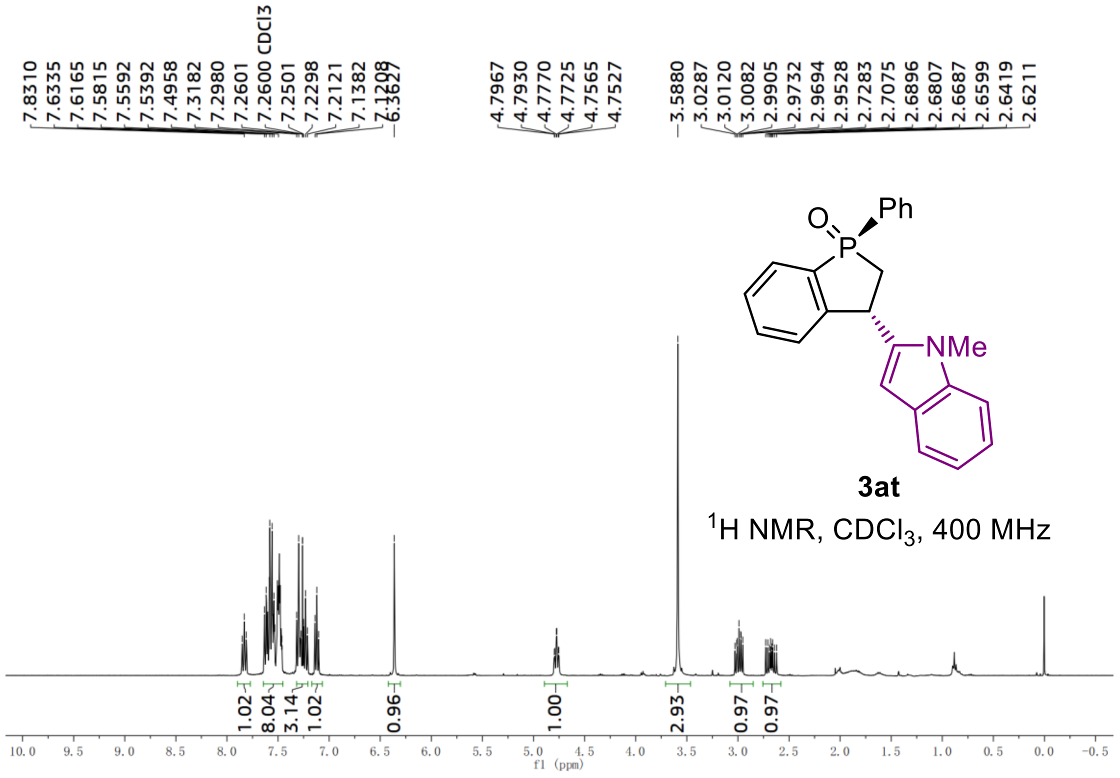

Supplementary Figure 75. ^{1}H NMR of the 3at (400 MHz, CDCl$_3$)

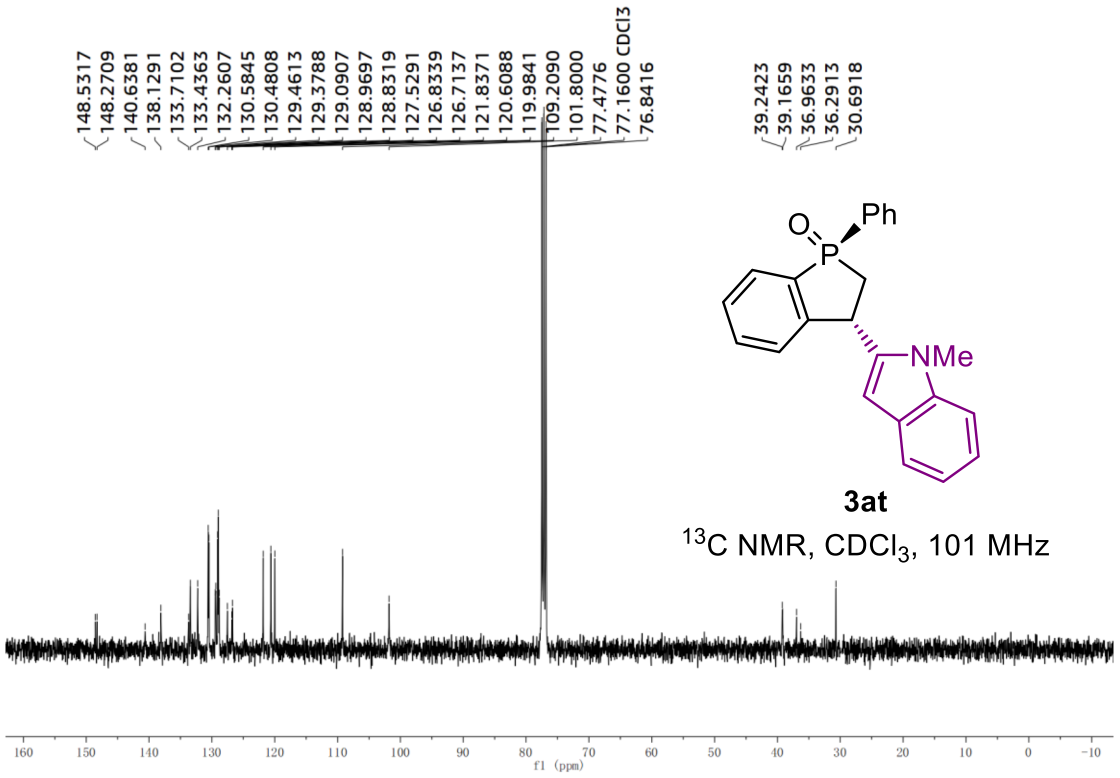

Supplementary Figure 76. ^{13}C NMR of the 3at (101 MHz, $CDCl_3$)

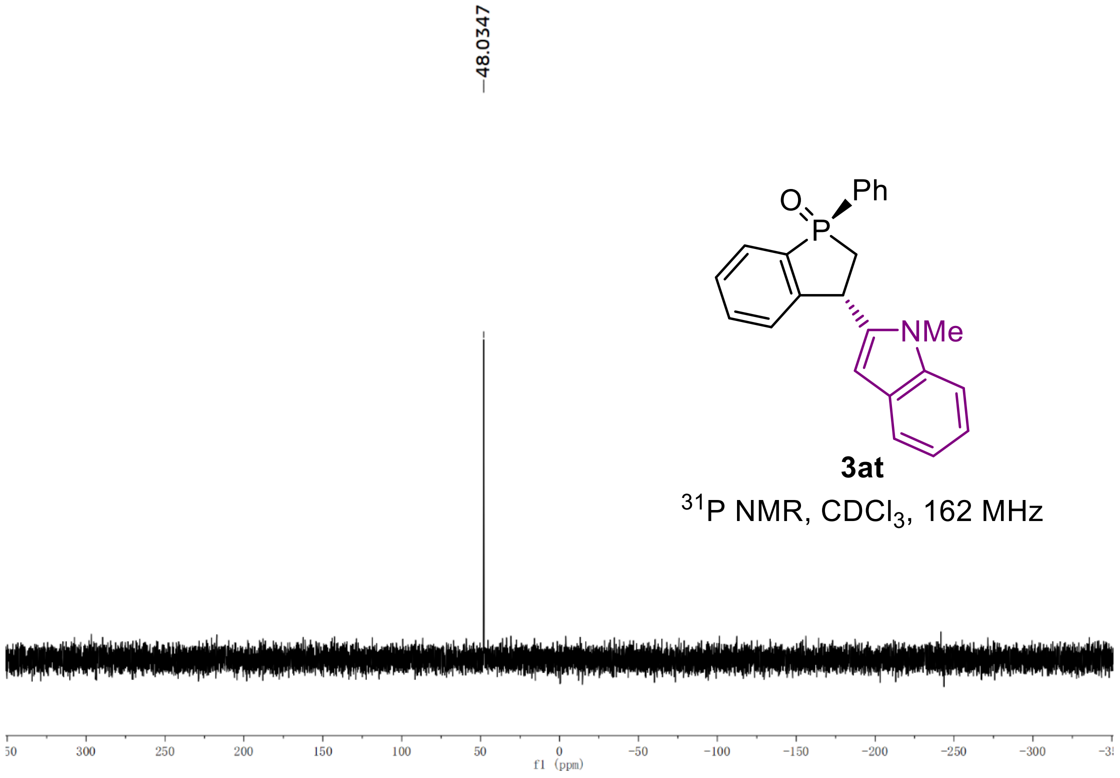

Supplementary Figure 77. ^{31}P NMR of the 3at (162 MHz, $CDCl_3$)

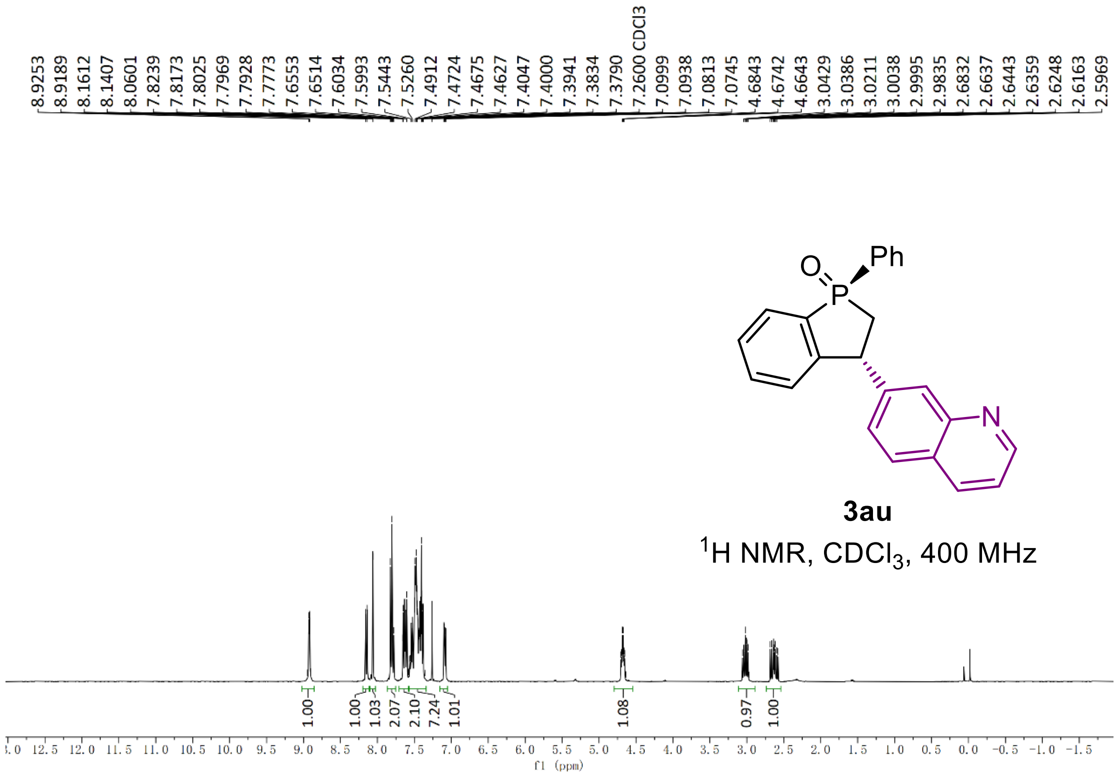

Supplementary Figure 78. ^{1}H NMR of the 3au (400 MHz, $CDCl_3$)

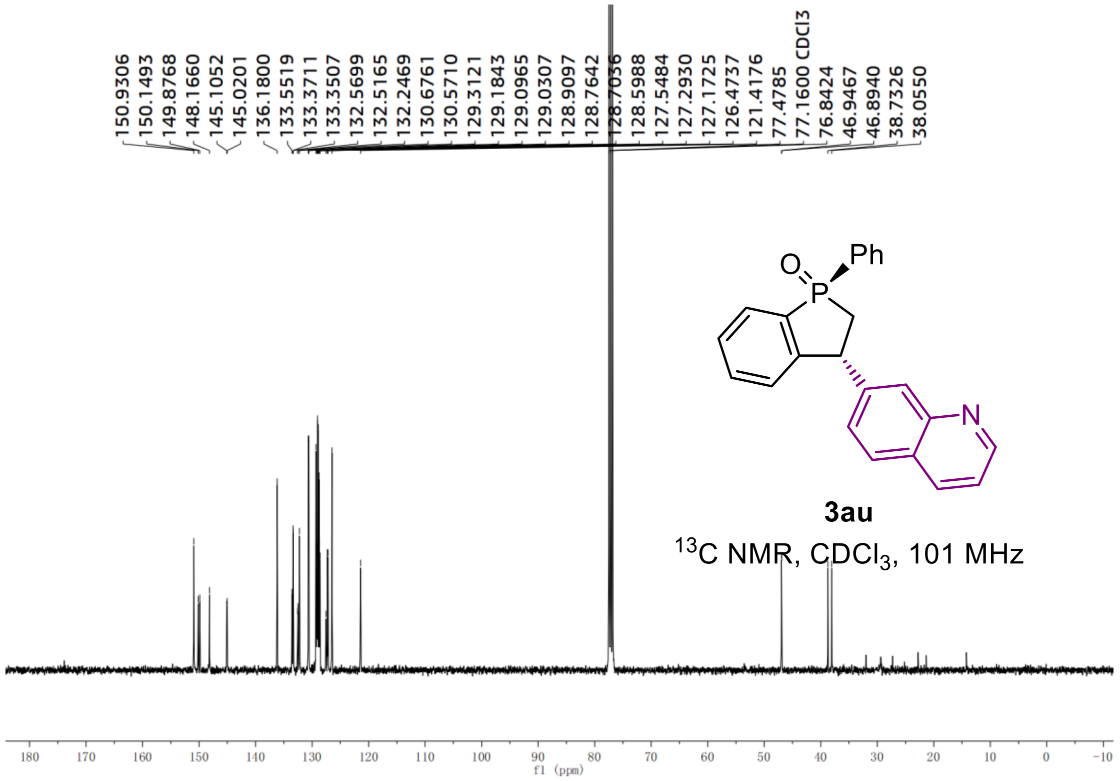

Supplementary Figure 79. ^{13}C NMR of the 3au (101 MHz, $CDCl_3$)

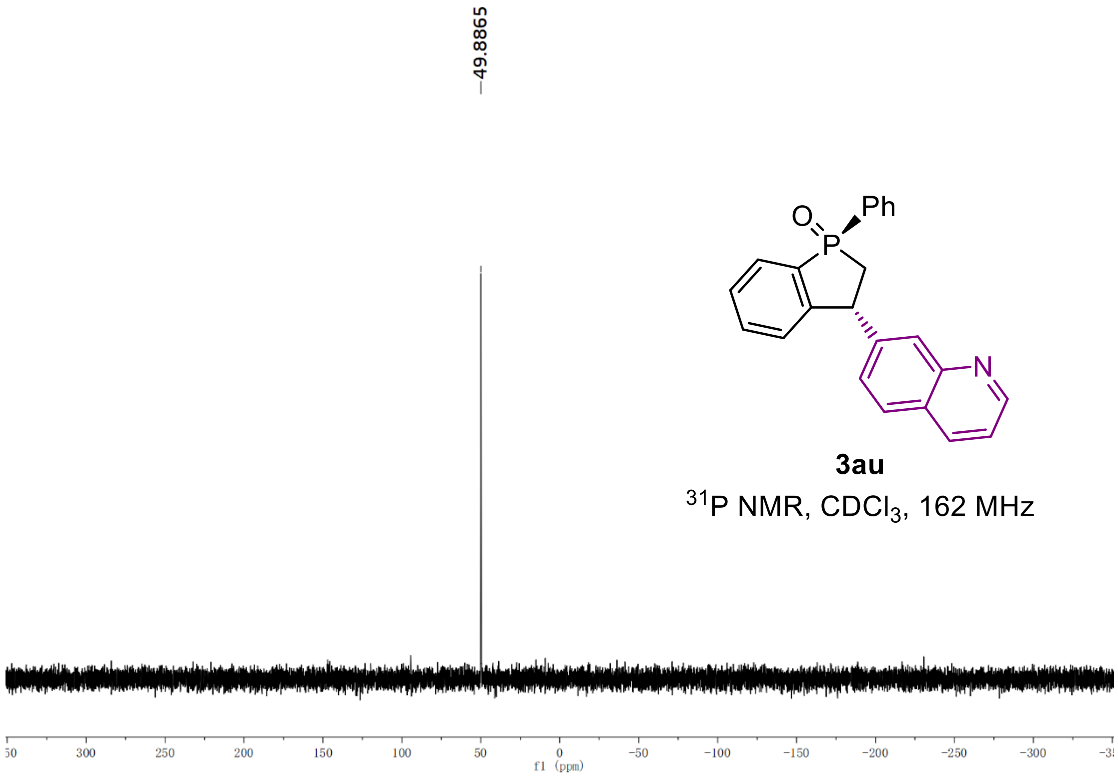

Supplementary Figure 80. ^{31}P NMR of the 3au (162 MHz, CDCl$_3$)

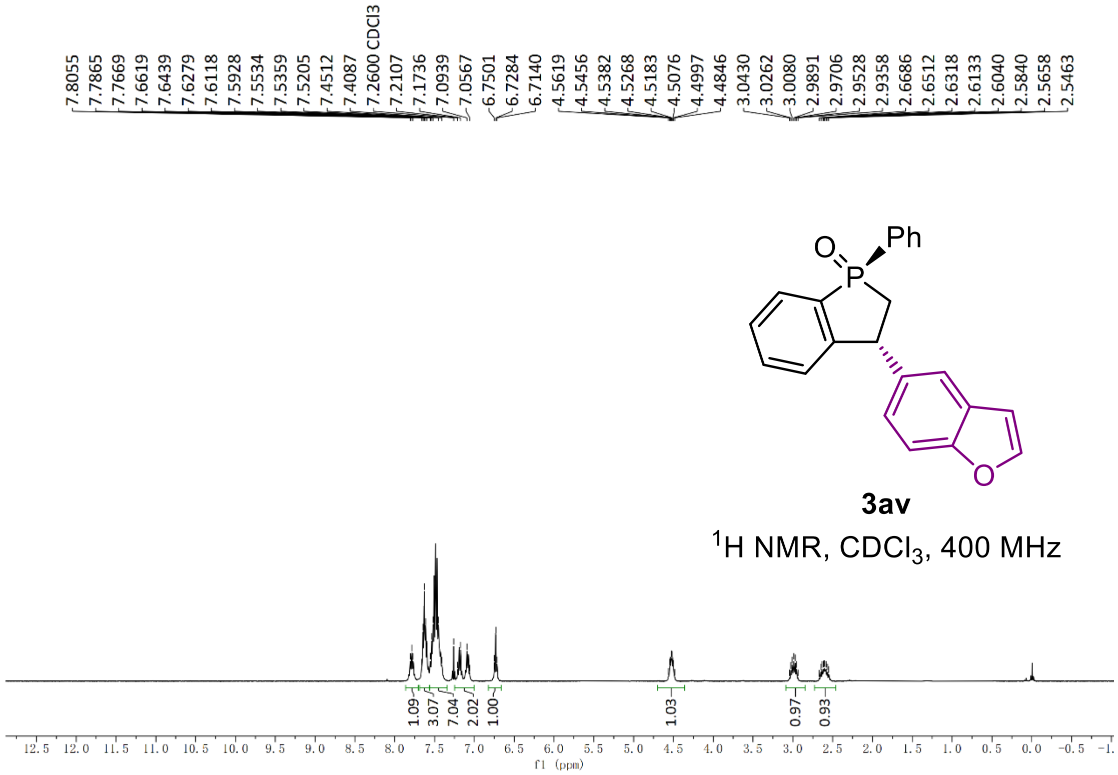

Supplementary Figure 81. ^{1}H NMR of the 3av (400 MHz, CDCl$_3$)

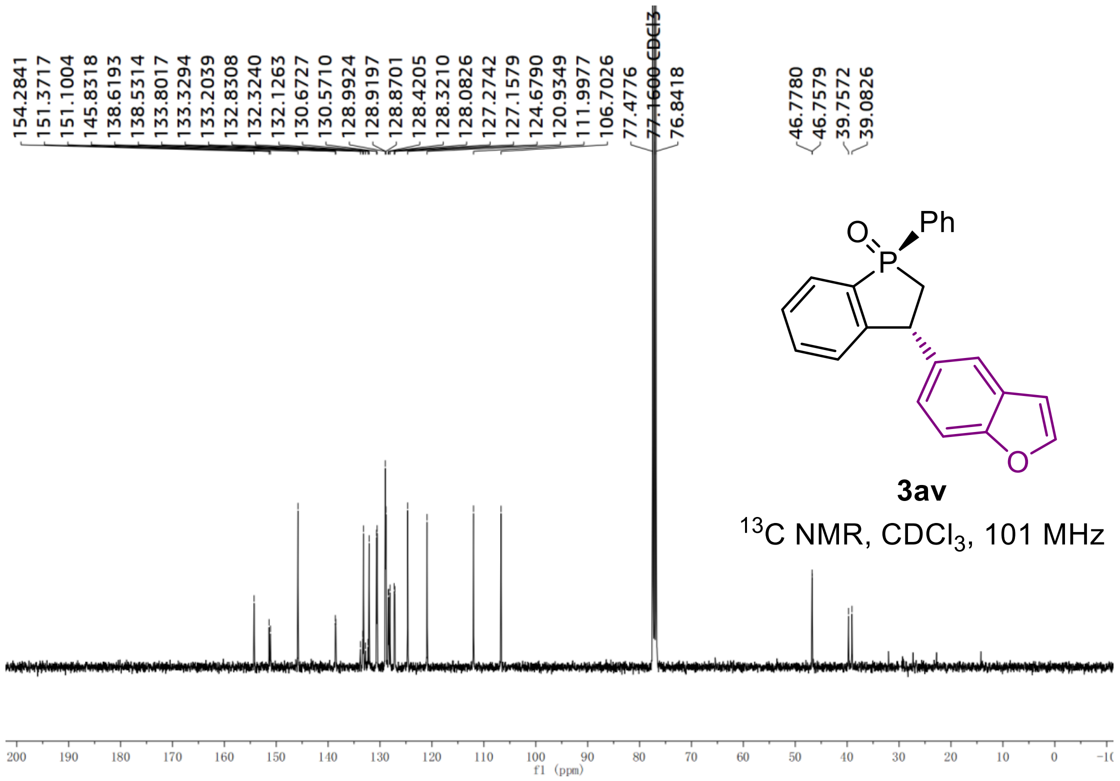

Supplementary Figure 82. ^{13}C NMR of the 3av (101 MHz, $CDCl_3$)

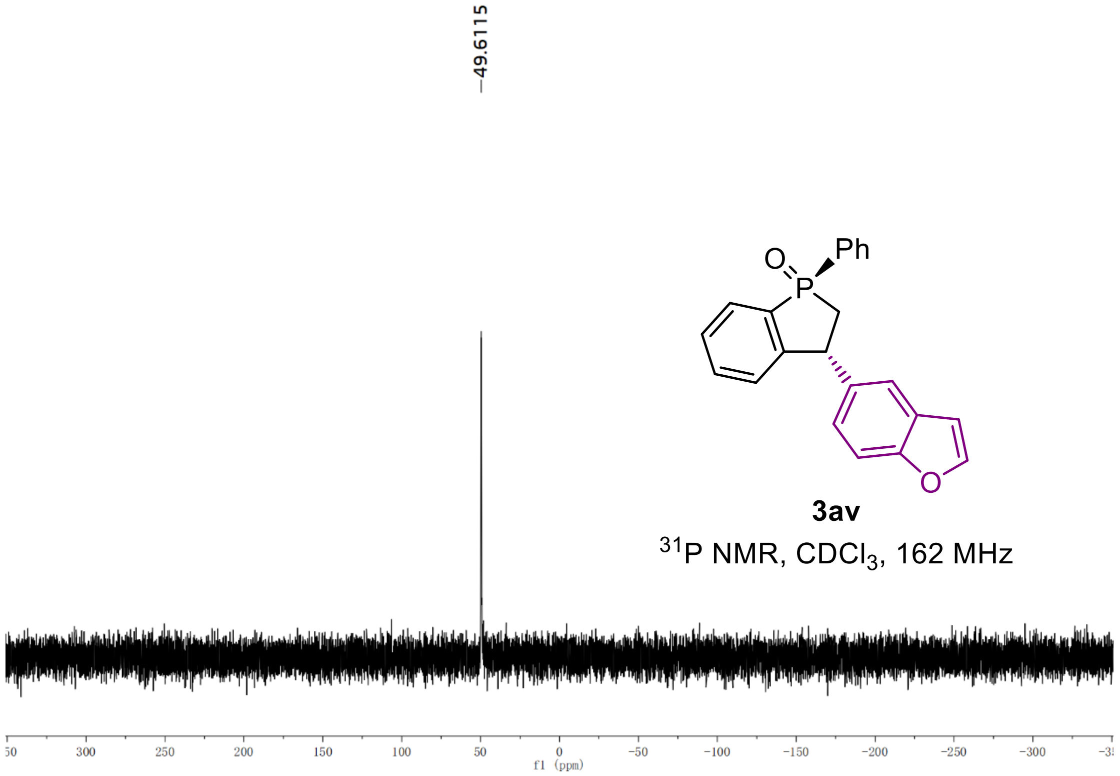

Supplementary Figure 83. ^{31}P NMR of the 3av (162 MHz, $CDCl_3$)

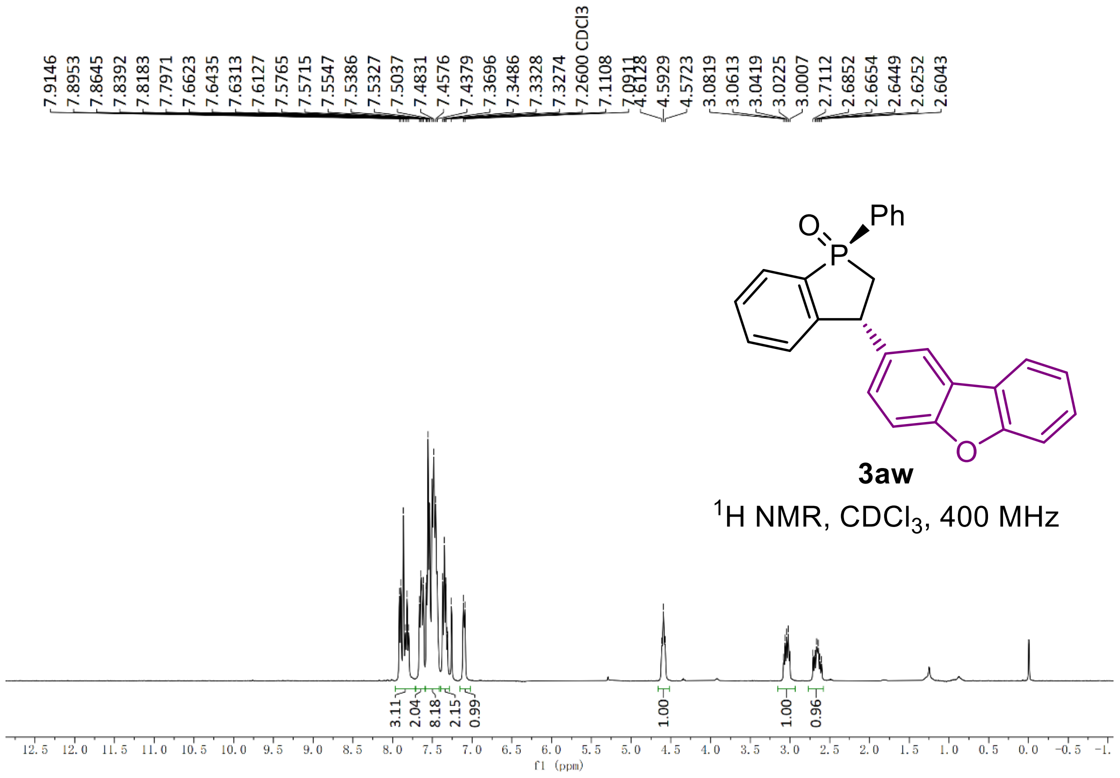

Supplementary Figure 84. ^{1}H NMR of the 3aw (400 MHz, $CDCl_3$)

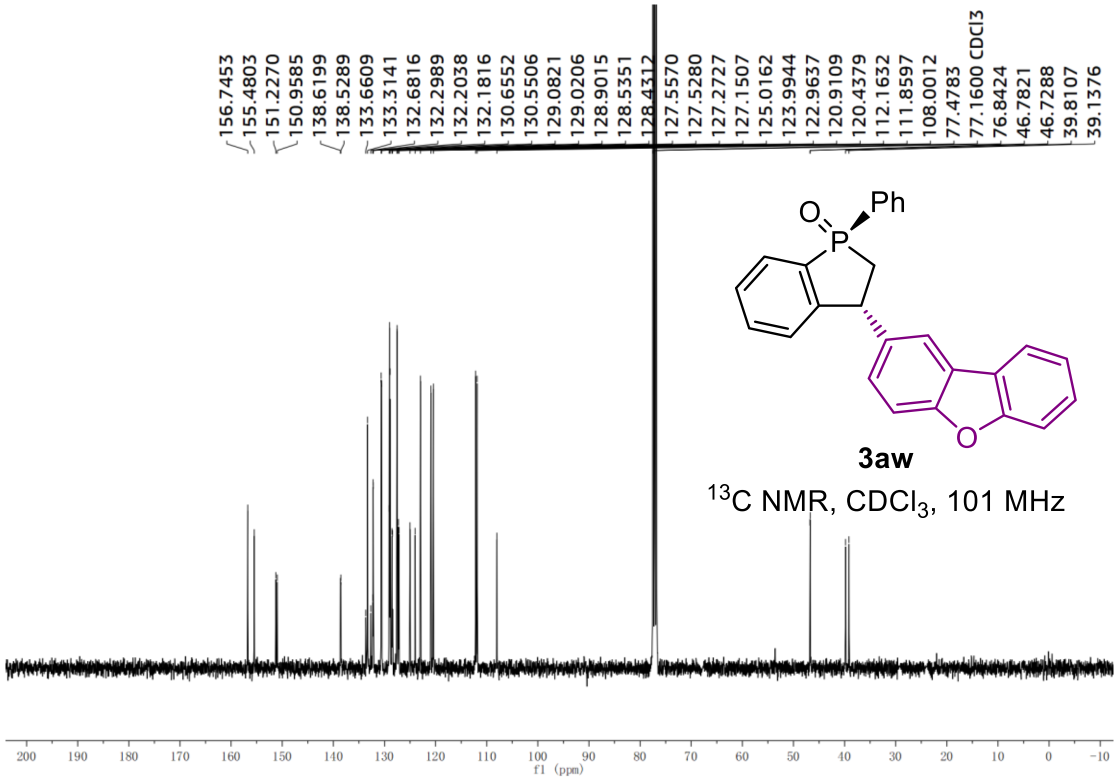

Supplementary Figure 85. ^{13}C NMR of the 3aw (101 MHz, $CDCl_3$)

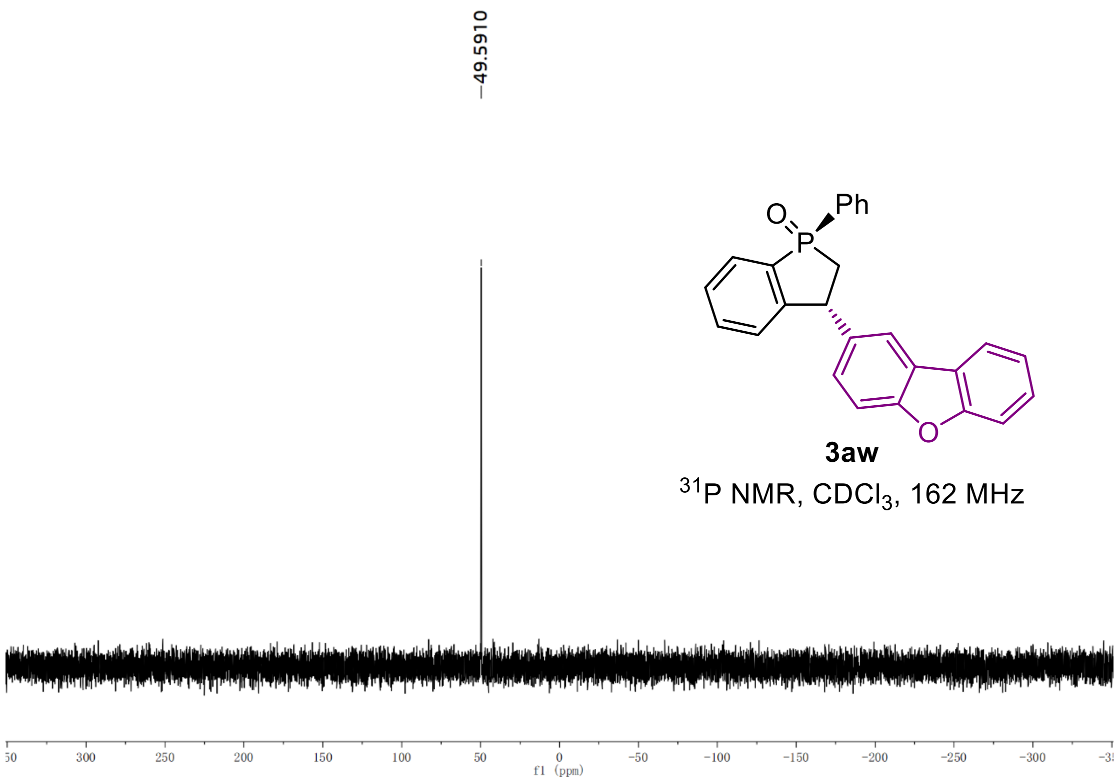

Supplementary Figure 86. ^{31}P NMR of the 3aw (162 MHz, CDCl$_3$)

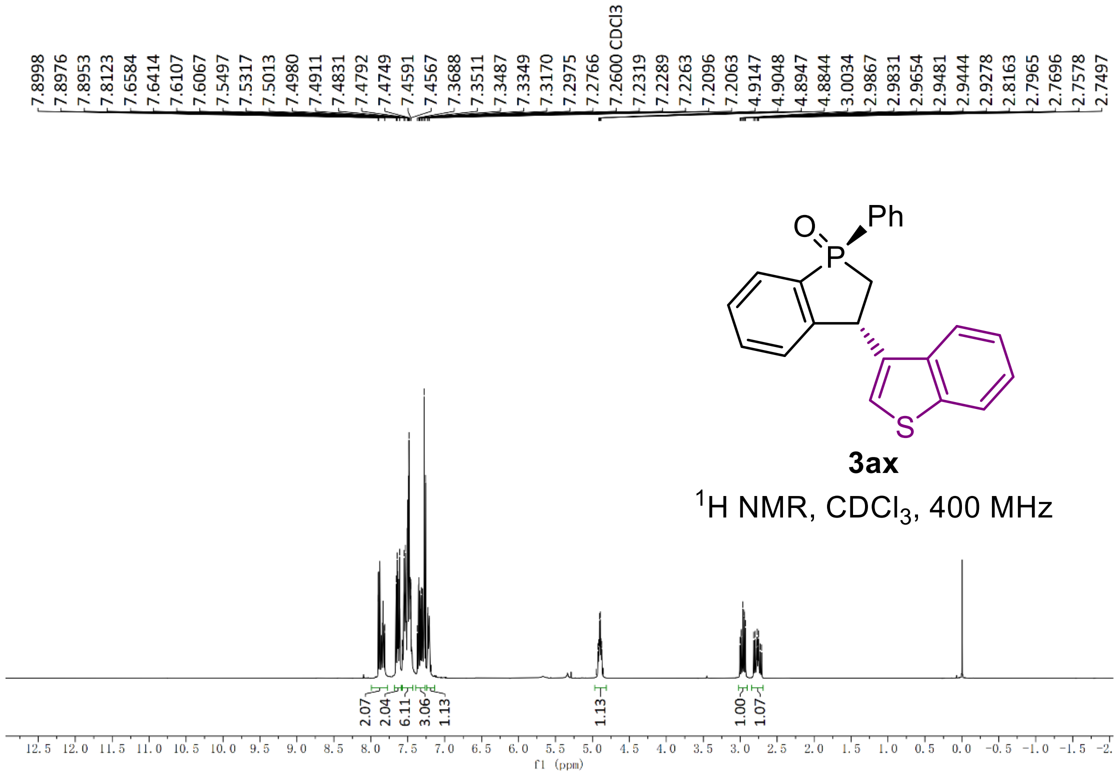

Supplementary Figure 87. ^{1}H NMR of the 3ax (400 MHz, CDCl$_3$)

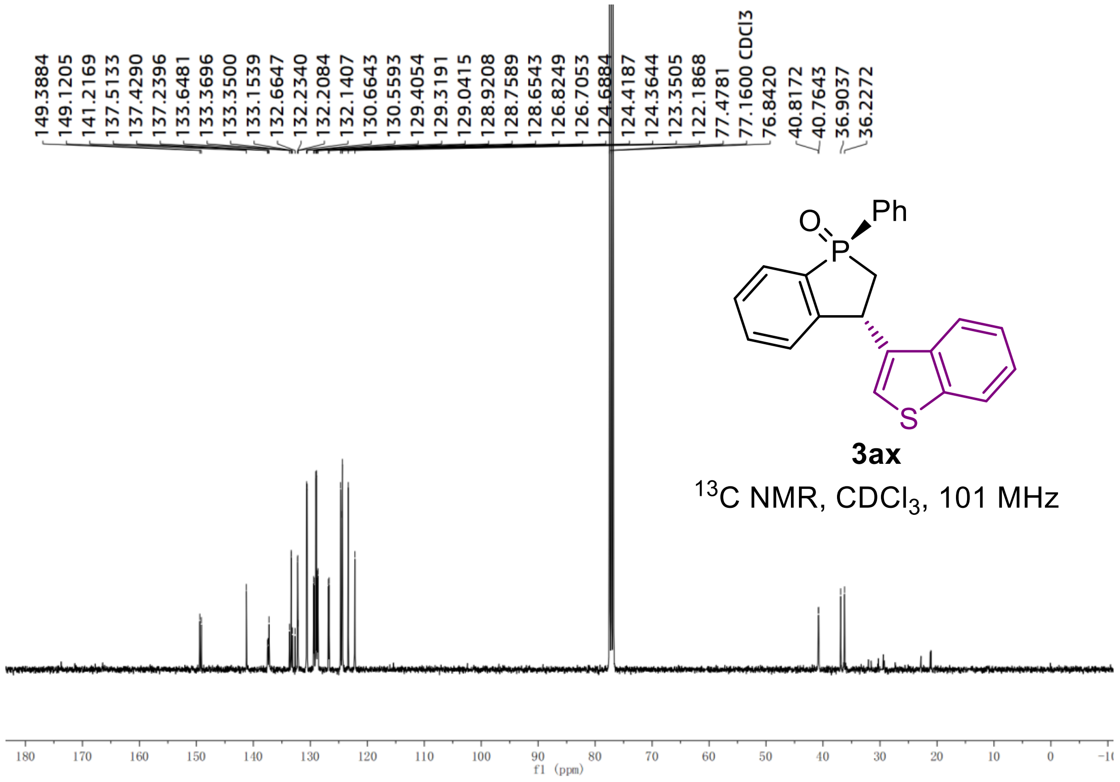

Supplementary Figure 88. ^{13}C NMR of the 3ax (101 MHz, $CDCl_3$)

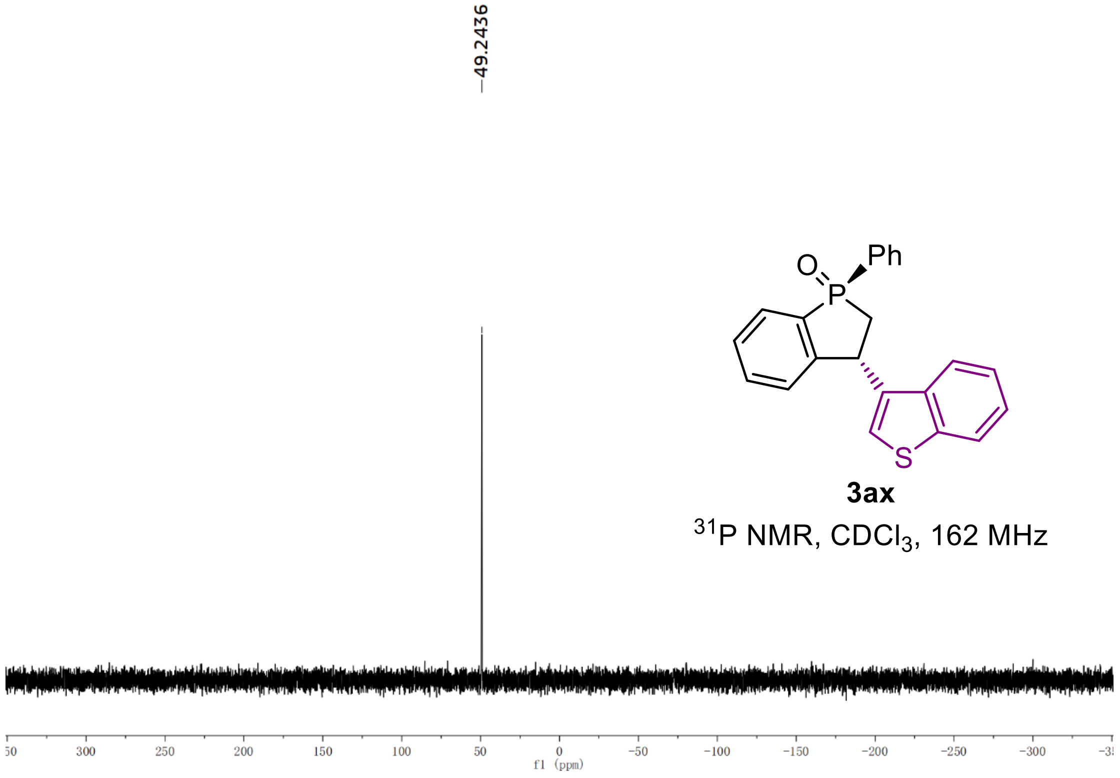

Supplementary Figure 89. ^{31}P NMR of the 3ax (162 MHz, $CDCl_3$)

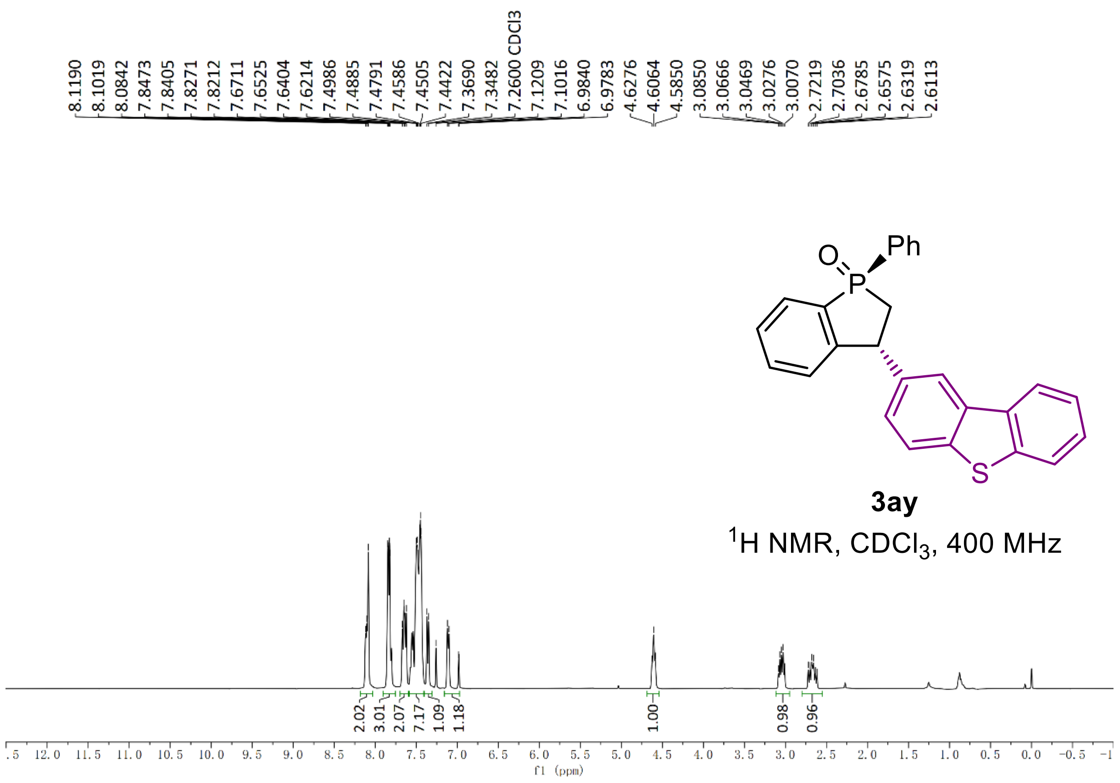

Supplementary Figure 90. ^{1}**H NMR of the 3ay (400 MHz, CDCl$_3$)**

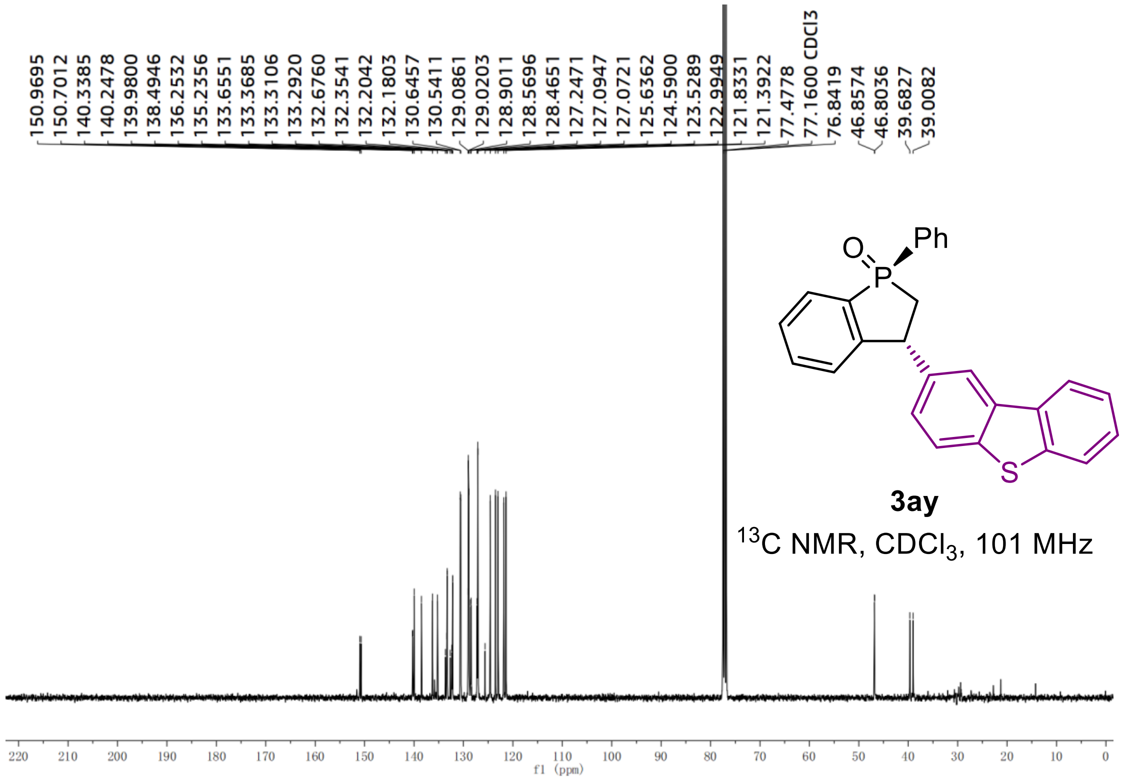

Supplementary Figure 91. ^{13}**C NMR of the 3ay (101 MHz, CDCl$_3$)**

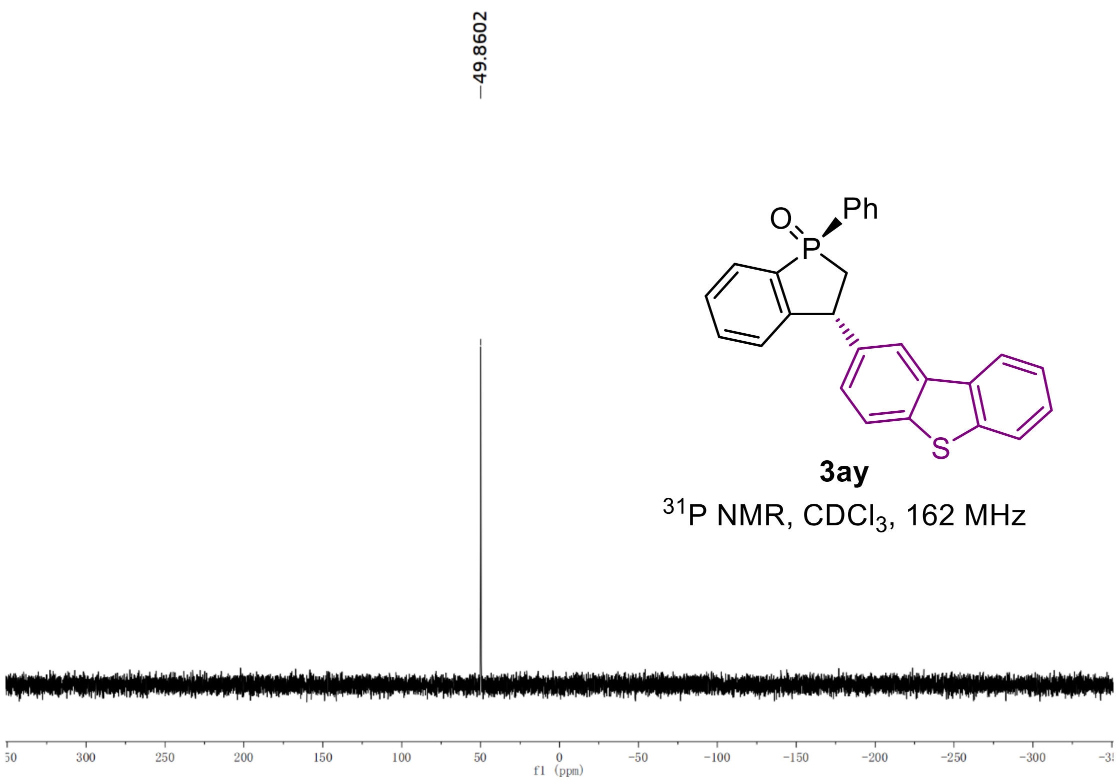

Supplementary Figure 92. ^{31}P NMR of the 3ay (162 MHz, $CDCl_3$)

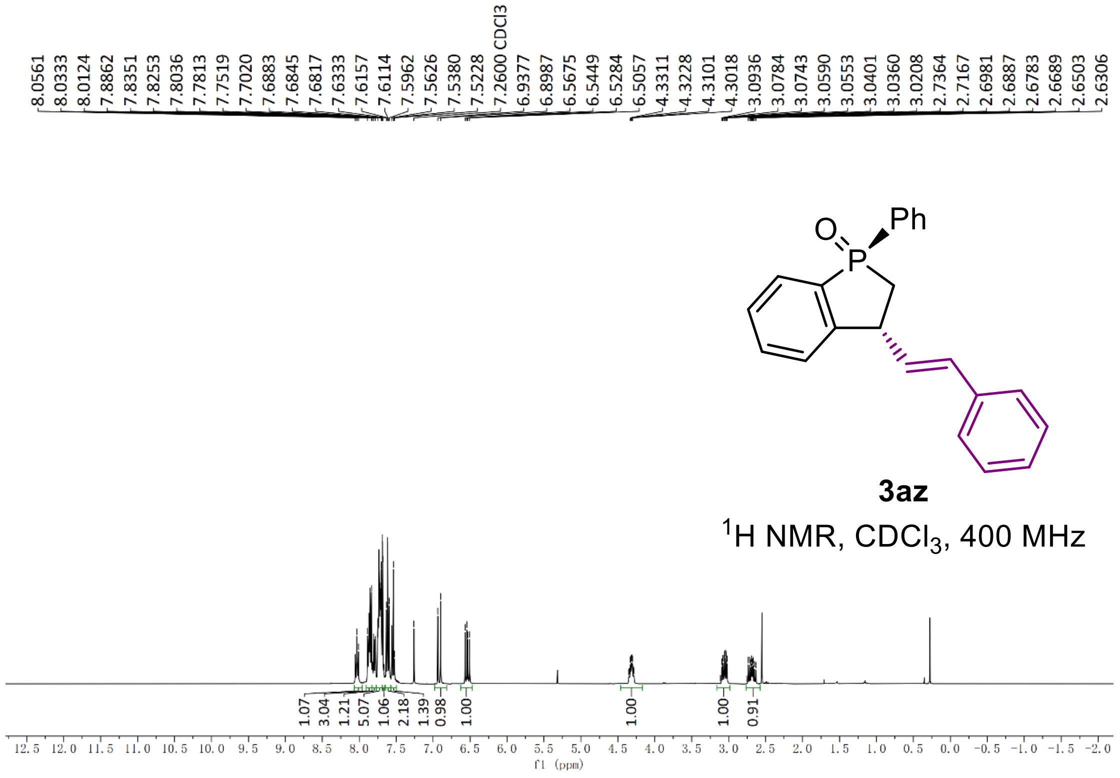

Supplementary Figure 93. ^{1}H NMR of the 3az (400 MHz, $CDCl_3$)

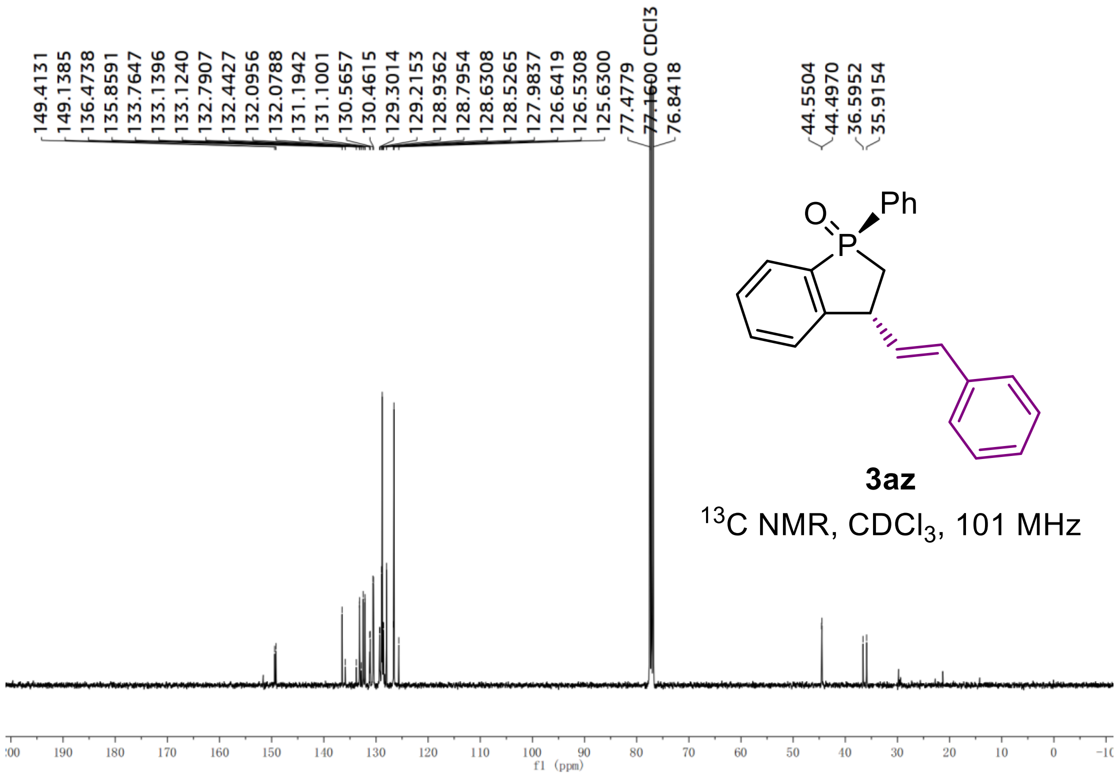

Supplementary Figure 94. ^{13}**C NMR of the 3az (101 MHz, CDCl$_3$)**

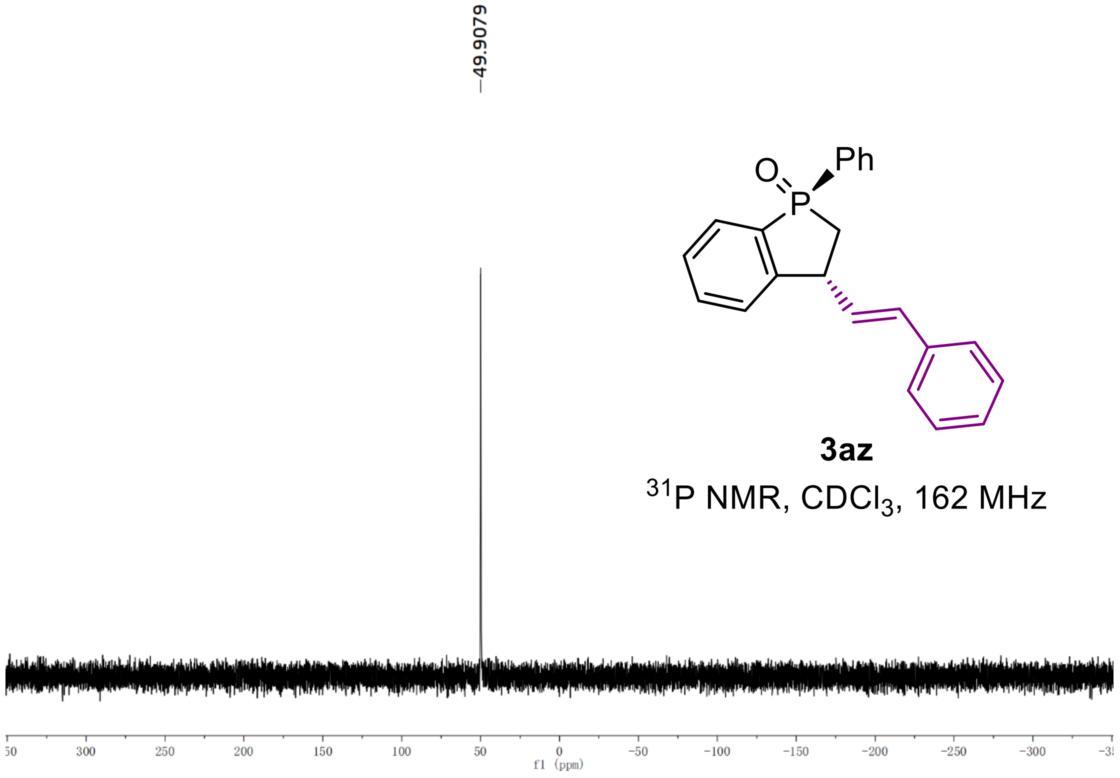

Supplementary Figure 95. ^{31}**P NMR of the 3az (162 MHz, CDCl$_3$)**

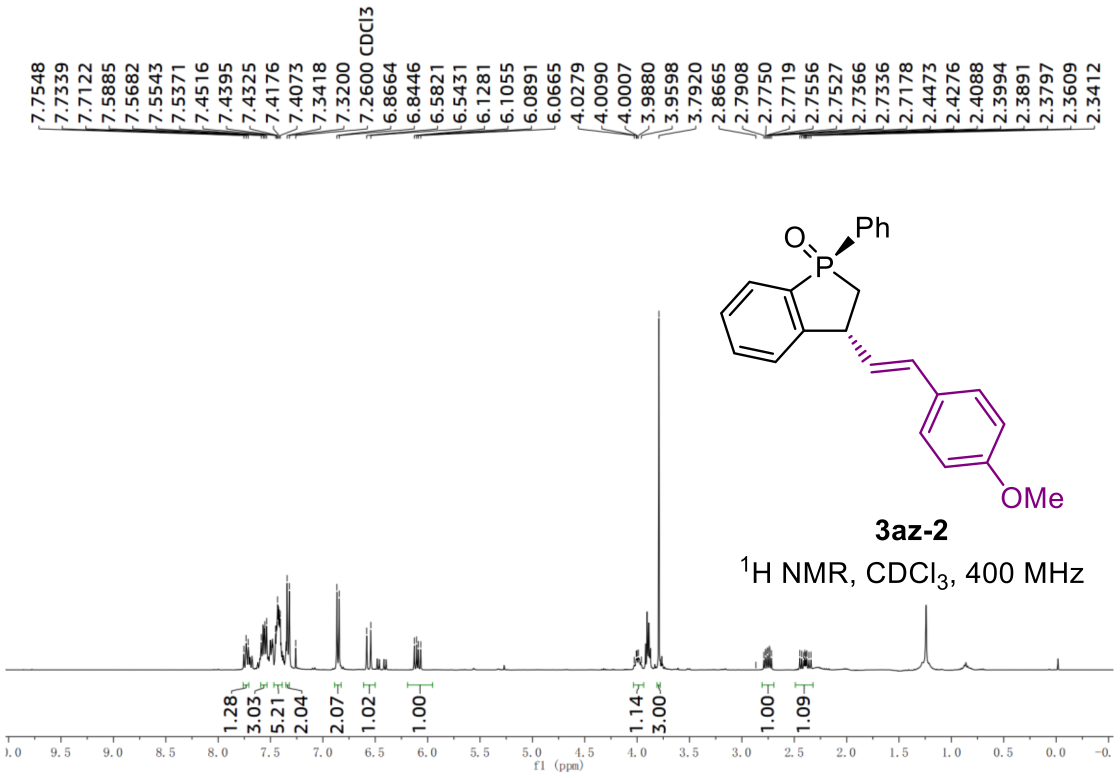

Supplementary Figure 96. ^{1}**H NMR of the 3az-2 (400 MHz, CDCl$_3$)**

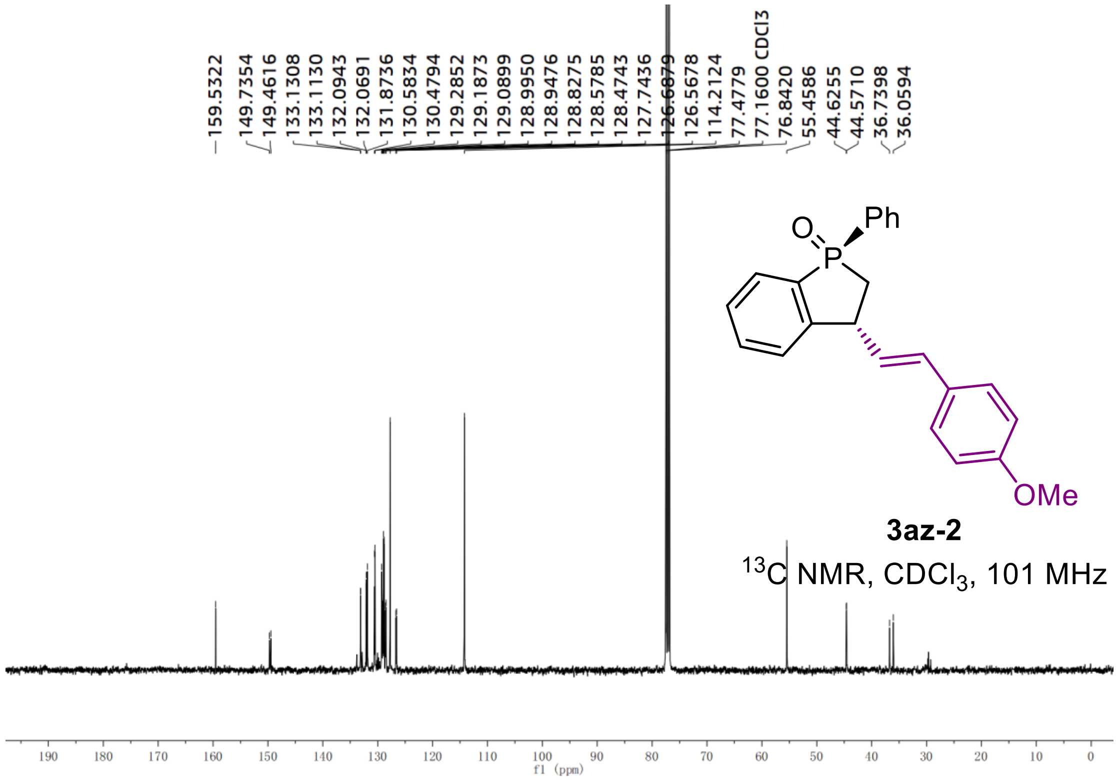

Supplementary Figure 97. ^{13}**C NMR of the 3az-2 (101 MHz, CDCl$_3$)**

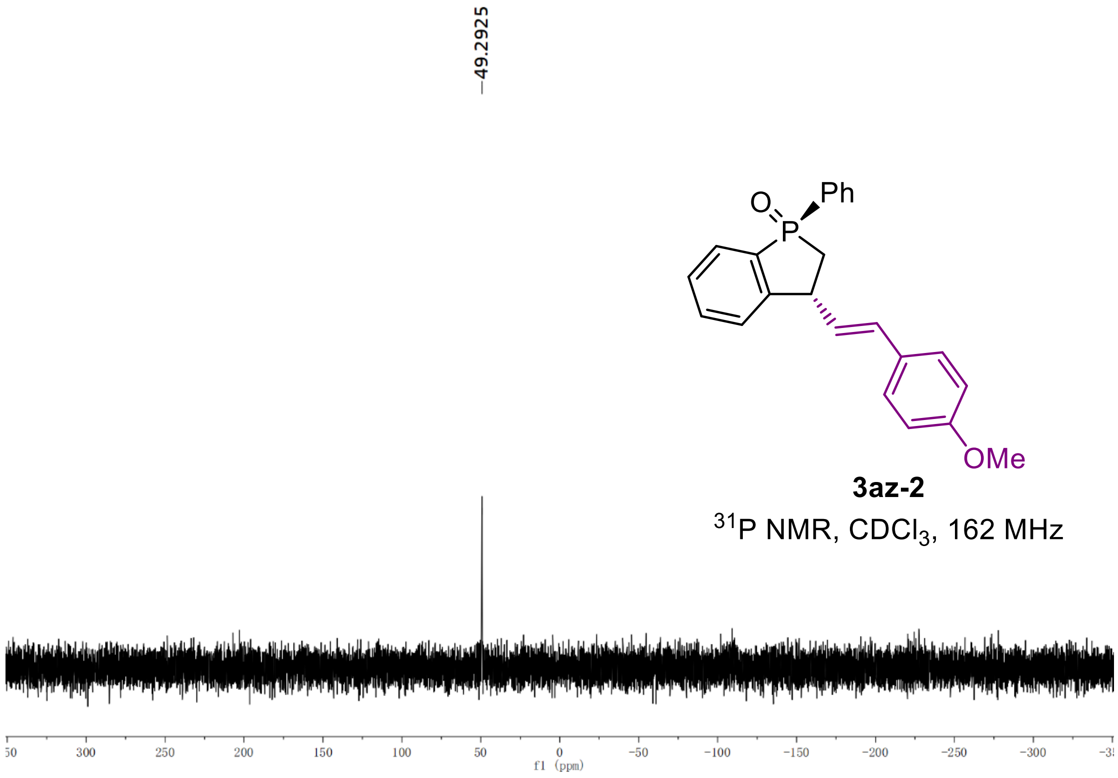

Supplementary Figure 98. ^{31}**P NMR of the 3az-2 (162 MHz, CDCl**$_3$**)**

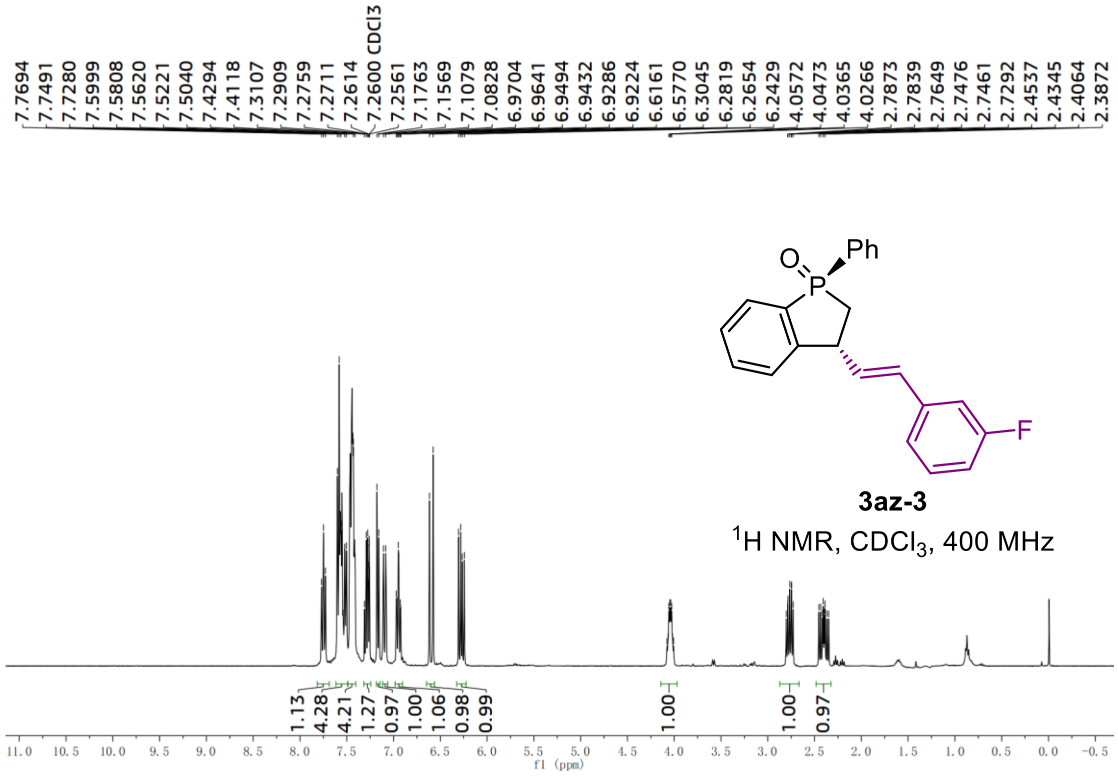

Supplementary Figure 99. ^{1}**H NMR of the 3az-3 (400 MHz, CDCl**$_3$**)**

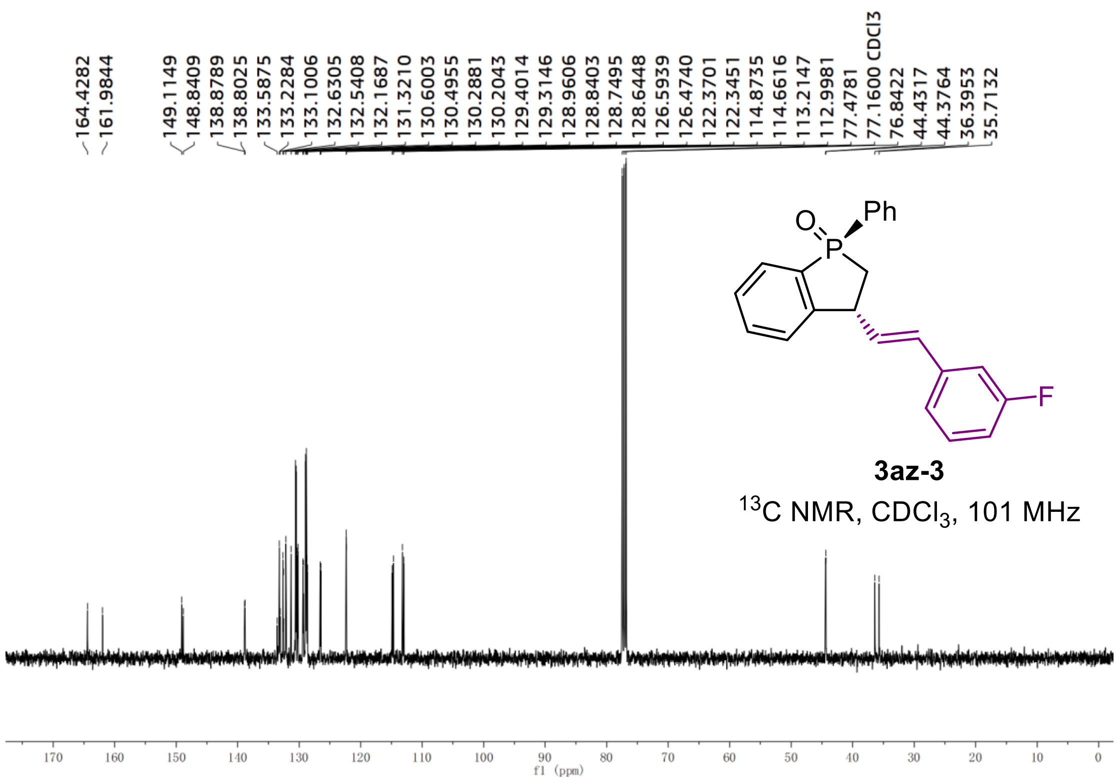

Supplementary Figure 100. ^{13}**C NMR of the 3az-3 (101 MHz, CDCl$_3$)**

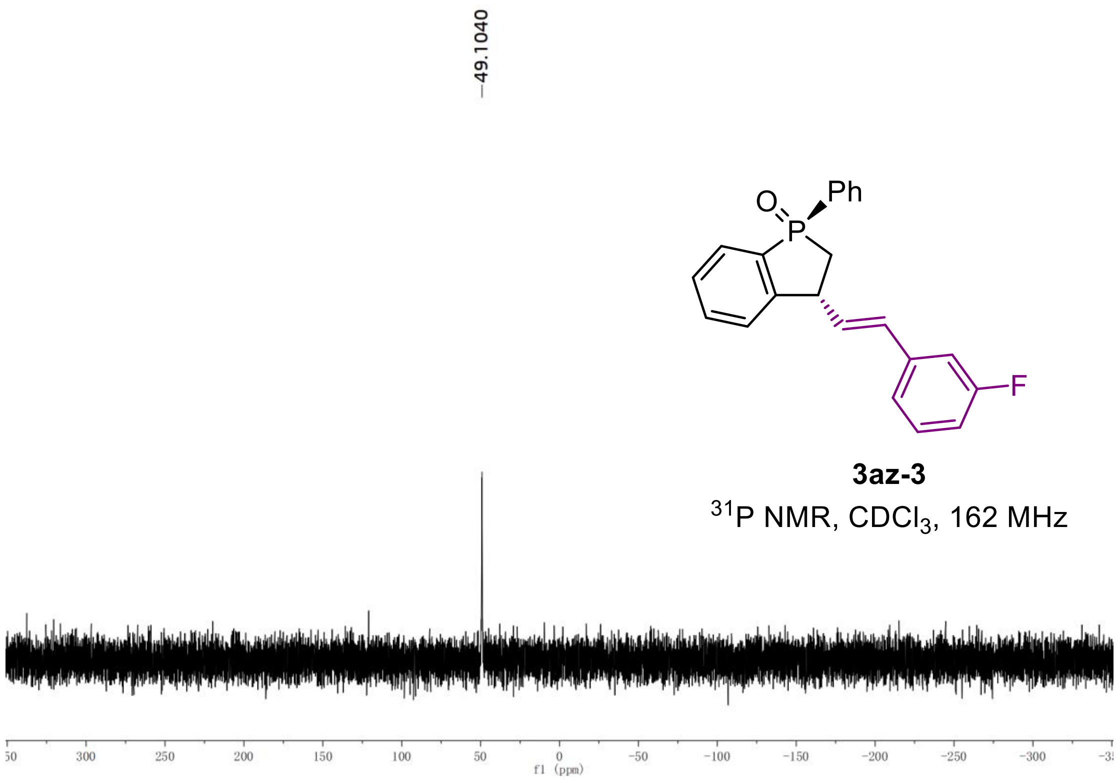

Supplementary Figure 101. ^{31}**P NMR of the 3az-3 (162 MHz, CDCl$_3$)**

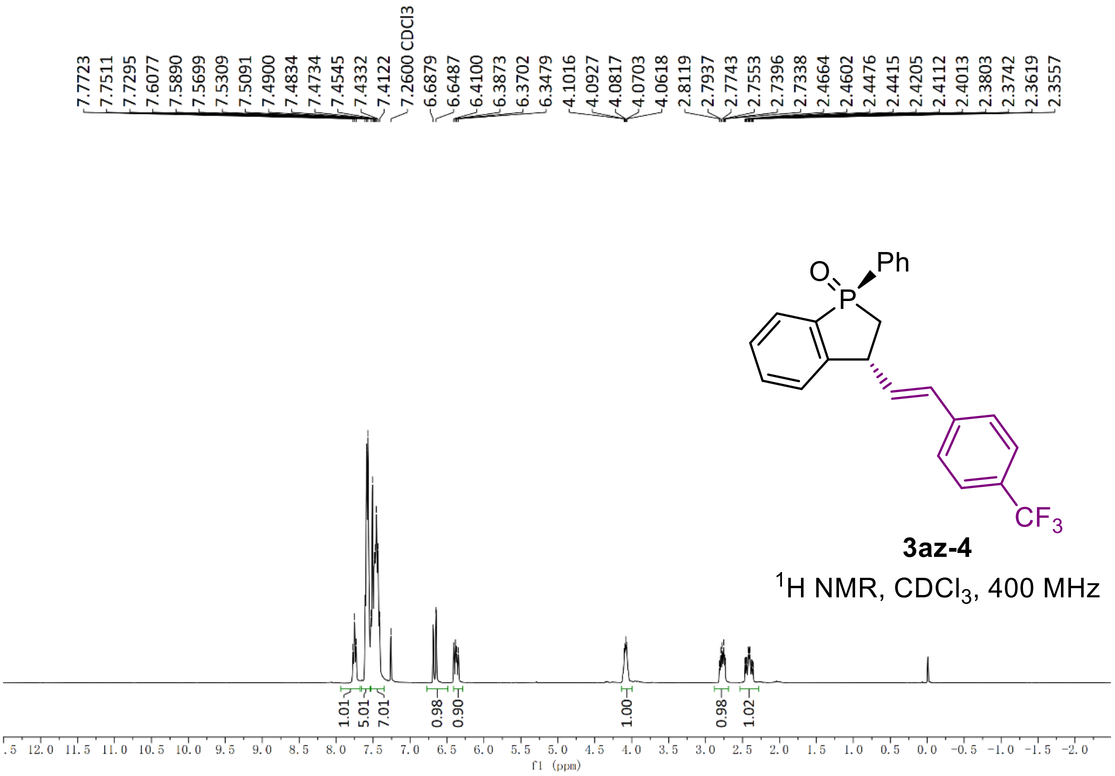

Supplementary Figure 102. ^{1}**H NMR of the 3az-4 (400 MHz, CDCl$_3$)**

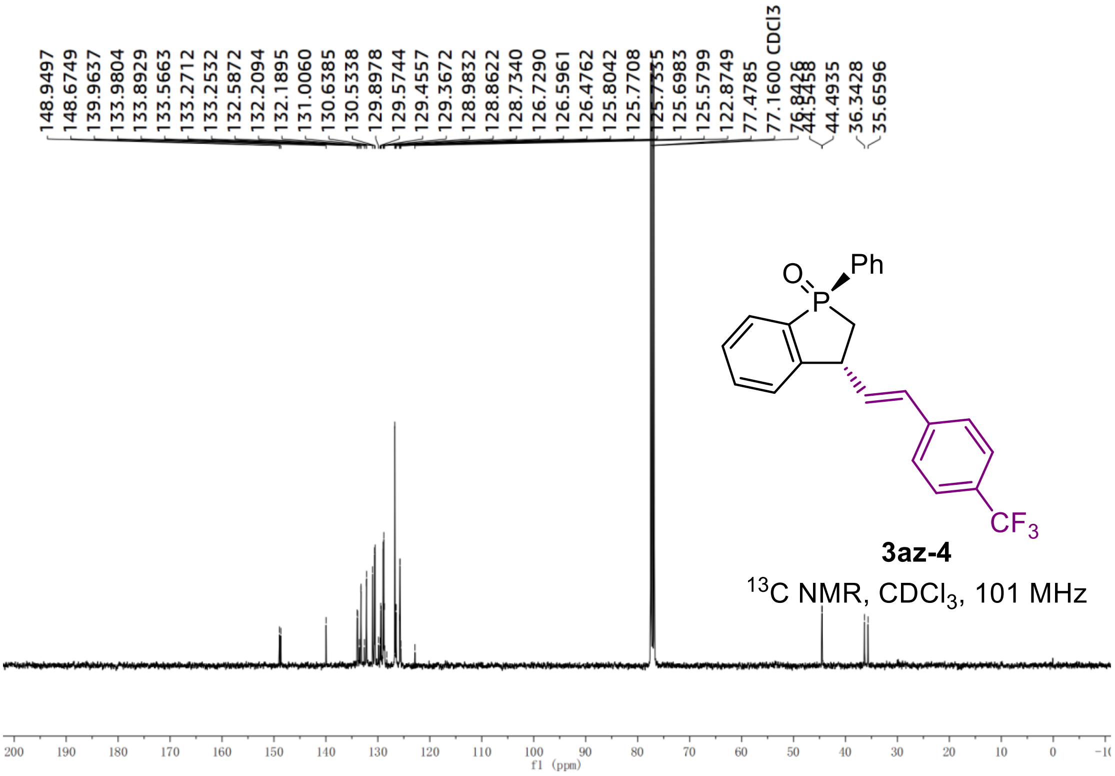

Supplementary Figure 103. ^{13}**C NMR of the 3az-4 (101 MHz, CDCl$_3$)**

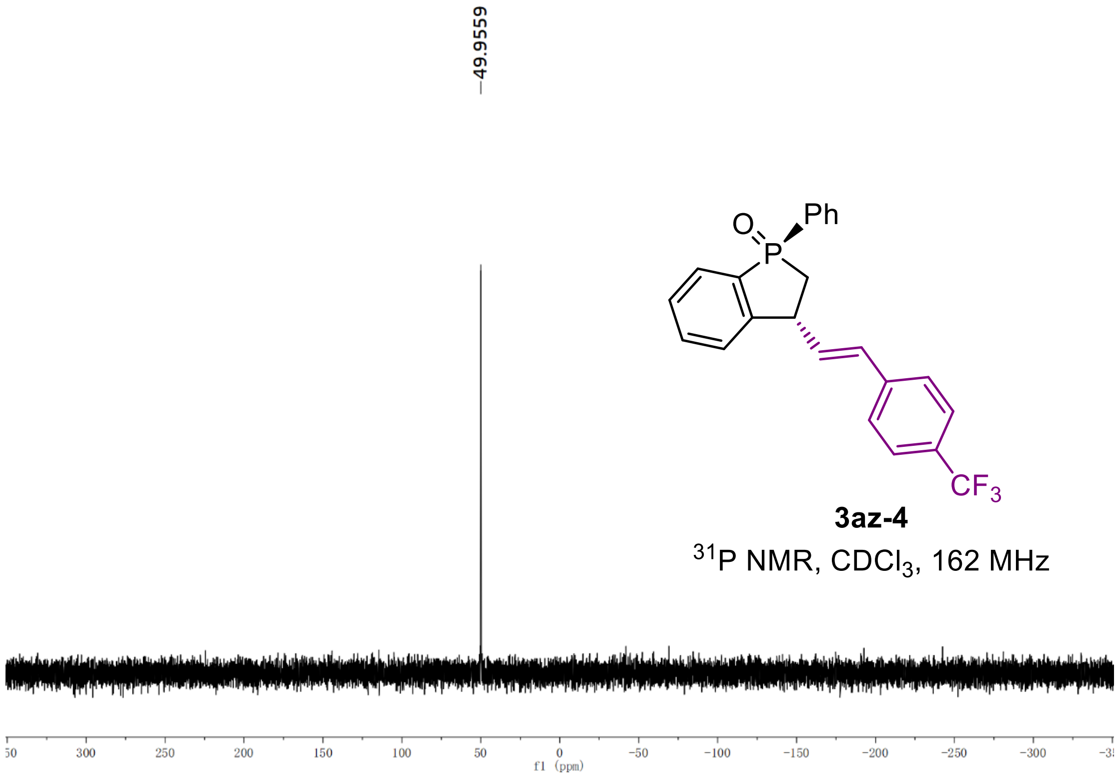

Supplementary Figure 104. ^{31}**P NMR of the 3az-4 (162 MHz, $CDCl_3$)**

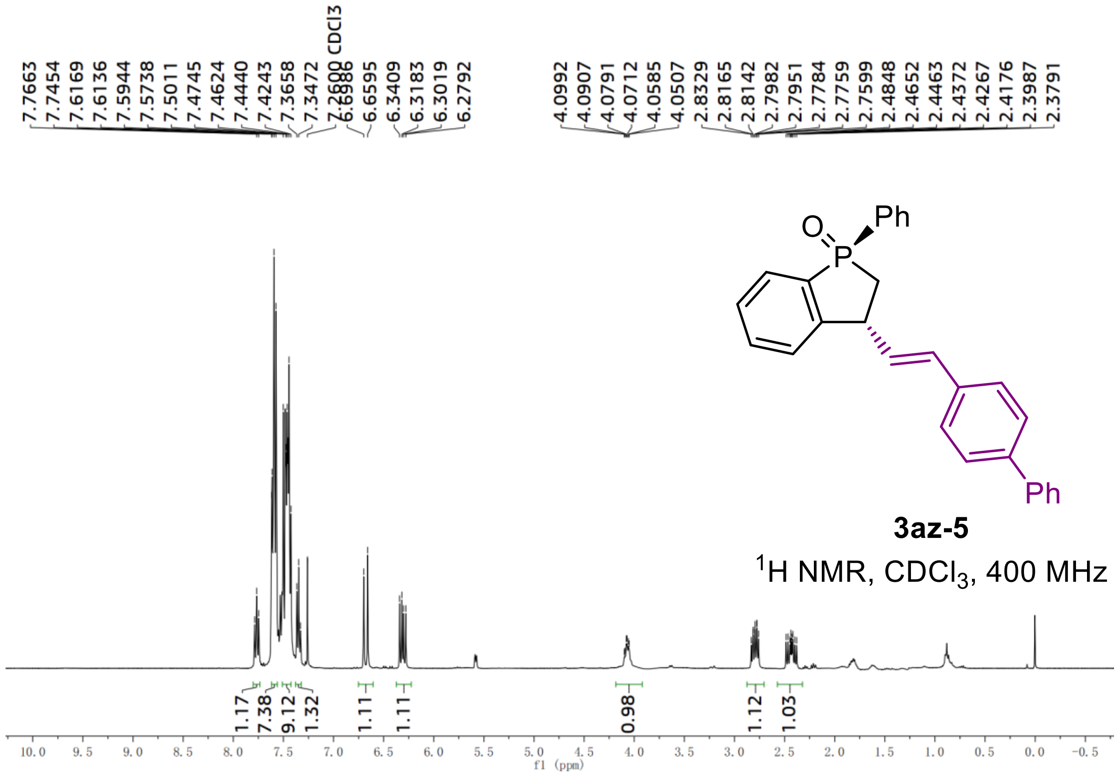

Supplementary Figure 105. ^{1}**H NMR of the 3az-5 (400 MHz, $CDCl_3$)**

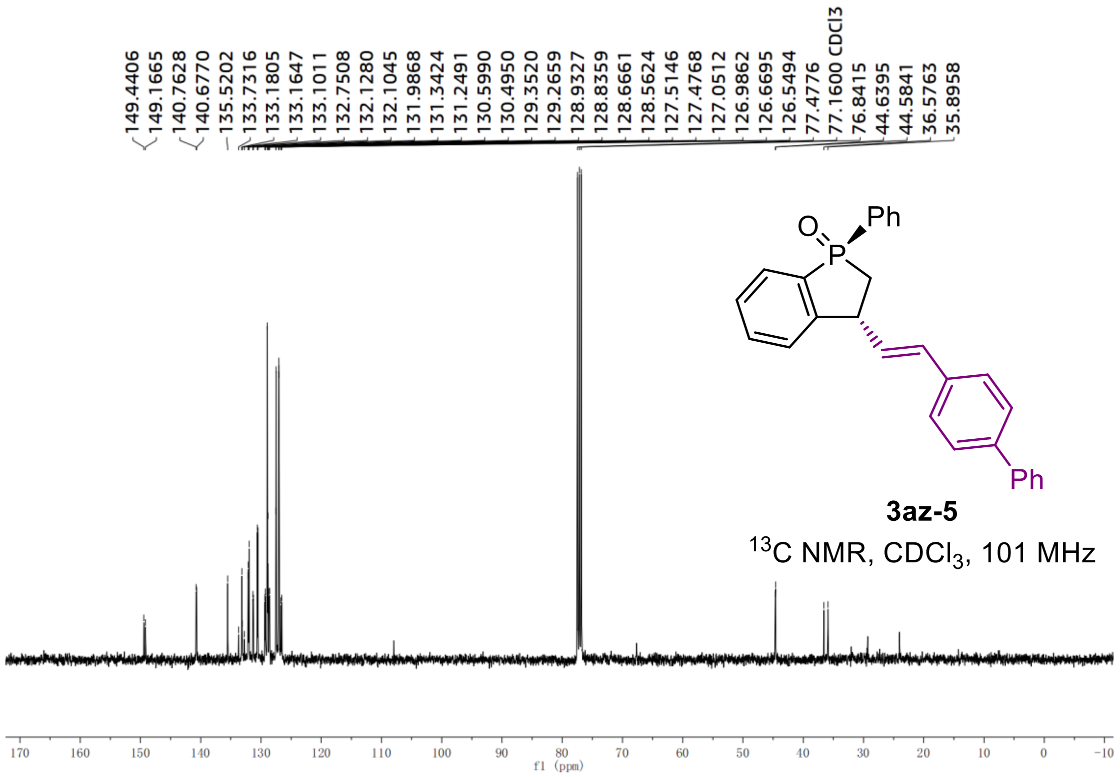

Supplementary Figure 106. ^{13}**C NMR of the 3az-5 (101 MHz, CDCl$_3$)**

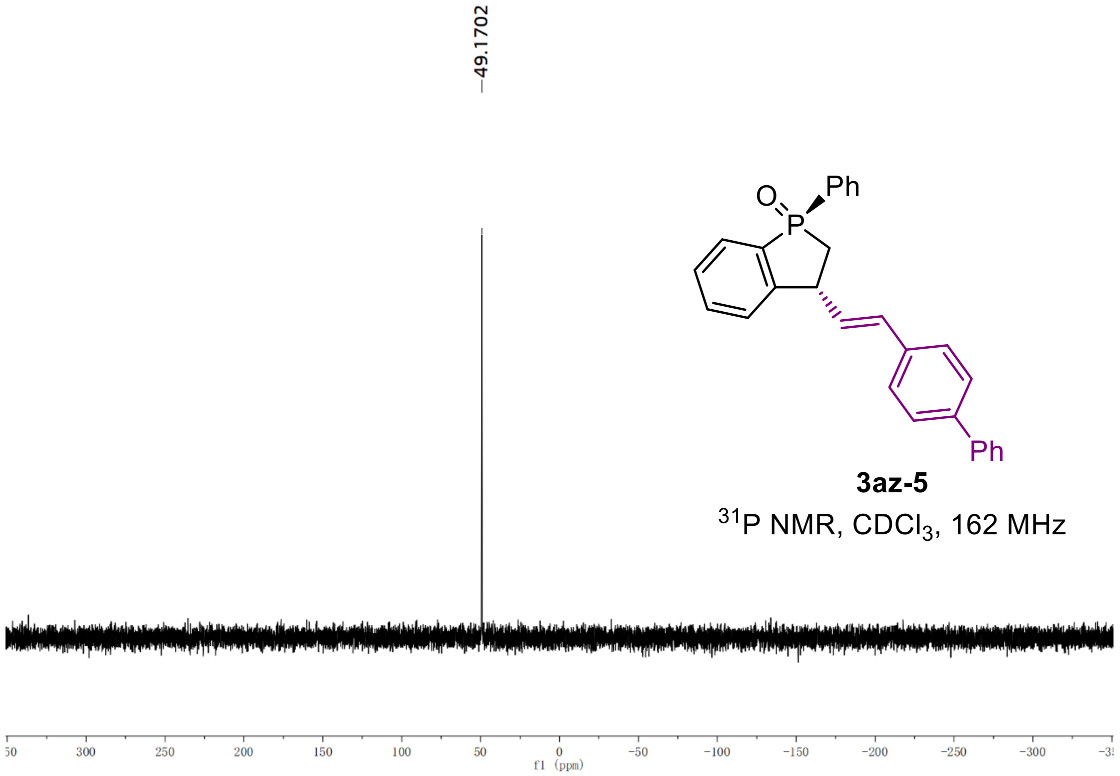

Supplementary Figure 107. ^{31}**P NMR of the 3az-5 (162 MHz, CDCl$_3$)**

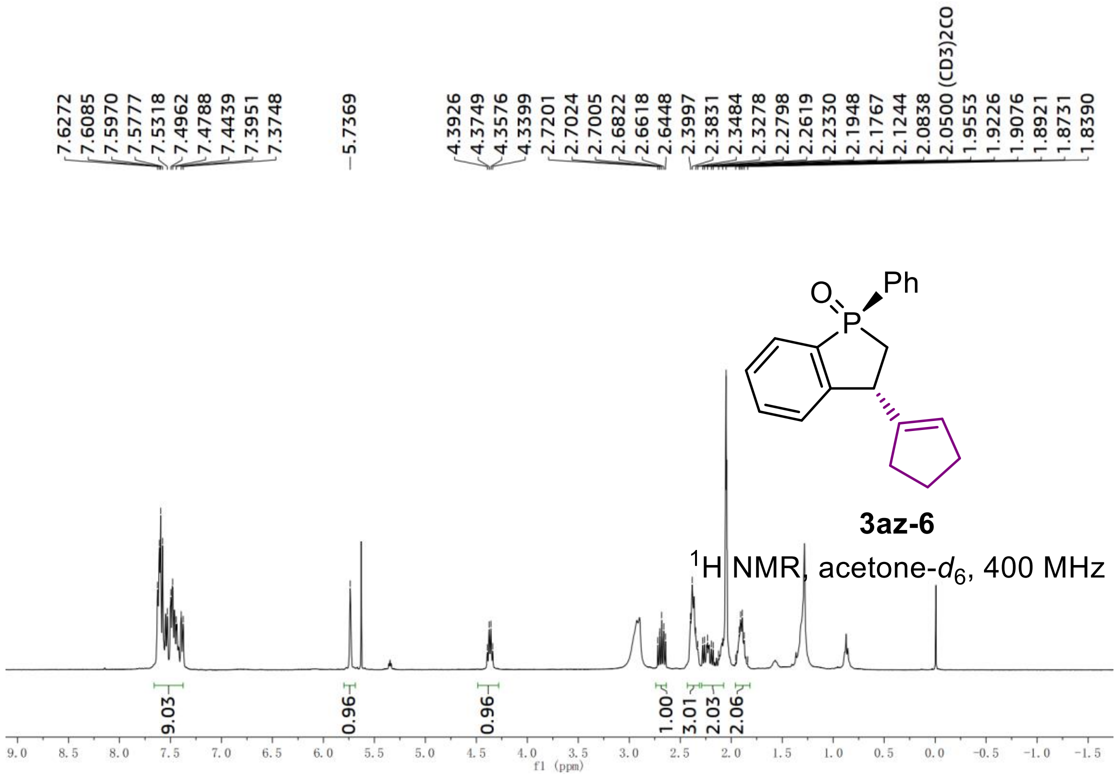

Supplementary Figure 108. ^{1}H NMR of the 3az-6 (400 MHz, acetone-d_6)

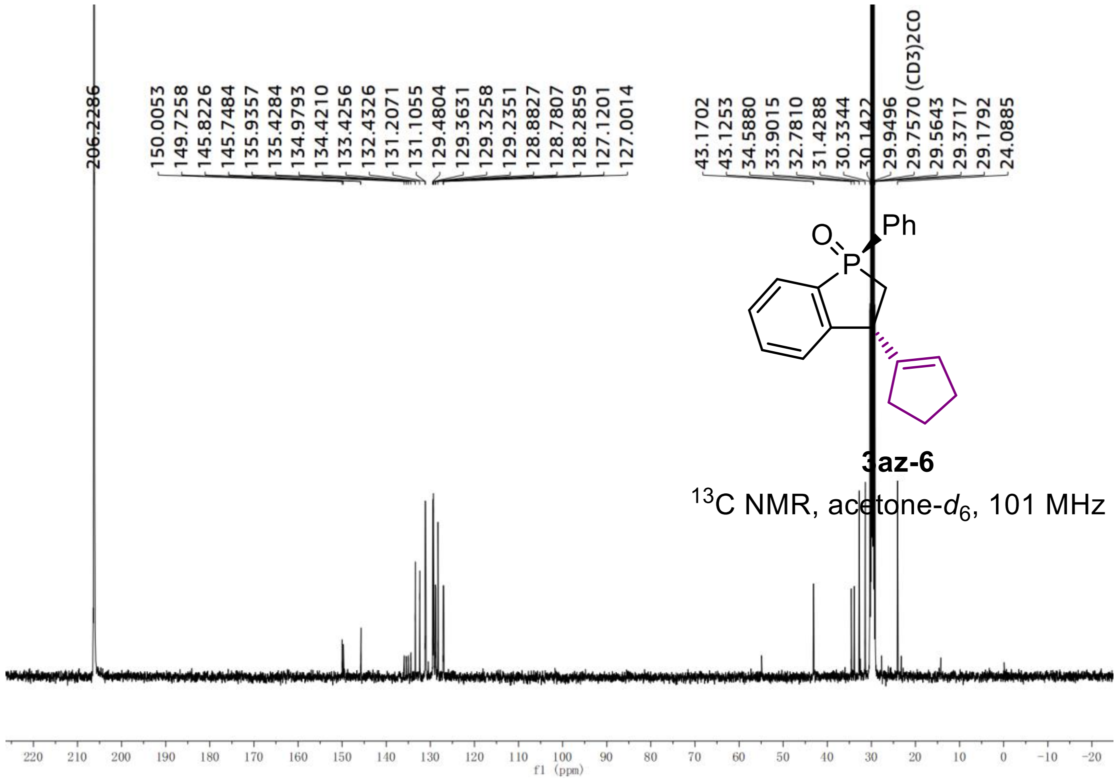

Supplementary Figure 109. ^{13}C NMR of the 3az-6 (101 MHz, acetone-d_6)

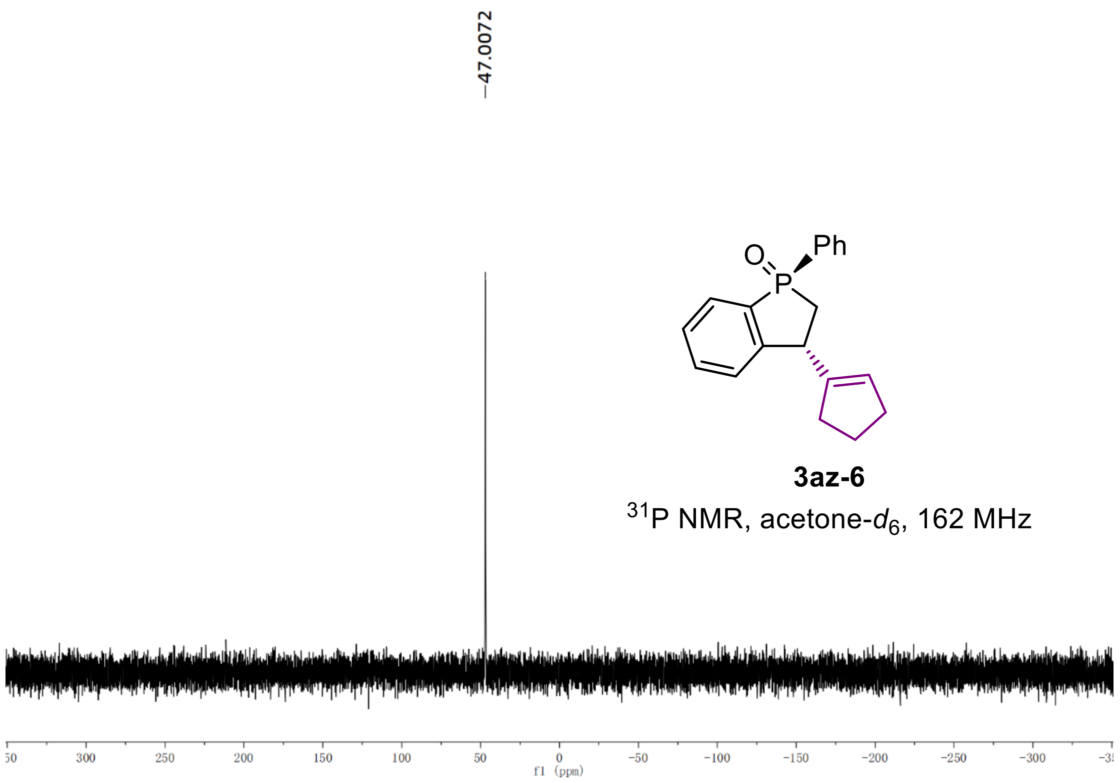

Supplementary Figure 110. ^{31}P NMR of the 3az-6 (162 MHz, acetone-d_6)

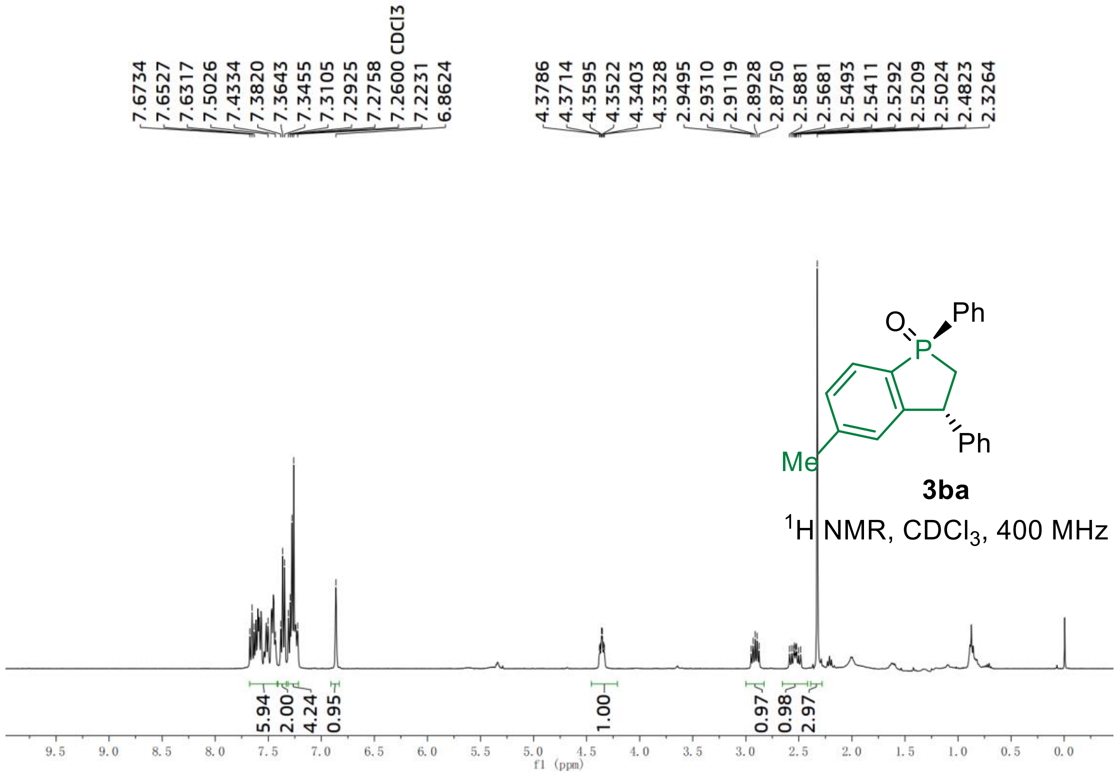

Supplementary Figure 111. ^{1}H NMR of the 3ba (400 MHz, $CDCl_3$)

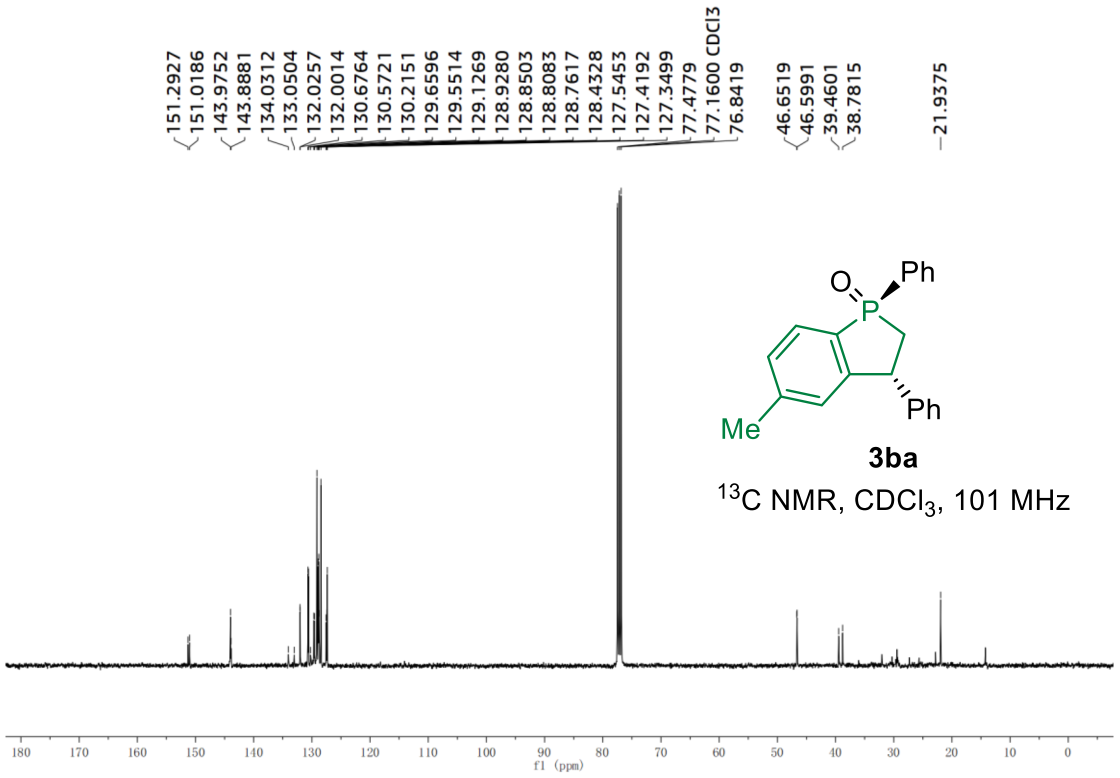

Supplementary Figure 112. ^{13}**C NMR of the 3ba (101 MHz,** $CDCl_3$**)**

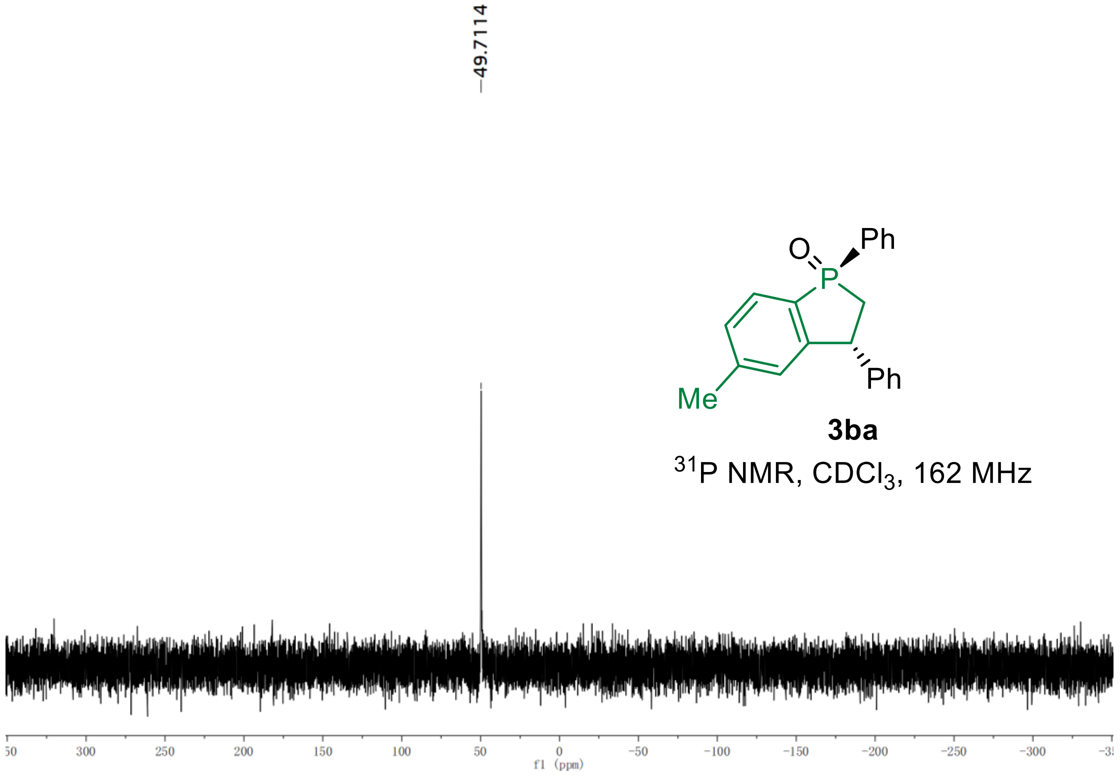

Supplementary Figure 113. ^{31}**P NMR of the 3ba (162 MHz,** $CDCl_3$**)**

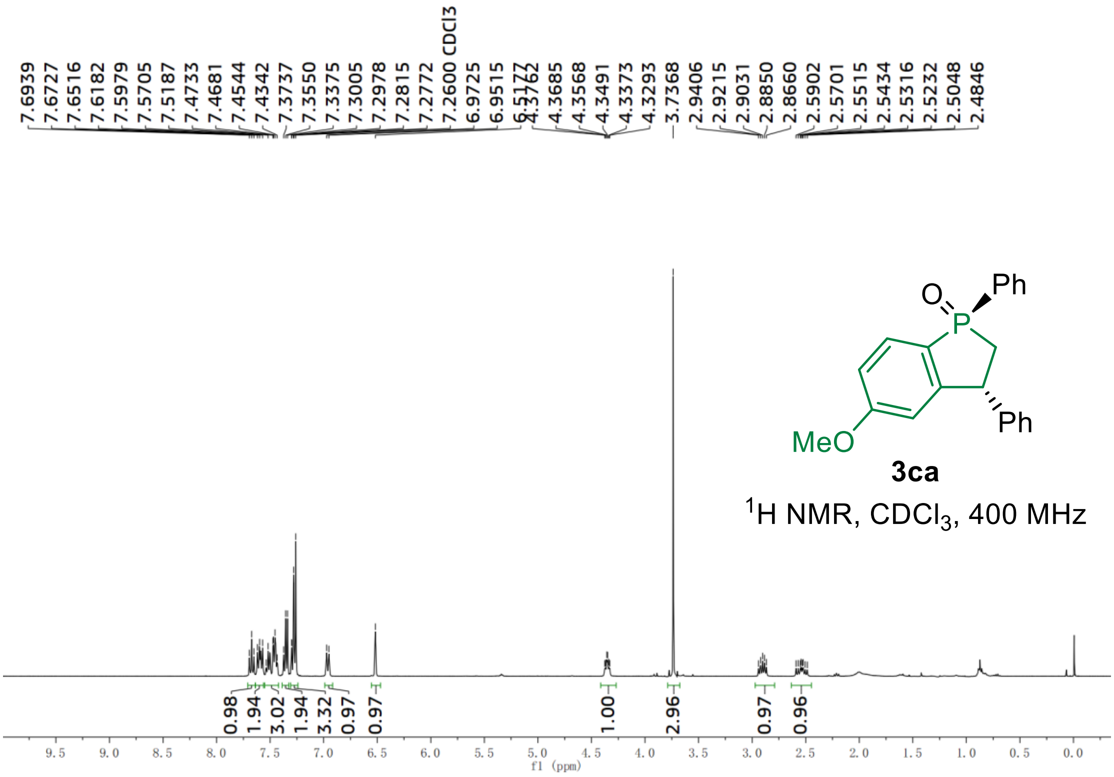

Supplementary Figure 114. ^{1}**H NMR of the 3ca (400 MHz, CDCl**$_3$**)**

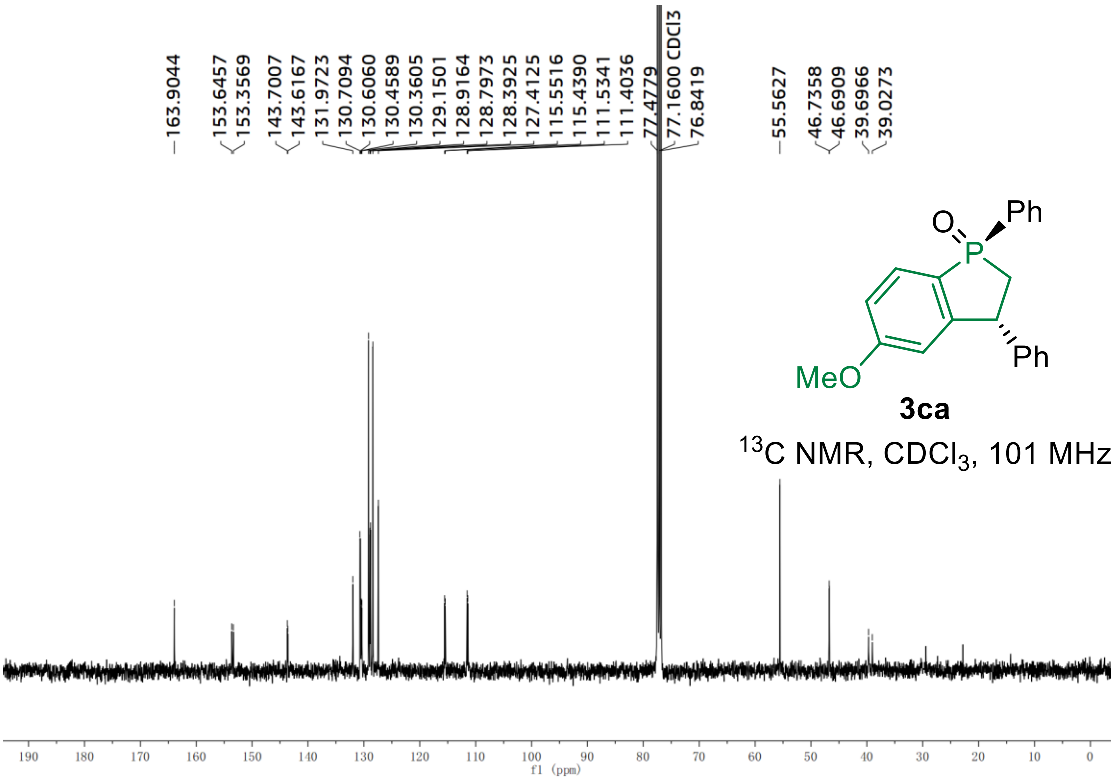

Supplementary Figure 115. ^{13}**C NMR of the 3ca (101 MHz, CDCl**$_3$**)**

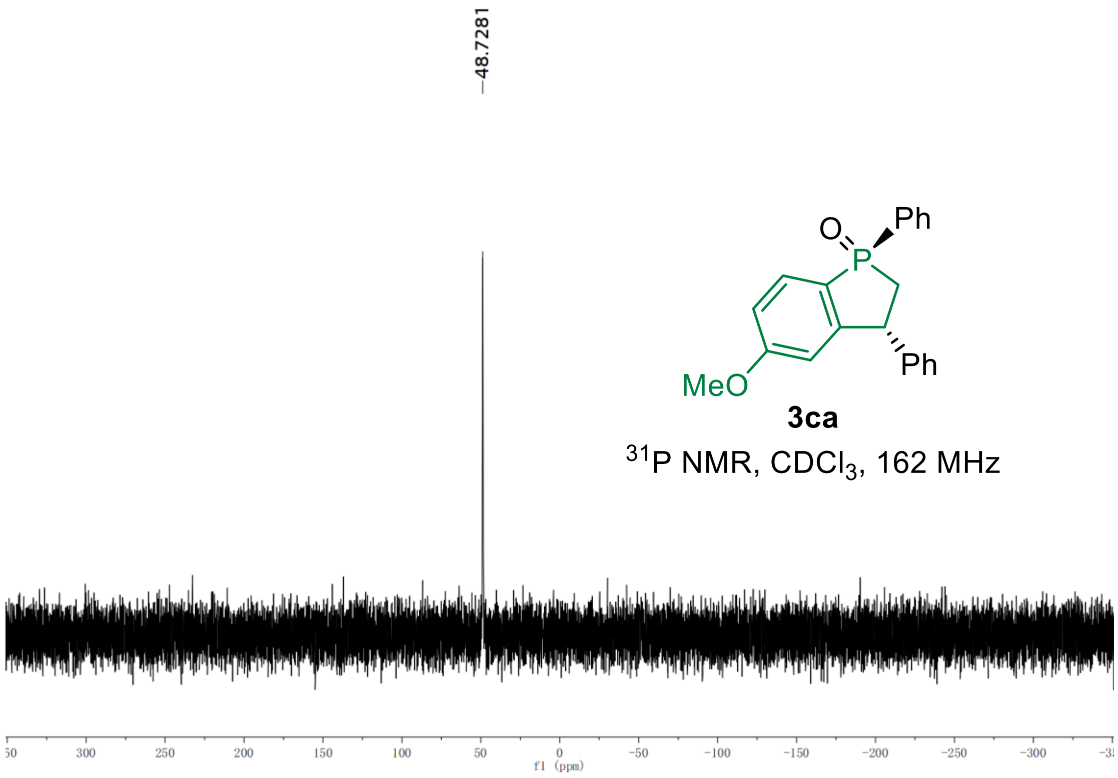

Supplementary Figure 116. ^{31}P NMR of the 3ca (162 MHz, $CDCl_3$)

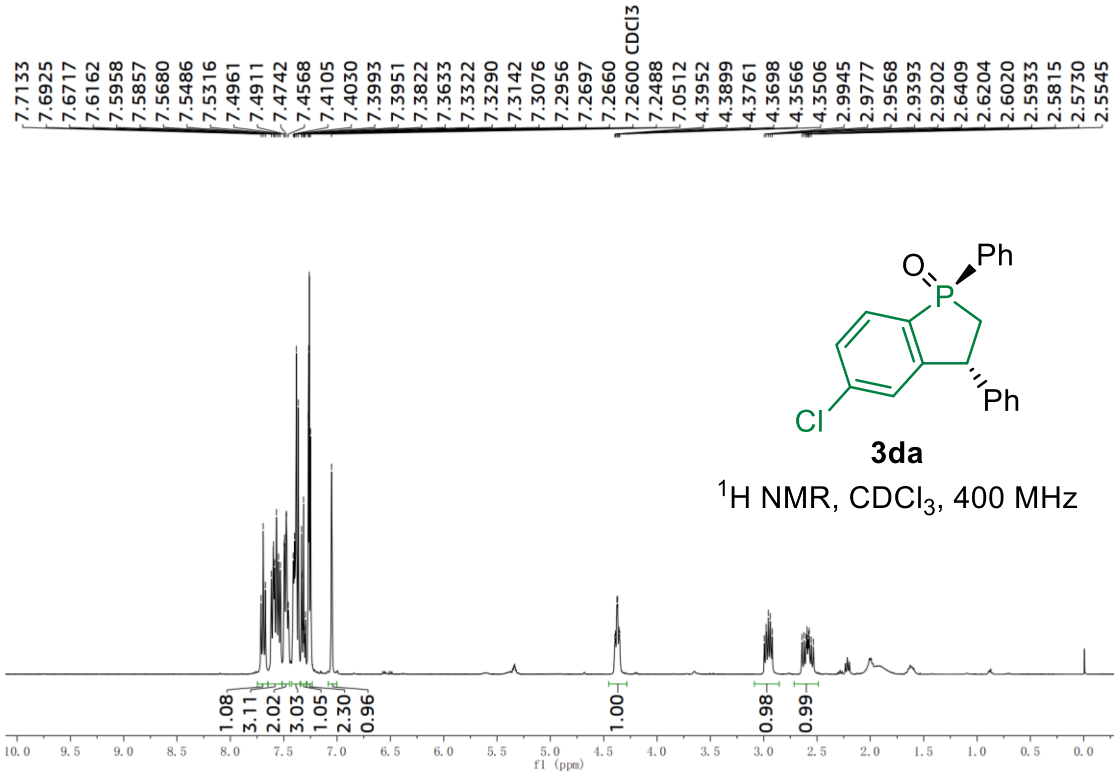

Supplementary Figure 117. ^{1}H NMR of the 3da (400 MHz, $CDCl_3$)

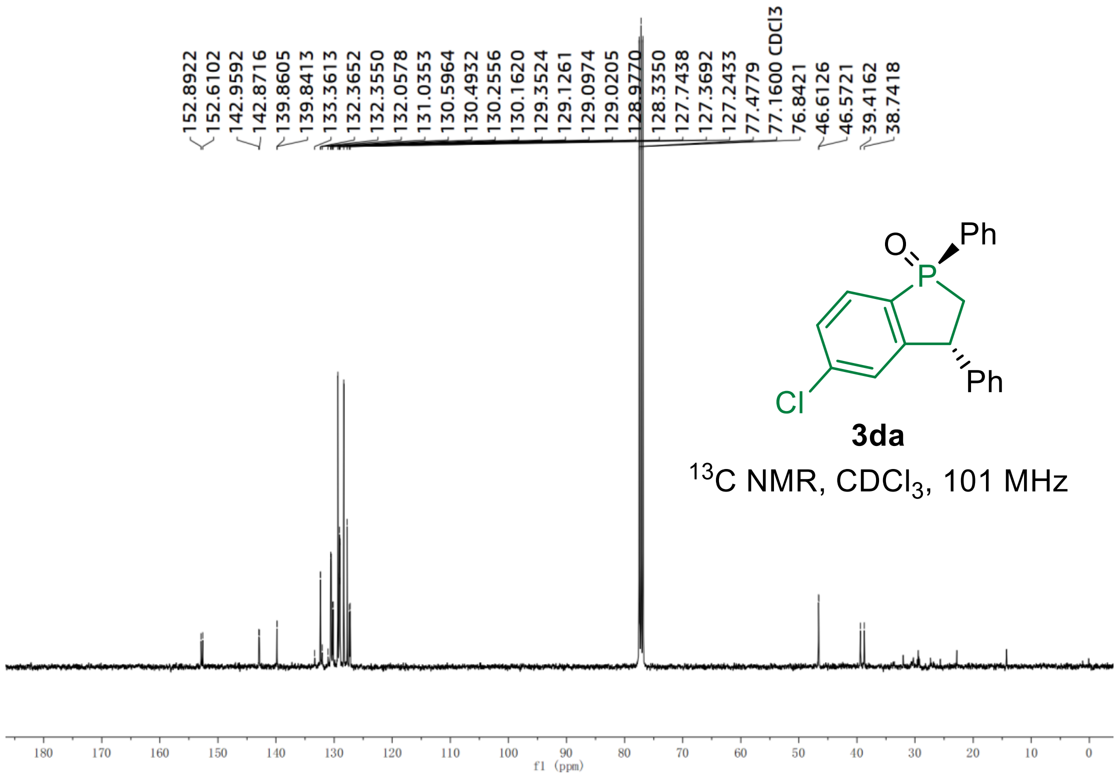

Supplementary Figure 118. ^{13}C **NMR of the 3da (101 MHz,** $CDCl_3$**)**

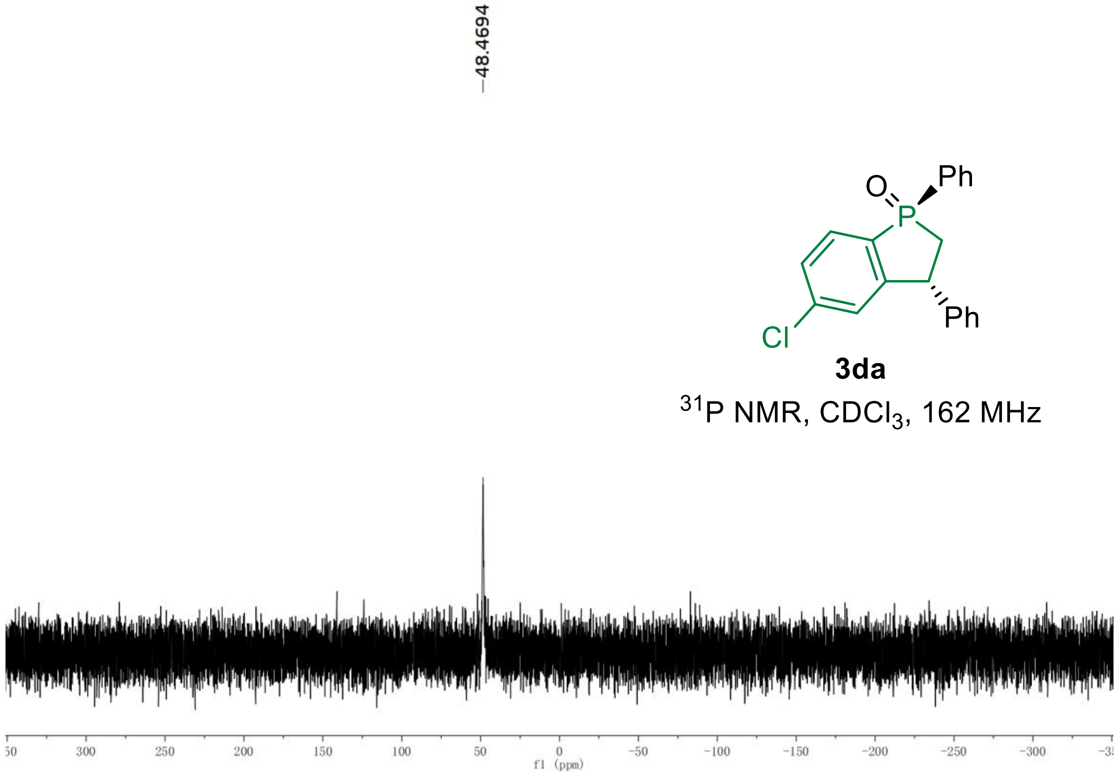

Supplementary Figure 119. ^{31}P **NMR of the 3da (162 MHz,** $CDCl_3$**)**

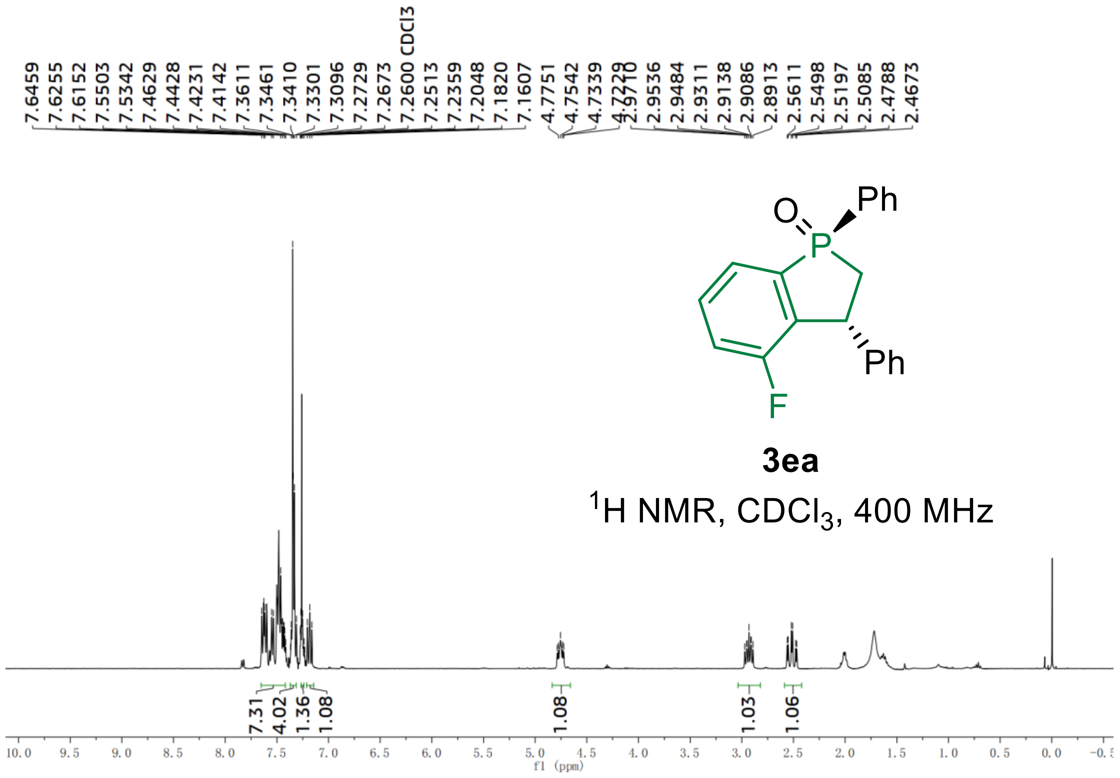

Supplementary Figure 120. ^{1}H NMR of the 3ea (400 MHz, $CDCl_3$)

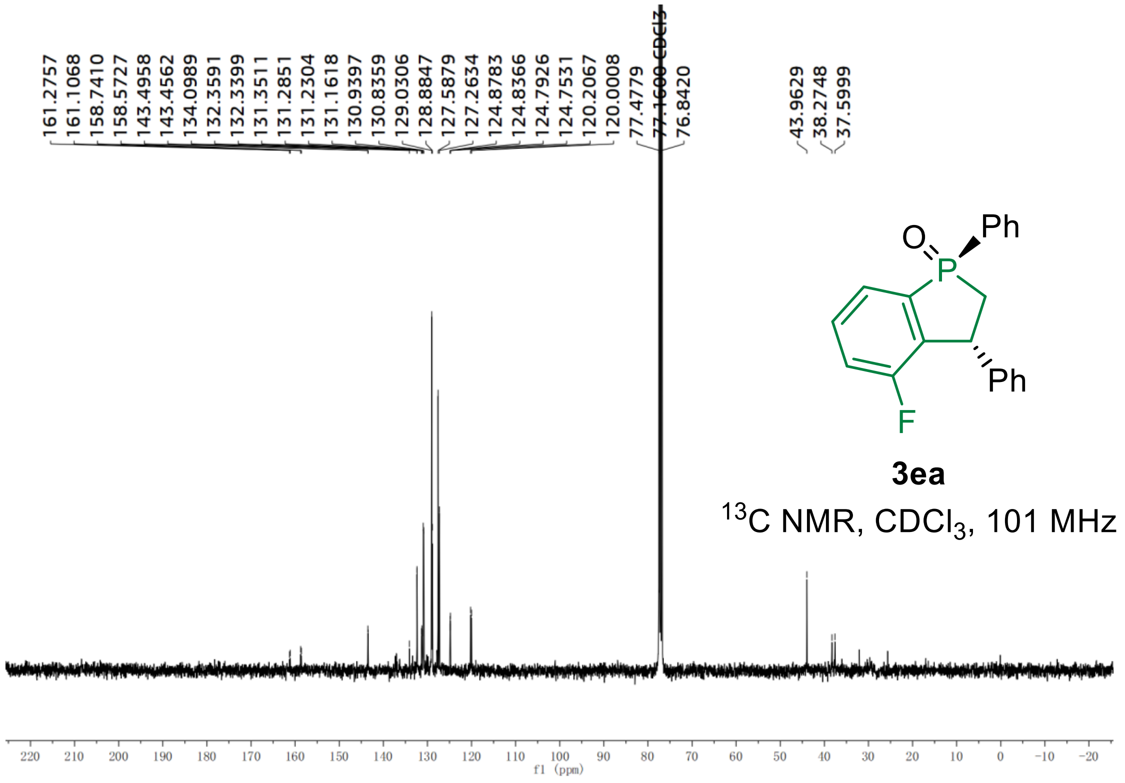

Supplementary Figure 121. ^{13}C NMR of the 3ea (101 MHz, $CDCl_3$)

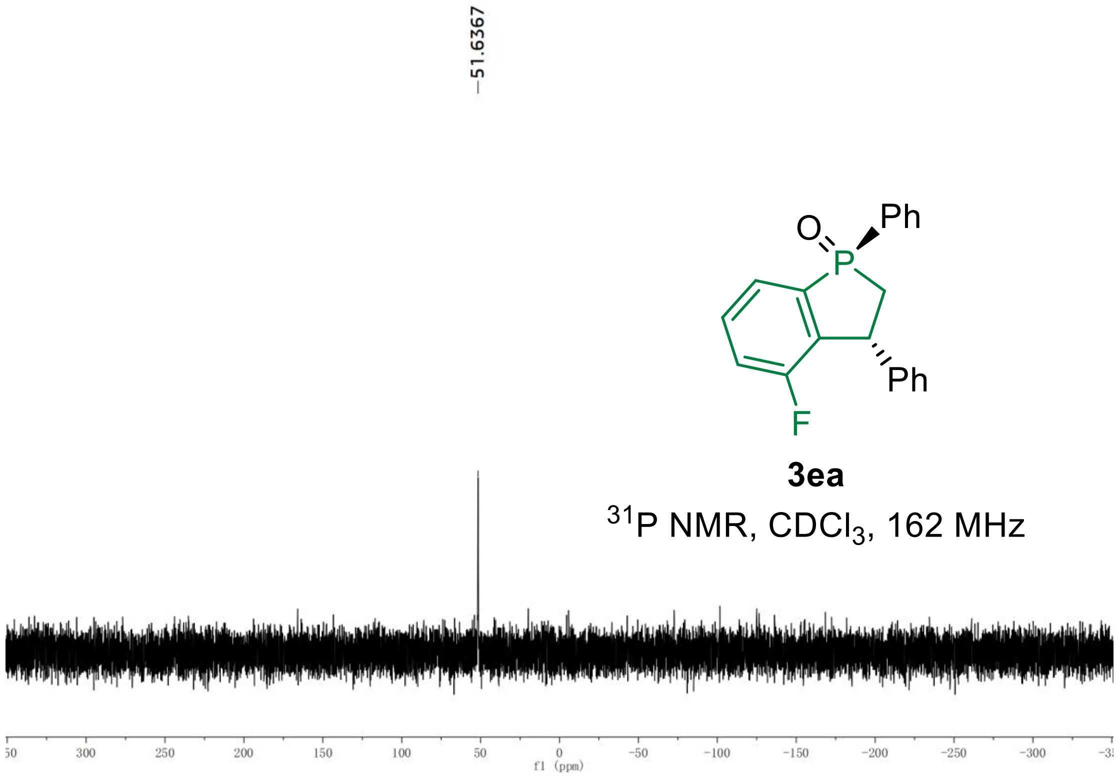

Supplementary Figure 122. ^{31}**P NMR of the 3ea (162 MHz,** $CDCl_3$**)**

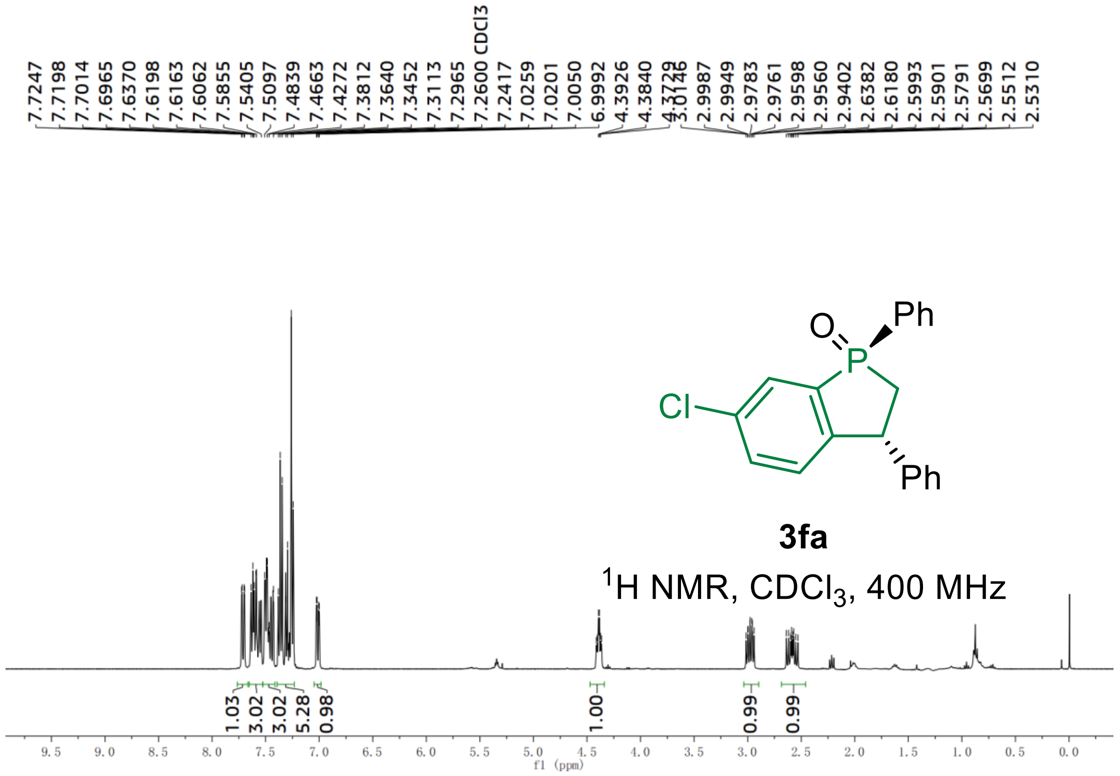

Supplementary Figure 123. ^{1}**H NMR of the 3fa (400 MHz,** $CDCl_3$**)**

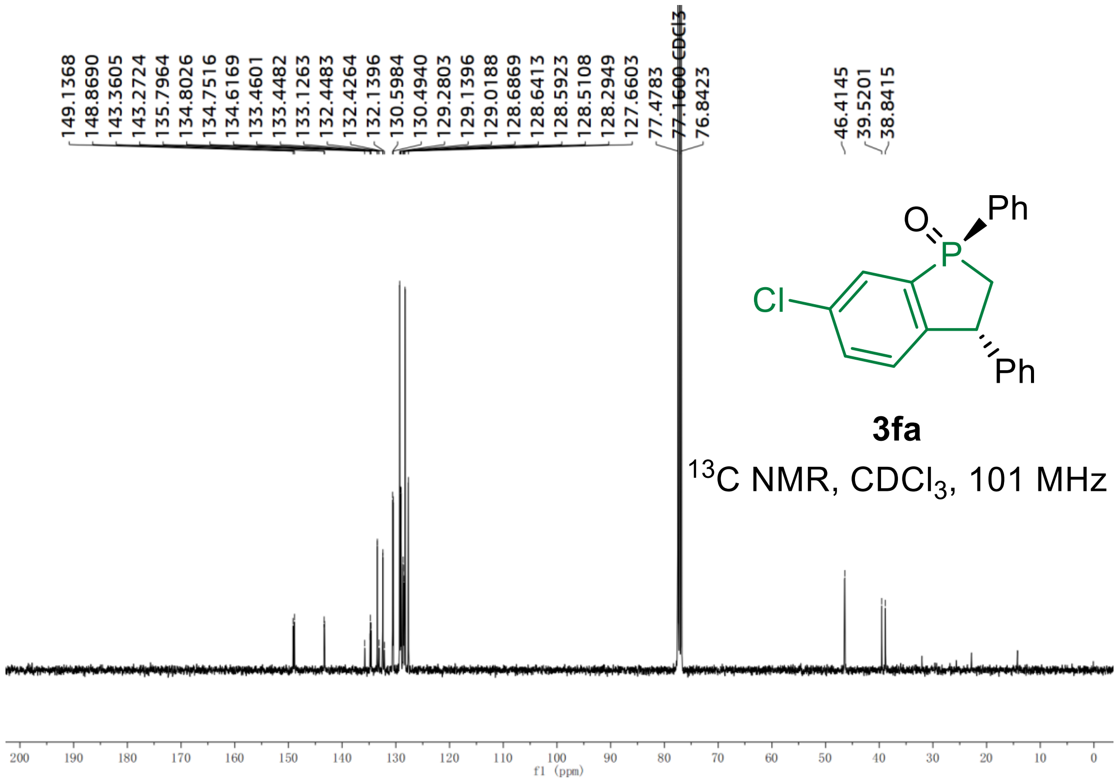

Supplementary Figure 124. ^{13}**C NMR of the 3fa (101 MHz, CDCl**$_3$**)**

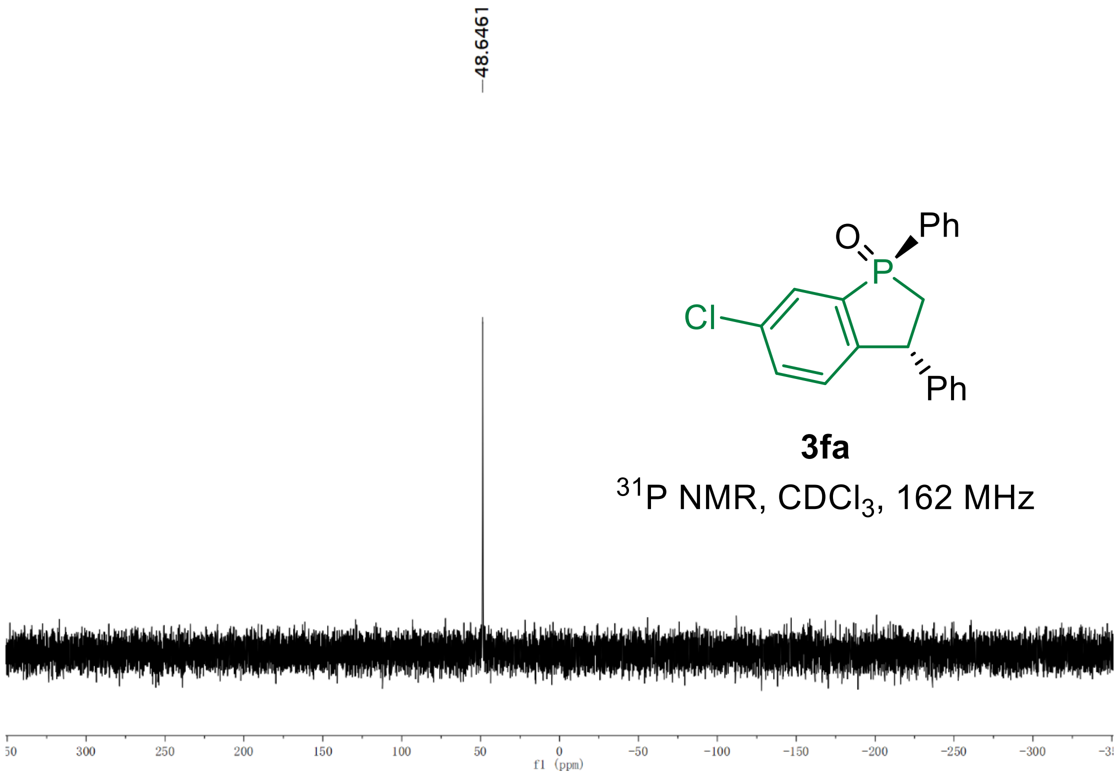

Supplementary Figure 125. ^{31}**P NMR of the 3fa (162 MHz, CDCl**$_3$**)**

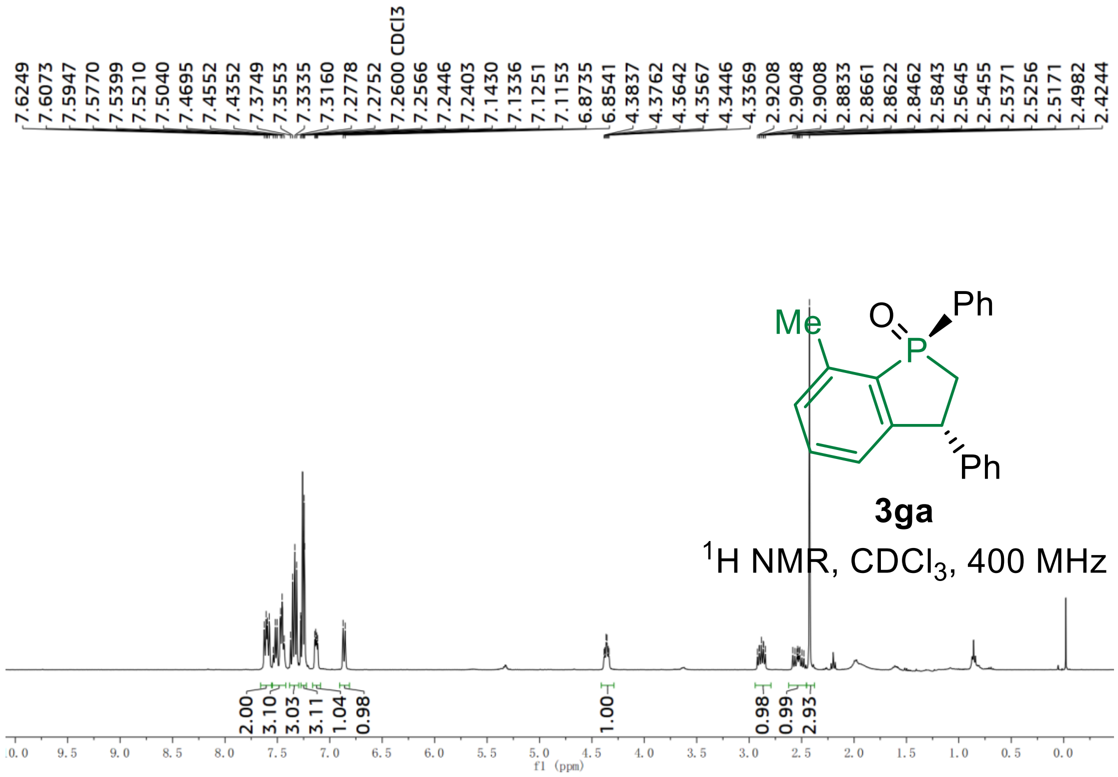

Supplementary Figure 126. ^{1}H NMR of the 3ga (400 MHz, $CDCl_3$)

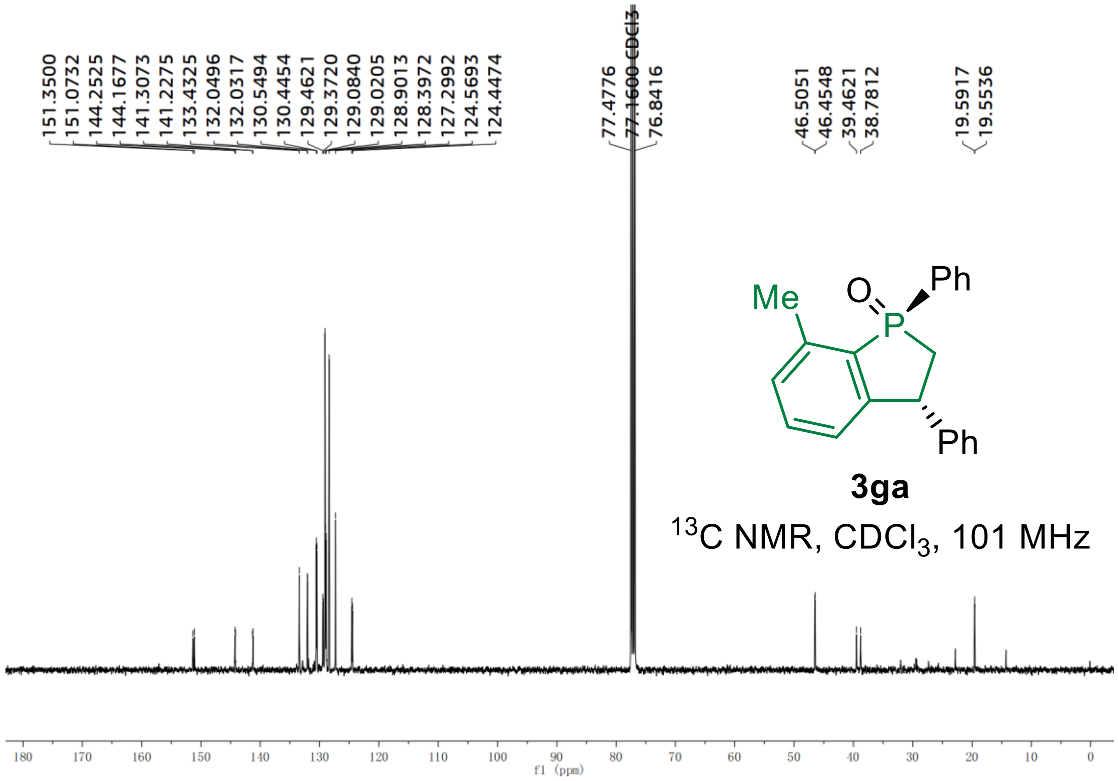

Supplementary Figure 127. ^{13}C NMR of the 3ga (101 MHz, $CDCl_3$)

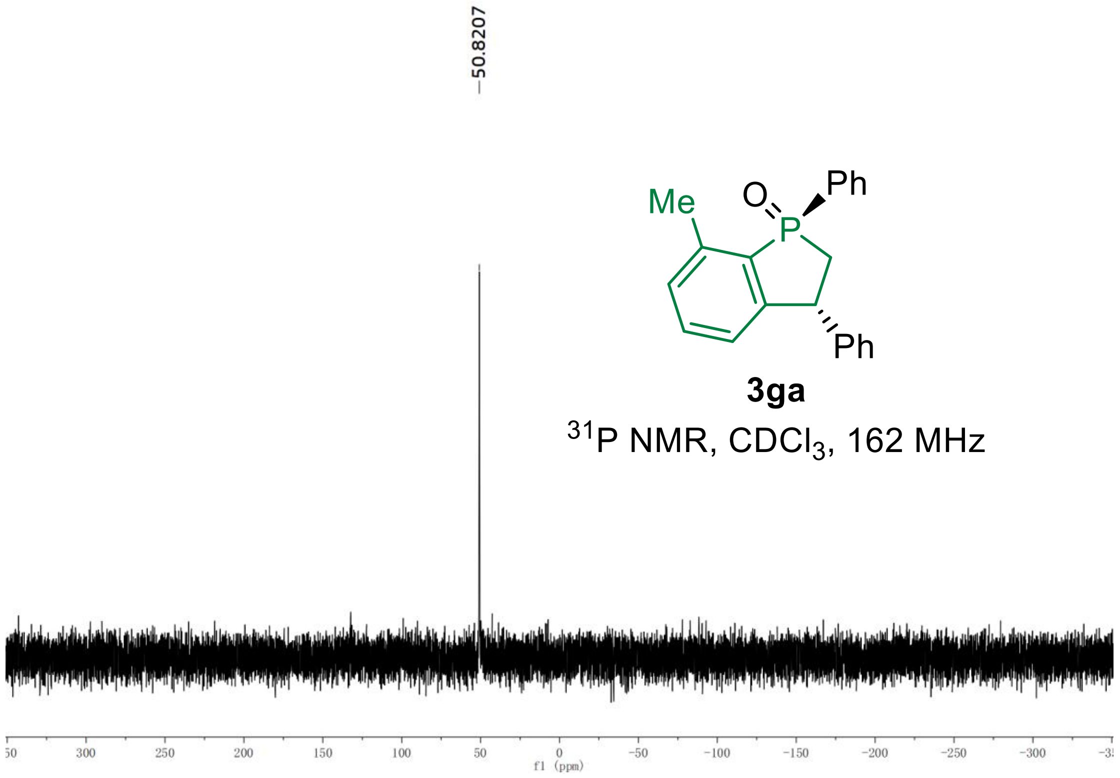

Supplementary Figure 128. ^{31}P NMR of the 3ga (162 MHz, $CDCl_3$)

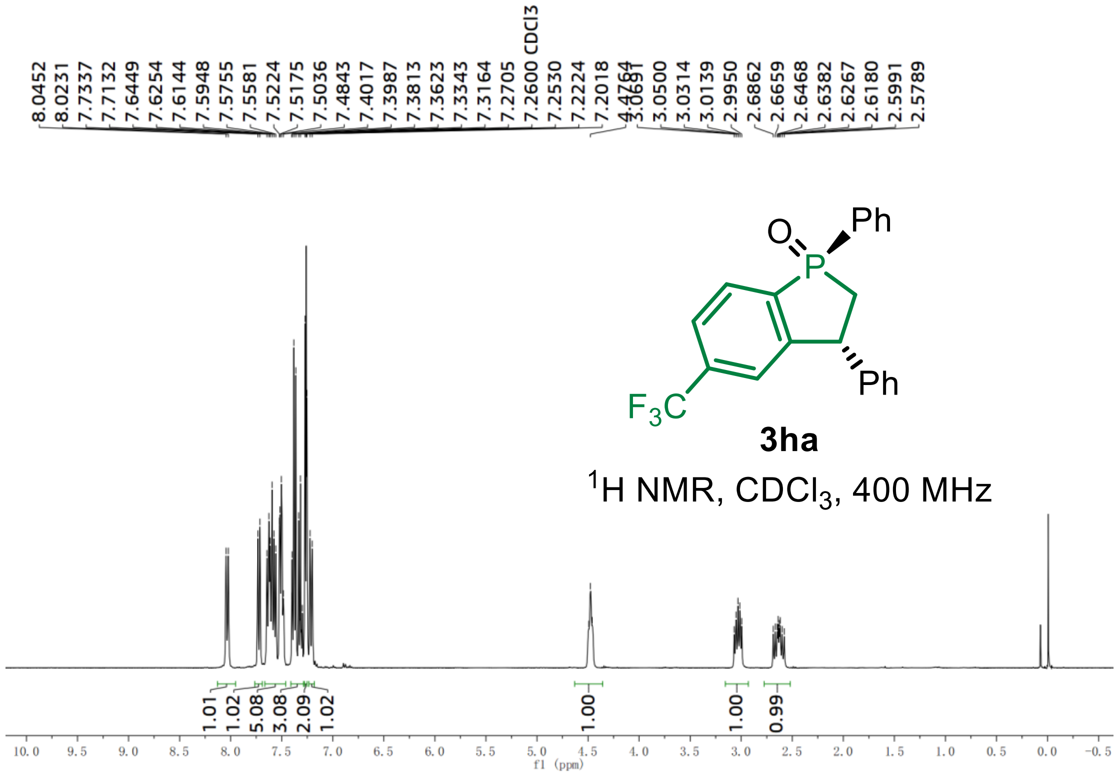

Supplementary Figure 129. ^{1}H NMR of the 3ha (400 MHz, $CDCl_3$)

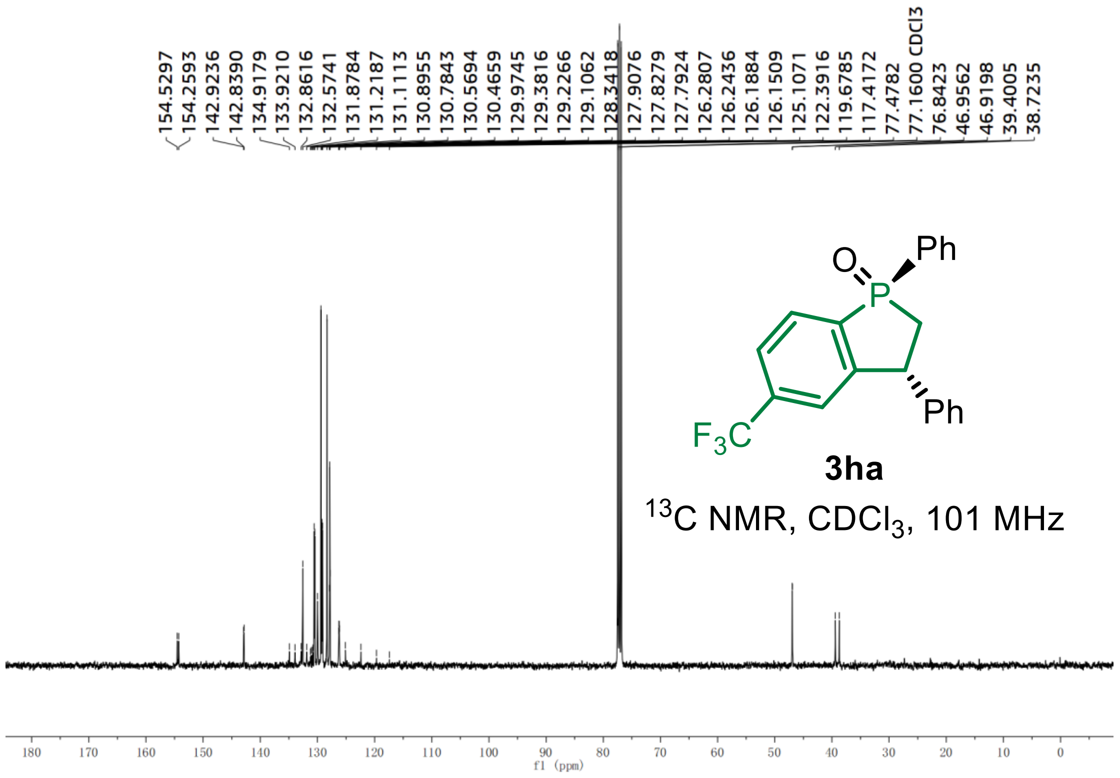

Supplementary Figure 130. ^{13}**C NMR of the 3ha (101 MHz,** $CDCl_3$**)**

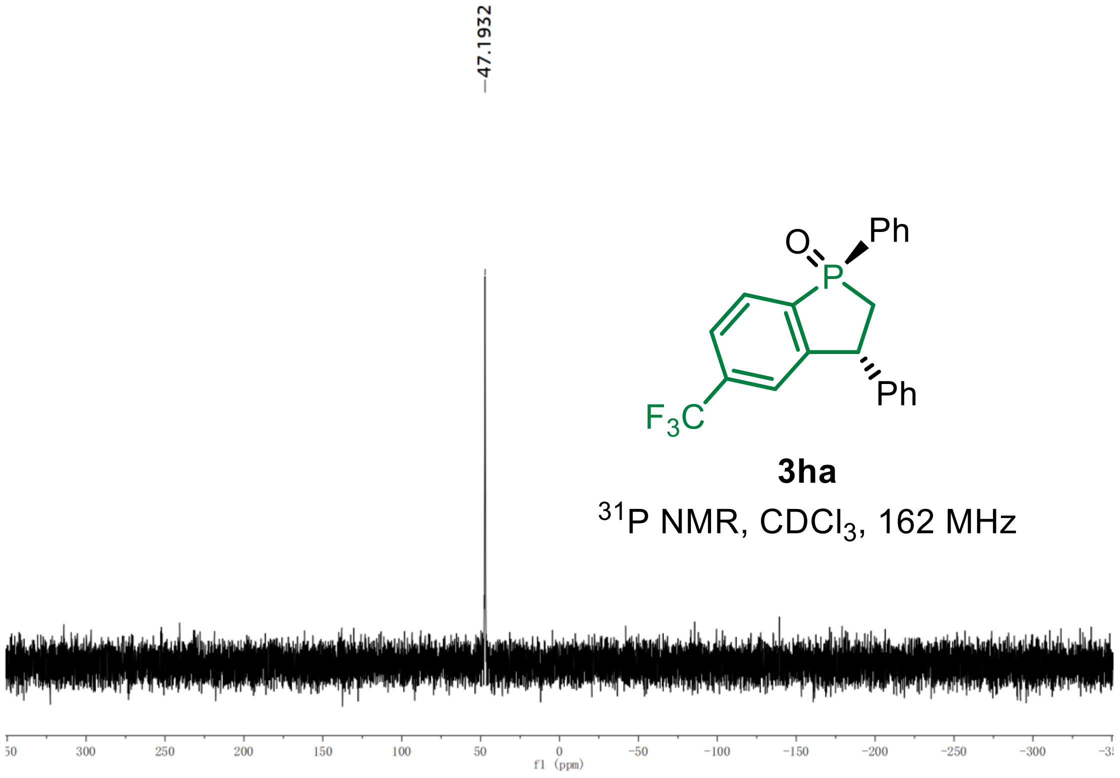

Supplementary Figure 131. ^{31}**P NMR of the 3ha (162 MHz,** $CDCl_3$**)**

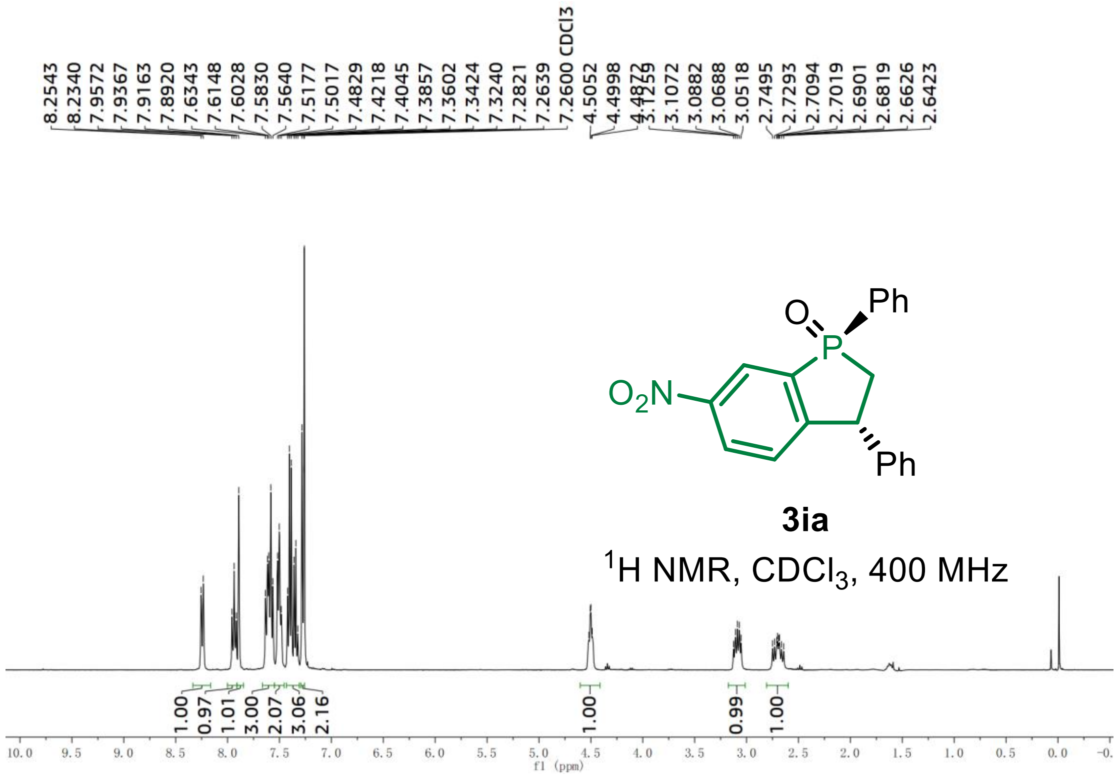

Supplementary Figure 132. ^{1}**H NMR of the 3ia (400 MHz, CDCl$_3$)**

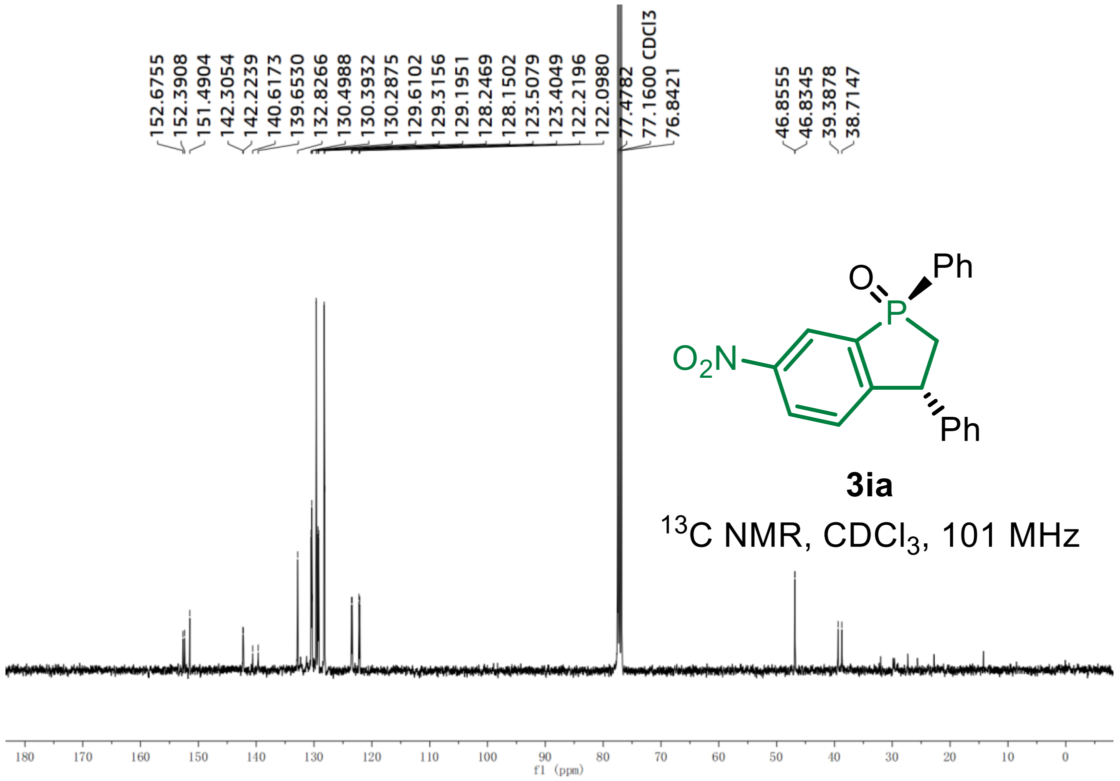

Supplementary Figure 133. ^{13}**C NMR of the 3ia (101 MHz, CDCl$_3$)**

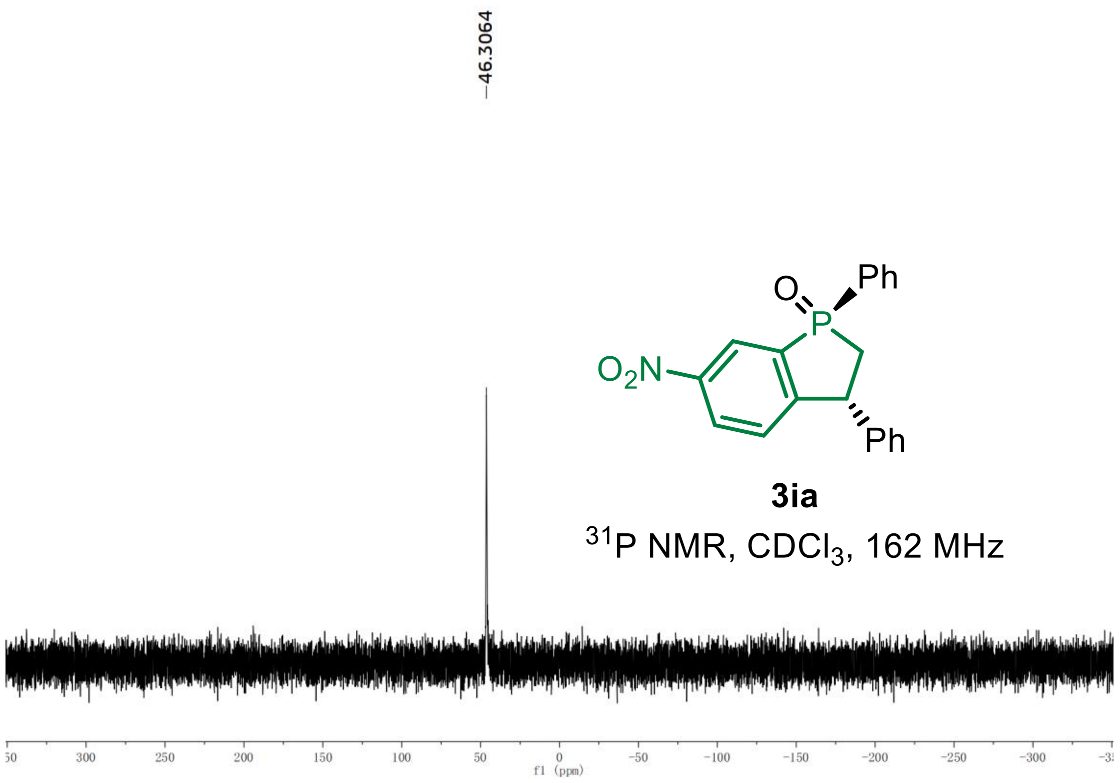

Supplementary Figure 134. ^{31}**P NMR of the 3ia (162 MHz, $CDCl_3$)**

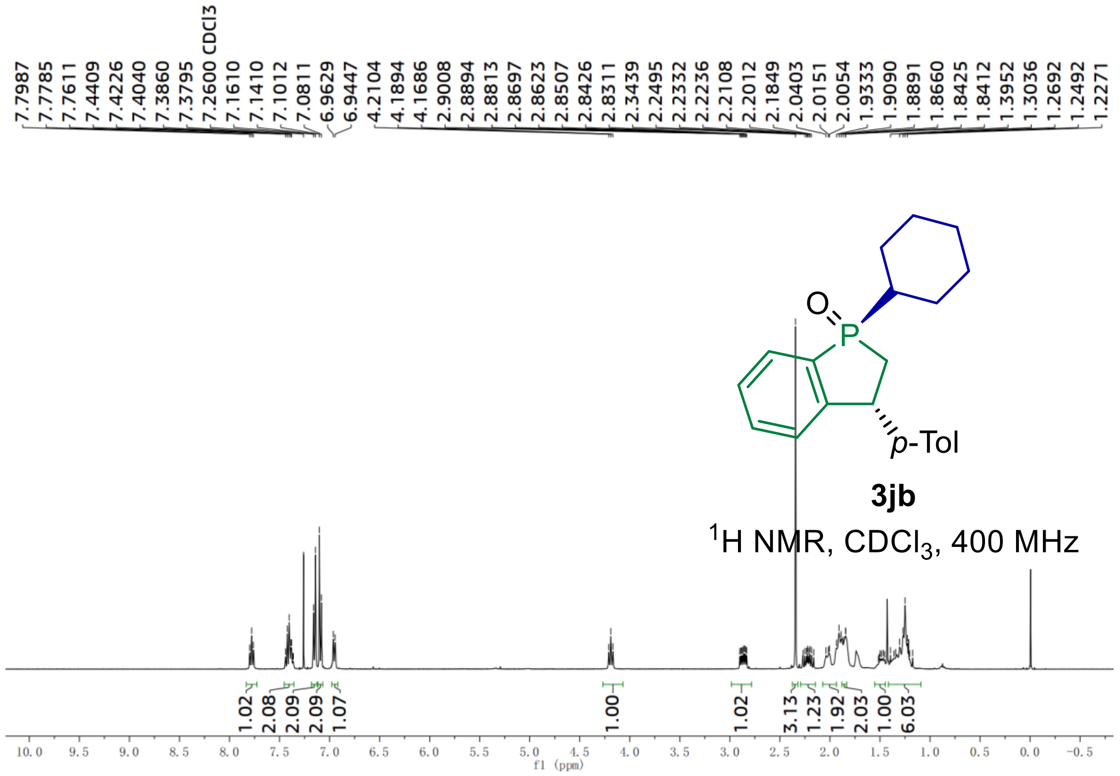

Supplementary Figure 135. ^{1}**H NMR of the 3jb (400 MHz, $CDCl_3$)**

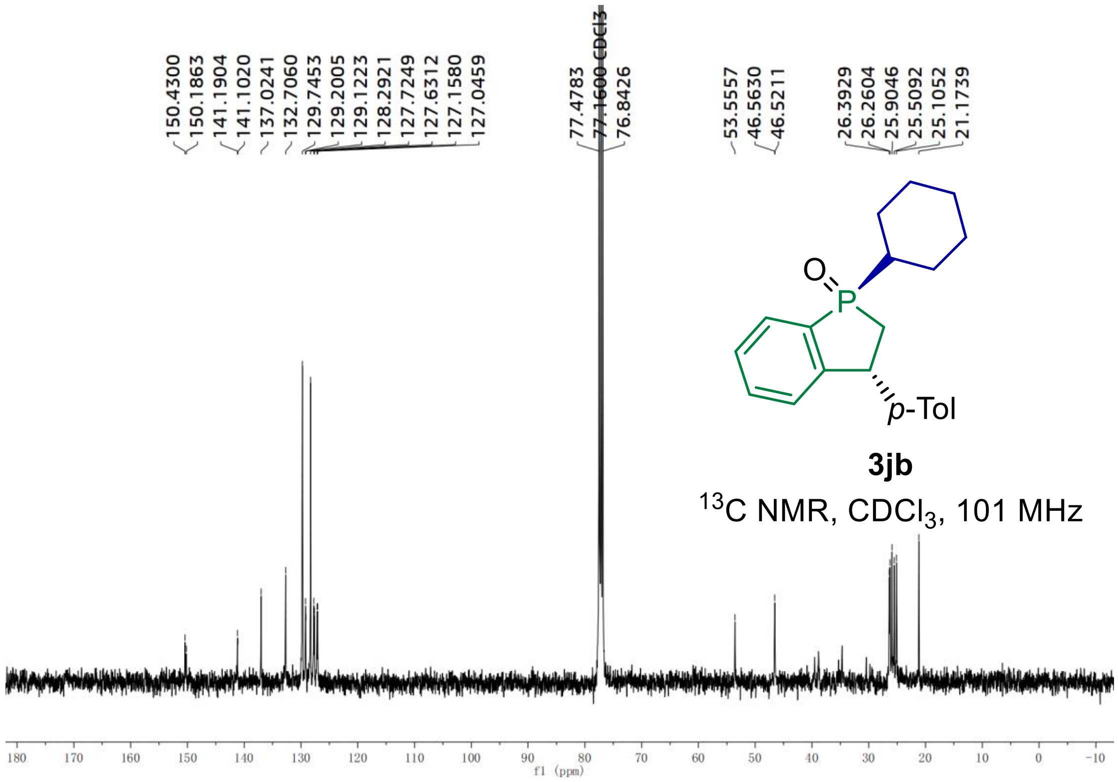

Supplementary Figure 136. ^{13}**C NMR of the 3jb (101 MHz,** $CDCl_3$**)**

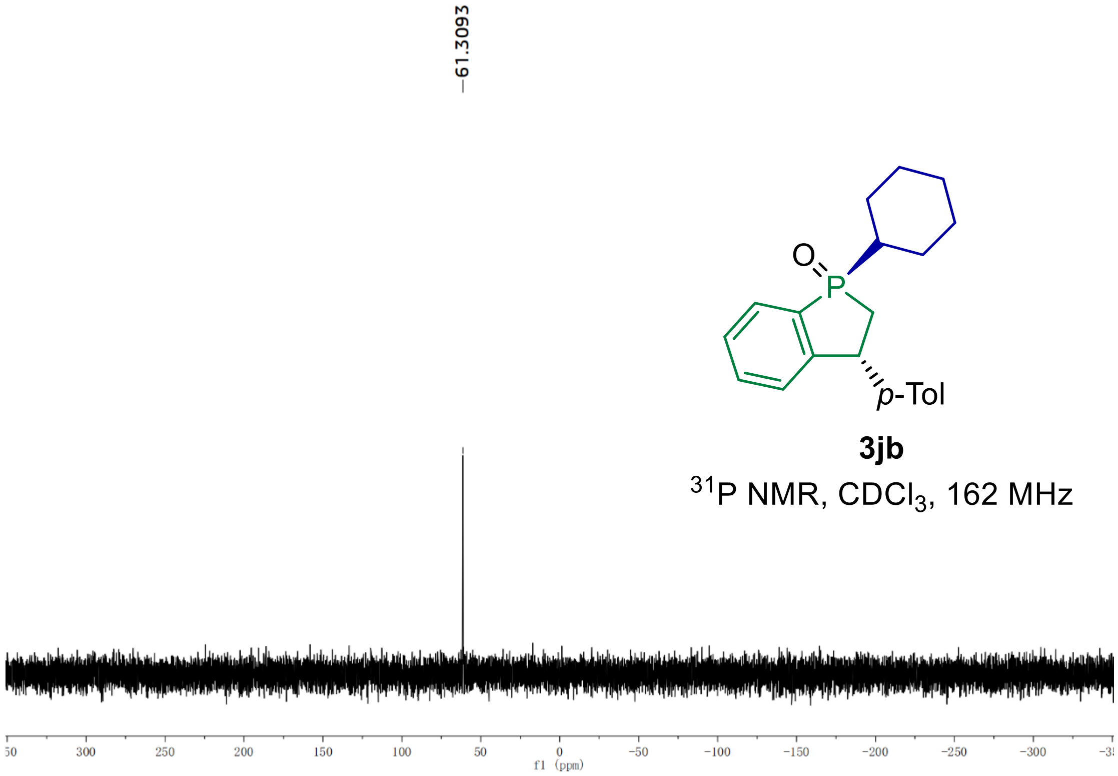

Supplementary Figure 137. ^{31}**P NMR of the 3jb (162 MHz,** $CDCl_3$**)**

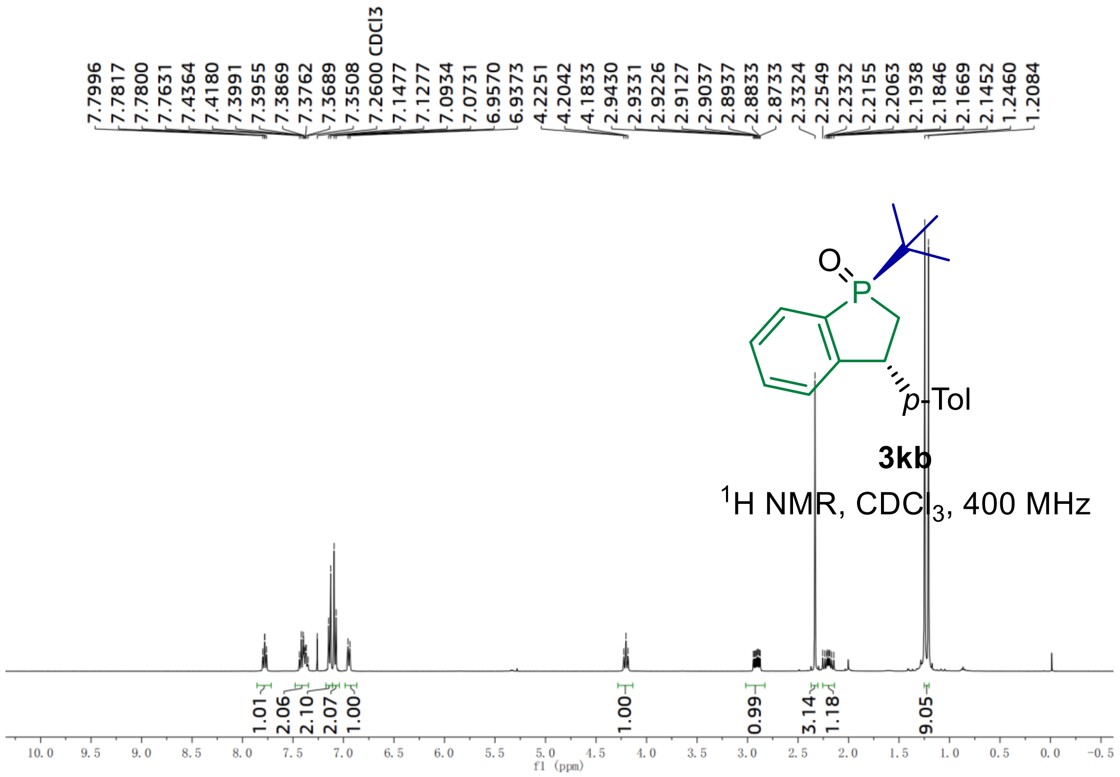

Supplementary Figure 138. ^{1}H NMR of the 3kb (400 MHz, $CDCl_3$)

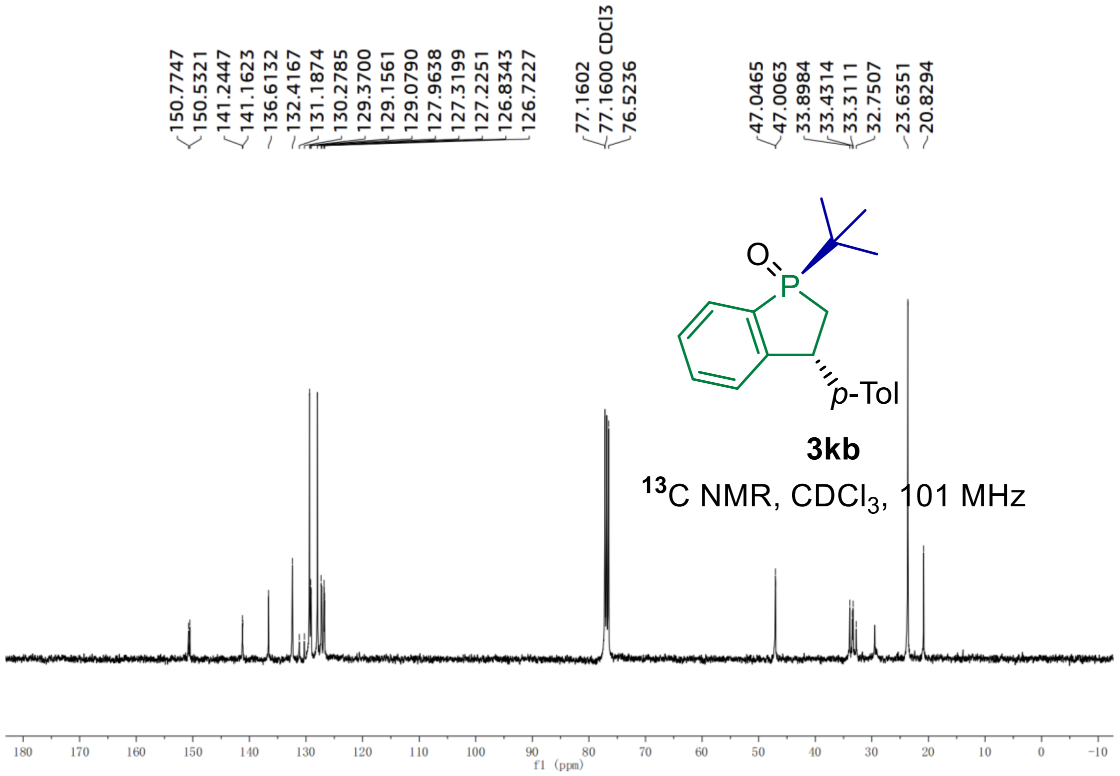

Supplementary Figure 139. ^{13}C NMR of the 3kb (101 MHz, $CDCl_3$)

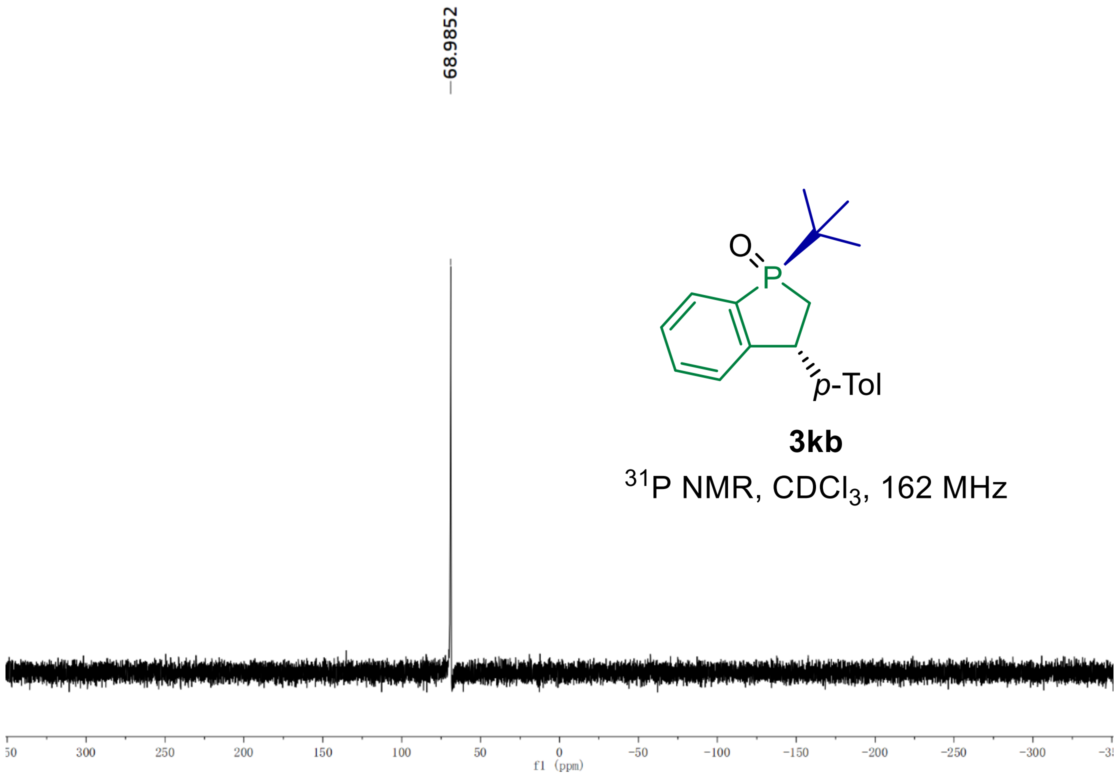

Supplementary Figure 140. ^{31}P NMR of the 3kb (162 MHz, $CDCl_3$)

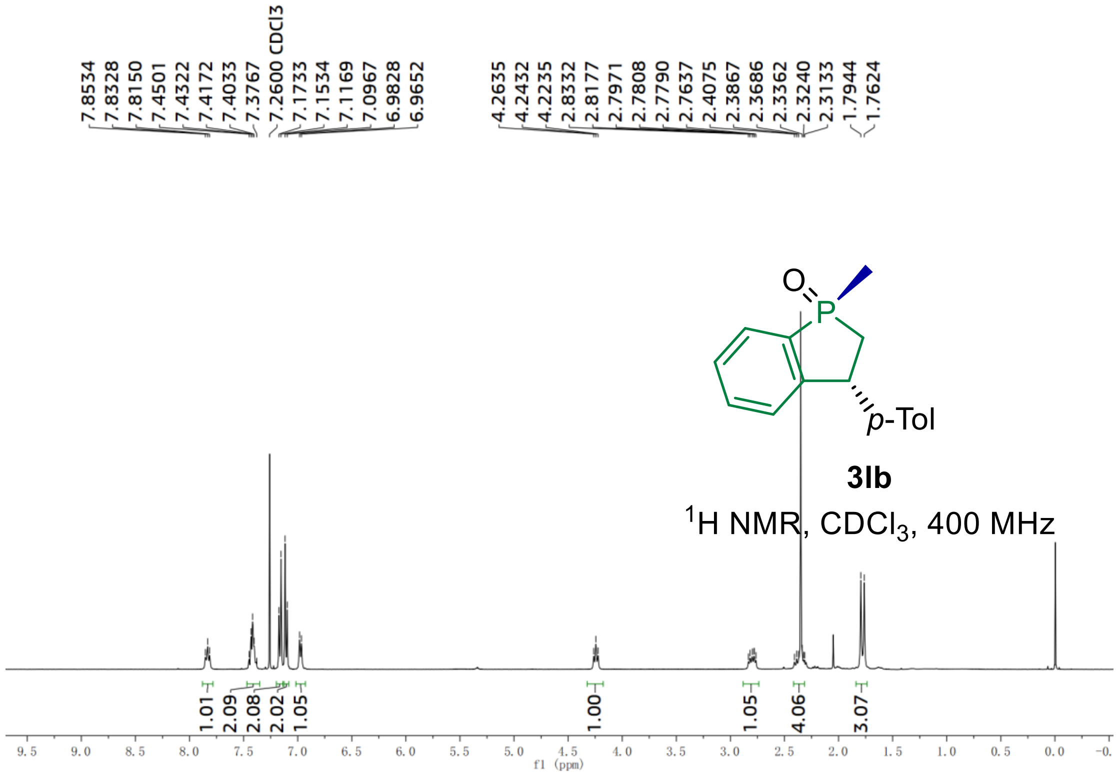

Supplementary Figure 141. ^{1}H NMR of the 3lb (400 MHz, $CDCl_3$)

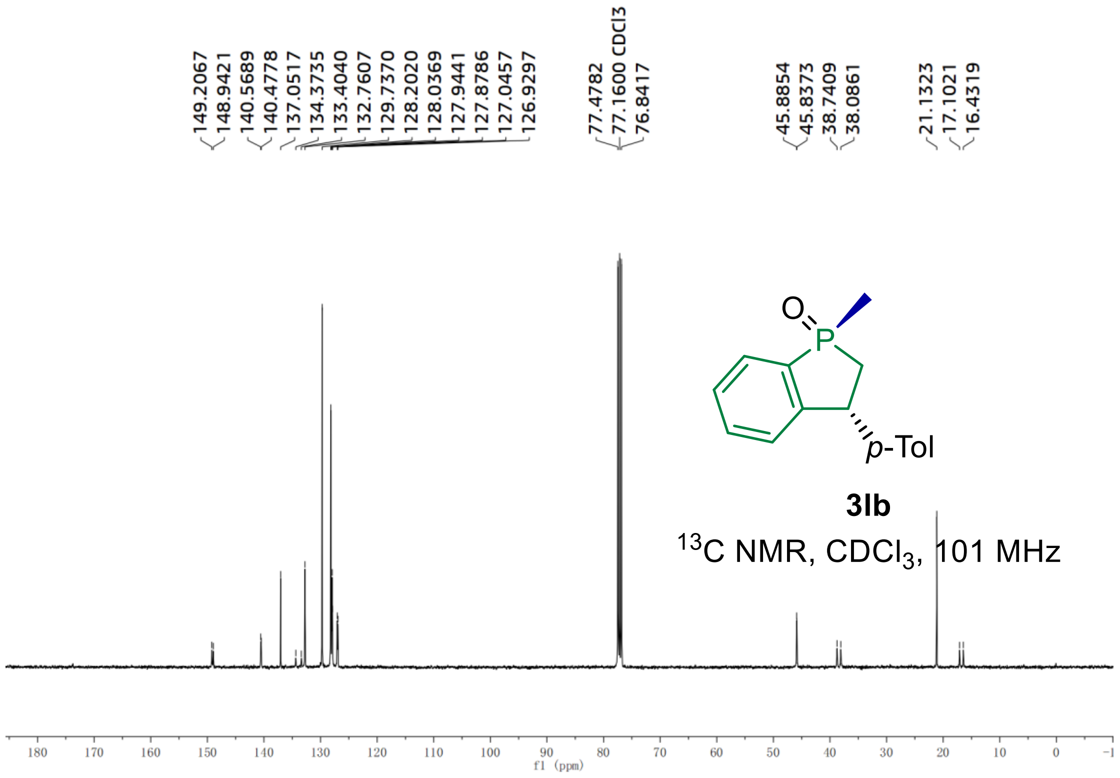

Supplementary Figure 142. ^{13}**C NMR of the 3lb (101 MHz,** $CDCl_3$**)**

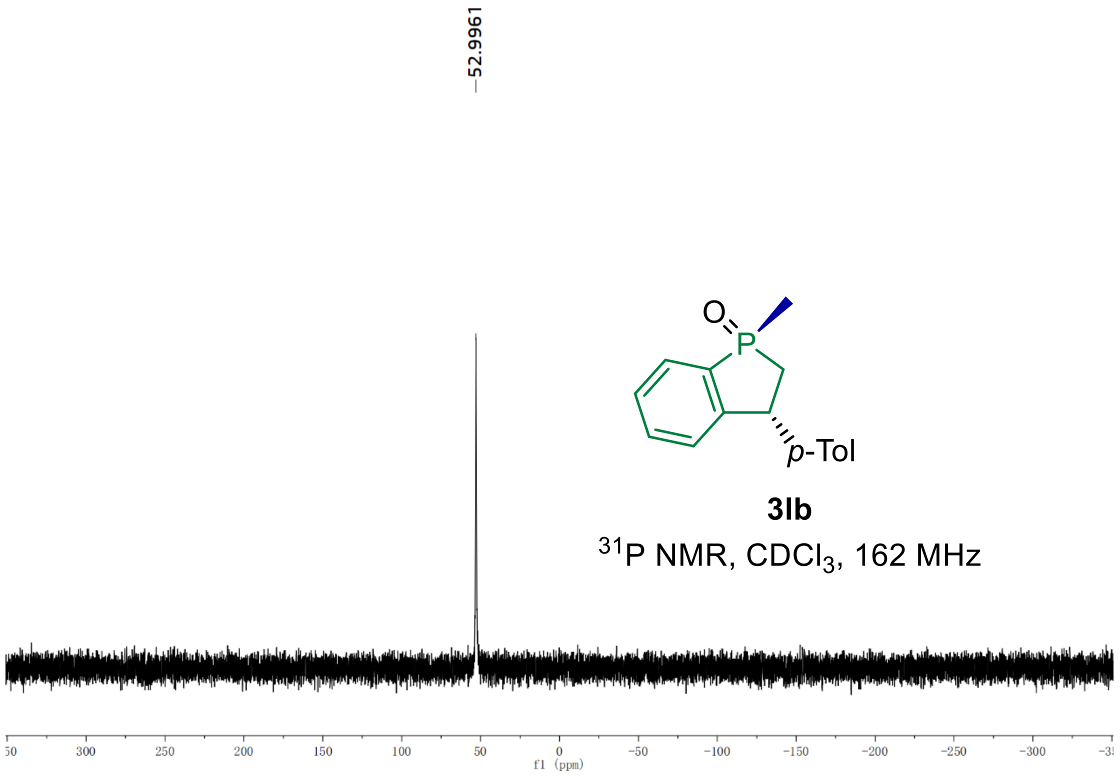

Supplementary Figure 143. ^{31}**P NMR of the 3lb (162 MHz,** $CDCl_3$**)**

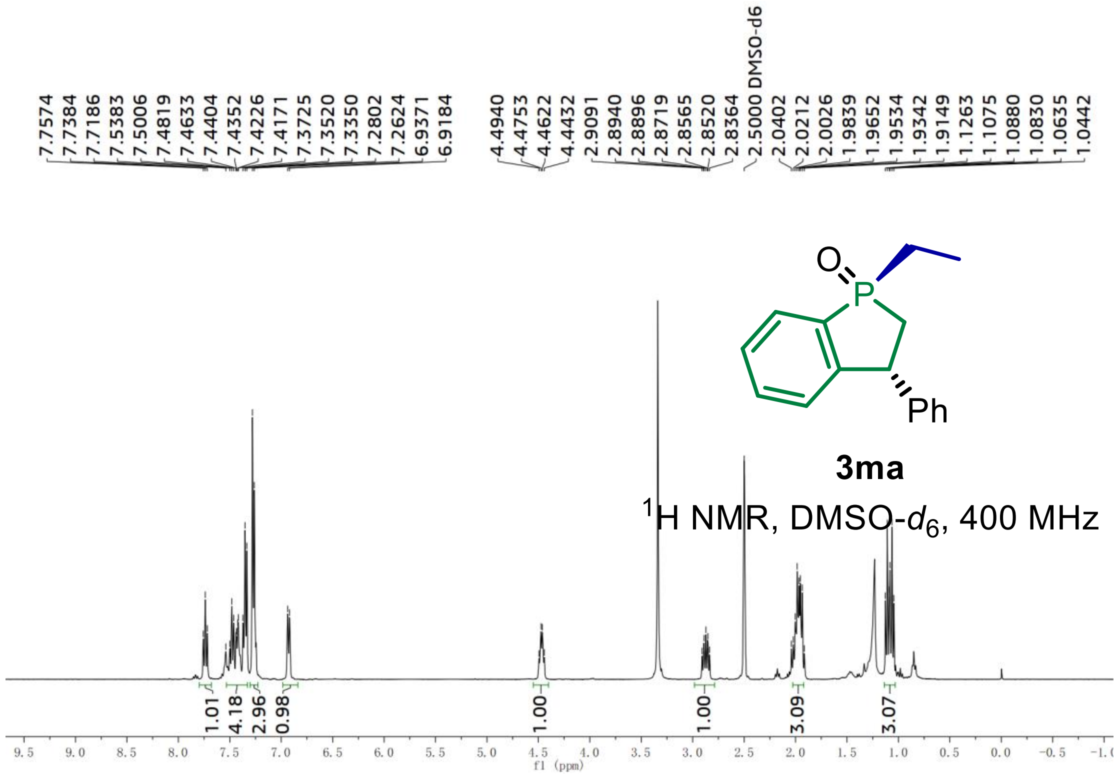

Supplementary Figure 144. ^{1}H NMR of the 3ma (400 MHz, DMSO-d_6)

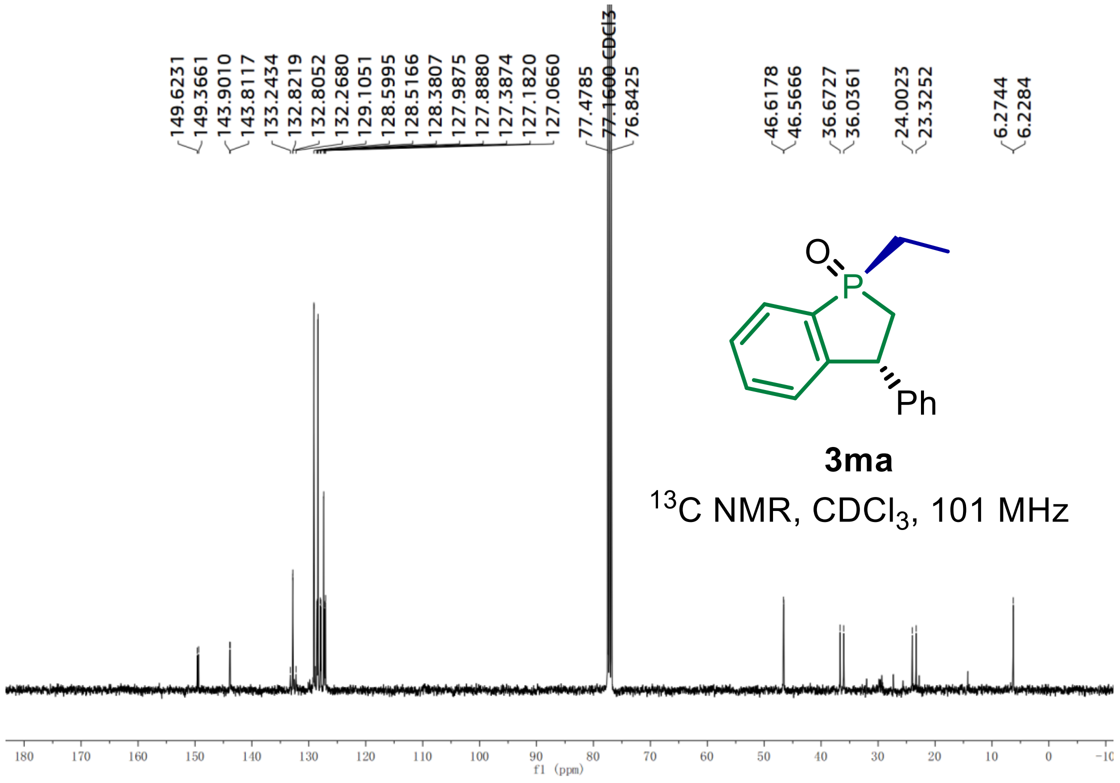

Supplementary Figure 145. ^{13}C NMR of the 3ma (101 MHz, $CDCl_3$)

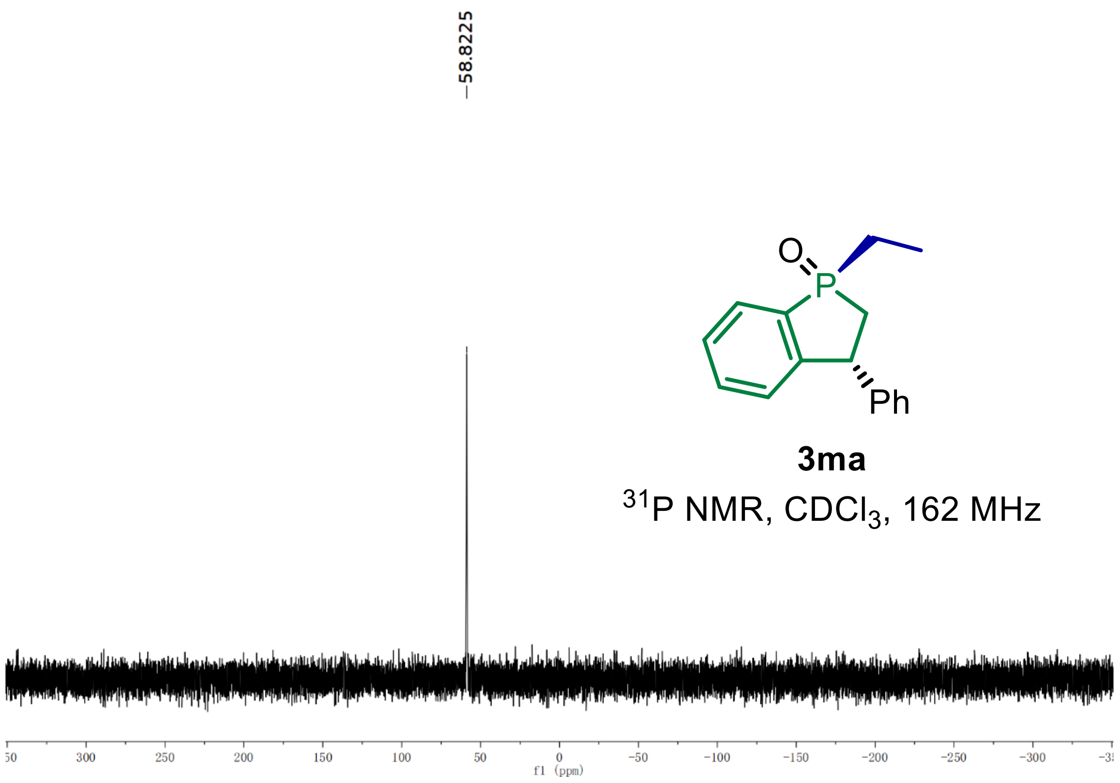

Supplementary Figure 146. ^{31}P NMR of the 3ma (162 MHz, $CDCl_3$)

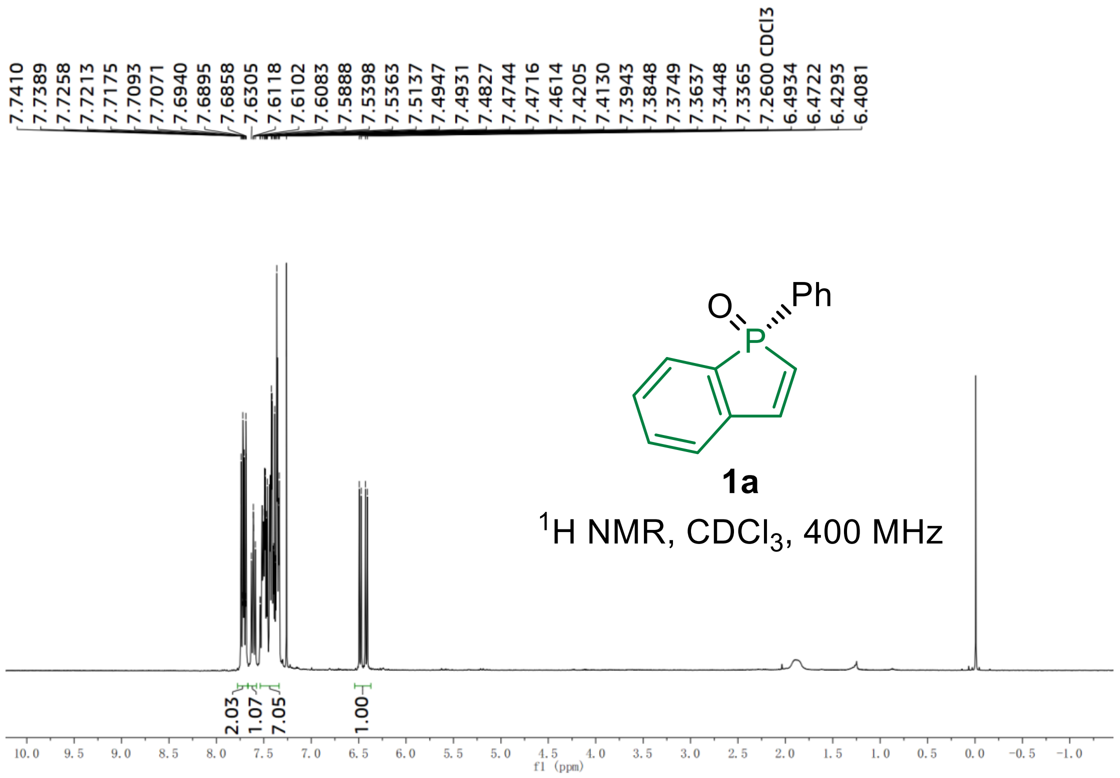

Supplementary Figure 147. ^{1}H NMR of the 1a (400 MHz, $CDCl_3$)

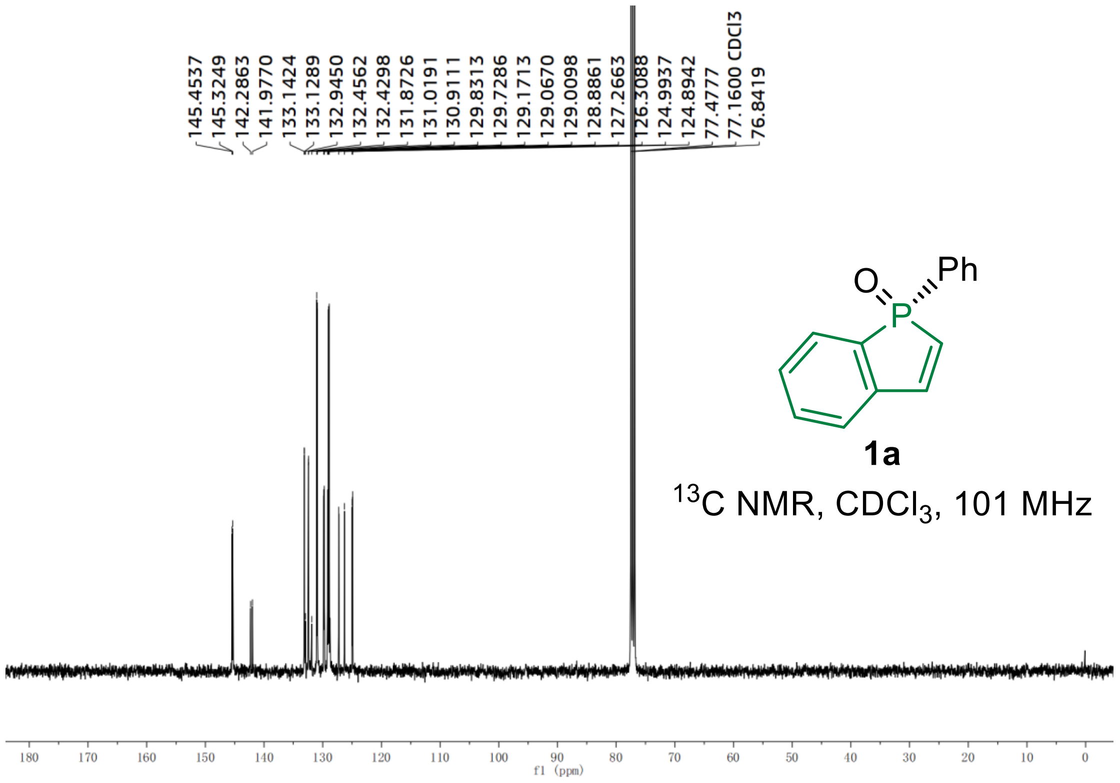

Supplementary Figure 148. ^{13}C NMR of the 1a (101 MHz, $CDCl_3$)

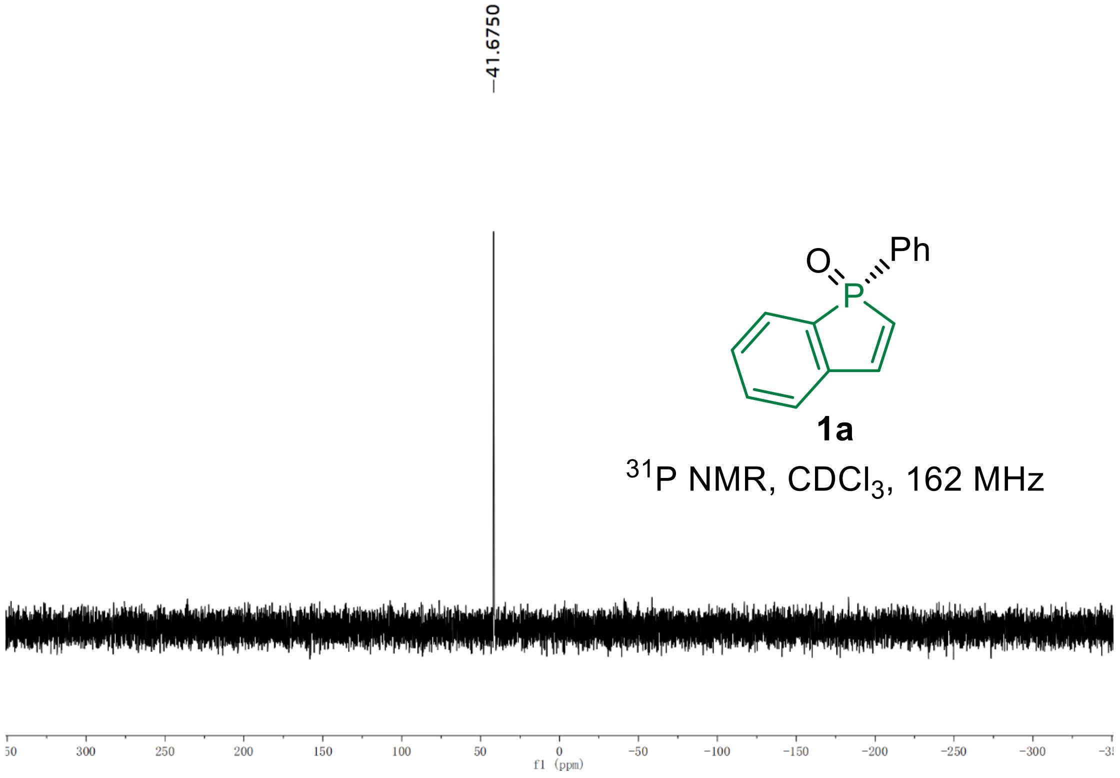

Supplementary Figure 149. ^{31}P NMR of the 1a (162 MHz, $CDCl_3$)

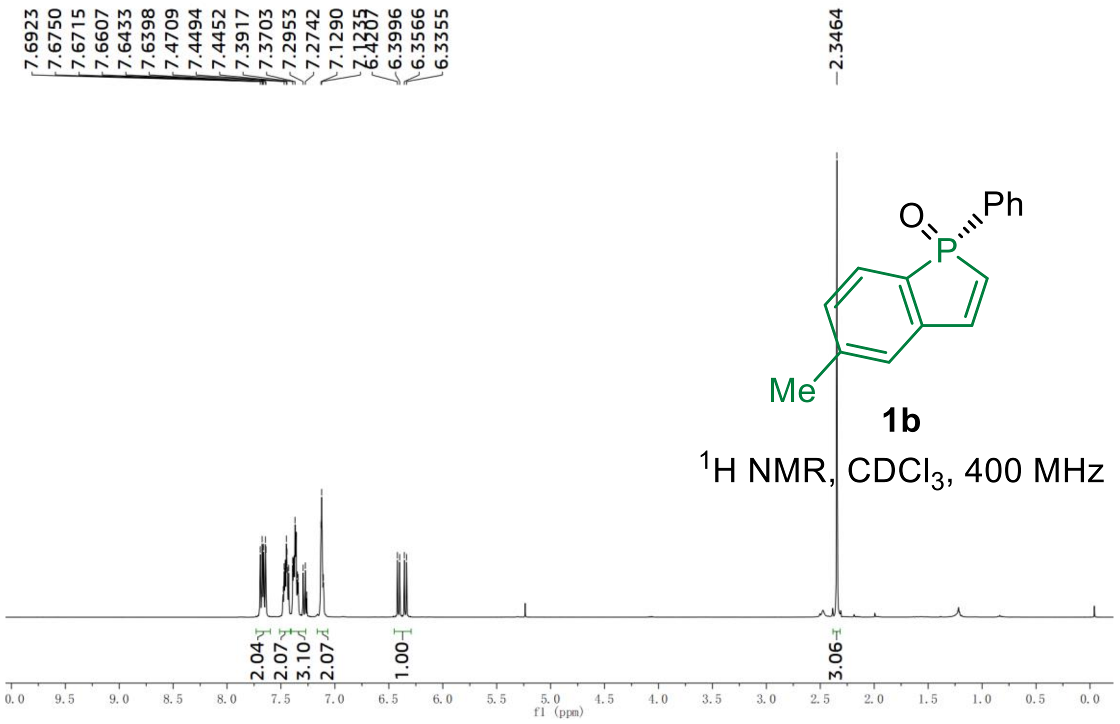

Supplementary Figure 150. ^{1}**H NMR of the 1b (400 MHz, CDCl$_3$)**

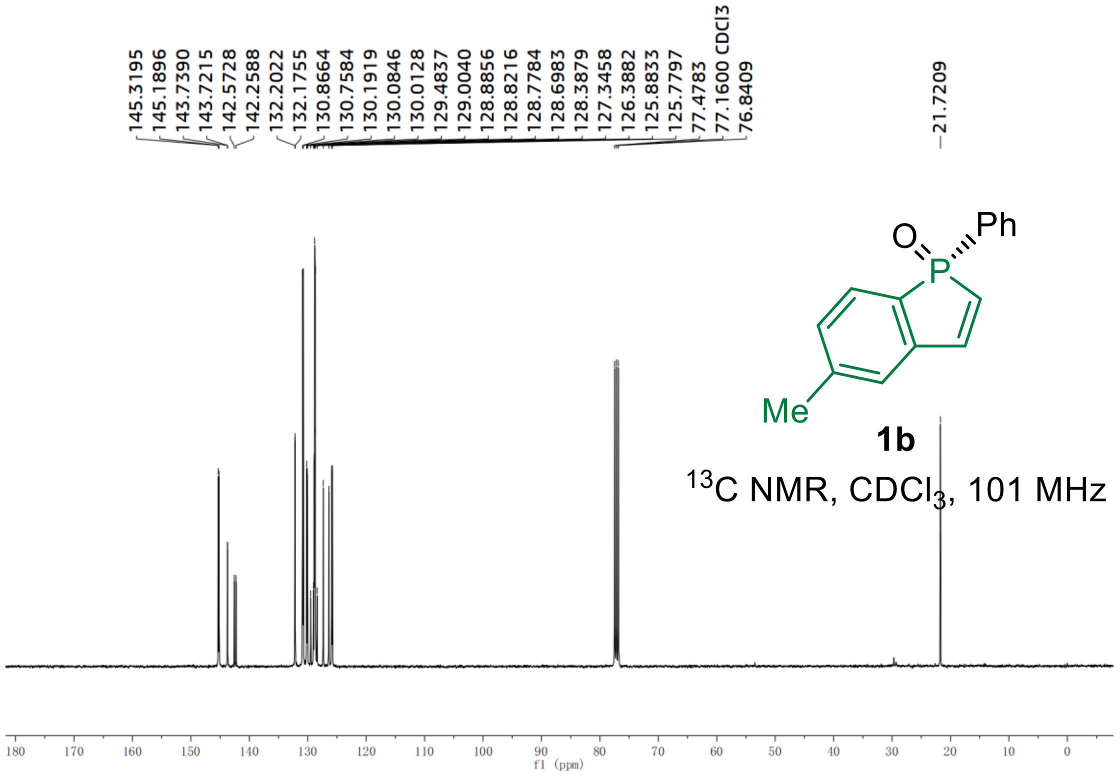

Supplementary Figure 151. ^{1}**H NMR of the 1b (101 MHz, CDCl$_3$)**

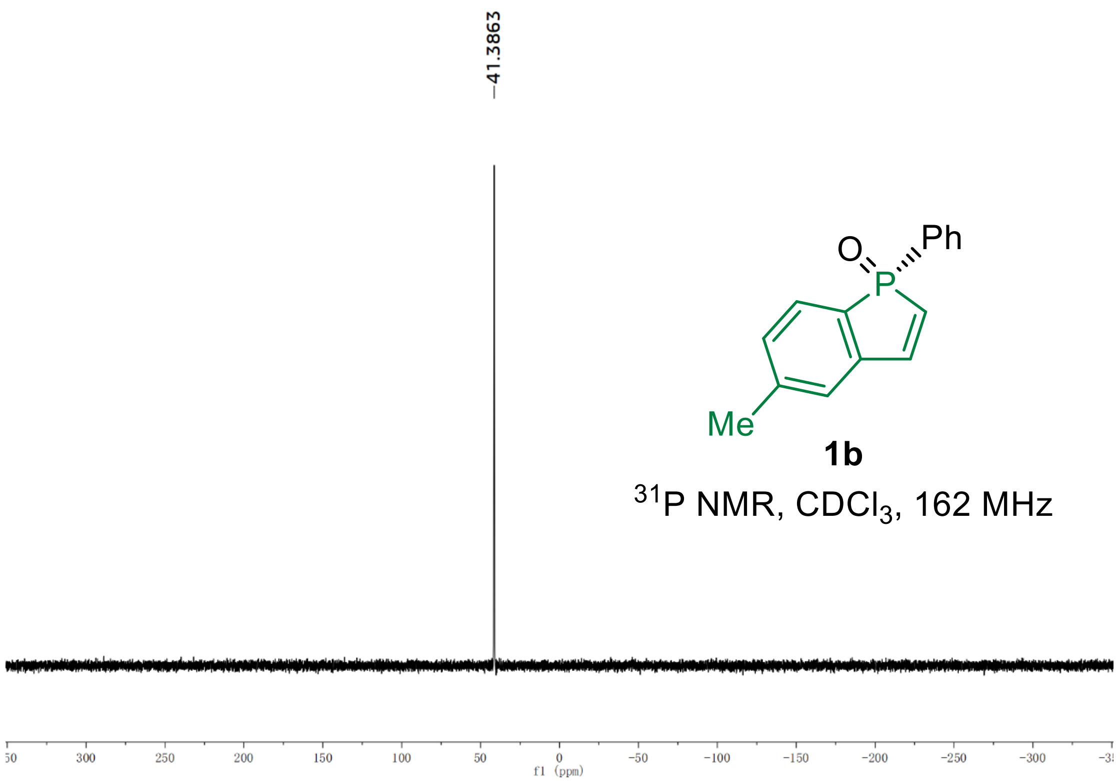

Supplementary Figure 152. ^{31}P NMR of the 1b (162 MHz, $CDCl_3$)

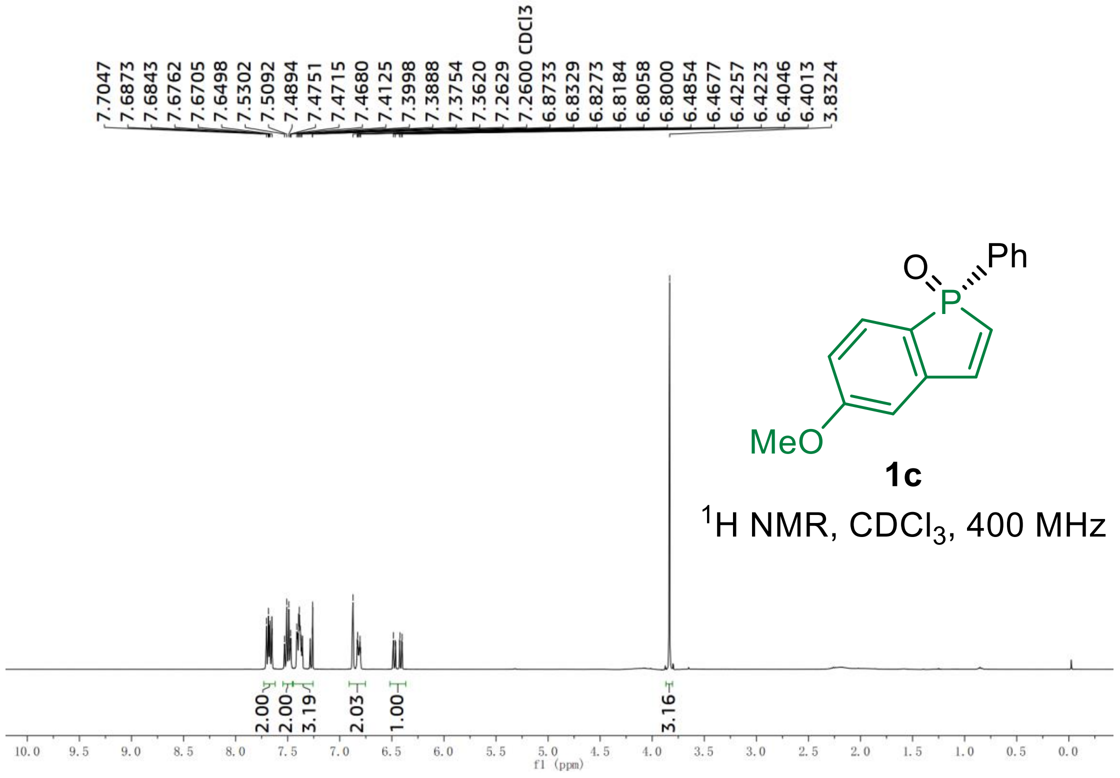

Supplementary Figure 153. ^{1}H NMR of the 1c (400 MHz, $CDCl_3$)

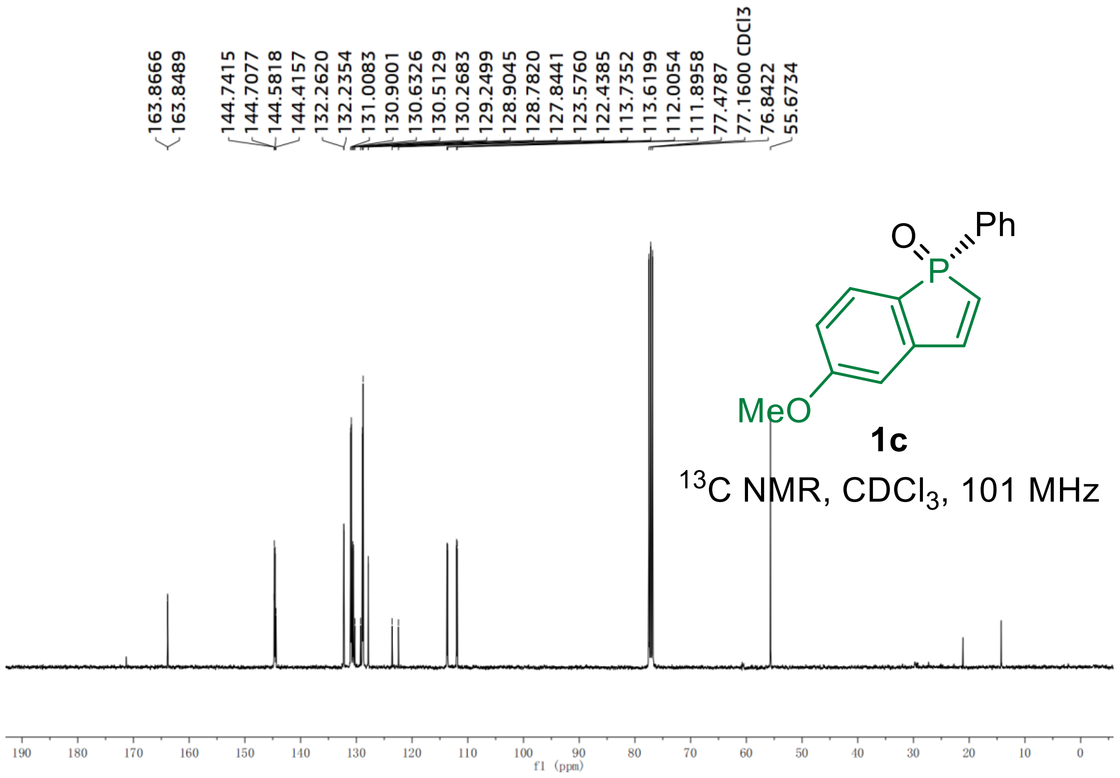

Supplementary Figure 154. ^{13}**C NMR of the 1c (101 MHz, $CDCl_3$)**

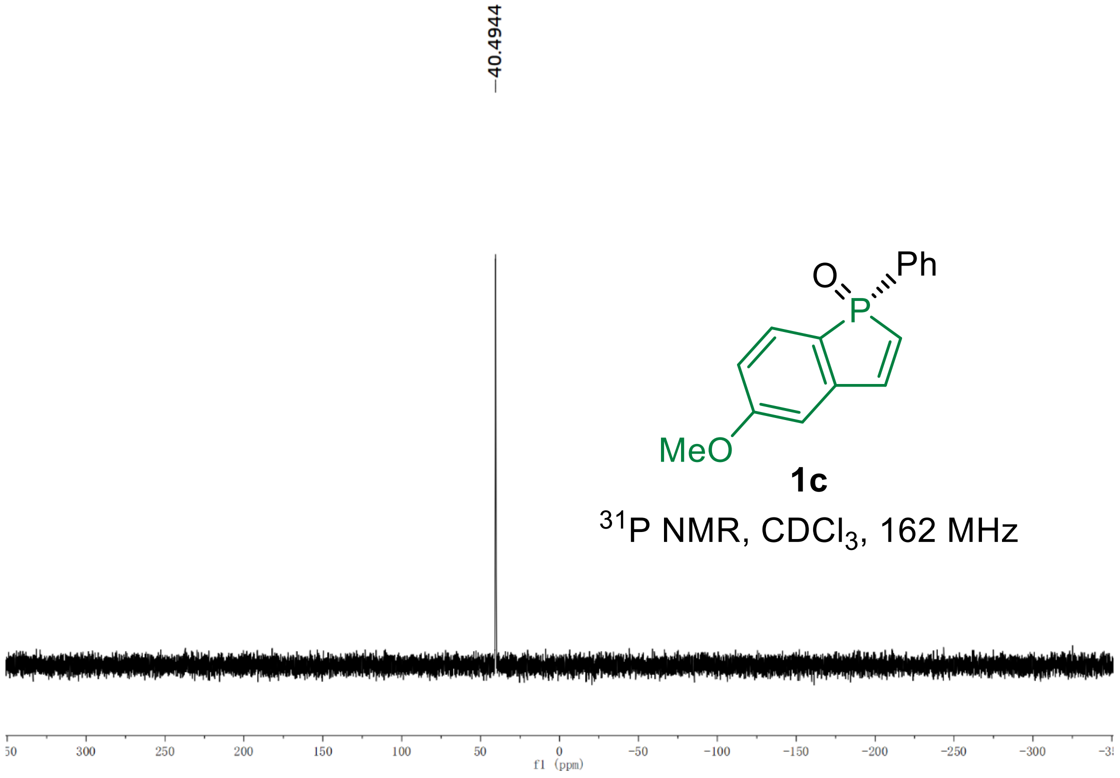

Supplementary Figure 155. ^{31}**P NMR of the 1c (162 MHz, $CDCl_3$)**

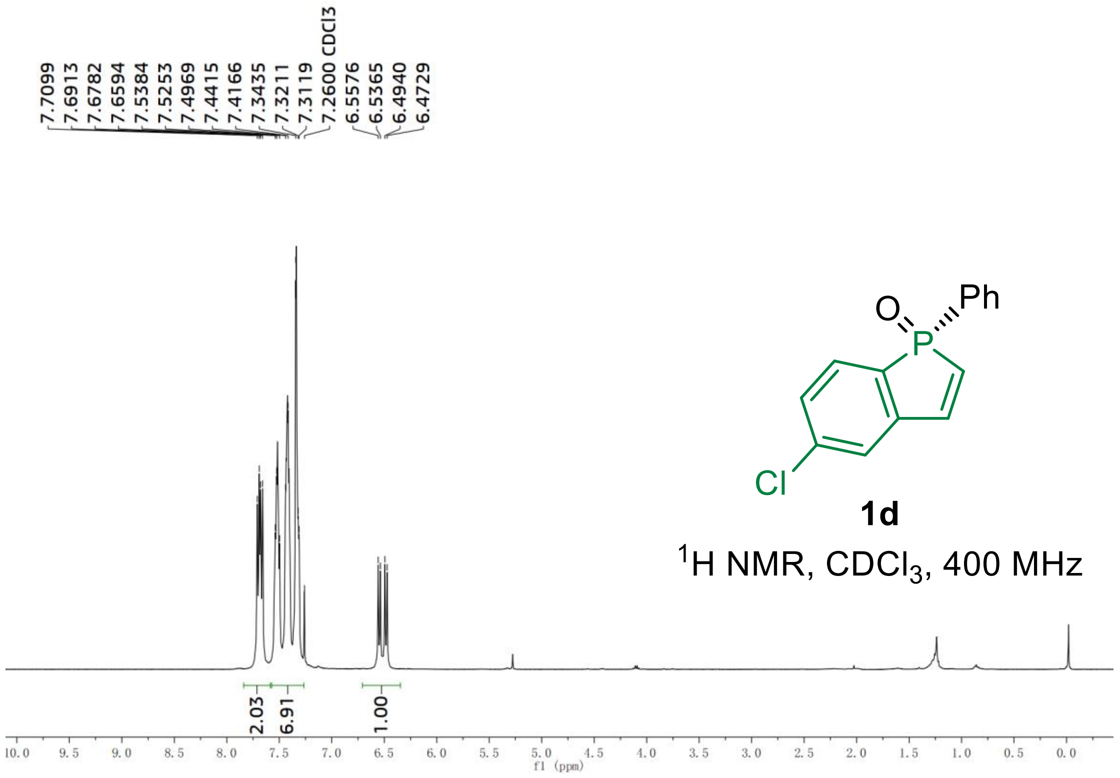

Supplementary Figure 156. ^{1}**H NMR of the 1d (400 MHz,** $CDCl_3$**)**

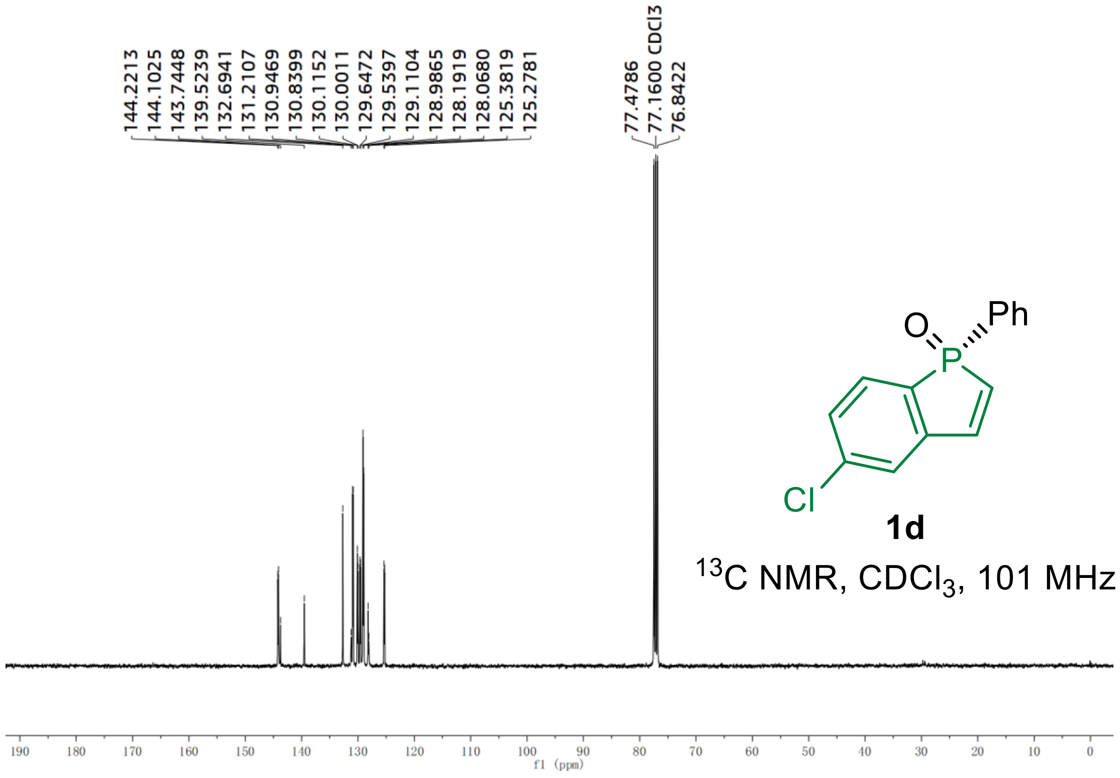

Supplementary Figure 157. ^{13}**C NMR of the 1d (101 MHz,** $CDCl_3$**)**

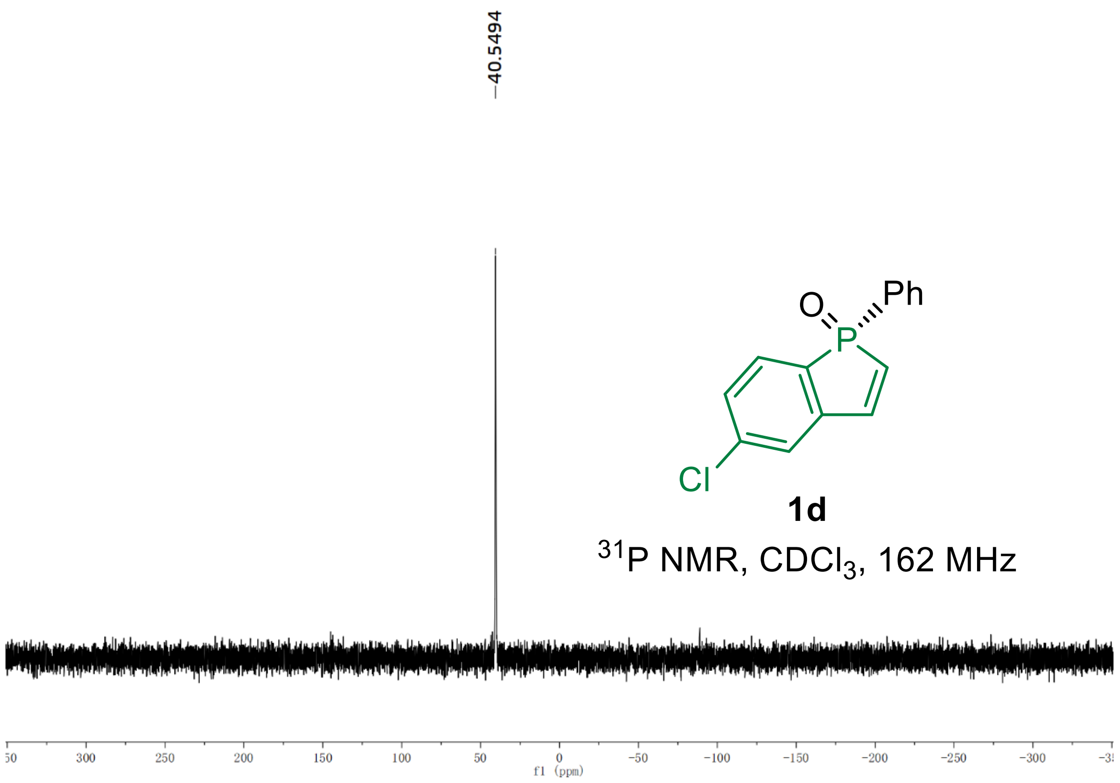

Supplementary Figure 158. ^{31}P NMR of the 1d (162 MHz, CDCl$_3$)

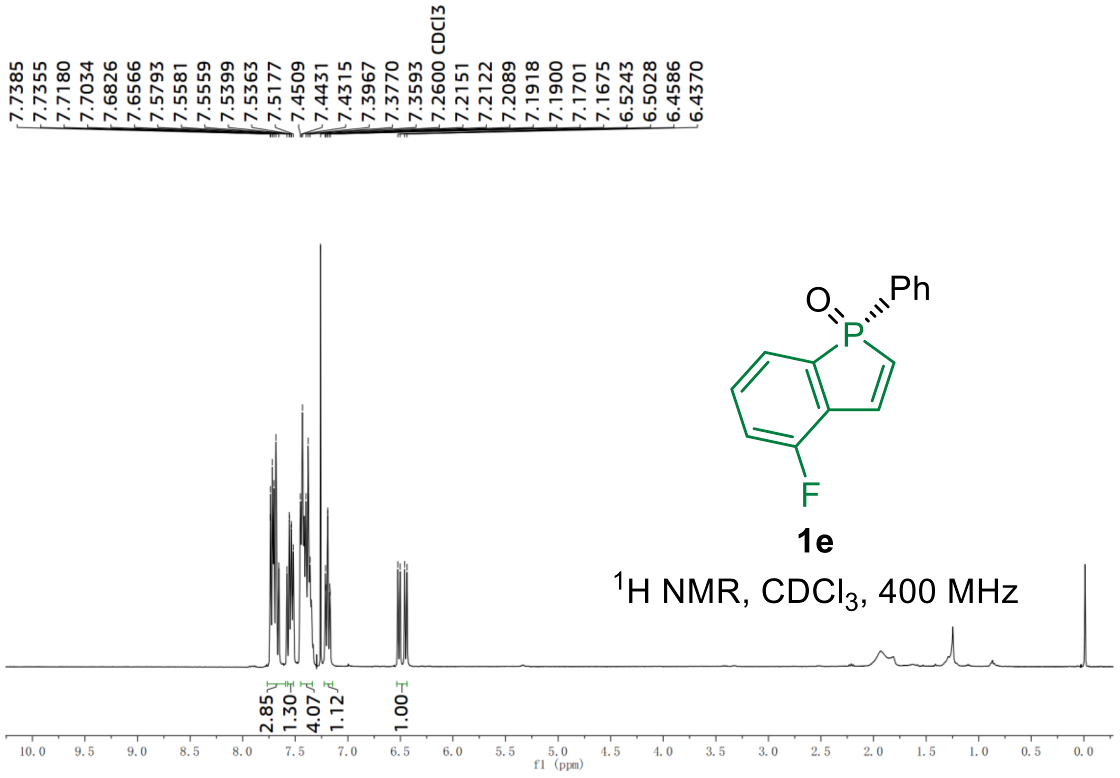

Supplementary Figure 159. ^{1}H NMR of the 1e (400 MHz, CDCl$_3$)

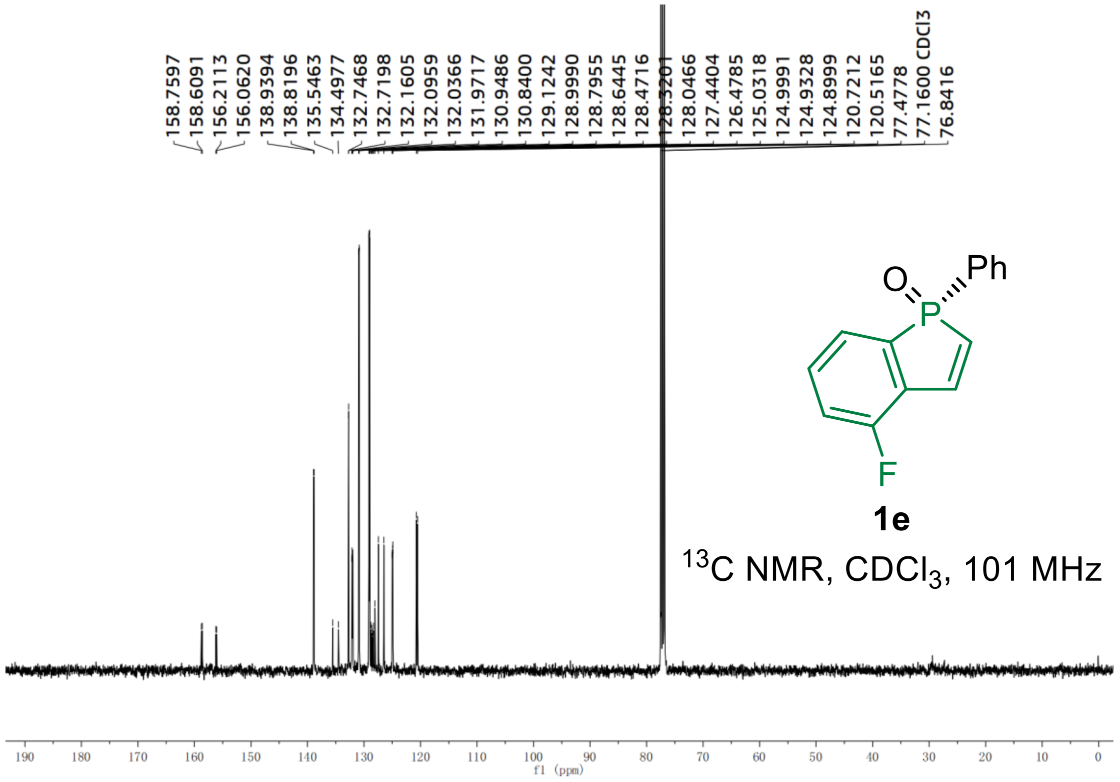

Supplementary Figure 160. ^{13}**C NMR of the 1e (101 MHz, CDCl$_3$)**

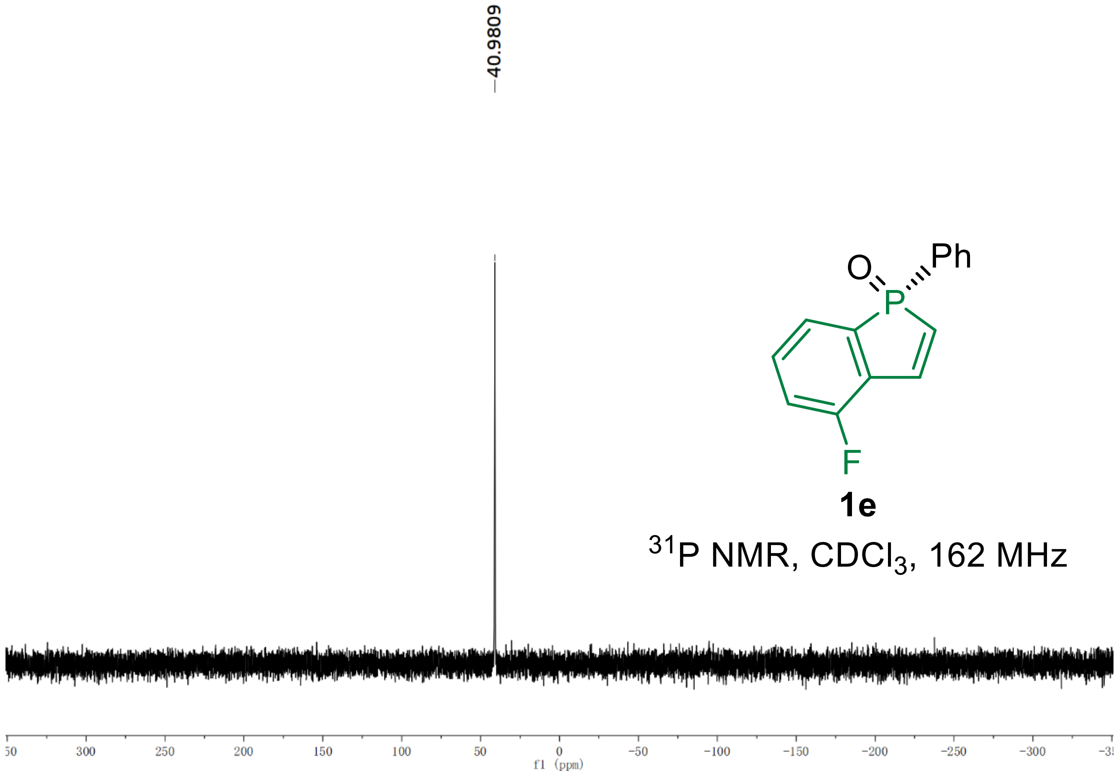

Supplementary Figure 161. ^{31}**P NMR of the 1e (162 MHz, CDCl$_3$)**

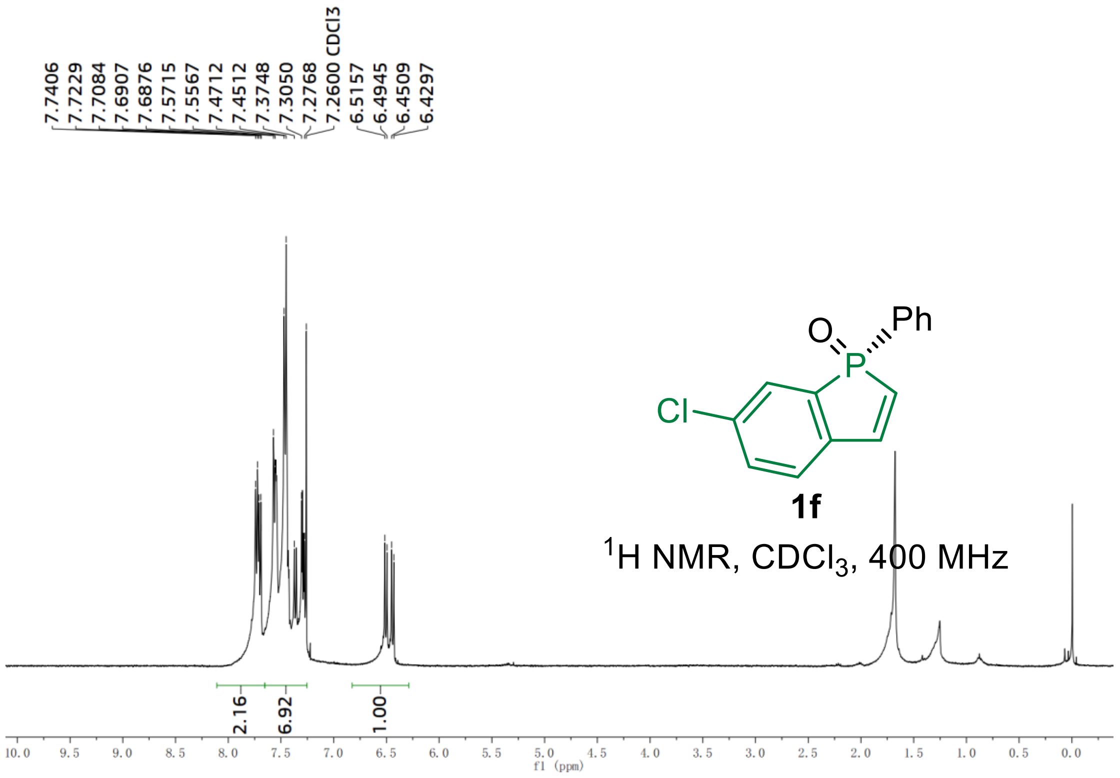

Supplementary Figure 162. ^{1}H NMR of the 1f (400 MHz, $CDCl_3$)

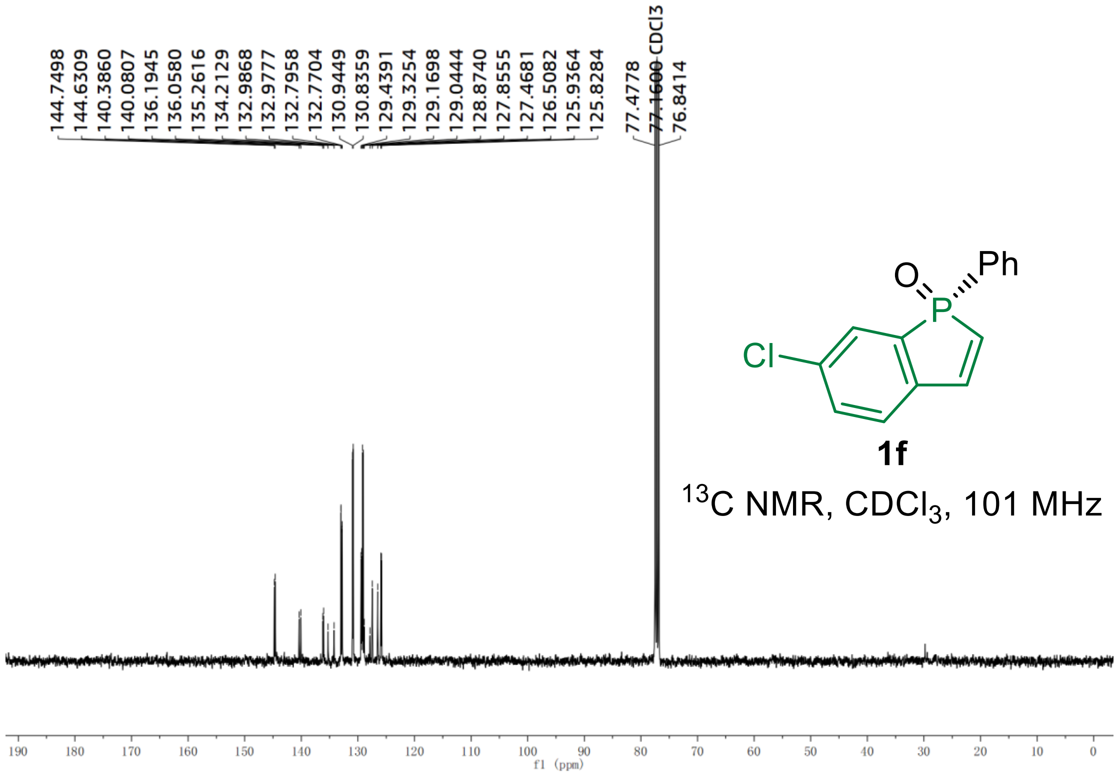

Supplementary Figure 163. ^{13}C NMR of the 1f (101 MHz, $CDCl_3$)

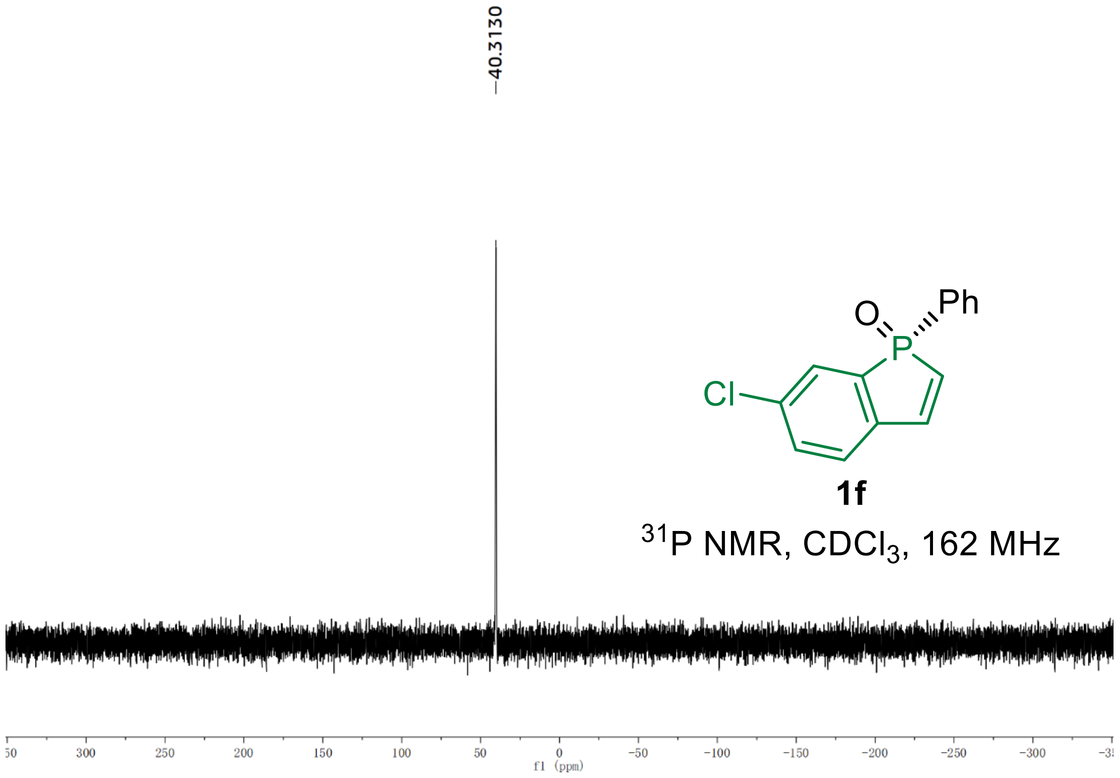

Supplementary Figure 164. ^{31}P NMR of the 1f (162 MHz, CDCl$_3$)

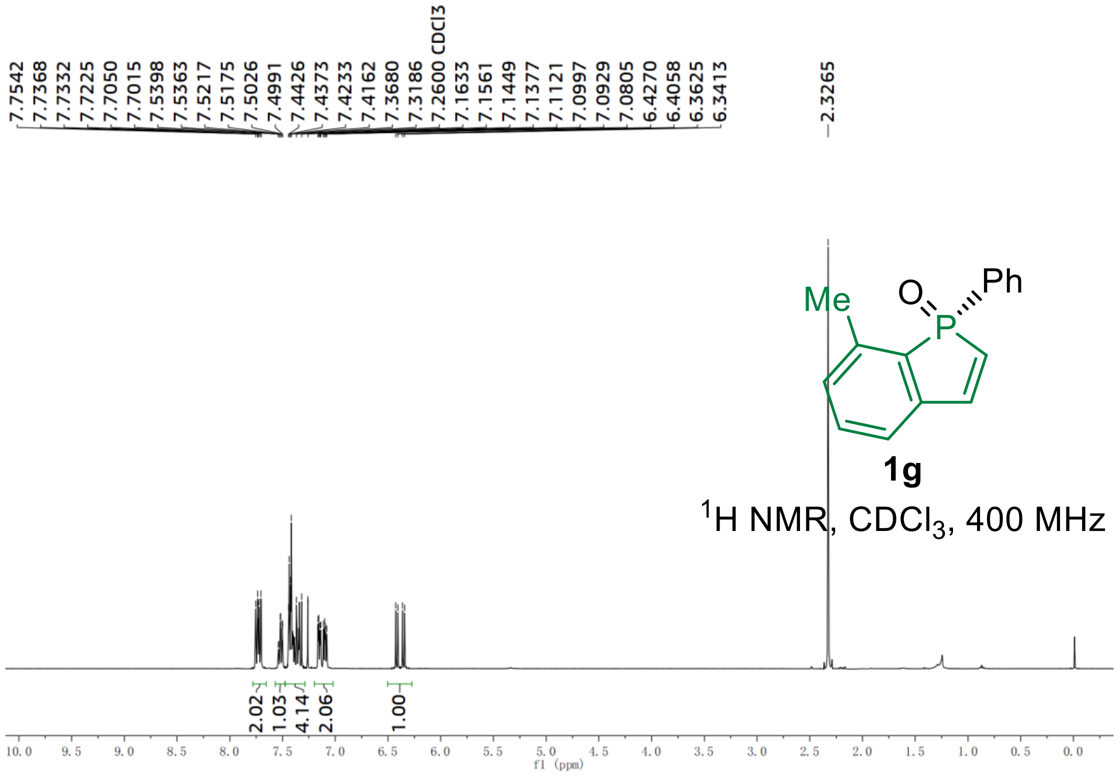

Supplementary Figure 165. ^{1}H NMR of the 1g (400 MHz, CDCl$_3$)

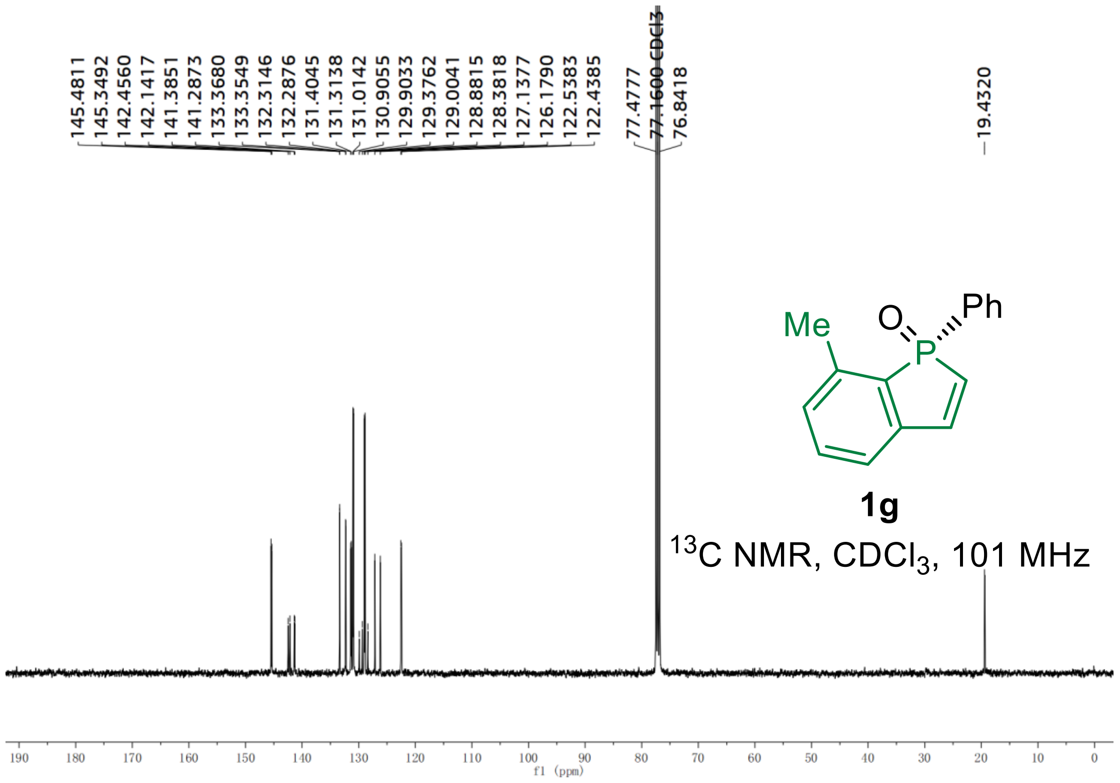

Supplementary Figure 166. ^{13}C NMR of the 1g (101 MHz, $CDCl_3$)

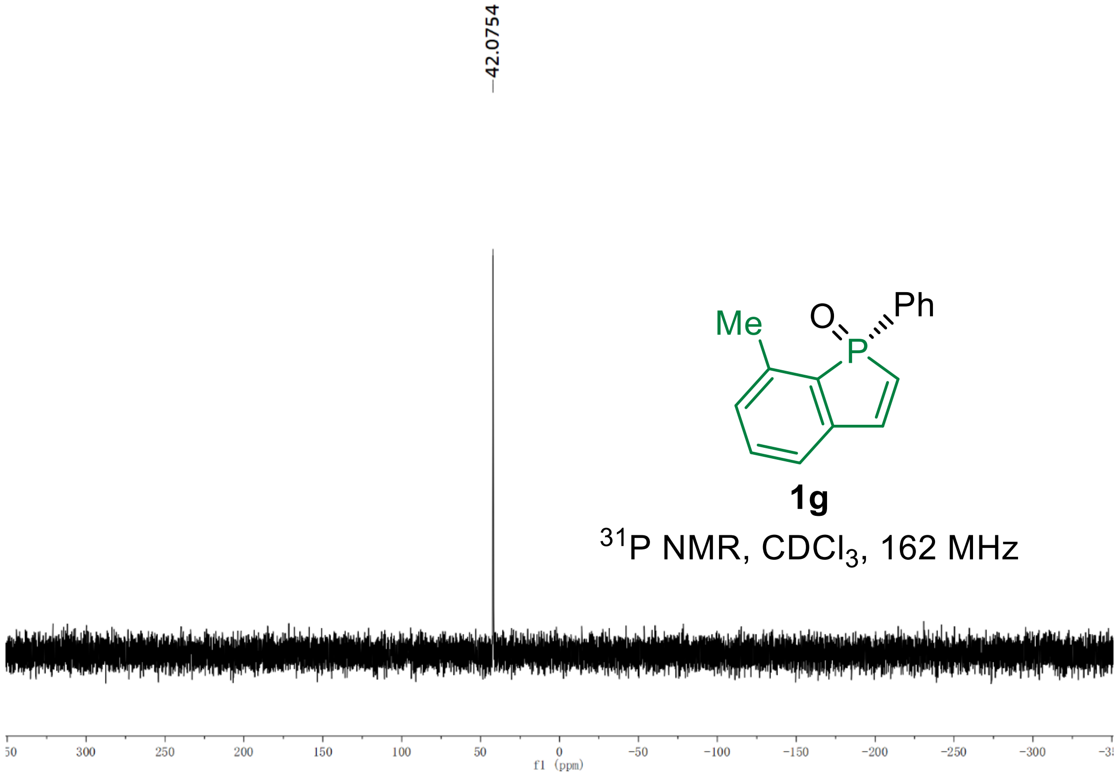

Supplementary Figure 167. ^{31}P NMR of the 1g (162 MHz, $CDCl_3$)

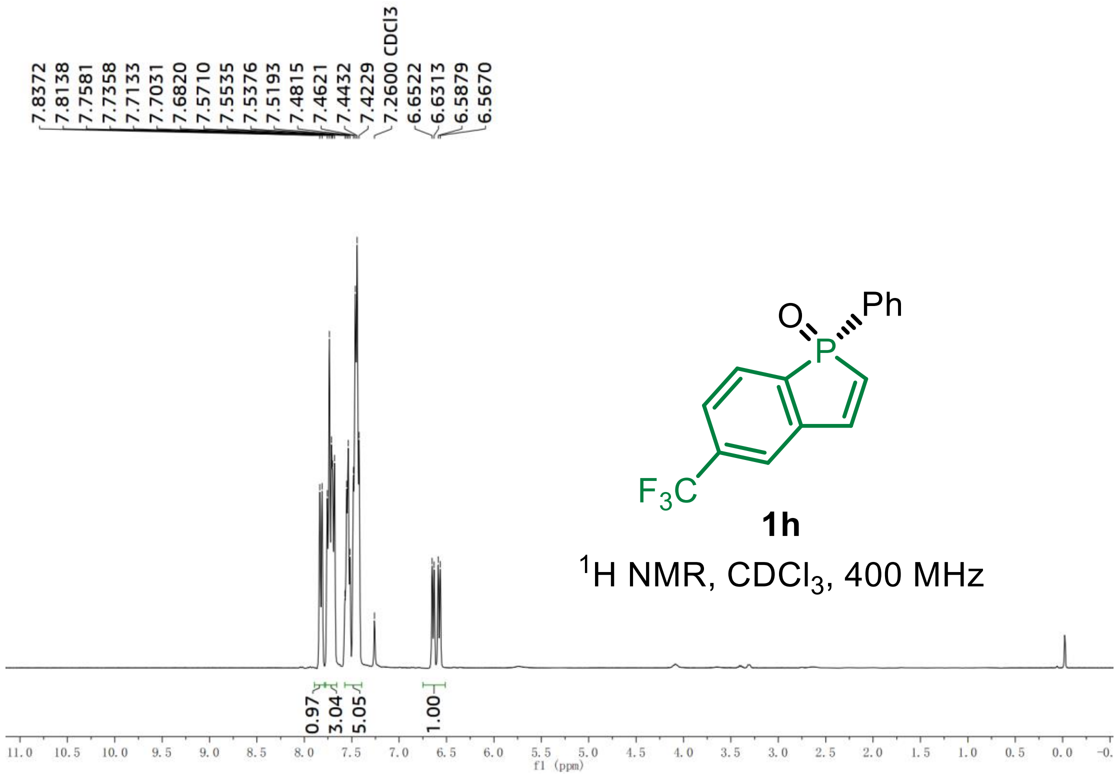

Supplementary Figure 168. ^{1}H NMR of the 1h (400 MHz, $CDCl_3$)

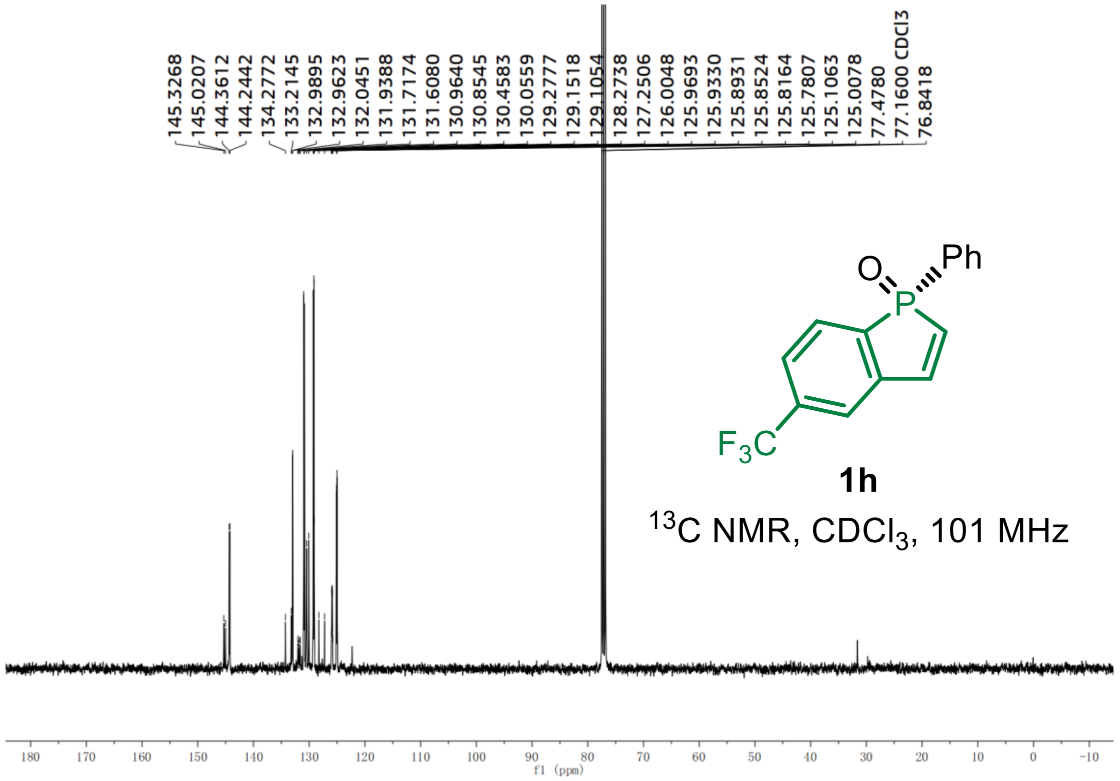

Supplementary Figure 169. ^{13}C NMR of the 1h (101 MHz, $CDCl_3$)

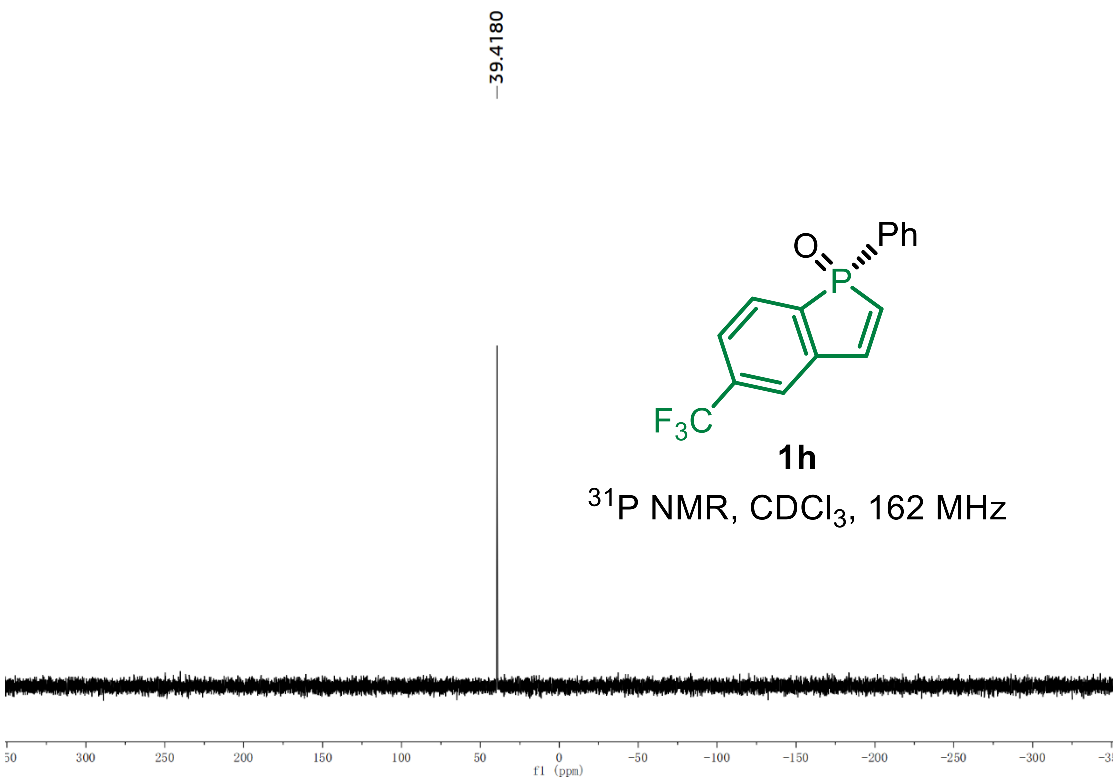

Supplementary Figure 170. ^{31}P NMR of the 1h (162 MHz, CDCl$_3$)

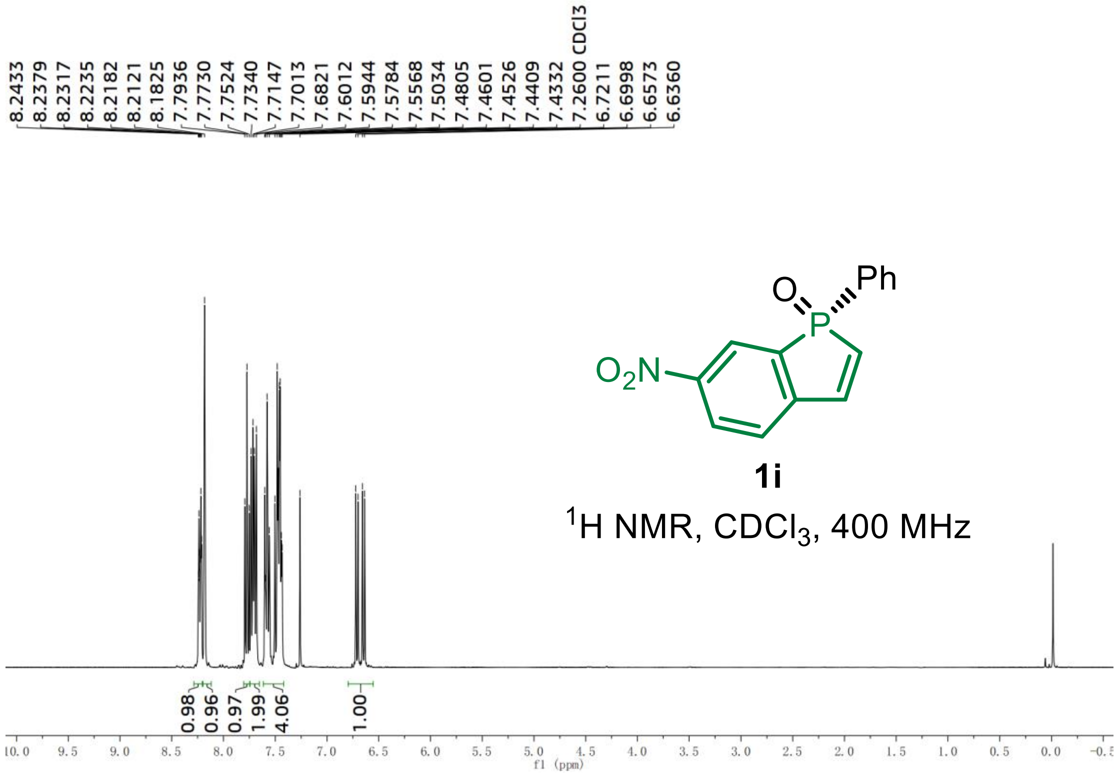

Supplementary Figure 171. ^{1}H NMR of the 1i (400 MHz, CDCl$_3$)

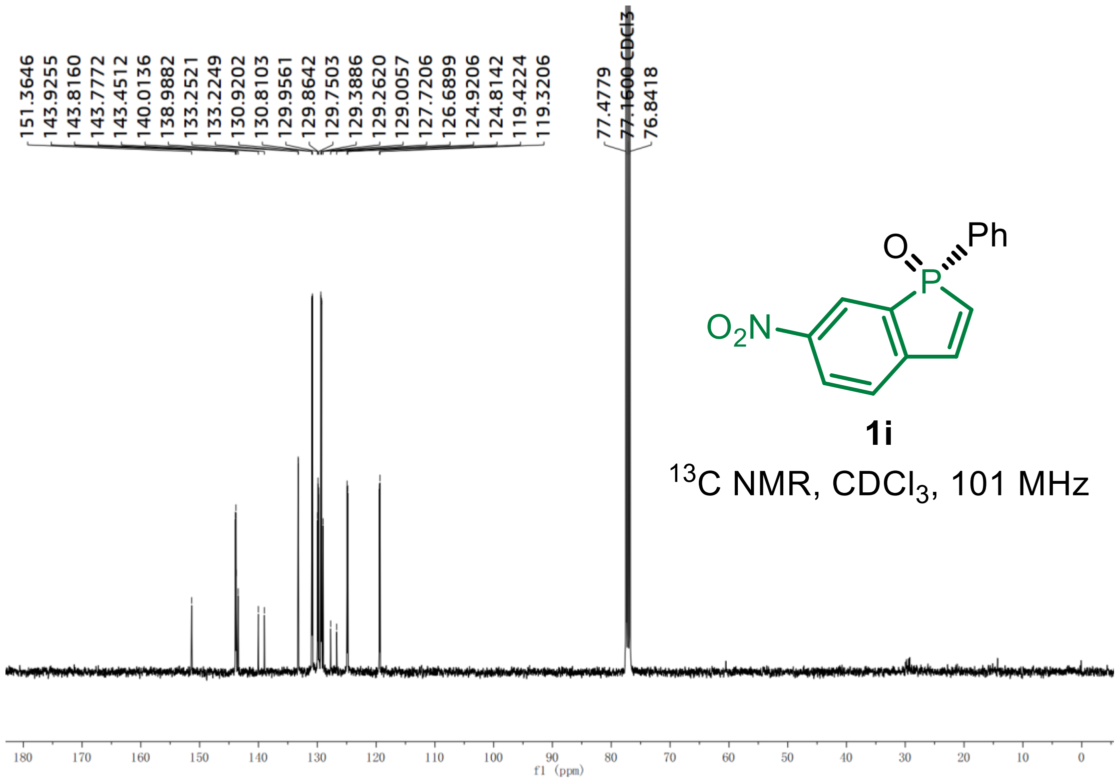

Supplementary Figure 172. ^{13}C NMR of the 1i (101 MHz, $CDCl_3$)

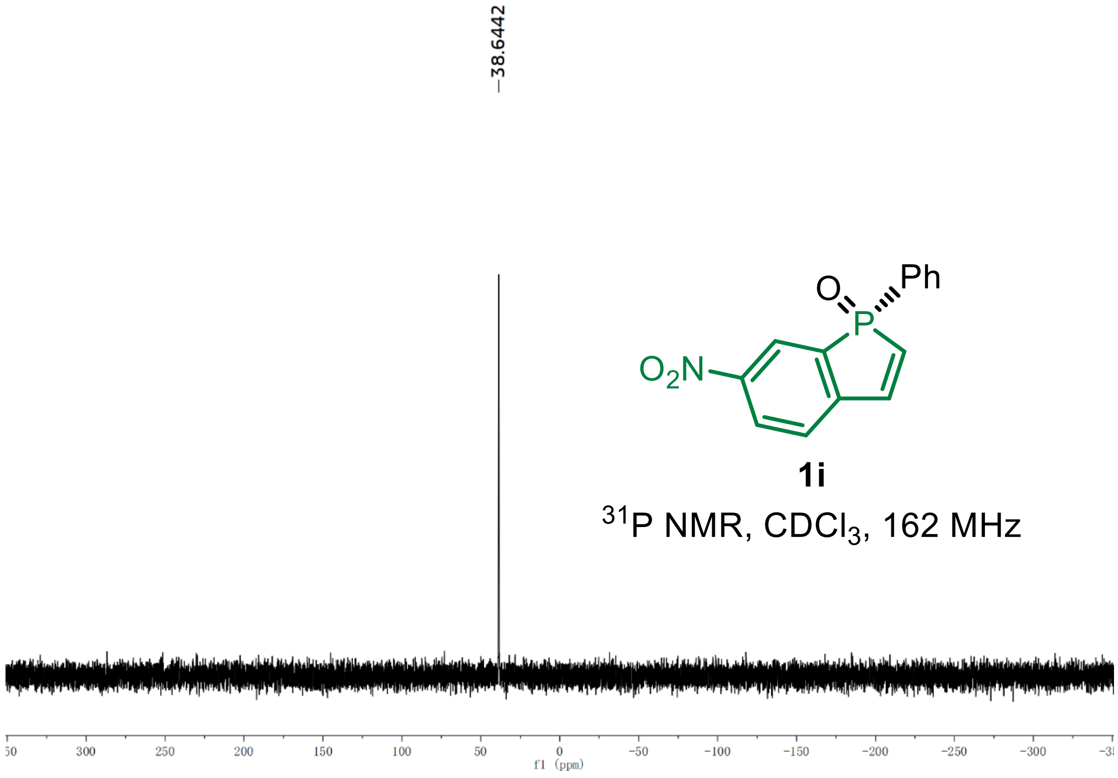

Supplementary Figure 173. ^{31}P NMR of the 1i (162 MHz, $CDCl_3$)

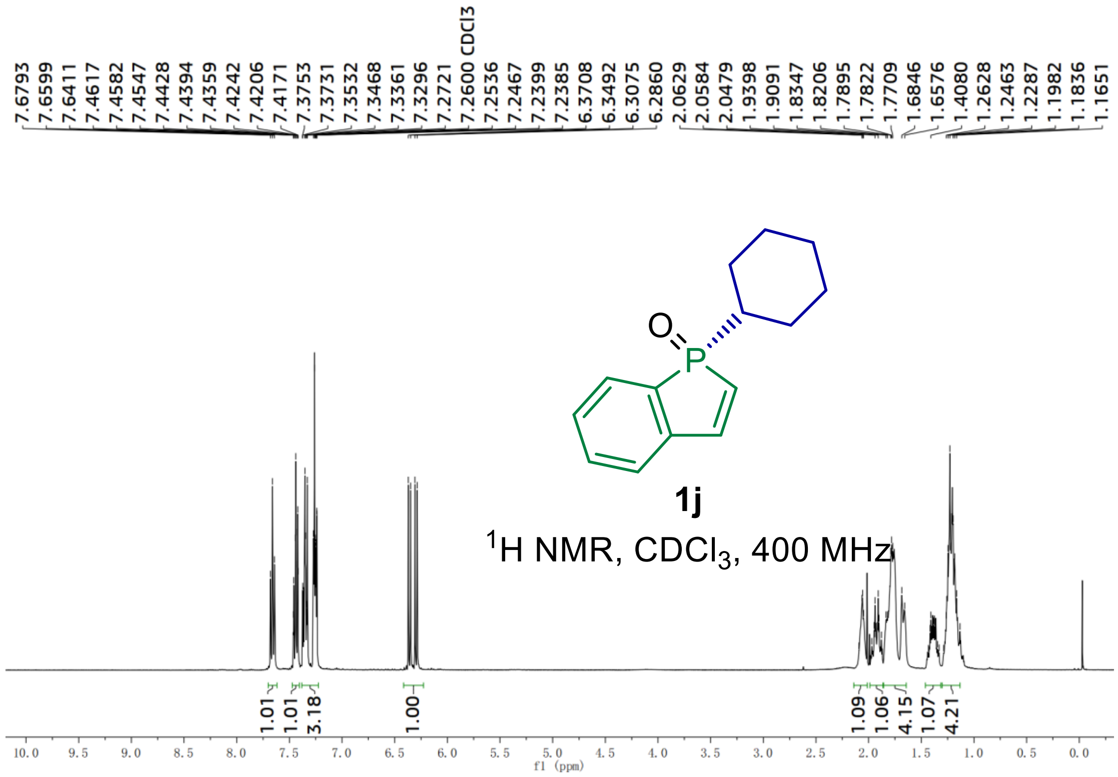

Supplementary Figure 174. ^{1}**H NMR of the 1j (400 MHz, CDCl$_3$)**

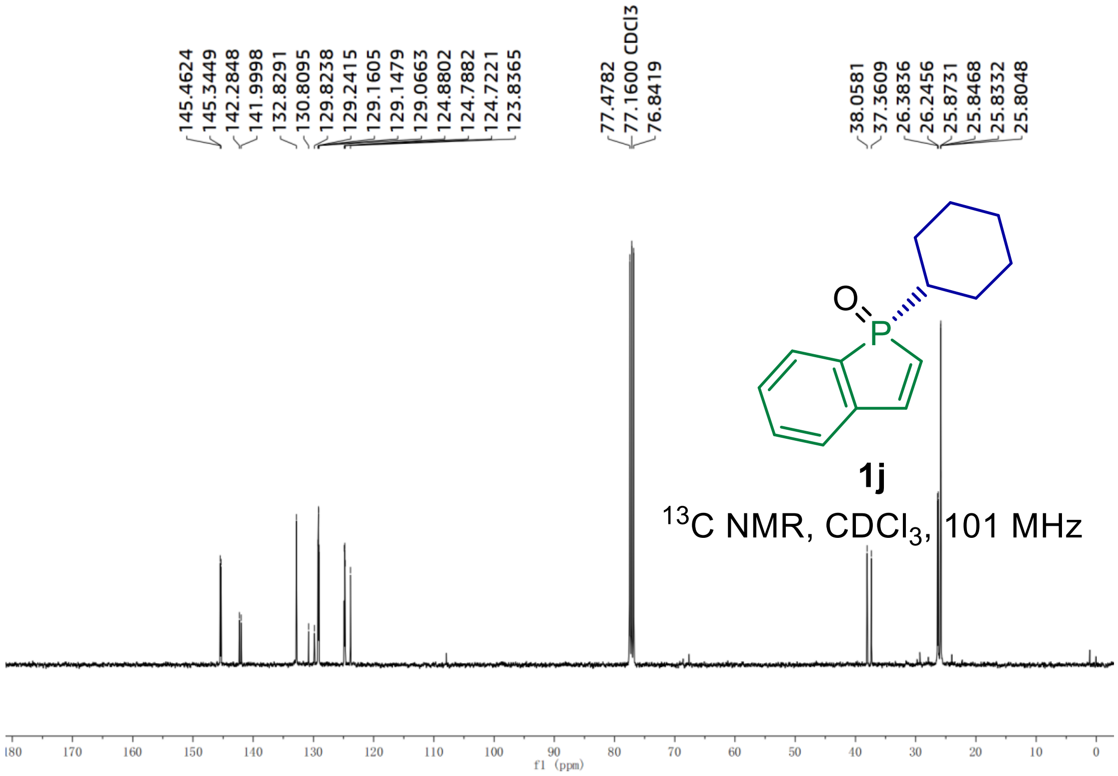

Supplementary Figure 175. ^{13}**C NMR of the 1j (101 MHz, CDCl$_3$)**

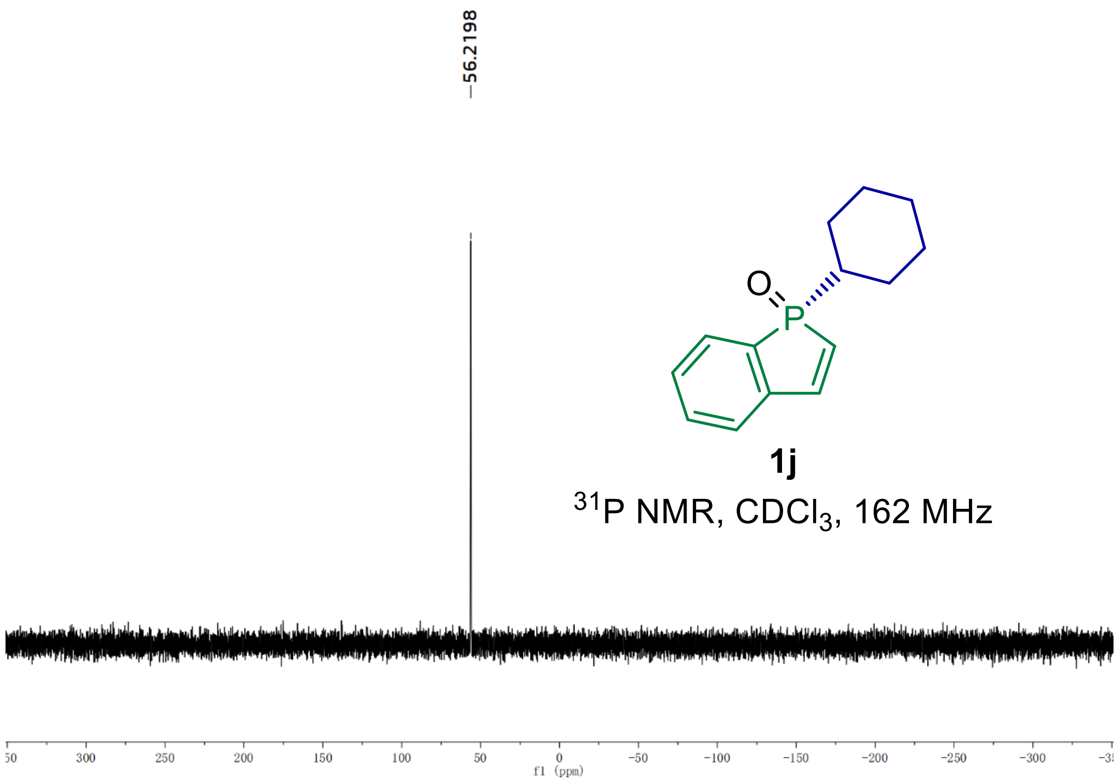

Supplementary Figure 176. ^{31}P NMR of the 1j (162 MHz, CDCl$_3$)

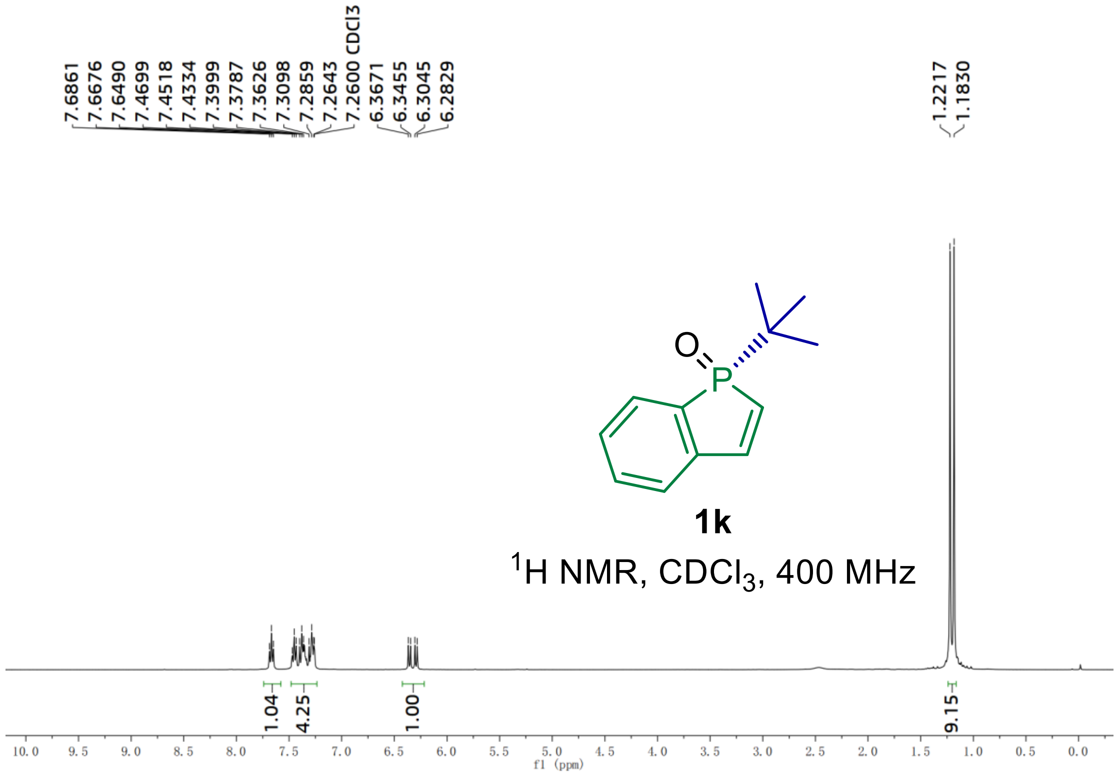

Supplementary Figure 177. ^{1}H NMR of the 1k (400 MHz, CDCl$_3$)

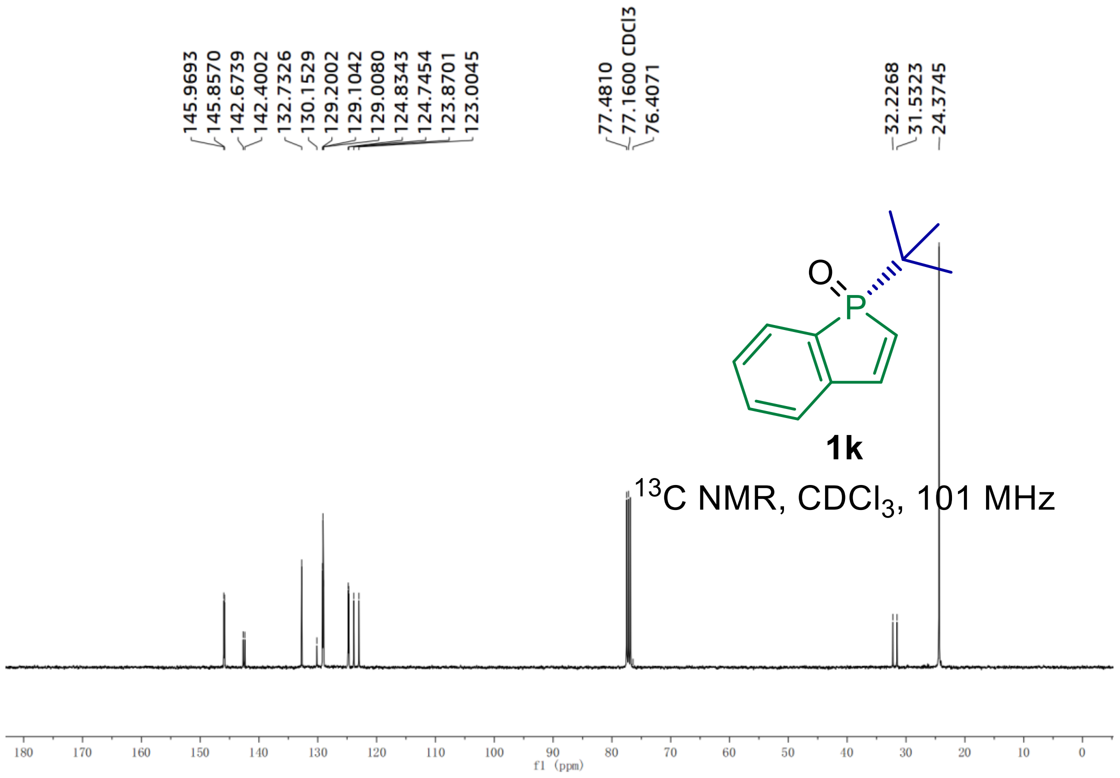

Supplementary Figure 178. ^{13}**C NMR of the 1k (101 MHz,** $CDCl_3$**)**

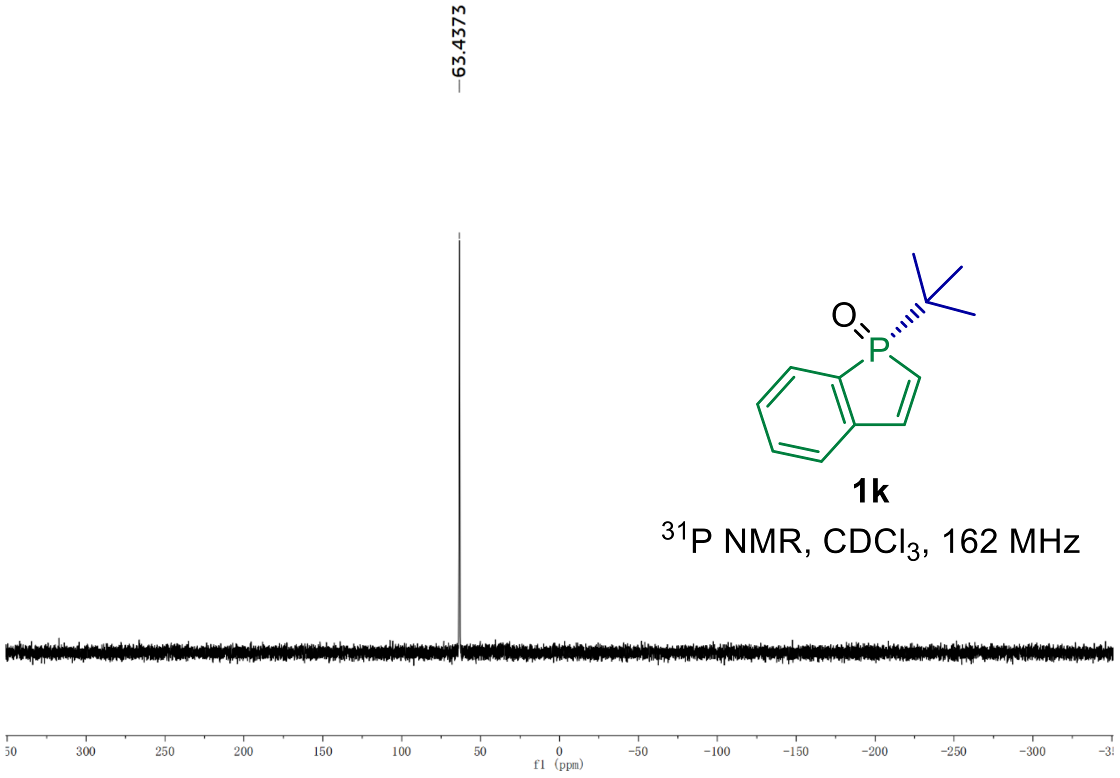

Supplementary Figure 179. ^{31}**P NMR of the 1k (162 MHz,** $CDCl_3$**)**

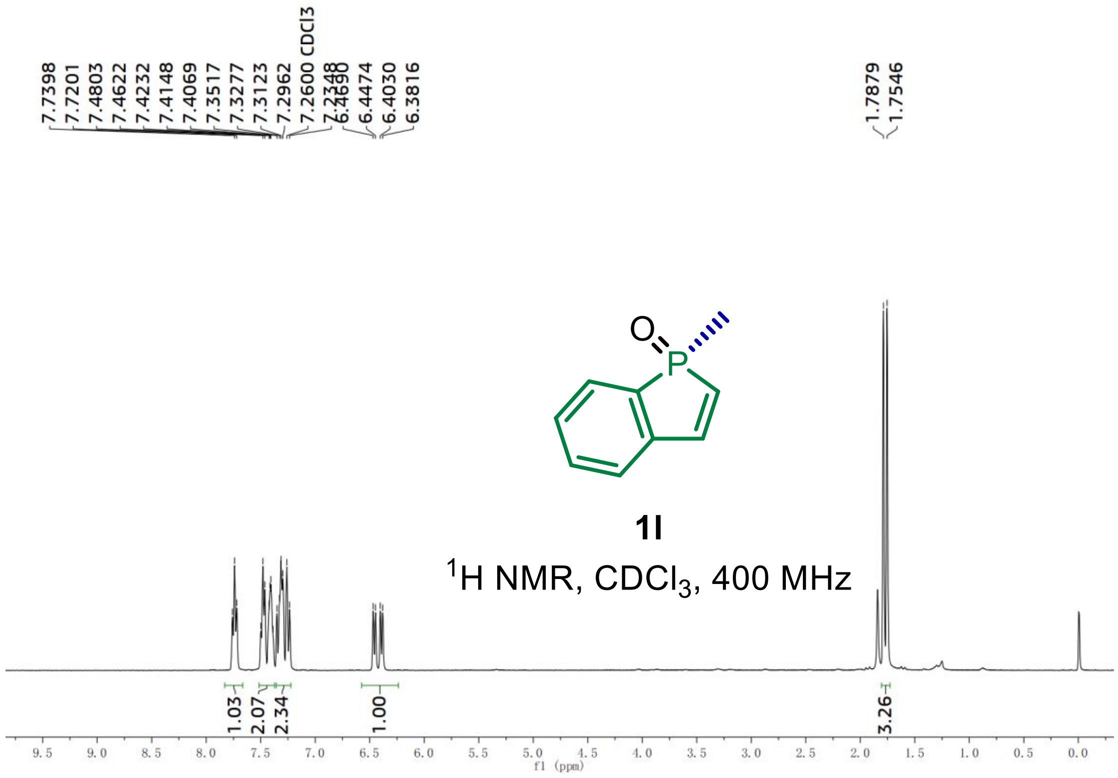

Supplementary Figure 180. ^{1}**H NMR of the 1l (400 MHz,** $CDCl_3$**)**

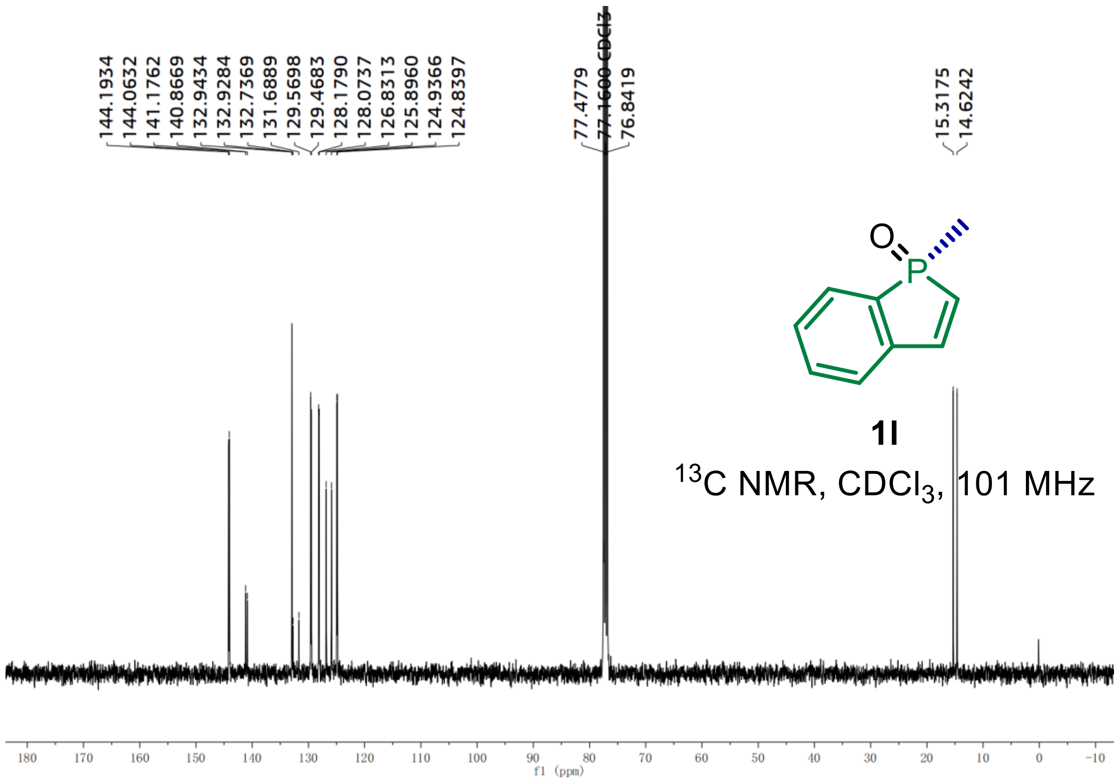

Supplementary Figure 181. ^{13}**C NMR of the 1l (101 MHz,** $CDCl_3$**)**

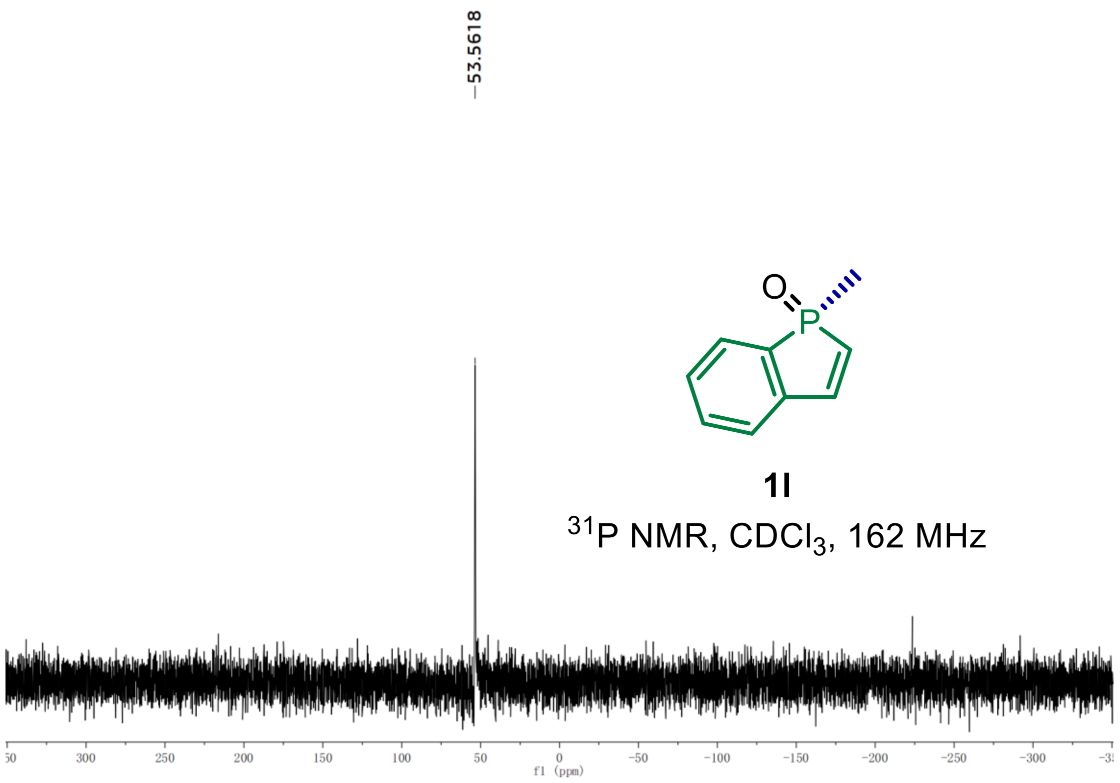

Supplementary Figure 182. ^{31}**P NMR of the 1l (162 MHz, CDCl₃)**

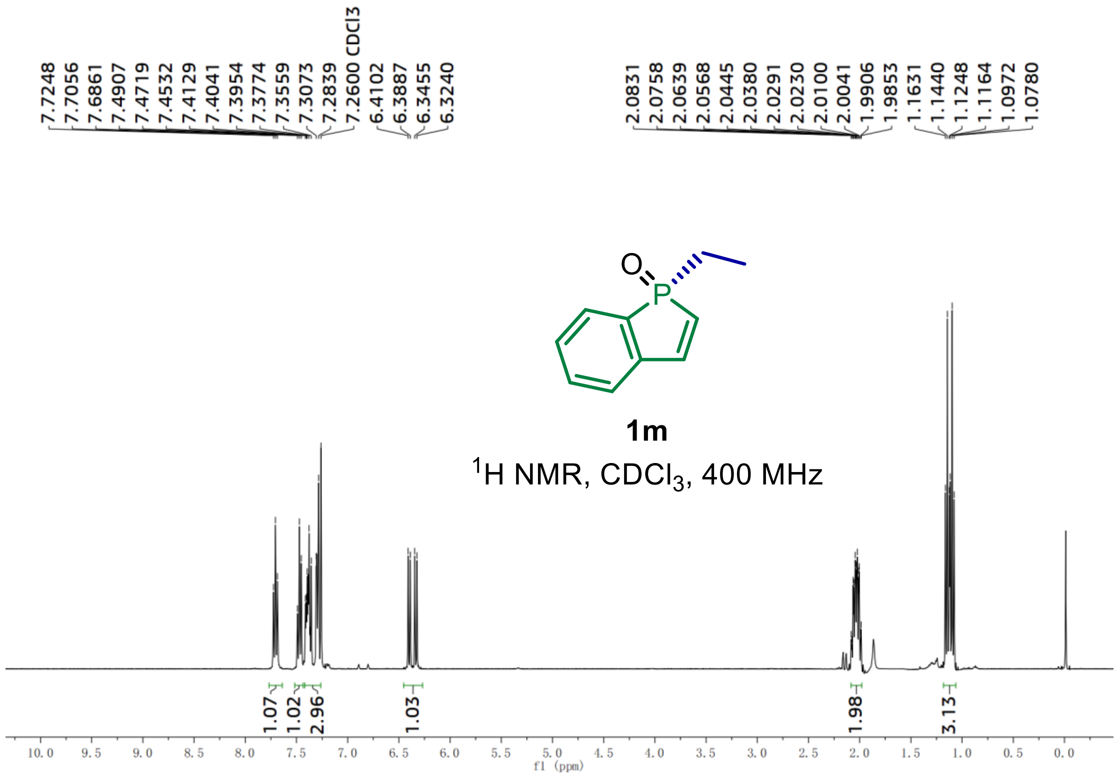

Supplementary Figure 183. ^{1}**H NMR of the 1m (400 MHz, CDCl₃)**

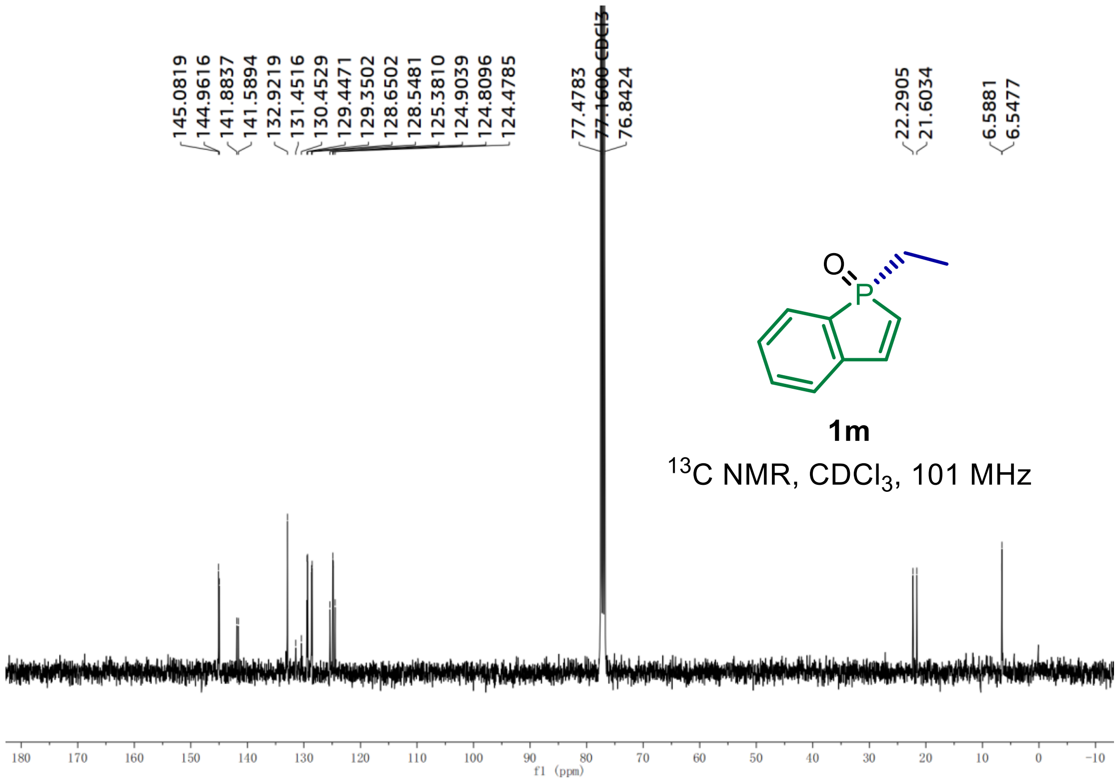

Supplementary Figure 184. ^{13}**C NMR of the 1m (101 MHz, CDCl**$_3$**)**

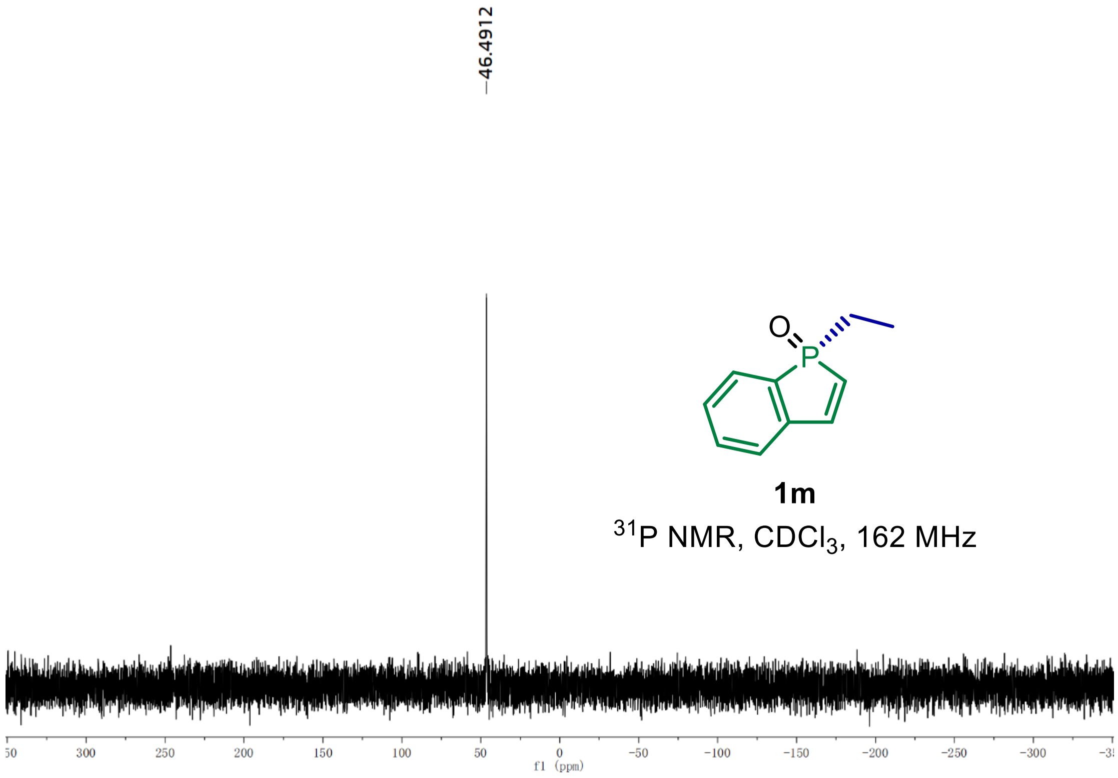

Supplementary Figure 185. ^{31}**P NMR of the 1m (162 MHz, CDCl**$_3$**)**

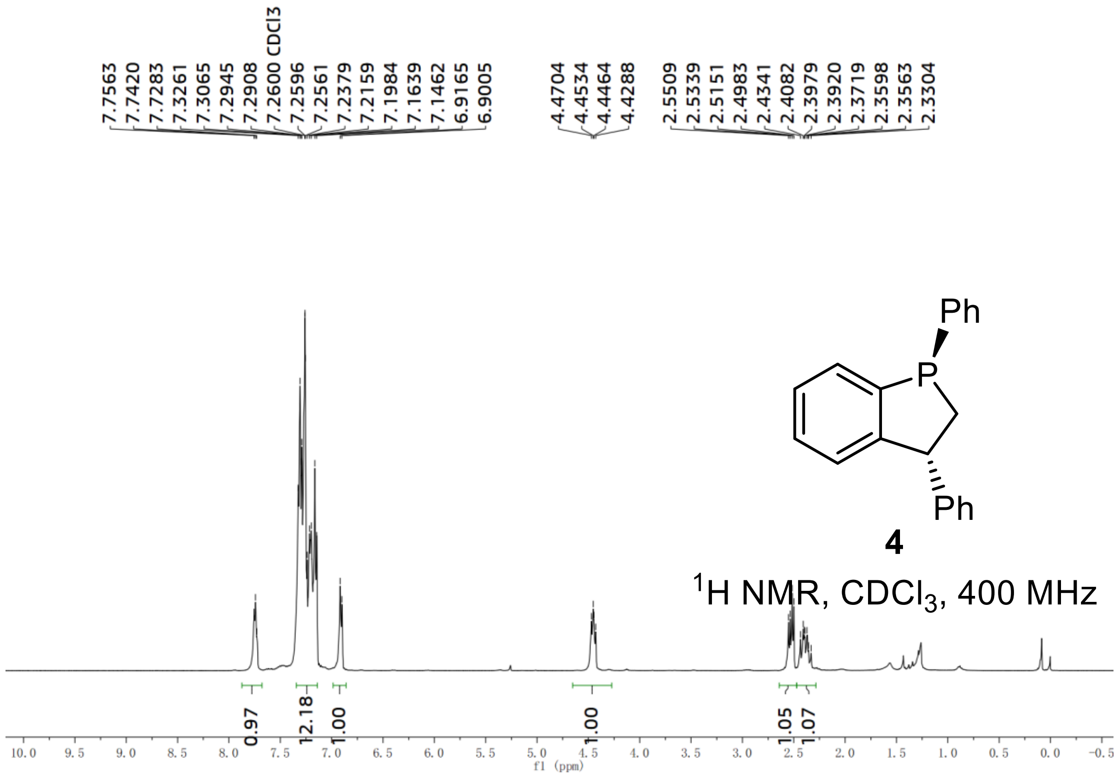

Supplementary Figure 186. ^{1}**H NMR of the 4 (400 MHz,** $CDCl_3$**)**

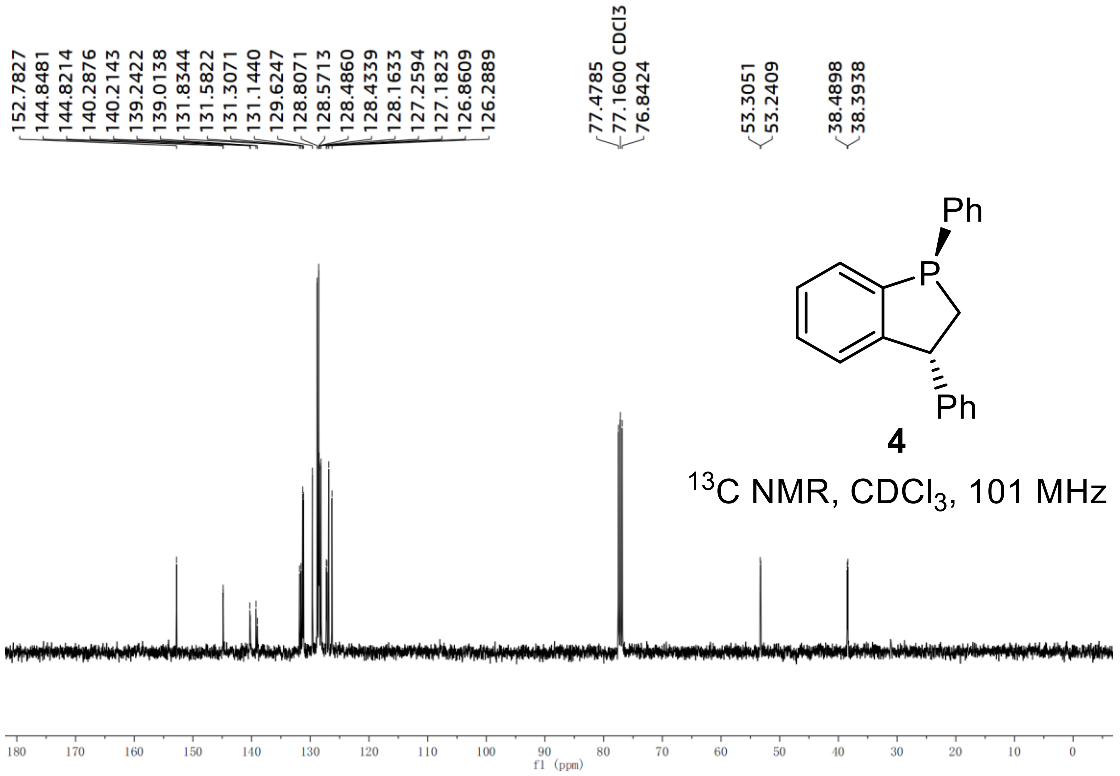

Supplementary Figure 187. ^{13}**C NMR of the 4 (101 MHz,** $CDCl_3$**)**

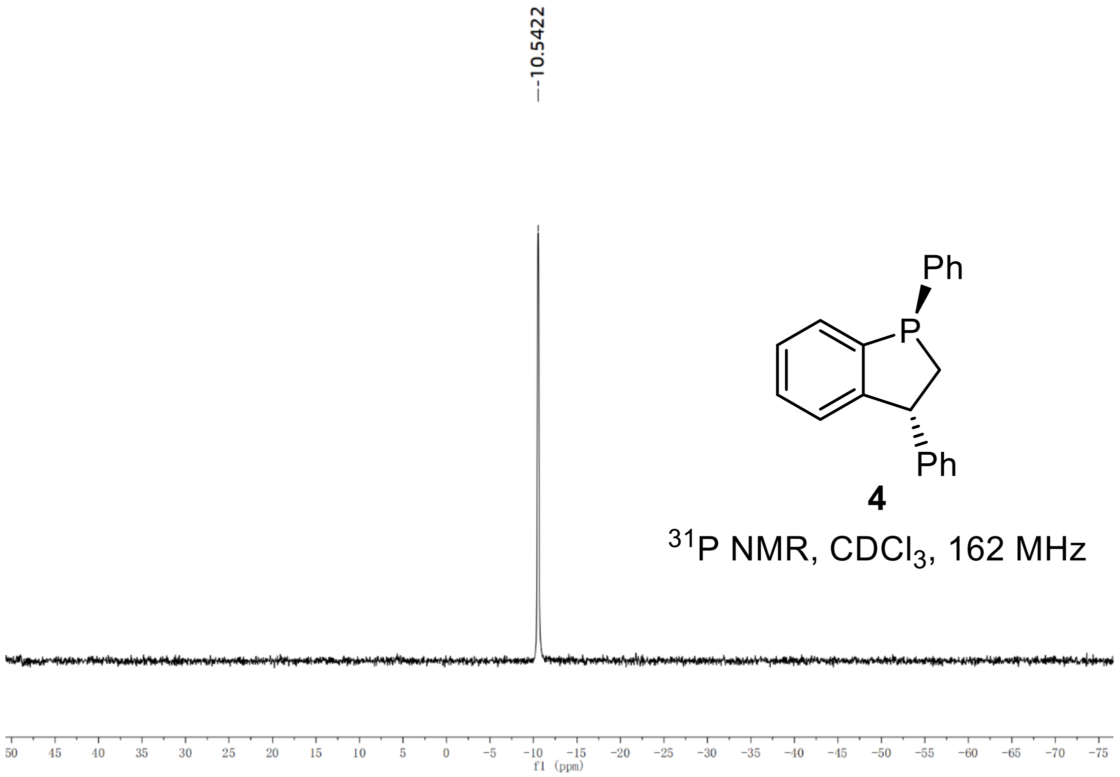

Supplementary Figure 188. ^{31}P NMR of the 4 (162 MHz, $CDCl_3$)

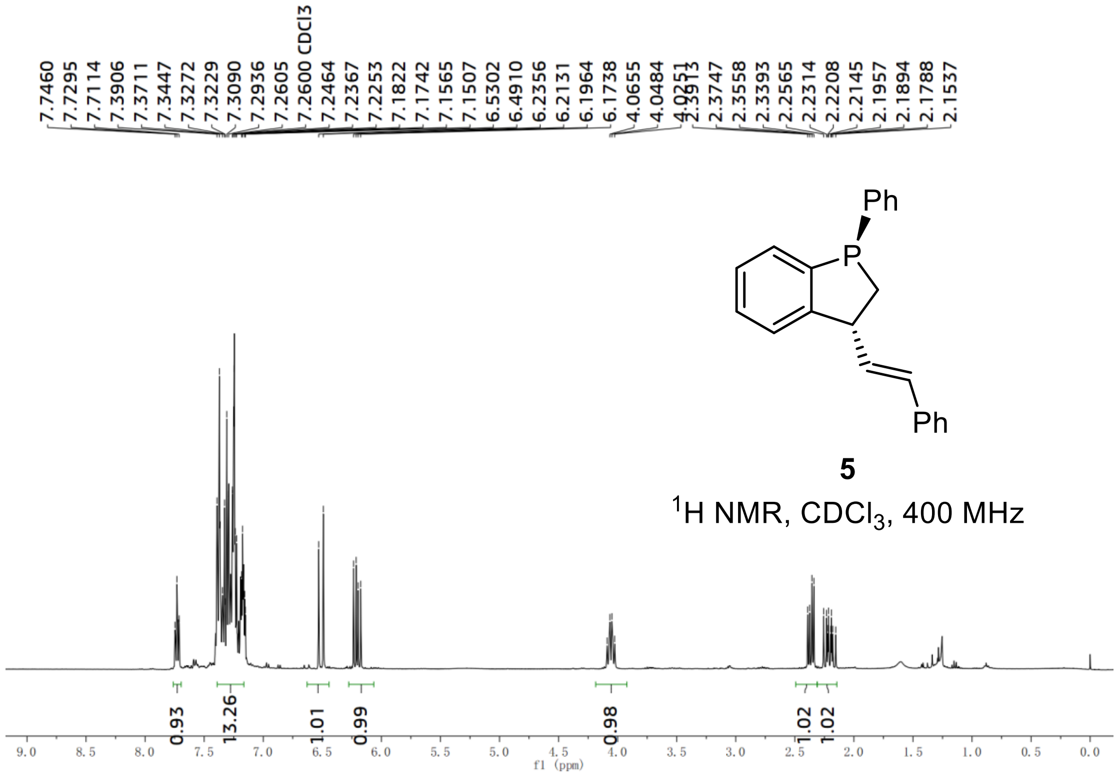

Supplementary Figure 189. ^{1}H NMR of the 5 (400 MHz, $CDCl_3$)

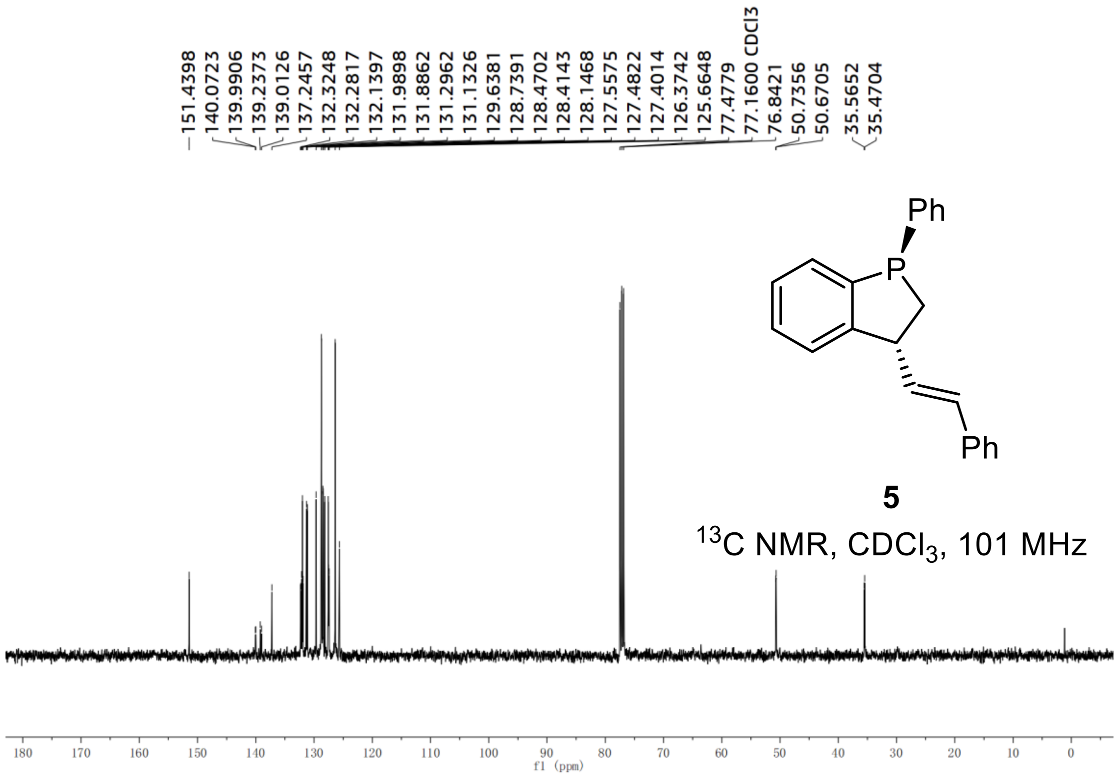

Supplementary Figure 190. ^{13}**C NMR of the 5 (101 MHz,** $CDCl_3$**)**

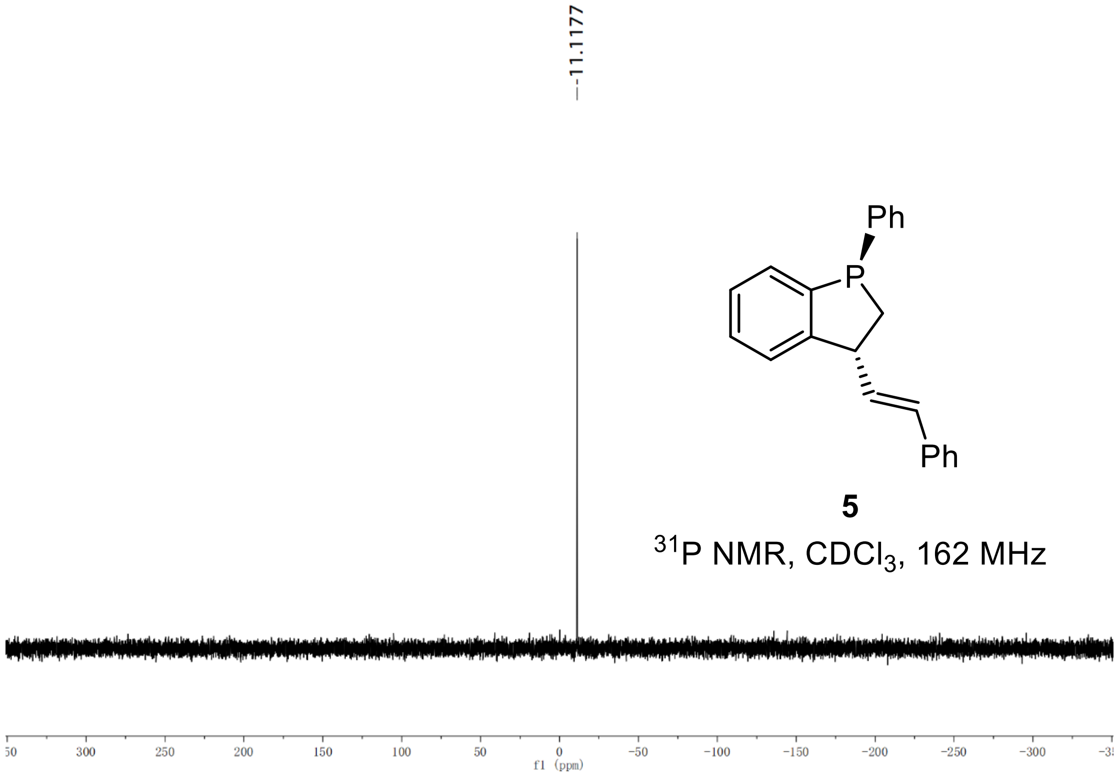

Supplementary Figure 191. ^{31}**P NMR of the 5 (162 MHz,** $CDCl_3$**)**

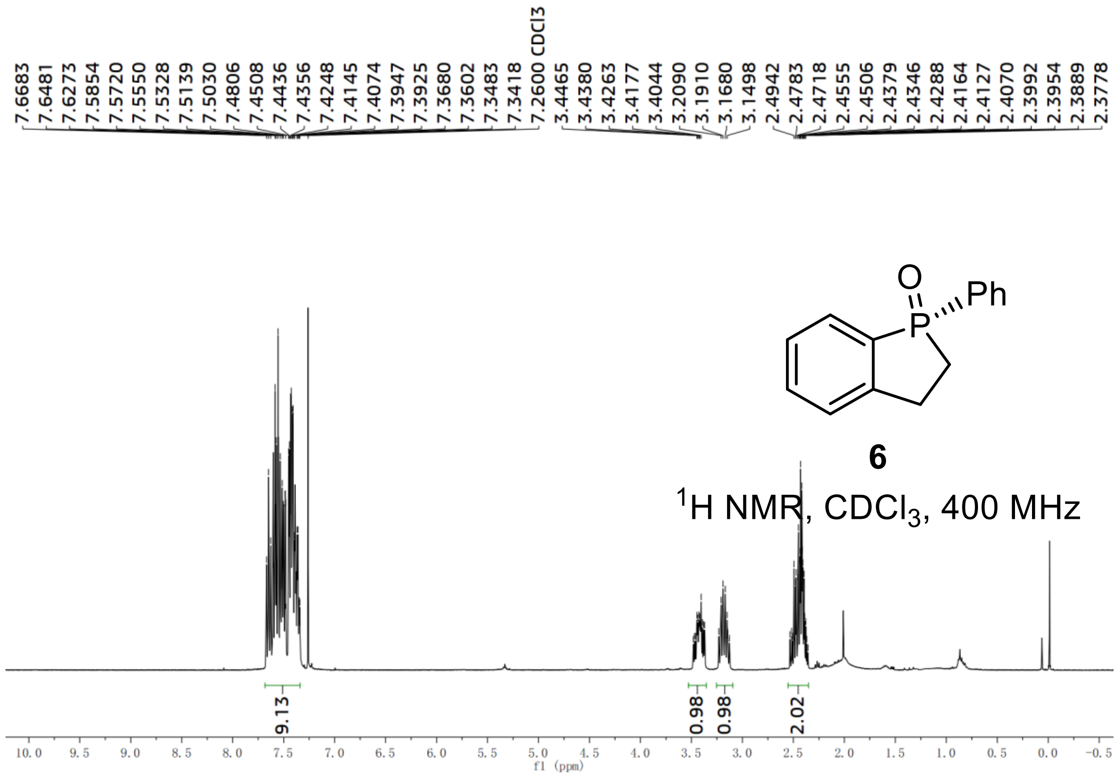

Supplementary Figure 192. ^{1}H NMR of the 6 (400 MHz, $CDCl_3$)

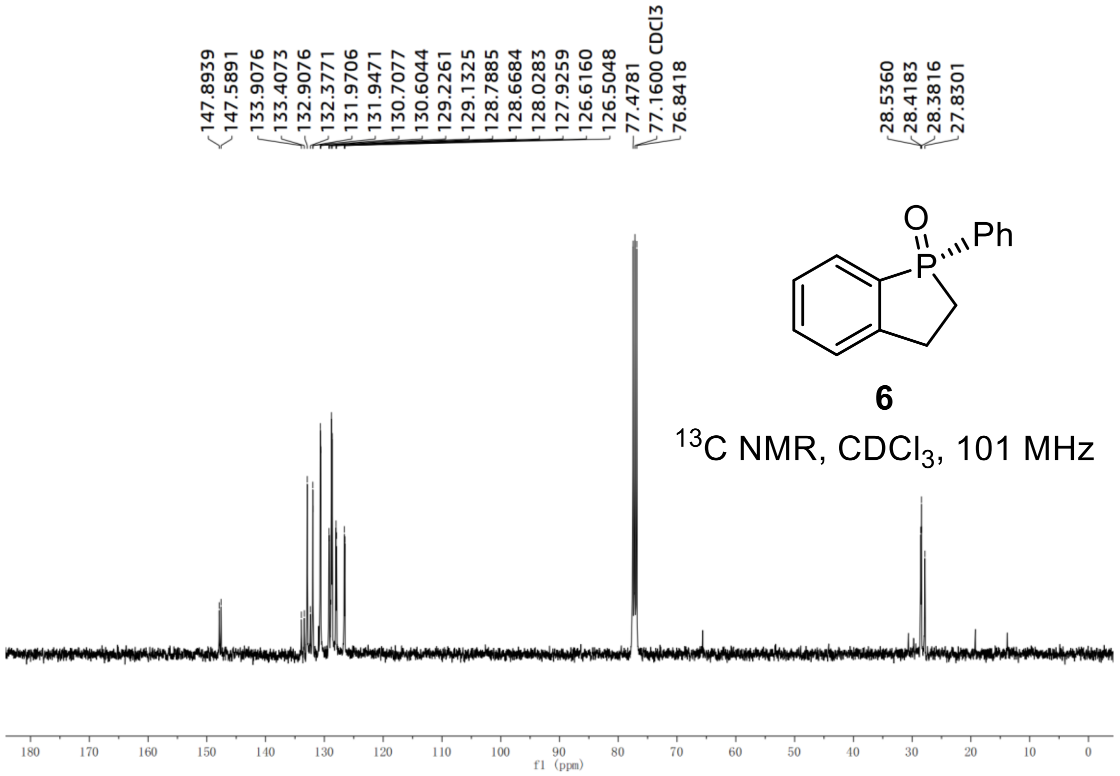

Supplementary Figure 193. ^{13}C NMR of the 6 (101 MHz, $CDCl_3$)

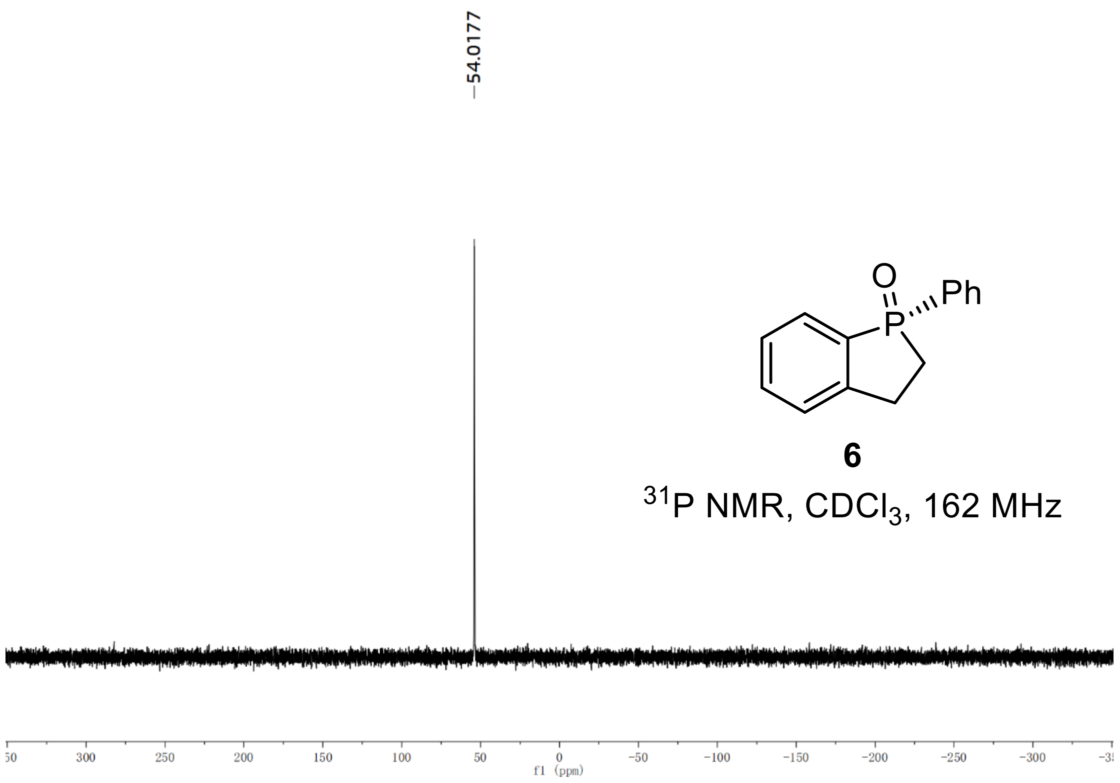

Supplementary Figure 194. ^{31}P NMR of the 6 (162 MHz, CDCl$_3$)

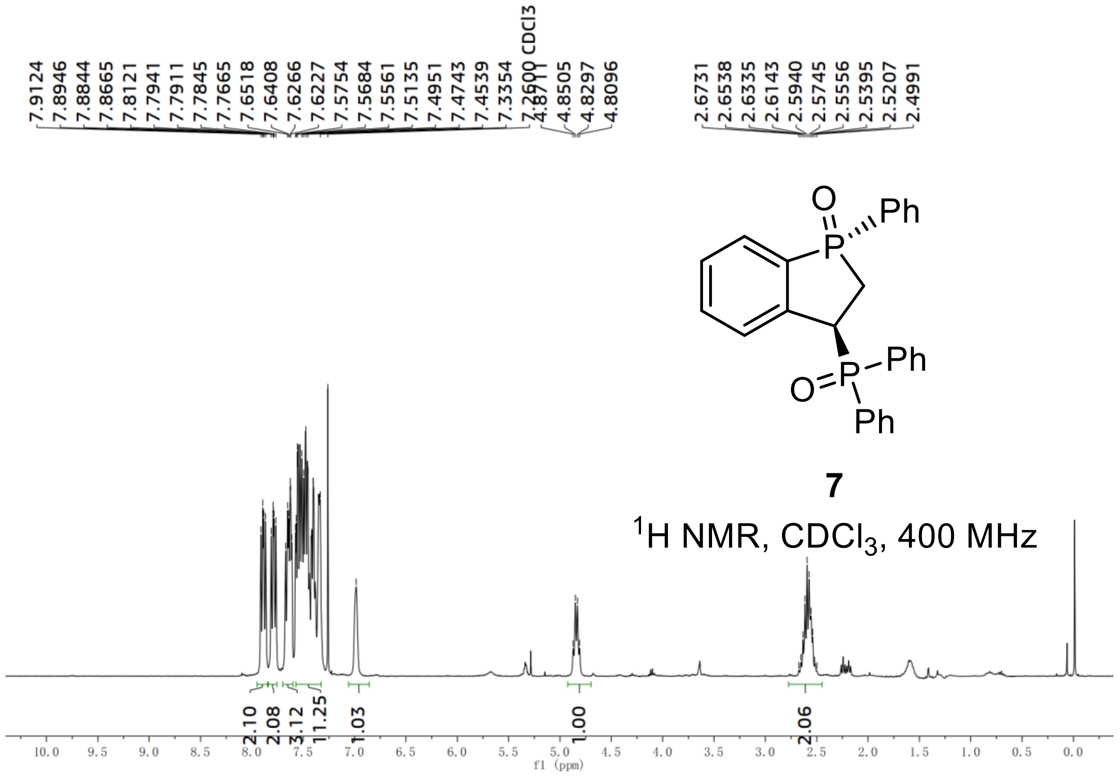

Supplementary Figure 195. ^{1}H NMR of the 7 (400 MHz, CDCl$_3$)

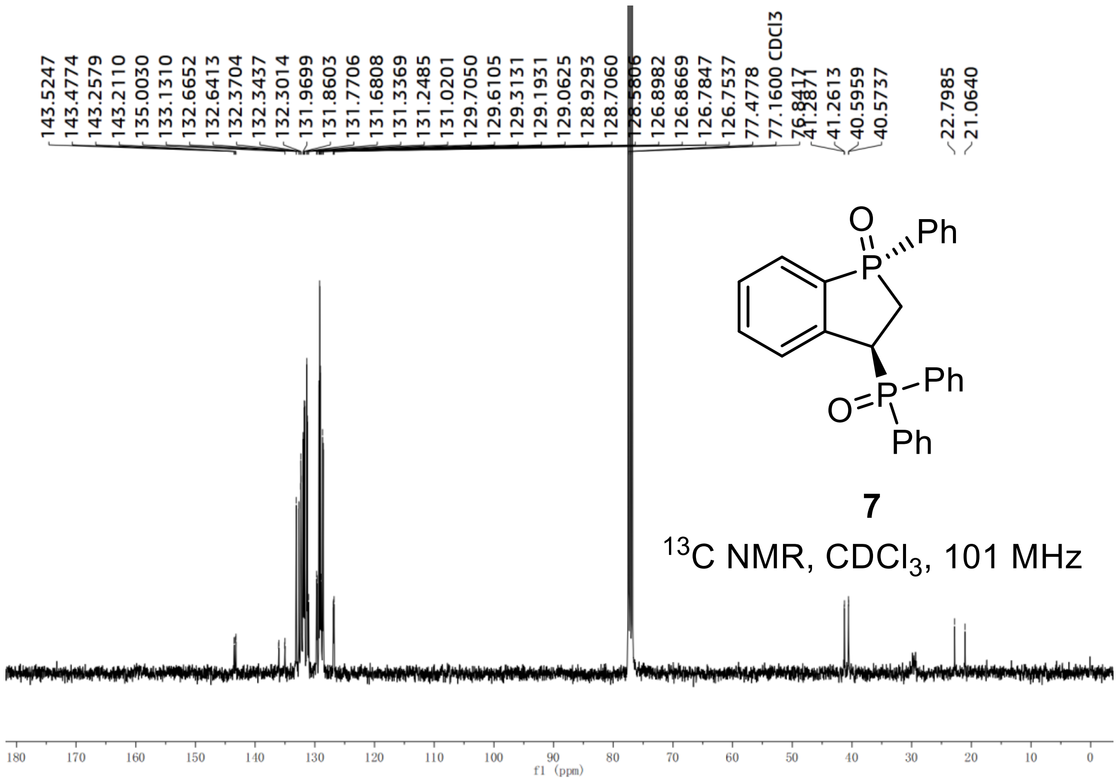

Supplementary Figure 196. ^{13}C NMR of the 7 (101 MHz, $CDCl_3$)

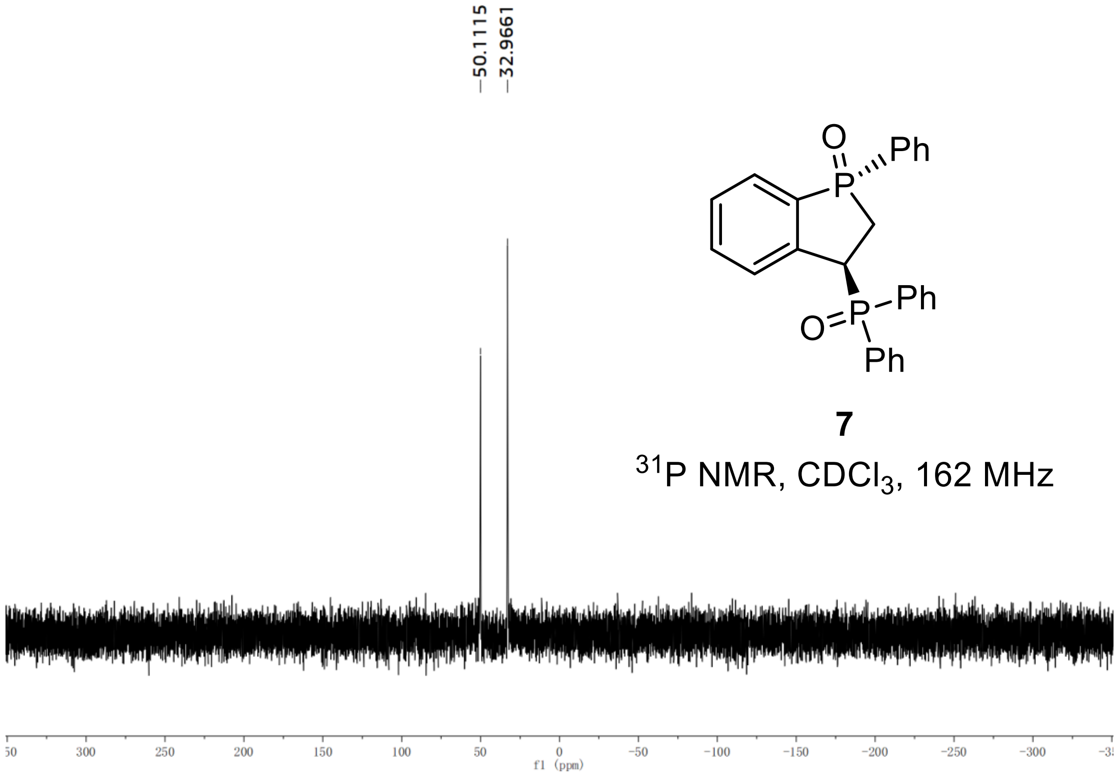

Supplementary Figure 197. ^{31}P NMR of the 7 (162 MHz, $CDCl_3$)

3.2 HPLC charts

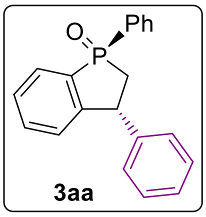

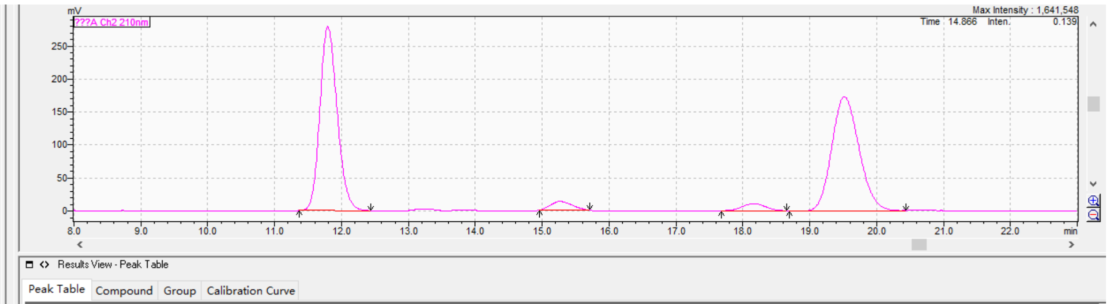

Peak#	Ret. Time	Area	Height	Mark	Conc.	Unit	ID#	Name	Area%
1	11.795	5004083	278794	M	47.534				47.534
2	15.273	302472	13419	M	2.873				2.873
3	18.162	264276	10754	M	2.510				2.510
4	19.519	4956557	173683	M	47.082				47.082
Total		10527386	476650		100.000				100.00

Supplementary Figure 198. HPLC spectrum of racemic 3aa

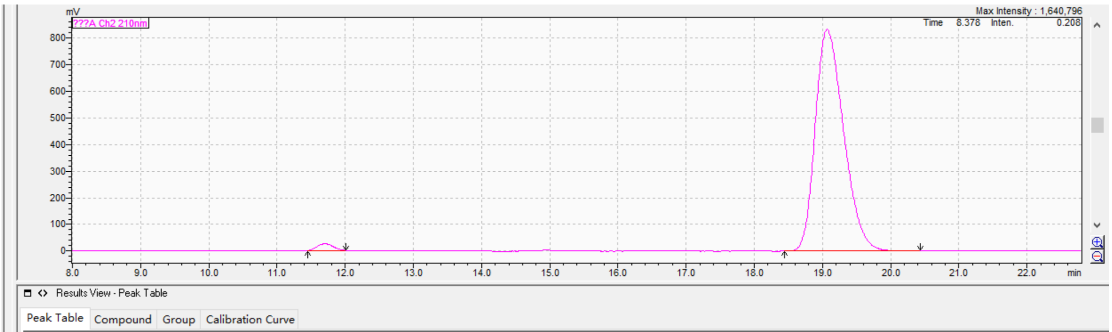

Peak#	Ret. Time	Area	Height	Mark	Conc.	Unit	ID#	Name	Area%
1	11.701	424709	25478	M	1.730				1.730
2	19.069	24127293	831320	M	98.270				98.270
Total		24552002	856798		100.000				100.00

Supplementary Figure 199. HPLC spectrum of 3aa

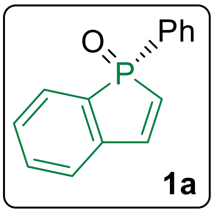

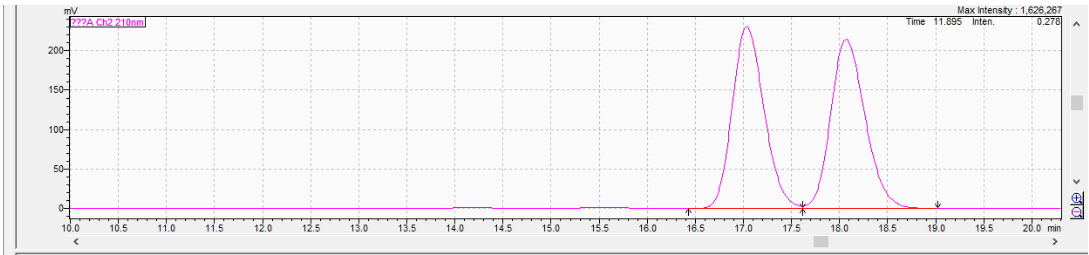

Results View - Peak Table

Peak Table Compound Group Calibration Curve

Peak#	Ret. Time	Area	Height	Mark	Conc.	Unit	ID#	Name	Area%
1	17.034	5302082	230514	M	49.921				49.921
2	18.069	5318813	214126	V M	50.079				50.079
Total		10620895	444641		100.000				100.00

Supplementary Figure 200. HPLC spectrum of racemic 1a

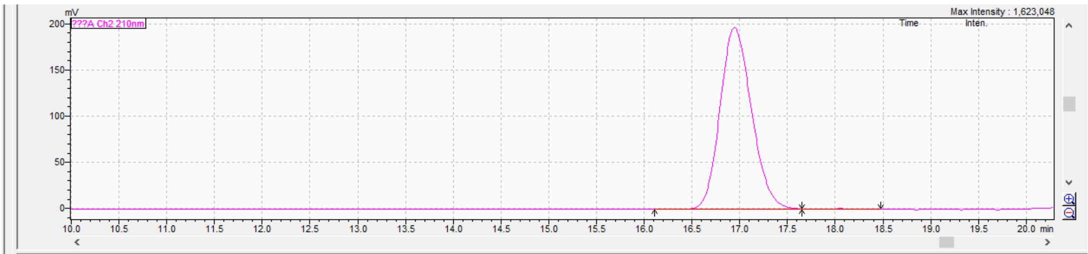

Results View - Peak Table

Peak Table Compound Group Calibration Curve

Peak#	Ret. Time	Area	Height	Mark	Conc.	Unit	ID#	Name	Area%
1	16.946	4532239	196513	M	99.587				99.587
2	18.049	18818	761	V M	0.413				0.413
Total		4551056	197274		100.000				100.00

Supplementary Figure 201. HPLC spectrum of 1a

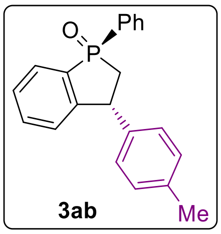

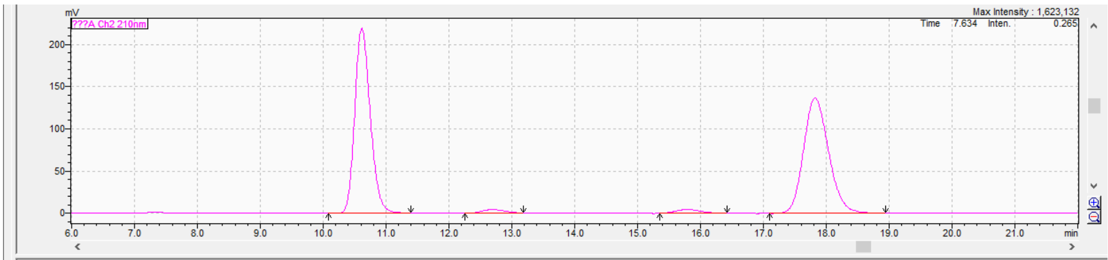

Results View - Peak Table

Peak Table | Compound | Group | Calibration Curve

Peak#	Ret. Time	Area	Height	Mark	Conc.	Unit	ID#	Name	Area%
1	10.613	3759864	218840	M	48.551				48.551
2	12.676	100576	4083	M	1.299				1.299
3	15.792	121045	4558	M	1.563				1.563
4	17.823	3762721	136273	M	48.588				48.588
Total		7744207	363752		100.000				100.00

Supplementary Figure 202. HPLC spectrum of racemic 3ab

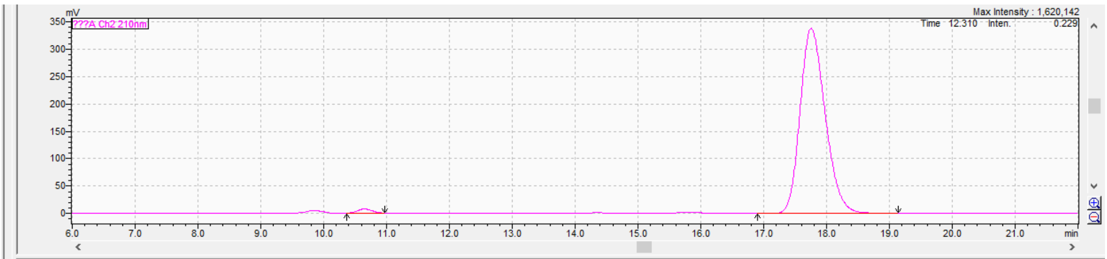

Results View - Peak Table

Peak Table | Compound | Group | Calibration Curve

Peak#	Ret. Time	Area	Height	Mark	Conc.	Unit	ID#	Name	Area%
1	10.646	130867	7906	M	1.377				1.377
2	17.756	9374790	337485	M	98.623				98.623
Total		9505656	345391		100.000				100.00

Supplementary Figure 203. HPLC spectrum of 3ab

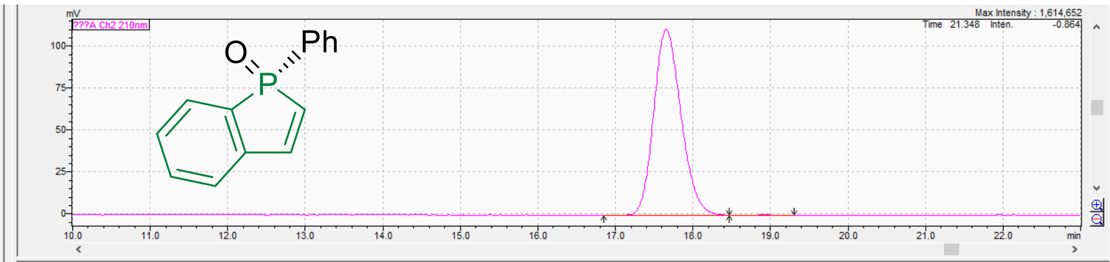

Results View - Peak Table

Peak Table | Compound | Group | Calibration Curve

Peak#	Ret. Time	Area	Height	Mark	Conc.	Unit	ID#	Name	Area%
1	17.655	2721217	111008	M	99.692				99.692
2	18.943	8414	341	V M	0.308				0.308
Total		2729631	111349		100.000				100.00

Supplementary Figure 204. HPLC spectrum of 1a

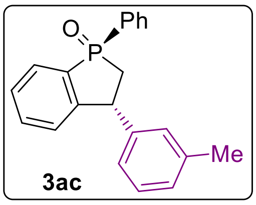

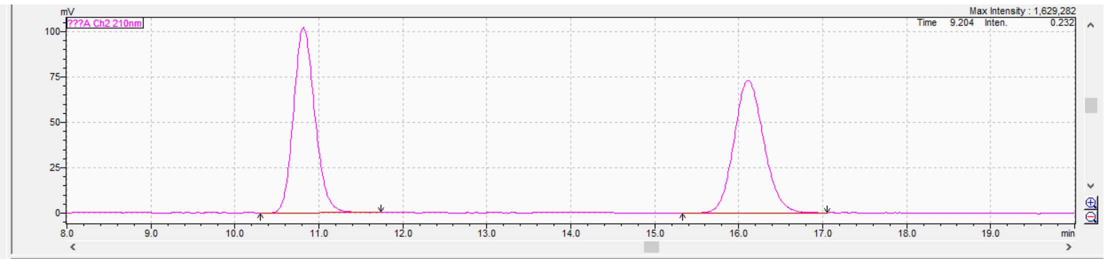

Peak#	Ret. Time	Area	Height	Mark	Conc.	Unit	ID#	Name	Area%
1	10.816	1800135	102001	M	49.765				49.765
2	16.110	1817131	73210	M	50.235				50.235
Total		3617266	175211		100.000				100.00

Supplementary Figure 205. HPLC spectrum of racemic 3ac

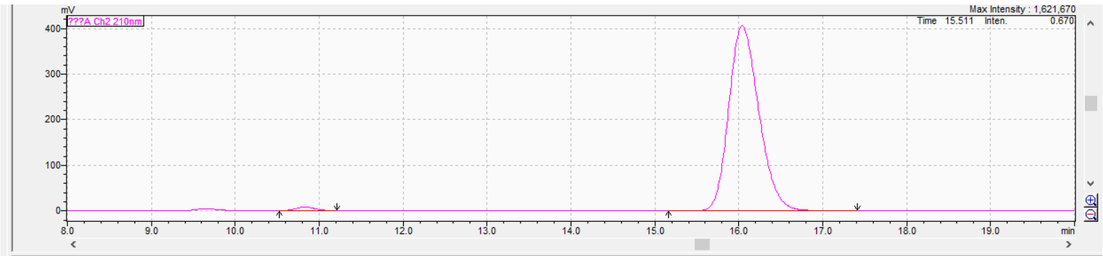

Peak#	Ret. Time	Area	Height	Mark	Conc.	Unit	ID#	Name	Area%
1	10.827	142944	8304	M	1.393				1.393
2	16.040	10119343	405897	M	98.607				98.607
Total		10262287	414201		100.000				100.00

Supplementary Figure 206. HPLC spectrum of 3ac

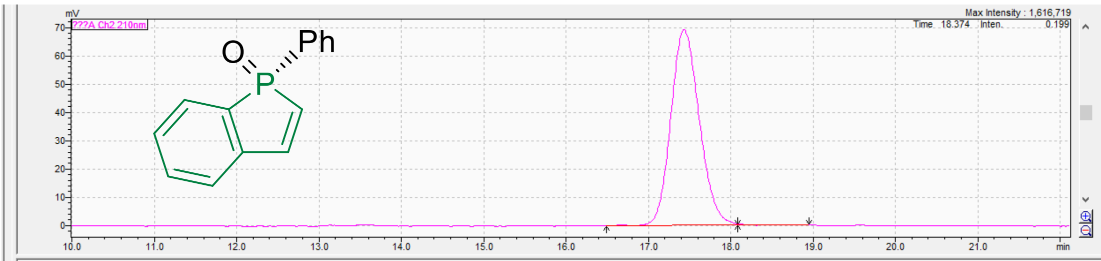

Peak#	Ret. Time	Area	Height	Mark	Conc.	Unit	ID#	Name	Area%
1	17.433	1667789	69184	M	99.752				99.752
2	18.110	4150	481	V M	0.248				0.248
Total		1671939	69664		100.000				100.00

Supplementary Figure 207. HPLC spectrum of 1a

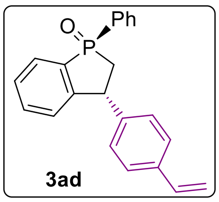

3ad

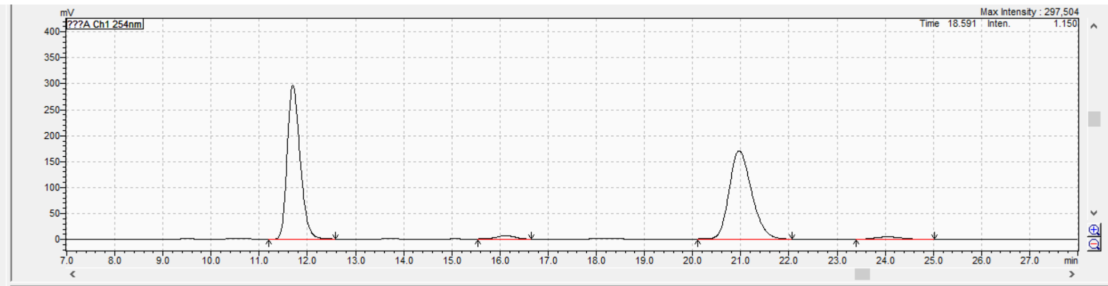

Results View - Peak Table

Peak Table | Compound | Group | Calibration Curve

Peak#	Ret. Time	Area	Height	Mark	Conc.	Unit	ID#	Name	Area%
1	11. 697	5524408	296802	M	48. 457				48. 457
2	16. 122	180053	6615	M	1. 579				1. 579
3	20. 967	5509621	170193	M	48. 327				48. 327
4	24. 049	186528	4815	M	1. 636				1. 636
Total		11400610	478425		100. 000				100. 00

Supplementary Figure 208. HPLC spectrum of racemic 3ad

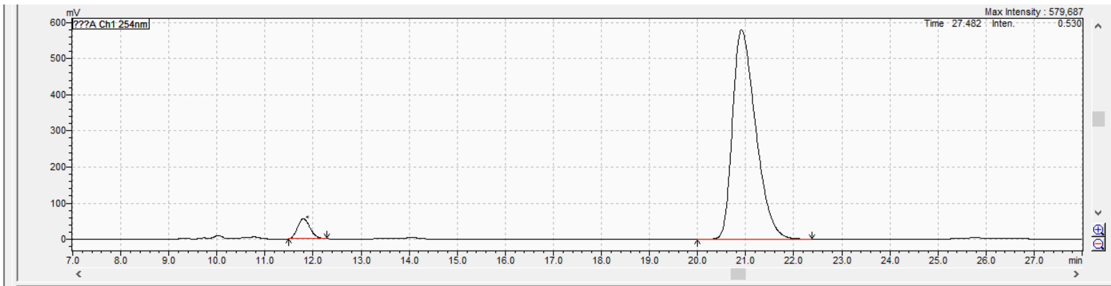

Results View - Peak Table

Peak Table | Compound | Group | Calibration Curve

Peak#	Ret. Time	Area	Height	Mark	Conc.	Unit	ID#	Name	Area%
1	11. 798	1001862	55371	M	4. 911				4. 911
2	20. 916	19397624	579218	M	95. 089				95. 089
Total		20399486	634589		100. 000				100. 00

Supplementary Figure 209. HPLC spectrum of 3ad

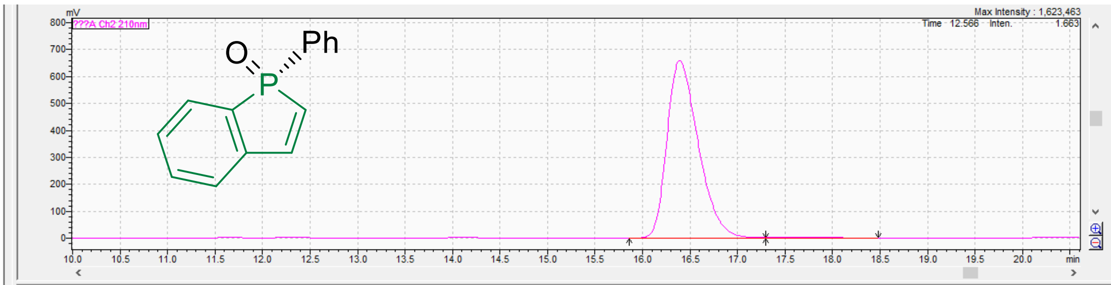

Results View - Peak Table

Peak Table | Compound | Group | Calibration Curve

Peak#	Ret. Time	Area	Height	Mark	Conc.	Unit	ID#	Name	Area%
1	16. 392	15074832	658101	M	99. 352				99. 352
2	17. 587	98358	3209	V M	0. 648				0. 648
Total		15173191	661310		100. 000				100. 00

Supplementary Figure 210. HPLC spectrum of 1a

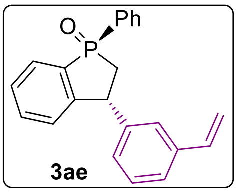

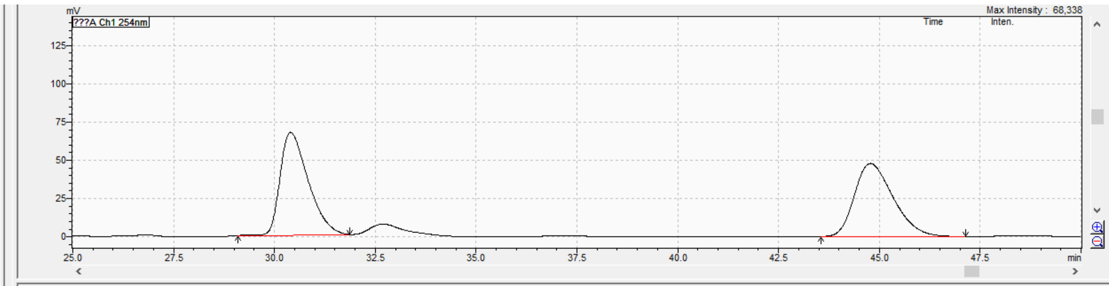

Results View - Peak Table

Peak Table | Compound | Group | Calibration Curve

Peak#	Ret. Time	Area	Height	Mark	Conc.	Unit	ID#	Name	Area%
1	30. 400	3251868	67191	M	50. 540				50. 540
2	44. 777	3182340	47798	M	49. 460				49. 460
Total		6434208	114989		100. 000				100. 00

Supplementary Figure 211. HPLC spectrum of racemic 3ae

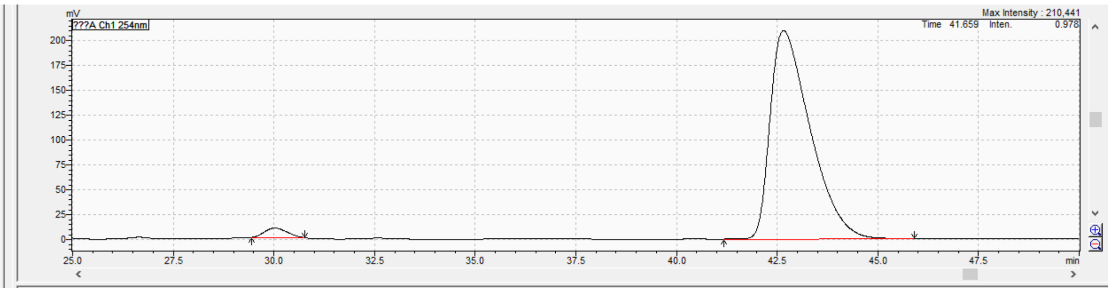

Results View - Peak Table

Peak Table | Compound | Group | Calibration Curve

Peak#	Ret. Time	Area	Height	Mark	Conc.	Unit	ID#	Name	Area%
1	30. 016	376287	9554	M	2. 539				2. 539
2	42. 658	14446577	209608	M	97. 461				97. 461
Total		14822864	219163		100. 000				100. 00

Supplementary Figure 212. HPLC spectrum of 3ae

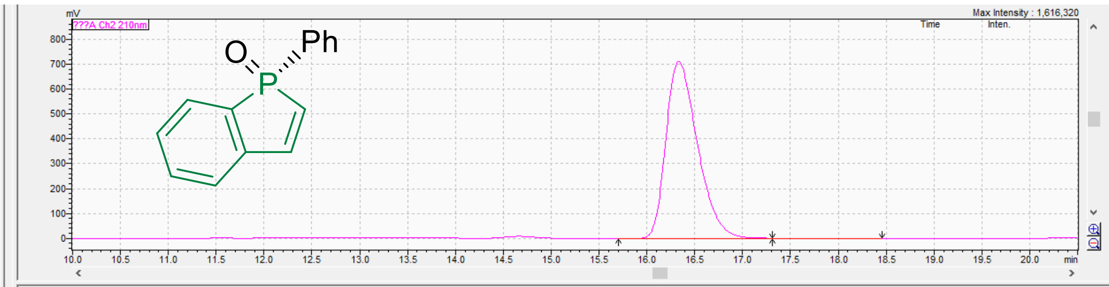

Results View - Peak Table

Peak Table | Compound | Group | Calibration Curve

Peak#	Ret. Time	Area	Height	Mark	Conc.	Unit	ID#	Name	Area%
1	16. 332	16376572	713005	M	99. 715				99. 715
2	17. 364	46759	1366	V M	0. 285				0. 285
Total		16423331	714371		100. 000				100. 00

Supplementary Figure 213. HPLC spectrum of 1a

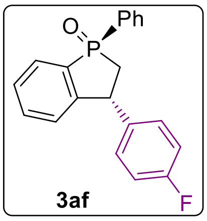

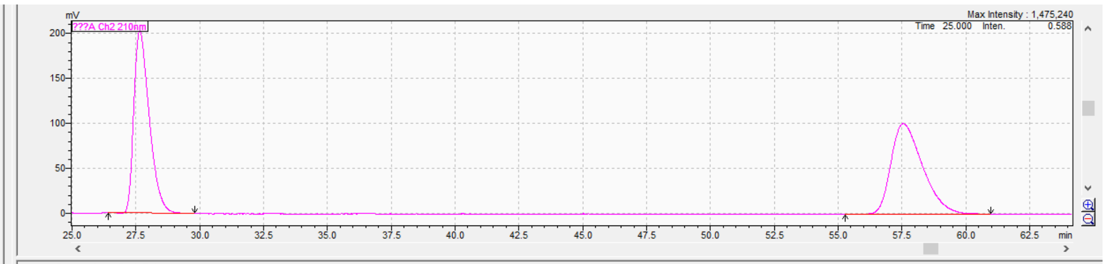

Results View - Peak Table

Peak Table | Compound | Group | Calibration Curve

Peak#	Ret. Time	Area	Height	Mark	Conc.	Unit	ID#	Name	Area%
1	27. 651	8520061	202472	M	49. 444				49. 444
2	57. 555	8711591	100885	M	50. 556				50. 556
Total		17231652	303357		100. 000				100. 00

Supplementary Figure 214. HPLC spectrum of racemic 3af

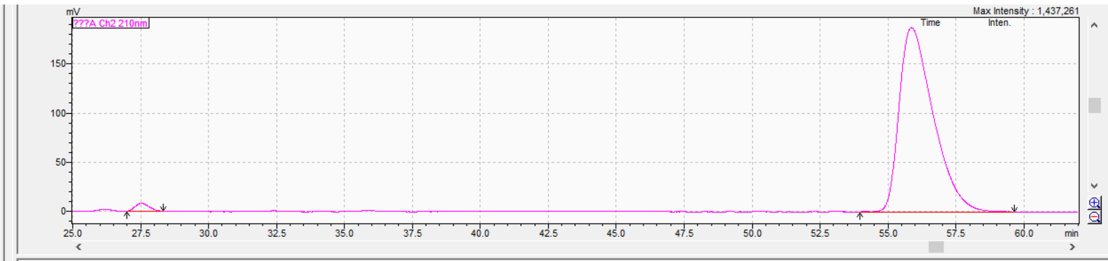

Results View - Peak Table

Peak Table | Compound | Group | Calibration Curve

Peak#	Ret. Time	Area	Height	Mark	Conc.	Unit	ID#	Name	Area%
1	27. 523	290166	7985	M	1. 747				1. 747
2	55. 861	16321975	187691	M	98. 253				98. 253
Total		16612141	195676		100. 000				100. 00

Supplementary Figure 215. HPLC spectrum of 3af

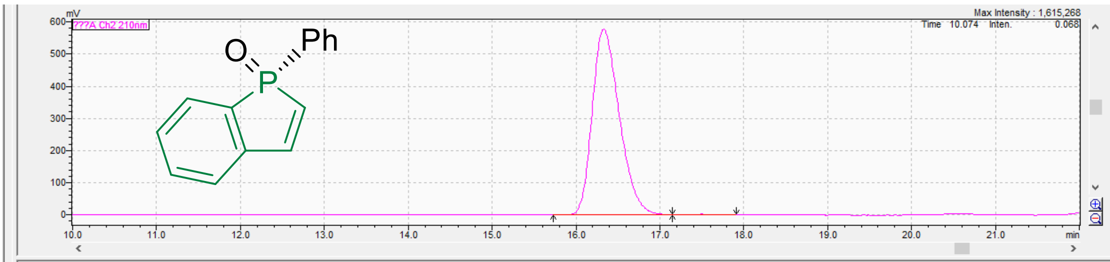

Results View - Peak Table

Peak Table | Compound | Group | Calibration Curve

Peak#	Ret. Time	Area	Height	Mark	Conc.	Unit	ID#	Name	Area%
1	16. 330	13021263	577803	M	99. 582				99. 582
2	17. 498	54598	2415	V M	0. 418				0. 418
Total		13075861	580218		100. 000				100. 00

Supplementary Figure 216. HPLC spectrum of 1a

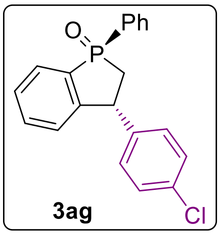

3ag

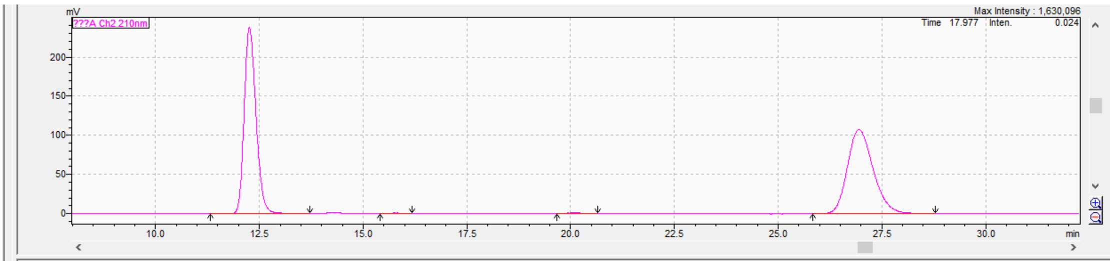

Peak#	Ret. Time	Area	Height	Mark	Conc.	Unit	ID#	Name	Area%
1	12. 260	4662423	237508	M	49. 950				49. 950
2	15. 752	16631	729	M	0. 178				0. 178
3	20. 032	18127	788	M	0. 194				0. 194
4	26. 945	4636943	107671	M	49. 677				49. 677
Total		9334123	346696		100. 000				100. 00

Supplementary Figure 217. HPLC spectrum of racemic 3ag

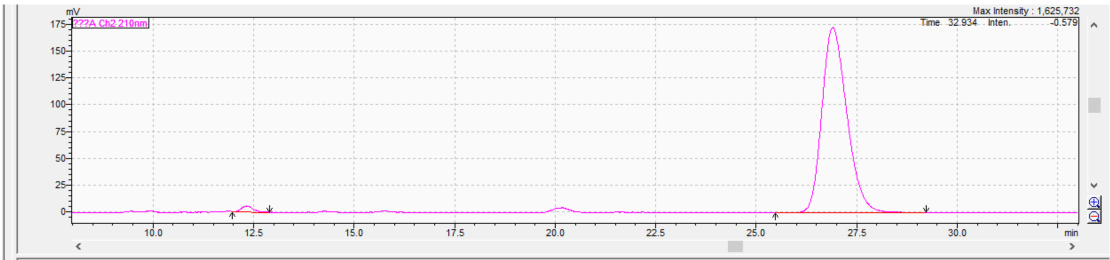

Peak#	Ret. Time	Area	Height	Mark	Conc.	Unit	ID#	Name	Area%
1	12. 329	106964	5433	M	1. 412				1. 412
2	26. 903	7466767	172504	M	98. 588				98. 588
Total		7573732	177937		100. 000				100. 00

Supplementary Figure 218. HPLC spectrum of 3ag

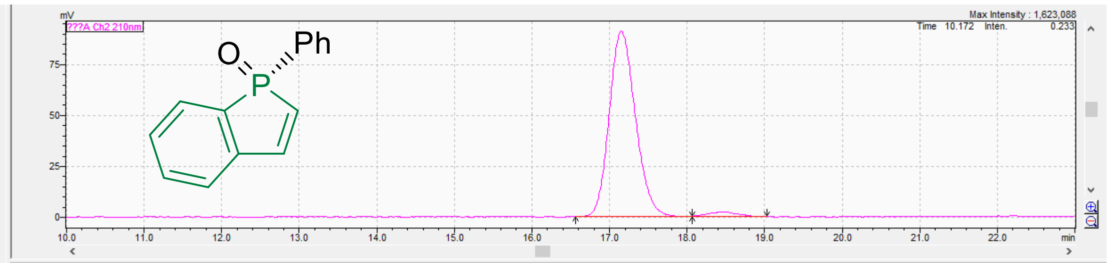

Peak#	Ret. Time	Area	Height	Mark	Conc.	Unit	ID#	Name	Area%
1	17. 152	2145575	91041	M	97. 094				97. 094
2	18. 501	64225	2223	V M	2. 906				2. 906
Total		2209800	93264		100. 000				100. 00

Supplementary Figure 219. HPLC spectrum of 1a

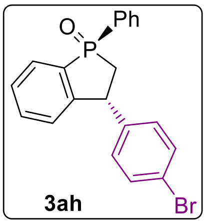

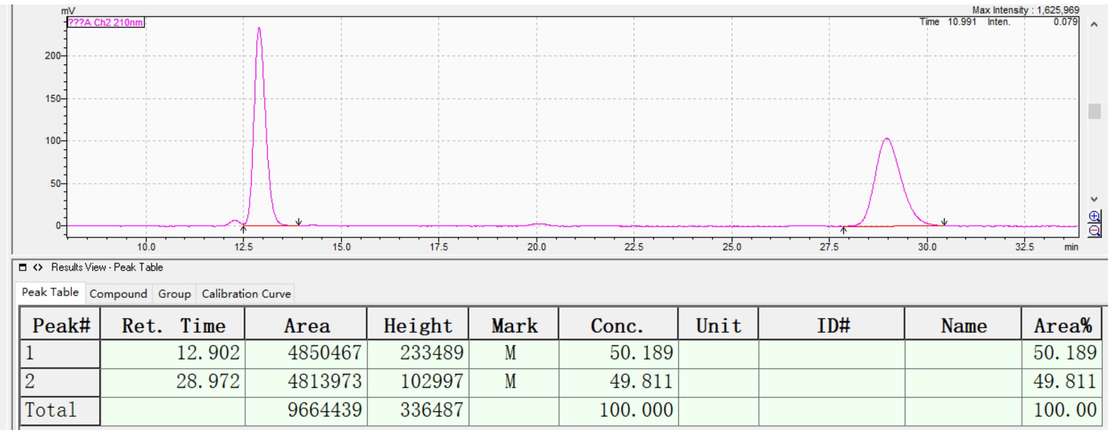

Peak#	Ret. Time	Area	Height	Mark	Conc.	Unit	ID#	Name	Area%
1	12. 902	4850467	233489	M	50. 189				50. 189
2	28. 972	4813973	102997	M	49. 811				49. 811
Total		9664439	336487		100. 000				100. 00

Supplementary Figure 220. HPLC spectrum of racemic 3ah

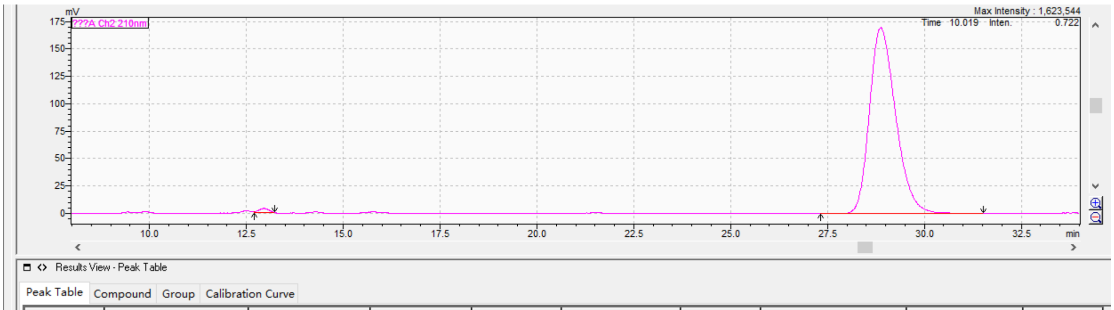

Peak#	Ret. Time	Area	Height	Mark	Conc.	Unit	ID#	Name	Area%
1	12. 962	71673	3884	M	0. 887				0. 887
2	28. 874	8005244	169730	M	99. 113				99. 113
Total		8076916	173614		100. 000				100. 00

Supplementary Figure 221. HPLC spectrum of 3ah

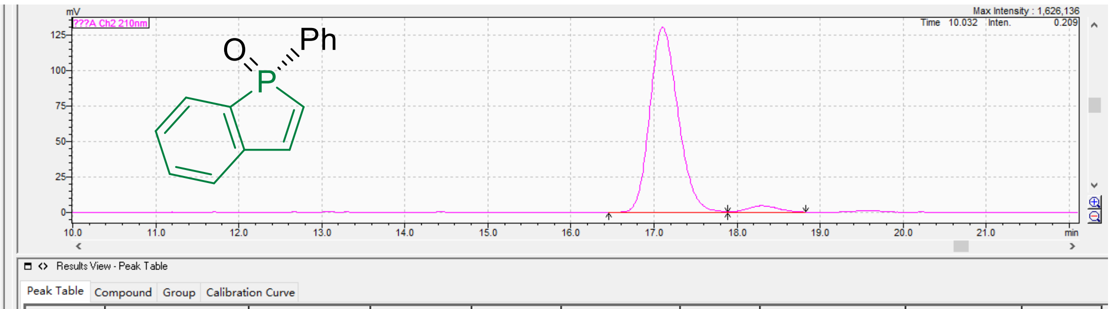

Peak#	Ret. Time	Area	Height	Mark	Conc.	Unit	ID#	Name	Area%
1	17. 104	3053987	129612	M	96. 283				96. 283
2	18. 295	117914	4595	V M	3. 717				3. 717
Total		3171901	134206		100. 000				100. 00

Supplementary Figure 222. HPLC spectrum of 1a

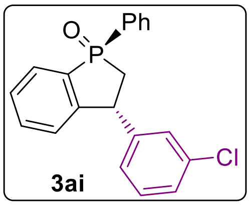

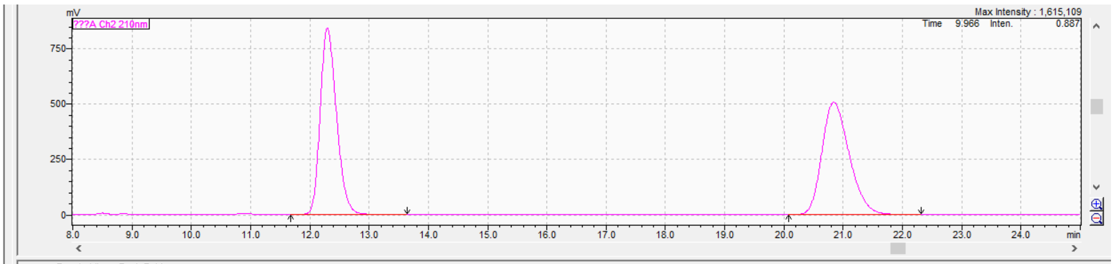

Results View - Peak Table

Peak Table | Compound | Group | Calibration Curve

Peak#	Ret. Time	Area	Height	Mark	Conc.	Unit	ID#	Name	Area%
1	12. 296	16122639	841112	M	49. 655				49. 655
2	20. 839	16346490	508070	M	50. 345				50. 345
Total		32469130	1349182		100. 000				100. 00

Supplementary Figure 223. HPLC spectrum of racemic 3ai

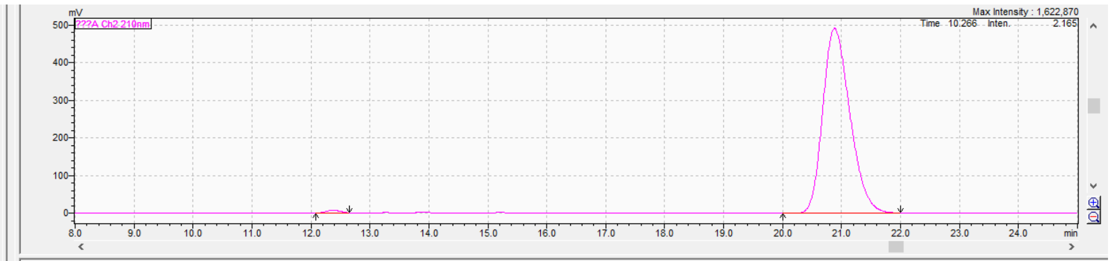

Results View - Peak Table

Peak Table | Compound | Group | Calibration Curve

Peak#	Ret. Time	Area	Height	Mark	Conc.	Unit	ID#	Name	Area%
1	12. 376	119238	7206	M	0. 755				0. 755
2	20. 880	15680863	490211	M	99. 245				99. 245
Total		15800101	497418		100. 000				100. 00

Supplementary Figure 224. HPLC spectrum of 3ai

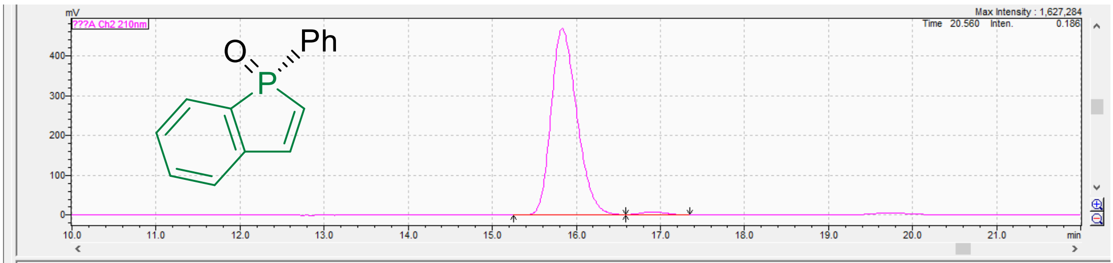

Results View - Peak Table

Peak Table | Compound | Group | Calibration Curve

Peak#	Ret. Time	Area	Height	Mark	Conc.	Unit	ID#	Name	Area%
1	15. 833	9994403	468501	M	98. 316				98. 316
2	16. 910	171206	8031	V M	1. 684				1. 684
Total		10165609	476532		100. 000				100. 00

Supplementary Figure 225. HPLC spectrum of 1a

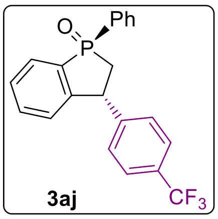

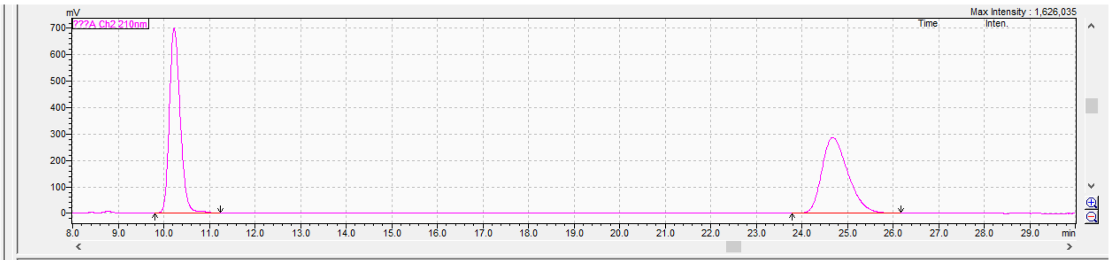

Results View - Peak Table

Peak Table | Compound | Group | Calibration Curve

Peak#	Ret. Time	Area	Height	Mark	Conc.	Unit	ID#	Name	Area%
1	10. 229	11572435	699361	M	50. 434				50. 434
2	24. 681	11373270	287000	M	49. 566				49. 566
Total		22945705	986361		100. 000				100. 00

Supplementary Figure 226. HPLC spectrum of racemic 3aj

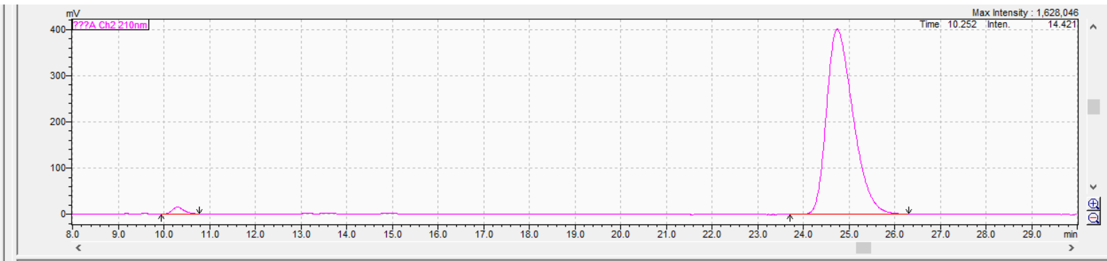

Results View - Peak Table

Peak Table | Compound | Group | Calibration Curve

Peak#	Ret. Time	Area	Height	Mark	Conc.	Unit	ID#	Name	Area%
1	10. 298	284615	15339	M	1. 733				1. 733
2	24. 745	16134314	400723	M	98. 267				98. 267
Total		16418929	416061		100. 000				100. 00

Supplementary Figure 227. HPLC spectrum of 3aj

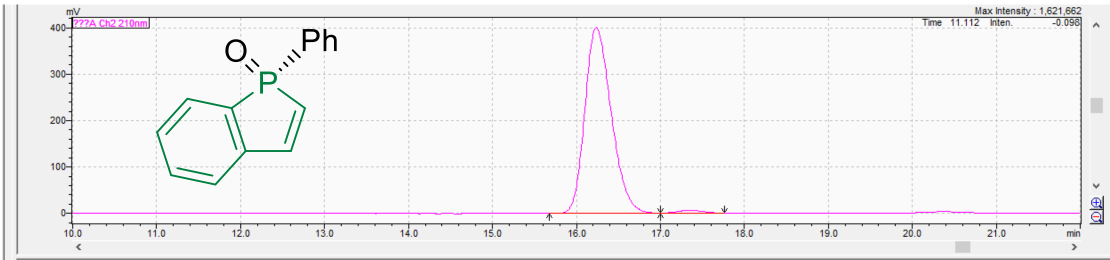

Results View - Peak Table

Peak Table | Compound | Group | Calibration Curve

Peak#	Ret. Time	Area	Height	Mark	Conc.	Unit	ID#	Name	Area%
1	16. 237	8780687	400613	M	98. 462				98. 462
2	17. 358	137139	6317	V M	1. 538				1. 538
Total		8917826	406929		100. 000				100. 00

Supplementary Figure 228. HPLC spectrum of 1a

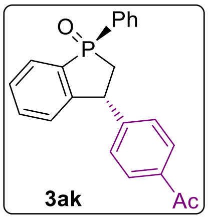

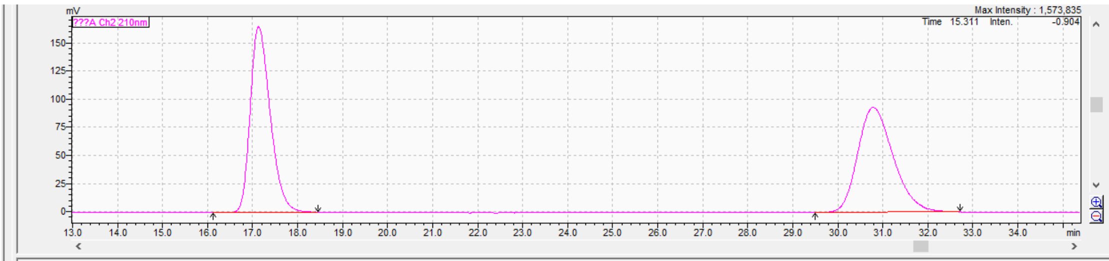

Results View - Peak Table

Peak Table | Compound | Group | Calibration Curve

Peak#	Ret. Time	Area	Height	Mark	Conc.	Unit	ID#	Name	Area%
1	17.134	5097715	165064	M	49.919				49.919
2	30.782	5114318	93228	M	50.081				50.081
Total		10212033	258292		100.000				100.00

Supplementary Figure 229. HPLC spectrum of racemic 3ak

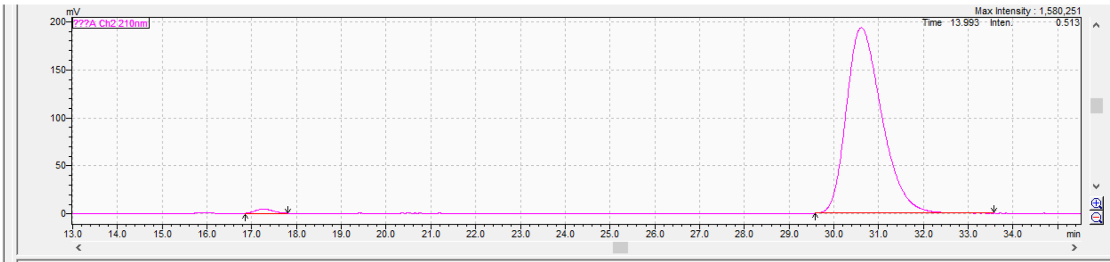

Results View - Peak Table

Peak Table | Compound | Group | Calibration Curve

Peak#	Ret. Time	Area	Height	Mark	Conc.	Unit	ID#	Name	Area%
1	17.250	122961	4439	M	1.147				1.147
2	30.612	10597403	193106	M	98.853				98.853
Total		10720364	197544		100.000				100.00

Supplementary Figure 230. HPLC spectrum of 3ak

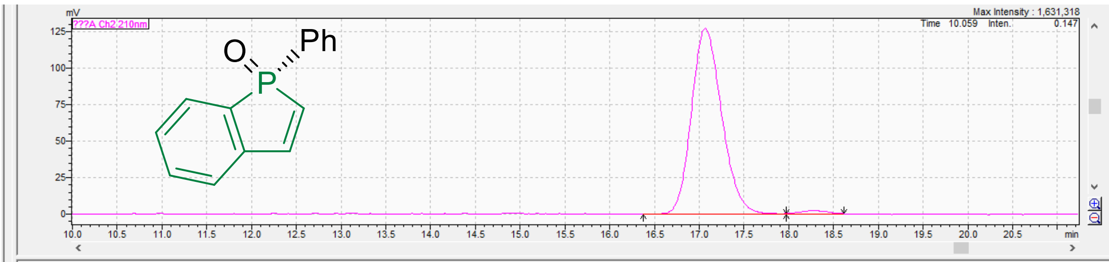

Results View - Peak Table

Peak Table | Compound | Group | Calibration Curve

Peak#	Ret. Time	Area	Height	Mark	Conc.	Unit	ID#	Name	Area%
1	17.066	2961296	126715	M	98.623				98.623
2	18.315	41343	2007	V M	1.377				1.377
Total		3002640	128722		100.000				100.00

Supplementary Figure 231. HPLC spectrum of 1a

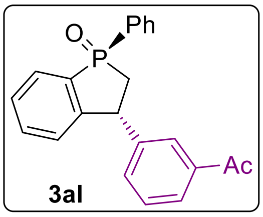

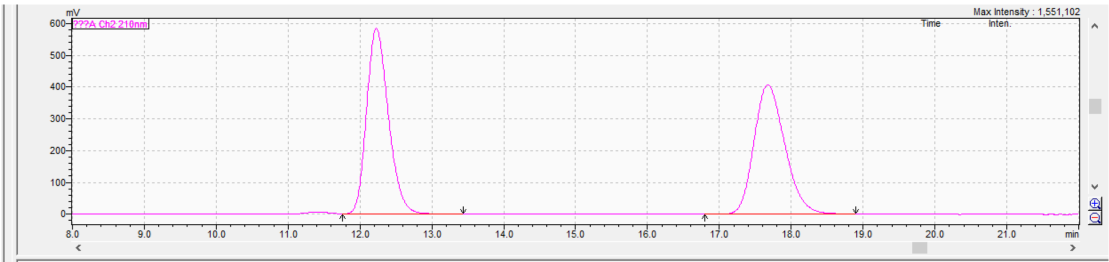

Peak#	Ret. Time	Area	Height	Mark	Conc.	Unit	ID#	Name	Area%
1	12. 226	12327142	583577	M	50. 366				50. 366
2	17. 674	12147767	407795	M	49. 634				49. 634
Total		24474909	991372		100. 000				100. 00

Supplementary Figure 232. HPLC spectrum of racemic 3al

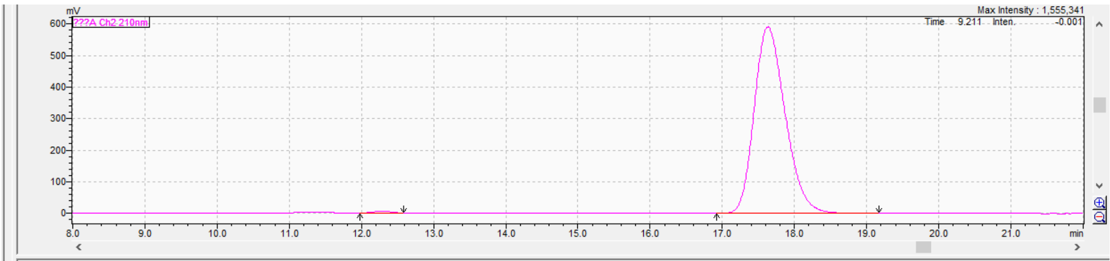

Peak#	Ret. Time	Area	Height	Mark	Conc.	Unit	ID#	Name	Area%
1	12. 272	114971	6282	M	0. 647				0. 647
2	17. 631	17641650	591084	M	99. 353				99. 353
Total		17756621	597367		100. 000				100. 00

Supplementary Figure 233. HPLC spectrum of 3al

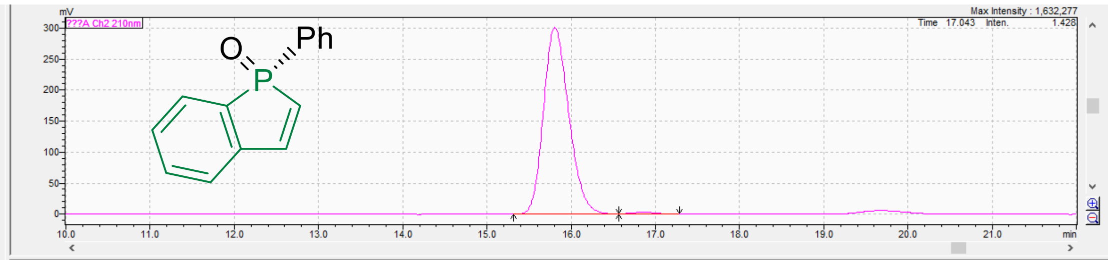

Peak#	Ret. Time	Area	Height	Mark	Conc.	Unit	ID#	Name	Area%
1	15. 806	6289580	300801	M	98. 981				98. 981
2	16. 882	64756	3031	V M	1. 019				1. 019
Total		6354336	303832		100. 000				100. 00

Supplementary Figure 234. HPLC spectrum of 1a

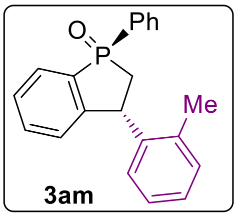

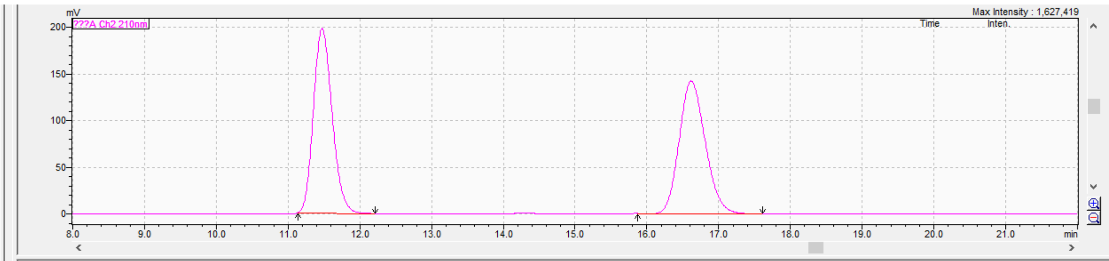

Results View - Peak Table

Peak Table | Compound | Group | Calibration Curve

Peak#	Ret. Time	Area	Height	Mark	Conc.	Unit	ID#	Name	Area%
1	11.473	3574143	197717	M	49.928				49.928
2	16.618	3584503	142168	M	50.072				50.072
Total		7158646	339884		100.000				100.00

Supplementary Figure 235. HPLC spectrum of racemic 3am

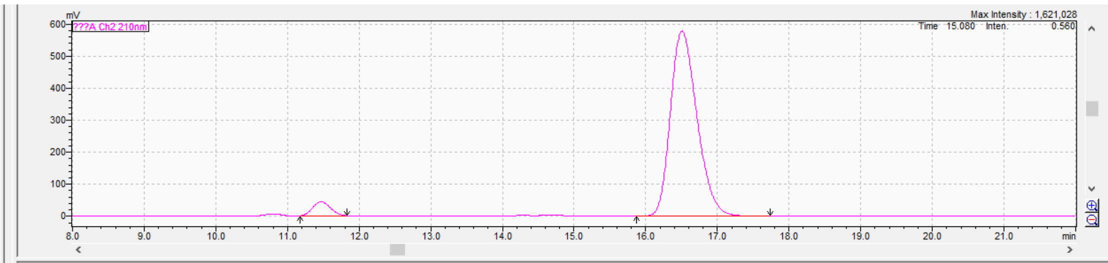

Results View - Peak Table

Peak Table | Compound | Group | Calibration Curve

Peak#	Ret. Time	Area	Height	Mark	Conc.	Unit	ID#	Name	Area%
1	11.468	750484	43973	M	4.809				4.809
2	16.505	14856661	577351	M	95.191				95.191
Total		15607145	621324		100.000				100.00

Supplementary Figure 236. HPLC spectrum of 3am

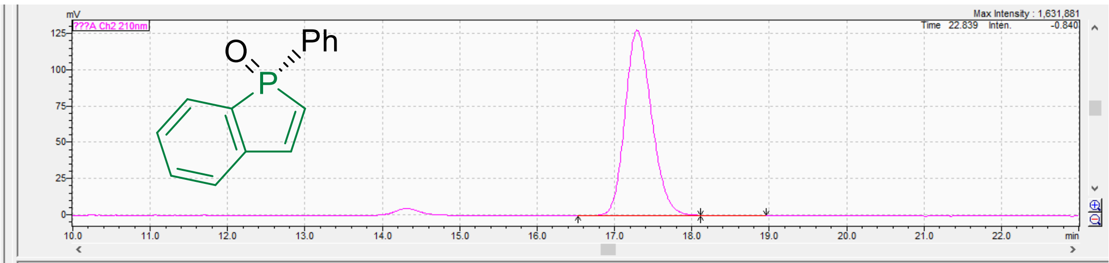

Results View - Peak Table

Peak Table | Compound | Group | Calibration Curve

Peak#	Ret. Time	Area	Height	Mark	Conc.	Unit	ID#	Name	Area%
1	17.293	3061326	127955	M	99.723				99.723
2	18.464	8498	328	V M	0.277				0.277
Total		3069824	128283		100.000				100.00

Supplementary Figure 237. HPLC spectrum of 1a

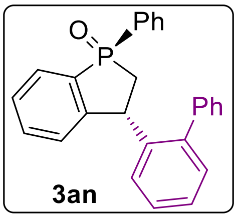

3an

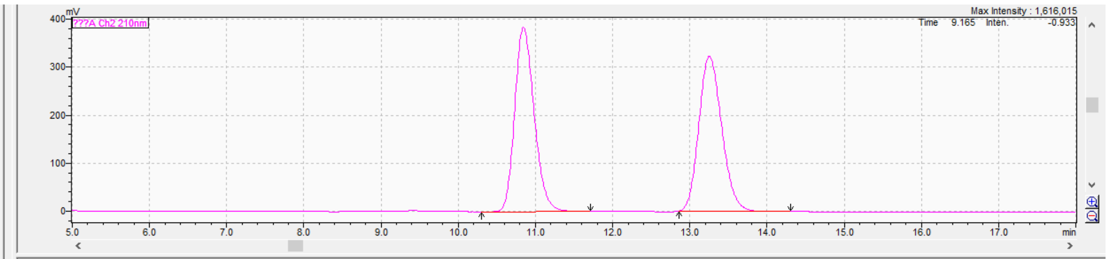

Peak#	Ret. Time	Area	Height	Mark	Conc.	Unit	ID#	Name	Area%
1	10. 845	6734788	384298	M	50. 256				50. 256
2	13. 253	6666298	323916	M	49. 744				49. 744
Total		13401087	708214		100. 000				100. 00

Supplementary Figure 238. HPLC spectrum of racemic 3an

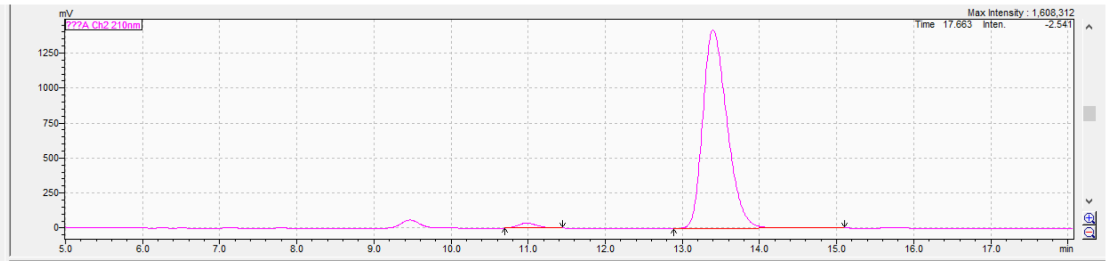

Peak#	Ret. Time	Area	Height	Mark	Conc.	Unit	ID#	Name	Area%
1	10. 985	603216	35139	M	1. 913				1. 913
2	13. 399	30931606	1415431	M	98. 087				98. 087
Total		31534822	1450570		100. 000				100. 00

Supplementary Figure 239. HPLC spectrum of 3an

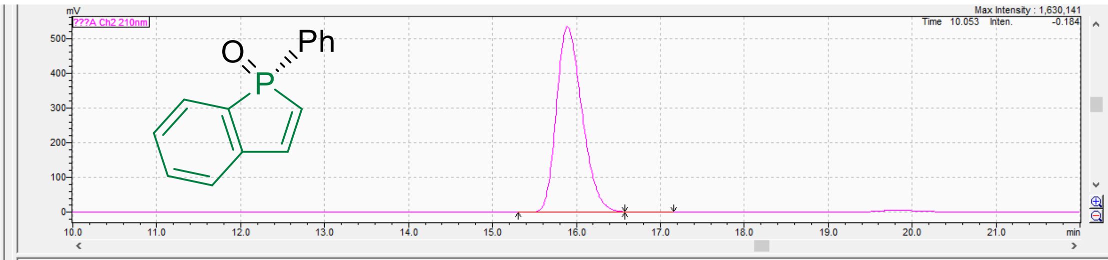

Peak#	Ret. Time	Area	Height	Mark	Conc.	Unit	ID#	Name	Area%
1	15. 894	11398749	534376	M	99. 843				99. 843
2	16. 588	17868	1609	V M	0. 157				0. 157
Total		11416618	535986		100. 000				100. 00

Supplementary Figure 240. HPLC spectrum of 1a

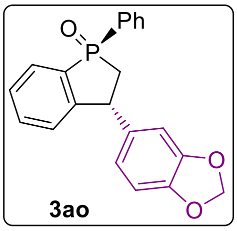

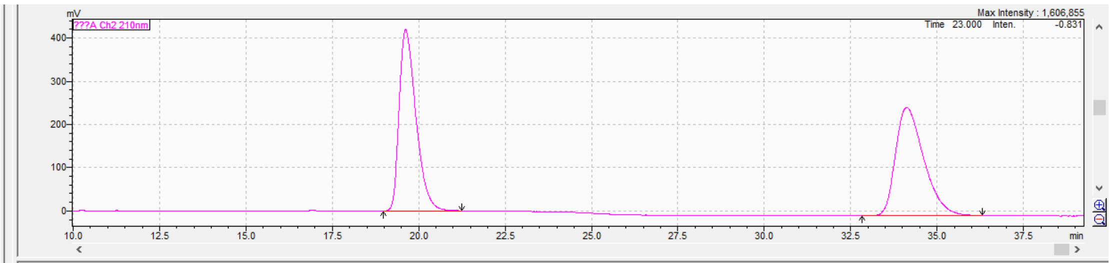

Results View - Peak Table

Peak Table | Compound | Group | Calibration Curve

Peak#	Ret. Time	Area	Height	Mark	Conc.	Unit	ID#	Name	Area%
1	19. 621	14131942	419775	M	49. 909				49. 909
2	34. 123	14183541	249791	M	50. 091				50. 091
Total		28315483	669566		100. 000				100. 00

Supplementary Figure 241. HPLC spectrum of racemic 3ao

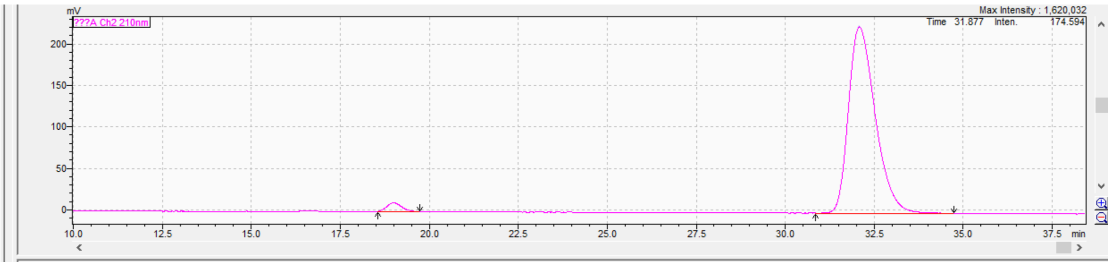

Results View - Peak Table

Peak Table | Compound | Group | Calibration Curve

Peak#	Ret. Time	Area	Height	Mark	Conc.	Unit	ID#	Name	Area%
1	19. 000	304955	10406	M	2. 530				2. 530
2	32. 079	11746861	224786	M	97. 470				97. 470
Total		12051816	235193		100. 000				100. 00

Supplementary Figure 242. HPLC spectrum of 3ao

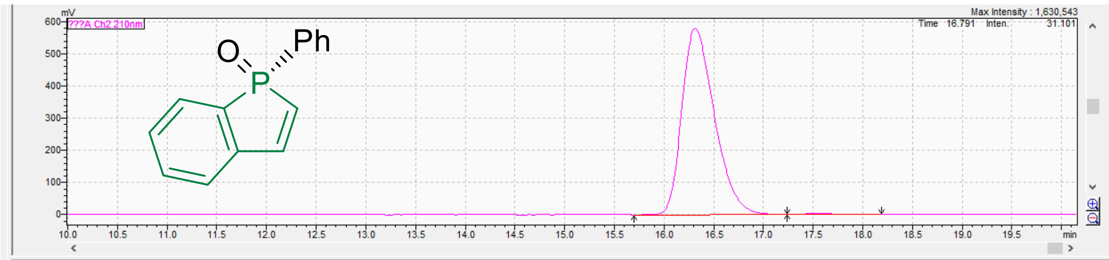

Results View - Peak Table

Peak Table | Compound | Group | Calibration Curve

Peak#	Ret. Time	Area	Height	Mark	Conc.	Unit	ID#	Name	Area%
1	16. 313	13189935	579187	M	99. 415				99. 415
2	17. 541	77666	3259	V M	0. 585				0. 585
Total		13267601	582446		100. 000				100. 00

Supplementary Figure 243. HPLC spectrum of 1a

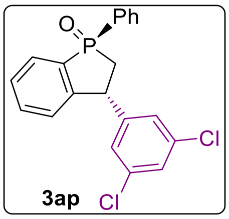

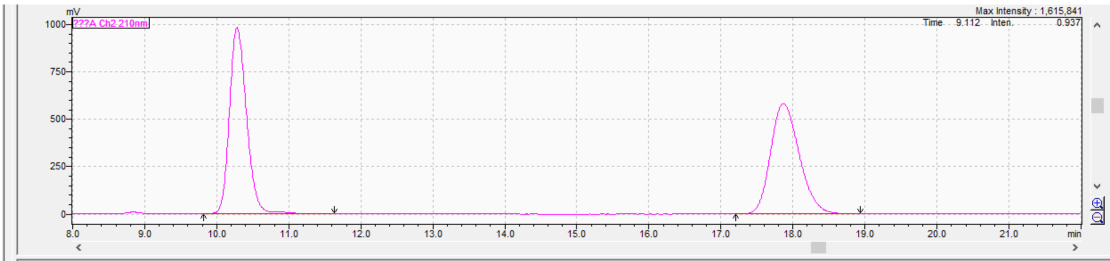

Results View - Peak Table

Peak Table | Compound | Group | Calibration Curve

Peak#	Ret. Time	Area	Height	Mark	Conc.	Unit	ID#	Name	Area%
1	10. 282	16200509	982641	M	50. 161				50. 161
2	17. 867	16096299	581381	M	49. 839				49. 839
Total		32296808	1564022		100. 000				100. 00

Supplementary Figure 244. HPLC spectrum of racemic 3ap

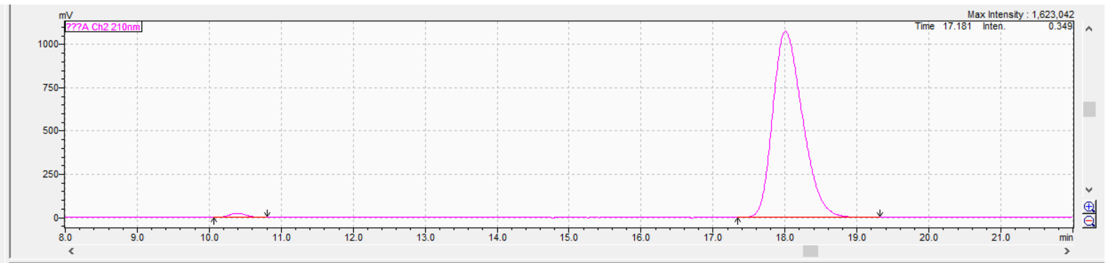

Results View - Peak Table

Peak Table | Compound | Group | Calibration Curve

Peak#	Ret. Time	Area	Height	Mark	Conc.	Unit	ID#	Name	Area%
1	10. 390	411331	24489	M	1. 316				1. 316
2	18. 006	30842905	1076203	M	98. 684				98. 684
Total		31254236	1100692		100. 000				100. 00

Supplementary Figure 245. HPLC spectrum of 3ap

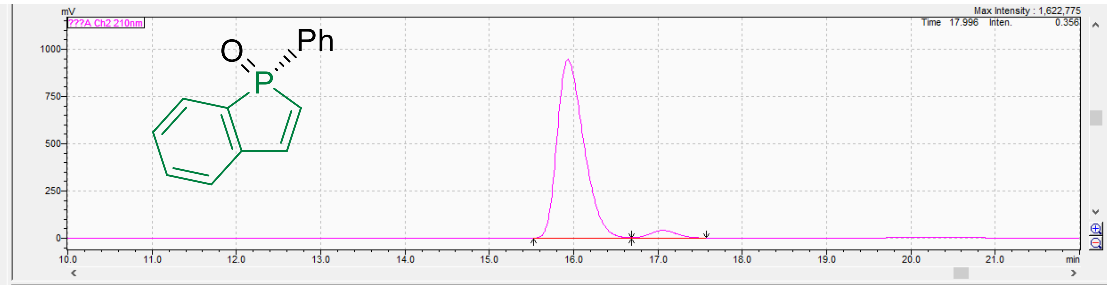

Results View - Peak Table

Peak Table | Compound | Group | Calibration Curve

Peak#	Ret. Time	Area	Height	Mark	Conc.	Unit	ID#	Name	Area%
1	15. 932	20750008	945746	M	95. 784				95. 784
2	17. 056	913251	40525	V M	4. 216				4. 216
Total		21663258	986271		100. 000				100. 00

Supplementary Figure 246. HPLC spectrum of 1a

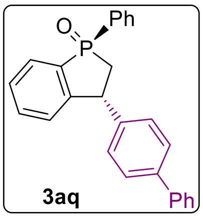

3aq

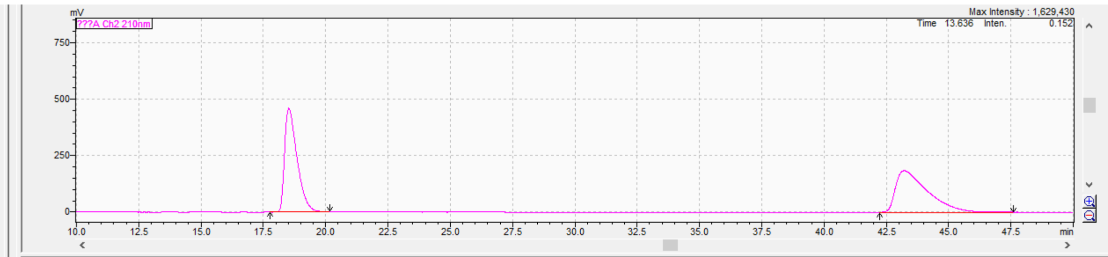

Results View - Peak Table

Peak Table | Compound | Group | Calibration Curve

Peak#	Ret. Time	Area	Height	Mark	Conc.	Unit	ID#	Name	Area%
1	18.522	15942446	459193	M	49.543				49.543
2	43.220	16236854	183753	M	50.457				50.457
Total		32179300	642947		100.000				100.00

Supplementary Figure 247. HPLC spectrum of racemic 3aq

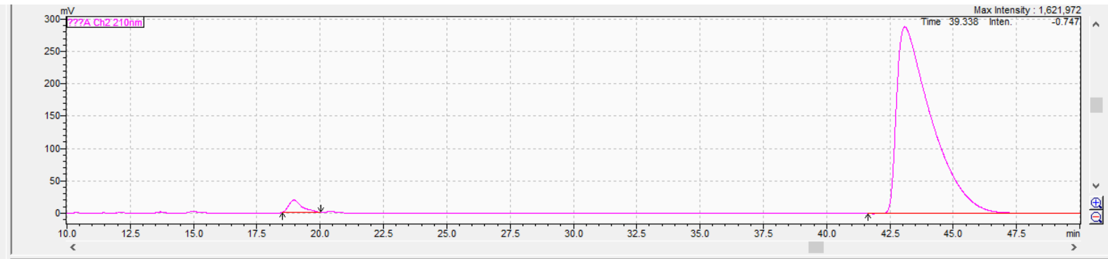

Results View - Peak Table

Peak Table | Compound | Group | Calibration Curve

Peak#	Ret. Time	Area	Height	Mark	Conc.	Unit	ID#	Name	Area%
1	18.956	678965	18821	M	2.397				2.397
2	43.090	27647415	289006	M	97.603				97.603
Total		28326380	307828		100.000				100.00

Supplementary Figure 248. HPLC spectrum of 3aq

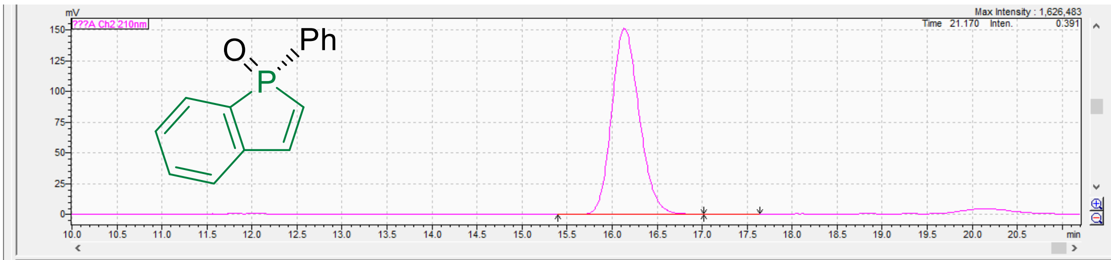

Results View - Peak Table

Peak Table | Compound | Group | Calibration Curve

Peak#	Ret. Time	Area	Height	Mark	Conc.	Unit	ID#	Name	Area%
1	16.136	3210341	151140	M	99.920				99.920
2	17.349	2576	158	V M	0.080				0.080
Total		3212917	151298		100.000				100.00

Supplementary Figure 249. HPLC spectrum of 1a

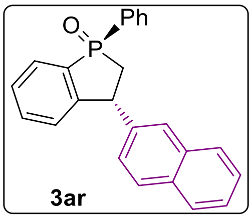

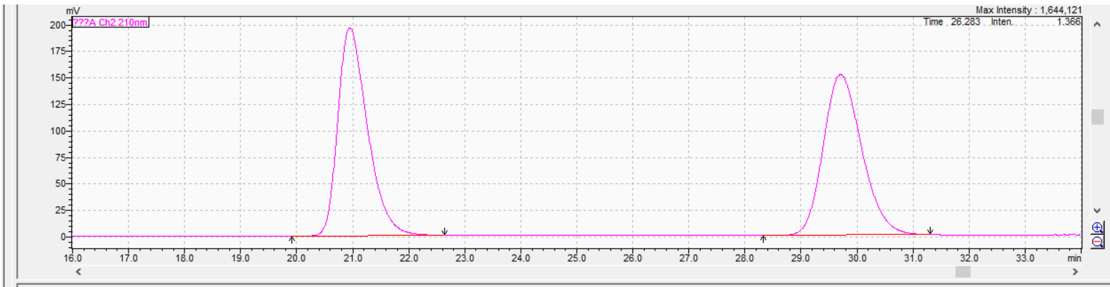

Results View - Peak Table

Peak Table | Compound | Group | Calibration Curve

Peak#	Ret. Time	Area	Height	Mark	Conc.	Unit	ID#	Name	Area%
1	20. 947	7205802	196196	M	49. 841				49. 841
2	29. 707	7251694	151582	M	50. 159				50. 159
Total		14457497	347778		100. 000				100. 00

Supplementary Figure 250. HPLC spectrum of racemic 3ar

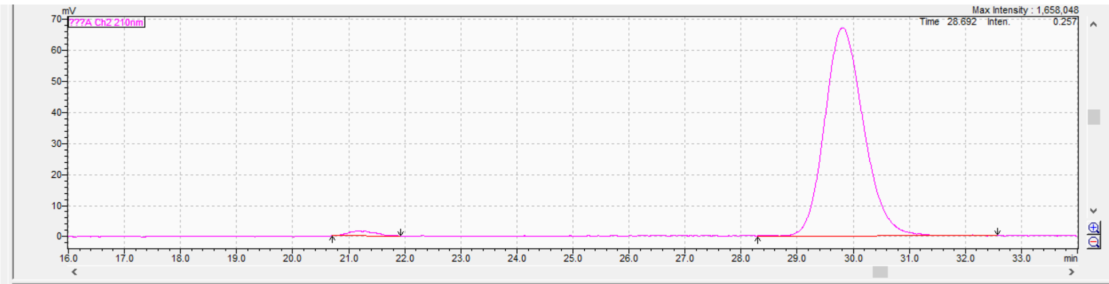

Results View - Peak Table

Peak Table | Compound | Group | Calibration Curve

Peak#	Ret. Time	Area	Height	Mark	Conc.	Unit	ID#	Name	Area%
1	21. 285	54754	1531	M	1. 677				1. 677
2	29. 813	3210790	66842	M	98. 323				98. 323
Total		3265545	68373		100. 000				100. 00

Supplementary Figure 251. HPLC spectrum of 3ar

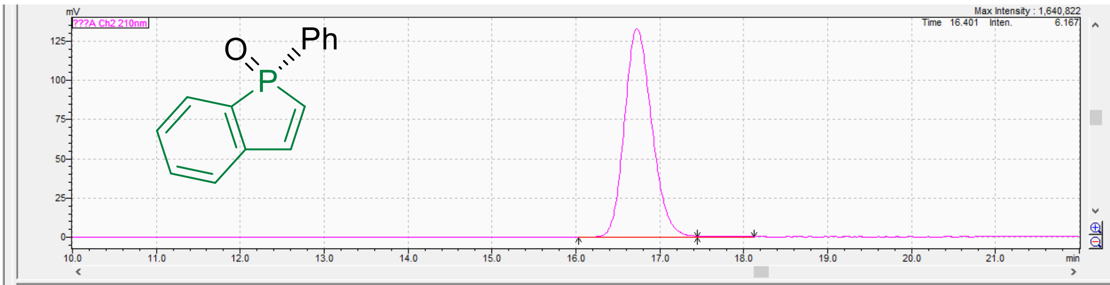

Results View - Peak Table

Peak Table | Compound | Group | Calibration Curve

Peak#	Ret. Time	Area	Height	Mark	Conc.	Unit	ID#	Name	Area%
1	16. 728	3009853	132838	M	99. 761				99. 761
2	17. 467	7202	375	V M	0. 239				0. 239
Total		3017055	133213		100. 000				100. 00

Supplementary Figure 252. HPLC spectrum of 1a

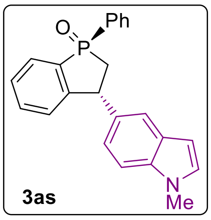

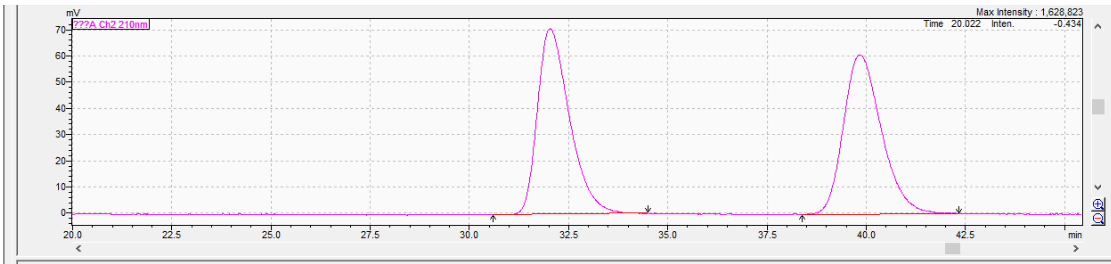

Results View - Peak Table

Peak Table | Compound | Group | Calibration Curve

Peak#	Ret. Time	Area	Height	Mark	Conc.	Unit	ID#	Name	Area%
1	32.033	3977225	70705	M	49.664				49.664
2	39.834	4031068	60782	M	50.336				50.336
Total		8008293	131487		100.000				100.00

Supplementary Figure 253. HPLC spectrum of racemic 3as

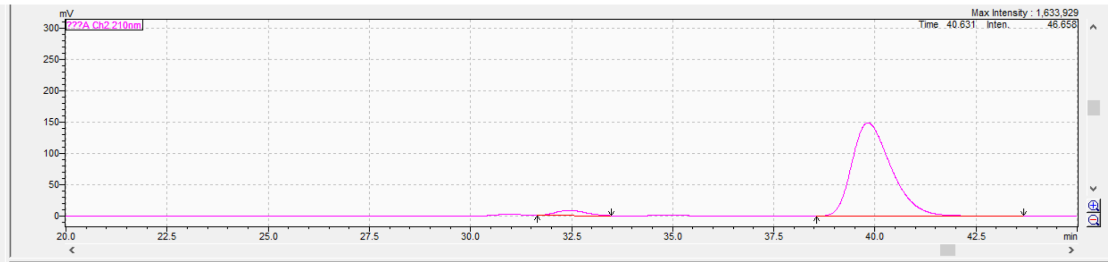

Results View - Peak Table

Peak Table | Compound | Group | Calibration Curve

Peak#	Ret. Time	Area	Height	Mark	Conc.	Unit	ID#	Name	Area%
1	32.415	417947	8231	M	4.004				4.004
2	39.823	10020102	149014	M	95.996				95.996
Total		10438049	157245		100.000				100.00

Supplementary Figure 254. HPLC spectrum of 3as

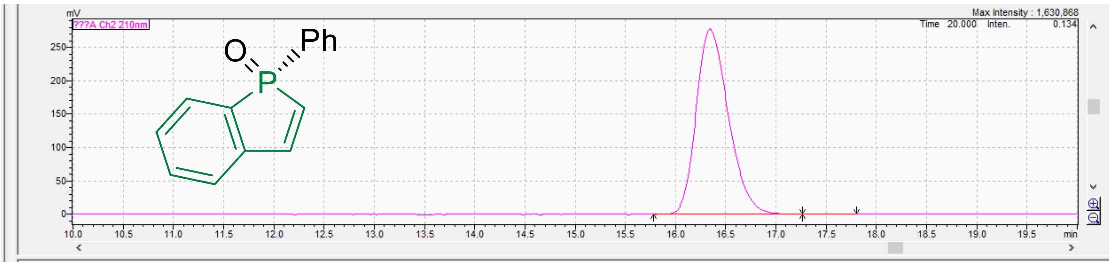

Results View - Peak Table

Peak Table | Compound | Group | Calibration Curve

Peak#	Ret. Time	Area	Height	Mark	Conc.	Unit	ID#	Name	Area%
1	16.344	6209529	277732	M	99.899				99.899
2	17.406	6273	378	V M	0.101				0.101
Total		6215802	278110		100.000				100.00

Supplementary Figure 255. HPLC spectrum of 1a

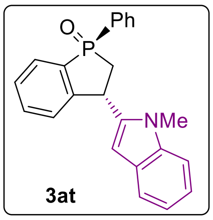

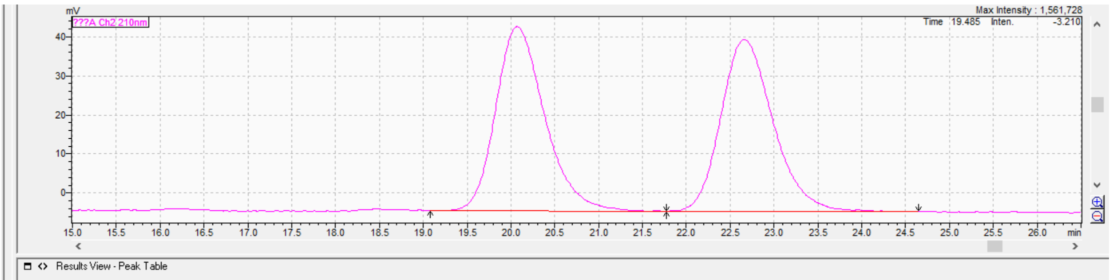

Peak#	Ret. Time	Area	Height	Mark	Conc.	Unit	ID#	Name	Area%
1	20.072	1839469	47165	M	50.192				50.192
2	22.661	1825431	44055	M	49.808				49.808
Total		3664900	91220		100.000				100.00

Supplementary Figure 256. HPLC spectrum of racemic 3at

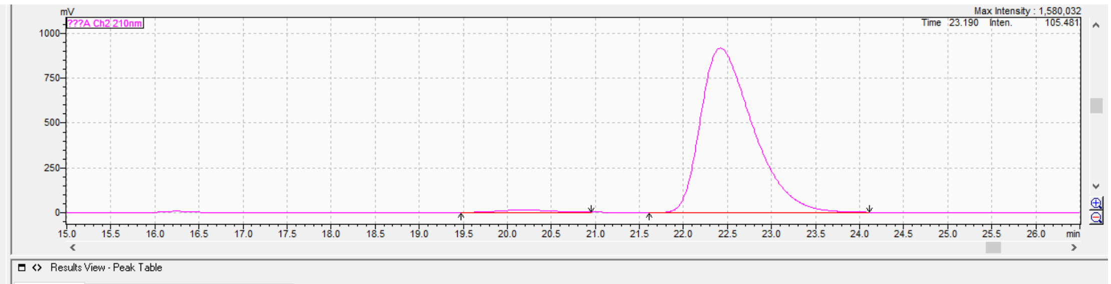

Peak#	Ret. Time	Area	Height	Mark	Conc.	Unit	ID#	Name	Area%
1	20.177	621473	14510	M	1.618				1.618
2	22.420	37784556	916629	M	98.382				98.382
Total		38406029	931139		100.000				100.00

Supplementary Figure 257. HPLC spectrum of 3at

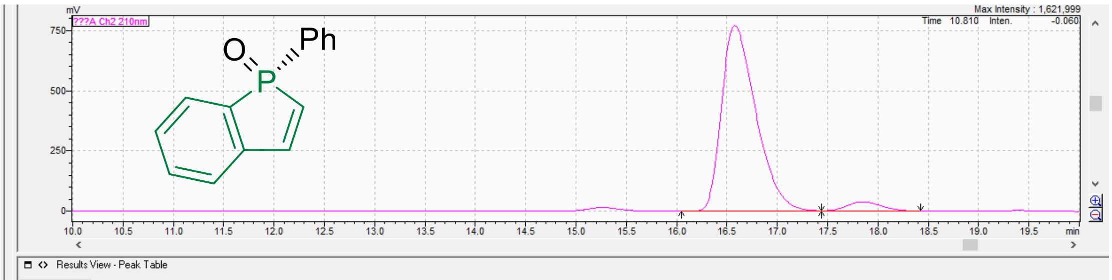

Peak#	Ret. Time	Area	Height	Mark	Conc.	Unit	ID#	Name	Area%
1	16.578	18313715	768970	M	95.033				95.033
2	17.849	957247	39222	V M	4.967				4.967
Total		19270962	808192		100.000				100.00

Supplementary Figure 258. HPLC spectrum of 1a

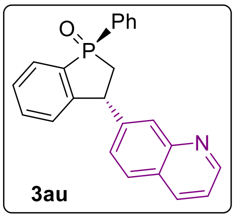

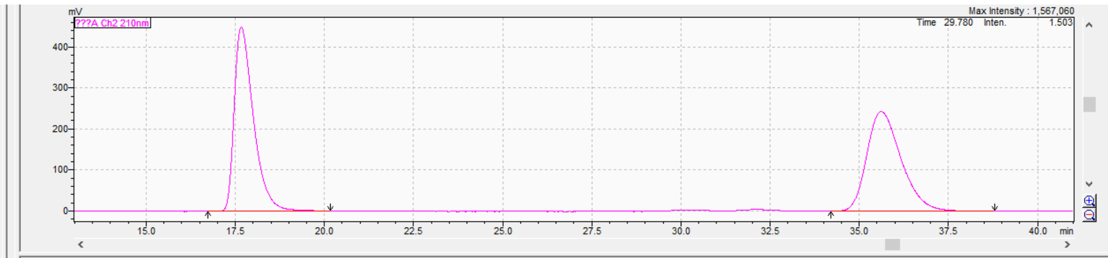

Peak#	Ret. Time	Area	Height	Mark	Conc.	Unit	ID#	Name	Area%
1	17.671	16763049	448041	M	50.820				50.820
2	35.614	16221861	242697	M	49.180				49.180
Total		32984910	690739		100.000				100.00

Supplementary Figure 259. HPLC spectrum of racemic 3au

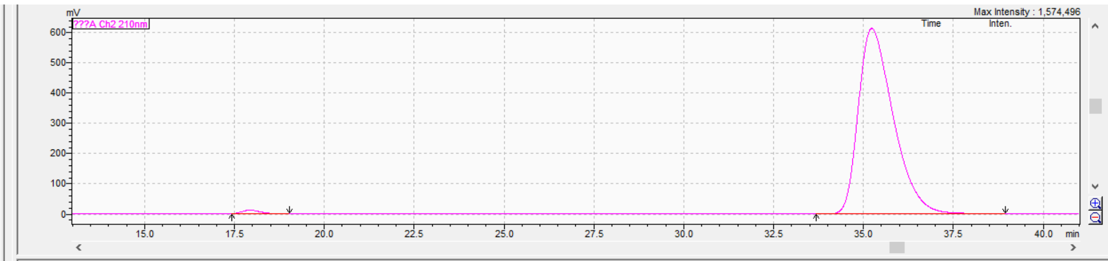

Peak#	Ret. Time	Area	Height	Mark	Conc.	Unit	ID#	Name	Area%
1	17.934	446914	12217	M	1.065				1.065
2	35.231	41502876	614185	M	98.935				98.935
Total		41949790	626402		100.000				100.00

Supplementary Figure 260. HPLC spectrum of 3au

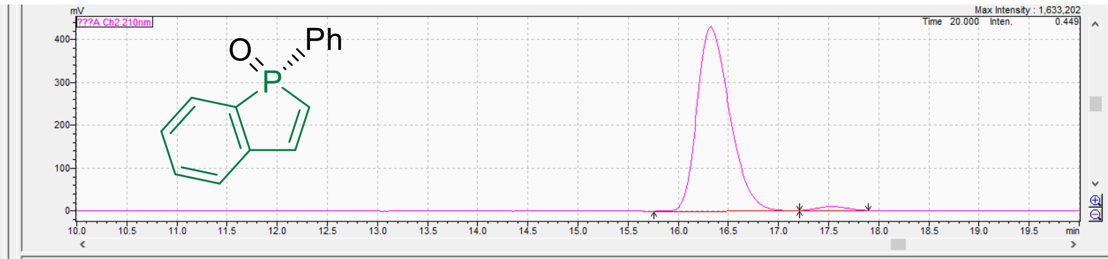

Peak#	Ret. Time	Area	Height	Mark	Conc.	Unit	ID#	Name	Area%
1	16.324	9703799	430321	M	97.889				97.889
2	17.525	209255	9789	V M	2.111				2.111
Total		9913054	440110		100.000				100.00

Supplementary Figure 261. HPLC spectrum of 1a

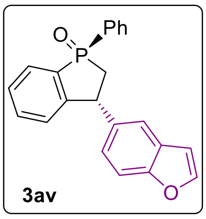

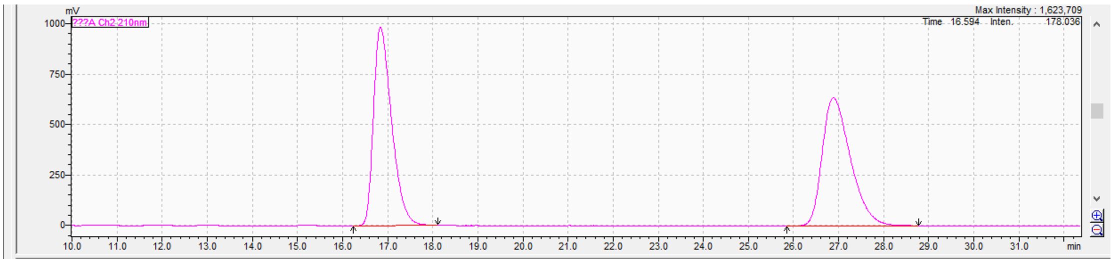

Peak#	Ret. Time	Area	Height	Mark	Conc.	Unit	ID#	Name	Area%
1	16. 842	27468596	982792	M	49. 836				49. 836
2	26. 885	27649384	634485	M	50. 164				50. 164
Total		55117981	1617278		100. 000				100. 00

Supplementary Figure 262. HPLC spectrum of racemic 3av

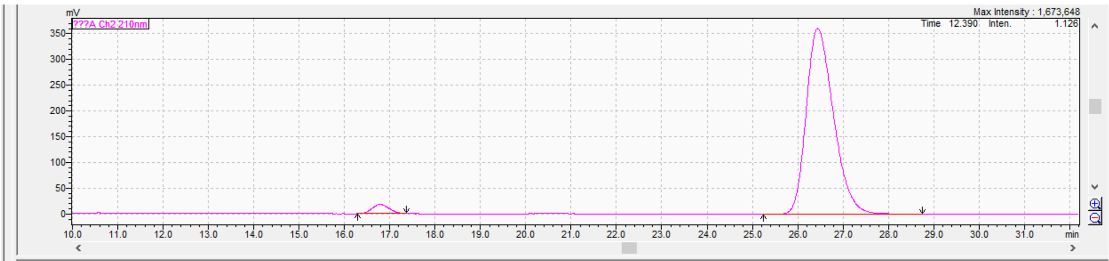

Peak#	Ret. Time	Area	Height	Mark	Conc.	Unit	ID#	Name	Area%
1	16. 786	464101	17922	M	2. 979				2. 979
2	26. 443	15112902	360940	M	97. 021				97. 021
Total		15577003	378863		100. 000				100. 00

Supplementary Figure 263. HPLC spectrum of 3av

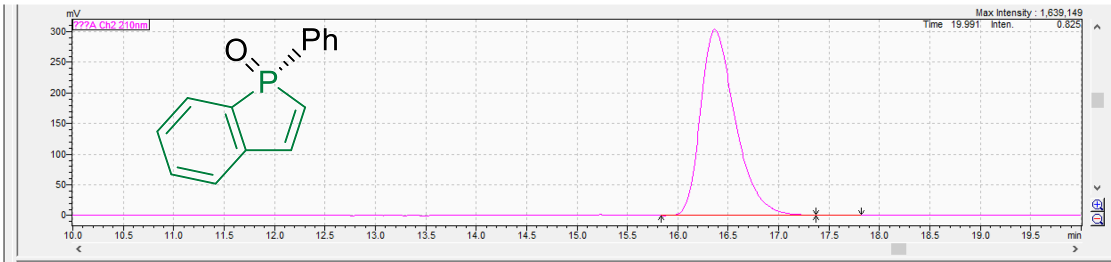

Peak#	Ret. Time	Area	Height	Mark	Conc.	Unit	ID#	Name	Area%
1	16. 366	7260381	303706	M	99. 913				99. 913
2	17. 371	6313	493	V M	0. 087				0. 087
Total		7266695	304199		100. 000				100. 00

Supplementary Figure 264. HPLC spectrum of 1a

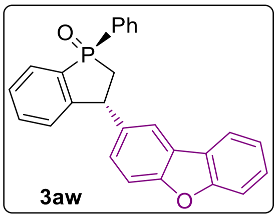

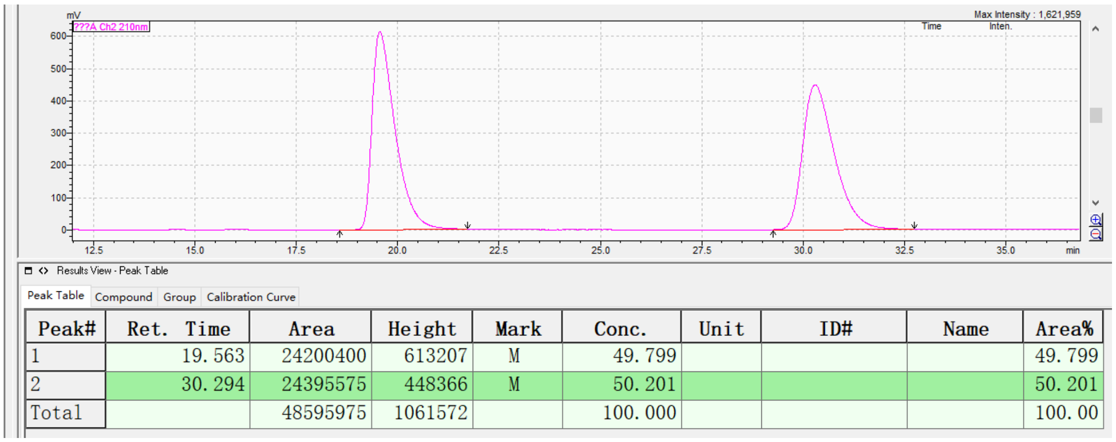

Peak#	Ret. Time	Area	Height	Mark	Conc.	Unit	ID#	Name	Area%
1	19.563	24200400	613207	M	49.799				49.799
2	30.294	24395575	448366	M	50.201				50.201
Total		48595975	1061572		100.000				100.00

Supplementary Figure 265. HPLC spectrum of racemic 3aw

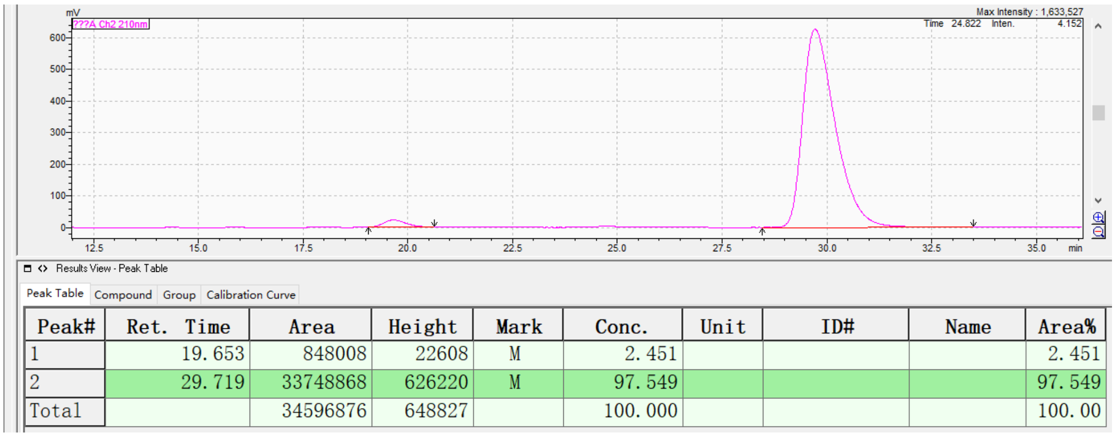

Peak#	Ret. Time	Area	Height	Mark	Conc.	Unit	ID#	Name	Area%
1	19.653	848008	22608	M	2.451				2.451
2	29.719	33748868	626220	M	97.549				97.549
Total		34596876	648827		100.000				100.00

Supplementary Figure 266. HPLC spectrum of racemic 3aw

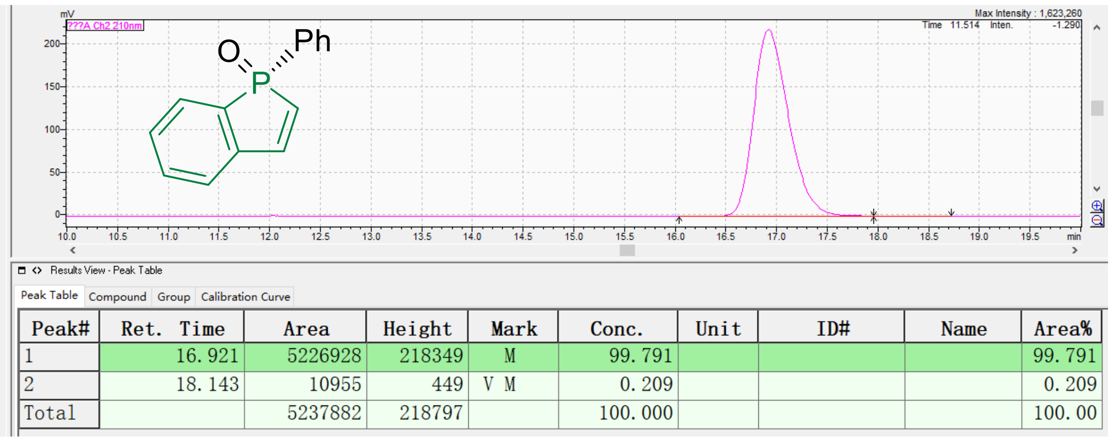

Peak#	Ret. Time	Area	Height	Mark	Conc.	Unit	ID#	Name	Area%
1	16.921	5226928	218349	M	99.791				99.791
2	18.143	10955	449	V M	0.209				0.209
Total		5237882	218797		100.000				100.00

Supplementary Figure 267. HPLC spectrum of 1a

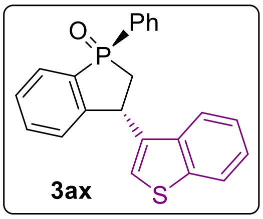

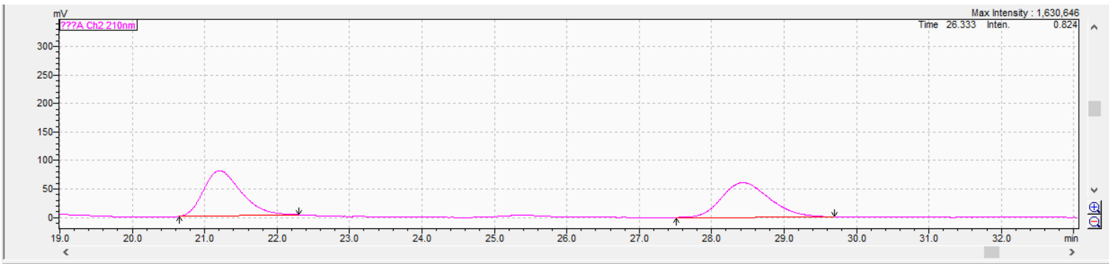

Peak#	Ret. Time	Area	Height	Mark	Conc.	Unit	ID#	Name	Area%
1	21. 203	2838770	79038	M	50. 415				50. 415
2	28. 425	2792050	60609	M	49. 585				49. 585
Total		5630819	139647		100. 000				100. 00

Supplementary Figure 268. HPLC spectrum of racemic 3ax

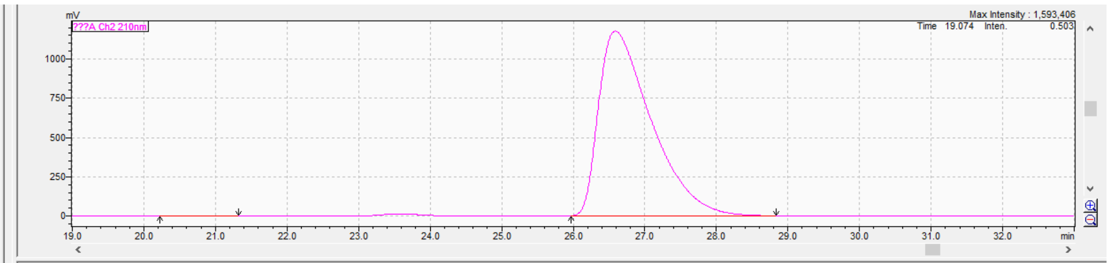

Peak#	Ret. Time	Area	Height	Mark	Conc.	Unit	ID#	Name	Area%
1	20. 683	82500	2382	M	0. 137				0. 137
2	26. 589	60180978	1176888	M	99. 863				99. 863
Total		60263477	1179271		100. 000				100. 00

Supplementary Figure 269. HPLC spectrum of 3ax

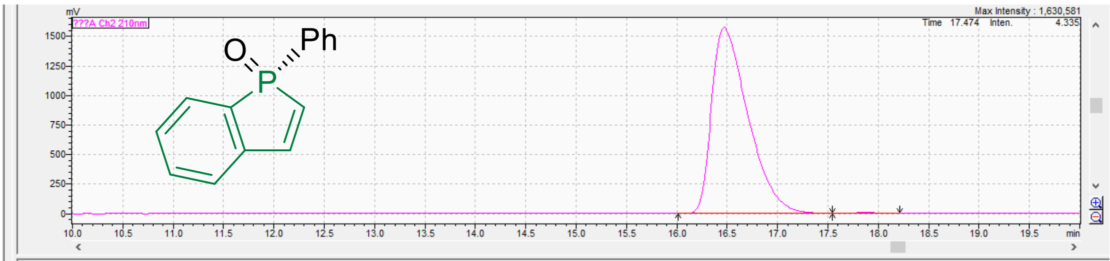

Peak#	Ret. Time	Area	Height	Mark	Conc.	Unit	ID#	Name	Area%
1	16. 471	39615253	1573847	M	99. 666				99. 666
2	17. 880	132704	5874	V M	0. 334				0. 334
Total		39747958	1579720		100. 000				100. 00

Supplementary Figure 270. HPLC spectrum of 1a

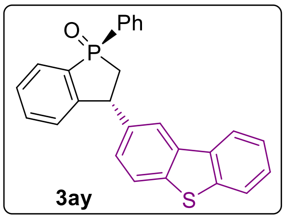

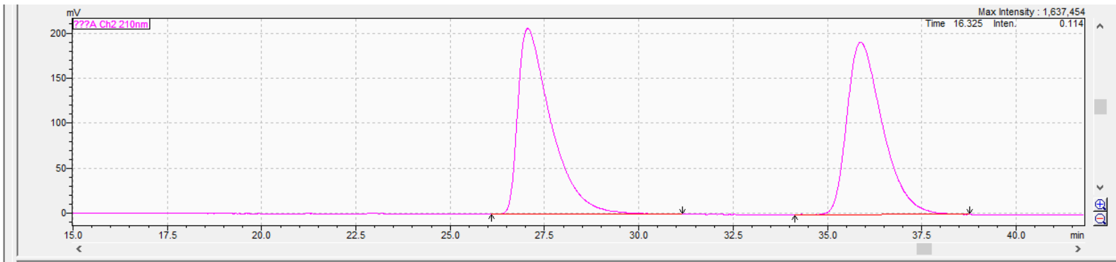

Peak#	Ret. Time	Area	Height	Mark	Conc.	Unit	ID#	Name	Area%
1	27.057	12598438	205854	M	50.117				50.117
2	35.879	12539528	191296	M	49.883				49.883
Total		25137966	397150		100.000				100.00

Supplementary Figure 271. HPLC spectrum of racemic 3ay

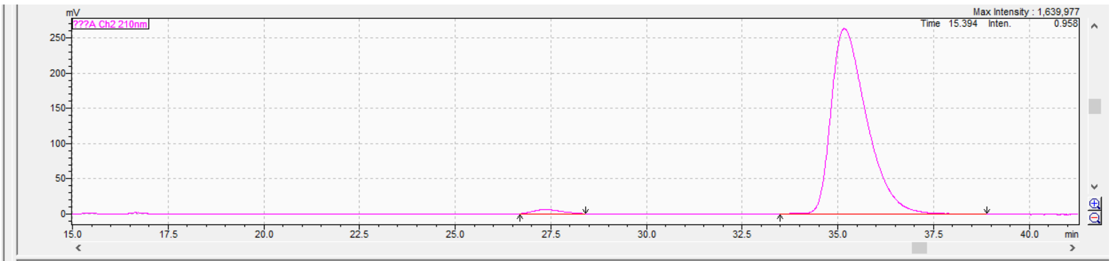

Peak#	Ret. Time	Area	Height	Mark	Conc.	Unit	ID#	Name	Area%
1	27.366	288547	5791	M	1.665				1.665
2	35.166	17042032	263518	M	98.335				98.335
Total		17330578	269310		100.000				100.00

Supplementary Figure 272. HPLC spectrum of 3ay

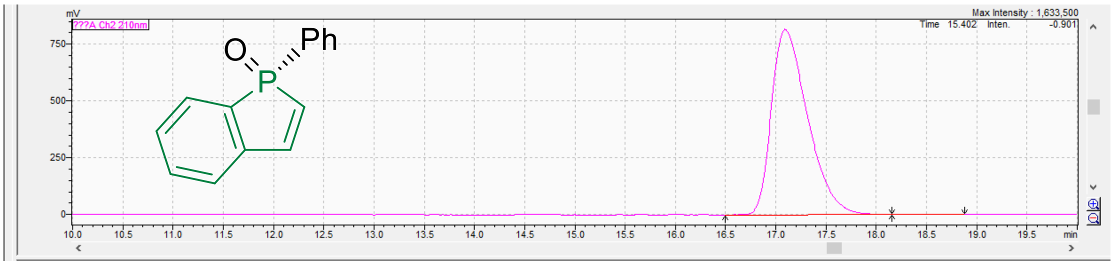

Peak#	Ret. Time	Area	Height	Mark	Conc.	Unit	ID#	Name	Area%
1	17.093	20454249	816003	M	99.651				99.651
2	18.487	71732	2876	V M	0.349				0.349
Total		20525980	818880		100.000				100.00

Supplementary Figure 273. HPLC spectrum of 1a

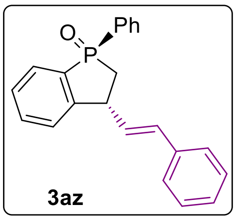

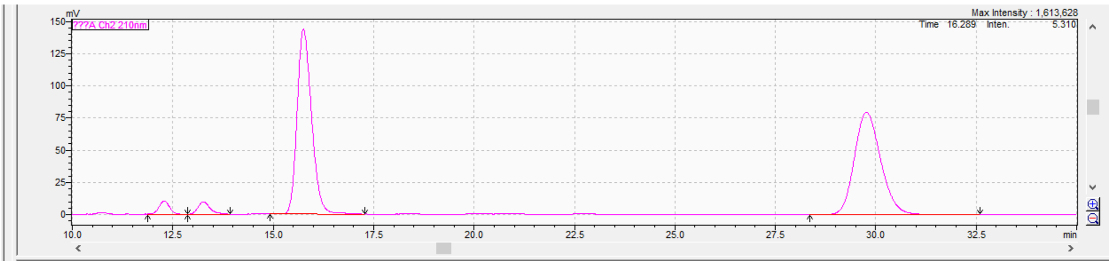

Peak#	Ret. Time	Area	Height	Mark	Conc.	Unit	ID#	Name	Area%
1	12. 289	185744	10388	M	2. 454				2. 454
2	13. 261	205632	9701	M	2. 717				2. 717
3	15. 755	3576413	143318	M	47. 249				47. 249
4	29. 766	3601470	79561	M	47. 580				47. 580
Total		7569260	242967		100. 000				100. 00

Supplementary Figure 274. HPLC spectrum of racemic 3az

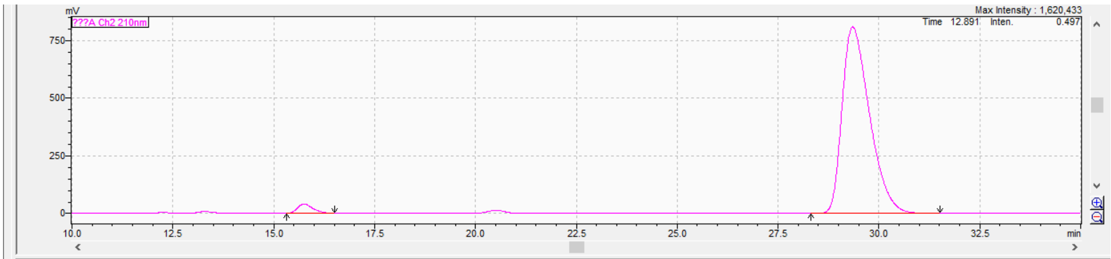

Peak#	Ret. Time	Area	Height	Mark	Conc.	Unit	ID#	Name	Area%
1	15. 753	1049886	40571	M	2. 669				2. 669
2	29. 350	38286199	809342	M	97. 331				97. 331
Total		39336086	849913		100. 000				100. 00

Supplementary Figure 275. HPLC spectrum of 3az

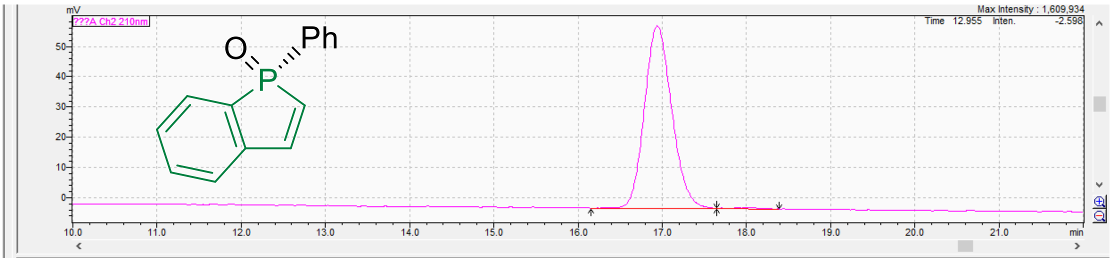

Peak#	Ret. Time	Area	Height	Mark	Conc.	Unit	ID#	Name	Area%
1	16. 941	1363442	60334	M	99. 397				99. 397
2	18. 057	8265	415	V M	0. 603				0. 603
Total		1371706	60750		100. 000				100. 00

Supplementary Figure 276. HPLC spectrum of 1a

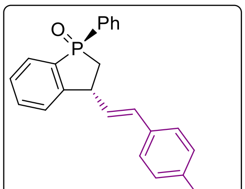

3az-2

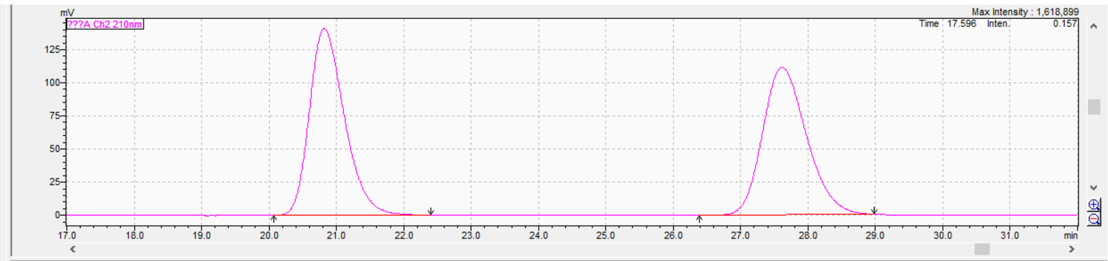

Results View - Peak Table

Peak Table | Compound | Group | Calibration Curve

Peak#	Ret. Time	Area	Height	Mark	Conc.	Unit	ID#	Name	Area%
1	20. 820	5077603	140717	M	50. 140				50. 140
2	27. 614	5049247	111171	M	49. 860				49. 860
Total		10126850	251888		100. 000				100. 00

Supplementary Figure 277. HPLC spectrum of racemic 3az-2

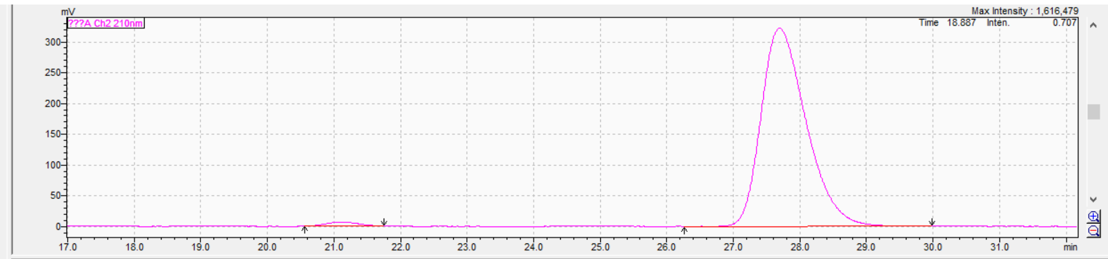

Results View - Peak Table

Peak Table | Compound | Group | Calibration Curve

Peak#	Ret. Time	Area	Height	Mark	Conc.	Unit	ID#	Name	Area%
1	21. 124	224827	6679	M	1. 465				1. 465
2	27. 695	15122606	322020	M	98. 535				98. 535
Total		15347433	328699		100. 000				100. 00

Supplementary Figure 278. HPLC spectrum of 3az-2

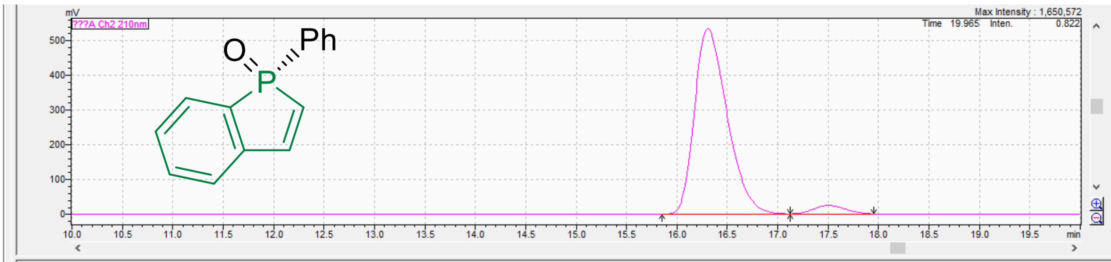

Results View - Peak Table

Peak Table | Compound | Group | Calibration Curve

Peak#	Ret. Time	Area	Height	Mark	Conc.	Unit	ID#	Name	Area%
1	16. 307	12067419	534435	M	95. 615				95. 615
2	17. 505	553458	24846	V M	4. 385				4. 385
Total		12620877	559281		100. 000				100. 00

Supplementary Figure 279. HPLC spectrum of 1a

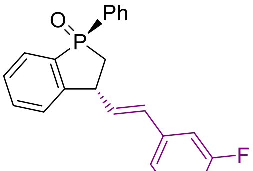

3az-3

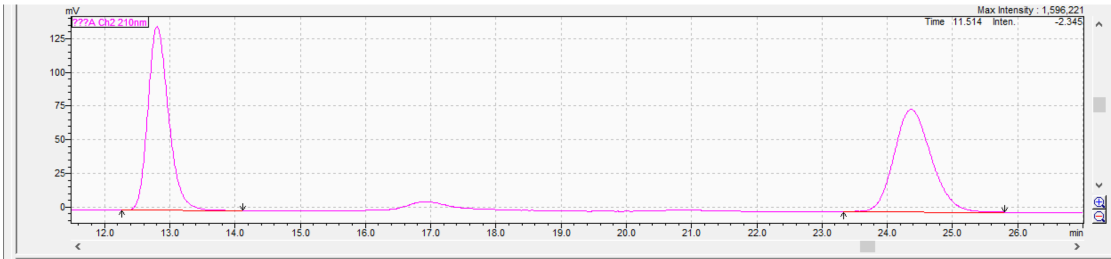

Results View - Peak Table

Peak Table | Compound | Group | Calibration Curve

Peak#	Ret. Time	Area	Height	Mark	Conc.	Unit	ID#	Name	Area%
1	12.805	3015774	136094	M	50.018				50.018
2	24.369	3013635	76059	M	49.982				49.982
Total		6029410	212153		100.000				100.00

Supplementary Figure 280. HPLC spectrum of racemic 3az-3

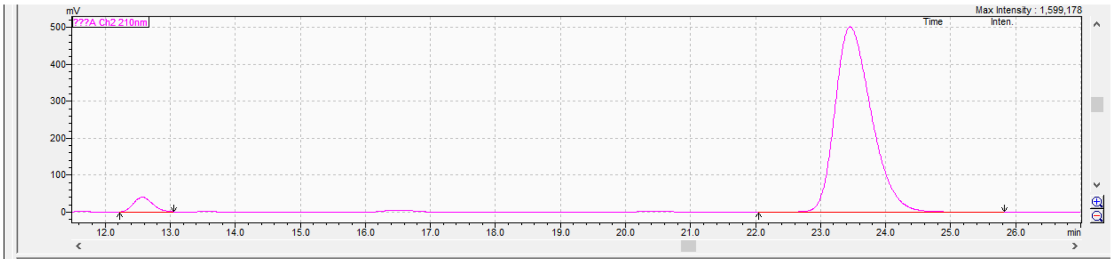

Results View - Peak Table

Peak Table | Compound | Group | Calibration Curve

Peak#	Ret. Time	Area	Height	Mark	Conc.	Unit	ID#	Name	Area%
1	12.572	813470	39775	M	3.994				3.994
2	23.456	19554848	502570	M	96.006				96.006
Total		20368317	542345		100.000				100.00

Supplementary Figure 281. HPLC spectrum of 3az-3

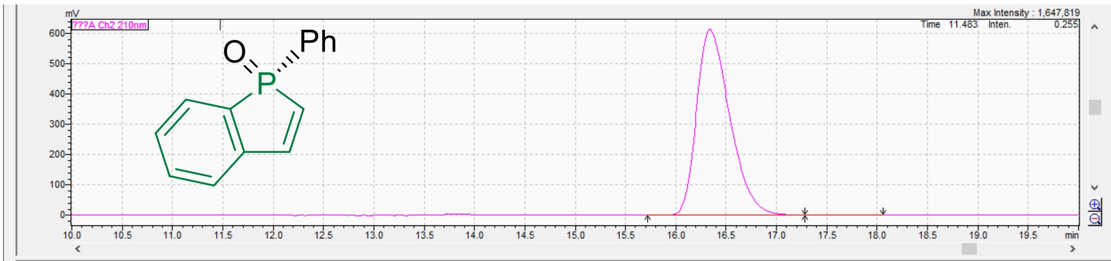

Results View - Peak Table

Peak Table | Compound | Group | Calibration Curve

Peak#	Ret. Time	Area	Height	Mark	Conc.	Unit	ID#	Name	Area%
1	16.339	14075497	613972	M	99.895				99.895
2	17.318	14725	792	V M	0.105				0.105
Total		14090222	614764		100.000				100.00

Supplementary Figure 282. HPLC spectrum of 1a

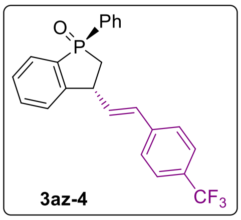

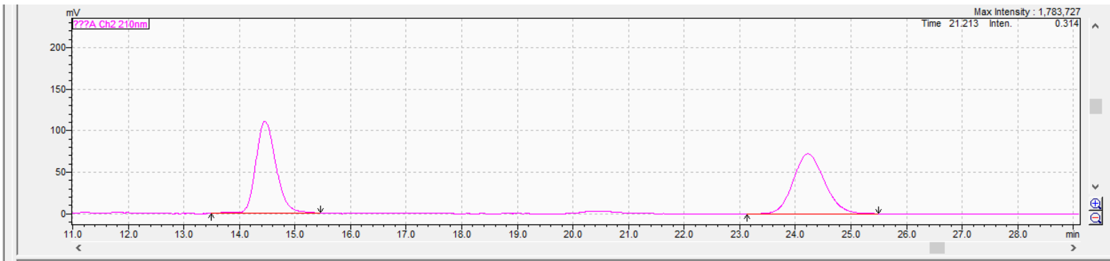

Peak#	Ret. Time	Area	Height	Mark	Conc.	Unit	ID#	Name	Area%
1	14. 461	2806414	110956	M	49. 841				49. 841
2	24. 227	2824276	72093	M	50. 159				50. 159
Total		5630690	183049		100. 000				100. 00

Supplementary Figure 283. HPLC spectrum of racemic 3az-4

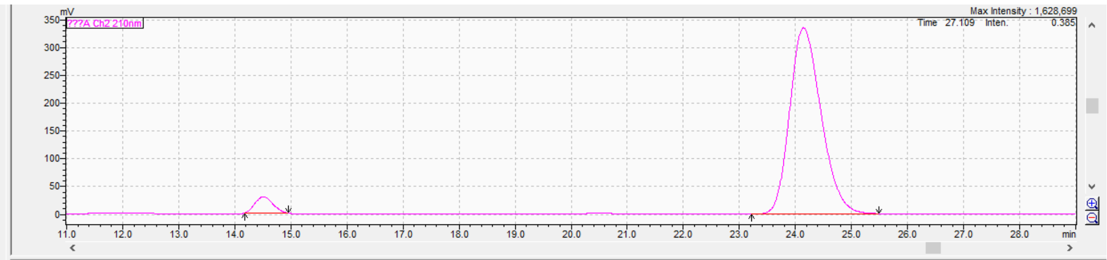

Peak#	Ret. Time	Area	Height	Mark	Conc.	Unit	ID#	Name	Area%
1	14. 507	646183	29177	M	4. 733				4. 733
2	24. 144	13005314	334896	M	95. 267				95. 267
Total		13651498	364072		100. 000				100. 00

Supplementary Figure 284. HPLC spectrum of 3az-4

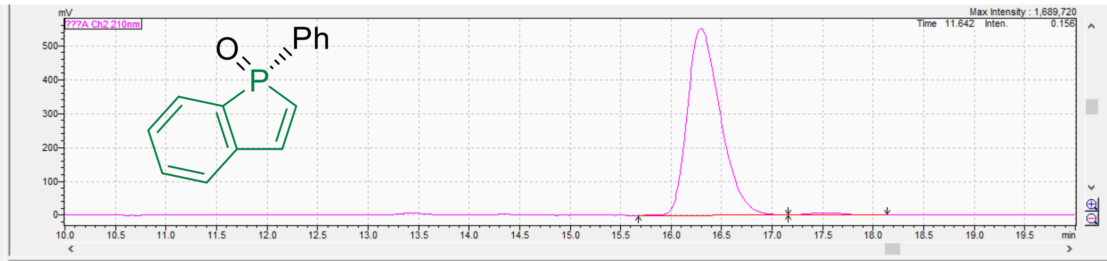

Peak#	Ret. Time	Area	Height	Mark	Conc.	Unit	ID#	Name	Area%
1	16. 294	12535992	550988	M	98. 541				98. 541
2	17. 517	185554	7598	V M	1. 459				1. 459
Total		12721546	558586		100. 000				100. 00

Supplementary Figure 285. HPLC spectrum of 1a

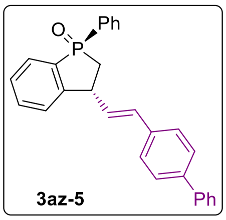

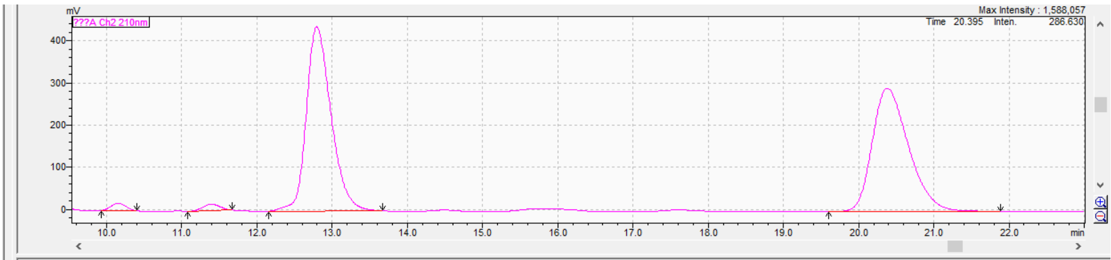

Results View - Peak Table

Peak Table | Compound | Group | Calibration Curve

Peak#	Ret. Time	Area	Height	Mark	Conc.	Unit	ID#	Name	Area%
1	10. 153	240041	17368	M	1. 180				1. 180
2	11. 396	238450	15129	M	1. 173				1. 173
3	12. 795	9997483	437420	M	49. 162				49. 162
4	20. 382	9859783	292694	M	48. 485				48. 485
Total		20335757	762612		100. 000				100. 00

Supplementary Figure 286. HPLC spectrum of racemic 3az-5

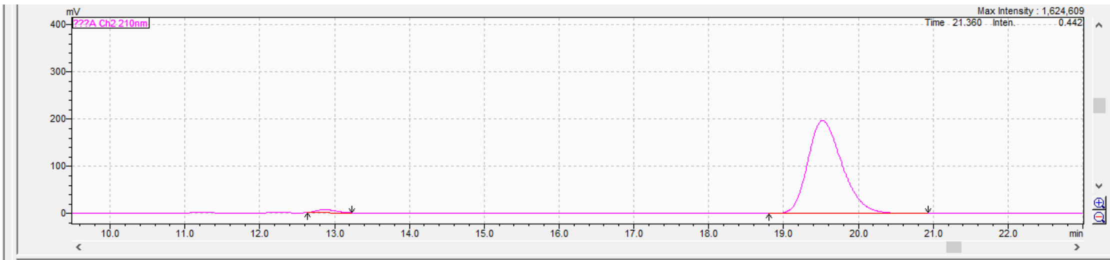

Results View - Peak Table

Peak Table | Compound | Group | Calibration Curve

Peak#	Ret. Time	Area	Height	Mark	Conc.	Unit	ID#	Name	Area%
1	12. 881	117135	6271	M	1. 836				1. 836
2	19. 521	6262273	196886	M	98. 164				98. 164
Total		6379408	203157		100. 000				100. 00

Supplementary Figure 287. HPLC spectrum of 3az-5

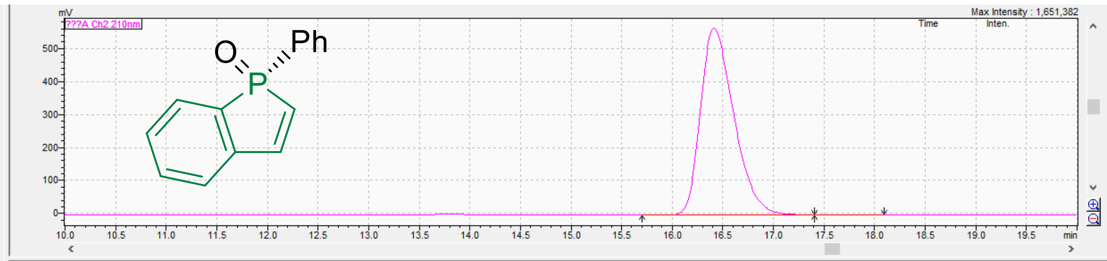

Results View - Peak Table

Peak Table | Compound | Group | Calibration Curve

Peak#	Ret. Time	Area	Height	Mark	Conc.	Unit	ID#	Name	Area%
1	16. 413	12994666	565471	M	99. 855				99. 855
2	17. 627	18828	820	V M	0. 145				0. 145
Total		13013494	566291		100. 000				100. 00

Supplementary Figure 288. HPLC spectrum of 1a

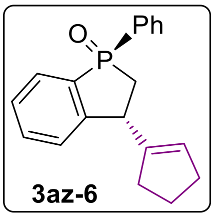

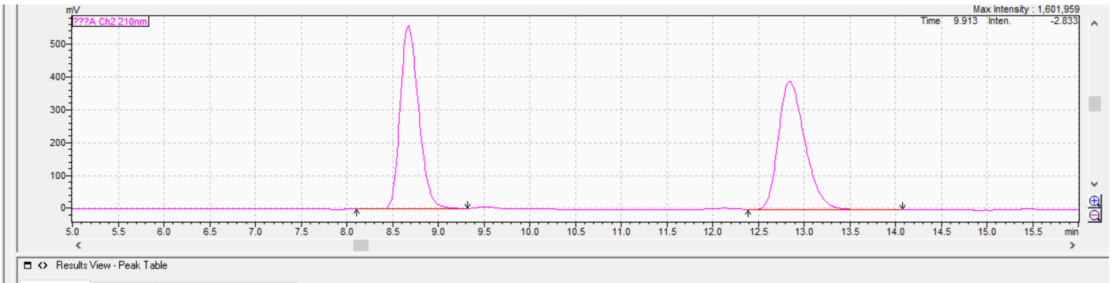

Peak#	Ret. Time	Area	Height	Mark	Conc.	Unit	ID#	Name	Area%
1	8.672	7788591	557026	M	49.671				49.671
2	12.842	7891794	391124	M	50.329				50.329
Total		15680385	948150		100.000				100.00

Supplementary Figure 289. HPLC spectrum of racemic 3az-6

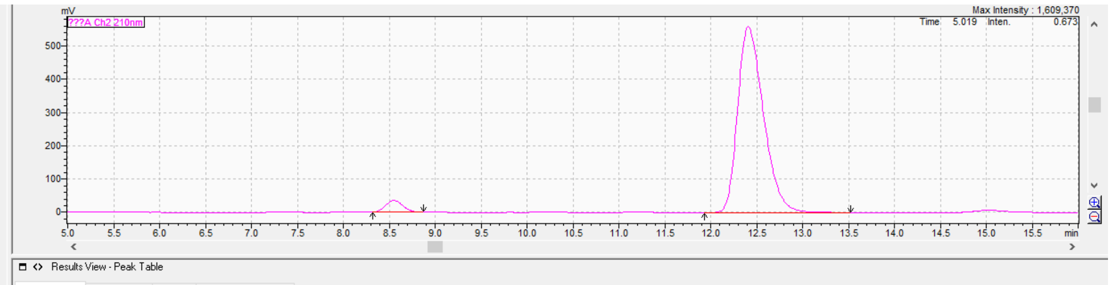

Peak#	Ret. Time	Area	Height	Mark	Conc.	Unit	ID#	Name	Area%
1	8.550	456871	35679	M	4.026				4.026
2	12.411	10891415	558772	M	95.974				95.974
Total		11348285	594451		100.000				100.00

Supplementary Figure 290. HPLC spectrum of 3az-6

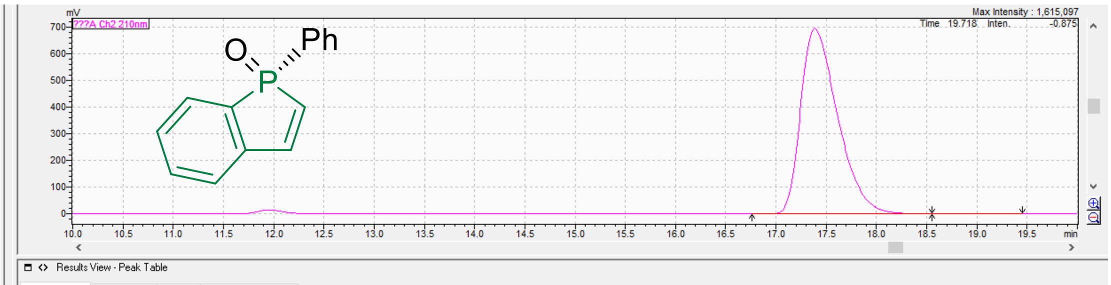

Peak#	Ret. Time	Area	Height	Mark	Conc.	Unit	ID#	Name	Area%
1	17.387	17787520	695537	M	99.800				99.800
2	18.813	35611	1226	V M	0.200				0.200
Total		17823132	696763		100.000				100.00

Supplementary Figure 291. HPLC spectrum of 1a

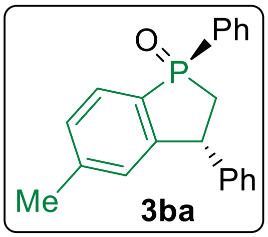

3ba

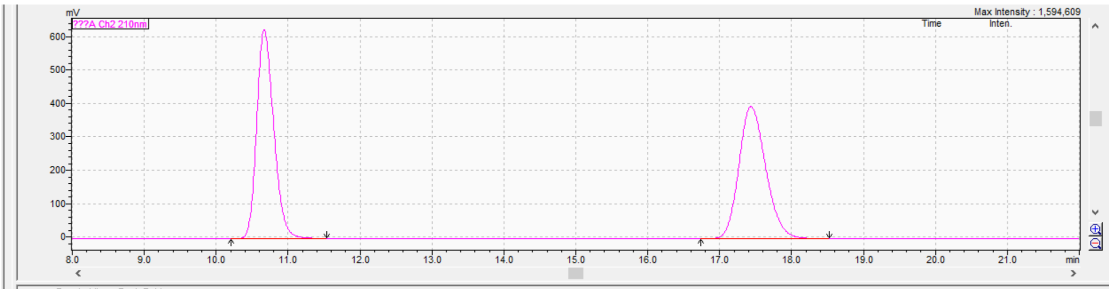

Results View - Peak Table

Peak Table | Compound | Group | Calibration Curve

Peak#	Ret. Time	Area	Height	Mark	Conc.	Unit	ID#	Name	Area%
1	10. 668	10316892	625550	M	50. 120				50. 120
2	17. 435	10267550	395639	M	49. 880				49. 880
Total		20584442	1021189		100. 000				100. 00

Supplementary Figure 292. HPLC spectrum of racemic 3ba

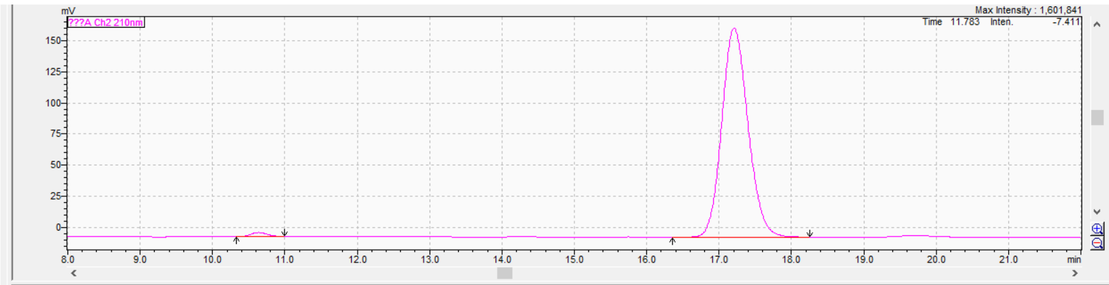

Results View - Peak Table

Peak Table | Compound | Group | Calibration Curve

Peak#	Ret. Time	Area	Height	Mark	Conc.	Unit	ID#	Name	Area%
1	10. 637	52223	3325	M	1. 220				1. 220
2	17. 208	4227650	168054	M	98. 780				98. 780
Total		4279873	171378		100. 000				100. 00

Supplementary Figure 293. HPLC spectrum of 3ba

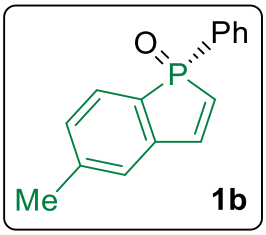

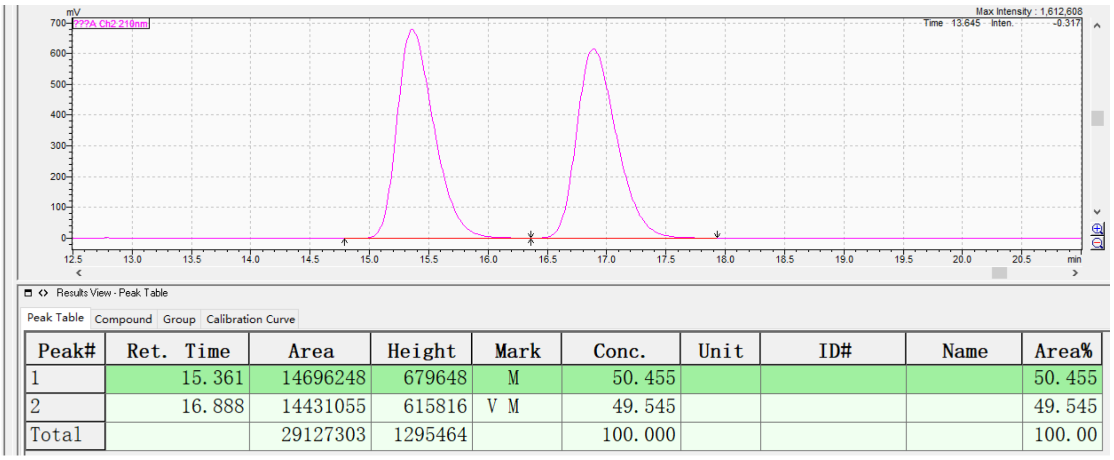

Peak#	Ret. Time	Area	Height	Mark	Conc.	Unit	ID#	Name	Area%
1	15. 361	14696248	679648	M	50. 455				50. 455
2	16. 888	14431055	615816	V M	49. 545				49. 545
Total		29127303	1295464		100. 000				100. 00

Supplementary Figure 294. HPLC spectrum of racemic 1b

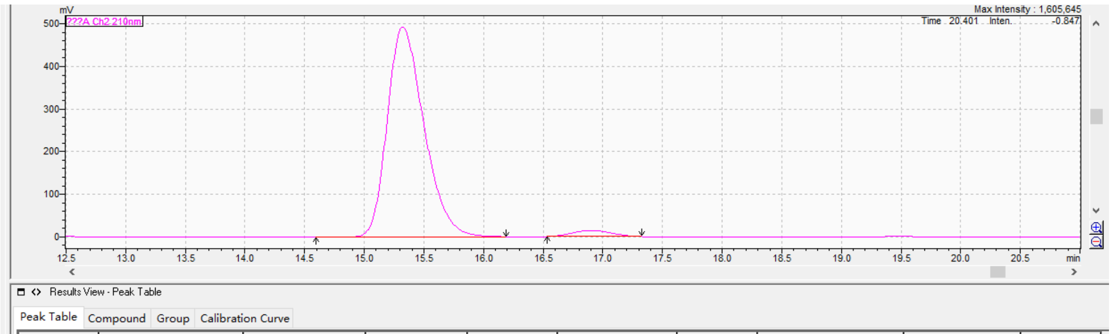

Peak#	Ret. Time	Area	Height	Mark	Conc.	Unit	ID#	Name	Area%
1	15. 322	10558527	492975	M	97. 002				97. 002
2	16. 900	326296	14483	M	2. 998				2. 998
Total		10884823	507458		100. 000				100. 00

Supplementary Figure 295. HPLC spectrum of 1b

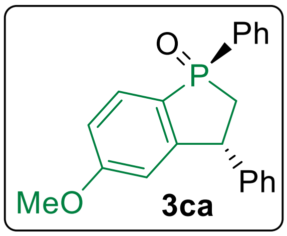

3ca

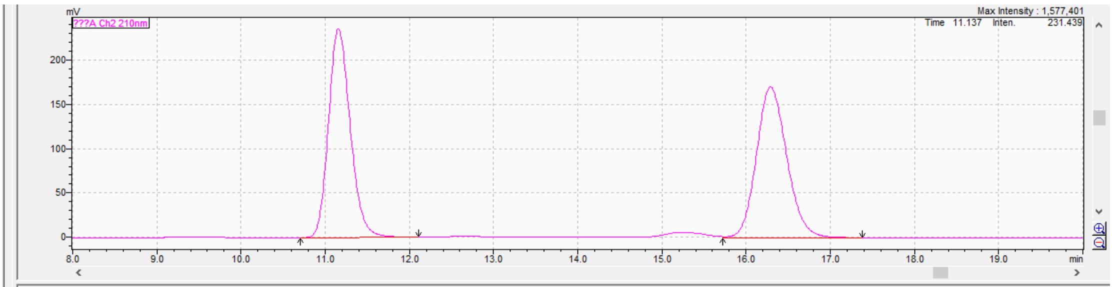

Results View - Peak Table

Peak Table | Compound | Group | Calibration Curve

Peak#	Ret. Time	Area	Height	Mark	Conc.	Unit	ID#	Name	Area%
1	11. 157	4222602	235282	M	49. 862				49. 862
2	16. 291	4246013	169649	M	50. 138				50. 138
Total		8468615	404931		100. 000				100. 00

Supplementary Figure 296. HPLC spectrum of racemic 3ca

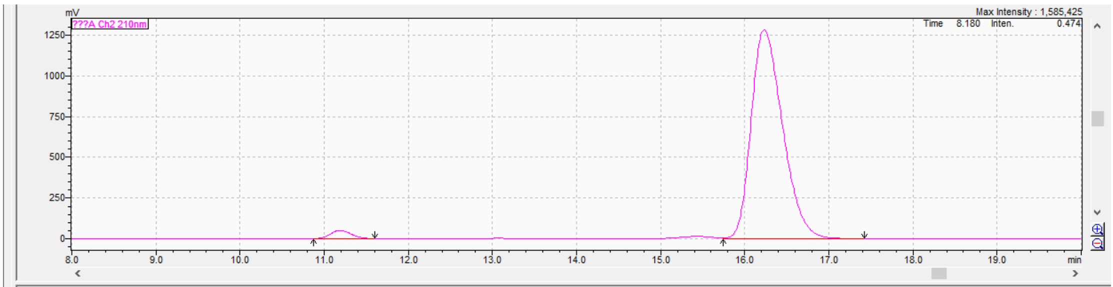

Results View - Peak Table

Peak Table | Compound | Group | Calibration Curve

Peak#	Ret. Time	Area	Height	Mark	Conc.	Unit	ID#	Name	Area%
1	11. 189	885774	50513	M	2. 593				2. 593
2	16. 230	33268530	1281309	M	97. 407				97. 407
Total		34154304	1331822		100. 000				100. 00

Supplementary Figure 297. HPLC spectrum of 3ca

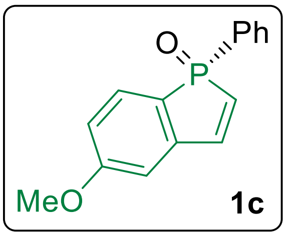

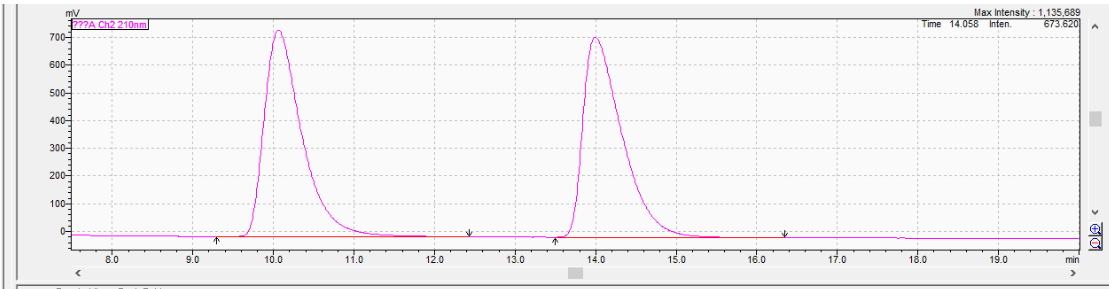

Peak#	Ret. Time	Area	Height	Mark	Conc.	Unit	ID#	Name	Area%
1	10.063	24274665	744458	M	49.976				49.976
2	13.996	24297741	721428	M	50.024				50.024
Total		48572406	1465886		100.000				100.00

Supplementary Figure 298. HPLC spectrum of racemic 1c

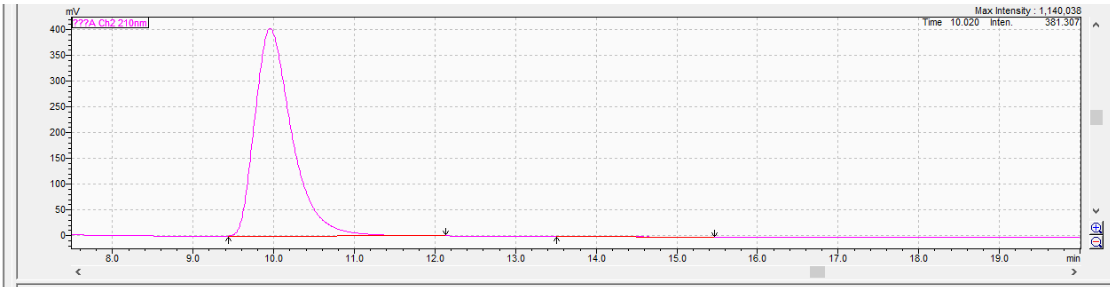

Peak#	Ret. Time	Area	Height	Mark	Conc.	Unit	ID#	Name	Area%
1	9.956	12831363	403343	M	99.887				99.887
2	13.619	14470	202	M	0.113				0.113
Total		12845832	403544		100.000				100.00

Supplementary Figure 299. HPLC spectrum of 1c

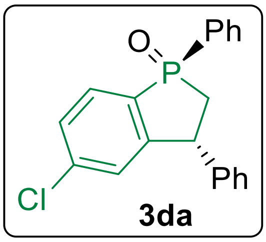

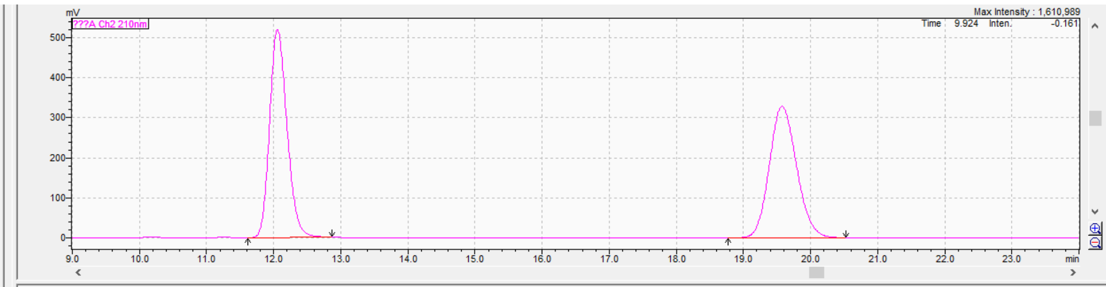

Peak#	Ret. Time	Area	Height	Mark	Conc.	Unit	ID#	Name	Area%
1	12. 056	9388173	520686	M	50. 258				50. 258
2	19. 577	9291654	329034	M	49. 742				49. 742
Total		18679827	849721		100. 000				100. 00

Supplementary Figure 300. HPLC spectrum of racemic 3da

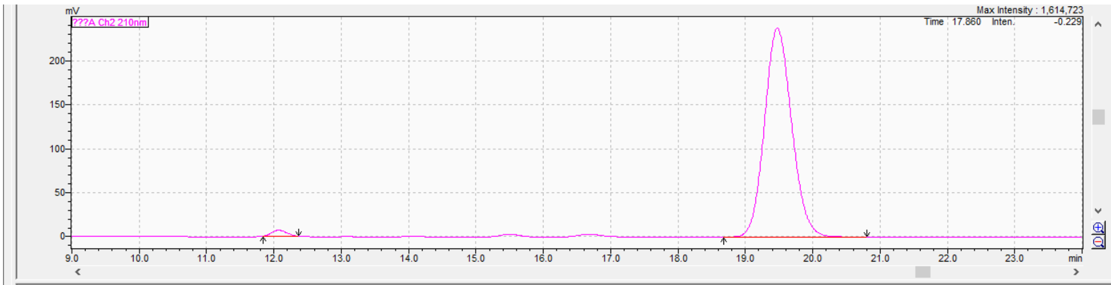

Peak#	Ret. Time	Area	Height	Mark	Conc.	Unit	ID#	Name	Area%
1	12. 073	104537	6741	M	1. 543				1. 543
2	19. 475	6670565	237903	M	98. 457				98. 457
Total		6775102	244645		100. 000				100. 00

Supplementary Figure 301. HPLC spectrum of 3da

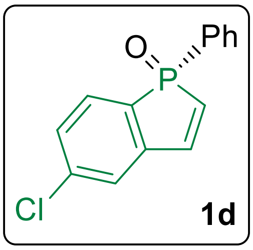

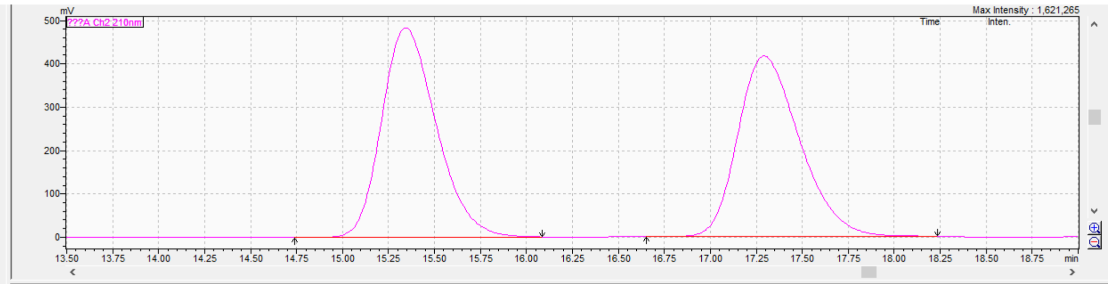

Results View - Peak Table

Peak Table | Compound | Group | Calibration Curve

Peak#	Ret. Time	Area	Height	Mark	Conc.	Unit	ID#	Name	Area%
1	15. 343	9974335	482909	M	50. 560				50. 560
2	17. 292	9753190	416572	M	49. 440				49. 440
Total		19727525	899481		100. 000				100. 00

Supplementary Figure 302. HPLC spectrum of racemic 1d

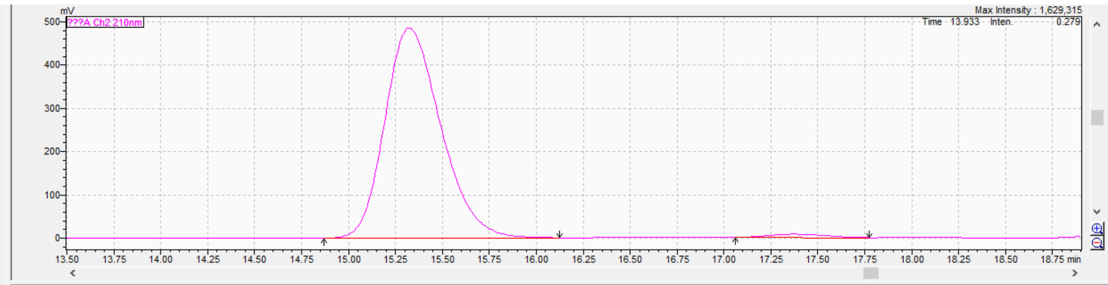

Results View - Peak Table

Peak Table | Compound | Group | Calibration Curve

Peak#	Ret. Time	Area	Height	Mark	Conc.	Unit	ID#	Name	Area%
1	15. 324	10045451	485294	M	98. 445				98. 445
2	17. 370	158670	7752	M	1. 555				1. 555
Total		10204120	493046		100. 000				100. 00

Supplementary Figure 303. HPLC spectrum of 1d

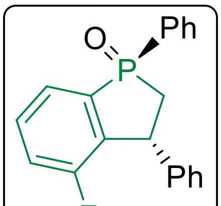

3ea

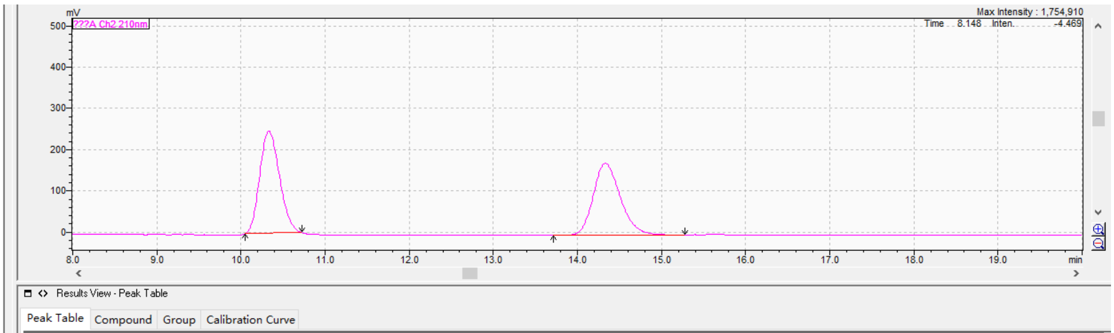

Peak#	Ret. Time	Area	Height	Mark	Conc.	Unit	ID#	Name	Area%
1	10. 331	4058204	248239	M	50. 915				50. 915
2	14. 332	3912335	174239	M	49. 085				49. 085
Total		7970539	422478		100. 000				100. 00

Supplementary Figure 304. HPLC spectrum of racemic 3ea

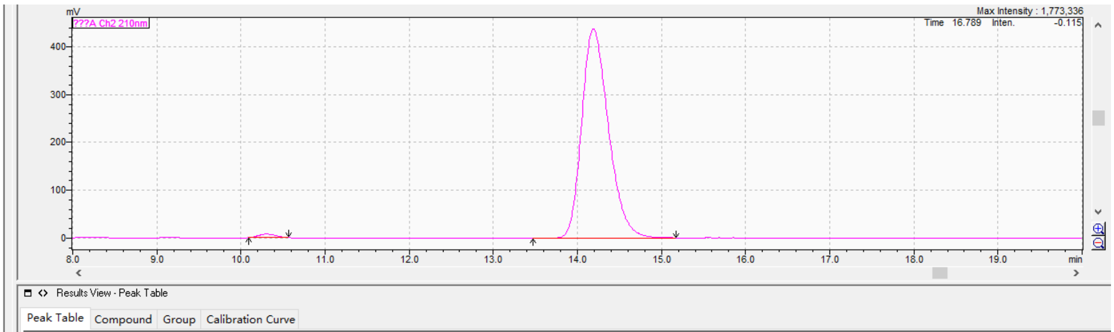

Peak#	Ret. Time	Area	Height	Mark	Conc.	Unit	ID#	Name	Area%
1	10. 306	107142	7323	M	1. 095				1. 095
2	14. 190	9677212	437713	M	98. 905				98. 905
Total		9784353	445036		100. 000				100. 00

Supplementary Figure 305. HPLC spectrum of 3ea

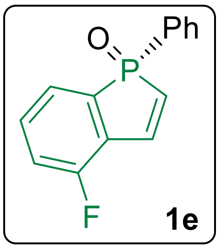

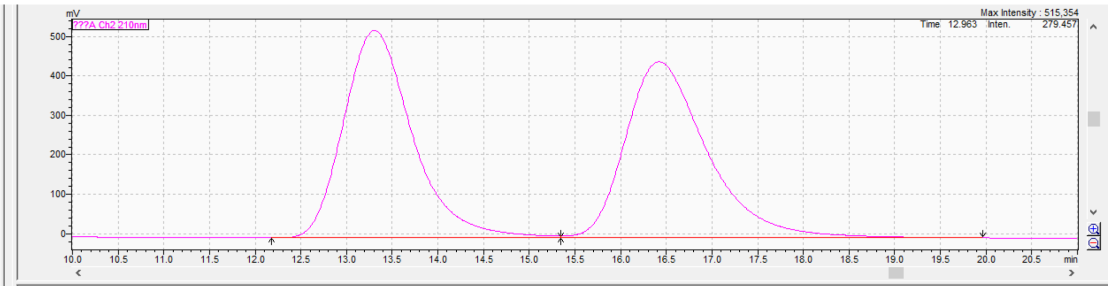

Results View - Peak Table

Peak Table | Compound | Group | Calibration Curve

Peak#	Ret. Time	Area	Height	Mark	Conc.	Unit	ID#	Name	Area%
1	13. 307	27579909	525858	V	49. 817				49. 817
2	16. 420	27782931	446252	SV	50. 183				50. 183
Total		55362841	972110		100. 000				100. 00

Supplementary Figure 306. HPLC spectrum of racemic 1e

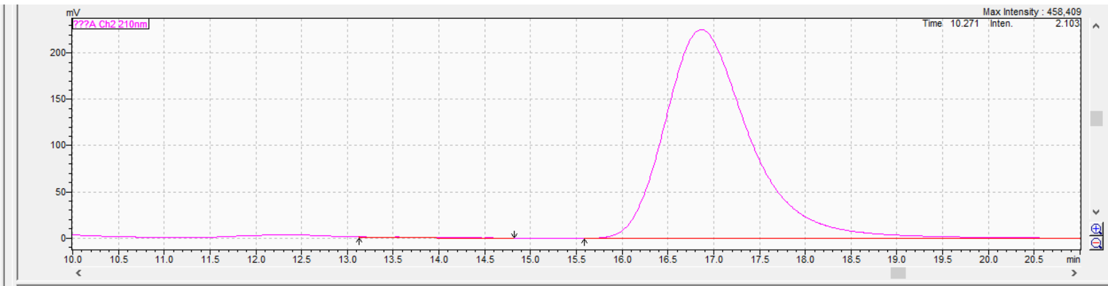

Results View - Peak Table

Peak Table | Compound | Group | Calibration Curve

Peak#	Ret. Time	Area	Height	Mark	Conc.	Unit	ID#	Name	Area%
1	13. 140	64195	1018	M	0. 441				0. 441
2	16. 867	14477350	226114	M	99. 559				99. 559
Total		14541545	227133		100. 000				100. 00

Supplementary Figure 307. HPLC spectrum of 1e

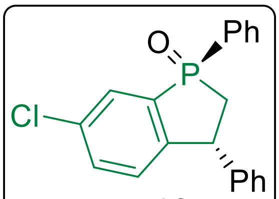

3fa

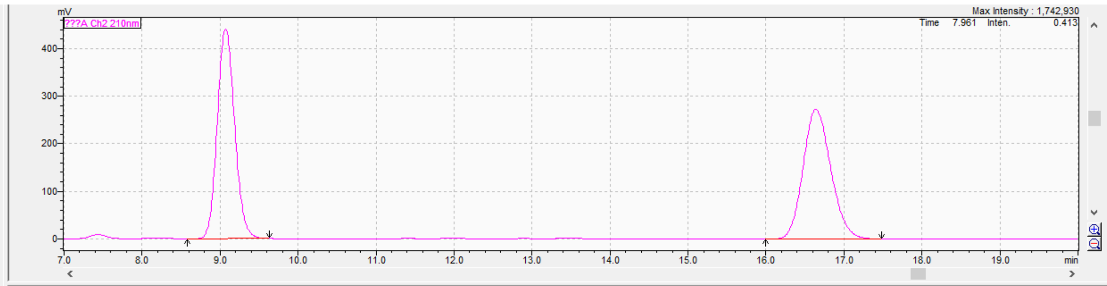

Results View - Peak Table

Peak Table | Compound | Group | Calibration Curve

Peak#	Ret. Time	Area	Height	Mark	Conc.	Unit	ID#	Name	Area%
1	9. 071	6828786	441423	M	50. 206				50. 206
2	16. 640	6772747	273132	M	49. 794				49. 794
Total		13601533	714555		100. 000				100. 00

Supplementary Figure 308. HPLC spectrum of racemic 3fa

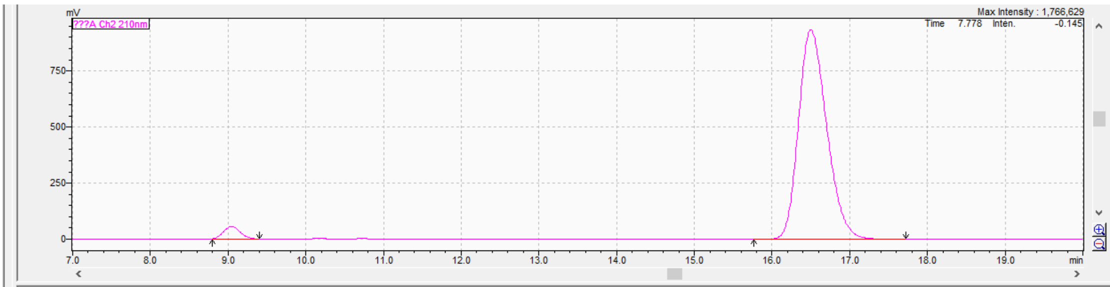

Results View - Peak Table

Peak Table | Compound | Group | Calibration Curve

Peak#	Ret. Time	Area	Height	Mark	Conc.	Unit	ID#	Name	Area%
1	9. 043	826813	55789	M	3. 394				3. 394
2	16. 499	23537276	934367	M	96. 606				96. 606
Total		24364090	990156		100. 000				100. 00

Supplementary Figure 309. HPLC spectrum of 3fa

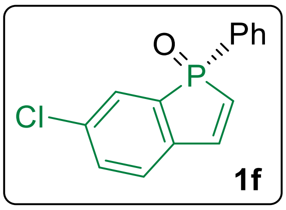

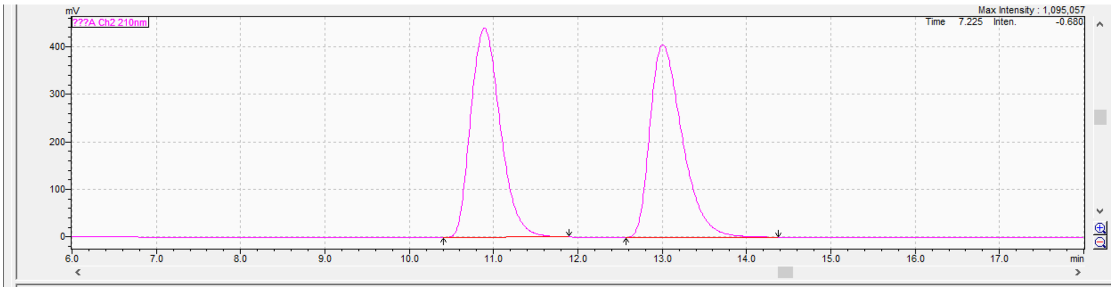

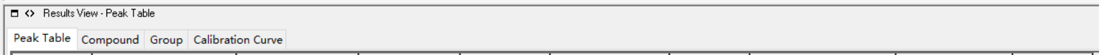

Peak#	Ret. Time	Area	Height	Mark	Conc.	Unit	ID#	Name	Area%
1	10.892	10761249	440537	M	49.982				49.982
2	13.006	10768975	404961	M	50.018				50.018
Total		21530224	845497		100.000				100.00

Supplementary Figure 310. HPLC spectrum of racemic 1f

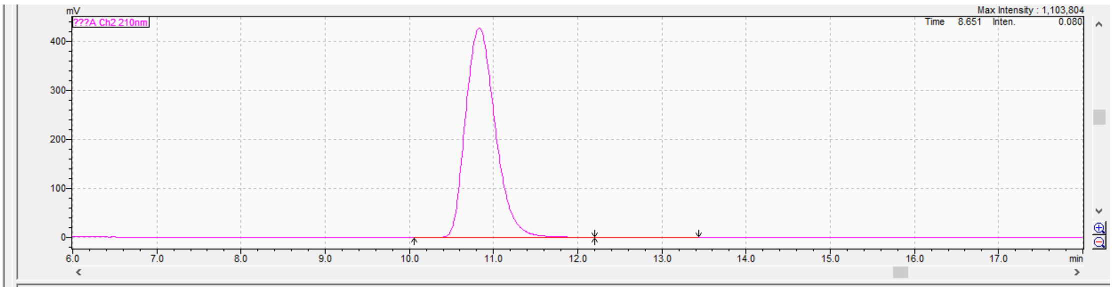

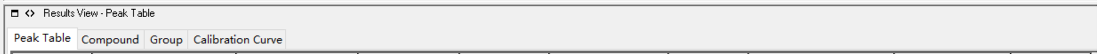

Peak#	Ret. Time	Area	Height	Mark	Conc.	Unit	ID#	Name	Area%
1	10.830	10467437	428326	M	99.995				99.995
2	13.156	495	91	V M	0.005				0.005
Total		10467933	428418		100.000				100.00

Supplementary Figure 311. HPLC spectrum of 1f

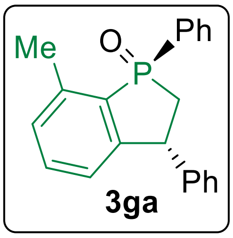

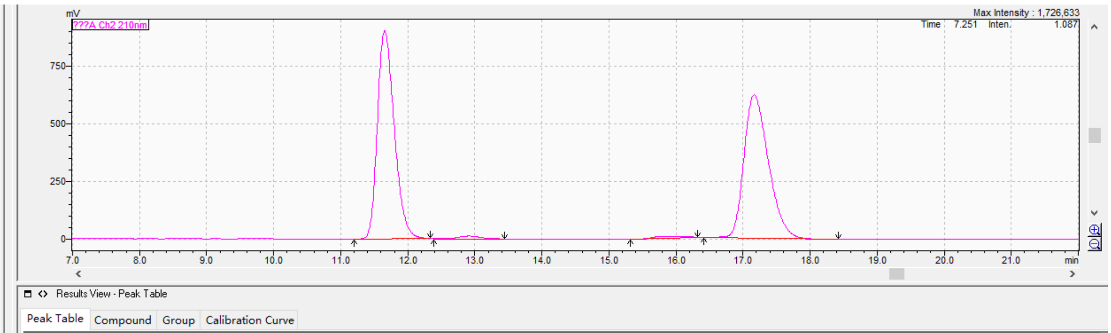

Peak#	Ret. Time	Area	Height	Mark	Conc.	Unit	ID#	Name	Area%
1	11. 654	15740505	902058	M	49. 054				49. 054
2	12. 910	278461	12049	M	0. 868				0. 868
3	16. 097	270102	6775	M	0. 842				0. 842
4	17. 165	15798888	621128	M	49. 236				49. 236
Total		32087955	1542010		100. 000				100. 00

Supplementary Figure 312. HPLC spectrum of racemic 3ga

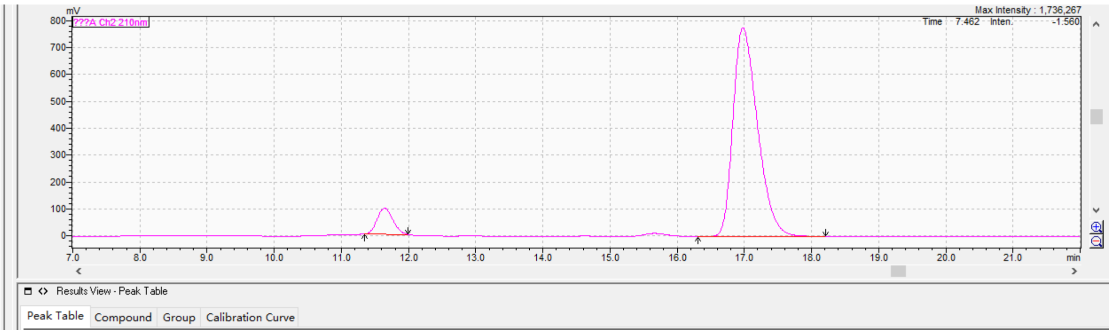

Peak#	Ret. Time	Area	Height	Mark	Conc.	Unit	ID#	Name	Area%
1	11. 643	1586054	96785	M	7. 465				7. 465
2	16. 980	19660345	776617	M	92. 535				92. 535
Total		21246399	873402		100. 000				100. 00

Supplementary Figure 313. HPLC spectrum of 3ga

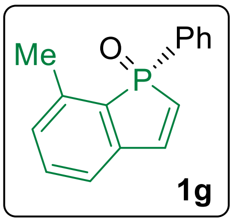

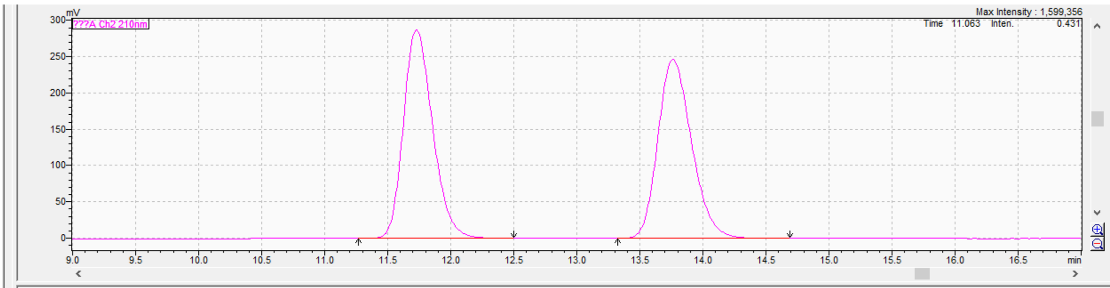

Results View - Peak Table

Peak Table | Compound | Group | Calibration Curve

Peak#	Ret. Time	Area	Height	Mark	Conc.	Unit	ID#	Name	Area%
1	11. 727	4604228	287195	M	50. 078				50. 078
2	13. 762	4589912	246969	M	49. 922				49. 922
Total		9194140	534164		100. 000				100. 00

Supplementary Figure 314. HPLC spectrum of racemic 1g

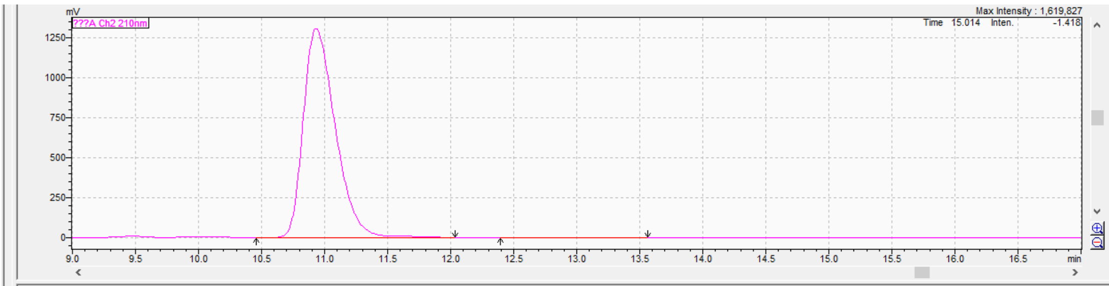

Results View - Peak Table

Peak Table | Compound | Group | Calibration Curve

Peak#	Ret. Time	Area	Height	Mark	Conc.	Unit	ID#	Name	Area%
1	10. 934	23077347	1305583	M	99. 946				99. 946
2	12. 656	12519	671	M	0. 054				0. 054
Total		23089866	1306253		100. 000				100. 00

Supplementary Figure 315. HPLC spectrum of 1g

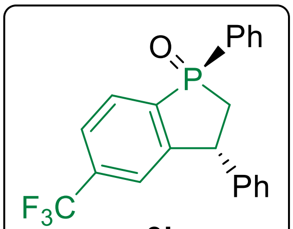

3ha

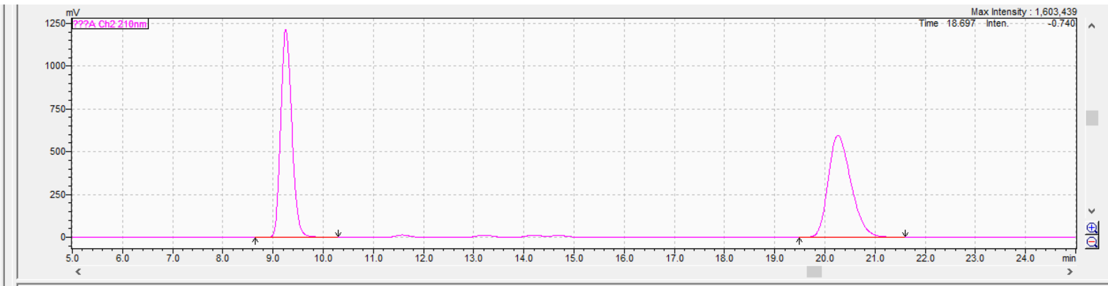

Results View - Peak Table

Peak Table | Compound | Group | Calibration Curve

Peak#	Ret. Time	Area	Height	Mark	Conc.	Unit	ID#	Name	Area%
1	9.254	18653416	1214264	M	49.769				49.769
2	20.260	18826705	595516	M	50.231				50.231
Total		37480121	1809780		100.000				100.00

Supplementary Figure 316. HPLC spectrum of racemic 3ha

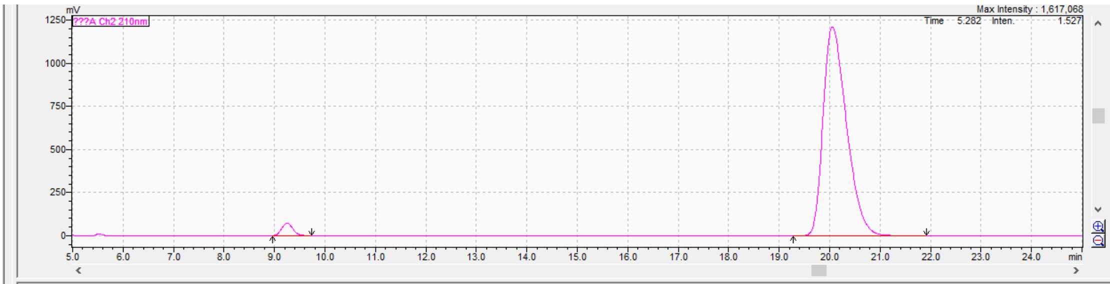

Results View - Peak Table

Peak Table | Compound | Group | Calibration Curve

Peak#	Ret. Time	Area	Height	Mark	Conc.	Unit	ID#	Name	Area%
1	9.246	1111635	73039	M	2.762				2.762
2	20.054	39135753	1212209	M	97.238				97.238
Total		40247387	1285248		100.000				100.00

Supplementary Figure 317. HPLC spectrum of 3ha

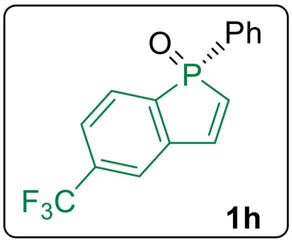

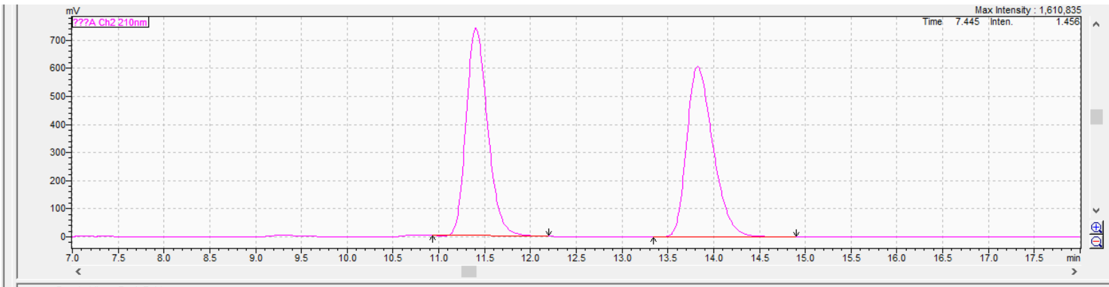

Results View - Peak Table

Peak Table | Compound | Group | Calibration Curve

Peak#	Ret. Time	Area	Height	Mark	Conc.	Unit	ID#	Name	Area%
1	11. 403	12076102	740553	M	49. 915				49. 915
2	13. 825	12117260	605803	M	50. 085				50. 085
Total		24193363	1346356		100. 000				100. 00

Supplementary Figure 318. HPLC spectrum of racemic 1h

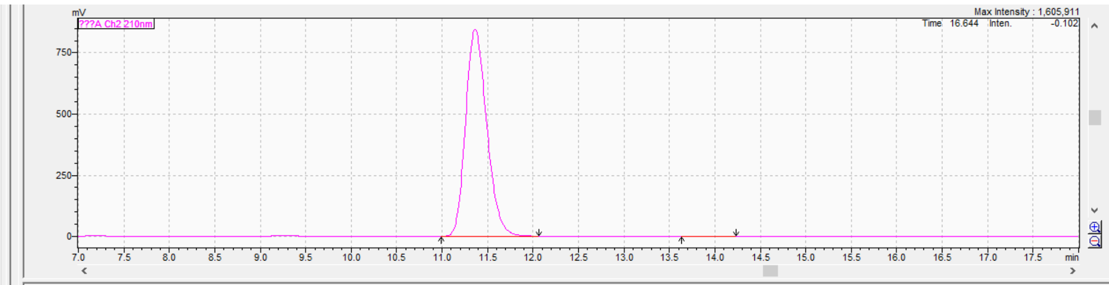

Results View - Peak Table

Peak Table | Compound | Group | Calibration Curve

Peak#	Ret. Time	Area	Height	Mark	Conc.	Unit	ID#	Name	Area%
1	11. 362	13706700	845151	M	99. 795				99. 795
2	13. 899	28174	1618	M	0. 205				0. 205
Total		13734875	846769		100. 000				100. 00

Supplementary Figure 319. HPLC spectrum of 1h

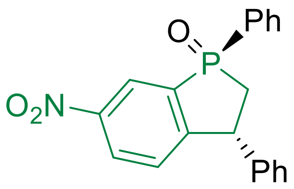

3ia

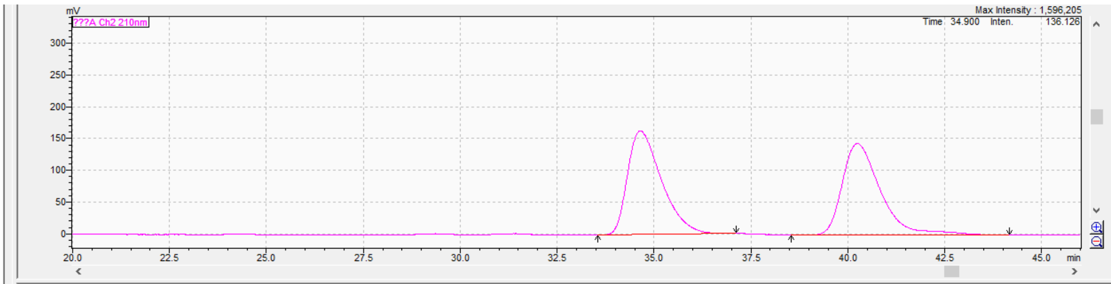

Results View - Peak Table

Peak Table Compound Group Calibration Curve

Peak#	Ret. Time	Area	Height	Mark	Conc.	Unit	ID#	Name	Area%
1	34. 650	9951650	163223	M	50. 167				50. 167
2	40. 251	9885570	143978	M	49. 833				49. 833
Total		19837221	307201		100. 000				100. 00

Supplementary Figure 320. HPLC spectrum of racemic 3ia

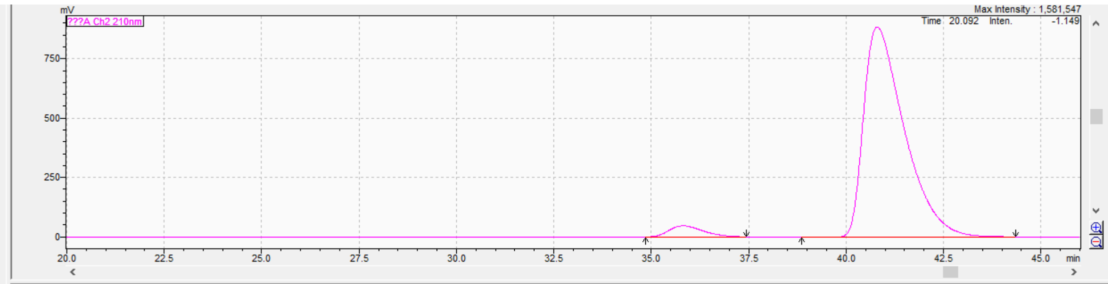

Results View - Peak Table

Peak Table Compound Group Calibration Curve

Peak#	Ret. Time	Area	Height	Mark	Conc.	Unit	ID#	Name	Area%
1	35. 812	2889629	46856	M	4. 288				4. 288
2	40. 800	64502006	885522	M	95. 712				95. 712
Total		67391635	932378		100. 000				100. 00

Supplementary Figure 321. HPLC spectrum of 3ia

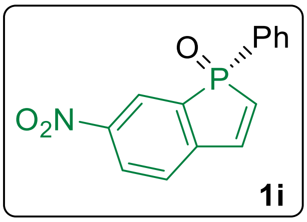

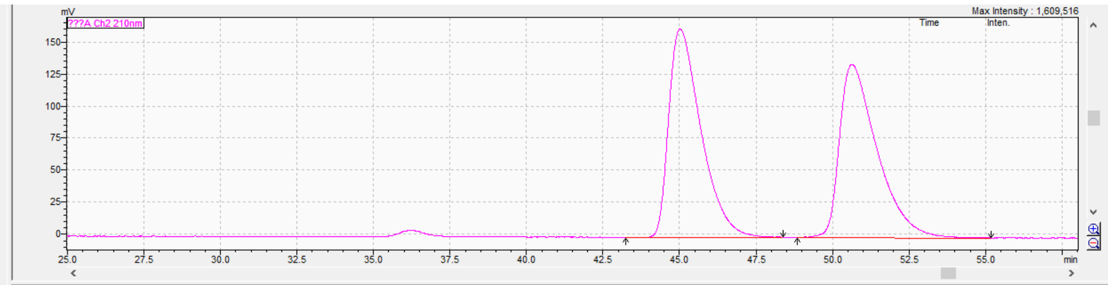

Results View - Peak Table

Peak Table | Compound | Group | Calibration Curve

Peak#	Ret. Time	Area	Height	Mark	Conc.	Unit	ID#	Name	Area%
1	45.020	11903575	162864	M	50.791				50.791
2	50.632	11532992	135140	M	49.209				49.209
Total		23436567	298004		100.000				100.00

Supplementary Figure 322. HPLC spectrum of racemic 1i

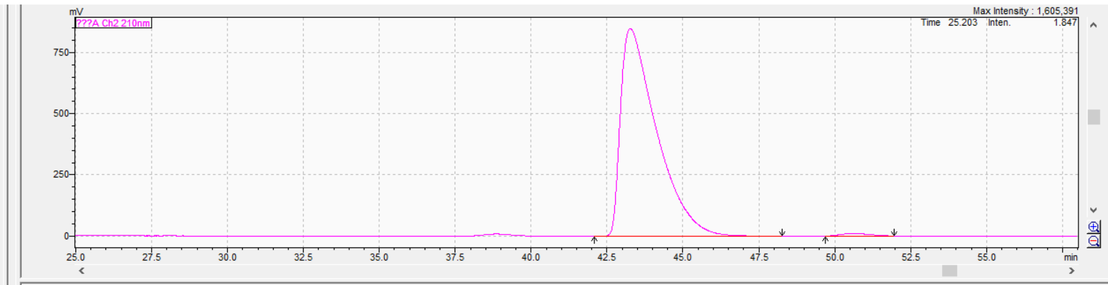

Results View - Peak Table

Peak Table | Compound | Group | Calibration Curve

Peak#	Ret. Time	Area	Height	Mark	Conc.	Unit	ID#	Name	Area%
1	43.267	69963734	849005	M	99.019				99.019
2	50.612	693003	10632	M	0.981				0.981
Total		70656736	859636		100.000				100.00

Supplementary Figure 323. HPLC spectrum of 1i

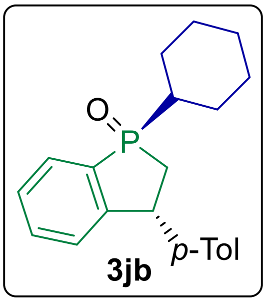

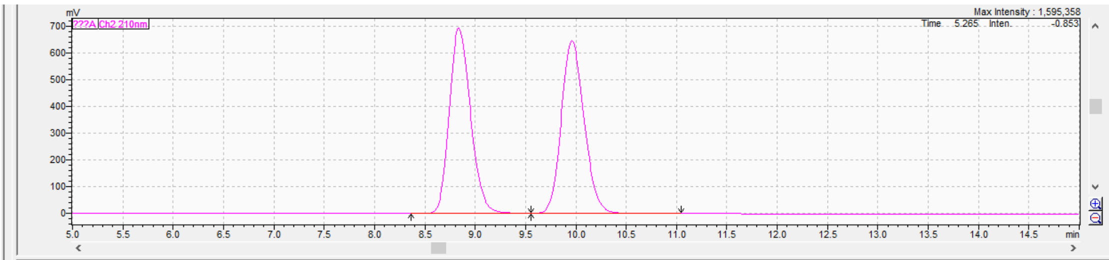

Results View - Peak Table

Peak Table | Compound | Group | Calibration Curve

Peak#	Ret. Time	Area	Height	Mark	Conc.	Unit	ID#	Name	Area%
1	8.838	10257979	694853	M	49.972				49.972
2	9.960	10269352	648489	V M	50.028				50.028
Total		20527331	1343342		100.000				100.00

Supplementary Figure 324. HPLC spectrum of racemic 3jb

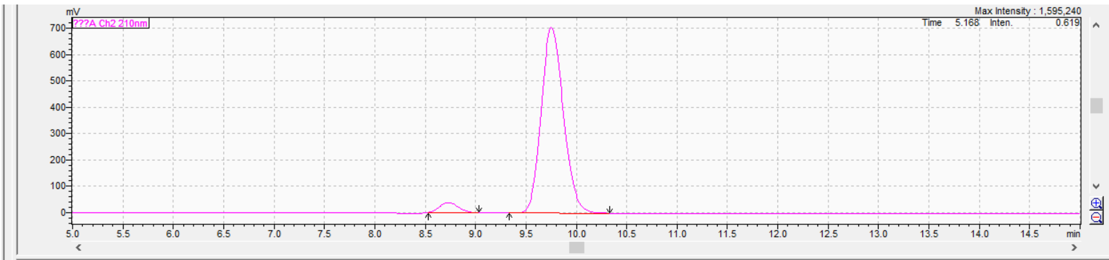

Results View - Peak Table

Peak Table | Compound | Group | Calibration Curve

Peak#	Ret. Time	Area	Height	Mark	Conc.	Unit	ID#	Name	Area%
1	8.731	484461	37264	M	4.296				4.296
2	9.753	10791651	703711	M	95.704				95.704
Total		11276112	740975		100.000				100.00

Supplementary Figure 325. HPLC spectrum of 3jb

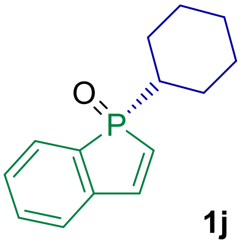

1j

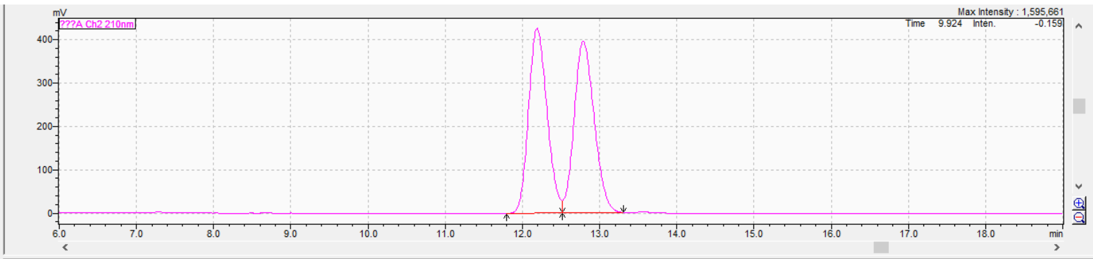

Results View - Peak Table

Peak Table | Compound | Group | Calibration Curve

Peak#	Ret. Time	Area	Height	Mark	Conc.	Unit	ID#	Name	Area%
1	12.189	7097210	426286	M	49.866				49.866
2	12.787	7135440	396349	V M	50.134				50.134
Total		14232650	822634		100.000				100.00

Supplementary Figure 326. HPLC spectrum of racemic 1j

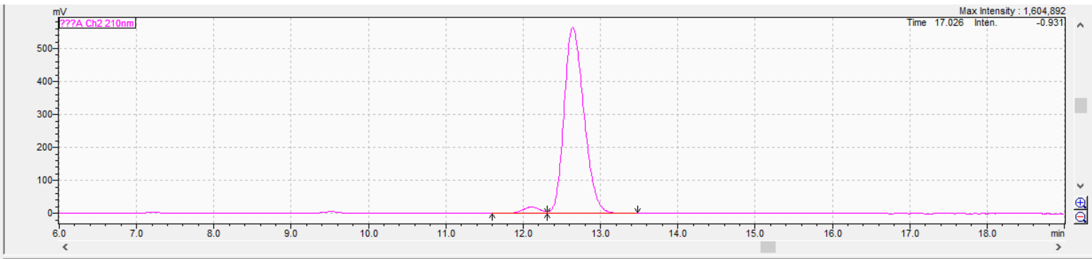

Results View - Peak Table

Peak Table | Compound | Group | Calibration Curve

Peak#	Ret. Time	Area	Height	Mark	Conc.	Unit	ID#	Name	Area%
1	12.107	298679	19093	M	2.888				2.888
2	12.642	10042411	563667	V M	97.112				97.112
Total		10341090	582760		100.000				100.00

Supplementary Figure 327. HPLC spectrum of 1j

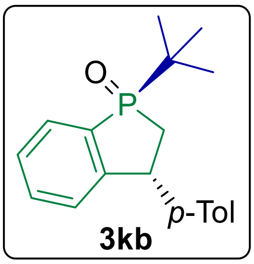

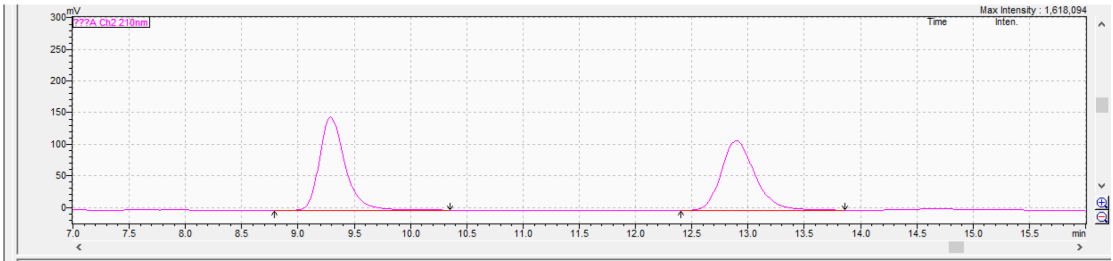

Results View - Peak Table

Peak Table | Compound | Group | Calibration Curve

Peak#	Ret. Time	Area	Height	Mark	Conc.	Unit	ID#	Name	Area%
1	9. 290	2317427	147205	M	50. 213				50. 213
2	12. 896	2297721	110124	M	49. 787				49. 787
Total		4615148	257329		100. 000				100. 00

Supplementary Figure 328. HPLC spectrum of racemic 3kb

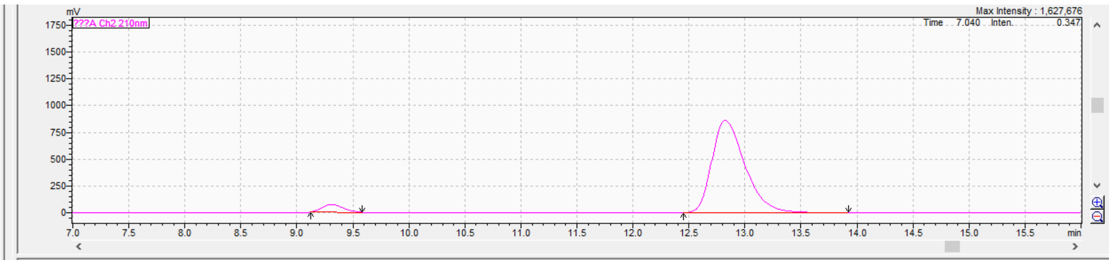

Results View - Peak Table

Peak Table | Compound | Group | Calibration Curve

Peak#	Ret. Time	Area	Height	Mark	Conc.	Unit	ID#	Name	Area%
1	9. 307	956459	70233	M	5. 097				5. 097
2	12. 825	17807628	862401	M	94. 903				94. 903
Total		18764086	932634		100. 000				100. 00

Supplementary Figure 329. HPLC spectrum of 3kb

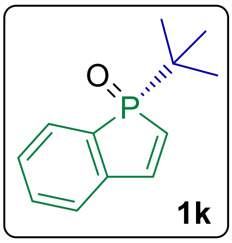

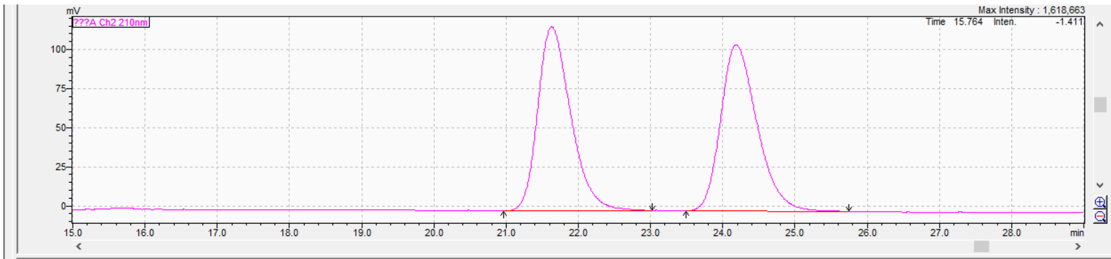

Results View - Peak Table

Peak Table | Compound | Group | Calibration Curve

Peak#	Ret. Time	Area	Height	Mark	Conc.	Unit	ID#	Name	Area%
1	21. 632	3728530	117587	M	50. 058				50. 058
2	24. 185	3719821	106104	M	49. 942				49. 942
Total		7448351	223691		100. 000				100. 00

Supplementary Figure 330. HPLC spectrum of racemic 1k

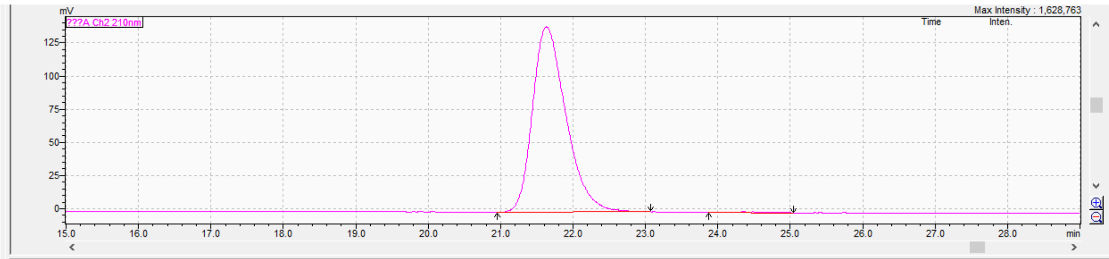

Results View - Peak Table

Peak Table | Compound | Group | Calibration Curve

Peak#	Ret. Time	Area	Height	Mark	Conc.	Unit	ID#	Name	Area%
1	21. 633	4472259	139516	M	99. 684				99. 684
2	24. 348	14178	554	M	0. 316				0. 316
Total		4486437	140070		100. 000				100. 00

Supplementary Figure 331. HPLC spectrum of 1k

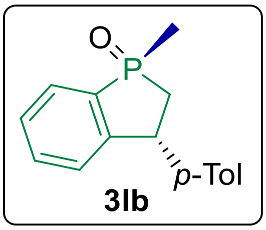

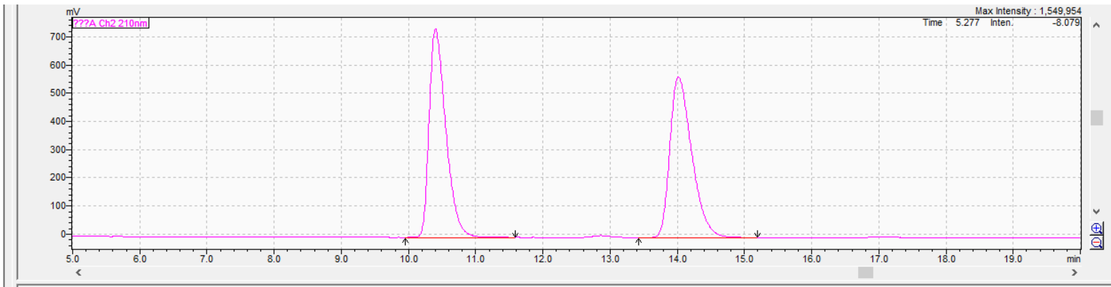

Results View - Peak Table

Peak Table | Compound | Group | Calibration Curve

Peak#	Ret. Time	Area	Height	Mark	Conc.	Unit	ID#	Name	Area%
1	10. 402	12730224	740734	M	49. 607				49. 607
2	14. 018	12931688	570808	M	50. 393				50. 393
Total		25661913	1311542		100. 000				100. 000

Supplementary Figure 332. HPLC spectrum of racemic 3lb

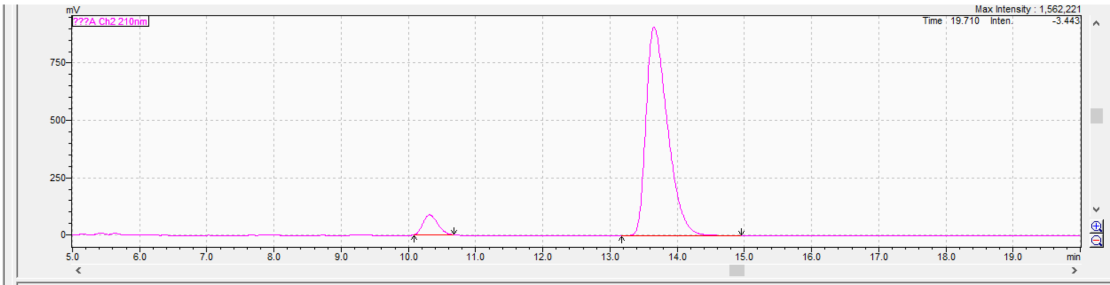

Results View - Peak Table

Peak Table | Compound | Group | Calibration Curve

Peak#	Ret. Time	Area	Height	Mark	Conc.	Unit	ID#	Name	Area%
1	10. 316	1321731	87032	M	6. 146				6. 146
2	13. 657	20182792	909528	M	93. 854				93. 854
Total		21504523	996560		100. 000				100. 000

Supplementary Figure 333. HPLC spectrum of 3lb

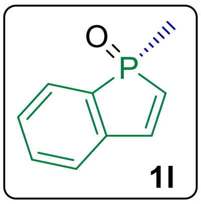

1l

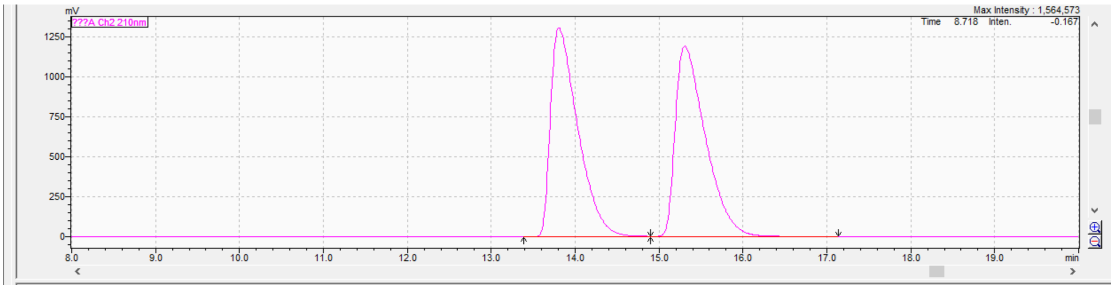

Results View - Peak Table

Peak Table | Compound | Group | Calibration Curve

Peak#	Ret. Time	Area	Height	Mark	Conc.	Unit	ID#	Name	Area%
1	13.808	29645291	1309198	M	49.798				49.798
2	15.308	29886151	1194647	V M	50.202				50.202
Total		59531442	2503845		100.000				100.000

Supplementary Figure 334. HPLC spectrum of racemic 1l

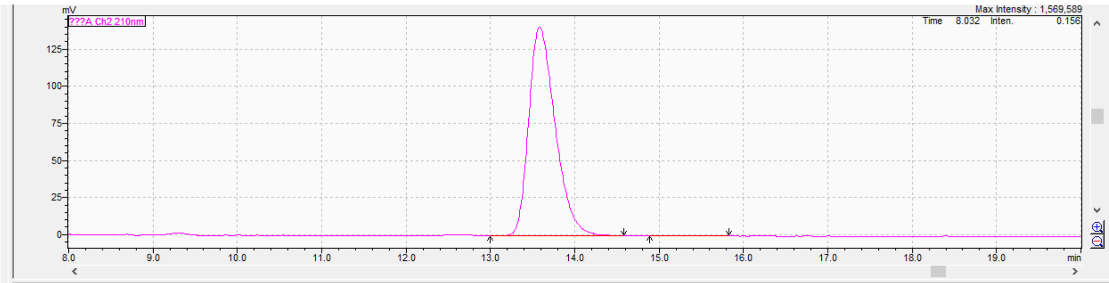

Results View - Peak Table

Peak Table | Compound | Group | Calibration Curve

Peak#	Ret. Time	Area	Height	Mark	Conc.	Unit	ID#	Name	Area%
1	13.583	3065773	140557	M	99.988				99.988
2	15.115	366	187	M	0.012				0.012
Total		3066138	140745		100.000				100.000

Supplementary Figure 335. HPLC spectrum of 1l

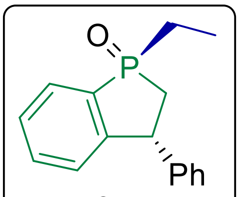

3ma

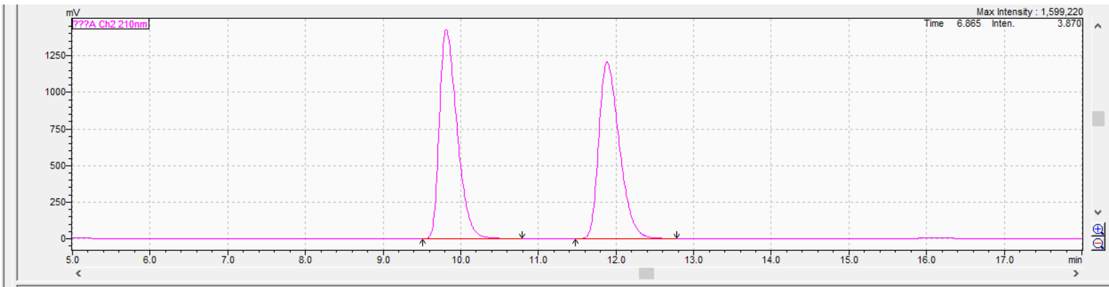

Results View - Peak Table

Peak Table | Compound | Group | Calibration Curve

Peak#	Ret. Time	Area	Height	Mark	Conc.	Unit	ID#	Name	Area%
1	9.812	22581696	1426690	M	50.472				50.472
2	11.886	22159648	1205588	M	49.528				49.528
Total		44741344	2632278		100.000				100.00

Supplementary Figure 336. HPLC spectrum of racemic 3ma

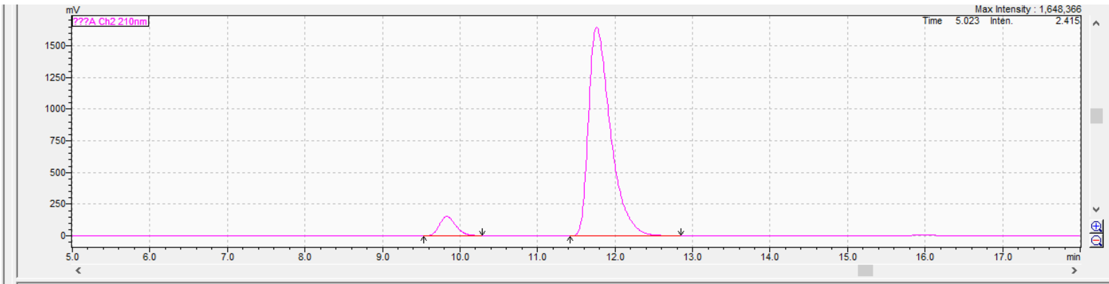

Results View - Peak Table

Peak Table | Compound | Group | Calibration Curve

Peak#	Ret. Time	Area	Height	Mark	Conc.	Unit	ID#	Name	Area%
1	9.831	2241717	152192	M	6.473				6.473
2	11.764	32388579	1647750	M	93.527				93.527
Total		34630297	1799942		100.000				100.00

Supplementary Figure 337. HPLC spectrum of 3ma

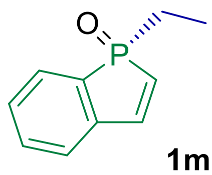

1m

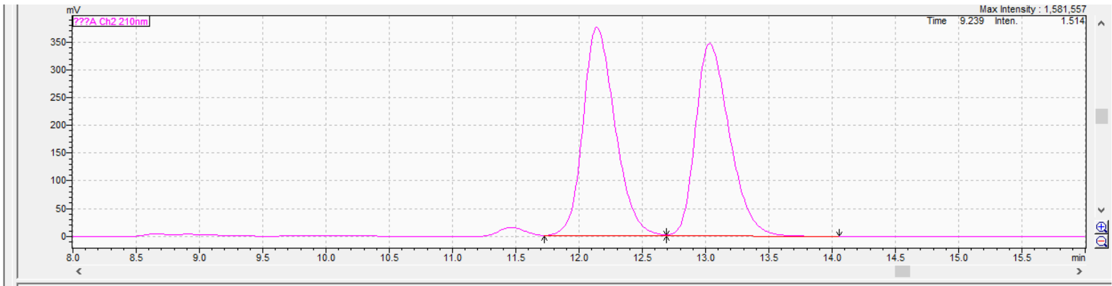

Results View - Peak Table

Peak Table | Compound | Group | Calibration Curve

Peak#	Ret. Time	Area	Height	Mark	Conc.	Unit	ID#	Name	Area%
1	12.141	6611993	376171	M	50.496				50.496
2	13.033	6482116	347148	V M	49.504				49.504
Total		13094109	723320		100.000				100.00

Supplementary Figure 338. HPLC spectrum of racemic 1m

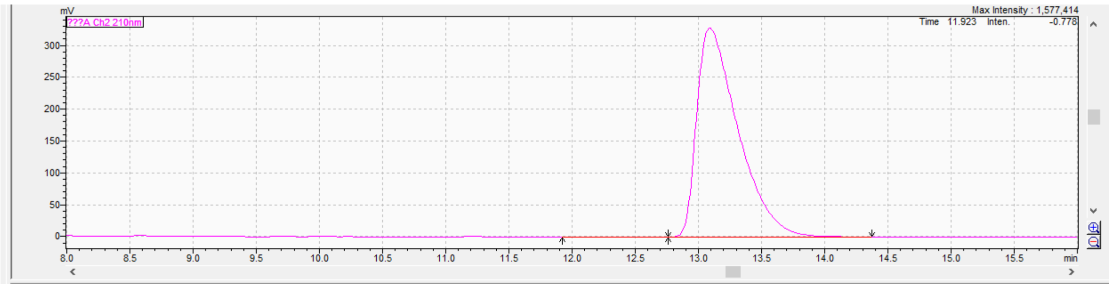

Results View - Peak Table

Peak Table | Compound | Group | Calibration Curve

Peak#	Ret. Time	Area	Height	Mark	Conc.	Unit	ID#	Name	Area%
1	12.118	5165	242	M	0.069				0.069
2	13.091	7447248	328269	M	99.931				99.931
Total		7452413	328511		100.000				100.00

Supplementary Figure 339. HPLC spectrum of 1m

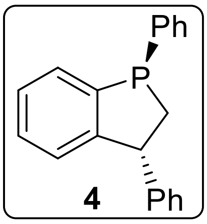

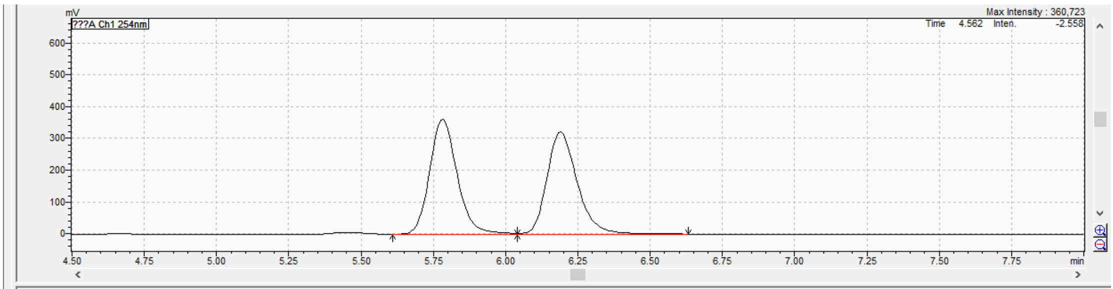

Results View - Peak Table

Peak Table | Compound | Group | Calibration Curve

Peak#	Ret. Time	Area	Height	Mark	Conc.	Unit	ID#	Name	Area%
1	5.781	2395745	362161	M	50.645				50.645
2	6.189	2334734	321613	V M	49.355				49.355
Total		4730480	683774		100.000				100.00

Supplementary Figure 340. HPLC spectrum of racemic 4

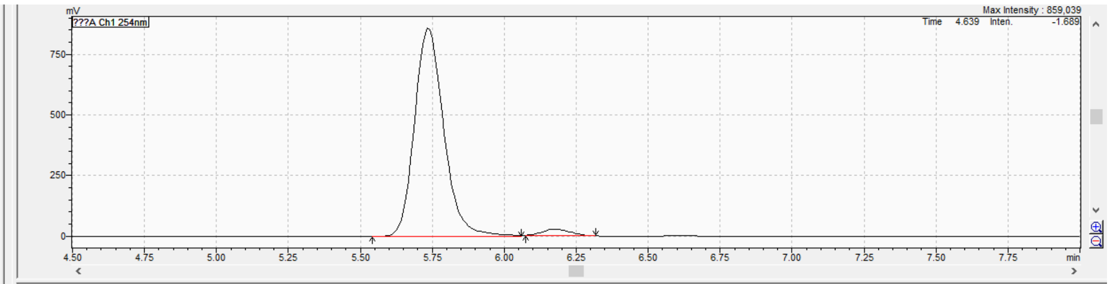

Results View - Peak Table

Peak Table | Compound | Group | Calibration Curve

Peak#	Ret. Time	Area	Height	Mark	Conc.	Unit	ID#	Name	Area%
1	5.735	6079451	860007	M	97.201				97.201
2	6.177	175067	26459	M	2.799				2.799
Total		6254518	886466		100.000				100.00

Supplementary Figure 341. HPLC spectrum of 4

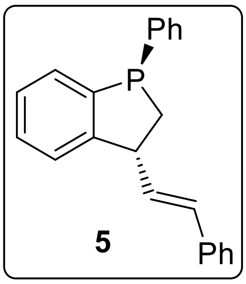

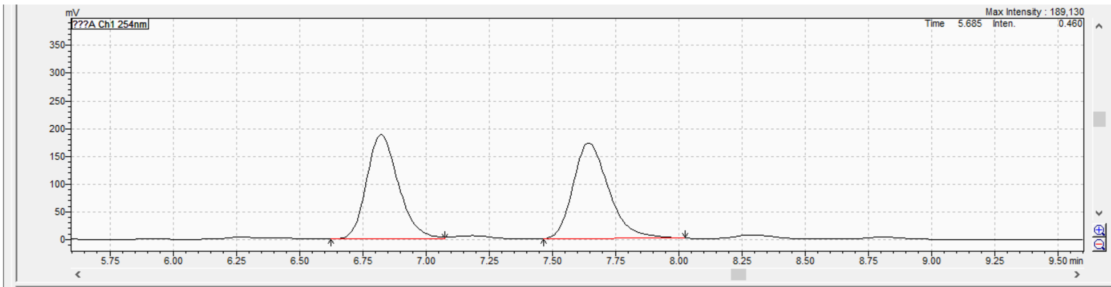

Results View - Peak Table

Peak Table | Compound | Group | Calibration Curve

Peak#	Ret. Time	Area	Height	Mark	Conc.	Unit	ID#	Name	Area%
1	6.823	1659355	188117	M	49.556				49.556
2	7.645	1689113	172406	M	50.444				50.444
Total		3348468	360523		100.000				100.00

Supplementary Figure 342. HPLC spectrum of racemic 5

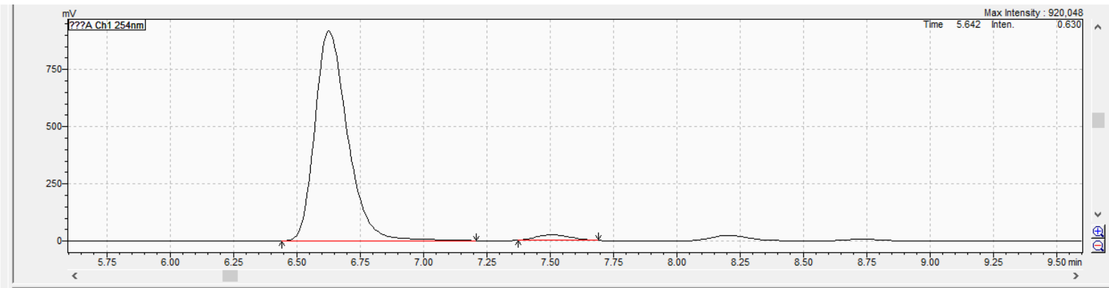

Results View - Peak Table

Peak Table | Compound | Group | Calibration Curve

Peak#	Ret. Time	Area	Height	Mark	Conc.	Unit	ID#	Name	Area%
1	6.627	8195819	918699	M	97.405				97.405
2	7.509	218335	23577	M	2.595				2.595
Total		8414155	942275		100.000				100.00

Supplementary Figure 343. HPLC spectrum of 5

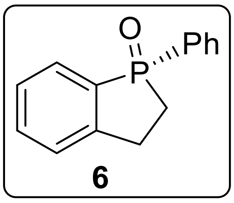

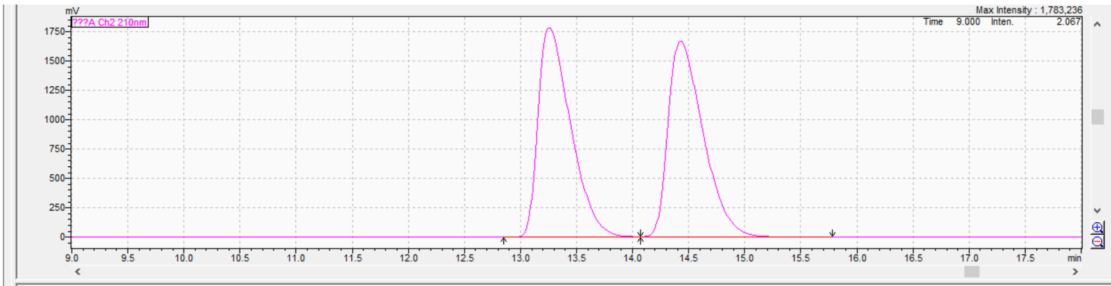

Results View - Peak Table

Peak Table | Compound | Group | Calibration Curve

Peak#	Ret. Time	Area	Height	Mark	Conc.	Unit	ID#	Name	Area%
1	13. 257	36078822	1782034	M	49. 857				49. 857
2	14. 429	36286217	1669514	V M	50. 143				50. 143
Total		72365039	3451548		100. 000				100. 00

Supplementary Figure 344. HPLC spectrum of racemic 6

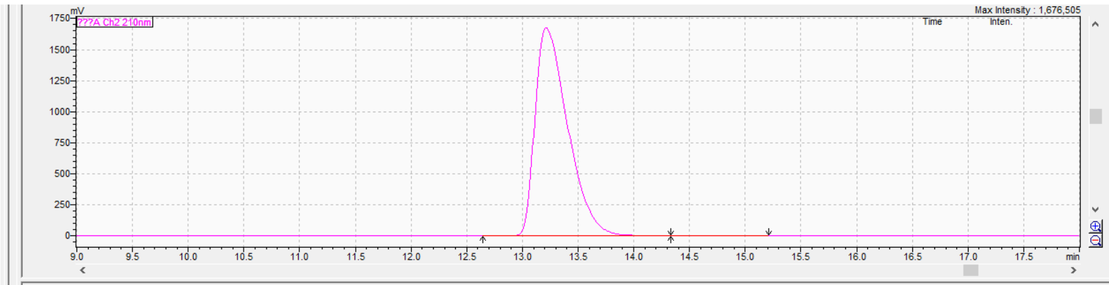

Results View - Peak Table

Peak Table | Compound | Group | Calibration Curve

Peak#	Ret. Time	Area	Height	Mark	Conc.	Unit	ID#	Name	Area%
1	13. 216	34138772	1676565	M	99. 937				99. 937
2	14. 577	21554	1253	V M	0. 063				0. 063
Total		34160326	1677818		100. 000				100. 00

Supplementary Figure 345. HPLC spectrum of 6

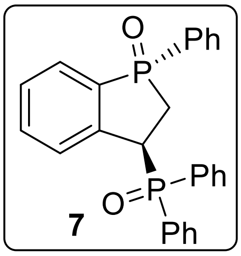

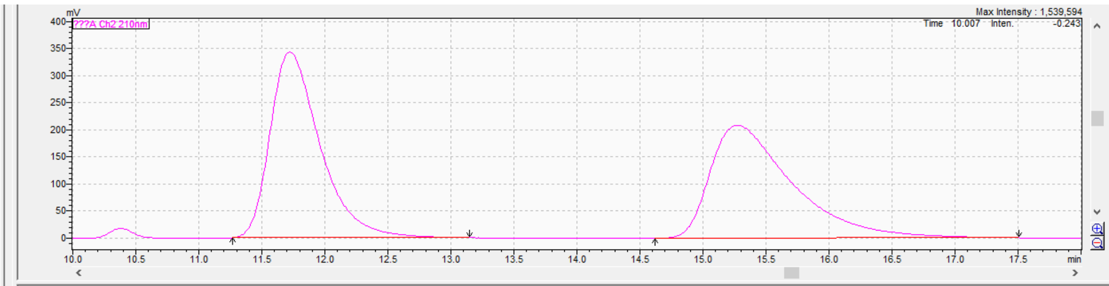

Results View - Peak Table

Peak Table | Compound | Group | Calibration Curve

Peak#	Ret. Time	Area	Height	Mark	Conc.	Unit	ID#	Name	Area%
1	11.723	9464514	342291	M	50.105				50.105
2	15.271	9424874	208223	M	49.895				49.895
Total		18889388	550514		100.000				100.00

Supplementary Figure 346. HPLC spectrum of racemic 7

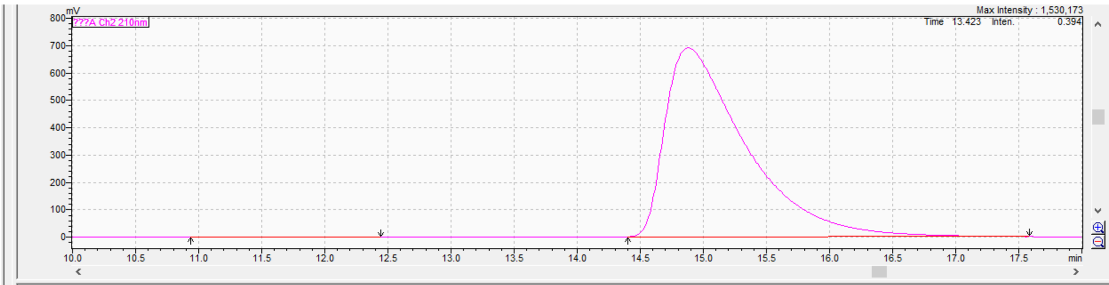

Results View - Peak Table

Peak Table | Compound | Group | Calibration Curve

Peak#	Ret. Time	Area	Height	Mark	Conc.	Unit	ID#	Name	Area%
1	11.281	56742	1477	M	0.185				0.185
2	14.880	30668493	689966	M	99.815				99.815
Total		30725235	691443		100.000				100.00

Supplementary Figure 347. HPLC spectrum of 7

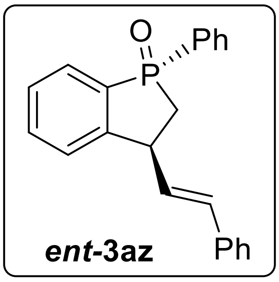

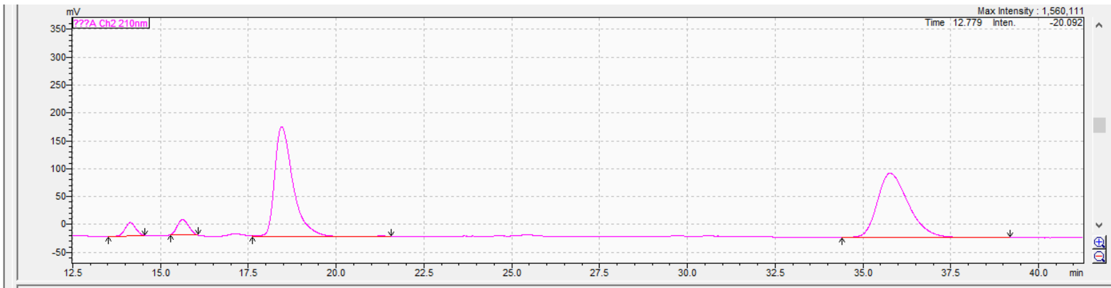

Results View - Peak Table

Peak Table | Compound | Group | Calibration Curve

Peak#	Ret. Time	Area	Height	Mark	Conc.	Unit	ID#	Name	Area%
1	14. 137	498281	23767	M	3. 267				3. 267
2	15. 630	607200	27032	M	3. 982				3. 982
3	18. 449	7058983	195990	M	46. 289				46. 289
4	35. 773	7085244	115249	M	46. 462				46. 462
Total		15249708	362038		100. 000				100. 00

Supplementary Figure 348. HPLC spectrum of racemic *ent*-3az

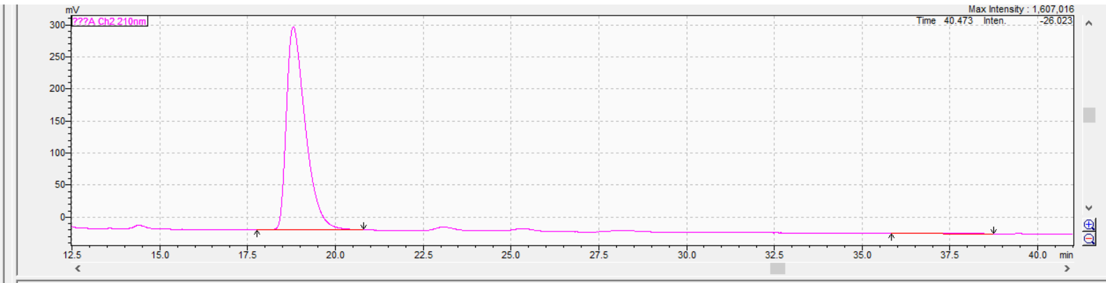

Results View - Peak Table

Peak Table | Compound | Group | Calibration Curve

Peak#	Ret. Time	Area	Height	Mark	Conc.	Unit	ID#	Name	Area%
1	18. 804	11844491	316626	M	99. 961				99. 961
2	35. 872	4619	76	M	0. 039				0. 039
Total		11849111	316702		100. 000				100. 000

Supplementary Figure 349. HPLC spectrum of *ent*-3az

4. Supplementary References

1. Ye, B. et al. Rhodium-Catalyzed Asymmetric Conjugate Pyridylation with Pyridylboronic Acids. *ACS Catal.* **12**, 2434–2440 (2022).

2. Wu, C. et al. Catalytic asymmetric indolization by a desymmetrizing [3+2] annulation strategy. *Chem. Sci.* **14**, 7980–7987 (2023).

3. Yasukawa, T., Suzuki, A., Miyamura, H., Nishino, K. & Kobayashi, S. Chiral metal nanoparticle systems as heterogeneous catalysts beyond homogeneous metal complex catalysts for asymmetric addition of arylboronic acids to *α*, *β*-unsaturated carbonyl compounds. *J. Am. Chem. Soc.* **137**, 6616–6623 (2015).

4. Rit, R. K. et al. A Scalable Synthesis of Chiral Himbert Diene Ligands for Asymmetric Catalysis. *Adv. Synth. Catal.* **365**, 1629–1639 (2023).

5. Tokunaga, N. et al. C2-Symmetric Bicyclo [2.2.2] octadienes as Chiral Ligands: Their High Performance in Rhodium-Catalyzed Asymmetric Arylation of N-Tosylarylimines. *J. Am. Chem. Soc.* **126**, 13584–13585 (2004).

6. Carr, D. J., Kudavalli, J. S., Dunne, K. S., Müller-Bunz, H. & Gilheany, D. G. Synthesis of 2, 3-dihydro-1-phenylbenzo [b] phosphole (1-phenylphosphindane) and its use as a mechanistic test in the asymmetric Appel reaction: decisive evidence against involvement of pseudorotation in the stereoselecting step. *J. Org. Chem.* **78**, 10500–10505 (2013).

7. Ponikiewski, Ł. & Sowa, S. Ring opening of triflates derived from benzophospholan-3-one oxides by aryl Grignard reagents as a route to 2-ethynylphenyl (diaryl) phosphine oxides. *J. Org. Chem.* **86**, 14928–14941 (2021).

8. Neese, F. The ORCA program system. *WIREs Comput. Mol. Sci.* **2**, 73–78 (2021).

9. Neese, F. Software update: the ORCA program system, version 4.0. *WIREs Comput. Mol. Sci.* **8**, e1327 (2018).

10. Neese, F., Wennmohs, F., Becker, U. & Riplinger, C. The ORCA quantum chemistry program package. *J. Chem. Phys.* **152**, 224108 (2020).

11. Neese, F. Software update: The ORCA program system—Version 5.0. *WIREs Comput. Mol. Sci.* **12**, e1606 (2022).

12. Bannwarth, C. et al. Extended tight-binding quantum chemistry methods. *WIREs Comput. Mol. Sci.* **11**, e01493 (2020).

13. Grimme, S., Bannwarth, C. & Shushkov, P. A robust and accurate tight-binding quantum chemical method for structures, vibrational frequencies, and noncovalent interactions of large molecular systems parametrized for all spd-block elements (Z = 1–86). *J. Chem. Theory Comput.* **13**, 1989–2009 (2017).

14. Bannwarth, C., Ehlert, S. & Grimme, S. GFN2-xTB—An accurate and broadly parametrized self-consistent tight-binding quantum chemical method with multipole electrostatics and density-dependent dispersion contributions. *J. Chem. Theory Comput*. **15**, 1652–1671 (2019).

15. Adamo, C. & Barone, V. Toward reliable density functional methods without adjustable parameters: The PBE0 model. *J. Chem. Phys.* **110**, 6158–6170 (1999).

16. Zhao, Y. & Truhlar, D. G. The M06 suite of density functionals for main group thermochemistry, thermochemical kinetics, noncovalent interactions, excited states, and transition elements: two new functionals and systematic testing of four M06-class functionals and 12 other functionals. *Theor. Chem. Acc.* **120**, 215–241 (2008).

17. Weigend, F. & Ahlrichs, R. Balanced basis sets of split valence, triple zeta valence and quadruple zeta valence quality for H to Rn: Design and assessment of accuracy. *Phys. Chem. Chem. Phys*. **7**, 3297–3305 (2005).

18. Weigend, F. Accurate Coulomb-fitting basis sets for H to Rn. *Phys. Chem. Chem. Phys*. **8**, 1057–1065 (2006).

19. Weigend, F. Hartree–Fock exchange fitting basis sets for H to Rn. *J Comput. Chem.* **29**, 167–175 (2008).

20. Neese, F., Wennmohs, F., Hansen, A. & Becker, U. Efficient, approximate and parallel Hartree–Fock and hybrid DFT calculations. A 'chain-of-spheres' algorithm for the Hartree–Fock exchange. *Chem. Phys*. **356**, 98–109 (2009).

21. Caldeweyher, E., Bannwarth, C. & Grimme, S. Extension of the D3 dispersion coefficient model. *J Chem Phys*. **147**, 034112 (2017).

22. Barone, V. & Cossi, M. Quantum calculation of molecular energies and energy gradients in solution by a conductor solvent model. *J. Phys. Chem. A* **102**, 1995–2001 (1998).
23. Marenich, A. V., Cramer, C. J. & Truhlar, D. G. Universal solvation model based on solute electron density and on a continuum model of the solvent defined by the bulk dielectric constant and atomic surface tensions. *J. Phys. Chem. B* **113**, 6378–6396 (2009).
24. Grimme, S. Supramolecular binding thermodynamics by dispersion-corrected density functional theory. *Chem. Eur. J.* **18**, 9955–9964 (2012).
25. Alecu, I. M., Zheng, J., Zhao, Y. & Truhlar, D. G. Computational thermochemistry: scale factor databases and scale factors for vibrational frequencies obtained from electronic model chemistries. *J. Chem. Theory Comput.* **6**, 2872–2887 (2010).
26. Lu, T. & Chen, F. Multiwfn: A multifunctional wavefunction analyzer. *J. Comput. Chem.* **33**, 580–592 (2012).
27. Hunter, J. D. Matplotlib: A 2D graphics environment. *Comput. Sci. Eng.* **9**, 90–95 (2007).
28. Johnson, E. R. et al. Revealing noncovalent interactions. *J. Am. Chem. Soc.* **132**, 6498–6506 (2010).
29. Legault, C. Y. CYLview20, Université de Sherbrooke, 2020 (www.cylview.org).
30. Cao, X. et al. Benzodiazepine derivatives as potent vasopressin V_2 receptor antagonists for the treatment of autosomal dominant kidney disease. *J. Med. Chem.* **65**, 9295–9311 (2022).
31. Zhang, H. et al. Long residence time at the vasopressin V_2 receptor translates into superior inhibitory effects in ex vivo and in vivo models of autosomal dominant polycystic kidney disease. *J. Med. Chem.* **65**, 7717–7728 (2022).
32. Li, X.-W. et al. 1-Indanone retards cyst development in ADPKD mouse model by stabilizing tubulin and down-regulating anterograde transport of cilia. *Acta Pharmacol. Sin.* **44**, 406–420 (2023).
33. Trott, O. & Olson, A. J. AutoDock Vina: improving the speed and accuracy of docking with a new scoring function, efficient optimization, and multithreading. *J.*

Comput. Chem. **31**, 455–461 (2010).
