# Supplementary material for: Asymmetric synthesis of P-stereogenic phosphindane oxides via kinetic resolution and their biological activity
Source: Nat Commun. 2024 Mar 21;15:2548. doi: 10.1038/s41467-024-46892-7 (PMC10957969; doi:10.1038/s41467-024-46892-7)
Supplement: Supplementary file 3 — Reporting Summary [file 41467_2024_46892_MOESM3_ESM.pdf]

nature portfolio

Corresponding author(s): Dong Guo, Xiaowei Dou, Mengzhe Guo

Last updated by author(s): Feb 26, 2024

Reporting Summary

Nature Portfolio wishes to improve the reproducibility of the work that we publish. This form provides structure for consistency and transparency in reporting. For further information on Nature Portfolio policies, see our Editorial Policies and the Editorial Policy Checklist.

Statistics

For all statistical analyses, confirm that the following items are present in the figure legend, table legend, main text, or Methods section.

n/a	Confirmed	
☐	☒	The exact sample size (*n*) for each experimental group/condition, given as a discrete number and unit of measurement
☐	☒	A statement on whether measurements were taken from distinct samples or whether the same sample was measured repeatedly
☐	☒	The statistical test(s) used AND whether they are one- or two-sided<br>*Only common tests should be described solely by name; describe more complex techniques in the Methods section.*
☒	☐	A description of all covariates tested
☐	☒	A description of any assumptions or corrections, such as tests of normality and adjustment for multiple comparisons
☐	☒	A full description of the statistical parameters including central tendency (e.g. means) or other basic estimates (e.g. regression coefficient) AND variation (e.g. standard deviation) or associated estimates of uncertainty (e.g. confidence intervals)
☐	☒	For null hypothesis testing, the test statistic (e.g. *F*, *t*, *r*) with confidence intervals, effect sizes, degrees of freedom and *P* value noted<br>*Give P values as exact values whenever suitable.*
☒	☐	For Bayesian analysis, information on the choice of priors and Markov chain Monte Carlo settings
☐	☒	For hierarchical and complex designs, identification of the appropriate level for tests and full reporting of outcomes
☒	☐	Estimates of effect sizes (e.g. Cohen's *d*, Pearson's *r*), indicating how they were calculated

Our web collection on statistics for biologists contains articles on many of the points above.

Software and code

Policy information about availability of computer code

Data collection	NMR spectra were recorded on JNM-ECZ400S. Enantiomeric excesses (ee) were determined by HPLC analysis on Shimadzu HPLC system with Daicel chiral columns. Optical rotations were measured on an Anton Paar MCP 100 automatic polarimeter. High resolution mass spectra (HRMS) were performed on Waters XEVO G2-S TOF or Agilent G6550A Q-TOF (ESI). Single crystal X-ray diffraction were obtained on a Bruker D8 Quest diffractometer. In the proteomic analysis, MS data was collected by Thermo Scientific Xcalibur 4.4. In the molecular docking analysis, Sybyle 6.9 and AutoDockTools 1.5.6 were used to construct and minimize ligands. In the DFT calculations, ORCA 5.0.4 was used to optimize structures and calculate the single point energies and solvation energies.
Data analysis	HPLC data analyzed using the software LabSolutions™ (version 5.90 LE). The structures of single crystal X-ray diffraction were solved by direct methods using Olex2 software. Peptide samples were analyzed by an EASY-nLC 1200 LC system coupled with Orbitrap mass spectrometry (Thermo Fisher). MS database search were performed by DIA-NN. Molecular docking was displayed using AutoDock v4.2.6 software. Pymol v2.3 was used to show the interaction modes of complex. GraphPad Prism 8.0 was used for statistical analysis. In the proteomic analysis, MS database search were performed by DIA-NN 1.8.1. The raw data was converted to mzML format by MSConvertGUI (64-bit). In the DFT calculations, Multiwfn 3.8 (dev) was used to preform the non-covalent interaction analysis, and CYLView20 and Python package Matplotlib 3.5.2 were used to visualization.

For manuscripts utilizing custom algorithms or software that are central to the research but not yet described in published literature, software must be made available to editors and reviewers. We strongly encourage code deposition in a community repository (e.g. GitHub). See the Nature Portfolio guidelines for submitting code & software for further information.

Data

Policy information about availability of data

All manuscripts must include a data availability statement. This statement should provide the following information, where applicable:

- Accession codes, unique identifiers, or web links for publicly available datasets
- A description of any restrictions on data availability
- For clinical datasets or third party data, please ensure that the statement adheres to our policy

All data generated and analyzed in this study can be found in the article and its Supplementary Information. The X-ray crystallographic coordinates for structures reported in this study have been deposited at the Cambridge Crystallographic Data Centre (CCDC), under deposition numbers CCDC 2246799 (3aa), CCDC 2246797 ((R)-1a), and CCDC 2246811 (3ak). These data can be obtained free of charge from the Cambridge Crystallographic Data Centre via www.ccdc.cam.ac.uk/data_request/cif. The crystal structure of tubulin [PDB ID: 6EW0, https://www.rcsb.org/structure/6EW0] was obtained from Protein Data Bank. The mass spectrometry proteomics data have been deposited to the ProteomeXchange Consortium via the PRIDE partner repository with the dataset identifier PXD049792 [https://proteomecentral.proteomexchange.org/cgi/GetDataset?ID=PXD049792]. The supplementary figure 13a that support the findings of this study is available from BioRender, and was used under licence (agreement number: LU26HODQYI) for this study. The source data underlying Figures 5a-d, and Supplementary Figures 10-12 are provided as a source data file. Data related to materials and methods, optimization of conditions, experimental procedures, mechanistic experiments, and spectra are provided in the Supplementary Information. All data are available from the corresponding authors upon request. Source data are provided with this paper.

Research involving human participants, their data, or biological material

Policy information about studies with human participants or human data. See also policy information about sex, gender (identity/presentation), and sexual orientation and race, ethnicity and racism.

Reporting on sex and gender	No human participants, their data, or biological material are involved.
Reporting on race, ethnicity, or other socially relevant groupings	see above
Population characteristics	see above
Recruitment	see above
Ethics oversight	see above

Note that full information on the approval of the study protocol must also be provided in the manuscript.

Field-specific reporting

Please select the one below that is the best fit for your research. If you are not sure, read the appropriate sections before making your selection.

☒ Life sciences ☐ Behavioural & social sciences ☐ Ecological, evolutionary & environmental sciences

For a reference copy of the document with all sections, see nature.com/documents/nr-reporting-summary-flat.pdf

Life sciences study design

All studies must disclose on these points even when the disclosure is negative.

Sample size	Sample sizes were chosen according to similar studies reported in the literature. For animal studies (Figure 5d), six mice per genotype or treatment group were used in independent experiments. To minimize any potential bias, we randomly assigned samples of each group. Based on the significant phenotype in the experiments, all of experiment results detected a statistically significant difference P < 0.05. For animal studies (Supplementary Figure 12), five mice per genotype or treatment group were used in independent experiments. To minimize any potential bias, we randomly assigned samples of each group. Based on the significant phenotype in the experiments, all of experiment results detected a statistically significant difference P < 0.05.<br>Figure 5a: Four compounds were chosen to colony formation. n = 3 for each group was chosen to allow one-way ANOVA followed by Dunnett's post-test.<br>Figure 5b: Two different concentrations of 3az compounds were used to MDCK cyst model. n=3 for each group was chosen to allow two-way ANOVA followed by Dunnett's post-test.<br>Figure 5c: n = 3 for each group was chosen to allow two-sided student's t test.<br>Figure 5d: n = 6 for each group was chosen to allow two-sided student's t test.<br>Supplementary Figure 10: n = 3 for each group was chosen to allow two-way ANOVA followed by Dunnett's post-test.<br>Supplementary Figure 11: Two different compounds were chosen to embryonic kidney cyst model. n = 3 for group was chosen to allow one-way ANOVA followed by Dunnett's post-test.<br>Supplementary Figure 12: n = 5 for each group was chosen to allow two-sided student's test.
Data exclusions	No data were excluded from the manuscript.

Replication	For each assessment/measurement, similar results were consistently obtained in multiple independent experiments with different biological replicates, and all experiments are reported in the manuscript.
Randomization	For all animal experiments, mice of the same age and genotype were mixed and then randomly grouped for subsequent analyses.
Blinding	For animal experiments, investigators were not blinded to the experiments. However, we followed standard laboratory procedures of randomization. Each experiment was associated with proper controls, and compared samples were collected and analyzed under the same conditions. The experimental observations would be consistent irrespective of blinding. Conclusions were based on independent experiments, quantitative parameters and statistical significance of the data.

Reporting for specific materials, systems and methods

We require information from authors about some types of materials, experimental systems and methods used in many studies. Here, indicate whether each material, system or method listed is relevant to your study. If you are not sure if a list item applies to your research, read the appropriate section before selecting a response.

Materials & experimental systems

n/a	Involved in the study
☒	☐ Antibodies
☐	☒ Eukaryotic cell lines
☒	☐ Palaeontology and archaeology
☐	☒ Animals and other organisms
☒	☐ Clinical data
☒	☐ Dual use research of concern
☒	☐ Plants

Methods

n/a	Involved in the study
☒	☐ ChIP-seq
☒	☐ Flow cytometry
☒	☐ MRI-based neuroimaging

Eukaryotic cell lines

Policy information about cell lines and Sex and Gender in Research

Cell line source(s)	Madin-Darby canine kidney (MDCK) cells were originally obtained from National Collection of Authenticated Cell Cultures (catalog#GNO23).
Authentication	Negative
Mycoplasma contamination	The cell line has been tested with no mycoplasma contamination
Commonly misidentified lines (See ICLAC register)	No commonly misdentified lines were used in this study

Animals and other research organisms

Policy information about studies involving animals; ARRIVE guidelines recommended for reporting animal research, and Sex and Gender in Research

Laboratory animals	Mice were maintained on the C57BL/6 background. All mice used in this study were 12 days old. Both male and female mice were used in autosomal dominant polycystic kidney disease (ADPKD) model. All mice were kept in the animal facility at Xuzhou Medical University under a 12/12-h light/dark cycle and up to five animals per cage. The ambient temperature and humidity of the laboratory is controlled at 18-24 degree and 50%-70%, with access to food and water ad libitum. The Laboratory Animal Ethics Committee of Xuzhou Medical University approved all experiments involving animals.
Wild animals	Wild animals are not involved in this study.
Reporting on sex	Both male and female mice were used in autosomal dominant polycystic kidney disease (ADPKD) model. For ADPKD mouse experiments as close to a 50:50 sex split as possible was performed.
Field-collected samples	The study did not involve samples collected from the field.
Ethics oversight	Animal experiments were approved and conducted in accordance with the Laboratory Animal Ethics Committee of Xuzhou Medical University (202309T012).

Note that full information on the approval of the study protocol must also be provided in the manuscript.

Plants

Seed stocks	N/A
Novel plant genotypes	N/A
Authentication	N/A
